# Second International Meeting of ISEV 2013: Boston, USA, April 17^th^-20^th^, 2013

**DOI:** 10.3402/jev.v2i0.20826

**Published:** 2013-04-15

## Scientific Program 2013 ISEV meeting Wednesday 17th April

**Opening Session (Imperial Ballroom) 8:00-8:30**

**8:00-8:30 Opening of ISEV-2013, Welcome, Year in review.** *J. Lötvall and F. Hochberg*

**Parallel Oral Sessions 1-3 8:45-10:00**

**Oral Session 1 (Imperial Ballroom): Biomarkers: Urinary Tract April 17**

**Chair: G. Camussi and J.M. Falcon-Perez 8:45-10:00**

**Markers of inflammation, tissue damage and regeneration in urinary microvesicles from transplanted patients**

B. Bussolati, V. Dimuccio, A. Moggio, E. Basso, A. Ranghino and G. Camussi

University of Torino, Turin, Italy

*Introduction and aim*: Urinary microvesicles are mainly derived from cells of the nephron, and they are an easily available source of information about the renal condition. In this study, we evaluated the expression of markers of inflammation and tissue damage by microvesicles in the urine of kidney-transplanted patients. Moreover, exosome markers and content were compared with those of renal resident stem cells, as a marker of renal regeneration. *Methods*: Urinary microvesicles were isolated from 11 control-transplanted patients, 9 patients with slow graft function (SGF, defined as creatinine level>3 mg/dL on day 5 post-transplant) and 10 matched healthy subjects. Microvesicles were obtained by ultra-centrifugation from the second fresh morning urine (collected 1 and 7 days after the transplant) or from the overnight supernatant of cultured CD133+ renal stem cells. Cytofluorimeter analysis was performed after adsorption on 4 µm aldehyde–sulphate latex beads. MicroRNA was isolated using mirVana kit and analysed by quantitative RT-PCR. *Results*: A panel of several markers was tested on urinary microvesicles. CD45 and CD56 were used as markers of inflammation and VEGFR2 as marker of endothelial damage. At day 1 after transplant, CD45, CD56 and VEGFR2 were higher in transplanted patients than in normal subjects. Moreover, CD45 and CD56 were higher in SGF patients with respect to controls. At day 7, CD45, CD56 and VEGFR2 were found decreased in control patients and returned to basal levels. In contrast, no decrease was observed in SGF patients, who maintained higher values. Finally, at day 7, an increase in surface markers and microRNA detected in renal stem cell microvesicles were observed in both control and SGF patients. *Conclusions*: These findings suggest that markers of inflammatory cells of endothelial damage and of cell regeneration can be detected in the urinary microvesicles of transplanted patients, suggesting an interesting diagnostic application.

**Urine exosome proteins to identify metastatic prostate cancer patients**

I. Bijnsdorp^1^, A.A. Geldof^1^, S.R. Piersma^2^, M. Lavalei^2^, R.J.A. Van Moorselaar^1^ and C.R. Jimenez^2^

^1^Department of Urology, VU University Medical Center, The Netherlands; ^2^OncoProteomics Laboratory, Department of Medical Oncology, The Netherlands

*Introduction*: The incidence of early-stage prostate cancer (PrCa) is increasing each year. Yet only a fraction of these patients will eventually develop aggressive disease and require therapy. To discriminate indolent from aggressive cancer, definitive markers are required. Exosomes are released by prostate (cancer) cells and contain (tumour-associated) proteins and can be found in the urine, of which the collection is non-invasive. Therefore, these urine exosomes are an excellent source for biomarker discovery. *Materials and methods*: We have measured the protein profile (LC-MS/MS) of exosomes from two PrCa cell lines (LNCaP and PC-3 cells), and the resulting protein profile and associated pathways were evaluated (David and String databases). Functionality of these proteins was determined by the effect of migration and invasion (Transwell assays) on non-cancerous prostate cells. Protein expression was determined by Western blotting. *Results*: The protein profile in exosomes of the PrCa cell lines contained different integrin proteins, involved in migration and invasion processes. Medium containing exosomes from these PrCa cell lines increased the migration and invasion capacity of non-cancerous prostate cells. Antibodies directed against the identified integrins reduced the migration/invasion back to control levels, indicating an active functioning of these exosomal integrins. The expression of the integrins in urine exosomes of patients suffering from metastatic PrCa was significantly higher (p<0.05) compared to expression in patients with benign prostate hyperplasia or localised PrCa. Future prospective studies will focus on larger patient groups and prognostic markers. *Conclusion*: We identified proteins involved in PrCa metastasis, which are highly expressed in urine exosomes of patients with metastatic PrCa. These proteins might be used for risk stratification of PrCa metastasis.

**Urinary extracellular vesicles in kidney stone formers**

M. Jayachandran, G.A. Lugo, H. Heiling, A.D. Rule and J.A. Lieske

Mayo Clinic, Scottsdale, AZ, USA

*Introduction*: Kidney stones commonly affect 10–12% of the general population, but the cellular mechanisms of their formation remain unknown. Activated and injured tubular cells shed extracellular vesicles (microvesicles and exosomes) into urine that may serve as biomarkers for stone formation. *Materials and methods*: Urinary extracellular vesicles were characterised in 24-h urine collections from persons presenting with their first kidney stone (n=128, mean age 44 years, 61% men) and controls (n=61, mean age 47 years, 59% men). Vesicles were captured by high-speed centrifugation, washed, characterised by flow cytometry and transmission electron microscopy (TEM) and lysed for protein array. *Results*: TEM revealed a heterogeneous population of urinary vesicles <1 µm. In stone formers, compared to controls, the number of vesicles containing phosphatidylserine (annexin-V), CD9 and CD63 (exosomal markers), podocalyxin (tubular cell or podocyte marker) and tissue factor (inflammatory marker) were significantly higher, while the number containing lymphocyte antigen 75 (proximal convoluted tubule epithelial cells marker) was significantly (p<0.05) lower. There were no differences in CD82-, TSG101-, nephrin-, synaptopodin-, aquaporin-2- and ICAM1-positive vesicles. Lysate content of osteopontin, β-2-microglobulin, cystatin C and neutrophil gelatinase-associated lipocalin were increased in stone formers compared to controls, while albumin and clusterin amounts were similar. *Conclusion*: Studies suggest that kidney cells can be injured by high concentrations of lithogenic molecules, such as oxalate as well as urinary crystals. Populations of renal-cell-derived urinary vesicles differ between first-time kidney stone formers and controls. These differences may reflect renal cell activation, provide clues regarding cellular pathogenic events during kidney stone formation and could potentially be monitored to assess disease activity.

**Exosomes secreted under hypoxia enhance invasiveness in prostate cancer cells**

G. Deep, A. Ramteke, S. Mateen, C. Agarwal, B.A. Frederick, M. Graner and R. Agarwal

University of Colorado Denver, Denver, CO, USA

*Introduction*: Hypoxic condition in prostate cancer (PCA) is associated with worse prognosis; however, the precise mechanism through which hypoxia induces malignant phenotype remains unclear. Here, we analysed the role of exosomes (Exo) secreted under hypoxia in conferring invasive phenotype to PCA cells. *Methods*: Human PCA LNCaP cells were exposed to hypoxic (1% O_2_) or normoxic (20% O_2_) conditions. Media was collected and Exo, secreted under hypoxic (ExoHypo) and normoxic conditions (ExoNorm), were isolated by ultracentrifugation and precipitation (ExoQuick) methods. ExoHypo and ExoNorm were characterised by Nanosight nanoparticle tracking analysis (NTA) and electron microscopy (EM). Invasiveness of LNCaP cells was analysed in the presence ExoHypo or ExoNorm using Transwells. ExoHypo and ExoNorm were compared for protein expression of many signalling molecules by immunoblotting and miRNAs expression using an array. *Results*: NTA and EM analyses confirmed the size/structure of exosomes. Co-culturing of naïve LNCaP cells with ExoHypo enhanced their invasiveness by 4-fold (p≤0.05) compared to ExoNorm. Immunoblotting showed that ExoHypo has significantly higher level of tetraspanins (CD63 and CD81), heat-shock proteins (HSP90 and HSP70) and Annexin II. Importantly, the expression of adheren junction proteins E-cadherin and β-catenin was decreased in LNCaP cells under hypoxia, while ExoHypo showed extremely low amounts of E-cadherin but high amounts of β-catenin and N-cadherin. ExoHypo also showed higher level of cytokines (TGFβ2, TNFα, IL6, VEGF and PDGF) and MMPs (2, 3 and 9). Zymography confirmed higher MMP activity in ExoHypo. Array analysis for 377 miRNAs showed that ExoHypo have 15 miRNAs with >1000-fold higher and 30 miRNAs with >90% lower expression compared to ExoNorm. *Conclusions*: Together, these results showed that exosomes secreted under hypoxic conditions are loaded with unique signalling molecules and miRNAs that confer enhanced invasiveness to PCA cells.

**Identification of microRNA biomarkers derived from the epididymis in human seminal microvesicles**

C. Belleannée, C. Légaré, E. Calvo and R. Sullivan

CRCHUQ/Université Laval, Quebec, Canada

*Introduction*: The epididymis is a long convoluted tubule that connects the testis to the vas deferens and is responsible for the acquisition of sperm fertilising ability. While dysfunction of this organ leads to male infertility, there is no proper non-invasive biomarker to diagnose it. Seminal plasma contains seminal microvesicles (SMVs) derived from the epididymis (epididymosomes) and accessory glands (mostly prostasomes). Considering that SMVs convey microRNAs (miRNAs), we aimed to characterise SMVs-miRNAs derived from the epididymis and evaluate their potential as non-invasive biomarkers of impaired sperm maturation. *Materials and methods*: Vasectomy prevents spermatozoa and epididymosomes from being released into seminal plasma and can be reversed by vasovasostomy surgery. Since SMVs from seminal plasma of vasectomised men are devoid of epididymosomes, these samples were used to assess the contribution of epididymosomes to SMV-miRNA repertoire. SMV-miRNA signatures from normospermic, vasectomised and vasovasostomised donors were determined by microarray. Identification of epididymosomal miRNAs was obtained after comparison of these arrays with miRNA signature from human epididymal tissues by using the Partek software. Results were confirmed by real-time PCR. *Results*: We identified 18 miRNAs exclusively found in SMVs from normospermic and vasovasostomised donors. Absence of these miRNAs in SMVs from vasectomised donors suggested that they originate from the epididymis and are shuttled to the seminal plasma via epididymosomes. Comparison with miRNAs from human epididymal tissues confirmed their tissue origin. Of relevance, miR-888 cluster, which is predicted to regulate sperm-associated proteins important for sperm maturation, is highly expressed in SMVs. *Conclusion*: In humans, a specific subset of SMV-miRNAs are derived from the epididymis and may be used as non-invasive biomarkers to determine the aetiology of male infertility cases related to impaired sperm maturation.

**Oral Session 2 (Plaza Ballroom): EV biogenesis April 17**

**Chair: *S. Gould and P. Zimmermann* 8:45-10:00**

**Analysis of ESCRTS functions in exosome biogenesis, composition and secretion reveals a role for ALIX in coordinating MHC class II trafficking**

M. Colombo ^1,2^, C. Moita^3^, G. van Niel^2^, J. Kowal^1^, J. Vigneron^1^, P. Benaroch^1^, N. Manel^1^, L. Ferreira Moita^3^, C. Théry^1^ and G. Raposo^2^

^1^INSERM U932, Institut Curie, Paris, France; ^2^UMR144, Institut Curie, Paris, France; ^3^Cell Biology of the Immune System Unit, Instituto de Medicina Molecular, Lisboa, Portugal

*Introduction*: As opposed to other extracellular vesicles (EVs), which bud directly off the plasma membrane, exosomes are secreted on fusion of multivesicular endosomes (MVE) with the plasma membrane. The mechanisms involved in exosome biogenesis remain unclear so far. Given the known role of the ESCRT machinery in the formation of internal vesicles of MVE, we studied its role in exosome secretion. *Materials and methods*: We performed an RNA interference screen targeting individual components of the ESCRT machinery in HeLa-CTIIA cells expressing MHC class II molecules; since CD63 accumulates on internal vesicles of MVEs, we quantified exosome secretion as released MHC II- and/or CD81-positive vesicles captured by anti-CD63-coated beads. Exosomes were also purified by differential ultracentrifugation and characterised by Western blotting and immuno-electron microscopy (IEM). *Results*: The results of the screen indicated that secretion of exosomes was decreased upon silencing of HRS, STAM, TSG101, but increased upon CHMP4C, ALIX, VPS4B depletion. Measurement of vesicle size and MHC II versus CD63 content by IEM unravels differential effects of depleting the ESCRT proteins on these features. In particular, ALIX depletion induced a specific increase in MHC class II content of exosomes from HeLa-CIITA, due to a general increase in cellular MHC class II. Interestingly, ALIX depletion in human dendritic cells also increased the MHC class II content of the cells, but not of the released vesicles, which we attributed to a stronger size heterogeneity, and higher MHC II and lower CD63 contents in EVs recovered from DCs as compared to HeLa-CIITA. *Conclusions*: The results reveal a role of selected ESCRT components in exosome secretion and composition by HeLa-CIITA, in addition to a more generic role of ALIX in MHC class II trafficking. They also highlight biogenetic differences in vesicles secreted by different cell types, namely a tumour cell line and primary DCs.

**Secretory mechanism and functions of senescence-associated exosomes**

H. Tahara, M. Okada, M. Muneoka and A. Nakamura

Department of Cellular and Molecular Biology, Institute of Biomedical & Health Sciences, Hiroshima University, Hiroshima, Japan

Exosomes are specialised small membranous vesicles and are released into the extracellular environment from a variety of cells, including tumour cells, dendritic cells, lymphoid cells and macrophage cells, and these secreted exosomes can be used for cell-to-cell communication. One of the recent exciting findings is that miRNAs exist in exosomes, and exosome-mediated miRNA transfer is an important mechanism of intercellular communication. We have been focusing on microRNA and exosomes in ageing and cancer and found that senescence-associated miRNAs, SA-miRNAs, are involved in the regulation of the senescence program in both normal and cancer cells and play a major role in tumour suppression. We found that miR-22, a SA-miRNA, is down-regulated in various cancer cell lines but up-regulated in senescent human fibroblasts and epithelial cells. Interestingly, we found that significant level of exosomes were secreted from senescent cells, but exosomes containing SA-miRNAs such as miR-22, miR-34a and miR-30a are quite low particles in senescent cells. To clarify the mechanism of exosome secretion in senescent cells, we screened exosome regulatory genes using siRNAs and found that knockdown of one candidate gene significantly reduced exosome secretion from senescent cells. In addition, this gene is up-regulated in senescent cells, and down-regulated in some cancer cells, suggesting that it plays a role not only in exosome secretion but also in tumour suppression. We also examined the particle size of vesicles using qNano equipment and protein amounts of exosome-membrane-associated proteins and found that exosome-membrane-associated proteins are altered during cellular senescence. We would like to discuss the function and regulation of senescence-associated exosomes in ageing and cancer.

**Syntenin exosome is regulated by small GTPases and requires specific phospholipid-modifying enzymes**

Pascale Zimmermann ^1^, R. Ghossoub^2^, F. Lembo^2^, C. Baron-Gaillard^1^ and S. Audebert^2^

^1^Inserm-CRCM/K.U.Leuven, Leuven, France; ^2^Inserm-CRCM, Marseille, France

*Introduction*: Exosomes are small vesicles of endosomal origin secreted by numerous cell types, including immune, epithelial and tumour cells. Exosomes can deliver functional cargo to target cells and are considered as extracellular vehicles by which cells can communicate. While tremendous investments are made to characterise exosomes from various sources and to establish their biological effects, relatively little is known about the molecular mechanisms that govern exosome biogenesis. We recently showed that syntenin together with syndecan can boost exosome production pending on ALIX interaction. Here we addressed the nature of the upstream regulators and downstream effectors of “syntenin exosomes”. *Materials and methods*: We used a combination of proteomics, silencing and rescue experiments, ultracentrifugation assays, and fluorescence and electron microscopy experiments. *Results*: We identified an unsuspected role for a small GTPase and a specific lipid-modifying enzyme in the production of exosomes and show that they control the formation of the intraluminal vesicles of multivesicular bodies. *Conclusions*: Our study provides novel insights on the mechanisms controlling the biogenesis of exosomes.

**Francisella TOL/PAL system, including FTN_0356, is involved in outer membrane vesicle and biofilm formation**

T. Pierson^1^, T.L. McNealy^2^ and M. van Hoek ^1^

^1^George Mason University, Fairfax, VA, USA; ^2^Clemson University, Clemson, SC, USA

Outer membrane vesicles (OMVs) are small outer membrane blebs constitutively produced by many Gram-negative bacteria, including Francisella. Francisella OMVs have been used in preliminary vaccine studies and contain many proteins associated with virulence. Little is known about OMV regulation in Francisella and their potential role in biofilm production and virulence. OMV production can be enhanced by factors that increase membrane instability, such as high salt, antimicrobial peptides or mutations in the Tol/Pal system. We examined Tol/Pal transposon mutants in *F. novicida* and identified several OMV overproducers, including FTN_0356, which resulted in increased in vitro biofilm production compared to wild-type *F. novicida*. Proteomic analysis of wild-type and Tol/Pal mutant OMVs demonstrated differential proteomic profiles, especially for some key virulence-associated proteins. Using the in vivo model system, *Galleria mellonella*, we observed an increase in virulence in only one of eight Tol/Pal transposon mutants. Treatment of Francisella bacteria with antimicrobial peptides (human-beta defensin 2 (hBD2) and hBD3, the human cathelicidin LL-37 and colistin) as well as growth under high salt conditions increased the production of OMVs in Francisella. Thus, membrane-destabilising treatments, such as high salt, antimicrobial peptides or Tol/Pal mutants, result in increased Francisella OMV production as well as increased biofilm production.

**The lipid flippase TAT-5 inhibits the budding of extracellular vesicles from the surface of C. elegans embryos**

Ann Wehman ^1^, B.D. Grant^2^ and J. Nance^3^

^1^Rudolf-Virchow-Zentrum, Würzburg, Germany; ^2^Rutgers University, Newark, NJ, USA; ^3^Skirball Institute, NYU School of Medicine, New York, NY, USA

*Introduction*: Cells release extracellular vesicles (ECVs) that can influence differentiation, modulate the immune response, promote coagulation and induce metastasis. Many ECVs form by budding outwards from the plasma membrane, but the molecules that regulate budding are unknown. In ECVs, the outer leaflet of the membrane bilayer contains phospholipids that are normally sequestered to the inner leaflet of the plasma membrane, suggesting a potential role for lipid asymmetry in ECV budding. *Results*: We show that loss of the conserved lipid flippase TAT-5 causes the large-scale shedding of ECVs and disrupts cell adhesion and morphogenesis in *C. elegans* embryos. ECVs accumulate between cells disrupting the structure of cell–cell contacts. TAT-5 localises to the plasma membrane and its loss results in phosphatidylethanolamine (PE) exposure on cell surfaces, suggesting that TAT-5 maintains plasma membrane PE asymmetry. Since viruses also bud from the surface of cells, we tested whether viral budding regulators also regulate ECV budding. We show that RAB-11 and endosomal sorting complex required for transport (ESCRT) proteins are enriched at the plasma membrane in TAT-5 mutants and are required for ECV production. *Conclusions*: TAT-5 provides a molecular link between loss of PE asymmetry and the dynamic budding of vesicles from the plasma membrane, supporting the hypothesis that lipid asymmetry regulates budding. Our results also suggest that viral budding and ECV budding may share common molecular mechanisms.

**Oral Session 3 (Georgian): Parasites and Fungi April 17**

**Chair: *H.A. del Portillo and J.M. Silverman* 8:45-10:00**

***Trypanosoma cruzi*: trypomastigotes secrete vesicles with proinflammatory virulence factors that enhance cell invasion**

Ana Claudia Torrecilhas ^1^, Ernesto S. Nakayasu^2^, Catherine Ropert^3^, Ricardo T. Gazzinelli^3^, Walter Colli^4^, Maria Julia Manso Alves^4^ and Igor Correia de Almeida^2^

^1^Universidade Federal de São Paulo, São Paulo, Brazil; ^2^The University of Texas at El Paso, El Paso, USA; ^3^Universidade Federal de Minas Gerais, Belo Horizonte, USA; ^4^Universidade de São Paulo, São Paulo, Brazil

*Introduction*: The surface of *Trypanosoma cruzi* is heavily coated by glycosylphosphatidylinositol-anchored molecules (i.e. mucins, trans-sialidase (TS)/gp85 glycoproteins and glycoinositolphospholipids) that strongly stimulate the host innate immunity via Toll-like receptor (TLR)-dependent pathways. One potential mechanism involves the continuous shedding of membrane-bound vesicles by host cell-derived trypomastigotes (Ves). *Materials and methods*: We fractionated Ves by gel-exclusion chromatography using a Sepharose CL-4B column. Using proteomic analysis, Ves was digested with three different proteolytic strategies - TU, TG and TM to LC-MS/MS analysis. Supernatants were collected for cytokine measurements by ELISA from murine peritoneal macrophages, C3He/HeJ and TLR2-KO mice. *Results*: Pre-treatment of BALB/c mice with Ves, followed by parasite challenge, could significantly exacerbate parasite load and inflammation of the heart and hasten animal mortality. Ves induced in vitro high levels of proinflammatory cytokines and nitric oxide by murine C3He/HeJ-derived macrophages, and considerably increased invasion of host cells. Using macrophages derived from TLR2-knockout mice, we clearly demonstrated that both proinflammatory response and host-cell invasion-enhancing activity were mediated by a TLR2-dependent pathway. Proteomic analysis of Ves by liquid chromatography-tandem mass spectrometry (LC-MS/MS) revealed 110 *T. cruzi*-specific proteins, of which over half (55%) were trans-sialidase/gp85 glycoproteins, which are well-established virulence factors. *Conclusion*: We conclude that *T. cruzi* trypomastigotes release vesicles as virulence factors to facilitate the parasite invasion of the host cell, a phenomenon that is compensated by the potent host proinflammatory response triggered by these vesicles.

**Biogenesis and biological activity of fungal extracellular vesicles**

Marcio Rodrigues

Fundação Oswaldo Cruz – Fiocruz, Centro de Desenvolvimento Tecnológico em Saúde (CDTS) and bInstituto de Microbiologia, Universidade Federal do Rio de Janeiro, Rio de Janeiro, Brazil

Several microbial molecules are released to the extracellular space in vesicle-like structures. In pathogenic fungi, these molecules include pigments, polysaccharides, lipids and proteins, which traverse the cell wall in vesicles that accumulate in the extracellular space. The diverse composition of fungal extracellular vesicles (EV) is indicative of multiple mechanisms of cellular biogenesis. Proteomics of fungal EV revealed the presence of molecules with both immunologic and pathogenic activities. In fact, fungal EV have been demonstrated to interfere with the activity of immune effector cells and to increase fungal pathogenesis. The aim of this session is to discuss the functions and biogenesis of fungal EV, as well as the potential role of these structures in fungal pathogenesis.

**Vesicle-associated small RNAs secreted by the gastrointestinal nematode *Heligmosomoides polygyrus***

Amy Buck, F. Simbari, A. Ceroni, G. Coakley, S. Kumar, K. Smith, Y. Harcus, M. Lear, A. Ivens, M. Blaxter and R Maizels

University of Edinburgh, United Kingdom

*Introduction*: The secretion and subsequent uptake of vesicular bodies provides a mechanism of cell-to-cell communication and enables RNA transport in mammals. Here, we examined whether the gastrointestinal nematode parasite, *Heligmosomoides polygyrus*, secretes RNAs during its residence in the mouse small intestine. *Materials and methods*: Secretion product was collected from adult worms harvested at 14 days post-infection. Small RNA libraries were prepared from the secretion product as well as the eggs, infective larvae and duodenal adults. FACs and confocal and electron microscopies were used to examine uptake properties of the parasite exosomes by host epithelial cells. *Results*: From the 46.2 million reads analysed, a total of unique 658 pre-miRNAs were identified. The most highly expressed miRNAs are conserved in other nematodes and display development-specific expression patterns similar to those reported in *C. elegans*. In the secretion product, 145 miRNAs were present at a frequency of <10 reads/million. Several of the most abundant miRNAs have homologous seed sites to mouse miRNAs associated with regulation of inflammatory responses. The secreted miRNAs are stabilised against degradation by RNases and associate with structures that pellet upon ultracentrifugation. Transmission electron microscopy reveals vesicle-like structures of 50–100 nm in the ultracentrifugation pellet. Flow cytometry and confocal microscopy analyses suggest that these vesicles are taken up by small intestinal cells and transfer parasitic RNAs to the host cell. *Conclusion*: This work suggests that RNA secretion by parasitic nematodes could mediate cross-species communication and may enable manipulation of host cell inflammatory responses by parasites.

**Proteomic analysis of Leishmania exosomes**

B.K. Singh, J.L. Weirather, P.H. Kelly, R.M. Pope and M.E. Wilson

The University of Iowa, University Heights, IA, USA

*Introduction*: *Leishmania infantum chagasi* (*Lic*), the protozoan parasite, causes human visceral leishmaniasis. Many of the excreted/secreted proteins of *Leishmania* are released in exosomes. Released proteins include virulence factors such as the metalloprotease GP63. *Leishmania* assumes an extracellular promastigote form in the sand fly vector, delivered into the host as an infectious metacyclic promastigote and intracellular amastigote in the mammalian host. Earlier studies characterised *Leishmania* exosome from promastigotes. Herein, we compared the exosomes of avirulent logarithmic promastigotes, virulent stationary promastigotes, purified metacyclic promastigotes and axenic amastigotes. *Materials and methods*: Exosomes were isolated using differential centrifugation and purified on linear sucrose gradients. Exosome presence was characterised by western blots, scanning electron microscopy and protease gels. Proteomic analysis was performed using liquid chromatography-mass spectrometry (LC-MS). *Results*: Eight hundred and fifteen unique exosomal proteins were identified, including the reported *Leishmania* exosome proteins EF1-α, GP63, HSP70, HSP90, Sti1 and LACK. Comparative analysis revealed 141 proteins common to all exosomes and several stage-specific exosomal proteins. Exo-proteins of logarithmic, stationary and metacyclic promastigotes included EF1-α, trypanothione reductase, trypanothione peroxidase and tryparedoxin, whereas exosomes from amastigotes contain amastin, iron superoxide dismutase, isocitrate dehydrogenase and UCH37/UCHL5. The metalloprotease MSP (GP63) was present in abundance in stationary and metacyclic promastigote exosomes. Protease gels confirmed that dominant exosomal protease was a metalloprotease. *Conclusions*: Proteomic evaluation identifies major differences between the exosomes of the different *Lic* life stages. We hypothesise that specific exosome contents facilitate *Leishmania* survival at all steps of infection.

**Composition and immunomodulatory effects of extracellular vesicles released by *Candida albicans***

L. Nimrichter ^1^, G. Vargas^2^, J. Dutra^3^, E.S. Nakayasu^4^, T.J. Sobreira^5^, I.C. Almeida^6^, E.A. Arigi^6^, J.D. Nosanchuk^7^, S. Frases^3^, C.G. Freire-de-Lima^3^ and M.L. Rodrigues^8^

^1^Instituto de Microbiologia, Universidade Federal do Rio de Janeiro, Rio de Janeiro, Brazil; ^2^Instituto de Microbiologia, Universidade Federal do Rio de Janeiro, Rio de Janeiro, Brazil; ^3^Instituto de Biofísica, Universidade Federal do Rio de Janeiro, Rio de Janeiro, Brazil; ^4^Pacific Northwest National Laboratory, Richland, USA; ^5^Instituto do Coração, São Paulo, Brazil; ^6^Department of Biological Sciences, University of Texas, El Paso, USA; ^7^Division of Infectious Disease, Department of Medicine, Albert Einstein College of Medicine, New York, USA; ^8^UFRJ and Fiocruz, Brazil

*Introduction*: The release of extracellular vesicles (EV) by fungal organisms is considered an alternative to trans-cell wall passage of macromolecules. Previous studies demonstrated the presence of EV in culture supernatant from *C. albicans*. Here, we analysed their composition, the kinetics of internalisation by macrophages (MO) and dendritic cells (DC), and their immunomodulatory activity. *Materials and methods*: Transmission electron microscopy and light scattering were used to determine morphology and relative size of vesicles. EV content was investigated by LC-MS/MS and TLC analysis. Presence of antigenic proteins and mannoproteins was determined by western blot. EV were labelled with DiIC18, and fluorescence microscopy was used to study their internalisation by host cells. ELISA and Griess reaction were used to measure cytokines and NO production by macrophages (MO) and dendritic cells (DC), incubated with EV. *Results*: Bilayered membranous vesicles ranging from 50 to 850 nm were visualised. Two seroreactive proteins (27 and 37 kDa) were observed. Polydisperse mannoproteins were also detected using concanavalin A. Proteins related with pathogenesis, cell organisation, carbohydrate and lipid metabolism, response to stress and other functions were identified by LC-MS/MS. Ergosterol, lanosterol and glucosylceramide were detected by TLC. EV were internalised by host cells after 15 min, and this process resulted in immunomodulation. Treated macrophages produced higher levels of NO, IL-12, TGF-β and IL-10. Similarly, DC produced IL-12p40, IL-10 and TNF-α. In addition, EV from *C. albicans* induced upregulation of CD86 and MHC-II. *Conclusions*: Our results demonstrate that *C. albicans* EV are immunologically active and could potentially interfere with the course of candidiasis.

Financial support: CNPq, CAPES, FAPERJ and FAPESP.

**Coffee and Poster Viewing April 17**

**Poster Sessions I-II 10:00-13:30**

**Parallel Oral Sessions 4-6 13:30-15:00**

**Oral Session 4 (Imperial Ballroom): Inflammation April 17**

**Chair: *M. Piper and S. Gabrielsson* 13:30-15:00**

**Exosomal-transfer of EBV-polymerase-III transcripts (Eber1) activates a type-1 IFN-mediated inflammatory response associated with autoimmunity**

D.M.P. Pegtel ^1^, M.E.E. van Eijndhoven^1^, D.K.L. Koppers-Lalic^1^, K.H. Heutinck^2^, J.B. Berenguer^1^, M.H. Hackenberg^3^, R.S.B. Bakker^1^, A.E.G. Greijer^1^, S.V. Verkuijlen^1^, J.K. Knol^1^, K.G. Grunberg^1^, T.W. Wurdinger^4^, G.S. Scheffer^1^, K.G. Gelderman, S.L. Lougheed^1^, T.D.G. Gruijl^1^, I.B. Bultink^1^, A.E.V. Voskuyl^1^ and J.M.M. Middeldorp^1^

^1^VU Medical Centre, Amsterdam, The Netherlands; ^2^Academic Medical Centre, Amsterdam, The Netherlands; ^3^University of Granada, Granada, Spain; ^4^VUmc/Harvard, The Netherlands

*Introduction*: Innate detection of nucleic acids and type I interferons (IFNs) induction is fundamental in viral defence. Epstein-Barr virus (EBV) establishes a lifelong infection in more than 90% of healthy human adult population worldwide, in part by employing multiple latency transcription programmes to elude recognition by viral sensors. *Methods/results*: We demonstrate co-culture studies that labelled endosome-derived exosomes, released by latent EBV-infected B-lymphocytes that specifically target tonsillar dendritic cells. By deep sequencing the complete exosomal small RNA content, we identified the incorporation of RNA polymerase-III-transcribed transcripts and demonstrated by gene-expression arrays and ELISA that EBV-EBER1 transfer is sufficient for inducing an IFN-mediated inflammatory response. Label-free quantitative proteomics on these exosomes indicated sorting of proteins belonging to an evolutionary-conserved ribonucleoprotein complex. While exogenous EBER1 delivery can trigger cytosolic- and endosomal RNA sensors into recipient cells, nucleo-cytoplasmic shuttling of EBER1 within infected cells goes unnoticed. Finally, we showed the relevance of EBV infection and its pathogenesis, in vivo. Using sensitive stem-loop RT-PCRs we detected EBER1 transcripts in exosomes isolated from systemic lupus erythematosus patient sera. Additionally we detected exosome-associated EBV small RNAs including EBER1, in lupus nephritis tissues without signs of EBV infection, suggesting transfer by exosomes. EBER1 was neither detected in healthy controls and rheumatoid arthritis patients nor in kidney tissues from appropriate controls. *Conclusions*: Our findings are consistent with EBV-EBER1 release and transfer via exosomes in autoimmune patients that suffer from elevated EBV loads. We reveal a novel host strategy for innate recognition of latent herpes virus infection and suggest that pro-inflammatory exosomes may potentiate autoimmune conditions in genetically predisposed individuals.

**Recipient dendritic cells acquire in vivo donor “intact” MHC molecules via exosomes eliciting the T-cell response that leads to rejection of cardiac allografts**

A.E. Morelli ^1^, Q. Liu^1^, D.M. Rojas-Canales^1^, W.J. Shufesky^1^, D. Beer Stolz^2^, M.L.G. Sullivan^2^, S.C. Watkins^2^ and A.T. Larregina^3^

^1^T.E. Starzl Transplantation Institute, University of Pittsburgh, Pittsburgh, USA; ^2^Department of Cell Biology, University of Pittsburgh, Pittsburgh, USA; ^3^Deparment of Dermatology, University of Pittsburgh, Pittsburgh, USA

*Introduction*: It has been classically assumed that after transplantation, donor (d) dendritic cells (DC) migrate to lymphoid organs and present donor “intact” MHC molecules to T-cells. However, the fact that such T-cells recognise dMHC molecules does not imply that dMHC Ag were presented by dDC. Alternatively, recipient DC (rDC) could acquire intact dMHC molecules released by dDC. We analysed the role of dDC vs. rDC in direct T-cell allo-recognition and the mechanism of transfer of dMHC Ag via exosomes after cardiac transplantation. *Methods*: Bone marrow cells from CD11c-diphteria toxin (DT) receptor (DTR) B6 mice were injected in wt B6 mice to generate CD11c-DTR-B6 chimeras. Isolation of splenic DC, immuno-labelling and IFN-γ ELISPOTs were done using standard methods. *Results*: To test if rDC present “acquired” dMHC Ag to T-cells, BALB/c hearts were grafted in CD11c-DTR-B6 chimeras and rDC were depleted by DT-injection. Under these conditions, T-cells did not respond to intact dMHC molecules (in IFN-γ ELISPOT) on post-operative day (POD) seven, indicating that dMHC molecules were transferred to rDC for T-cell presentation. FACS-analysis confirmed that dMHC molecules were transferred intact to rDC in the spleen. In BALB/c (donor) DC-B6 (acceptor) DC co-cultures, we found that transfer of BALB/c MHC Ag was mediated via exosomes that attached to B6 DC. Chase experiments showed that a % of BALB/c exosomes were internalised by B6 DC. B6 DC with bound BALB/c DC-exosomes presented BALB/c MHC-I H2Dd to 2C CD8 T-cells. To confirm our results in vivo in a cardiac transplantation model, rDC with acquired BALB/c MHC molecules were FACS-sorted from the spleen, 3 days after transplantation of BALB/c hearts. IEM analysis detected clusters of exosomes bearing dMHC molecules on the surface of rDC. *Conclusion*: After cardiac transplantation, rDC of graft-draining lymphoid organs present to T-cells dMHC molecules transferred intact via exosomes from dDC mobilised from the graft.

**Co-delivery of protein and glycolipid antigens by exosomes enhances innate and adaptive antitumour immunity via iNKT cells**

Susanne Gabrielsson, U. Gehrmann, S. Hiltbrunner, A.M. Georgoudaki, M.C.I. Karlsson and T. Näslund

Karolinska Institutet, Stockholm, Sweden

Exosomes, nanosized endogenous vesicles, can induce adaptive immune responses and have been tested in cancer treatment. Similarly, the iNKT cell ligand alpha-galactosylceramide (aGC) presented on the MHC class I-like molecule CD1d has been used for therapeutic purposes in cancer trials. Here, we investigated if exosomes can be loaded with aGC to activate iNKT cells and potentiate a cancer-specific adaptive immune response. We showed that aGC loaded exosomes readily activate iNKT cells both in vitro and in vivo. In addition, exosomes loaded with aGC and the model antigen ovalbumin (OVA) induced a potent NK and gammadelta T cell innate immune response, followed by synergistically amplified T- and B-cell responses. Interestingly, aGC/OVA-loaded exosomes did not induce iNKT cell anergy, in contrast to soluble aGC, and were more potent than soluble aGC and OVA in inducing adaptive immunity. Finally, tumour-bearing mice treated with aGC/OVA-loaded exosomes displayed decreased tumour growth and increased T cell tumour infiltration in comparison to mice immunised with OVA-loaded exosomes in an OVA-specific mouse melanoma model. These results show that exosomes loaded with protein antigen and the glycolipid aGC effectively activate both innate and adaptive immunity, important for future cancer immunotherapy design.

**Neutrophil-derived ectosomes down-regulate inflammasome-driven inflammation in vivo**

A. Cumpelik ^1^, D. Zecher^2^ and J.A. Schifferli^3^

^1^Department of Biomedicine, University Hospital Basel, Basel, Switzerland; ^2^Department of Transplantation Immunology and Nephrology, University Hospital Basel, Basel, Switzerland; ^3^Department of Internal Medicine, University Hospital Basel, Basel, Switzerland

*Introduction*: Inflammasomes are molecular platforms for IL-1β maturation with IL-1β being one of the dominant cytokines driving systemic inflammatory responses. Upon activation, neutrophils (PMN) rapidly release large numbers of ectosomes (PMN-Ecto) from their surface. Here, we used a model of sterile peritoneal inflammation induced by the prototypical inflammasome activators mono-sodium urate crystals (MSU) and alum (AL) to analyse the in vivo effects of PMN-Ecto. *Materials and methods*: The peritoneum of C57BL/6 mice was preconditioned with PMN-Ecto and subsequently stimulated with either MSU or AL. Four and 14 hours after stimulation, we assesed the cytokine profile (IL-1β, IL-6, MCP-1 and KC) and phenotyped leukocyte subsets in both the peripheral blood and peritoneal compartment. Liposomes containing either phosphatidylserine (PS) or control phosphatidylcholine (PC) were used to implicate PS expression in the regulatory mechanism of PMN-Ecto. *Results*: Mice preconditioned with PMN-Ecto showed a two-fold suppression of early inflammatory cytokines in both compartments and failed to raise blood PMN counts in comparison to mice treated with MSU or AL only. This initial unresponsiveness translated into a three-fold decrease in peritoneal PMN counts at a later point of time. While these immunosuppressive effects were reproduced by PS liposomes, control liposomes had no effect. Notably, when peritoneal influx of PMN was the highest, following challenge with MSU, we recovered substantial numbers of endogenous PMN-Ecto from the peritoneal cavity. *Conclusion*: These results show that PMN-Ecto are immunosuppressive in vivo containing inflammasome-driven inflammation. PMN-Ecto-mediated control of inflammation is a compelling concept of an auto-regulatory mechanism initiated by the otherwise prototypically proinflammatory PMN. Recovery of endogenous PMN-Ecto at the time of the highest PMN influx suggests that PMN-Ecto release occurs and is relevant in vivo.

**Red blood cell-derived microvesicles amplify systemic inflammation in endotoxemic mice by thrombin-dependent activation of complement**

D. Zecher ^1^, A. Cumpelik^2^ and J.A. Schifferli^3^

^1^Department of Research and Department of Transplantation Immunology and Nephrology, University Hospital Basel, Basel, Switzerland; ^2^Department of Research, University Hospital Basel, Basel, Switzerland; ^3^Department of Research and Department of Medicine, University Hospital Basel, Basel, Switzerland

*Introduction*: Transfusion of blood after extended storage has been associated with increased morbidity and mortality and the development of acute lung injury in the critically ill. During storage, red blood cells release large numbers of microvesicles (ectosomes). These ectosomes (E-Ecto) accumulate over time in the storage bags and are given to patients at the time of transfusion. We hypothesised that E-Ecto might be responsible for some of the deleterious effects of aged blood transfusions. *Methods*: We established a mouse transfusion model using E-Ecto purified from aged mouse RBC. Mice received E-Ecto intravenously with or without prior priming with LPS. Four hours later, lung histology, pulmonary as well as peripheral blood leukocyte counts and serum cytokines were analysed. *Results*: Injection of E-Ecto into healthy mice had no effect. However, they aggravated pulmonary leukocyte sequestration and peripheral blood leukopenia induced by LPS without causing overt lung injury. LPS-induced pro-inflammatory cytokines were significantly increased following injection of E-Ecto. These effects were not seen in C5aR-deficient mice. In vitro, E-Ecto bound C3 fragments after incubation with serum but failed to bind IgM, IgG or C1q. C3 fragment binding in vitro as well as the in vivo effects of E-Ecto could be prevented by inhibiting thrombin generation. The E-Ecto-induced phenotype in vivo could be recapitulated using phosphatidylserine (PS)-expressing liposomes, suggesting that surface expression of PS by E-Ecto was involved in the observations made. *Conclusions*: The interplay between the coagulation and complement systems appears to be necessary for E-Ecto to enhance LPS-induced inflammation. These findings offer a possible mechanism for some of the deleterious effects of aged blood transfusions in the critically ill. Recognition of E-Ecto as potential mediators of transfusion-related morbidity has implications for blood storage, preservation and allocation strategies.

**Immuno suppressive miRNA that is free of exosomes targets effector T cells**

Philip Askenase

Yale University, New Haven, CT, USA

*Introduction*: We studied circulating immunosuppressive extracellular miRNA free of exosomes in murine contact sensitivity (CS). *Methods*: The exRNA was induced by high antigen dose tolerisation of CD8+ suppressor T cells in the spleen and lymph nodes. *Results*: The suppressive miRNA was identified by differential enzymatic susceptibility and progressive molecular chemical enrichment. The miRNA free of exosomes had a protective hydrophobic chaperone protein, circulated in the blood plasma, and targeted and suppressed distant effector T cells antigen-specifically even in the absence of exosomes or immunoglobulins. This was due to specific antibody-coated exosomes released by B cells among the targeted effector T-cell population, transfected prior to their adoptive transfer of CS. miR-150 was responsible since its transfection alone sufficed to mediate suppression down to a dose of 750 pg (femtomolar, 10-15). The miRNA free of exosomes also suppressed responses in actively sensitised mice, suggesting transfection of relevant exosomes in vivo. *Conclusions*: This is the first demonstration that freely circulating ex-miRNA can inhibit targeted effector T cells by entering antigen-specific exosomes produced by B cells in the targeted cell population that then alter targeted cells. This is the only mechanism yet described for passing functional isolated free exRNA between cells and contrasts release of exosomes containing miRNA from donor cells. This represents an alternate pathway for the transfer of miRNA between cells systemically in an endocrine manner.

**Oral Session 5 (Plaza Ballroom): Detection technology April 17**

**Chair: *R. Nieuwland and R. Weissleder* 13:30-15:00**

**Progress on standardisation of the pre-analytical and analytical steps of plasma microvesicles determination**

R. Lacroix ^1^, M.J. Mooberry^2^, N.S. Key^2^ and F. Dignat-George^1^

^1^VRCM, UMR-1076, INSERM, Aix Marseille Université, UFR de Pharmacie, Marseille, France; ^2^Department of Medicine, University of North Carolina, Chapel Hill, NC, USA

Cell-derived microvesicles (MV) are sub-micron-sized vesicles released from cell membranes in response to activation or apoptosis. MV originating from blood and vascular cells are elevated in a variety of disorders. Although MV counts may provide useful diagnostic/prognostic information, assessment of their pathophysiological relevance in multicentre studies is hampered by methodological concerns and a lack of standardisation. The Scientific Subcommittee on Vascular Biology of the International Society in Thrombosis and Haemostasis (ISTH) has set up a network aimed at standardising pre-analytic and analytical variables for MV analysis. This presentation will focus on the key steps achieved by this group towards this goal. A pre-analytical protocol has been recently proposed and validated in a multicentre study. It proved to significantly reduce the variability of MV measurement. A collaborative workshop has also been organised to define the inter-laboratory reproducibility of MV counts using standard flow cytometry (FCM). As a result, a specific beads-based calibration system proved to be useful to allow instrument qualification and monitoring; however, differential behaviour among flow cytometers (FCMr) sub-types impeded a universal standardisation for MV enumeration. A modified strategy has been recently proposed in order to provide optimised scatter-based reference levels. In preliminary studies, similar counts were obtained using different platforms, indicating the ability of this optimised standardisation strategy to achieve inter-instrument reproducibility even with various types of instruments. Moreover, this strategy was adapted to the new generation of high-sensitivity FCMr. Based on this new approach, a further workshop has been initiated to evaluate the inter-instrument reproducibility of MV counts among different platforms. Progress in standardisation is critical to define the usefulness of MV as true biomarkers in clinical practice.

**High sensitivity analysis of individual membrane vesicles**

John Nolan

La Jolla Bioengineering Institute, San Diego, CA, USA

*Background*: Cell-derived lipid membrane vesicles have been implicated in a wide variety of important biological roles. Microvesicles may serve as useful biomarkers for a number of conditions, including cardiovascular disease, autoimmune disease and cancer. However, their small size and low refractive index make the analysis of single vesicles very difficult. The lack of appropriate standards adds to this difficulty. *Methods*: We have developed and characterised a nanoparticle flow cytometer (NPFC) optimised for analysis of individual membrane vesicles. Liposomes were prepared by extrusion through membrane filters for use as standards for flow cytometry measurements. Lipophilic dyes were characterised for their ability to stain these liposomes. Cell-derived microvesicles were generated from THP-1 cells via stimulation with ionophore A23187 and stained for a variety of surface markers, including annexin V, CD14 and tissue factor (TF). *Results*: The NPFC showed a 5- to 10-fold improvement in fluorescence detection efficiency (Q) compared to a commercial instrument and resolution limits of 230 MESF FITC and 71 MESF PE. Liposomes were detected based on signal from lipophilic dyes and different liposome size populations showed fluorescence intensity to be proportional to vesicle surface area. Annexin V staining of THP-1-derived vesicles revealed distinct populations of PS+ and PS- particles. Interestingly, both TF and CD14 were below the detection limit of our high-sensitivity instrument. *Conclusion*: Microvesicles present in biological fluids are likely to be heterogeneous with respect to cell of origin and composition, making the analysis of single microvesicles essential. Here we demonstrate optimised instrumentation and fluorescence-based triggering for detection of individual small lipid vesicles. We believe this approach is a first step towards both more rigorous characterisation of vesicle populations and standardisation of vesicle measurements via flow cytometry.

**High-resolution flow cytometric analysis of nano-sized vesicles: how ‘swarm detection’ influences quantitative and qualitative analysis of nano-sized vesicles**

G.J.A. Arkesteijn^1^, E.N.M. Nolte-‘t Hoen^2^ and M.H.M. Wauben ^2^

^1^Department of Immunology, Faculty of Veterinary Medicine, Utrecht University, Utrecht, The Netherlands; ^2^Department of Biochemistry and Cell Biology, Faculty of Veterinary Medicine, Utrecht University, Utrecht, The Netherlands

*Introduction*: Cell-derived vesicles are heterogeneous with the majority of vesicles in the nano-sized range (<300 nm). To unravel the molecular composition of individual vesicles, single particle-based high-throughput multi-parameter techniques are needed. We developed a high-resolution fluorescence-based flow cytometric method which enables integrated analysis of multiple parameters (quantity, light scattering, buoyant density and surface markers) of individual nano-sized vesicles. A problem related to flow cytometric analysis of individual nano-sized vesicles is ‘swarm detection’, that is, the simultaneous illumination and detection of multiple vesicles measured as a single event. We investigated the contribution and influence of ‘swarm detection’ in our high-resolution flow cytometric method. *Methods*: Serial dilutions of fluorescently labelled nano-sized beads, liposomes and cell-derived vesicles were analysed by (high-resolution) flow cytometry. Influences of particle concentration, pressure and core stream diameter on quantitative and qualitative particle analysis were investigated. *Results*: ‘Swarm detection’ obscures the analysis of nano-sized particles in highly concentrated samples. Besides an underestimation of the vesicle quantity, ‘swarm detection’ resulted in increased wide-angle forward light scattering and fluorescence signals. Accurate titration of samples resolved these problems and resulted in reliable quantitative and qualitative high-resolution flow cytometric analysis of individual nano-sized vesicles. *Conclusion*: For quantitative and qualitative high-resolution flow cytometric analysis of individual nano-sized vesicles appropriate sample titration is of utmost importance to avoid misinterpretations due to ‘swarm detection’. Increased signals caused by ‘swarm detection’ in highly concentrated nano-sized vesicle preparations explain false detection above the resolution limit and quantitative underestimation of nano-sized vesicles on mainstream flow cytometry.

**Plasmonic nanohole arrays for detection and molecular profiling of cancer exosomes**

Hyungsoon Im, H. Shao, M. Liong, C. Castro, R. Weissleder and H. Lee

Massachusetts General Hospital, Boston, MA, USA

*Introduction*: Surface plasmon resonance (SPR) biosensors have been widely used to study the interactions of proteins, small molecules and nucleotides. Their sensing range (50–200 nm) parallels the size range of exosomes. Here, we demonstrate the detection and profiling of exosomes obtained from ovarian cancer cells using novel plasmonic nanohole arrays. Material and *Methods*: Plasmonic nanohole arrays were patterned into a 200-nm-thick gold film on a glass slide and integrated with a multi-channel flow cell. Twelve different antibodies with duplicates were captured via a PEG layer on the surface of 24 nanohole arrays through the multi-channel flow cell, allowing exosomes to be screened for molecular markers, simultaneously. Exosomes obtained from ovarian cancer cells were introduced and their binding to antibodies was measured from a spectral shift in the resonance wavelength of nanohole array. *Results*: *T*he nanoholes displayed high sensitivity for exosome detection. Using CD63 as a pan-exosomal target marker, we established the detection threshold of ~2,000 exosomes. The level of CD63 also correlated well with total exosome counts, and thereby was used to estimate exosome concentration in a given sample. We next profiled exosomes for a panel of cancer biomarkers. The profiling results showed an excellent match with parental cells, indicating that cancer-derived exosomes reflect the molecular signature of their primary tumour. Importantly, we identified a unique set of markers for ovarian cancer detection: the combined expression of EpCAM, MUC1 and CD44 was highly elevated. *Conclusions*: The plasmonic nanohole is a promising platform for exosomal analyses. The system can accurately quantify and profile exosomes in multiplexed, parallel fashion. Its simple optical setup for operation could be readily adapted into clinical applications.

**Evaluating novel methods for characterisation of circulating microparticles: nanoparticle tracking analysis and scanning ion occlusion sensing**

Shona Pedersen ^1^, M. Mørk^2^, S. Nejlund^2^ and S.R. Kristensen^2^

^1^Aalborg University Hospital, Aalborg, Denmark; ^2^Department of Clinical Biochemistry, Aalborg University Hospital, Aalborg, Denmark

*Introduction*: Microparticles (MP) appear to play a significant role in many biological processes both physiological and pathological. Thus, methods for their detection, analysis and characterisation are in great demand. Recently, novel detection platforms have emerged but a clinical evaluation of these new technologies is lacking. Thus, the aim of this study was to assess and compare two novel devices for the characterisation of MPs, namely nanoparticle tracking analysis (NTA) (NanoSight^®^) and scanning ion occlusion sensing (SIOS; qNano^®^). In addition, we examined the feasibility for establishing a normal reference range for each method. *Material and methods*: Polystyrene microbeads were used to determine the accuracy of particle size and distribution. The impact of various pre-analytical variables, including sample dilution, freezing, day-to-day variation and fasting versus non-fasting on plasma samples from healthy persons, was evaluated. To determine normal reference ranges for these methods, platelet-free plasma from 40 healthy subjects will be prepared according to international guidelines. *Results*: The methods display reproducibility for microparticle measurements performed on plasma samples, suggesting a foundation for their use in patient studies. We have observed proportionality between the degree of plasma dilution and measured microparticle concentration. Freezing of the plasma samples at −80°C results in a rise of varying magnitude of microparticles concentration in plasma, when compared to fresh samples. Preliminary data suggest that fasting blood samples display significantly lower microparticle concentrations than postprandial samples. *Conclusion*: NTA and qNano display reproducible and rather precise measurements and can be used as complementary methods for microparticle detection in human plasma. We will propose preliminary normal reference values based on particle size and concentration for fasting versus non-fasting plasma samples.

**From exosomes to cells: tuneable resistive pulse sensors (TRPS) for high-resolution characterisation of nano- to micro-scale vesicles**

Darby Kozak ^1^, W. Anderson^2^, R. Vogel^2^ and M.F. Broom^3^

^1^Izon Science, USA; ^2^The University of Queensland, Australia; ^3^Izon Science, New Zealand

*Introduction*: Accurate high-resolution characterisation of complex systems such as microvesicles is critical to understanding their function and potential utilisation as predictive markers. Tuneable resistive pulse sensors (TRPS), such as the Izon qNano, have been used to accurately characterise the size, charge and concentration of nano- to micro-scale particulate suspensions. Measuring the properties of each particle (e.g. exosome, microvesicle or cell) provides resolution analysis better than optical techniques. Furthermore, the capacity to simultaneously measure the size and charge on a particle-by-particle basis provides a unique insight in the role that these properties play. Herein, we describe the principles and demonstrate how TRPS has been used to improve the characterisation of complex vesicle suspensions. *Materials and methods*: Measurements were made using an Izon qNano, New Zealand, unless otherwise stated. Polystyrene particles were purchased from Bangs Laboratories, USA, unmodified and modified vesicles from FormuMax, USA. Particle concentration, size and charge (zeta-potential) were calculated using Izon Control Suite Software V2.4. *Results*: The properties of synthetic and biological particles were measured using an Izon qNano. Fundamental modelling of the TRPS signal enables simultaneous measurement of individual particle size and charge (zeta-potential). This particle-by-particle analysis is a new and unique tool that was used to characterise differences between modified and unmodified vesicles. In addition to obtaining the properties of each vesicle, collating the data gives a true depiction of the overall sample distribution. This holds significant promise for studying extracellular vesicles where differences in the surface properties may dictate their function. *Conclusions*: The high-resolution size, charge and concentration analysis of TRPS make it a unique tool for better understanding cellular and extracellular vesicles.

**Oral Session 6 (Georgian): Glioma-derived EVs April 17**

**Chair: *E. Krichevzki and A. Charest* 13:30-15:00**

**A collaborative study to determine the sensitivity and specificity of plasma and cerebrospinal fluid exosome EGFRVIII detection compared to tissue EGFRVIII detection by QPCR assay of exosomal RNA**

Bob Carter ^1^, F. Hochberg^2^, R. Kim^1^, J. Skog^3^, S. Sivaraman^2^, M. Wallace^4^ and The Exosome Consortium*^5^

^1^University of California, San Diego, CA, USA; ^2^The Massachusetts General Hospital, Boston, MA, USA; ^3^Exosome Diagnostics, New York, NY, USA; ^4^ABC2 Foundation, San Francisco, CA, USA; ^5^The Exosome Consortium, USA

*UCSD (Bob Carter), Miami (Ricardo Komotar), Neurochirurgische Klinik und Poliklinik, Germany (Florian Ringel), Utah (Randy Jensen), Henry Ford (Steve Kalkanis), MGH (Will Curry, Dan Cahill), Brigham (E.A. Chiocca), Hopkins (Chetan Bettegowda), Swedish (Gregory Foltz), MD Anderson (Frederick Lang), UCLA (Linda Liau), Cornell (John Boockvar), and Vanderbilt (Reid Thompson)

Exosome Consortium links 18 brain tumour centres in a collaborative effort to validate a biomarker diagnostic using exRNA studies. Funded by ABC2, the Consortium pilots exRNA analysis of plasma and cerebrospinal fluid (CSF) EGFRvIII. We have: (a) evaluated EGFRvIII status in plasma, CSF and tumour exosomes from glioma patients; (b) assessed the feasibility of collecting the 3 biofluids from patients prior to tumour excision; (c) developed protocols for specimen handling and assay performance; (d) designed a study by which to determine the sensitivity and specificity of plasma and CSF exosome EGFRvIII detection compared to tissue EGFRvIII detection by qPCR assay of exRNA; (e) designed fields of clinical, MRI and pathologic data retrieval that inform correlations with tissue, plasma and CSF Exo EGFRvIII status; and (f) assess the potential evaluation of tumour, CSF and plasma exRNA for genomic, proteomic and bioinformatics analyses. The SOPs, study kits, IRB approvals and site initiations have been conducted for the Consortium members (above) and visits planned for UCSF, Duke, City of Hope, Columbia and Florida Hospital Cancer Institute. To date, a total of 38 matched CSF, blood and tumour tissue sets have been collected since July 2012. The study hypothesises CSF-based exRNA EGFRvIII detection which demonstrates a true sensitivity of 90% with analyses using the facilities of exosome diagnostics. We will present specimen handling procedures, processing of plasma, CSF and tumour tissue as well as preliminary results of assays.

**Analysis of glioblastoma microvesicles using a µNMR chip**

Ralph Weissleder

Massachusetts General Hospital, MA, USA

Glioblastomas shed large quantities of small, membrane-bound microvesicles (MV) into the circulation. While these hold promise as potential biomarkers of therapeutic response, their identification and quantitation in the clinic remain challenging. We have recently developed a highly sensitive and rapid analytical technique for profiling circulating MV directly from blood samples of glioblastoma patients. MV, introduced onto a dedicated microfluidic chip, are labelled with target-specific magnetic nanoparticles and detected by a miniaturised nuclear magnetic resonance system (µNMR). Compared with current methods, this integrated system has a much higher detection sensitivity and can differentiate glioblastoma MV from non-tumour host cell-derived MV. We also show that circulating MVs can serve as a surrogate for primary tumour mutations and a predictive metric of treatment-induced changes. This platform could provide both an earlier indicator of drug efficacy and a potential molecular stratifier for human clinical trials.

**Heparin blocks horizontal transfer of extracellular vesicles mediated via glioma**

N.A. Atai ^1^, L. Balaj^2^, H. van Veen^3^, J. Skog^4^, P. Jarzyna^2^, C.J.F. van Noorden^3^, X.O. Breakefield^1^ and C.A. Maguire^2^

^1^Harvard University/MGH, USA; ^2^Massachusetts General Hospital, Boston, MA, USA; ^3^University of Amsterdam, Amsterdam, The Netherlands; ^4^Exosome Diagnostic Inc., USA

*Introduction*: Heparin has been used in the clinic since the late 1930's, mainly as an anticoagulant to prevent the formation of clots in blood for procedures such as organ transplants and surgeries, leading to great therapeutic benefit in humans. This glycosaminoglycan is the most highly negatively charged, natural-occurring biochemical and is conserved across multiple organisms. Heparin possesses multiple biological roles. This study focuses on the role of heparin on extracellular vesicles (EVs). *Methods*: Transwell system was used to study the transfer of tumour-derived EVs in presence of heparin. Binding assays, transmission electron microscopy and confocal microscopy were used to visualise the interaction of heparin with EVs. *Results*: We found that inclusion of heparin greatly diminished the uptake of EVs from donor to recipient cells. Incubation of EVs with heparin resulted in micron-sized mesh-like structures, leading to direct interaction between heparin and EVs. *Conclusion*: We show that heparin interacts with the uptake of EVs into recipient cells. The effect of heparin on EVs uptake may provide a unique research tool to study EV functions and opens the door for exploring the potential of heparin as a therapeutic for disease in which EVs play a role. *Future direction*: Next, we want to use qRT-PCR to study effect of heparin on transfer of EGFRvIII containing EVs. We expect to see block in the uptake of this gene into recipient cells.

**Extracellular vesicles mediate hypoxic signalling in cancer**

M. Belting ^1^, H.C. Christianson^1^, J.E. Welch^1^, K.J. Svensson^1^, E. Fredlund^2^, M. Ringnér^1^, M. Mörgelin^1^, E. Bourseau-Guilmain^1^ and P. Kucharzewska^1^

^1^Lund University, Lund, Sweden; ^2^Karolinska Institute, Solna, Sweden

*Introduction*: Malignant brain tumours, such as glioblastoma (GBM), are characterised by hypoxia, endothelial cell (EC) hyperplasia and hypercoagulation. However, how these phenomena of the tumour microenvironment may be linked at the molecular level during tumour development remains ill-defined. *Results*: We recently provided evidence that hypoxic cancer cells release substantial amounts of tissue factor (TF), that is, the major initiator of coagulation, associated with secreted EVs with exosome-like characteristics (1). Interestingly, EVs derived from GBM cells were found to trigger PAR-2-dependent activation of ECs. On-going in vitro hypoxia experiments with GBM cells and studies with patient materials (2) reveal the enrichment in EVs of hypoxia regulated mRNAs and proteins, several of which were associated with poor glioma patient prognosis. These results were associated with potent modulation of stromal cells by hypoxic EVs in vitro and in vivo. *Conclusions*: We conclude that EVs constitute a novel, potentially targetable mediator of hypoxia-driven tumour development and suggest that the hypoxic vesicle signature may serve as a non-invasive biomarker to assess the oxygenation status and aggressiveness of malignant tumours.

References

1. Svensson KJ, et al. PNAS, 2011;108:13147–52.

2. Kucharzewska P, et al. under revision. 2013.

**Circulating microparticles of glial origin and tissue factor bearing in glioblastoma multiforme**

Maria Teresa Sartori ^1^, E. Campello^1^, A. Della Puppa^2^, A. Ballin^1^, E. Be Bon^1^, D. d'Avella^2^, G. Cella^1^ and P. Simioni^1^

^1^Department of Cardiologic, Thoracic and Vascular Sciences, Univer, Italy; ^2^Department of Neurosciences, University Hospital, Padova, Italy

*Introduction*: Circulating microparticles (MPs) seem to be involved in several processes, including venous thromboembolism (VTE) development. Patients with glioblastoma multiforme (GBM, WHO grade IV glioma) are at high risk for VTE. Therefore, we prospectively investigated the presence and behaviour of MPs of different origin in a population of GBM patients before and after treatment of the neoplasm. *Materials and methods*: Sixty-one consecutive patients with GBM undergoing gross-total (41) or sub-total (20) surgical resection followed by combined radio-chemotherapy and 20 healthy controls were studied. Glial- (GFAP+) and endothelial (CD62E+)-derived MPs, annexin V-positive (AV+) and tissue-factor-bearing (TF+) MPs were assayed by flow cytometry on blood samples drawn before 1 week and 1, 4 and 7 months after surgery. *Results*: In GBM patients, baseline levels of GFAP+, TF+ and GFAP+TF+ MPs were significantly higher than in controls (p<0.001). All these MPs levels significantly increased 1 week, 1, 4 and 7 months after surgery with respect to pre-operative levels. GFAP+ and GFAP+TF+ MPs levels were significantly higher at each time control after surgery as compared to baseline in both gross-total and sub-total resected patients; at the 7th month, a further significant increase with respect to 7 days control was seen only in sub-total-resected patients. Eleven patients with VTE showed baseline TF+ MPs levels significantly higher than patients who did not present VTE (230.91±99.87 vs. 163.98±85.84 MPs/µL, p 0.04). TF+ MPs levels above the 90th percentile were seen in 5 of 11 patients with VTE and in 5 of 49 patients without VTE; the different prevalence was statistically significant (p 0.0046; RR 4.17, 95% CI 1.57–11.03). *Conclusions*: Glial-derived and/or TF-bearing MPs were increased in GBM patients, especially after tumour treatment, and correlated with disease progression. A possible role of TF+ MPs on the risk of VTE is suggested.

**Direct comparison of glioblastoma large oncosomes and exosomes/microvesicles**

L. Balaj^1^, M. Morello ^2^, H. Shao^3^, V. Minciacchi^2^, X. Zhang^1^, M.R. Freeman^4^, H. Lee^5^, X.O. Breakefield^1^ and D. Di Vizio^4^

^1^Department of Neurology and Program in Neuroscience, Massachusetts General Hospital and Harvard Medical School, Boston, MA, USA; ^2^Cancer Biology Program, Samuel Oschin Comprehensive Cancer Institute, Cedars-Sinai Medical Center, Los Angeles, CA, USA; ^3^Center for Systems Biology, Massachusetts General Hospital and Harvard Biophysics Program, Harvard, MA, USA; ^4^Cancer Biology Program, Cedars Sinai Medical Center, Los Angeles, CA, USA; Boston Children's Hospital, Boston, MA, USA; ^5^Center for Systems Biology, Massachusetts General Hospital, Boston, MA, USA

*Introduction*: Prostate cancer and other tumour cells secrete large oncosome vesicles ranging between 1 and 10 µm in diameter. Cells also secrete smaller extracellular vesicles (EVs), including exosomes and microvesicles (EMs). We sought to characterise EMs and large oncosomes released by the U87 glioblastoma cells for size, morphology and tumour biomarker load as well as some putative exosomal markers. *Materials and Methods*: The two EVs populations from glioblastoma U87 and HUVEC cells were separated by differential centrifugation, followed by sucrose gradient in selected experiments and compared by nanoparticle tracking analysis (NTA), cryoEM, immunofluorescence (IF), qRT-PCR, western blotting and µNMR (nuclear magnetic resonance). *Results*: We report that both cell lines produce more EMs than large oncosomes. IF and cryoEM data indicate that a portion of large oncosomes incorporate EMs. We also compared the content of EGFRvIII RNA in both EVs populations from U87 expressing this mutated form of the receptor, and showed that, although the total number of large oncosomes was smaller, the content of EGFRvIII was comparable between the two populations, suggesting that large oncosomes may better represent the content of tumour cells. We will present comparison by FACS as well diagnostic NMR. Furthermore, we injected a group of nude mice with the U87EGFRvIII cell line and compared the EGFRvIII load in large oncosomes versus EMs, as well as detection rate of EGFRvIII in the plasma of these mice, in large oncosomes and EMs using qRT-PCR as well as µNMR. *Conclusions*: Here, we report for the first time a direct comparison of large oncosomes and EMs released by a glioblastoma (U87) and normal endothelial cells (HUVEC). Large oncosomes contain tumour biomarkers and do not require ultracentrifugation, thus they may represent an advantage over EMs for biomarker detection. Furthermore, because of their size, they may allow single particle analysis of the content derived from a tumour.

**Coffee and Poster Viewing April 17**

**Poster Sessions I-II 15:00-15:45**

**Parallel Oral Sessions 7-9 15:45-17:45**

**Oral Session 7 (Imperial Ballroom): Immune modulation April 17**

**Chair: *M. Wauben and P. Askenase* 15:45-17:45**

**Structure and function of MHC class I molecules on exosomes**

Simon Powis ^1^, S. Lynch^1^, S. Santos^1^, A. Antoniou^2^ and E. Campbell^1^

^1^University of St Andrews, UK; ^2^University College London, UK

*Introduction*: MHC class I molecules function by binding and presenting short antigenic peptides to T lymphocytes of the immune system, allowing the detection and elimination of virally infected cells. MHC class I molecules are normally detected as monomeric structures on the surface of cells. We have previously reported novel dimeric MHC class I structures on exosomes. Here we have studied the ability of the different loci of human HLA MHC class I molecules (HLA-A, -B and -C) to form dimeric structures. *Materials and methods*: MHC class I deficient cells lines, CEM and .221, were transfected with a range of HLA-A, -B and -C molecules. Exosomes were isolated from culture supernatants by ultracentrifugation and analysed by immunoblotting under non-reducing conditions. Antigen presentation was measured by IFNγ ELISPOT. *Results*: HLA-A and most HLA-B alleles formed dimeric structures on exosomes, controlled by a disulphide link between unpaired cysteine residues in the cytoplasmic tail domain. Exosome-expressed MHC class I molecules were also able to present viral peptides to T cells. In marked contrast, HLA-C molecules appeared not to form similar dimeric structures on exosomes. *Conclusions*: Many MHC class I molecules have the ability to form novel dimeric disulphide-linked structures on exosomes. However, HLA-C molecules appear to behave differently. The mechanisms controlling dimer formation and their influence on antigen presentation are currently under investigation.

**Endothelial cell microparticles as modulators of immune responses**

J. Wheway, S. Latham, V. Combes and G.E.R. Grau

The University of Sydney, Australia

Endothelial cells (ECs) closely interact with circulating lymphocytes. Aggression or activation of the endothelium leads to the shedding of EC microparticles (MP), found in high plasma levels in numerous immuno-inflammatory diseases, for example, atherosclerosis, sepsis, multiple sclerosis and cerebral malaria, supporting a role for endothelial MP as effectors and markers of vascular dysfunction. Given the recently described role for human brain microvascular endothelial cells (HBEC) in modulating immune responses (Wheway, PLoS ONE 2013), we investigated how HBEC-derived microparticles (EC-MP) could interact with and support the proliferation of T cells. EC-MP, like their mother cells, expressed molecules important for antigen presentation and T-cell co-stimulation, that is, MHC I, MHC II, CD40 and ICOSL. Compared to resting HBEC, cytokine-stimulated HBEC released higher numbers of MHC I- and ICOSL-positive MP with a higher surface antigen expression. Interestingly, HBEC were able to take up fluorescently labeled antigens via macropinocytosis and released EC-MP also containing fluorescent antigens, suggestive of antigen carryover from EC to EC-MP. Following co-culture with T cells, fluorescently labeled MP from resting or cytokine-stimulated HBEC readily bound to both CD4+ and CD8+ T cells as assessed by confocal microscopy. Flow cytometry showed that EC-MP formed long-lasting conjugates with both CD4+ and CD8+ subsets. Moreover, in CFSE, T-cell proliferation assays using anti-CD3/CD28 mAbs or other T-cell mitogens, EC-MP promoted the proliferation of both CD4+ and CD8+ subsets. Importantly, EC-MP alone was not enough to trigger T-cell proliferation as T cells required exogenous stimuli for EC-MP to promote their proliferation. In conclusion, this study provides evidence that EC-MP can enhance T-cell activation and potentially ensuing antigen presentation, thereby pointing towards a novel role for MP in neuro-immunological complications of infectious diseases.

**Human macrophage pro-resolving lipid mediators are regulated by neutrophil microparticles in resolution of inflammation via novel transmicroparticle-cellular route**

J. Dalli and C.N. Serhan

Brigham and Women's Hospital and Harvard Medical School, USA

Leukocytes release microparticles both under homeostatic conditions and during inflammatory response. We have recently found that a subset of neutrophil microparticles is produced during resolution of inflammation and carry precursors for the biosynthesis of pro-resolving lipid mediators. Here we carried out lipid mediator metabololipidomics to assess the actions of these microparticles on human primary leukocytes during the resolution of inflammation. Incubation of human neutrophil microparticles with human monocyte-derived macrophages stimulated macrophage efferocytosis of apoptotic neutrophils and up-regulated the biosynthesis of pro-resolving mediators, including Resolvin (Rv) D1, RvD2 and RvE2. We found that these actions were mediated via direct interactions of microparticle with G-protein-coupled receptors expressed on macrophages, since incubation of macrophages with cholera toxin or pertussis toxin inhibited the up-regulation of pro-resolving lipid mediators in macrophages. Using deuterium-labelled lipid mediator precursors (d8-arachidonic acid, d5-eicosapentaenoic acid and d5-docosahexaenoic acid) in microparticles demonstrated their contributions to the biosynthesis of pro-resolving mediators, including RvD2, Maresin 1 and Protectin D1, during efferocytosis via a novel transmicroparticle-cellular biosynthetic route. Together, these results establish that human neutrophil microparticles stimulate macrophage efferocytosis and contribute to macrophage biosynthesis of pro-resolving mediators during resolution of inflammation.

**Platelet ectosomes suppress NK cell function**

S. Sadallah ^*^, L. Schmied^*^, H. Nozad, C. Eken, M. Stern and J.A. Schifferli

Department of Biomedicine, University Hospital Basel, Basel, Switzerland

*equal contribution

When stored for transfusion, platelets (PLT) release microvesicles, mainly ectosomes (Ecto). We showed previously that these Ecto have suppressive activities on macrophages, DC and CD4 T cells. Here we studied their effects on NK cells. Total PBMC or enriched NK cells obtained by CD56-positive selection were exposed to different concentrations of PLT-Ecto. After 24 h, NK cells were analysed by FACS for (a) their cytotoxic activity by incubating them with target cells at a ratio of 5:1 followed by CD107a and IFNγ determinations; (b) their surface expression of NKG2D, NKp30 and NKp46. Exposure of PBMC to PLT-Ecto (n = 4) induced a dose-dependent inhibition of cytotoxic activity of NK cells with a decreased expression of CD107a (19–9%) and IFNγ (24–15%). This suppressive activity was confirmed using enriched NK cells: PLT-Ecto (n = 5) decreased CD107a (from 24 to 17%) and ?F?γ (from 17 to 11%). The analysis of NKG2D, NKp30 and NKp46 expression showed that PLT-Ecto decreased significantly NKG2D (n = 5; p < 0.036) and NKp30 (n = 6; p ≤ 0.001) with no effect on NKp46. TGFβ1 is expressed by PLT-Ecto. It impairs NK cell cytotoxic activity and reduces NKG2D expression, which is in line with our observation. TGFβ1 present on PLT-Ecto can only explain some of the inhibitory activities, since Ecto released by PMN and erythrocytes (ERY) had similar effects: PMN-Ecto inhibited NK cells of PBMC as efficiently as PLT-Ecto and slightly less purified NK cells. By contrast, ERY-Ecto did not modify NK cells of PBMC but inhibited purified NK cells. In addition, neither modified surface marker expression. These apparent discrepancies are best explained by direct inhibitory effects of Ecto on NK cells as well as indirect effects through cells other than NK cells in PBMC and by specific effects of each Ecto type in relation to phosphatidylserine, TGFβ1 or the expression of other molecules. In sum, the inhibition of NK cells may well play an immunomodulatory role upon PLT transfusion.

**TLR4 senses oxidative stress mediated by oxidative stress-derived microvesicles**

Mateja M. Keber ^1^, M. Frank^2^, S. Hayer^3^, V. Kralj-Igliè^4^, B. Rozman^2^, S. Horvat^5^ and R. Jerala^5^

^1^National Institute of Chemistry, Slovenia; ^2^Department of Rheumatology, University Medical Centre Ljubljana, Slovenia; ^3^Division of Rheumatology, Department of Internal Medicine III, Medical University of Vienna, Austria; ^4^Laboratory of Clinical Biophysics, Faculty of Medicine, University of Ljubljana, Slovenia; ^5^Department of Biotechnology, National Institute of Chemistry, Slovenia

*Introduction*: Oxidative stress is a hallmark of inflammation caused by infection or sterile tissue injury. ROS that are released cause damage to cell structures, including proteins, nucleic acids and lipids, particularly unsaturated fatty acid chains. Apart from Gram-negative infections, the role of TLR4 has been established also in a number of diseases with sterile inflammation (e.g. atherosclerosis, rheumatoid arthritis (RA)). There were reports on the role of oxidised phospholipids in TLR4 activation. *Methods*: MVs were isolated from patients with RA, healthy donors or HEK293 cell line stimulated with A23187. MVs were oxidised using Fenton reaction (30 min or 24 h). In vivo MVs activity was measured by i.v. injection to C57BL/6J mice; in vitro on HEK293 cells expressing TLR4/MD-2, BMDMs from C3H/HeN (TLR4wt) and C3H/HeJ (TLR4mut) mice. Extracted RNA from stimulated BMDMs from C57BL/6J mice was hybridised to mouse affymetrix genechip 2.0 arrays and microarray analysis was performed. *Results*: Here we show that partially oxidised phospholipids in microvesicles from plasma of patients with RA or cells submitted to oxidative stress induce activation of TLR4 in cultured cells and in vivo. MVs from sera of healthy donors were converted to TLR4 agonists by limited oxidation (30 min), while prolonged oxidation (24 h) abrogated the activity. Hydro(pero)xyl PLs were identified as agonistic molecules. Activation of TLR4 by phospholipids/MVs mimics the mechanism of TLR4 activation by LPS, demonstrated by the effects of MD-2, mutations and inhibitors. However, signals from pathogens (LPS) and endogenous danger signals (MVs) induced different transcriptional response profile in mice BMDMs with a strong inflammation-resolving component induced by the endogenous signal. *Conclusions*: Hydro(pero)xyl PLs in MVs represent a ubiquitous endogenous danger signal released under the oxidative stress, which underlies the pervasive role of TLR4 signalling in inflammation.

**Exosomes trigger NFκB and STAT3 activation via TLR signalling in monocytic THP-1 cells**

Niko P. Bretz ^1^, J. Ridinger^1^, A.K. Rupp^1^, K. Rimbach^2^, S. Keller^1^, T. Eigenbrod^2^, A. Dalpke^2^, M. Sammar^3^ and P. Altevogt^1^

^1^German Cancer Research Center DKFZ, Germany; ^2^University of Heidelberg, Germany; ^3^ORT Braude College Kamiel, Israel

*Introduction*: Recent data have suggested that tumour-derived exosomes can impair immune cell functions and induces myeloid-derived suppressor cells (MDSCs). Exosomes cargo functional proteins, mRNAs and miRNAs and can be taken up by other cells, including immune cells. We analysed the effects of exosomes on monocytic differentiation/activation using THP-1 cells as a model. *Materials/methods*: Exosomes were purified from body fluids such as amniotic fluid, urine, liver cirrhosis ascites and malignant ascites from ovarian carcinoma patients by ultracentrifugation. They were characterised and used to stimulate the monocytic cell-line THP-1. Uptake of exosomes was studied by FACS and confocal fluorescence microscopy. Signal transduction was analysed via immunoblot and qRT-PCR. Cytokines were measured by ELISA. *Results*: Exosomes were efficiently taken up by THP-1 cells and induced the production of IL-1β, TNFα, IL-6 and IL-23 that are known NFκB/STAT3 target genes. Analysis of signalling pathways revealed a fast activation of NFκB and a delayed activation of STAT3. Kinetic experiments confirmed the biphasic activation, and blocking experiments showed that the initial production of IL-6 was instrumental for subsequent STAT3 activation. Importantly, triggering of cell signalling was not a unique property of tumour exosomes but was also observed by other exosomes. Pre-treatment of exosomes with proteinase K but not DNase or RNase abolished the signalling capacity. Signalling was TLR dependent, and human exosomes induced the release of cytokines from mouse bone-marrow-derived dendritic cells and macrophages in dependence of MyD88. *Conclusions*: Our results suggest that cancer-derived exosomes and exosomes from other sources trigger TLR-dependent signalling pathways in monocytic precursor cells and other immune cells. The cytokines released by STAT3-activated cells might be of importance for further micro-environmental immune modulations.

**Gene expression data suggests a crosstalk between extracellular vesicle and TNF signalling in U937 monocytes**

T.G.S. Szabo, K.S.T. Szabo, B.G.N. Gyorgy, B.T.N. Tarr, K.P.N. Paloczy, K.E. Eder, S.T. Toth, X.O. Osteikoetxea, A.N. Németh, A.K. Kittel and E.I.B. Buzás

Semmelweis University DGCI, Hungary

*Introduction*: Several lines of evidence support an immunomodulatory role of extracellular vesicles (EV). These data come from experiments with isolated EVs. Given that in body fluids cytokines and EVs may be present simultaneously, we hypothesised that a combined treatment of cells in vitro with both EVs and cytokines might reflect more precisely the biological role of EVs. *Materials and methods*: CCRF human T-lymphoma cell-derived extracellular vesicles were added to U937 cells with or without TNF. Particle concentrations and EVs’ size distributions were assessed by resistive pulse sensing (IZON qNano) and transmission electron microscopy. Gene expression levels following the treatments with EVs, TNF or a combination of EVs and TNF were analysed by a gene-expression microarray and Taqman real-time PCR. *Results*: Cluster analysis of the gene expression pattern after the “Combined” (EVs + cytokine), “EVs only” or “Cytokine only” treatments resulted in subgroups of genes, where the effects of EVs and TNF showed to be additive, antagonistic or independent. Differential expression of representative genes (such as IL-8, CD36, CCL-2 and cannabinoid receptor 2) of the major subgroups was validated by real-time PCR. Following treatment with EVs, gene set enrichment analysis showed that most differentially expressed genes were involved in cell surface G-protein-coupled receptor and NF-κB signalling, and a great portion of genes was associated with the cell membrane. *Conclusions*: Gene expression changes reported in this work suggest that the effect of EVs might be modulated by cytokines, which are present in the same extracellular compartment. The interplay between these 2 forms of intercellular communication calls for a more sophisticated model of the inflammatory environment, including both EVs and cytokines.

**Mesenchymal stem cells use extracellular vesicles to transfer mitochondria and microRNAs to modulate macrophages in lung fibrosis**

M. Di Giuseppe^1^, J. Njha^2^, D. Phinney^3^, N. Kaminski^4^, L.A. Ortiz ^5^ and E. Sala^6^

^1^EOH, USA; ^2^EOH University Pittsburgh, USA; ^3^Scripps Institute Forida, USA; ^4^University of Pittsburgh, USA; ^5^EOH University of Pittsburgh, USA; ^6^University of Mallorca, Spain

*Rationale*: Mesenchymal stem cells (MSCs) transfer mitochondria as a mechanism for macrophage regulation. Mitochondrial DNA is inflammatory and to facilitate the mitochondrial transfer MSCs silence innate immune responses. *Methods*: We labelled the mitochondria and phagosomes of human MSCs with baculovirus and co-cultured these MSCs in the presence of macrophages to follow the transfer of the GFP-labelled mitochondria. MSCs secrete different microvesicles (MV) that can be isolated from culture media by centrifugation. Larger MVs are isolated from MSC cultured media by low speed (10,000*g*) centrifugation, while typical exosomes require differential centrifugation (100,000*g*/18 h) followed by sucrose gradient separation. Total RNA extracted from exosomes isolated from MSCs was hybridised on Agilent Human microRNA microarrays. Results were analysed using Agilent GeneSpring GX 11 and validated using qRT-PCR. To determine the effect of MV and exosomes on macrophages we followed their uptake and assessed activation. To determine in vivo effects we administered silica-exposed mice with MSC-derived exosomes. *Results*: Larger MVs contain mitochondria that are loaded into phagosomes and are extruded to blebs in the cell surface of MSC where they are taken by nibbling macrophages. Following incubation with MSCs, macrophages stained positively against GFP from the MSC mitochondria and hybridised against hCox IV 2 weeks after their initial incubation, indicating that the organelle transfer is stable. Full genome miRNA microarray showed differential expression of 156 miRs with 45 significantly enriched in exosomes and 111 significantly decreased. The miR 451 and mir-630 are dramatically enriched in exosomess. Macrophage uptake of exosomes reduced TLR expression and induced M1 polarisation. MSC-derived exosomes reduce monocyte recruitment and silica-induced fibrosis in mice. *Conclusions*: MSCs utilise extracellular vesicles to transfer mitochondria and shuttle microRNAs to re-program the innate immunity.

**Oral Session 8 (Plaza Ballroom): Capture Technology April 17**

**Chair: *S. Sharma and K. Witwer* 15:45-17:45**

**Isolation and molecular characterization of circulating tumour cells using a microfluidic CTC-chip**

Shannon Stott

Massachusetts General Hospital, BioMEMS Center, Boston, MA 02114, USA

Tumour-derived epithelial cells, referred to as circulating tumour cells (CTCs), have been identified in blood from patients with cancer, including lung, prostate, colon, breast, liver and ovarian cancers. While extremely rare, CTCs provide a potentially accessible source for detection, characterization and monitoring of non-haematological cancers that would otherwise require invasive serial biopsies. We have developed a high throughput microfluidic mixing device, or the ‘Herringbone CTC-Chip’, that allows for the isolation and characterization of CTCs from the blood of cancer patients. The chip design was centred on the concept of passive mixing of blood through the generation of microvortices, ultimately increasing capture of these rare cells by dramatically increasing the number of interactions between the target cells (CTCs) and the antibody-coated substrate. Data will be presented from studies involving CTCs isolated from prostate, pancreatic, lung and breast cancer patients, demonstrating the rich content that can be derived from blood-based assays and potential application with exosomes.

**Nanosensor platform for protein typing of circulating microvesicles**

H. Shao ^1^, J. Chung^1^, L. Balaj^1^, A. Charest^2^, D.D. Bigner^3^, B.S. Carter^4^, F.H. Hochberg^1^, X.O. Breakefield^1^, R. Weissleder^1^ and H. Lee^1^

^1^Massachusetts General Hospital, USA; ^2^Tufts University, USA; ^3^Duke University, USA; ^4^UCSD, USA

*Introduction*: Glioblastomas shed large quantities of small, membrane-bound microvesicles (MVs) into the circulation. While these hold promise as potential biomarkers of therapeutic response, there remain hurdles to their identification and quantitation. Here, we describe a highly sensitive and rapid analytical technique for profiling circulating MVs directly from blood samples of glioblastoma patients. *Methods*: A microfluidic system was developed for clinical, point-of-care MV detection. The device is comprised of three components: (a) a chaotic mixer for reacting MVs with antibodies and magnetic nanoparticles, (b) a membrane filter for washing and concentrating targeted MVs and (c) a microcoil for NMR detection. The system provides multiplexed, on-chip profiling using small sample volumes (<1 µL), which enable its use for rapid and sensitive bedside detection of MVs. *Results*: Compared with current standard assays (e.g. Western blotting, ELISA and flow cytometry), this integrated system has a much higher detection sensitivity and can differentiate glioblastoma multiforme (GBM) MVs from non-tumour host cell-derived MVs. In particular, we identified a three-GBM marker combination (EGFR, EGFRvIII and PDPN) that can distinguish GBM-derived MVs from host cell-derived MVs. The system further showed that circulating GBM MVs could serve as a surrogate for primary tumour by reflecting its molecular signature. The amount of MVs also correlated well with tumour volumes and predicted treatment-induced changes. *Conclusions*: GBM-derived circulating MVs were rapidly detected in serum with high sensitivity using a nanotechnology-inspired sensing approach. This new platform would have a wide range of applications, providing both an earlier indicator of drug efficacy and a potential molecular stratifier for human clinical trials.

**Microfluidic capture of tumour-derived extracellular vesicles from serum**

K.E. van der Vos ^1^, N.A. Atai^1^, C.P. Lai^1^, F.P. Floyd^2^, S. Sivaraman^1^, J. Skog^3^, M. Toner^2^, X.O. Breakefield^1^ and S.L. Stott^2^

^1^Department of Neurology and Radiology, Massachusetts General Hospital, Harvard Medical School, Boston, MA, USA; ^2^Center for Engineering in Medicine, Massachusetts General Hospital, Harvard Medical School, Boston, MA, USA; ^3^Exosome Diagnostics, Inc, New York, NY, USA

*Introduction*: Selective isolation of tumour-derived extracellular vesicles (EVs) from body fluids can yield a window into the genetic profile of tumours to serve as diagnostic markers for disease status. *Methods*: We isolated tumour EVs from serum using antibody-mediated capture in microfluidic chambers, coated with antibodies directed against tumour antigens. The device contains eight microchannels with a herring-bone pattern on their upper surface to maximize interactions of EVs with the walls of the chip. Avidin was bound covalently to the surface of the chamber, allowing functionalization with biotinylated antibodies. Efficient EV capture was validated using tumour EVs from various glioma cell lines spiked into healthy human serum. Samples were passed through microfluidic chambers coated with antibodies directed against tumour antigens. Captured EVs were lysed inside the chip and RNA was isolated and compared with input and output RNA. TaqMan assays for tumour-specific messages were performed to analyze the tumour EV capture. *Results*: Initial experiments indicated that high-quality RNA can be isolated from EVs captured inside our microfluidic device. Subsequent TaqMan analysis of tumour-specific messages showed that tumour EVs were enriched in devices coated with an EGFR antibody. This enrichment was illustrated visually by fluorescent imaging of captured EVs from glioma cells expressing palmitoylated GFP. Next, we designed a cocktail of antibodies that can potentially capture tumour EVs from multiple glioma subtypes. Devices coated with this cocktail showed efficient capture of EVs from different human glioma cell lines. *Conclusions*: Tumour-derived EVs in serum can be selectively captured in microfluidic chambers providing information about the genetic status of tumours. In future experiments serum samples from glioma patients will be tested to investigate whether this innovative isolation method can increase sensitivity of detection of oncogenic mutations.

**Immunoelectrophoresis on a microcapillary chip for analyzing exosomes at single-particle level**

T. Ichiki ^1^, T. Akagi^1^, K. Kato^1^, N. Hanamura^1^, N. Kosaka^2^ and T. Ochiya^2^

^1^The University of Tokyo, Japan; ^2^National Cancer Center Research Institute, Japan

Toward the future application of exosomes as a disease biomarker for low-invasive diagnostics, technical challenges lie in the development of sensitive and precise analysis methods for exosomes. In this situation, biodevice technology is expected to provide a new solution to overcome the limitation of traditional analytical methods or tools. Recent progress in biodevice technology has come to provide some innovative tools for biology research such as single cell analysis, and the same situation is also expected in the analysis of nanobiopartices like exosomes. In this presentation, we will present new biodevice technologies toward the evaluation of immunogenicity of exosomes at single-particle level. Human breast cancer cell line (MDA-MB-231) was cultured to confluence in an RPMI medium, followed by the 24-h culture with a serum-free RPMI medium. Exosomes isolated by centrifugation were incubated with an anti-human CD63 antibody for 30 min at 4°C. The samples were introduced into the flow channel of a microcapillary electrophoresis (*f*ÊCE) chip. To perform the electrophoresis experiments, migrating exosomes were recorded under an electric field of 50 V/cm using a laser dark-field microscope. The zeta potentials of exosomes were evaluated from the measured EPM using the Smoluchowski equation. The on-chip *f*ÊCE system with a laser dark-field microscope was found to be particularly suitable for the tracking analysis of individual exosomes and the particle-by-particle measurement of exosome zeta potential was successfully performed. Zeta-potential distributions of exosomes measured before and after reacting with an anti-human CD63 antibody were compared. The zeta-potential distributions of exosomes showed negative shifts after immunoreaction. The results have demonstrated that on-chip immunoelectrophoresis is a promising methodology for single exosome analysis.

**Surface plasmon resonance for highly specific and sensitive detection of exosomes**

Déborah Rupert ^1^, M. Bally^1^, C. Lässer^2^, M. Eldh^2^, J. Lötvall^2^ and F. Höök^1^

^1^Chalmers University, Sweden; ^2^Gothenburg University, Sweden

*Introduction*: Detection of exosome levels could become important in the context of early disease diagnostics and to follow disease progression but, since the level of such nanovesicles is very low, the biosensing platforms must provide high selectivity and sensitivity. As a proof of principle, we present how low levels of CD63-positive exosomes (CD63+) can be detected using surface-based sensing with surface plasmon resonance (SPR). *Materials and methods*: Nanovesicles were isolated from HMC-1 and TF-1 cell culture supernatants and the total particle content was quantified with nanoparticle tracking analysis (NanoSight). CD63+ exosomes present in this complex suspension were specifically detected upon binding to an SPR gold chip (Biacore) which was first passivated with a thiolated and biotinylated oligo(ethylene glycol) self-assembled monolayer and further functionalized with biotinylated anti-CD63 antibodies via a neutravidin linker. Non-specific binding was estimated using a chip coated with biotinylated anti-rabbit antibodies. *Results*: The SPR biosensing platform developed here made it possible to detect with high sensitivity low concentrations of CD63+ exosomes with a detectable signal from below 4 ug/ml of total protein content (TPC). Moreover, excellent specificity was achieved with a non-specific signal <0.5% to the specific one. We further make an attempt to quantify the amount of CD63+ exosomes and to estimate the ratio CD63+/nanovesicles. For this, we exploit the fact that, when operated under mass transport conditions, the exosome binding rate can be directly correlated to the concentration in solution (1). Sample consumption was low and as little as 20 ng from the 4 µg/µl TPC stock was needed to perform a measurement. *Conclusions*: We present a sensitive label-free biosensing platform based on SPR read-out to specifically detect and quantify CD63+ exosomes as a sub-group of nanovesicles.

Reference

1. Karlsson, et al. J Immunol Methods 1993;166:75–84.

**Zeta potential as a parameter for the characterization of exosome interaction**

C. Helmbrecht and H. Wachernig

Particle Metrix GmbH, Germany

In the field of nanoparticle characterization, the zeta potential (ZP) has long been known for the characterization of nanoparticle (NP) stability and NP-surface interactions. However, with conventional techniques, the determination of ZP of low concentrated nanoparticle suspensions, such as exosomes, is difficult. Micro-electrophoresis is a technique suited for this demanding application; the zeta potential is derived from the electrokinetic migration of particles. Particles are illuminated by a laser and the 90° scattering light is visualized by magnification on a CCD camera. In addition to zeta potential, particle counting and sizing of exosomes is performed by digital image analysis in one measurement with the automated particle tracking (APT) technique. Using the Einstein–Smoluchowski relationship, the particle size distribution is derived from the Brownian motion of the particles. The advanced APT technique comprises autofocus and automated sequential measurements on up to 11 points within the measurement cell, supporting the user in obtaining statistical data. Typical applications are, for example, monitoring of particle size distribution and zeta potential while optimizing medium, storing conditions and sample preparation. We utilize the advantages of the micro-electrophoretic setup with emphasis on practical aspects and applications relevant for exosome characterization. Potential applications, such as monitoring of pH sensitive exosome–exosome and exosome–wall interactions in combination with multiparameter characterization will be discussed on selected examples.

**Distinct impact of secreted phospholipase A2 enzymes on detection and quantification of extracellular vesicles**

Matthieu Rousseau ^1^, A.C. Duchez^1^, N. Cloutier^1^, T. Levesque^1^, M. Dieudé^2^, M.H. Gelb^3^ and E. Boilard^1^

^1^Rheumatology and Immunology Research Center, Université Laval, Québec, Canada; ^2^Research Centre, Centre hospitalier de l'université de Montréal (CRCHUM), Montréal, Québec, Canada; ^3^Departments of Chemistry and Biochemistry, University of Washington, Seattle, WA, USA

*Introduction*: Microvesicles (MVs) can be released upon cellular activation and apoptosis. Their precise detection in diverse biological fluids is crucial for the development of biomarkers and the comprehension of their functional activities. A family of soluble proteins, the secreted phospholipase A2 (sPLA2), comprises enzymes that catalyse the hydrolysis of phospholipid membranes. Given that sPLA2 enzymes and MVs are simultaneously present in biological fluids, we aimed to determine whether sPLA2s could digest the MV membrane and thereby impact their detection and quantification. *Materials and methods*: We produced the native murine and human recombinant sPLA2s – IIA, V and X, the 3 most active and abundant sPLA2 enzymes, for our in vitro studies. We examined their impact on endothelial cell-, platelet- and thymocyte-derived MVs from human and mouse species by high-sensitivity flow cytometry using (a) specific integrin antibodies, (b) the phosphatidylserine marker annexin V and (c) fluorescent dyes. For comparison, catalytically inactive sPLA2s mutants were included in our assays. To determine the repercussion of sPLA2s on MVs in vivo, we quantified MVs in blood and thymus from wild-type and mutant mice. *Results*: We demonstrate that distinct sPLA2 enzymes impair the quantification of MVs differently depending on the species studied (human or mouse), the cellular source of MVs and the mean of detection employed. *Conclusion*: It is recognised that the expression of sPLA2s is modulated by diverse stimuli. Considering that sPLA2s are often present in biological fluids and cell cultures, the induction or reduction of the expression of MVs should be interpreted with caution. Depending on the MVs studied, the determination of the sPLA2s is thus recommended.

**Are biologically active exosomes stable in blood plasma?**

Suresh Mathivanan ^1^, M. Hulett^2^, C. Adda^2^, R. Simpson^2^, A. Mechler^2^ and H. Kalra^2^

^1^La Trobe University, Melbourne Victoria, Australia; ^2^La Trobe Institute for Molecular Sciences, La Trobe University, Melbourne Victoria, Australia

Exosomes are biologically active membranous vesicles of endocytic origin released by a variety of cells in vivo and in vitro. As cells release exosomes into the extracellular space, they can also be detected in bodily fluids including saliva, urine, breast milk, blood, amniotic fluid and malignant ascites. Depending on the originating tissue, exosomes contain a subset of the host-cell-specific molecular cargo including proteins, lipids and RNA. The affordability of accessing exosomes in bodily fluids and the presence of host-cell-specific cargo have created immense interest in utilising exosomes for biomarker analysis. Although blood plasma is the specimen of choice for exosomes-based biomarker analysis, its high complexity and viscosity create significant obstacles in isolating pure population of exosomes. In addition, the stability of exosomes in blood plasma is poorly understood. To optimise exosome isolation methods from blood plasma, it is critical to evaluate the commonly used methods in the context of purity. In the first part of this study, a comparative evaluation of 3 exosome isolation techniques (differential centrifugation coupled with ultracentrifugation [UC], epithelial cell adhesion molecule immunoaffinity pull down [EI] and OptiPrep^TM^ density gradient [DG] separation) was performed using normal human plasma. Western blotting, microscopic and mass spectrometry analyses revealed that DG exosomes contained membranous vesicles and were enriched with exosomal markers. However, EI and UC were enriched with high abundant plasma proteins highlighting DG method as relatively superior in isolating pure exosomes.

**Oral Session 9 (Georgian): Platelet-derived EVs April 17**

**Chair: *C. Gardiner and P. Siljander* 15:45-17:45**

Platelets promote the formation of gaps in the inflamed vasculature with dimensions compatible with those of extracellular vesicles

N.C. Cloutier^1^, A.P. Paré^2^, R.W.F. Farndale^3^, R.H.S. Schumacher^4^, P.A. Nigrovic^5^, S.L. Lacroix^2^ and E.B. Boilard ^1^

^1^Rheumatology and Immunology Research Center, Université Laval, Québec, Canada; ^2^CHU de Quebec, Quebec, Canada; ^3^University of Cambridge, Cambridge CB2 1TN, United Kingdom; ^4^University of Pennsylvania, Philadelphia, PA, USA; ^5^Brigham and Women's Hospital, Boston, MA, USA

*Introduction*: There is a growing appreciation of the presence of extracellular vesicles in inflammatory exudates. Although extracellular vesicles can be potentially considered as biomarkers in different diseases, how they may accumulate outside the vasculature is intriguing. Human inflammatory arthritis for instance is associated with tissue oedema attributed to enhanced permeability of the synovial microvasculature. We hypothesised that such vascular leak facilitates entry of submicron vesicles and may thereby promote joint inflammation. *Materials and methods*: We used an in vivo model of autoimmune arthritis and intravital imaging for our studies. *Results*: We demonstrate the presence of endothelial gaps in inflamed synovium with dimension compatible with those of microvesicles. Importantly, these gaps permit the egress of submicron fluorescent microspheres from blood vessels and their specific accumulation in the diseased joints. Surprisingly, permeability in the inflamed joints is abrogated if the platelets are absent. This effect is mediated by platelet serotonin accumulated via the serotonin transporter and can be antagonised using serotonin-specific reuptake inhibitor antidepressants. *Conclusions*: These findings demonstrate that platelets may themselves promote the accumulation of extracellular microvesicles outside the inflamed vasculature by stimulating the formation of endothelial gaps. Whereas platelets typically maintain microvascular integrity, this demonstration that platelets are capable of promoting permeability adds to the rapidly growing list of unexpected functions for platelets.

**Procedures for handling of biofluids for EV analysis: ISEV consensus**

Kenneth Witwer

The Johns Hopkins University School of Medicine, USA

The potential of extracellular RNA molecules as biomarkers of disease and therapeutic targets has intensified interest in extracellular vesicles (EV) as shuttles of RNA in normal and disease states. Paradigm-shifting findings in this area, and particularly in the field of oncology, led recently to a set of exRNA-specific requests for application through the NIH Common Fund. As EV and exRNA research expands, important questions about technical protocols, standards for specimen handling and appropriate normative controls have been raised. Development and implementation of these standards might cast light upon discordant results and facilitate progress in the field. These considerations were the subject of a workshop of the ISEV in New York City in October, 2012. Extended roundtable discussions provided an evidence-based framework for isolation of EV and the purification and analysis of associated RNA molecules. In this presentation, the outcome of these discussions is used as a starting-off point to present the importance of standardisation and complete reporting and to identify a list of controversies and outstanding questions that must be addressed as the field advances.

**Microparticles and vascular markers relationship in patients with essential thrombocythaemia**

A. Piccin ^1^, I. Pusceddu^1^, L. Marcheselli^2^, C. Murphy^3^, E. Eakins^3^, G. Amaddii^1^, C. Moesender Frajria^1^, D. Veneri^4^, O. Perbellini^4^, E. Pacquola^5^, M. Gottardi^5^, F. Gherlinzoni^5^, F. Favaro^6^, D. Faggian^6^, M. Plebani^6^, G. Pizzolo^4^, W.G. Murphy^3^ and S. Cortelazzo^1^

^1^Haematology Department and BMT UNIT, San Maurizio Regional Hospital, Bolzano, Italy; ^2^Department of Onco-Haematology, Modena University Hospital, Italy; ^3^Irish Blood Transfusion Service, Dublin, Ireland; ^4^Department of Haematology, University of Medicine, Verona, Italy; ^5^Department of Haematology, Cà Foncello Hospital, Treviso, Italy; ^6^Department of Laboratory Medicine, University Hospital, Padova, Italy

Essential thrombocythaemia (ET) patients are at risk of developing thrombotic events. Qualitative PLT abnormalities, activation of endothelial cells (EC) and PLT may be involved. Microparticles (MP) may originate from PLT (PMP), EC (EMP) or red cells (RMP) and may affect endothelial modulators such as vasorelaxant nitric oxide (NO), adrenomedullin (ADM) and endothelin-1 (ET-1). We investigated the relationship between MP, NO, ADM and ET-1 in ET patients treated with hydroxyurea (HU), anagrelide (AN) and aspirin (ASA) only. Fifty-two patients with ET diagnosis were studied: 18 on HU, 15 on AN and 19 on ASA. Samples were analysed for MP (absolute total values) and functional markers (percentage values) by flow cytometry, as described (Jy W et al. JTH 2004). PMP was identified using CD62P, CD36 and CD63, EMP using CD105 and RMP using CD235a. Endothelial modulator markers NO, ADM and ET-1 were measured by ELISA. Total-MP was increased in all patient groups compared to controls (p<0.01). Strong correlations were seen for tot-MP and %MP CD62P (R=0.89, p<0.05), tot-MP and%MPCD36 (R=0.97, p<0.05), tot-MP CD62P+ and CD36+ (R=0.85, p<0.05) and for tot-MP TF+ and%MP CD105+ (R=0.66, p<0.05). Bonferroni test showed higher MP total number in the AN group compared to the HU and the ASA group (p<0.05). The percentage of CD63 MP was higher than controls in both the HU and AN groups (p<0.05), while CD36 MP% was higher in the AN group compared to the HU group (p<0.05). NO and ADM values were higher in the HU group, while ET-1 values were lower in the AN group (p<0.05). This study confirms previous findings that MP are present in ET. We also showed that MP are affected by treatment and that MP are mainly PMP. We also showed that HU increases NO and ADM levels, while AN reduces ET-1. As NO and ET-1 have antagonistic actions on endothelial cells, we suggest that HU and AN may have overlapping effects on endothelium, although using different biochemical pathways.

**Activated platelets may deliver messenger RNA regulatory Ago2–microRNA complexes to endothelial cells through microparticles**

Benoit Laffont, A. Corduan, H. Plé, A.C. Duchez, N. Cloutier, E. Boilard and P. Provost

CHUQ Research Center/CHUL, Canada

*Introduction*: Platelets play a crucial role in the cardiovascular system and contain an abundant amount and diversity of microRNAs, which are known as master regulators of messenger RNA (mRNA) translation. Since platelets release microparticles (MP) upon activation, we hypothesised that MP released by activated platelets contain microRNAs that may participate in intercellular signalling and exert extra-platelet mRNA regulatory effects in recipient cells. *Material and methods*: Platelets were isolated from the venous blood of healthy human subjects and stimulated with thrombin. Platelet-derived microparticles (PMP) were characterised by flow cytometry. Internalisation of PMP by cultured human umbilical endothelial vein cells (HUVEC) was analysed by confocal microscopy, whereas the transfer of microRNAs from PMP to HUVEC and the level of an mRNA target of platelet miR-223 were analysed by quantitative PCR (qPCR). *Results*: qPCR and Western blot analyses revealed the presence of microRNAs and of the microRNA effector protein Argonaute 2 (Ago2) in PMP. Immunoprecipitation and mRNA sensor activity assays demonstrated that PMP contain Ago2–microRNA complexes functional in mRNA regulation. We observed internalisation of PMP upon co-incubation with cultured HUVEC and detected the presence of platelet-derived microRNAs, such as miR-223, in HUVEC. PMP-derived miR-223, whose levels in HUVEC persisted for 48 h, could exert extra-platelet mRNA regulatory effects in recipient cells, since the level of endothelial ephrin A1 mRNA, a predicted target of miR-223, was reduced in HUVEC exposed to PMP. *Conclusion*: Our results suggest that PMP may represent a vehicle that can deliver functional platelet-derived Ago2–microRNA regulatory complexes to endothelial cells. Such intercellular signalling events may contribute to the gene expression programming of the endothelium and conditioning of the cardiovascular system under specific health conditions associated with platelet activation.

**Quantitation of miRNAs in extracellular vesicles derived from glioblastoma patients**

J. Akers^1^, V. Ramachandram^1^, J. Skog^2^, X. Breakefield^3^, F. Hochberg^3^, B.S. Carter^1^ and C.C. Chen ^1^

^1^University of California San Diego, USA; ^2^Exosome Diagnostics, USA; ^3^Massachusetts General Hospital, USA

The discovery that glioblastoma cells secrete extracellular vesicles (ECVs) containing tumour-specific genetic material has opened a new platform for biomarker development. Since normal cells also secrete ECVs and non-neoplastic cells typically out-number neoplastic cells by orders of magnitude, tumour-specific ECVs remain a rarity in clinical patient samples. As such, quantitative polymerase chain reactions (qPCR) are typically required for detection and quantitation. Typically, the relative abundance of a query gene is normalised to an abundantly expressed reference gene. This method yields accurate quantitative data only if there is little cell-to-cell variation in terms of the expression of the reference gene. House-keeping genes, such as GADPH and 18S RNA, are expressed at high levels in most cells and exhibit little cell-to-cell variability. Consequently, they are often used as reference genes in qPCR analysis. However, the relative abundance of these genes in ECVs remains poorly characterised. Since the mechanisms by which genetic material is transported into the ECV are largely unknown, it is conceivable that their abundance in ECVs exhibit non-uniform distribution. Despite these knowledge gaps, these genes are frequently used as reference genes for ECV analysis. Here, we report the relative abundance of GADPH, 18S RNA, miR-103 and RNU49, four frequently used reference genes for qPCR analysis of ECV genetic material. In contrast to their abundance in the cellular cytoplasm, the levels of these transcripts in cell line-derived ECVs were low. Moreover, the abundance of these transcripts in ECVs isolated from different cell lines varied by an order of magnitude. We observed significant sample-to-sample variation in terms of their presence in the ECVs derived from patient serum and cerebrospinal fluid. Our results suggest that normalising ECV content by the four genes studied here may arbitrarily bias the quantitative analysis.

**Platelet microparticle-associated immune complexes induce the production of pro-inflammatory mediators in human neutrophils**

L.H. Boudreau ^1^, N. Cloutier^1^, S. Tan^2^, C. Cramb^3^, R. Subbaiah^4^, L. Lahey^3^, A. Albert^1^, R. Shnayder^5^, R. Gobezie^6^, P.A. Nigrovic^5^, R.W. Farndale^7^, W.H. Robinson^3^, A. Brisson^2^, D.M. Lee^8^ and E. Boilard^1^

^1^Rheumatology and Immunology Research Center, Université Laval, Canada; ^2^UMR-CBMR Université Bordeaux-1, France; ^3^Stanford University School of Medicine, USA; ^4^Case Western Reserve University, USA; ^5^Havard Medical School, USA; ^6^University Hospitals of Cleveland, USA; ^7^University of Cambridge, United Kingdom; ^8^Novartis Institutes for Biomedical Research, Switzerland

*Introduction*: Immune complexes (IC) typically comprise immunoglobulins, antigens and complement. ICs are recognised to propagate inflammation in autoimmune diseases such as systemic lupus erythematosus (SLE) and rheumatoid arthritis (RA). Intriguingly, we and others demonstrated that immunoglobulins and microparticles frequently associate in SLE and RA, thus forming true microparticle ICs (mpICs). Given that lipid mediators derived by neutrophils are active players in chronic inflammatory disorders, we hypothesised that mpICs may trigger neutrophil activation and lipid mediator production. *Material and methods*: To determine the potency of mpICs on neutrophils, we initially had to delineate how to produce mpICs in vitro. In this first aim, we employed high sensitivity flow cytometry, electron microscopy and proteomic approaches to comprehend the mpIC structure. Human neutrophils were next incubated in the presence of the mpICs we generated in vitro and the release of lipid mediators was assessed using reverse-phase high-performance liquid chromatography. *Results*: We observe that the mpICs in RA synovial fluid frequently express the integrin CD41, consistent with platelet origin. Since these platelet MPs exhibit fibrinogen and associate with anti-fibrinogen autoantibodies in RA synovial fluid, the mpICs were produced in vitro by co-incubating MPs and anti-fibrinogen. We find that neutrophils promptly release inflammatory lipid mediators upon incubation with mpICs in an Fc receptor-dependent manner. Conversely, MPs and anti-fibrinogen are inactive on neutrophils when added separately. *Conclusion*: Taken together, our data suggest a unique role for platelet MPs as autoantigen-expressing elements capable of perpetuating formation of active inflammatory ICs.

**Blood platelets take up exosomes and can be used as diagnostics platform for tumour-derived RNA biomarkers**

J. Nilsson^1^ and T. Wurdinger ^2^

^1^Umea University, Sweden; ^2^VU University Medical Center, Netherlands

*Introduction*: Diagnostic platforms providing biomarkers which are highly predictive for diagnosing, monitoring and stratifying cancer patients are key instruments in the development of personalised medicine. *Results*: We demonstrate that tumour cells transfer (mutant) RNA via microvesicles into blood platelets. We show that blood platelets isolated from various cancer patients contain cancer-associated RNA biomarkers. In addition, gene expression profiling revealed distinct RNA signature in platelets from cancer patients as compared to normal control subjects. *Conclusion*: Because platelets are easily accessible and isolated, they may form an attractive platform for the companion diagnostics of cancer.

**Ex vivo vesiculation is inhibited in acid-citrate dextrose anticoagulant tube enabling the improved assessment of extracellular vesicles**

B. Gyorgy^1^ , K. Paloczi^1^, A. Kovacs^1^, E. Barabas^1^, G. Beko^1^, K. Varnai^1^, E. Pallinger^1^, K.E. Szabo-Taylor^1^, G.T. Szabo^1^, A.A. Kiss^2^, A. Falus^1^ and E.I. Buzas^1^

^1^Semmelweis University, Hungary; ^2^Military Hospital, National Health Institute, Hungary

*Introduction*: The analysis of microvesicles (MVs) in biological fluids is confounded by many factors such as the activation of blood cells in the collection tube that leads to ex vivo vesiculation. In this work, we aimed at analysing the effect of anticoagulation on MV levels in blood plasma samples and on artifactual ex vivo vesicle formation. We also analysed RNA content in citrate and ACD tubes. *Materials and methods*: Blood was drawn into blood collection tubes containing different anticoagulants from 30 healthy volunteers. We compared the levels of platelet MVs (PMVs) and non-PMVs in platelet-free plasma (PFP) by flow cytometry and Zymuphen assay. The extent of ex vivo vesiculation (induced by agitation and elevated temperature) was compared in citrate and acid-citrate-dextrose (ACD) tubes. We examined size distributions of MVs in samples using qNano. RNA content was analysed from plasma and total extracellular vesicles from citrate and ACD tubes using bioanalyser and miRNA qRT-PCR assays. *Results*: Anticoagulation substantially influenced both the PMV and non-PMV levels. Agitation and storage of blood samples at 37°C for 1 hour induced a strong release of both PMVs and non-PMVs. Strikingly, ex vivo vesiculation was prevented in plasma samples using ACD tubes. Importantly, MV levels elevated in vivo (in plasma samples from healthy pregnant women) were not confounded in ACD tubes. There was no difference in the size distribution of MVs collected into citrate and ACD tubes. RNA profiles were similar between ACD and citrate tubes, mostly small RNAs were detected both in plasma and vesicles. We confirmed the presence of miR16, miR24, miR451 and let7a in the plasma samples and in extracellular vesicles isolated from citrate and ACD tubes. *Conclusion*: We propose the general use of the ACD tube for the assessment of plasma MVs based of the efficient inhibition of ex vivo vesiculation in ACD tubes. Furthermore, we show that ACD tube is also applicable for RNA analysis.

**Caris-sponsored Satellite Symposium (Imperial Ballroom) 18:00-19:00**

**Poster Sessions I-II 8:30-18:00**

**Poster Session I (Arlington-Berkeley): Biomarkers April 17**

**Chair: *E.I. Buzas and J. Lötvall***

**Beaming QRT-PCR analysis of mutant IDH1 mRNA in tumour extracellular vesicles**

Leonora Balaj ^1^, Walter W. Chen^2^, Linda M. Liau^3^, Casey A. Maguire^2^, Lori LoGuidice^4^, Horacio Soto^3^, Matthew Garrett^3^, Lin Dan Zhu^2^, Sarada Sivaraman^2^, Eric T. Wong^5^, Bob S. Carter^6^, Fred H. Hochberg^2^, Xandra O. Breakefield^2^ and Johan Skog^2^

^1^Massachusetts General Hospital, USA; ^2^MGH, USA; ^3^UCLA, USA; ^4^Exosome Diagnostics Inc, USA; ^5^BIDMC, USA; ^6^UCSD, USA

*Introduction*: A promising source of biofluid-based biomarkers are extracellular vesicles (EVs); they are actively released from normal as well as transformed cells and end up in the surrounding biofluids. These EVs could potentially be used to non-invasively monitor the molecular phenotype of tumours over time. We are investigating whether the mutant IDH1 is detected in the EVs from blood or cerebrospinal fluid (CSF) of glioma patients. *Materials and methods*: Single nucleotide mutations are very difficult to detect when the fraction of mutated transcripts is low. To increase the sensitivity of the assay we adapted a well-established and highly sensitive form of digital PCR known as BEAMing to examine the mRNA within EVs for the presence of mutant IDH1. *Results*: We have analysed CSF from 8 glioma patients where the mutation in IDH1 has been confirmed by sequencing the tumour tissue, as well as 6 CSF samples from glioma patients with wild-type IDH1, and two healthy controls. Using BEAMing PCR we report 63% sensitivity and 100% specificity for the IDH1 mutation in CSF. The 2 false-negative patients tended to be grade II tumours indicating that small tumours may be more difficult to detect. BEAMing analysis was also performed on 4 serum samples from glioma patients with the IDH1 mutation as well as 4 serum samples from healthy controls. So far we have not been able to detect the IDH1 mutation in serum samples. Interestingly the copy number of IDH1 was significantly lower in serum samples when compared to CSF samples, so increasing the serum input may improve sensitivity. The IDH1 mutation results will also be assessed using droplet PCR in collaboration with RainDance. *Conclusions*: MV-BEAMing can be used to reliably detect the mutation status of IDH1 (and presumably other genes) through a CSF sample. This is a non-invasive alternative to biopsies for clinicians to do molecular classification of MRI lesions in the brain.

**Selection of antibodies recognising melanoma exosomes in in vitro models and in human plasma samples**

A. Corrado, G. Radano, E. Lari, A. Chiesi and N. Zarovni

Exosomics Siena SpA, Italy

*Introduction*: Detection of tumour-associated exosomal markers in human plasma provides “liquid biopsy” as a non-invasive approach to screening and early detection of cancer. Its implementation in research and clinics is dependent on definition and validation of exosomal tumour markers as diagnostic targets for immunoassay development. We analysed a panel of antibodies (Ab) directed against putative melanoma markers on exosomes from tumour cell lines, tumour patients and healthy plasma samples. Binding kinetics and efficiency of these Ab in capturing and detection of melanoma-related exosomes is assessed by FACS, label-free detection and ELISA (ExoTESTTM). *Material and methods*: Exosomes were purified by ultracentrifugation from supernatants and human plasma samples. Panel of Ab against melanoma-associated antigens were used to perform FACS analysis of bead-conjugated exosomes or binding assessment to sensor-bound exosomes. Selected Ab were differently combined and used as exosome capture and detection reagents in ELISA (ExoTESTTM). *Results*: Ab used in the study address acknowledged exosome markers, general cancer or melanoma markers, tumour progression indicators, stromal and other proteins with distinct biological function and pathological rational. We identified several Ab displaying differential binding affinity and specificity to melanoma exosomes in vitro, and between patients and healthy individuals. Marker expression profiles differ between cell cultures and human plasma samples limiting thus translational significance of proteomic characterisation of in vitro tumour models. *Conclusions*: Accurate selection of reagents, methods and reference samples enable efficient Ab screening for identification of melanoma-associated exosomal markers and optimisation of versatile ELISA-enabling selective capture and detection of tumour exosomes from complex biological samples. Validation of assay resolution potential and performance is warranted in an enlarged set of clinical samples.

**Study of miRNAs in vesicular and non-vesicular fractions extracted from plasma before and after a half marathon race**

R.W. Pereira, G.P. Oliveira Jr, C.P.C. Gomes and B. Madrid

Catholic University of Brasilia – Genomic Science and Biotechnology Graduate Program, Brazil

*Introduction*: The endurance exercise plays a decisive role in health promotion and protection against diseases. However, the molecular mechanisms related to how physical activity is essential for human health is not well known. miRNAs appeared in the scientific literature 19 years ago. They play a pivotal role as effectors in the control of gene expression. Their presence in serum or plasma brought the promises of a new class of biomarkers. These circulating miRNAs can be transferred horizontally from one tissue to another through vesicles, taking messages not yet fully understood by the scientific community. *Material and methods*: We analysed the level of five circulating miRNAs (miR-16, -let-7a, -1, -133a and -206) into two plasma fractions (supernatant and vesicular) of 10 recreational athletes after running a half marathon (21 km), as well as the stability of such miRNAs after treatment with proteinase K (PK). *Results*: The results showed a decrease of miRNAs miR-16, miR-1, miR-133a, slight increase in miR-let7a and an extraordinarily large increase in miR-206 in the vesicular fraction after the marathon. The treatment with PK led to a strong decrease of miR-16 and miR-133a in the plasma supernatant fraction. This indicates that these circulating plasma miRNAs may be associated with protein complexes. Otherwise, miR-let7a, miR-1 and miR-206 had no substantial reduction in plasma levels in the supernatant and vesicular plasma fractions after treatment with PK, which indicates that these miRNAs circulate in plasma associated with the lipid vesicles. *Conclusions*: The results suggest that plasma levels of miR-206 associated with vesicular fraction increases strongly in response to endurance exercise, it being the muscle-specific miRNA studied that showed higher response after the exercise stimulus. It would be valuable to understand the target genes regulated by miR-206 in the tissues targeted by microvesicle carriers of miR-206.

**Detection of circulating microRNAs in colorectal cancer**

S.G. Jensen ^1^, D.K. Jeppesen^1^, H.J. Nielsen^2^, T.F. Ørntoft^1^, M.S. Ostenfeld^1^ and C.L. Andersen^1^

^1^Department of Molecular Medicine, Aarhus University Hospital, Skejby, Denmark; ^2^Department of Surgical Gastroenterology, Hvidovre Hospital, Denmark

*Introduction*: It is expected that detection of colorectal cancer (CRC) at an earlier stage, than is done today, will improve the overall survival for this group of patients. A potential diagnostic approach for early detection of CRC could be profiling of circulating microRNAs (miRNAs). miRNAs in blood are believed to be either protein bound or contained in exosomes. The aim of this study was to investigate the feasibility of using miRNA profiles from exosomes isolated from plasma as novel biomarkers for early detection of CRC. *Material and methods*: The study was initiated by evaluation of methods for isolation of tumour-derived exosomes and for profiling miRNAs with low abundance. Dynabeads were used to isolate exosomes expressing EpCAM. Western blotting and transmission electron microscopy (TEM) were used to verify successful isolation of exosomes and the presence of EpCAM. qRT-PCR was conducted to profile the miRNA expression. *Results*: Western blotting of three proteins and TEM revealed successful isolation of exosomes secreted from SW620 and SW480 colorectal cancer cell lines. Furthermore, western blotting demonstrated expression of EpCAM on exosomes and verified successful isolation of exosomes using Dynabeads. Using serial dilutions of isolated exosomes spiked into PBS or plasma, a high linearity in miRNA signal vs. input material was observed using EpCAM beads as opposed to negative IgG control beads. Furthermore, miRNA fold changes observed between cancer and control plasma samples were improved when quantification on EpCAM+ exosomes rather than total plasma miRNA was carried out. *Conclusions*: Specific miRNAs associated with EpCAM+ exosomes present in the plasma showed potential to distinguish cancer patients from healthy individuals.

**MicroRNA profiles from serum and plasma-derived exosomes utilising miRCURY LNA™ Universal RT microRNA PCR**

Thorarinn Blondal, M.W. Teilum and P. Mouritzen

Exiqon AS, Denmark

*Background*: MicroRNAs function as post-transcriptional regulators of gene expression. The high relative stability of microRNAs in common clinical tissues (e.g. FFPE) as well as biofluids (e.g. plasma and serum) combined with utilising microRNA expression profiles to accurately classify discrete tissue types and disease states have positioned microRNA quantification as a promising tool for a wide range of diagnostic applications. The microRNAs in serum and plasma are protected from degradation by being encapsulated in exosomes and/or bound to protein complexes (e.g. HDL and Ago2). Comparison of the different microRNA cell-free content in these compartments is of interest when assessment is made of the suitable source of microRNA for biomarker studies. To facilitate discovery and clinical development of microRNA-based biomarkers, we developed the genome-wide miRCURY LNA™ Universal RT microRNA PCR platform with unparalleled sensitivity and robustness. This platform has allowed us to generate reference ranges for the expression of microRNA based on thousands of plasma samples. *Methods*: Many different specific exosome purification methods have been described to date, including the traditional differential centrifugation as well as more general methods based on precipitation or column binding and other more specific procedures based on capturing exosomes with antibodies followed by isolation. Pools of either plasma or serum samples were made and exosomes purified using several of these different exosome purifying methods. RNA was isolated from exosome-enriched, exosome-depleted and non-enriched sources to address the robustness and reproducibility of different methods. The samples were profiled using the LNA™-based Serum/Plasma Focus microRNA PCR panels. *Results*: The miRNA profiles of different exosome isolation methods will be presented and compared to evaluate the differences between methods and standard profiles of whole plasma and serum.

**Proteomic characterisation of exosomes in expressed prostatic secretions associated with prostate cancer**

Simona Principe, Yunee Kim, E. Ellen Jones, Thomas Kislinger and Richard R. Drake

UHN-OCI, Canada

*Introduction*: Expressed prostatic secretions (EPS) are prostate-proximal fluids used as a clinical source for diagnostic and prognostic assays for prostate cancer (PCa). EPS include direct EPS (dEPS), collected from the prostate before prostatectomy, and EPS urine (EPSu), expelled in voided urine after digital rectal examination (DRE). Both fluids are rich in nano-sized vesicles that harbour proteins, RNAs and lipids and represent a valuable source for biomarker discovery. The goal of the present study is to characterise EPSu exosomes by employing shotgun proteomics to uncover their protein landscape in the context of Pca. *Material and methods*: EPSu samples were collected from 12 patients with low grade PCa and 12 non-cancers. Exosomes were purified by differential ultracentrifugation. Proteins were extracted using trifluoroethanol and exosomal proteins were identified and quantified using a Q Exactive mass spectrometer and the MaxQuant analysis pipeline. *Results*: Our initial proteomics analyses revealed that the EPSu-derived exosomes contained>500 proteins, including markers as CD63, CD9 and CD81. Functional GO terms analyses representing biological processes showed enrichment in vesicle transport and metabolic process; the cellular component term was enriched in cytoplasmic membrane-bounded vesicle and endosome, supporting the multivesicular origin of these particles. Using our previously generated proteomic datasets on dEPS, EPSu, publicly available data on urinary exosomes and 5 different PCa cell lines, we created a highly annotated resource of exosomal proteins in EPSu. Using label-free quantification we generate a panel of proteins associated with PCa that can serve as candidates for future biomarker studies. *Conclusions*: This study provides a description of the protein cargo of as-derived exosomes, in the context of PCa. Detailed analyses of the derived proteome will set the stage for future evaluations as potential PCa biomarkers using larger patient cohorts.

**Identification and implications of transcription factors in circulating microvesicles from cancer patients**

D. Holterman, T. Hornung, S. Kankipati, S. Smith, P. Kennedy, M. Maheshwari, C. Alva, A. Benton, S. Logie, W. Chen and D. Spetzler

Caris Life Sciences, USA

Circulating microvesicles (cMV) are small membrane-bound particles that play important roles in the pathogenesis of many human diseases including heart disease, autoimmunity and cancer. cMV are known to contain proteins and RNA molecules derived from their cell of origin, but until recently little was known about the presence of transcription factors (TF) within disease-associated cMV. Recently, researchers have identified TF within cancer-associated cMV, including c-Myc, p53, AEBP1 and HNF4a. Using multiparametric flow cytometry and an antibody sandwich assay, several TF were identified in prostate cancer (PCA) cMV. STAT3 was identified in permeabilised exosomes from the PCA cell line VCaP, and STAT3+ cMV from PCA patients was elevated when compared to non-cancer males. Additionally, analysis on isolated cMV from breast cancer and non-cancer female plasma revealed that the signal for a Y-box cell cycle-associated TF was higher in breast cancer cMV compared to those from non-cancer female controls. A prostate tissue-specific ETS-associate transcription factor (PTE-TF) was elevated in cMV from biopsy-confirmed PCA plasma compared to non-cancer prostate conditions in men undergoing prostate biopsies to rule out PCA. Specifically, the mean fluorescence of PTE-TF in men with benign diagnosis (n=39) was 91, inflammatory prostatic disease (n=29) was 101, cellular atypia (n=8) was 68, HGPIN (n=21) was 102 and PCA (n=80) was 188. This higher trend for PTE-TF expression in cMV with increasing risk of prostate malignancy suggests that PTE-TF in cMV may assist in the treatment of PCA in high risk men. Lower cellular PTE-TF has been associated with more aggressive phenotypes and higher Gleason score indicating that shedding of this TF into cMV may play an important and previously unrecognised role in PCA progression by actively reducing cellular levels.

**Healthy vs polycystic kidney disease: a study on microRNA content of extracellular vesicles from rats urine**

A. Moggio ^1^, H. Dweep^2^, C. Sticht^2^, G. Camussi^1^, B. Bussolati^3^ and N. Gretz^2^

^1^Department of Medical Sciences, University of Turin, Italy; ^2^Medical Research Center, Medical Faculty Mannheim, University of Heidelberg, Germany; ^3^Department of Molecular Biotechnology and Health Sciences, University of Turin, Italy

*Introduction*: Autosomal dominant polycystic kidney disease (PKD) is the most common hereditary renal pathology characterised by cysts formation and progressive renal failure. Our aim was to analyse urinary extracellular vesicles (EVs) of rats with PKD mutations to gain insight on the pathogenesis and progression mechanisms. *Material and methods*: Urine was obtained from PKD rats model and from healthy rats (SD) placed in metabolic cages overnight. The collected urine was centrifuged at 3000 rpm and ultracentrifuged at 100,000*g* for 1 h at 4°C. Singles EVs collections were pooled together and resuspended in DTT (200 mg/ml) and ultracentrifuged again. EVs were quantified and characterised and processed for a miRNA analysis (Affymetrix). *Results*: The characterisation showed different number (13.25×10^11^ particles/ml in SD urine and 3.73×10^11^ particles/ml in PKD urine) and size (mean: 109±45 nm in SD and 210±83 nm PKD) with the appearance of a subpopulation of bigger vesicles in PKD urine. Microarray analysis showed a differential expression of 86 miRNAs. miRNAs target analysis allowed identification of several mRNAs already found differentially regulated in PKD renal tissue and highlighted the deregulation of endocytosis and fibrogenesis pathways. *Conclusion*: Microarray analysis provided data for the identification of microRNAs involved in fibrogenesis, a stage of the disease development, suggesting that EVs released in the urinary apparat may be involved in the communication and expansion of the cysts. Moreover, the presence of the change of released vesicles may suggest the deregulation of endocytosis/ectocytosis pathways.

**Poster Session I (Arlington-Berkeley): RNA Analysis April 17**

**Chair: *L. Balaj and H. Tahara***

**RNA ExoTESTTM: a novel multiplatform for exosomes capture, ELISA and RNA analysis**

P. Guazzi, T. Oja, J. Muhhina, A. Chiesi and N. Zarovni

Hansabiomed OU, Estonia

*Introduction*: Exosome-shuttled proteins and RNAs are indicative of the parent cell type and condition, featuring novel source of diagnostic biomarkers. ExoTESTTM is an immunocapture platform that enables isolation, quantification and characterisation of overall and/or specific exosomes from biological samples facilitating thus detection of known and novel biomarkers. We combined the use of ExoTESTTM for direct capture of exosomes from human plasma and several methods for total RNA extraction and analysis. We compared the yield, composition and quality of RNA extracted from immunocaptured exosomes vs. exosomes prepared by ultracentrifugation and addressed the isolation efficiency determined by different capturing antibodies in healthy and tumour patients’ plasma samples. *Material and methods*: Exosomes from human plasma were purified by ultracentrifugation or captured on ExoTESTTM plates and used for total RNA extraction using phenol-based, column-based and combined techniques. RNA yield and purity was measured by spectrophotometer and using Agilent Bioanalyzer that also addressed RNA size distribution in differently processed samples. The quality of RNA was further assessed by RT-PCR. *Results*: Our data demonstrate that ExoTESTTM is a highly efficient tool for specific plasma exosome capture that enables high yield and integrity of recovered exosomes and allows contemporary analysis of multiple protein and RNA markers. We report optimised protocols for multifold increase in RNA yield and confirm the enrichment in small RNAs. *Conclusions*: ExoTESTTM represents a novel, reliable and easy solution for isolation and analysis of total exosome population in plasma or a defined exosome sub-population, likely reducing the background that interferes with the analysis of RNA sequences. Co-detection of overlapping protein and RNA markers results in a realistic potential to accurately distinguish different sample types and conditions.

Funded by: EAS EU29269

**Contribution of foetal calf serum exosomal RNA in in vitro experiments**

G. Shelke, C. Lässer and J. Lötvall

Krefting Research Centre, University of Gothenburg, Sweden

*Background*: Exosomes are present in many biological fluids, including serum. Cell culture media is often supplemented with foetal calf serum (FCS), which may contribute to exosome extracted from any cell culture. Ultracentrifugation of FCS has been suggested to eliminate FCS exosomes. This study aims to determine whether RNA in FCS exosomes can contribute to RNA in exosomes from cell cultures and whether the FCS exosome contamination can be eliminated by depletion steps. *Materials and methods*: Exosomes from FCS (several batches) or FCS-supplemented cell culture medium were isolated using centrifugation and filtration steps. Isolation and profiling of exosomal RNA was performed using miRCURY and Bioanalyzer. Exosomes were obtained from media with (w) and without (w/o) cells (human mast cell-1: HMC1). Cell culture media was supplemented with FCS (10%) prior to exosomes isolation. FCS exosomes were depleted by 0, 1.5 or 18 h of centrifugation at 120,000*g*. *Results*: Ultracentrifugation substantially reduced the total FCS exosomal RNA content. Detectible but significantly reduced exosomal RNA was still possible to identify in exosomes in FCS after 90 min of ultracentrifugation. The step of 1.5 h centrifugation did not remove vesicular small RNA (30–100 bp). In cell cultures, the percentage contributions of RNA from FCS exosomes are approximately 4, 1 and 0.5% after 0, 1.5 and 18 h of pre-culture centrifugation of FCS in HMC1 supernatant. *Conclusion*: Even after different depletion steps, FCS exosomes still contribute to a small extent to the RNA found in exosomes from cells cultured in the presence of FCS, even though the FCS has been filtered and “exosomes depleted”. Such contribution of FCS exosomes to isolated exosomal RNA should be minimised as much as possible and may especially be considered in the analysis of low abundance RNAs in deep sequencing data.

**Investigations of anticoagulant, freeze–thaw cycles and RNA isolation methods in EV research and application of methods to a model of HIV-1 disease**

Kenneth Witwer, D. Muth, N. Sangal and M. McAlexander

The Johns Hopkins University School of Medicine, USA

*Introduction*: Discussions in the extracellular vesicle research community, including at recent ISEV events, have highlighted the need for rigorous investigations of the influence of pre-analytical variables on EVs and downstream RNA assays. We investigated the influence of anticoagulant, freeze–thaw cycles on overall plasma particle and EV populations. We also tested RNA isolation methods for PCR inhibitor removal. Finally, we used optimised methods to examine samples from a model of HIV-1 CNS disease. *Material and methods*: Blood was collected from healthy donors with anticoagulants, including sodium citrate, EDTA, ACD and lithium heparin. Blood was processed immediately to obtain “platelet-free” plasma. Nanoparticle tracking analysis was performed with or without a lipid dye, and quantum dot-conjugated antibodies to surface proteins were also tested. Changes in overall particle concentration in blood and cerebrospinal fluid were assessed by nanoparticle tracking analysis before freezing (−80°C) and following one and two freeze–thaw cycles. RNA was isolated from whole and fractionated plasma using up to four different methods in parallel and examined by RT-qPCR. We then analysed plasma samples obtained serially pre- and post-infection from an animal model of HIV-1 CNS disease. *Results*: Anticoagulant had no significant effect on apparent circulating particle number when blood was processed within 30 min of draw. Effect of freeze–thaw cycle on plasma and CSF particle counts was minimal. Dramatic differences in RNA recovery and/or PCR inhibitor co-purification were observed between RNA isolation methods. *Conclusions*: NTA indicates minimal effects of anticoagulant and freezing on particle size and number, but specific classes of EVs possibly respond significantly. As expected, some anticoagulants inhibit PCR assays. Newly available biofluids RNA isolation protocols provide improvements over previous methods; however, RNA extraction should always be optimised carefully.

**Visualisation and tracking of extracellular vesicle-mediated RNA delivery**

C.P. Lai, O. Mardini and X.O. Breakefield

Massachusetts General Hospital, Harvard Medical School, USA

*Introduction*: Due to their nanoscale size, extracellular vesicles (EVs) can only be visualised in real-time by microscopy techniques using EV-associated proteins fused to fluorophores, and/or fluorescent chemical labelling. Meanwhile, studying the dynamics of RNA delivery by EVs has been challenging due to size and reporter sensitivity issues. Here we developed a dual-function reporter system to visualise and track both EVs and EV-mediated RNA delivery. *Material and methods*: To visualise EVs, GFP or tdTomato were fused with a palmitoylation signal at the N-terminus (palm-GFP, palm-tdTomato) and expressed in EV-producing cells followed by EV isolation. To monitor EV-RNAs, the palm-tdTomato transcripts were tagged with MS2 RNA-binding sequences (palm-tdTomato-MS2) and detected by bacteriophage MS2 coat protein fused with GFP (MS2CP-GFP). *Results*: Palm-GFP/tdTomato-MS2 reporters were confirmed to be EV-specific by sucrose gradient centrifugation of isolated EVs followed by western blotting. The EVs were readily visible under live-cell confocal microscopy. This EV-labelling strategy also encompasses CD63 EV populations as shown by a co-localisation of signals between CD63-GFP and palm-tdTomato-MS2 in EVs isolated from cells co-expressing the two EV reporters. Interestingly, in EV-recipient cells, we observed a different sub-cellular EV distribution pattern over time following EV treatment between EVs co-labelled with either palm-tdTomato and PKH67, or palm-tdTomato and CD63-GFP. EVs isolated from cells co-expressing palm-tdTomato-MS2 and MS2CP-GFP revealed signal co-localisations between tdTomato and GFP, indicating visualisation of EVs and EV-packaged MS2 RNAs, respectively. *Conclusions*: To our knowledge, this is the first report of a dual-function reporter strategy that enables live-cell detection and tracking of both EV and its RNA cargo. Studies are underway in examining EV uptake, EV-RNA release and their subsequent fates in EV-recipient cells.

**RNA Profiling In Human Plasma Exosomes By Deep Sequencing**

L.W. Wang^1^, T.Y. Yuan^1^, M.T. Tschannen^1^ Z.S. Sun^2^, H.J. Jacob^1^, M.D. Du^1^, M.L. Liang^3^, R.D. Dittmar^1^, Y.L. Liu^1^, M.L. Liang^1^, M.K. Kohli^2^, S.T. Thibodeau^2^, L.B. Boardman^2^ and X.H. Huang ^1^

^1^Medical College of Wisconsin, United States; ^2^Mayo Clinic, United States; ^3^Second Affiliated Hospital of Harbin Medical University, China

*Introduction*: Exosomes contain a specific set of RNA transcripts that are involved in cell-cell communication and hold potentials as disease biomarkers. To systemically characterize exosomal RNA profiles, we performed RNA sequencing analysis using three human plasma samples and evaluated efficacies of small RNA library preparation protocols from 3 manufacturers. *Results*: From 14 (7 replicates) size-selected sequencing libraries, we received a total of 101.8 million raw single-end reads, ~7.27 million reads per library on average. We observed a diverse collection of RNA species, of which microRNA (miRNA) was the most abundant, contributing to over 42.32% of all raw reads and 76.20% of all mappable reads. At the current read depth, we detected a total of 593 miRNAs, of which the five most common miRNAs (miR-99a-5p, miR-128, miR-124-3p, miR-22-3p, and miR-99b-5p) collectively accounted for 48.99%. miRNA target gene enrichment analysis found that these high abundant miRNAs may play an important role in biological functions such as protein phosphorylation, RNA splicing, chromosomal abnormality and angiogenesis. From unknown RNA sequences, we predicted 185 potential miRNA candidates. Furthermore, we detected significant fractions of other RNA species including: rRNA (9.16% of all mappable counts), lncRNA (3.36%), piRNA (1.31%), CDS (1.36%), tRNA (1.24%), 5'UTR (0.21%), 3'UTR (0.54%), snRNA (0.18%), and snoRNA (0.01%). In addition to the RNA composition, we found that all tested commercial kits were able to generate sufficient DNA fragments for sequencing but each with significant bias toward capturing specific RNAs. *Conclusions*: This study demonstrated a variety of RNA species embedded in the circulating vesicles. Further characterization of these extracellular RNAs in the diverse human population will provide reference profiles and open new doors for the development of blood-based biomarkers for human diseases.

**Poster Session I (Arlington-Berkeley): Isolation April 17**

**Chair: *R. Weissleder and R. Nieuwland***

**Presence of GM1 ganglioside and absence of phosphotidylserine on EV surface: a parameter to define and isolate exosomes**

Ronne Wee Yeh Yeo, S.S. Tan, R.C. Lai and S.K. Lim

Institute of Medical Biology, A*STAR, Singapore

*Introduction*: Extracellular vesicles (EVs) encompass all membrane vesicles produced by cells. They are implicated in many physiological and pathological processes. Efforts to study EVs are limited by the lack of tools to differentiate and isolate different classes of EVs. We have previously described an HPLC-purified population of homogeneously sized MSC-secreted vesicles as “MSC exosomes”. Here we report that despite size homogeneity, “MSC exosomes” could be further fractionated by their membrane lipid components into 2 fractions, each with a distinct cargo. *Materials and methods*: Membrane vesicles were purified by HPLC from a serum-free chemically defined medium conditioned for 72 h by hESC-derived MSCs (Lai et al., 2010). Vesicles with GM1 ganglioside-enriched membranes or exposed phosphotidylserine (PS) were isolated by their affinity to cholera toxin B (CTB) or annexin V (AV) respectively, using magnetic bead technology. Exosomal markers were detected by standard ELISA or immunoblotting. *Results*: Analysis of HPLC-purified vesicles revealed that 10% of them have GM1 ganglioside-enriched membranes and bind CTB. Exosomal markers such as Alix, Tsg101 and CD9 were exclusively detected in CTB-binding vesicles. While no vesicles with exposed PS were detected in the CM from healthy cells, CM from cells treated with staurosporine and undergoing apoptosis showed presence of AV-binding vesicles. We also showed that CTB-binding vesicles have a density of 1.10–1.19 g/ml, typical of exosomes. Most interestingly, their proteome was distinct from that of non-CTB-binding vesicles and is enriched with endosomal markers. *Conclusions*: Exosomes have exposed GM1 gangliosides but not PS. This lipid feature could not only further delineate exosomes from other EVs, it could also be exploited to isolate exosomes. Unlike conventional exosome isolation technologies, membrane lipid-based technologies discriminate membrane vesicles from other similarly sized biological aggregates.

**Re-assessing distribution of “exosome” markers within sub-populations of extracellular vesicles**

J. Kowal, M. Colombo and C. Théry

Institut Curie, INSERM U932, France

*Introduction*: In multicellular organisms, cells interact by various means, including secretion of extracellular vesicles whose content can influence the behaviour of other cells. Some vesicles are shed from the cells’ plasma membrane, whereas exosomes are formed in multivesicular endosomes and secreted upon fusion of these compartments with the plasma membrane. A widely used protocol of exosome purification involves removing large vesicles by 10,000*g* centrifugation, followed by pelleting small vesicles at 100,000*g*. The resulting pellet qualifies as exosomes if enriched in endosomal markers. But we recently observed that vesicles obtained this way from mouse tumour cells contain at least two distinct sub-populations of vesicles, secreted by different molecular machineries, and differentially enriched in some exosome markers (Bobrie et al, JEV 2012). We have now performed extensive re-assessment of the distribution of proteins classically used as exosome markers in the different sub-populations of vesicles. *Materials and methods*: Vesicles were purified by differential ultracentrifugation and sucrose gradient floatation from conditioned medium of human monocyte-derived dendritic cells (DCs) and SKOV3 ovarian carcinoma cell line. Vesicles were analysed by western blotting, electron microscopy and NanoSight. *Results*: In our culture conditions, SKOV3 secretes very few vesicle-associated markers, mainly recovered after 100,000*g* centrifugation, whereas DCs secrete many markers recovered in all pellets. Floatation into sucrose shows that DCs’ 10,000 versus 100,000*g* pellets float mainly respectively at 1.19 g/ml versus 1.14 g/ml. In the 100,000*g* pellet, tetraspanins, MHC class II and flotillin-1 are differently enriched in the 1.14, 1.17 and 1.19 g/ml fractions. *Conclusion*. Our data highlight the differences of vesicle secretion by different cells and stress the need to evaluate separately sucrose gradient fractions within the previously defined 1.13–1.19 g/ml exosome density.

**Comparison of protocols for efficient isolation and characterisation of extracellular vesicles in human breast milk**

M.I. Zonneveld ^1^, E.N.M. Nolte-‘t Hoen^2^, F.A. Redegeld^1^, J. Garssen^3^ and M.H.M. Wauben^2^

^1^Division of Pharmacology, Department of Pharmaceutical Sciences, Faculty of Science, Utrecht University, Netherlands; ^2^Department of Biochemistry & Cell Biology, Faculty of Veterinary Medicine, Utrecht University, Netherlands; ^3^Danone Research Centre for Specialised Nutrition, Netherlands

*Introduction*: Extracellular vesicles (EV) with immune modulatory properties were identified in human breast milk. We hypothesise that EV in breast milk can instruct the immune system of infants. The exact composition and function of EV sub-populations in breast milk is unknown. Purification of EV from milk is difficult due to the complex nature of this body fluid. Furthermore, effects of milk storage on the EV population have not been addressed. We compared various approaches for efficient recovery of EV from (stored) human breast milk. *Methods*: Various modifications of differential centrifugation and sucrose density gradient ultracentrifugation protocols were applied to define efficient protocols for EV isolation from breast milk. Furthermore, effects of low temperature storage on the milk EV population were assessed. The presence of immune modulatory proteins and tetraspanins in defined milk EV populations was determined using western blot and multiplex immunoassays. *Results*: Loading of 10,000*g* milk supernatant on top of sucrose gradients resulted in more efficient and better quantitative EV isolation compared to flotation of 100,000*g* pelleted material up into overlaid sucrose gradients. We found that freezing unprocessed fresh milk resulted in a complete loss of milk cells, which led to a profound contamination of the milk EV population with material derived from dead cells. Freezing of cell-depleted 3,000*g* milk supernatant is now investigated as an alternative method to store milk for EV analysis. Analysis of milk EV by western blotting revealed the presence of several immunologically relevant proteins, such as MHC class II, FasL and MFG-E8. The presence of soluble immune modulatory molecules in milk EV, such as cytokines, is currently analysed using multiplex immunoassays. *Conclusions*: We compared and optimised protocols for efficient and quantitative milk EV isolation allowing reliable characterisation of immune modulatory properties of milk-derived EV.

**Differences in sub-populations of exosomes in human breast milk in relation to allergy and lifestyle, and allergic outcome of the child at the age of 2**

Patricia Torregrosa Paredes, C. Gutzeit, Q. Khaleda Rahman, S. Johansson, C. Admyre, J. Alm, A. Scheynius, and S. Gabrielsson

Karolinska Institutet, Sweden

*Introduction*: Breastfeeding has many beneficial effects on the developing immune system of the newborn. Breast milk contains immunoregulatory factors, such as nano-sized vesicles named exosomes. This study aimed at characterising breast milk exosomes from colostrum and mature milk and to investigate if allergic sensitisation as well as an anthroposophic lifestyle could influence the exosome profile in mature milk. *Material and methods*: Milk was collected from 20 mothers at days 3–6 and from 55 mothers at 2 months post-partum. Analyses were made concerning allergic status, anthroposophic lifestyle and the allergic outcome of the child at 2 years of age. Molecules on exosomes bound to beads coated with anti-MHC class II or anti-CD63 were analysed by flow cytometry. *Results*: While we found a decrease in exosomal MHC class II with time after partum, there was a trend towards increase in MHC class I expression. We show that human milk contains different sub-populations of exosomes and that allergic mothers have significantly lower levels of mucin-1 (MUC1) on exosomes within the sub-population selected for CD63 expression compared to healthy mothers. However, a lower level of MUC1 was found on MHC class II-selected exosomes from anthroposophic mothers compared to those following a non-anthroposophic lifestyle. Furthermore, mothers having babies who later developed allergy had an increased amount of MHC class I on their exosomes. We have also been able to detect and characterise exosomes in mouse milk. *Conclusions*: The content of exosomes in breast milk varies with the allergic status of the mother as well as her lifestyle, and this might influence allergy development in the child.

**A comparison of flow cytometry and nanoparticle tracking analysis to enumerate changes in plasma MV over time following traumatic injury**

Clara Yates ^1^, M. Foster^2^, S.P. Watson^2^ and G.B. Nash^2^

^1^National Institute for Health Research Surgical Reconstruction and Microbiology Research Centre, USA; ^2^National Institute for Health Research Surgical Reconstruction and Microbiology Research Centre, United Kingdom

*Introduction*: The concentration of circulating microvesicles (MV) detected by flow cytometry (FC) has been reported to increase following traumatic injury, in association with a hypercoagulable state (1). FC allows assessment of origin as well as number of MV, but typically does not detect the great majority of MV with a diameter <500 nm. Nanoparticle tracking analysis (NTA) allows quantification of MV with a diameter of 50–1000 nm. We characterised MV from patients with an injury severity score (ISS)>15, using both FC and NTA, to gain more complete information on changes with time following traumatic injury. *Material and methods*: Blood was collected from 19 trauma patients immediately post-injury and at time points up to 1 month post-injury. Plasma MV were collected from CPDA-coagulated blood by double centrifugation (2,000*g* 20 min; 13,000*g* 2 min) and stored at −80°C. MV from age- and sex-matched controls were also collected. MV >500 nm and those labelled with CD42 were counted by FC. Total plasma MV concentration and MV diameter were quantified using NTA (LM10; NanoSight, UK). *Results*: The total concentration of MV in normal plasma using FC was ~106/ml (with most being of platelet origin), but NTA detected ~1010/ml (with >95% having diameter <400 nm). Patients with trauma had higher numbers of MV, and this number tended to increase on average over the first month. *Conclusion*: NTA and FC detect different populations of MV and it is not clear which are linked to functional effects in coagulation or inflammation. Trauma is associated with changes in these populations, and follow-up on the functional effects of the MV in samples following trauma may elucidate how these populations are linked to evolution of thrombotic or inflammatory complications.

Reference

1. Park et al. Surgery. 151, 831–836.

**Effect of biofluid viscosity on size and sedimentation efficiency of microvesicles**

F. Momen-Heravi ^1^, L. Balaj^2^, S. Alian^1^, A.J. Trachtenberg^1^, F.H. Hochberg^2^, J. Skog^3^ and W.P. Kuo^1^

^1^Harvard Medical School, Boston, USA; ^2^Massachusetts General Hospital, Boston, USA; ^3^Exosome Diagnostics Inc, New York, USA

*Introduction*: The different chemical and molecular compositions of biofluids have an effect on its viscosity and this could affect movements of the particles inside the fluid. In this presentation we addressed the issue of whether viscosity has an effect on sedimentation efficiency of microvesicles (MVs) using ultracentrifugation. *Material and methods*: In this study we used biobanked plasma and serum as well as culture media (CM) from HEK-293T cells. As controls, polystyrene beads with the specific diameter of 100 nm were used to make control samples (plasma+beads, serum+beads, CM+beads, PBS+beads). We defined “pre-ultracentrifugation” (pre-UC) as aliquots of each sample prior to ultracentrifugation, used for quantity measurement of microvesicles/microparticles (MVs/MPs). After ultracentrifugation, pellets of samples were collected and re-suspended in 50μl PBS and now considered as “post-ultracentrifugation” (post-UC).The concentration and size of MVs/MPs for pre-UC samples and post-UC was identified by NanoSight LM10 system. Relative viscosities of pre-UC samples were measured using an Ostwald-type viscometer. *Results*: We noticed a significant difference between sedimentation efficiency of plasma, serum and culture media (p<0.001). The viscosity of the plasma, serum, CM and PBS were 1.65, 1.4, 1.1 and 1.0 cP, respectively. The Pearson correlation was −0.912 (p<0.001), indicating that a greater viscosity leads to lower sedimentation efficiency. The mean size of the MVs/MPs in both plasma and serum were found to be significantly larger in post-UC compared to pre-UC. Difference between the size of MVs in CM, less viscous biofluid, pre-UC and post-UC were insignificant. *Conclusions*: We demonstrate that MVs recovery inversely correlates with viscosity and as a result, sample dilutions should be considered prior to ultracentrifugation when processing any biofluids.

**Velocity gradient separates exosomes from HIV-1 particles**

Myriam Vaillancourt and C. Gilbert

Centre de recherche du CHU de Québec, Université Laval, Canada

Exosomes are endosome-derived particles of diameter up to approximately 100 nm. They are involved in intercellular communication and may contain miRNA, signalling and apoptotic proteins as well as viral proteins. Since their production and content vary as a result of pathological conditions, their involvement in immune homeostasis is considered likely. Increased production in response to human immunodeficiency virus (HIV-1) stimulation has been reported. HIV-1 is an enveloped retrovirus sharing several similarities with exosomes. To distinguish the specific roles of exosomes and HIV-1 in pathogenesis, it is crucial to obtain HIV-free exosome fractions and exosome-free HIV-1 fractions. The challenge is daunting because of the very similar size, density, protein and lipid composition. To characterise HIV-1-induced exosome release, Raji/CD4 cells were infected with NL4.3 virus or subjected to a mock infection treatment for 5 days. The filtered supernatant was centrifuged at 100,000*g*. The pellet containing HIV-1 virus, exosomes and possibly plasma membrane microvesicles was re-suspended in PBS and used to compare separation of HIV-1 virus from exosomes on sucrose density gradient (standard technique for exosome purification), iodixanol velocity gradient or ExoQuick™. Acetylcholinesterase activity, plasma membrane proteins and viral capsid protein p24 in each fraction of both gradients and in the ExoQuick pellet and supernatant were analysed using a spectrophotometer, western blot or ELISA. The results show that protein p24 was found in the fraction corresponding to exosomes in sucrose density gradient, but not in iodixanol velocity gradient, and that ExoQuick precipitated both HIV-1 and exosomes. We thus show that neither sucrose gradient nor ExoQuick discriminates between exosomes and HIV-1 particles. These results demonstrate that iodixanol velocity gradient remains the most effective method to separate HIV-1 from exosomes derived from culture supernatant.

**Qualitative and quantitative analysis of preservation techniques on extracellular vesicles**

A. Sorokina, J.M. Aliotta, M.S. Dooner, S. Wen, L.R. Goldberg, D.A. Adler, M. DelTatto, E. Papa and P.J. Quesenberry

Rhode Island Hospital, Warren Alpert Medical School of Brown University, Department of Medicine, USA

*Introduction*: Extracellular vesicles (EVs) are capable of altering cell phenotype and whole cell population fate (Ratajczak et al., 2006). Camussi et al. (2008) demonstrated that mesenchymal EV administration, during kidney injury, stimulates tubular cell regeneration and accelerates morphological and functional recovery. These studies suggest novel uses of EV in both treatment and prevention of disease. In order to facilitate the use of EV for clinical application, it is crucial to develop efficient methods for their long-term storage without compromising their function. *Material and methods*: EVs from lung were stored in PBS (1% DMSO) at 4 and −20°C for up to 7 days. Quantitative analysis was performed on the EVs pre- and post- preservation (NanoSight, BCA protein assay). In order to establish functional conservation, murine whole bone marrow (WBM) cells were co-cultured with fresh and preserved EVs for 2 weeks. Expression of pulmonary epithelial cell mRNA by co-cultured WBM was measured by RT-PCR. *Results*: Preservation at 4 and −20°C had no quantitative effect on EVs. No statistically significant difference of WBM cell expression of the pulmonary epithelial cell genes surfactants B, D and clara cell-specific protein was observed after co-culture with EVs. Additionally, fresh and preserved EVs did not impact the viability of WBM cells in co-culture. *Conclusion*: Murine lung-derived EVs can be preserved at −20°C without quantitative or functional compromise.

**Do stimulated erythrocyte microvesicles commonly produced for research purposes have the same biological structure and function as those releases naturally?**

S. Harry, U.L.P. Fairbrother and S.M.B. Heugh

London Metropolitan University, United Kingdom

*Introduction*: Erythrocyte-derived microvesicles (eMVs) are small vesicles released from the plasma membrane of the mature erythrocyte, and they play a significant role in health and disease. Naturally occurring entities, resulting from biochemical and morphological changes to the plasma membrane (e.g. intracellular Ca2+, oxidative stress) encountered during microcirculation affecting erythrocyte structure and stability, results in eMV release. Current research in this area often evolves from induced (i) eMVs; this study investigates structural and functional features of induced i-eMVs vs. constituent (c)eMVs. *Materials and methods*: eMVs were generated from erythrocytes incubated with and without normal human serum (NHS) extracted by high speed centrifugation, size and number was measured by qNano and guava easyCyte^TM^ flow cytometry and protein concentration of each was determined. eMVs’ interactions with THP-I monocytic leukaemia cells were visualised using LumaScope 500. *Results*: Evaluation of the eMVs produced revealed two distinct populations between c-eMVs (released naturally) and i-eMVs. c-eMVs are smaller in size (8–900 nm) and released in lower numbers (0.01/erythrocyte), whereas greater numbers (0.057/erythrocyte) are produced from those stimulated with NHS and are larger with a typical size (9–1,100 nm). Protein content is lower in c-eMVs, than i-eMVs (30% less). Biological interactions with THP-1 cells revealed contact and uptake of non-induced c-eMVs but interactions were not observed with those induced with NHS. *Conclusion*: This study demonstrates a significant difference in size and protein content between the two populations of eMVs and altered biological activity in relation to phagocytosis by TPH-1 cells; this preliminary work indicates that NHS-induced eMVs will not make good experimental models. As work into the role of eMVs in cellular communication is an area yet to be investigated, it may be prudent to focus on naturally released eMVs to mimic in vivo actions.

**Characterisation of exosomes secreted from senescent fibroblasts**

M. Okada, A. Nakamura, M. Muneoka and H. Tahara

Hiroshima University, Japan

Exosomes are lipid membrane vesicles secreted by cells known to act as a tool for cell to cell communication. They participate in a variety of biological function in our bodies. It is known that the quality and the quantity of exosomes are changed during human aging and diseases. Therefore the characterisation of exosome is very important not only for the application of disease diagnosis and treatment, but also the breakthrough in mechanisms of aging and disease. We examined the exosomes derived from senescent cells to reveal the changes of exosomes during the aging process. First, we used the replicative senescence model of human normal fibroblast to clarify the characteristics of exosomes in cellular senescence. We isolated exosomes from human fibroblast, TIG-3 cells, and measured the number and the size of exosomes using qNano. The amount of their total protein and membrane-associated protein of exosomes were measured by using Micro BCA assay. The results indicated that replicative senescent cells secreted much more amount of exosomes than young cells. To elucidate the mechanism of exosome production in senescent cells, we performed functional analysis using siRNAs-targeted exosome-regulatory genes and found that one of the genes regulates exosome secretion in senescence. Furthermore, we try to reveal the function of exosomes derived from senescent cells. For example, co-culture of young cells with senescent cells inhibited exosomes secretion, and we added the exosomes derived from senescent cells to young cells. In this presentation, we would like to discuss about the technique to characterise the exosome from cultured media, especially focusing on the purification and the counting of exosomes.

**Phenotyping of various membrane markers on plasma exosomes from 80 healthy donors using protein microarray**

Rikke Bæk, M. Jørgensen, E.K.L. Søndergaard and K. Varming

Department of Clinical Immunology, Aalborg University Hospital, Denmark

*Introduction*: Exosomes are endosome-derived vesicles between 40 and 100 nm in diameter that are secreted by many cell types. The quantity and molecular composition of exosomes shed from various cell types differ considerably. It is therefore expected that plasma from healthy donors will contain a wide range of exosomes with different phenotypes, reflecting the phenotype of the cells that produced them. *Materials and methods*: In this study the novel multiplexed platform of protein microarray is used for capturing, detecting and profiling exosomes in plasma from 80 healthy donors. The assay is based on the antibody capture of microvesicles and subsequent detection of the captured microvesicles by biotin-labelled anti-tetraspanin antibodies (CD9, CD63 and CD81). Antibodies against 21 different exosome biomarkers were used to capture the exosomes. The panel of antibodies contained the well-known exosome markers (CD9, CD63, CD81 and HLA-ABC) and 17 other membrane markers and antigens related to, for example, cancer and inflammation. *Results and conclusion*: Using protein microarray it was possible to detect and profile exosomes for 21 analytes simultaneously using only 10 µl of plasma. In the cohort of 80 healthy donors, the amount of exosomes varied from 5×108 to 1.5×109 exosomes/ml plasma. The distribution of the 21 exosome markers varied greatly among the donors and was visualised using clustering analysis. The clustering clearly demonstrates that donors with a low amount of exosomes expresses exosomes with a higher percentage of the normal exosome markers (CD9 and CD81). This is in relation to donors with a high amount of exosomes that seem to have exosomes with a broader range of markers.

**Active sorting of membrane proteins on exosomes: methods for label-free analysis in drug discovery and diagnostic**

M. Lotierzo ^1^, C. Leveque^2^, M. Prorok-Hamon^1^, R. Burrer^1^ and R. Mamoun^1^

^1^CILOA, France; ^2^Université d'Aix-Marseille, France

*Introduction*: Ciloa innovative technology provides an efficient way to characterise membrane proteins and their ligands in native context. Membrane proteins play a crucial role in many cellular and physiological processes, mediating the transfer of information between cells and their environment. However, transmembrane proteins are often fully functional only when anchored in a membrane hydrophobic environment. *Materials and methods*: Ciloa technology to target membrane proteins on exosomes is combined to perform ant analysis systems. In particular, surface plasmon resonance (SPR) using label-free methods and microfluidic circuits provides a powerful means to monitor interactions with protein-based biochip. Several kinds of membrane proteins, from GPCR to ion channels are presented by recombinant exosomes in a high number of copy. Ciloa technology takes advantage of a patented pilot peptide fused to proteins of interest to address and enrich exosomes with target proteins. This is a unique tool because these membrane proteins are now easy to manipulate, can be produced in large batches and used exactly like a simple hydrophilic protein solution. Exosomes-based ELISA, FACS or SPR biosensors were applied, to analyse the well-folding and conformation of membrane proteins. *Results*: Results showed that natural ligands as well as specific antibodies or small molecules interact specifically with the membrane proteins harboured by exosomes; in particular, monoclonal antibodies are able to recognise non-linear epitopes in the extracellular domain(s) of proteins. Kinetics and affinity parameters of different ligands are calculated. *Conclusions*: Combination of recombinant protein exosomes and label-free analysis methods allow using disruptive technologies for interaction studies on pharmaceutical target proteins, leading to powerful applications in drug discovery and diagnostic.

**Characterisation of exosomes secreted from in vitro neuronal cell lines**

J. Li ^1^, Y. Lee^1^, P. Vader^1^, S. El Andaloussi^1^, I. Mager^1^, C. Gardiner^2^, I. Sargent^2^ and M.J. Wood^1^

^1^Department of Physiology, Anatomy and Genetics, University of Oxford, United Kingdom; ^2^Nuffield Department of Obstetrics and Gynaecology, University of Oxford, United Kingdom

*Introduction*: Exosomes from bone marrow dendritic cells (BMDC) have been shown to be successful delivery vehicles for targeted siRNA delivery to the brain. The major challenge of using BMDC to produce exosomes is that the primary culture does not reproducibly generate a large amount of exosomes for clinical practice. The preparation of the cells is quite difficult and time-consuming. One alternative parental cell type which may potentially be more applicable is neuronal cells. Here we report an in vitro study of neuronal cell lines to assess their potential as sources of exosomes for nervous system drug delivery. *Material and methods*: Using NSC-34 and N2a cell lines, we studied exosome production under 3 different seeding ratios (low, medium and high) growing in 2 types of media (DMEM supplemented with exosome-free FCS and Opti-MEM) and harvested across 5 days. We used western blot to semi-quantify exosome production by probing for exosomal markers and nanoparticle tracking analysis (NTA) to measure the size and concentration of particles isolated from these cell lines. *Results*: Our results showed a population of exosomes produced from these cells as indicated by clear flotillin and CD9 bands on western blots. Exosomes harvested at day 4 showed the strongest bands compared to the exosomes harvested at other days, which might indicate that exosomes are produced before or along cell death. In Opti-MEM, cells produced more particles than in DMEM. *Conclusions*: We have characterised the exosomes from NSC-34 and N2a cell lines and believe that they have the potential as sources of exosomes and can be used conveniently as neuronal sources of exosomes. These cell lines can be used to carry out in vitro studies to understand exosomal biology and might be used for future studies on neurological disease applications. However, caution must be taken for in vivo application as the exosomes from these cell lines may have neuroblastoma features.

**Serum-derived exosomes evaluated early in the second trimester provide predictive biomarkers of risk for spontaneous preterm birth**

A.M. Ezrin ^1^, B. D. Brohman^1^, J. Willmot^1^, S. Baxter^2^, K. Moore^2^, M. Luther^2^, M. Fannon^3^ and B. Sibai^4^

^1^NxPharmaGen Inc., USA; ^2^David H. Murdock Research Institute, USA; ^3^BioIT Solutions Inc, USA; ^4^UT Health – Houston Division of Maternal Fetal Medicine, USA

*Introduction*: The purpose of this study was to characterise proteomic differences found in circulating serum exosomes during the early second trimester of pregnancy as potential biomarkers of spontaneous preterm birth. Exosomes shed from the syncytiotrophoblast freely circulate in high titre and reflect the maternal–foetal interface status. Subtle homeostatic changes can be reflected in the identification of a unique set of dysregulated proteins, which may serve as early risk predictors of preterm birth. *Materials and methods*: Frozen serum samples (n=48) were obtained from a biorepository from asymptomatic healthy women at 15–17 weeks of gestation whose pregnancies resulted in a live birth. Exclusion criteria included multiple births, women who had IVF/ICSI or who have a pre-existing medical condition(s) and those with known foetal anomalies. Serum exosomes were isolated and proteins analysed using an open proteomics LC-MS platform. Peptide features were differentially analysed in a blinded manner from patients delivering >37 weeks (n=24) and patients delivering <34 weeks (n=24). *Results*: A unique protein expression pattern was observed amongst 98 proteins that were identified as potential biomarkers. Eighteen proteins differentiated between the preterm and term populations (12 preterm, 6 term) that were observed in both first and second pregnancies, with a minimum of 2 significant peptides and reproduced by two independent statistical interrogations. The protein biomarkers identifying preterm outcomes at 15–17 weeks’ gestation were confirmed based on peptide scoring in an independent laboratory and map to inflammatory and cell injury pathways that differ from the term profile. *Conclusion*: A novel library of statistically valued exosomal biomarkers has been identified with potential clinical utility in defining women at risk for preterm birth. These markers are being further evaluated for clinical use and validation in a small-plex assay.

**Fluorescence activated vesicle sorting: a novel directed approach to subset individual exosomes**

James Higginbotham, M. Demory Beckler, J.L. Franklin, and R.J. Coffey

Vanderbilt University Medical Center, USA

*Introduction*: Previously, we showed that extracellular vesicles (ECVs) containing EGF receptor (EGFR) ligands or mutant KRAS promote features of cancer progression in vitro, including invasion and growth in 3D (1). The average diameter and proteins contained in these ECVs are consistent with exosomes. During the course of these studies, we developed fluorescence-activated vesicle sorting (FAVS), a technique that provides accurate and quantitative characterisation of individual exosomes. By FAVS, we detected the EGFR ligand amphiregulin (AREG) in exosomes from MDCK cells stably over-expressing AREG and found an average of 24 molecules of AREG per vesicle by quantitative confocal microscopy. To further extend the utility of FAVS, we now demonstrate that using a multicolour-based FAVS approach we can efficiently subset DLD-1 cell-derived exosomes based on their levels of different endogenous EGFR ligands. *Materials and methods*: A BD FACSAria III upgraded with a forward scatter PMT was utilised for all FAVS analysis. DLD-1 cell exosomes were used as they express moderate levels of TGF-alpha (TGF-a), HBEGF and AREG. All additional reagents used were used as outlined (1). *Results*: FAVS analysis of DLD-1-derived exosomes stained individually for AREG, HBEGF and TGF-a resulted in 84.3%, 58.5 and 42% staining, respectively. Multicolour analysis utilising FAVS of DLD1 exosomes further revealed that a minor subset of exosomes containing all three ligands was limited to 28.5%. We are currently developing a 4-colour panel to determine the levels of these ligands and EGFR on exosomes purified from human plasma. *Conclusions*: FAVS offers a novel platform to perform sensitive and quantitative measurements of exosome composition. A distinct advantage of FAVS is that it allows subsetting of heterogeneous populations of exosomes and the potential purification of those populations.

Reference

1. Higginbotham J, Demory Beckler M, Franklin JL, Coffey RJ. Curr Biol. 2011;21:779–86.

**Standardisation of collection, handling and detection of extracellular vesicles**

Y. Yuana, A.N. Böing, C.M. Hau, A.E. Grootemaat, A. Sturk and R. Nieuwland

Department of Clinical Chemistry, Academic Medical Centre, University of Amsterdam, Netherlands

*Introduction*: Biological fluids contain high numbers of extracellular vesicles (EV). The presence and composition of EV depend on disease state and on collection, handling, storage and isolation procedures. The most commonly used method to detect EV is flow cytometry (FCM), which detects only 1–2% of all EV present. Due to detection problems, results from EV research are difficult to compare between laboratories. Thus, we aim to develop standard collection and handling protocols and to perform sensitive detection of EV using suitable techniques such as resistive pulse sensing (RPS) and nanoparticle tracking analysis (NTA). *Materials and methods*: EV were prepared from erythrocytes, platelets and from cell lines of ovarian origin, prostate origin and mammary epithelial cells. EV were isolated by using two centrifugation protocols, in which the speed of centrifugation (18,890*g* and 100,000*g*) and centrifugation time (0.5–2 h) were varied. EV were reconstituted in either PBS or vesicle-depleted human plasma. EV preparations were measured directly or snap-frozen in liquid N_2_ and stored at −80°C for further analysis. *Results*: In comparison to FCM, RPS and NTA detect 1,000-10,000-fold more particles in all EV preparations. Generally, the concentration and particle size of EV are more affected by the single freeze/thaw cycle than by centrifugation conditions. For example, freshly prepared EV samples from erythrocytes measured by RPS and NTA contain 2×10^10^ particles/mL with typical diameter of 200 nm, regardless of the applied centrifugation conditions. After thawing, EV reconstituted in PBS contain less particles (9×10^9^ particles/mL) with slightly smaller diameter (160–180 nm), whereas those reconstituted in vesicle-depleted plasma are comparable to the freshly prepared EV. *Conclusions*: Type of EV, reconstitution solution and detection limit of techniques used to measure EV are important factors to standardise protocols.

**The biological effect of erythrocytes-derived microvesicles on the growth of Jurkat, THP-1, PC3-M and MCF-7 cell lines. Does this biological function indicate potential therapeutic roles?**

R. Freezor, A. Haidery and S.M.B. Heugh

London Metropolitan University, United Kingdom

*Introduction*: Erythrocyte microvesicles (eMVs) are a heterogeneous population of small plasma-derived vesicles that have been reported as being smaller than other microvesicles subtypes (0.1–0.4 µm). Research indicates elevated number of eMVs in stored blood and several disease states (e.g. sickle cell anaemia, thalassemia and idiopathic thrombocytopenic purpura). This work forms part of an ongoing study designed to provide robust evidence of biological interactions of eMV with different cell lines to determine intracellular communication systems. *Materials and method*: Erythrocytes were isolated from fresh blood by centrifugation; the pellet was incubated (30 min, 37°C) with RPMI 40 and CaCl_2_. eMVs were extracted by high-speed centrifugation, then confirmed and enumerated using guava easyCyte^TM^ flow cytometry. eMVs were incubated with Jurkat, THP-1, PC-3M or MCF-7 (all 95% viable). eMVs and cell line interactions were visualised using LumaScope 500. *Results*: Significant inhibition of growth rates of Jurkat and THP-1 cells was observed from 24 h, but no major effect on cell viability. eMVs effects on Jurkat cell line growth was the most pronounced vs. untreated cells. Time-lapse alternating bright field and fluorescence microscopy photography produced images of eMVs aggregating, moving towards cells, interacting with the membrane, eMV Annexin V-FITC-labelled material was observed inside THP-1 and Jurkat cells, but no interaction with PC3-M and MCF-7. *Discussion*: Initial results reveal direct microvesicle interaction and effect on Jurkat and TPH-1, but not with PC3-M and MCF-7 cells in vitro. Annexin-V blocking indicates that phosphatidylserine (PS) receptors are not the site of entry into the cells; therefore the observed inhibition of growth (Jurkat, and THP-1) cells is not due to immunosuppression or initiation of apoptosis associated with PS. eMV interaction with Jurkat (tALL) and THP-1 (AML) model cells shows potential as a novel therapeutic delivery vector.

**Cancer cell expulsion of anticancer drugs through shedding of microvesicles: association with drug resistance and tumour survival**

Jameel Inal ^1^ and S. Jorfi^2^

^1^London Metropolitan University, United Kingdom; ^2^London Metropolitan University, Cellular and Molecular Immunology Research Centre, United Kingdom

*Introduction*: Microvesicles (MVs) are small (0.1–≤1 µm in diameter), heterogeneous vesicles released from cells constitutively or upon activation, that mediate intercellular communication. Multidrug resistance has been defined as the ability of cancer cells to survive after treatment with various drugs. However, the mechanism(s) used by cancer cells to evade apoptosis induced by anticancer drugs remain unclear and was the subject of our investigation. *Materials and methods*: Cells (5×104/well) were seeded into 12-well plates in triplicate and were treated with various concentrations of 5-fluorouracil (5-FU) or methotrexate (MTX). Cells were washed 30 min after treatment with 5-FU and MTX and related condition medium collected to isolate MVs. Cells were washed and resuspended in complete growth medium (GM) and incubated at 37°C with 5% CO_2_. Cell viability was determined on a guava flow cytometer with viacount assay every day for 72 h. *Results*: Here we report a mechanism of cancer cell expulsion of anticancer drugs through the release of MVs. In addition, we show for the first time that inhibition of MV release by pretreatment of PC3M cells with the calpain inhibitor, calpeptin, sensitises cancer cells to drug-elicited apoptosis mediated by the addition of methotrexate and docetoxel (DOC) using at least 10-fold lower concentrations, both in vitro and in vivo. Treatment of cancer patients with MET or DOC leads to significant side effects due to the use of higher doses. Here we show that these drugs when administered together with calpeptin can be given at doses 100 times lower and still induce effective killing of target cancer cells. *Conclusions*: Overall our studies shed light on the role of MV release in cancer cell expulsion of anticancer drugs and subsequent evasion and survival from apoptosis and suggest new combination therapies for existing cancer drugs.

**Extracellular vesicles: using their own weapons against them**

J.Y. Hur ^1^, D.Y. Choi^2^, H.J. Kim^3^, K.Y. Lee^3^ and K.P. Kim^2^

^1^Seoul National University, Konkuk University, Republic of Korea; ^2^Konkuk University, Republic of Korea; ^3^Konkuk University School of Medicine, Republic of Korea

*Introduction*: Cancer cells secrete extracellular vesicles (EVs) – including exosomes, apoptotic bodies and microvesicles – to extracellular environment. Cancer-derived EVs have various proteins, mRNAs, microRNAs and DNAs that can deliver their information to recipient cells, and they are present in body fluids such as amniotic fluid, milk, saliva, urine and blood. EVs are very important suborganelles related to the process of cancer immune suppression, metastasis and angiogenesis. Therefore, the study of cancer-derived EVs is useful for finding cancer biomarkers and therapy. *Material and methods*: We treated EVs of Gefitinib-resistant PC9 human lung cancer cells (PC9/GR) to Gefitinib-sensitive PC9 cells, and then treated Gefitinib to PC9 cells. Gefitinib, an inhibitor of epidermal growth factor receptor (EGFR) tyrosine kinase, has been shown to suppress the activation of EGFR signalling for survival in non-small cell lung cancer (NSCLC) cell lines. Vesicular proteins from PC9 and PC9/GR were analysed using mass spectrometry (MS) and vesicular DNA mutation was determined using PNA-mediated real-time PCR. *Results*: Gefitinib-sensitive PC9 cells obtained drug resistance from extracellular vesicles. EVs of PC9/GR cells had abundant Caveolar-mediated endocytosis, integrin and PI3K-AKT signalling proteins compared to EVs of PC9 cells. Cancer-derived EVs had mutant DNA. These factors together gave drug resistance to sensitive cells. *Conclusions*: We found that EVs transfer drug resistance to a drug-sensitive cancer cell. We will analyse how drug-resistant cancer-derived EVs affect sensitive cells to have drug resistance. EVs have their own unique characteristic of biomolecules such as proteins, mRNAs, microRNAs and DNAs. Thus cancer-derived EVs have potential as diagnostics and medical treatment for cancer. Also, cancer cells use EVs to efficiently resist current treatments for cancer, but if we can use EVs against the cancer cell it would increase efficacy of cancer treatments.

**Intracellular trafficking and integration of P-GP IN MCF-7 cells following microparticle transfer**

P.V. Dalla^1^, F. Luk ^1^, L. Turnbull^2^, C. Whitchurch^2^, G.E.R. Grau^3^ and M. Bebawy^1^

^1^School of Pharmacy, University of Technology, Sydney, Australia; ^2^The iThree Institute, University of Technology, Sydney, Australia; ^3^Vascular Immunology Unit, Sydney Medical School and Bosch Institute, The University of Sydney, Australia

*Introduction*: Multidrug resistance (MDR) in cancer is a multifactorial problem which results in the failure of tumour's response to chemotherapeutics. The over-expression of cell surface efflux transporters, including P-glycoprotein (P-gp), is shown to contribute towards MDR. Microparticles (MPs) are small (0.1–>1 µm) vesicles which bud from the cell surface of malignant cells. MPs originating from MDR cells carry a selection of pathologically significant proteins including P-gp, as well as nucleic acids and other cargo. Our team has established that in leukaemia, the MPs shed from drug-resistant cells transfer functional P-gp to their drug-sensitive counterpart upon exposure. *Materials and methods*: MPs were isolated from drug-resistant MCF-7 breast cancer cells over-expressing P-gp and green fluorescent fusion proteins (P-gp–GFP) and visualised by super-resolution microscopy. The isolated MPs were cultured with their drug-sensitive counterpart, MCF-7 cells, over a time course and analysed using wide field microscopy. *Results*: The MCF-7–P-gp-–GFP isolated MPs have a diameter of 1.2×1.28 µm. A limited amount of MP docking is observed on the surface membrane of the MCF-7 cell at 30-min co-culture. At 2 h, the MP docking is more prominent and minor quantities of MP cargo are trafficked to intracellular organelles. The MP cargo including P-gp is abundant within the cell in a diffuse pattern after 4 h of co-culture and at 8 h this pattern has become punctuate, concentrating within cells within the peri-nuclear region. *Conclusions*: Our results are consistent with our previous studies using flow cytometry, demonstrating the transfer of P-gp from drug-resistant cells to drug-sensitive cells. Future studies will include charting the intercellular pathway of the MP cargo and characterising whether the intercellular uptake of P-gp via this method can contribute to the overall MDR trait.

Funding: This work was supported by the National Health and Medical Research Council: APP1007613

**Intercellular transfer of functional MRP1 and the re-templating of intrinsic resistance pathways via microparticles**

J. Lu ^1^, R. Jaiswal^2^, F. Luk^1^, G.E.R. Grau^2^, and M. Bebawy^1^

^1^University of Technology, Sydney, Australia; ^2^University of Sydney, Australia

*Introduction*: Multidrug resistance (MDR) is a major obstacle to the success of chemotherapy in clinical oncology. The ATP-dependent transmembrane proteins, P-glycoprotein (P-gp, ABCB1) and multidrug resistance-associated protein 1 (MRP1, ABCC1) are primary contributors to MDR in cancer. These proteins maintain a sublethal concentration of intracellular chemotherapeutics by virtue of its active efflux capacity. This study reports the acquisition and dissemination of functional MRP1 in drug-sensitive cells via microparticle (MP)-mediated intercellular transfer. We also demonstrate that MP donor cell trait can dominate and re-template recipient cells which possess an alternative dominant resistance mechanism. *Materials and methods*: MPs are co-cultured with drug sensitive cells. Western blot was used to confirm the expression of MRP1 in whole cells and isolated MPs. To assess the functionality of transferred MRP1, we used probenecid (MRP1 inhibitor) and Calcein-AM drug accumulation assays on flow cytometric analysis. The presence of ABCC1 and ABCB1 mRNA, in isolated MPs and in whole cells was assessed using qRT-PCR. *Results*: We show the transfer and time-dependant functionality of MRP1 in drug-sensitive leukaemia cells which are exposed to MPs shed by MRP1-overexpressing cells. MPs shed from cells with P-gp dominant resistance profile were found to re-template a pre-existing MRP1 dominant profile in haematological or non-haematological recipient cells. *Conclusions*: Our findings demonstrate a potent capacity of MPs to alter MDR traits and transcriptional environments to which they are exposed to. This work is likely to have significant potential for the translation into alternative oncological treatment strategies and circumvention of MDR. Furthermore, these findings have significance in understanding the molecular basis for tumour overexpressing phenotypes and reiterates MPs as potential clinical targets to prevent the spread of MDR in cancer.

**Poster Session I (Arlington-Berkeley): Cancer Protein Biomarkers April 17**

**Chair:** *J. Leonard and J. Rak*

**Expression profiles of common exosomal markers in human plasma can distinguish tumour types and stages**

P. Guazzi^1^, J. Muhhina^1^, T. Oja^1^, E. Lari^2^, A. Chiesi^1^ and N. Zarovni ^1^

^1^Hansabiomed OU, Estonia; ^2^Exosomics Siena SpA, Italy

*Introduction*: Exosomes carry a range of membrane and cytosolic proteins comprising endosomal compartment and transport/fusion proteins, and specific proteins indicative of cell type and functional state. Though former ones are ubiquitously found on exosomes from different biological samples and referred to as common identification markers, their expression can vary across exosomes from different sources. We have addressed the variation in expression of some common exosomal proteins (CD9, CD81, CD63, Rab5, HSP70, TSTA3) and tumour-specific proteins (i.e. TM9SF4) in plasma exosomes from patients with different solid tumours. Protein levels detected by ExoTESTTM were correlated to actual vesicle number and protein content measured by NTA and IR spectrometry. *Material and methods*: ExoTESTTM was used to screen exosomal markers in five solid tumours vs. healthy individuals. Exosomes were captured on ExoTESTTM plate by incubation with precleared plasma samples. Different capture/detection antibodies as well as NTA analysis (LM10HS NanoSight) were used to define the correspondence between expression profiles and number of exosomes in each sample. *Results*: ELISA analysis of human plasma exosomes revealed different and somewhat unexpected expression variations in common exosomal markers during cancer progression and across different tumour types. In particular CD63 expression changed with tumour stage in a way that did not correspond to the overall number of circulating exosomes. Potential of distinct exosomal markers for accurate overall quantification and identification of tumour-related exosomes is reported. *Conclusions*: When used in a quantitative immunoassay, some commonly acknowledged exosomal proteins can reveal as specific markers of tumour type and stage due to variations in overall exosome number or altered protein levels. ExoTESTTM is a versatile platform for revealing informative biomarker profiles in research and diagnostic applications.

Funded by: EAS EU29269 and EU31369

**Multicolour flow cytometric analysis of cancer-derived microvesicles reveals a unique subpopulation ratio in plasma from prostate cancer patients**

J. Schettini, M. Maheshwari, K. Desai, B. Rhees and D. Spetzler

Caris Life Sciences, USA

Circulating microvesicles (cMV) are cell-derived vesicles that can be isolated from many biofluids and culture media. Previous studies have shown that cMV are released by several cell types including immunocytes, endothelial, embryonic, tumour cells and also platelets. cMV in blood are a source of potential biomarkers of disease diagnosis and progression. The purpose of this study was to determine whether exposed biomarkers on the surface of cMV from processed plasma could distinguish prostate cancer microvesicles from atypia, high-grade prostatic intraepithelial neoplasia (HGPIN), benign or prostate inflammation. Isolated cMV from positive biopsy cancer patient blood were stained with a panel of specific conjugated antibodies to compare phenotype, frequency and marker expression. Samples were collected prospectively prior to biopsy. The distribution of the cohort included 80 men with previously undiagnosed prostate cancer, 13 men with previously diagnosed prostate cancer (active surveillance), 6 atypia, 23 HGPIN, 28 inflammation, 49 benign and 25 normal samples. The cMV from these patients were analysed by multicolour flow cytometry. Subpopulations of cMV were determined based on multiple combination of marker expression through proper gating. Our results showed that single marker expression on cMV were not enough to establish a protein expression pattern among samples. However, a systematic review of all possible combinations of markers showed a triple positive subpopulation ratio of cMV significantly augmented in prostate cancer samples (current biopsy) and HGPIN over other conditions. These results demonstrate that isolated cMV from plasma can be used to determine the specific subpopulation relevant in prostate cancer diagnosis.

**Prostate cancer microparticles for follow-up after prostatectomy**

Hon Leong ^1^, C. Biggs^1^, A. Al-zahrani^1^, V. Yutkin^1^, J. Chin^2^ and J.D. Lewis^3^

^1^Translational Prostate Cancer Research Group, London Health Sciences Centre, Canada; ^2^Division of Urology, Department of Surgery, University of Western Ontario, Canada; ^3^University of Alberta, Canada

*Introduction*: Radical prostatectomy (RP) is a viable option for treatment of localised prostate cancer (PCa). The presence of plasma-borne prostate tumour cell fragments (microparticles/exosomes/oncosomes) is thought to correlate with the magnitude of the tumour burden. The primary objective of this study is to assess wither PCa microparticle (PCMPs) can help in the follow-up of patients after RP. *Material and methods*: This is a prospective study to measure PCMP counts before and 3 weeks after RP. Ethic Review Board of the institution approved this trial. To identify the PCMP population by nanoscale flow cytometry (A50-Micro, Apogee Flow Systems Inc.), fluorophore-conjugated antibodies specific for the extracellular domain of PSMA (anti-PSMA mouse IgG-RPE) and the metastasis-specific 1A5 antibody (1A5 mouse IgG-FITC) were used to stain 20 µL of plasma. PCMPs were defined as exhibiting a diameter range between 12 and 880 nm and bonded both PSMA and 1A5 mAbs. We compared the total number of PSMA-positive events, 1A5+PSMA-positive events and percentage of 1A5+PSMA/total PSMA-positive events before and 3 weeks after RP. *Results*: In all, 25 patients were recruited for this study. Twenty-two patients had their blood tested for PCMPs before and after RP. The clinico-pathological features of these patients are summarised in Table 1. Sixteen patients (76.1%) showed decline in the level of PCMPs after RP. The level of PCMPs did not change or increase in 6 patients (23%). Clinical follow-up to determine if these PCa patients are at risk for persistence or recurrence of their disease is currently underway. There was no correlation between the PCMPs change status and clinico-pathological characteristic. *Conclusions*: Enumeration of prostate cancer microparticles may provide a clinical means to follow patients after surgical intervention. Larger cohort analysis and longer follow-up times are needed to confirm these findings.

**Towards biobanking of microvesicles: microvesicular research of patient samples in Helsinki urological biobank**

Maija Puhka ^1^, T. Hällström^1^, K. Pitkänen^1^, A. Rannikko^2^ and O. Kallioniemi^1^

^1^Institute for Molecular Medicine Finland FIMM, Finland; ^2^Hospital District of Helsinki and Uusimaa, Finland

*Introduction*: Helsinki urological biobank is a joint project of Hospital district of Helsinki and Uusimaa and Institute for Molecular Medicine, Finland. Based on the informed consent and by first ensuring the needs of diagnostics and treatment, Helsinki urological biobank collects tissue, blood and urine samples from patients with bladder, testicle, kidney and prostate cancer or benign prostatic hyperplasia, coupled with comprehensive clinical information. We aim to set up extraction protocols of microvesicles from the biobank samples starting from urine in order to serve the needs of biobank users and our own research projects on prostate cancer. *Materials and methods*: Microvesicles were isolated using microfiltration and precipitation reagents. Isolated microvesicles were validated and characterised by western blotting and other commonly used strategies. *Results*: We show that microvesicles from patient samples contain several exosomal markers and some markers characteristic of the disease. We find no or only minor amounts of contaminating molecules such as organelle markers indicating a reasonable purity of the microvesicle sample. *Conclusions*: Patient material stored in the biobank is well preserved and amenable for microvesicle extraction. Microvesicles obtained with the methods used here are suitable for further research.

**Isolation and molecular characterisation of exosomes derived from breast cancer patients and individuals with benign breast disease**

S. Ceder and T. Panaretakis

Karolinska Institutet, Sweden

*Introduction*: Despite the improvement of standard therapy against breast cancer (BrCa), it remains one of the leading causes of cancer-related deaths in women. It is well-established that early, pre-metastatic detection of cancer lesions increases exponentially the efficacy of cancer therapy leading to the eradication of the disease. Despite intense investigations, few novel biomarkers have been identified and introduced in clinical BrCa diagnostics. Thus, there is an urgent and unmet need for the discovery of biomarkers that will predict accurately and reproducibly the development of cancer, allow for early diagnosis, predict metastatic potential and response to therapy. Exosomes are small bioactive vesicles that function as a vehicle for cell-free intercellular communication. Importantly, exosomes isolated from biological fluids (e.g. blood or urine) and malignant effusions (e.g. ascites) contain antigen specific to the tumour. *Materials and methods*: In the present study we have isolated and analysed exosomes from samples collected for the Karma study, one of the world's best characterised BrCa cohorts located in Sweden. Comparative proteomic analysis was performed on exosomes isolated from plasma of healthy individuals, individuals with benign breast diseases and breast cancer patients. *Results*: Our results show that there was a dramatic increase both in the protein concentration and level of circulating exosomes in patients with breast cancer. The data obtained indicate significant differences in the molecular composition of the exosomes isolated from these three groups and have led to the identification of several putative novel biomarkers that will need to be validated in the clinic. *Conclusions*: Overall, identifying breast cancer-specific markers on exosomes isolated from patient plasma offer a novel, non-invasive diagnostic tool for the early detection of breast cancer.

**Cancer stem cells markers profiling of colon cancer exosomes**

Elena Khomyakova ^1^, M. Loguinova^1^, V. Lazarev^1^, D. Klinov^1^, S. Chernyshov^2^, E. Generozov^1^ and V. Govorun^1^

^1^Research Institute of Physico-Chemical Medicine, Russian Federation; ^2^State Research Center of Coloproctology, Russian Federation

*Introduction*: Colorectal cancer, the third most common cancer, remains a serious health concern. Most of colon cancer deaths result from the metastatic propagation of drug-resistant cells. According to the stem cell concept of carcinogenesis, ineffectiveness of standard therapies has been attributed to subpopulation of self-renewing, drug-resistant cells: cancer stem cells (CSCs). CSCs are responsible for tumour initiation, metastasis formation and treatment failure. Tumour-derived exosomes could be potentially considered as carriers of CSC markers and therefore used for tumour characterisation. The present study is aimed to analyse the expression of colon CSC markers CD44, CD166 and markers CD66 and A33 on HT29 and CaCo-2 cell lines and exosomes derived by these cells and serum-circulating exosomes of cancer patients. Contrary to highly differentiated CaCo-2 cells, HT29 cells have an intermediate capacity to differentiate. Due to the fact that the lower propensity of tumour cells to differentiate, in general, corresponds to higher malignancy of the tumour and higher proportion of CSCs in it, CSC markers expression should be higher in HT29 cells as compared to CaCo-2 cells. *Materials and methods*: Surface marker profiling of HT29 and CaCo-2 was performed using flow cytometry. Exosomes were either isolated directly from cell culture supernatants by immuno-magnetic extraction or purified by ultracentrifugation followed by immuno-precipitation on Dynabeads (Life Technologies). Bead–exosome complexes were stained and analysed by flow cytometry. *Results and discussion*: CD66 and A33 are highly expressed in both HT29 and CaCo-2 cells and their exosomes and could be used for immuno-isolation of colon cancer exosomes. The fraction of CD166+ cells and exosomes was observed exclusively in HT29. The pilot experiments aimed to analyse the expression of CD166 on serum-circulating exosomes of colon cancer patients revealed those of them with potentially high risk for tumour progression.

**The influence of bowel preparation and colonoscopy on the secretion of circulating microvesicles**

T. Hornung, C. Alva, A. Benton, S. Logie, M. Maheshwari, J. Kimbrough, S. Kankipati, B. Rhees and D. Spetzler

Caris Life Sciences, USA

Circulating microvesicles (cMV) are small membrane structures that are secreted by multiple cell types and have been found in blood, urine, saliva and other body fluids. cMV transfer information from cell to cell by transporting selected proteins, mRNA and microRNA that correlate to their cell of origin. Due to the abundance of biomarkers secreted from diseased cells, cMV are of particular interest in discovering new assays. The number of cMV shed by cells increases when the cell is biochemically stressed. To determine if the physical stress associated with bowel preparation and colonoscopy would result in an increase in the amount of colon cMV shed into the vascular system, blood was collected prospectively from 27 individuals at different time points and processed into plasma. Five time points were chosen for this study to establish the basal level of colon cMV, the effect of the procedure on cMV levels and when cMV levels return to baseline. Specifically, the five time points were (1) before bowel preparation; (2) after bowel preparation and before colonoscopy; (3) one day post-colonoscopy; (4) 3–5 days post-colonoscopy; and (5) one week post-colonoscopy. The cMV levels were profiled using 115 protein markers that have been correlated to colon tissue, or colon cancer in the literature. There was no statistical difference between any of the time points, suggesting that neither bowel preparation nor colonoscopy influence the secretion and composition of cMV; thus, the physical stress generated by the colonoscopy procedure does not appear to influence the secretion of colon cMV.

**Modulation of extracellular vesicles dynamics upon therapeutic treatment of glioblastoma**

Katy A. Wong, Victoria A. Appleman, H.J. Jun, Lily Keung and Alain Charest

Tufts Medical Center, Boston, MA, USA

*Introduction*: Glioblastoma multiforme (GBM) is the most common and lethal form of malignant primary brain cancers. Although advances have been made in understanding GBM biology, little clinical improvement has been observed. Poor patient survival is due to rapid growth, heterogeneous cell population and extreme invasiveness of these tumours. These characteristics of GBM make the tumour microenvironment an important factor when studying GBM biology and therapeutics. A recently discovered method by which tumours communicate with the surrounding environment is by the release of extracellular vesicles (EVs). GBM tumours have been shown to differentially sort cargo into EVs. These EVs are then able to deliver their cargo to surrounding cells and thus modify their physiology. Our lab set out to investigate how EV number and cargo is altered upon therapeutic treatment, and how these vesicles modify the tumour microenvironment. *Methods*: GBM formation in our mouse models for human GBM is driven by the somatic over-expression of human EGFR and loss of the tumour suppressor gene Cdkn2a. Using these models and primary cultures of GBM cells derived from them, we are able to study EVs release and content both in vivo and in vitro. Our system allows us to use these cells to isolate tumour EVs and to examine their number and cargo. *Conclusions*: Using our models we observed a decrease in serum-derived EVs upon tumour formation. During the course of therapeutic intervention, we also observed a significant increase in tumour microglia infiltration upon treatment with EGFR inhibitor, suggesting that inhibition of EGFR alters the tumour microenvironment. Using primary GBM cells, we have demonstrated that EV cargo is differentially sorted upon treatment. Finally, we demonstrate that isolated EVs from primary GBM cells are incorporated into microglia cells. Our data suggest that treatment of GBMs with clinically relevant therapies alters tumour microenvironment through EVs content.

**Exosome as potential biomarkers in oncology drug development**

Shidong Jia

Genentech Inc, USA

*Introduction*: Exosomes are promising resources for biomarker development in cancer. Currently, we are assessing the potential of exosomes as surrogate biomarkers in drug development. *Material and methods*: Cell lines, patient tumour tissues and matched plasma are used to study the plasma exosomes- and tumour-derived gene signature (mRNA and microRNA). *Results*: We have obtained exosome-based gene signature using plasma exosomes. Studies are ongoing to explore the correlation between plasma exosomes- and tumour-derived gene signature using patient samples. *Conclusion*: Plasma exosome is a promising resource of biomarkers for oncology drug development.

**Pancreatic cancer serum exosomes as diagnostic marker**

Margot Zöller ^1^, B. Madhavan^1^, S. Rana^1^, S. Yue^1^, N. Giese^2^, H. Kalthoff^3^ and M.W. Buechler^2^

^1^Tumor Cell Biology, University Hospital of Surgery, Germany; ^2^University Hospital of Surgery, Germany; ^3^Molecular Oncology, University Kiel, Germany

*Introduction*: Dismal prognosis of pancreatic cancer (PaCa) due to late diagnosis urgently suggesting a reliable non-invasive diagnosis, we explored, whether PaCa serum exosome proteins or miRNA provide a diagnostic means. *Material and methods*: Exosomes were collected from serum of healthy donors and patients with PaCa, benign tumours or chronic pancreatitis. Exosomal proteins were analysed by flow cytometry and miRNA by qRT-PCR. miRNA was also analysed in exosome-depleted serum. *Results*: Serum exosomes were tested for CD44v6, MET, Tspan8, EpCAM and CD104, highly expressed on PaCa culture-derived exosomes. CD9, CD63 and CD151, also expressed on healthy donor exosomes, served as controls. PaCa patients’ exosome reactivity differed significantly from that of all control groups, but chronic pancreatitis patients’ exosome reactivity was comparably high, which was corrected by omitting MET. Thereby pancreatitis patients’ exosome reactivity was reduced to 9%, with 92% of PaCa patients’ exosomes remaining positive. PaCa patients’ exosome reactivity was independent of tumour grading and staging, reactivity being seen even in Tis and T1 stages and not being affected by metastasis. To select for PaCa-related miRNA, microarray analysis was performed with pools of serum exosomes and exosome-depleted serum from healthy donors and PaCa patients. PaCa culture supernatant exosomes served as control. PaCa patients’ serum exosome miRNA differed strikingly from that of healthy donors; such distinctions were not observed with miRNA from PaCa versus healthy donors’ exosome-depleted serum. Surprisingly, miRNA with highest recovery in PaCa patients’ serum exosomes also were highly enriched in PaCa culture supernatant exosomes. Based on these results, serum exosomes are currently analysed for highly expressed miR-1246, -4708, -4800, -3960, -4454, not recovered in healthy donors’ exosomes. *Conclusions*: For diagnosis, we suggest to combine protein and miRNA analyses, as some PaCa patients’ serum exosomes were n

**Vesicle RNA signature as a treatment-independent biomarker in acute myeloid leukaemia**

N.I. Hornick, J. Huan, N.A. Goloviznina and P. Kurre

Pape Pediatric Research Institute, Department of Pediatrics, Oregon Health & Science University, USA

*Introduction*: Acute myeloid leukaemia (AML) is the most common acute leukaemia in adults. Although the majority of treated patients achieve remission, more than half of them relapse. Current post-remission surveillance requires bone marrow aspiration, an invasive procedure that suffers from sampling bias to a single region of marrow. We recently showed that AML cells release exosomes containing leukaemia-specific RNA. Here, we investigated in a murine xenograft model whether exosomes can provide a minimally invasive, cell-free RNA biomarker platform to track residual disease. As clinically useful biomarkers should be durable throughout cycles designed to disrupt cancer cell signalling pathways, we were also interested in how drugs commonly used during AML therapy impact candidate RNA incorporation. *Materials and methods*: In vivo: engraftment of MOLM-14 into NSG mice, serum collection. In vitro: culture of leukemic cell lines including drug treatment with cytarabine, quizartinib and ruxolitinib; vesicle isolation via centrifugation, qRT-PCR. *Results*: We isolated vesicles from the serum of NSG mice engrafted with an AML cell line and were able to detect both leukaemia-specific mRNA and a consistent pattern of miRNA levels in engrafted animals that differed from the controls. Towards determining whether these changes were affected by therapy, we evaluated vesicle RNA from serum samples isolated from a variety of leukemic cell lines before and after in vitro exposure to cytarabine, quizartinib and ruxolitinib. Results show that vesicle RNA was not only enriched over cellular background, but levels were stable throughout treatment. Future experiments will evaluate the stability of vesicle RNA as a biomarker through in vivo leukaemia treatment. *Conclusions*: Circulating vesicle RNA changes coincide with the development of leukaemia in AML-engrafted mice, and these changes withstand in vitro drug treatment of leukemic cells.

**Insights into prostate biomarkers using urinary exosome/microvesicle RNA**

Leileata Russo ^1^, A.N. Scott^1^, P. Motamedinia^2^, K.L. Bate^1^, N. Sadeghi^2^, G. Salazar^1^, M. Lipsky^2^, J. Lin^2^, G. Hruby^2^, K.K. Badani^2^, D.P. Petrylak^2^, W.D. Comper^1^ and J.M. McKiernan^2^

^1^Exosome Diagnostics, USA; ^2^Columbia University, USA

*Introduction*: Clinical use of exosomal/microvesicle RNA (exoRNA) relies on a “clinically viable” isolation method and robust exoRNA compatible biomarkers. Here we examine the accuracy of exoRNA to predict tissue expression of the prostate cancer-related gene fusion TMPRSS2:ERG (T:E) as well as determine the ability of biomarkers to differentiate prostate cancer-positive (BxPos) from prostate cancer-negative (BxNeg) patients. *Materials and methods*: Urinary ExoRNA was isolated using a filtration-based technology followed by RNA extraction. To determine concordance between tissue and exoRNA, T:E expression in pre-radical prostatectomy (RP) urine versus prostate tissue was compared. To examine the correlation of exoRNA derived T:E expression with age, prostate specificity and prostate cancer, a cohort of 207 subjects (BxNeg (n=39), BxPos (n=47), post-RP (n=37), no biopsy (n=44) and male controls (<35 years) (n=40)) was examined. Receiver operator characteristic (ROC) curves were used to determine whether coding and non-coding prostate cancer biomarkers could differentiate BxPos (n=47) from BxNeg patients (n=39) using exoRNA standardised to the prostate marker KLK3. *Results*: Correlation between T:E expression in tissue versus exoRNA demonstrated a sensitivity of 81.25% (13/16), specificity of 80% (4/5) and an overall accuracy of 81% (17/21). The rate of T:E increased with age and expression level was correlated with BxPos status. Analysis of exoRNA demonstrated that genes including PCA3 (ROC 0.695), ERG (ROC 0.802), T:E (ROC 0.763) and BIRC5 (ROC 0.703) could segregate BxNeg from BxPos patients. *Conclusion*: Analysis of exoRNA in young males gave rare insights into prostate cancer gene expression and analysis of post-RP urine confirmed that T:E expression was prostate specific. ExoRNA was found to reflect tissue expression and previously identified prostate cancer biomarkers were found to perform in exoRNA. This study adds to our understanding of exoRNA as a potential diagnostic platform.

**Protein and TGF-beta1 levels of exosomes persisting in AML plasma during chemotherapy indicate the presence of residual disease**

M.L. Boyiadzis, C.S. Hong and T.L. Whiteside

University of Pittsburgh Cancer Institute, USA

*Introduction*: Exosomes isolated from plasma of patients with newly diagnosed AML were found to be enriched in TGF-beta1, MICA/MICB, blast markers CD33, CD34, CD117 and inhibited functions of natural killer (NK) cells. To judge the impact of blast-derived exosomes on the disease process, their protein and TGF-beta1 content were evaluated in cohorts of AML patients undergoing chemotherapy (CT). *Materials and methods*: Plasma specimens were collected at the time of AML diagnosis (n=16); after induction CT (n=10); during consolidation CT (n=7) and from patients in long-term remission (n=4). Exosomes were isolated from patients’ plasma by exclusion chromatography followed by ultracentrifugation, and their protein content (µg/mL plasma) was compared as was TGF-beta1 expression by semi-quantitative western blots. The results were correlated with the patients’ cytogenetic profile, percentage of blast in bone marrow and clinical data. *Results*: At diagnosis, the protein and TGF-beta1 contents of exosomes were higher (p<0.0001) than those in exosomes from normal donors’ plasma. These values decreased in exosome fractions in response to induction CT (p<0.001). During consolidation CT, protein/TGF-beta1 levels in exosomes were persistently but variably elevated, and in the long-term remission group, they decreased to normal plasma levels. *Conclusions*: The persistence and protein/TGF-beta1 content of AML exosomes pre- and during consolidation CT are consistent with the presence of residual disease when leukemic blasts are undetectable in the bone marrow. Similar analyses of serially acquired specimens are in progress to confirm the predictive impact of exosomes in AML.

**The exosomal transport of the tumour suppressor PTEN is regulated by p53**

Putz Ulrich, A. Doan and S.S. Tan

The Florey Institute, Australia

*Introduction*: The two most important tumour suppressors, p53 and PTEN (phosphatase and tensin homolog), have multiple layers of direct and indirect regulation. We have shown that PTEN can be exported in exosomes under the control of Ndfip1, an adaptor for Nedd4 ubiquitin ligases. Exosomal PTEN can be internalised by recipient cells with functional effects and strong implications for using exosomal PTEN as a mode of portable tumour suppression. Here, we provide evidence for a new layer of control of PTEN by p53, another tumour suppressor protein. *Material and methods*: We used the supernatant from transfected 293T cells, wild-type and p53 KO MEFs to harvest exosomes for electron microscopy, western blotting and uptake experiments. *Results*: We demonstrate that p53 can bind Ndfip1. Over-expression of p53 in 293T cells led to a reduction of Ndfip1 and PTEN, but not other proteins, in exosomes. Removal of p53 in KO MEFs produced more Ndfip1 in exosomes compared to wild-type MEFs. These results identify p53, the most commonly mutated tumour suppressor protein, to be an important regulator for exosomal export of PTEN and Ndfip1. This degree of molecular crosstalk between the two most prominent tumour suppressors in exosomal export of PTEN was unanticipated and suggests that intercellular communication via exosomes needs to be considered as an additional modus vivendi for tumour suppression. *Conclusions*: The ability of p53 to regulate exosomal secretion of PTEN has significant implications for how tumour suppressors use exosomes to communicate between cells.

**Poster Session II (Statler): Biogenesis April 17**

**Chair: *S. Gould and P. Zimmermann***

**Role of galectin-3 in exosome biogenesis and secretion in dendritic cells**

Meng-Lin Chiang, P.C. Chiang, D. Hsu, H.Y. Chen, and F.T. Liu

Institute of Biomedical Sciences, Academia Sinica, Taiwan

Exosomes are small vesicles (50–100 nm) secreted by different cell types. The exosomal secretion pathway is primed by intraluminal vesicles (ILVs) budding into the late endosomes to form multivesicular bodies (MVBs), which then fuse with the plasma membrane resulting in the release of ILVs to the extracellular space. The exosomal component proteins include the ESCRT (endosomal sorting complex required for transport) family members (Alix, Tsg101), tetraspanins family members (CD9, CD63, CD81), membrane-trafficking members (Rab5, Rab11b) and galectin-3. Exosomes function in cell-to-cell communication, antigen presentation, reticulocyte differentiation and tumour progression. However, the mechanism of exosome secretion and biogenesis is still unclear. Galectin-3 has been identified as a component of dendritic cell (DC)-derived exosomes by proteomic analysis. As galectin-3 can be associated with Alix in a number of cell types, we hypothesise that galectin-3 may participate in exosome biogenesis through interacting with Alix. Exosomes were purified from gal3+/+ or gal3–/– BMDCs by ultracentrifuge and subject to immunoblotting analyses, semi-quantitative fluorescence-activated cell scanning (FACS)-based assay and immuno-EM to identify exosomal phenotype. Finally, MVBs in gal3+/+ and gal3–/– BMDCs were studied by immunofluorescence staining and immune-EM. Immunofluorescence staining showed that galectin-3 is co-localised with Alix and CD63 in BMDCs. Immuno-EM and immunoblotting analyses showed that galectin-3 is expressed on the surface as well as inside of DC-derived exosomes. By comparing exosomes collected from BMDCs, we further discovered that gal3–/– BMDCs secreted more exosomes than gal3+/+ BMDCs. Immunoblotting analysis of the protein composition indicated exosomes from gal3–/– BMDCs contained different levels of protein components compared to those from gal3+/+ BMDCs. These results suggest that galectin-3 may play a role in regulating exosome biogenesis and secretion.

**Stimulus-dependent properties of neutrophil-derived microvesicles**

Erzsébet Ligeti, M.Á. Lőrincz, C.I. Timár and K. Marosvári

Department of Physiology, Semmelweis University, Hungary

*Introduction*: We have shown earlier that both the number and the biological properties of microvesicles (MVs) produced from isolated neutrophilic granulocytes (PMN) depended on the type of stimulant. Chemotactic agents, bacterial products and cytokines produced few MVs that did not affect bacterial growth. In contrast, stimulation with opsonised particles initiated the release of MVs with significant antibacterial capacity. Proteomic analysis supported the stimulus-dependent difference of MV composition (1). The mechanism of formation of antibacterial MVs will be explored in this presentation. *Materials and methods*: PMN isolated from healthy volunteers were stimulated with bacteria or zymosan particles under different conditions of opsonisation and for different incubation times. The number of released MVs was determined by flow cytometry. Protein content was also determined. The effect of the produced MVs on bacterial growth was measured by our semi-automated method in an ELISA reader and the aggregation ability was followed by confocal microscopy. *Results*: Stimulation of PMN with opsonised *Saphylococcus aureus* or *Escherichia coli* or zymosan particles resulted in the formation of MVs that impaired bacterial growth to similar extent. Both the incubation time and the opsonisation conditions proved to be critical for the development of the antibacterial capacity of MVs. Superoxide production by the NADPH oxidase and glucose supply were not critical for the production of antibacterial MVs, whereas interference with cytoskeletal remodelling prevented MV formation. *Conclusion*: Phagocytosis seems to be the key factor leading to release of MVs with antibacterial properties, but the different plasma membrane receptors (PRR, Fc and CR) play differential roles.

Funded by: Supported by OTKA 75084 and TAMOP4.2.2/B10/1-2010-0013

Reference

1. Timar et al. Blood. 2013.

**Exosomes derived from liver mitochondria are present in human plasma: activity of ornithin carbamoyl transferase as a potential marker of exosome release under metabolic stress**

Irma Aguilar-Delfin ^1^, R. Hernandez-Munoz^2^ and L. Orozco^1^

^1^Instituto Nacional de Medicina Genomica, Mexico; ^2^Instituto de Fisiologia Celular, UNAM, Mexico

*Introduction*: Ornithin carbamoyl transferase (OCT) is a large, homotrimeric mitochondrial enzyme encoded in the nucleus that acquires enzymatic activity only after precursors synthesised in the cytosol are imported and assembled into the mitochondrial matrix. OCT gene is expressed exclusively in hepatocytes. However, OCT activity has been demonstrated in non-hepatic tissues including leukocytes, lung and blood. Ectopic OCT activity would imply enzyme translocation through both mitochondrial membranes and the plasma membrane of the hepatocyte. We set out to investigate whether functional OCT is found in human plasma and plasma exosomes and if there are differences between individuals with type-2 diabetes and healthy controls. *Material and methods*: Peripheral blood samples were collected in EDTA vacutainer tubes from 108 adult fasting individuals. Plasma was separated within 8 h of blood collection and kept at 4°C. Three pools of 36 samples were ultracentrifuged to obtain the exosomal fraction (500*g* 30 min, 12,000*g* 45 min, 110,000*g* 120 min). OCT enzymatic activity was quantitated in pooled whole plasma, exosomes and fractions from intermediate purification steps, as well as an independent set of plasma samples from type-2 diabetic individuals and healthy controls (n=40). *Results*: Catalytically active OCT was found in human plasma and appears to be enriched in the fraction corresponding to exosomes. OCT activity was significantly increased in individuals with type-2 diabetes compared to healthy controls (p=0.034). *Conclusions*: Catalytically active OCT is released into human plasma at least partially inside exosomes. This might explain the ectopic OCT activity documented in phagocytic cells and extrahepatic organs. Furthermore, it could be evidence that in hepatocytes mitochondrial matrix contents can be shuttled into exosomes by a yet uncharacterised pathway. Plasma OCT in type-2 diabetes suggests mitochondrially derived exosomes might be markers of metabolic stress.

**Non-conventional apical secretion of galectin-8 is mediated by exosomes in MDCK cells**

M.A. Lopez-Verrilli, C. Oyanadel, A. Soza and A. Gonzalez

CARE (12/2007) Fac Ciencias Biológicas and Dept Inmunología Clínica y Reumatología, Fac Medicina, P, Chile

*Introduction*: Galectins are a family of carbohydrate-binding proteins implicated in a variety of cellular processes, including adhesion, migration and apoptosis. Although galectins lack the classical signal peptide they are released to the extracellular medium by a non-conventional secretory route. Galectin-8 (Gal-8), one of the most widely expressed galectins, is involved in tumour progression and modulation of immune responses. Here, we study the polarity and the possibility of secretion of Gal-8 via exosomes in polarised epithelial cells. *Materials and methods*: MDCK cells stably expressing Gal-8 were cultured in transwell filters and pulse-chase metabolic experiments using 35S-Met/Cys during different times were performed. The conditioned media was precipitated with 10% trichloroacetic acid and the synthesised Gal-8 was measured by western blot. Exosomes were purified from confluent MDCK cells by ultracentrifugation and characterised by electron microscopy and western-blot. *Results*: Pulse-chase experiments performed in fully polarised MDCK cells growing in transwell filters detected newly synthesised Gal-8 only in the apical medium, as soon as 30 min after the pulse. Conditioned media from confluent MDCK cells maintained in D-MEM for 48 h was almost completely depleted of Gal-8 after ultracentrifugation. Gal-8 was detected in the exosome fraction together with the exosome markers CD63, Hsp70, Hsp90 and flotillin. Sensitivity to trypsin digestion indicated that Gal-8 is present inside the exosomes, while treatment with 50 mM lactose to detach Gal-8 from cell surface glycans, as well as electron microscopy, revealed Gal-8 also on the surface of exosomes. *Conclusion*: Gal-8 is apically released by MDCK cells involving non-conventional secretion of exosomes.

Funded by: Supported by FONDECYT 11121445 and Programa de Financiamiento Basal 12/2007

**The role of extracellular vesicles derived from bladder cancer cells in intercellular communication**

Eva Ogorevc ^1^, P. Veranic^2^, S. Hudoklin^2^ and V. Kralj-Iglic^3^

^1^Faculty of Electrical Engineering, University of Ljubljana, Slovenia, Slovenia; ^2^Institute of Cell Biology, Faculty of Medicine, University of Ljubljana, Slovenia; ^3^Faculty of Health Sciences, University of Ljubljana, Slovenia

*Introduction*: Extracellular vesicles (EVs) derived from tumour cells can take part in altering non-cancerous counterparts to facilitate tumour growth and invasion. It is becoming evident that EVs may emerge from membrane rafts; however, the mechanisms of EV interaction with recipient cells are still poorly understood. Fusion of EV with plasma membrane and endocytosis (phagocytosis) are currently supposed to be the two most possible mechanisms of EV uptake. The aim of the present study was to further investigate the mechanisms of intercellular transfer of bladder cancer cell-derived EVs to non-cancerous and cancerous cells. *Materials and methods*: EVs were isolated from culture medium of human urinary bladder epithelial cancerous cell line (T24), labelled with fluorescent lipophilic dyes (DiO, DiI) and membrane raft markers (cholera toxin). The isolated EVs were applied to human cell lines (T24, RT4) and mouse cell line (G/g). The uptake of tumour EVs by recipient cells was assessed by fluorescent and electron microscopy. *Results and discussion*: The fluorometric analysis, indicating the efficiency of EV transfer, showed that in recipient cells the highest quantity of fluorescence was in RT4 cells, moderate in G/g cells and lowest in T24 cells. Low rate of EV-derived fluorescence in recipient T24 cells might be the consequence of the clearance of fluorescent dyes by intense subsequent vesiculation of plasma membrane, characteristic for cancer cells. The uptake of EVs by G/g cells indicates the possibility of interspecies communication via membranes. Our recipient cells were found to uptake EVs mostly by phagocytosis; however, to a smaller extent also by membrane fusion. *Conclusions*: Our observations show that EVs can mediate the intercellular communication between bladder cancer and non-cancerous cells. Moreover, phagocytosis seems to be the most efficient way of EV uptake.

**Nanoparticle tracking analysis of exosomes and microvesicles secreted by cell lines inhibited for expression of RAB27**

Simon Powis, Y. Zheng, E. Campbell and A. Riches

University of St Andrews, United Kingdom

*Introduction*: Nanoparticle tracking analysis (NTA) allows the rapid detection and quantification of exosomes and microvesicles present in tissue culture supernatants and biological fluids. We have used RNA inhibition to target Rab27 components of the exosome pathway and determined by NTA the effects on exosome secretion. *Material and methods*: Short hairpin RNA targeted to Rab27a was transfected into the breast cancer cell line MDA-MB-231 cells. NTA was used to assess the release of exosomes into the culture supernatant. Purified exosomes from wild-type MDA-MB-231 cells were added back to shRNA targeted cell cultures and colony formation determined. *Results*: shRNA targeting of Rab27a resulted in inhibition of both Rab27a and Rab27b expression and increased CD63-positive vesicular structures inside cells. Lower numbers of exosomes were detected in the culture supernatants of shRNA targeted cells as determined by NTA. The addition of purified wild-type exosomes restored the colony growth capacity of the shRNA targeted cells. *Conclusions*: Inhibition of the Rab27 pathway impacts on exosome secretion, which can be easily detected by NTA technology. Thus NTA presents a rapid screening tool for drugs and agents that influence the release of exosomes and microvesicles.

**Mutation of simple in Charcot-Marie-Tooth 1C alters production of exosomes**

H. Zhu^1^, S. Guariglia^2^, R.Y.L. Yu^3^, W. Li^3^, D. Brancho^3^, H. Peinado^4^, D. Lyden^4^, J. Salzer^5^, C. Bennett^6^ and C.W. Chow ^1^

^1^Albert Einstein College of Medicine, USA; ^2^CUNY College of Staten Island, USA; ^3^Albert Einstein College of Medicine, USA; ^4^Weill Cornell Medical College, USA; ^5^New York University Langone Medical Center, USA; ^6^University of Washington School of Medicine, USA

*Introduction*: Charcot-Marie-Tooth (CMT) disease is an inherited neurological disorder. Mutation found in protein SIMPLE accounts for the rare autosomal dominant demyelination in CMT1C patients. Molecular basis of these SIMPLE mutations in causing autosomal dominant demyelination in CMT1C remains elusive. The lack of knowledge on the role of SIMPLE further complicates the understanding of SIMPLE in CMT1C demyelination. *Materials and methods*: We introduced by gene targeting a single point mutation in floxed exon 3 of the Simple gene to convert the codon Thr115 (ACC) to that for Asn (AaC) (T115N) and model CMT1C disease. Our targeting model also allows deletion of exon 3 to generate SIMPLE null. Primary cells from CMT1C and SIMPLE null mice were isolated to investigate the function of SIMPLE and the molecular pathology of CMT1C. *Results*: Previous studies indicated localisation of SIMPLE in endosomal trafficking compartments. Immunoelectron microscopy confirmed localisation of SIMPLE in endosome-like tubular structures and lysosome-like electron dense granules. Surprisingly, we also found membrane-bound SIMPLE on intraluminal vesicles inside MVBs. Indeed, SIMPLE is secreted via the exosome pathway. In addition, we showed that expression of SIMPLE increases the number of exosomes and secretion of specific exosome proteins. Conversely, CMT1C mutations abrogate exosomal localisation of SIMPLE. Furthermore, secretion of exosomes is compromised upon CMT1C mutation or ablation of SIMPLE. In CMT1C cells, vacuolated appearances in MVBs were observed. CMT1C patient B cells also elicited similar defects in the production of intraluminal vesicles and generates vacuolated appearances in MVBs. *Conclusions*: We have demonstrated that SIMPLE plays a critical role in exosome formation. Mutation of SIMPLE, which abolishes the proper formation of MVBs and exosome biogenesis, could elicit intracellular and extracellular consequences and account for the CMT1C molecular pathogenesis.

**Deciphering exosome biogenesis in *C. elegans*: focus on the exocyst**

V. Hyenne, M. Diem, A. Apaydin, Y. Schwab and M. Labouesse

IGBMC, Strasbourg, France

*Introduction*: Despite intensive characterization of exosome contents in various systems, little is known about the mechanisms governing their formation within the cell. Exosomes arise from the fusion of multi-vesicular bodies with the plasma membrane, but the molecular pathways involved in MVB formation, their targeting to the plasma membrane, as well as their attachment and fusion remain unclear. *Materials and methods*: To address this question, we use the model organism *C. elegans*, which presents the advantage of allowing a combination of genetics, live imaging and electron microscopy in a multicellular organism. Our laboratory has previously shown that extracellular vesicles, presumably exosomes, are released at the apical surface of epithelial cells and contribute to the formation of a particular cuticular structure of the worm, called the alae. *Results*: We designed a RNAi based screen for alae defects in *C. elegans* and identified over 60 genes that could be involved in exosome biogenesis. We are characterising these genes by 1) analysing their expression pattern and dynamics by rapid confocal 2) quantifying the effects of their depletion on the structure of the MVBs within the epithelial cells by electron microscopy. Here, we present our results on the involvement of the evolutionarily conserved exocyst complex and its regulator, the GTPase ral-1,in the attachment of MVBs to the apical plasma membrane of epithelial cells in *C. elegans*. *Conclusions*: Our study demonstrates the importance of ral-1 and the exocyst in exosome release. We expect that the study of other candidate genes found in the screen will allow us to define a molecular cascade controlling the biogenesis of these extra-cellular vesicles.

**The flippase APT1 is involved in mechanisms of vesicular export of polysaccharides in the fungal pathogen cryptococcus neoformans**

J.A. Rizzo ^1^, L.S. Joffe^1^, D.L. Oliveira^2^, F.G. Lopes^3^, G. Hu^2^, S. Frases^1^, F.L. Fonseca^1^, I.C. Almeida^3^, J.W. Kronstad^2^ and M.L. Rodrigues^1^

^1^Universidade Federal do Rio de Janeiro, Brazil; ^2^The University of British Columbia, Canada ^3^The University of Texas at El Paso, United States

*Introduction*: In eukaryotes, many pathways are involved in extracellular vesicle (EV) formation, including those requiring aminophospholipid translocases (flippases, APTs). A previous study demonstrated that a *Cryptococcus neoformans* (Cn) mutant lacking APT1 expression had defects in ER-Golgi trafficking and attenuated virulence. Pathogenesis in Cn requires EV-mediated export of polysaccharides (PS). In this context, we hypothesised that EV-mediated PS export and Apt1p are functionally connected in Cn. *Materials and methods*: Cn EVs were purified from culture supernatants of wild type (WT) and Δapt1 mutant cells for sterol determination by thin layer chromatography, analysis of dimensions by dynamic light scattering and PS determination by serologic methods. The morphological aspects of WT and Δapt1 cells were analysed by fluorescence and scanning electron microscopy. Structural aspects of the PS were analysed by gas chromatography coupled to mass spectrometry. Analysis of pathogenic mechanisms was performed by infecting mice with WT or Δapt1 cells for further determination of fungal survival in host tissues. *Results*: The Δapt1 mutant and WT cells shared important phenotypic characteristics, including capsule morphology and size, PS composition, molecular dimensions and serologic properties. The Δapt1 mutant, however, was more efficient in producing EVs, in comparison to WT cells. EVs produced by mutant and WT cells had similar diameters. However, the Δapt1 mutant produced EVs with lower PS content. This observation correlated with diminished PS production during both regular growth and macrophage infection. This phenotype was associated with decreased survival in the lung of infected mice and inefficacy to colonize the brain. *Conclusions*: Lack of APT1 caused defects in both PS synthesis and vesicular export to the extracellular milieu by Cn via processes that are apparently related with the pathogenic mechanisms used by this fungus during animal infection.

**Poster Session II (Statler): Organisms April 17**

**Chair: *D. Mulhall and Y.Y. Kim***

**In vitro and in vivo endothelial cell activation by bacterial extracellular vesicles**

Y.J. Yoon ^1^, J.H. Kim^2^, E.J. Choi^2^, S.C. Jang^2^, S.R. Kim^2^, J. Lötvall^3^, Y.K. Kim^2^ and Y.S. Gho^2^

^1^Pohang University of Science and Technology, School of Interdisciplinary Bioscience, Republic of Korea; ^2^Pohang University of Science and Technology, Department of Life Science, Republic of Korea; ^3^University of Gothenburg, Department of Internal Medicine, Sweden

*Introduction*: Gram-negative bacteria, including Escherichia coli, constitutively release extracellular vesicles, also known as outer membrane vesicles (OMVs). Recently, it has been reported that E. coli OMVs induce systemic inflammatory response as characterised by systemic induction of proinflammatory mediators and dysfunction of the lung. However, the exact role of endothelial cells in OMV-induced systemic inflammatory response is not completely understood. *Material and Methods*: Bacteria-free OMVs were purified from E. coli, which was isolated from the peritoneal lavage fluid of cecal ligation and puncture-operated mice. These OMVs were intraperitoneally injected into wild-type or TLR4 knockout mice and inflammation in the lungs was assessed by measuring the expression of cytokines and chemokines and leukocyte recruitment. The involvement of TLR4 was studied in the endothelial cells and HEK293 cells expressing human TLR4 and MD2 by the treatment of LPS antagonist. *Results*: RT-PCR analyses revealed that human microvascular endothelial cells express TLR4, MD2, MyD88, Mal, TRIF, and TRAM. The addition of bacteria-free OMVs to human endothelial cells significantly increased the expression of IL-8 and CXCL10 in a dose-dependent manner. In addition, we revealed that OMV-induced endothelial production of IL-8 and CXCL10 was MyD88- and TRIF-dependent, respectively. Moreover, the OMV-induced expression of these cytokines was suppressed by the small interference RNA-mediated down-regulation of either MyD88 or TRIF and NF-κB inhibitor. Furthermore, the production of IL-8 and CXCL10 by OMVs was effectively inhibited by the LPS antagonist in vitro and in TLR4 knockout mice in vivo. *Conclusions*: Our results indicate that E. coli-derived OMVs potently induce the innate immune responses by the TLR4-dependent production of proinflammatory cytokines in endothelial cells in vivo.

**Environment dependent regulation of membrane vesicle production in *Pseudomonas aeruginosa***

Masanori Toyofuku, H. Uchiyama and N. Nomura

University of Tsukuba, Japan

*Introduction*: Gram-negative bacteria naturally secrete sphere shaped vesicles that are approximately 50 to 250 nm. These vesicles are mainly composed of cellular outer membrane and hence called membrane vesicles (MVs). MVs are related to the pathogenesis of several bacterial pathogens where they function as a cargo for virulence factors against their host. MVs also play important role in the bacterial ecology by functioning in the competition against other cells, or on the other hand, by carrying signaling molecules to support the cooperative behaviors of bacteria. In addition, it is proposed that MVs are involved in cell material transfer among bacterial cells such as plasmid transfer. While it is suggested that MVs have great impact on the bacterial physiology, the biogenesis of MVs are not fully understood. Here we report a new regulatory pathway involved in *Pseudomonas aeruginosa* MV formation. *Material and Methods*: MVs from anaerobic conditions were purified by density gradient ultracentrifugation. Gene knock out mutants were produced by homologous recombination. *Results*: The production of MVs in P. aeruginosa depends largely on the presence of an intercellular signal, PQS. Here we found that P. aeruginosa produce MVs even under anaerobic conditions where PQS is not produced. Further analysis revealed that SOS response is involved in the production of MVs, and nitric oxide may be triggering this response under anaerobic conditions. These results demonstrate a novel pathway in *P. aeruginosa* MV production.

**Staphylococcus aureus extracellular vesicle-mediated non-inherited β-lactam resistance**

J. Lee ^1^, E.Y. Lee^1^, D.K. Kim^1^, K.S. Park^1^, K.P. Kim^2^, Y.K. Kim^1^, T.Y. Roh^1^ and Y.S. Gho^1^

^1^Pohang University of Science and Technology, Department of Life Science, Republic of Korea; ^2^Konkuk University, Department of Molecular Biotechnology, Republic of Korea

*Introduction*: Gram-negative bacteria produce extracellular vesicles (EVs), also known as outer membrane vesicles, which play diverse roles in intercellular communication. Recently, it has been reported that Gram-positive bacteria, including *Staphylococcus aureus*, also produce EVs. However, little is known about the functions of Gram-positive bacterial EVs, especially in the bacterial community. Here, we studied the role of *S. aureus* EVs in inter-bacterial communication under antibiotic stress. *Materials and methods*: EVs were purified from the culture supernatant of ampicillin-resistant and ampicillin-susceptible *S. aureus* by ultracentrifugation. *S. aureus* EV-mediated survival of ampicillin-susceptible bacteria was measured in the presence of ampicillin. The effect of ampicillin on the production of EVs and their associated beta-lactamase (BlaZ) was investigated. *Results*: Although *S. aureus* beta-lactamase was known to be secreted as a soluble form, we found that ampicillin-resistant *S. aureus* liberated beta-lactamase, BlaZ, via EVs. These BlaZ-associated EVs rendered other Gram-negative and Gram-positive bacteria to survive against ampicillin treatment by hydrolysing the environmental beta-lactams. However, the survival was transient and *S. aureus* EVs did not contain blaZ genes, suggesting that the EV-mediated antibiotic resistance occurs through a non-inherited mechanism. In addition, ampicillin treatment to ampicillin-resistant *S. aureus* significantly increased the release of EVs (3-folds) and their associated BlaZ (17-folds). *Conclusions*: Our observations provide a novel non-inherited mechanism of antibiotic resistance transfer, by which *S. aureus* EVs contribute to the bacterial community to evolve and prosper against antibiotic attack.

**RNA transcriptome of fungal extracellular vesicle preparations from pathogenic species of *Paracoccidioides***

R.S. Peres da Silva^1^, L.R. Alves^2^, M.L. Rodrigues^3^, J.P.C. Cunha^4^, S. Goldenberg^2^ and R. Puccia ^1^

^1^EPM-UNIFESP, Brazil; ^2^ICC/FioCruz, Brazil; ^3^Microbiologia/UFRJ and CDTS/FioCruz, Brazil; ^4^IBU, Sao Paulo, SP, Brazil

*Introduction*: We have recently characterised extracellular vesicular structures (EV) in the yeast pathogenic phase of *Paracoccidioides brasiliensis* that together with *Paracoccidioides lutzii* (Pb01) causes human paracoccidiodomycosis (PCM). EV were found in both Pb18 and Pb3 isolates, which represent two distant *Paracoccidioides* phylogenetic groups that evoke distinct patterns of experimental PCM. EV proteomic and lipidomic analyses of these isolates unravelled 205 proteins, phospholipids, sterols and glycolipids. The present work aimed at detecting and characterising EV RNA in Pb18, Pb3 and Pb01, for which full genome information is available. *Material and methods*: EV from cell-free supernatants of yeast cultures were concentrated and pelleted for 1 h at 100,000*g*. RNA was isolated using miRNeasy and RNeasy MinElute Cleanup (Qiagen). RNA sequencing was performed by next generation technology (SOLiD) and data were analysed by CLC Genomics Workbench software. *Results*: We found multiple and heterogeneous small and large RNA species. Validated sequences mapped mostly within exons, but there were minor sequences within intergenic and intronic regions. A total of 171 small RNAs from Pb18, Pb3 and Pb01 mapped within transcripts, 42% of which referred to translation, signalling, transport, nucleotide metabolism or were nuclear proteins. All validated *Paracoccidioides* small RNA sequences were compared with a specific microRNA bank, yielding 66 matches, among which 14 were over-expressed in highly virulent Pb18. They include mir-125 and mir-466 that can apparently interfere with the immune system homeostasis. The large RNA vesicular content included functional mRNA related with Ras/Rho GTPase family, as verified by sequencing in vitro translated proteins. *Conclusions*: Our results indicate that *Paracoccidioides* extracellular vesicles carry small and large RNA potentially capable of interacting with other cells.

Funded by: FAPESP, CNPq, Capes

**Cargo selection in bacterial outer membrane vesicles**

W.E. Elhenawy, M.F. Haurat, M.O. Debelyy and M.F. Feldman

Department of biological sciences, University of Alberta, Canada

*Introduction*: Outer membrane vesicles (OMV) are small blebs originating from the outer membrane of Gram-negative bacteria. Being released from the outer membrane (OM), both OM and OMV are similar in their basic structure which consists mainly of lipopolysaccharide (LPS), outer membrane proteins and phospholipids. OMV can act as long distance delivery tools by which bacteria can transfer soluble and non-soluble cargo into the host cells. OMV play a role in pathogenesis as they can carry virulence factors to the host cells. Moreover, OMV were suggested to be involved in biofilm formation, DNA transfer and, recently, in stress response. Despite the different roles suggested for vesicles, whether OMV are generated by an active process or by simple disintegration of the OM remains to be determined. In our lab we used a proteomic approach to analyse the protein and LPS content of OM and OMV in two species of Gram-negative bacteria, *Porphyromonas gingivalis* (dental pathogen) and *Bacteroides fragilis* (important member of the gut microbiota). *Materials and methods*: OM and OMV proteins of *P. gingivalis* and *B. fragilis* were purified and separated by SDS-PAGE. Protein bands were cut and trypsin digested. The resulting peptides were purified using ZipTip C18 then analysed via LC-ESI-MS/MS followed by protein identification with Mascot search engine. *Results*: OM and OMV exhibited different compositions. A subset of proteins appeared to be selectively packed in OMV while the rest of OM proteins were excluded from the vesicles. In *P. gingivalis*, OMV were enriched in proteases while in *B. fragilis* different proteases, glucosidases and galactosidases were found in OMV. *Conclusions*: Bacteria are able to pack proteins in vesicles selectively. The packed OMV proteins include hydrolases that might contribute to pathogenesis, interbacterial competition or nutrient acquisition. Cargo selection provides evidence that vesiculation is a directed process in bacteria.

**Proteomic analysis of membrane-associated fraction and enriched outer membrane vesicles fractions from multidrug-resistant Acinetobacter baumannii DU202**

G.H. Kim, S.H. Yun, Y.G. Lee, Y.H. Hong, E.C. Park, C.W. Choi, Y.J. Lee and S.I. Kim

Korea Basic Science Institute/Division of Life Science, Republic of Korea

*Introduction*: *Acinetobacter baumannii* is an important nosocomial pathogen, particularly in intensive care units of the hospitals. Multidrug-resistant (MDR) *A. baumannii* is difficult to treat with antibiotics, and treatment failure in infected patients is of great concern in clinical settings. Outer membrane vesicles (OMVs) are used by bacteria in a secretion mechanism that leads to the delivery of various bacterial proteins and lipids into host cells, thus eliminating the need for bacterial contact with the host cell. MDR *A. baumannii* is also known to produce OMVs. *Material and methods*: To investigate proteome regulation in *A. baumannii* under antibiotic stress conditions and relative proteomic analyses of a clinical MDR strain *A. baumannii* DU202 cultured in subminimal inhibitory concentrations of tetracycline and imipenem. Preparation of membrane-associated fractions was performed according to modified methods by sodium carbonate preparation methods. OMV fractions was separated using ultracentrifuge with the Quix-stand. Comparative proteomic analysis of membrane fraction and OMV fraction are used by 1DE-LC-MS/MS proteomic approaches. *Result*: Proteomic analysis of OMV showed outer membrane proteins (Omp38, OmpH, etc.), putative glucose-sensitive porin (OprB-like), putative outer membrane protein and TonB-dependent siderophore receptor as major outer membrane proteins of OMV of *A. baumannii* DU202. In total, 248 (LB), 264 (tetracycline) proteins were identified and 221 (imipenem) were classified as outer membrane, periplasmic or plasma membrane proteins. *Conclusions*: Through this study, we will have a better understanding of resistant mechanisms of MDR *A. baumannii* to active antibiotics in a proteomic perspective.

**Evolutionary analysis of proteomes in bacteria-derived extracellular vesicles reveals universal signatures of common vesicular proteins**

D.K. Kim ^1^, J. Lötvall^2^, D. Hwang^1^, Y.K. Kim^1^ and Y.S. Gho^1^

^1^Pohang University of Science and Technology, Republic of Korea; ^2^University of Gothenburg, Sweden

*Introduction*: The secretion of extracellular vesicles is a common cellular activity observable not only in simple unicellular organisms (e.g. archaea, Gram-positive and Gram-negative bacteria) but also in complex multicellular ones. This suggests that the extracellular vesicle-mediated communication is evolutionarily conserved. However, molecule-based evolutionary study on vesicular proteins has not been performed. *Material and methods*: Using bacteria-derived vesicular proteomes catalogued in EVpedia, we performed orthologue identification by OrthoMCL. By orthologue groups, we defined conservation score of each vesicular protein as the number of its orthologue appearances in the vesicular proteomes, and classified the vesicular proteins with their conservation score. Gene Ontology analysis and network analysis were performed on each category. *Results*: Using EVpedia (http://evpedia.info), a comprehensive database of high-throughput datasets from prokaryotic and eukaryotic extracellular vesicles, we compared vesicular proteomes derived from different bacterial strains to identify the common vesicular proteins. We observed that common vesicular proteins were enriched in the outer membrane and in the cytoplasm although bacteria-specific proteins were enriched in the outer membrane and in the periplasmic space. Moreover, the common vesicular proteins represented enriched biological processes, which identified universal functions of bacteria-derived extracellular vesicles from different strains such as the primary cellular processes, metabolic processes, survival related processes and outer membrane assembly. The interactions of the common vesicular proteins were used to reconstruct a network delineating the conserved cellular processes and their relationships. *Conclusions*: Our evolutionary analysis of bacterial proteomes reveals that bacteria-derived extracellular vesicles harbour universal signatures of common proteins that should be involved in common biological functions.

**The stress inducible protein 1 is released by melanoma cell lines via extracellular vesicles**

Marcos Dias, B.C. Silva, J.R.B. Batista and V.R. Martins

AC Camargo Hospital, Brazil

*Introduction*: The co-chaperone STI1 (stress-inducible protein 1) was characterised as a prion protein (PrPC)-specific ligand. STI1 and PrPC are expressed in various tumours and it is of great importance to determine their role in tumour processes. STI1 is secreted to the extracellular milieu and binds to PrPC in the cell membrane modulating cellular functions such as proliferation. However, STI1 lacks a signal peptide for secretion and the mechanisms modulating its secretion are unknown. The goal of this study was to explore whether STI1 is secreted in extracellular vesicles (EVs) in melanoma cells. *Material and methods*: Conditioned medium (CM) derived from the B16F1 (low metastatic variant) and B16F10 (high metastatic variant) murine melanoma cell lines was submitted to classical centrifugation protocols to isolate EVs. Size and concentration of EVs were evaluated using nanoparticle tracking analysis (NTA) and transmission electron microscopy. The expression of STI1 on EVs released in CM from both cell lines was examined by western blot and ELISA. Flow cytometry assays were performed to assess the localisation of STI1 in EVs. *Results*: Our data showed that both cell lines secreted EVs with a median diameter of 100–200 nm and that B16F10 releases a two-fold higher concentration of EVs than B16F1. SDS-PAGE silver-staining approaches demonstrated that B16F10 appears to secrete larger amounts of protein in EVs than B16F1. Also, B16F10 EVs have higher amount of STI1 than B16F1. Flow cytometry analysis showed positive staining for STI1, as well as for PrPC and flotillin (surface markers), indicating that at least part of the STI1 was present at the outer leaflet of MVs. *Conclusions*: These results suggest that STI1 is released by melanoma cell lines associated to the surface of EVs and that this protein is secreted in higher levels by the more metastatic cell line than by the lower metastatic variant.

Funded by: Sao Paulo State Foundation (FAPESP).

**Golgi reassembly and stacking protein is involved in the vesicular traffic of polysaccharides in the fungal pathogen *Cryptococcus neoformans***

L.S. Joffe ^1^, J.A. Rizzo^1^, L. Kmetzsch^2^, C. Staats^2^, C.L. Ramos^1^, K. Miranda^1^, S. Frases^1^, M.H. Vainstein^2^ and M.L. Rodrigues^3^

^1^UFRJ, Brazil; ^2^UFRGS, Brazil; ^3^UFRJ/FIOCRUZ, Brazil

We have recently described that Golgi reassembly and stacking protein (GRASP) is required for polysaccharide secretion and virulence in the encapsulated fungal pathogen *Cryptococcus neoformans*, the etiologic agent of human cryptococcosis. We have also observed in previous studies that secretion of capsular polysaccharides in *C. neoformans* involves extracellular vesicle (EV) formation. Since GRASP is potentially involved in vesicular secretion in other eukaryotes, we hypothesised that EV-mediated polysaccharide export and GRASP are functionally connected in *C. neoformans*. Wild-type *C. neoformans* cells and a mutant lacking GRASP expression (▵grasp) had comparable levels of EV formation. However, polysaccharide concentration in EVs produced by the grasp mutant was decreased, in comparison with WT cells. Vesicular polysaccharide fibres produced by the grasp mutant also showed reduced dimensions, as determined by dynamic light scattering. EVs obtained from cultures of the grasp mutant also differed from those produced by WT cells in diameter distribution. Analysis of the *C. neoformans* surface architecture by scanning electron microscopy revealed that, in comparison to WT cells, the mutant had capsular polysaccharide fibres with reduced dimensions. These results were suggestive of defects in polysaccharide traffic. We also evaluated whether lack of GRASP would interfere with polysaccharide synthesis in *C. neoformans*. Analysis of crude cellular extracts revealed that the grasp mutant was in fact less efficient than WT cells in synthesising capsular polysaccharides. These results demonstrated that GRASP is required for both polysaccharide synthesis and EV-mediated traffic in *C. neoformans*. Since polysaccharides are determinant for virulence in this fungus, we conclude that EV formation, GRASP and pathogenesis are directly connected in the *C. neoformans* model.

**Physiological and proteomic characterisation of outer membrane vesicle of *Pseudomonas putida* KT2440**

C.W. Choi, S.H. Yun, Y.G. Lee, Y.J. Lee, G.H. Kim, Y.H. Hong, E.C. Park, S.I. Kim

Korea Basic Science Institute/Division of Life Science, Republic of Korea

*Introduction*: Outer membrane vesicles (OMVs) have been found from various pathogenic gram-negative bacteria such as *Escherichia coli, Pseudomonas aeruginosa and Acinetobacter baumannii*. In this study, we isolated OMV from a representative soil bacterium, *Pseudomonas putida* KT2440, which has biodegradability to various aromatic compounds. *Materials and methods*: *P. putida* KT2440 was obtained from ATCC. Bacterial cultivation was performed according to previous methods (Yun et al. 2011). OMVs of *P. putida* KT2440 were purified from the bacterial culture supernatants by using a modified method from Kwon et al. LC-MS/MS analysis was performed according to a previous method (Park et al. 2006). *Results*: Proteomic analysis of OMV showed outer membrane proteins (OmpF, OmpW, et al), TonB-dependent receptors, outer membrane ferric siderophore receptor, and 17 kDa surface antigen as major outer membrane proteins of OMV of *P. putida* KT2440. OMV of *P. putida* KT2440 showed pathological effect on the culture cells originated from human lung cell. Production and its protein components of OMV were dependable to the carbon sources of culture media. Production of OMV was significantly increased in rich medium (LB) compared with in minimal medium containing benzoate and succinate as sole carbon sources, respectively. However, porins (benzoate-specific porin, BenF-like porin, et al.) and enzymes (catechol 1,2-dioxygenase, benzoate dioxygenase, et al.) for benzoate degradation were uniquely found in OMV prepared from *P. putida* KT2440 cultured in benzoate. *Conclusions*: This result suggests that production and components of OMV of *P. putida* KT2440 were controlled by the environmental stimulus or nutrients.

**Poster Session II (Statler): Proteomics April 17**

**Chair: *S. Mathivanan and Y.S. Gho***

**Proteomic analysis of exosomes enriched using protein microarray**

A. Stensballe ^1^, M. Jørgensen^2^. R. Bæk^2^ and K. Varming^2^

^1^Section of Biotechnology, Aalborg University, Aalborg, Denmark; ^2^Department of Clinical Immunology, Aalborg University Hospital, Aalborg, Denmark

*Introduction*: Functional analysis of biological classes of extracellular microvesicles (MV) necessitates efficient means for purification of enough biological material needed for subsequent detailed analysis. Exosomes are endosome-derived vesicles between 40 and 100 nm in diameter that are secreted by various cell types. The aim of this study was to investigate the applicability for mass spectrometry-based subproteome analysis of the novel multiplexed platform of protein microarray used for capturing exosomes of different biological origins including plasma from human donors, stable isotope-labelled cancer cell cultures and synovial fluid. *Materials and methods*: A novel protein array based on biotin-labelled anti-tetraspanin (CD9, CD63 and CD81) antibodies specific for exosomes in general enabled capture of exosomes from crude human donor plasma and MV preparations from stable isotope-labelled cancer cells and synovial fluid. A range of elution conditions and sample preparation methods prior to high performance mass spectrometry based protein identification (UPLC-Orbitrap tandem MS) and bioinformatics analysis. *Results and conclusion*: Protein microarray-aided enrichment enables profiling and enrichment of exosomes from minute amounts of starting material. A biophysical validation confirmed that the protein microarray mainly captured microparticles below 100 nm in size; thus, the protein microarray is a well-functioning new method for exosome characterisation and phenotyping using only small amounts of starting material. Our optimisation of washing and conditions for exosome elution enabled recovery of enriched exosomes. Preliminary data confirms an elution and up concentration of exosomes enabling the unambiguously profiling of exosomal proteins from a broad range of biological samples. Our findings conclude that state-of-the-art mass spectrometry enables exosomal profiling enriched from multiple parallel enrichment protein arrays.

**Quantitative proteomics of fractionated membrane and lumen exosome proteins from isogenic metastatic and non-metastatic bladder cancer cells**

D.K. Jeppesen ^1^, A. Nawrocki^2^, K. Thorsen^1^, S.G. Jensen^1^, B. Whitehead^3^, K.A. Howard^3^, L. Dyrskjøt^1^, T.F. Ørntoft^1^, M.R. Larsen^2^ and M.S. Ostenfeld^1^

^1^Department of Molecular Medicine, Aarhus University Hospital, Skejby, Denmark; ^2^Department of Biochemistry and Molecular Biology, University of Southern Denmark, Denmark; ^3^The interdisciplinary Nanoscience Center (iNANO), University of Aarhus, Denmark

*Introduction*: Exosomes are small extracellular membrane vesicles present in biological fluids. They are secreted by various cell types and feature a rich array of membrane and luminal proteins. Cancer and stromal cells release exosomes to both the cancer microenvironment and to circulation, where they may facilitate cancer progression and metastasis. *Material and methods*: Here we present the first quantitative proteomic analysis of exosomes fractionated by membrane or luminal localisation, from isogenic bladder cancer cells with different metastatic potential. By using the CLAD1000 bioreactor system, an increased yield of exosomes was obtained by ultracentrifugation from human bladder carcinoma cell line T24 without metastatic capacity, and its two isogenic derivate cell lines SLT4 and FL3, which form metastases predominantly in the liver and lungs of mice, respectively. An iTRAQ proteomic approach was used to quantify the abundance of exosomal proteins. *Results*: A greater release of exosomal protein was observed from metastatic FL3 cells versus non-metastatic T24 cells. Successful separation of exosomal proteins into membrane and luminal fractions was verified by immunoblotting for the membrane proteins Alix, CD9 and CD63, and the luminal protein calpain. We have identified and quantified exosomal proteins in the membrane and luminal fractions that display significantly altered abundance in the metastatic exosomes compared to non-metastatic exosomes. Ingenuity pathway analysis of these proteins revealed significant enrichment for proteins associated with cell-to-cell signalling and interaction, and cellular movement in exosomes with metastatic potential. *Conclusions*: We have developed a new strategy of fractionation for quantitative proteomics of exosomal membrane and luminal proteins. The abundance of specific proteins is altered in metastatic versus non-metastatic exosomes from isogenic bladder carcinoma cells.

**Human prostasomes express glycolytic enzymes with capacity for ATP production**

Göran Ronquist, B. Ek, A. Stavreus-Evers, A. Larsson, G. Ronquist

Uppsala University, Sweden

*Introduction*: Prostasomes transmit signalling complexes between acinar epithelial cells of the prostate and sperm cells. Most prostasomes have a diameter of 30–200 nm with a surrounding membrane bilayer. Using a selected proteomic approach, it became increasingly clear that prostasomes harbour distinct subsets of proteins that may be linked to adenosine triphosphate (ATP) metabolic turnover that in turn might be of importance in the role of prostasomes as auxiliary instruments in the fertilisation process. *Methods*: Prostasomes were purified from human seminal plasma by differential centrifugation steps including preparative ultracentrifugation and gel chromatography. Pooled elution of prostasomes was top-loaded on a sucrose gradient and the band on 1.5 M sucrose was collected and prostasomes were concentrated to 2 mg/mL by a BCL kit. For evaluation of ATP produced by incubation of prostasomes, a luciferase-based ATP estimation kit (InVitrogen) was used. Glucose, fructose and glyceraldehyde 3-phosphate were used as energy-yielding substrates in incubation reactions. *Results*: The identified enzymes in their prostasomal context were operational for ATP formation when supplied with substrates. The net ATP production was low due to a high concomitant prostasomal ATPase activity that could be partially inhibited by vanadate that was utilised in order to profile the ATP forming ability of prostasomes. Glucose and fructose were equivalent as glycolytic substrates and the enzymes involved were apparently surface-located on prostasomes, since an alternative substrate not being membrane-permeable (glyceraldehyde 3-phosphate) was operative, too. Prostasomal adenylate kinase-catalysed ATP formation was additional to glycolytic reactions. *Conclusions*: Prostasomes have the capacity for extracellular ATP formation. The exact role of this ATP-forming ability of prostasomes is not known, but extracellular ATP may be an important substrate for diverse protein kinase reactions.

**Molecular lipidomics analysis of exosomes released by PC-3 prostate cancer cells: implications for exosome membrane structure**

A. Llorente ^1^, T. Skotland^1^, T. Sylvänne^2^, D. Kauhanen^2^, K. Ekroos^2^ and K. Sandvig^3^

^1^Oslo University Hospital, The Norwegian Radium Hospital, Norway; ^2^Zora Biosciences Oy, Finland; ^3^Oslo University Hospital-The Norwegian Radium Hospital, Norway

*Introduction*: In contrast to the extensive information about the protein composition of exosomes, not much is known about their lipid composition. We believe that the lipid composition, alone and collectively with the protein composition, holds key answers in the exosome biology. Therefore, we performed a study aimed at obtaining the most detailed lipid composition of exosomes to date. *Materials and methods*: Exosomes were isolated from the conditioned media of PC-3 prostate cancer cells by ultracentrifugation and characterised by electron microscopy and western blot. Sophisticated shotgun- and targeted lipidomic assays were assessed for in-depth analysis of the lipidomes of PC-3 cells and exosomes. *Results*: More than 200 molecular lipid species from 22 lipid classes were quantified in exosomes released from the prostate cancer cell line PC-3. The analysis revealed an 8.4-fold enrichment of lipids per milligram of protein in the released vesicles compared to the parent cells. Furthermore, exosomes were highly enriched in glycosphingolipids, sphingomyelin and cholesterol and contained more saturated phospholipids and less monounsaturated phospholipids than the parent cells. *Conclusions*: There is a remarkable selectivity of lipid species enriched in exosomes. This selectivity is observed even in classes not enriched in exosomes.

**Proteome analysis of multidrug-resistant, breast cancer-derived microparticles**

D. Pokharel ^1^, M.P. Padula^2^, J. Tacchi^2^, F. Luk^1^, G.E.R. Grau^3^ and M. Bebawy^1^

^1^School of Pharmacy, The University of Technology, Sydney, Australia; ^2^The ithree Institute, The University of Technology, Sydney, Australia; ^3^School of Medical Sciences, University of Sydney, Australia

*Introduction*: Cancer multidrug resistance (MDR) occurs when cancer cells decrease drug influx or increase drug efflux and in doing so survive the effects of anticancer drugs. Previously, we have demonstrated that breast cancer cells spontaneously shed microparticles (MPs) from their surface and can transfer cell surface resistance proteins from resistant cells to drug-responsive cells and confer MDR in a few hours. This study aims to characterise the proteome of drug-resistant and drug-resistive breast cancer cells and their MPs in order to understand the molecular basis for the acquisition and dominance of the donor cell trait by MPs. *Materials and methods*: MPs were isolated from the drug-sensitive and drug-resistant human breast adenocarcinoma cells, MCF-7 and MCF-7/DX cells respectively as previously described (1). Samples were sonicated, alkylated, reduced, ran on the 1D-SDS PAGE, trypsinised and analysed by mass spectrometry (MS). The MS data was evaluated with the help of Mascot software (www.matrixscience.com). *Results*: We detected approximately 200 unique proteins per MP sample. Of these, the MP proteins from the drug-sensitive parental cells differed by 70% when compared to the drug-resistant cells. Of particular interest to us are those proteins that may be implicated in facilitating the transfer and insertion of multidrug resistance protein into recipient cells via this pathway, including proteins involved in cell adhesion. *Conclusion*: We describe the first comparative proteome analysis of MPs isolated from drug-sensitive versus drug-resistant human breast cancer cells. We observe selective packaging of proteins depending on the drug sensitivity of the donor cell.

Funded by: National Health and Medical Research Council (1007613)

Reference

1. Bebawy et al. Membrane microparticles mediate transfer of P-glycoprotein to drug sensitive cancer cells. 2009.

**Characterisation of miRNA carried by exosomes in human semen**

Lucia Vojtech ^1^, S.M. Hughes^1^, J.R. Chevillet^2^, M. Tewari^2^ and F. Hladik^1^

^1^University of Washington, USA; ^2^Fred Hutchinson Cancer Research Center, USA

*Introduction*: It has been established that factors in semen directly influence early events in viral transmission and the immune response in the genital mucosa, but which components are responsible is not well understood. Our finding that trillions of microvesicles are secreted into each ejaculate led us to explore the exosomal compartment of semen as a source of immunoregulation in the recipient mucosa. *Material and methods*: Seminal exosomes (SE) were isolated from seminal plasma. Entry of SE into antigen-presenting cells (APCs) was tested by exposing vaginal Langerhans cells to labelled SE. Exosomal RNA was isolated and confirmed as exosome-associated by RNAse/proteinase protection assays. In 6 separate semen donors, size-selected RNA from 10 to 40 nucleotides and 50 to 110 nucleotides was sequenced. Sequencing results were compared to miScript RT-PCR array analysis of 24 abundant miRNAs in exosomes from 4 additional samples. Select miRNA target sequences were cloned into the pMIR-glo vector and transfected into THP-1 cells to test delivery of functional miRNAs by seminal exosomes. *Results*: Ejaculates contained on average 2.2×1013 exosomes (2.25×10^12^–9.4×10^13^), which rapidly entered LCs. SE carried a mean of 1477 ng RNA (210–4800 ng), with at least 229 different miRNAs. The miRNA content of the 6 biological replicates was highly correlated (mean sequencing count Pearson r^2^=0.933). miRNAs were present both in mature and precursor (pre-miRNA) forms. For many of the most abundant miRNAs in seminal exosomes (e.g. let-7b, miR-375), immunomodulatory activities have been reported or predicted. Using the THP1 monocytic cell line transfected with miRNA target reporter plasmids, we are currently testing whether SE can deliver functional miRNAs to APCs in sufficient quantities to regulate target mRNAs. *Conclusions*: Exosomes in semen carry specific miRNAs with high biological reproducibility and rapidly enter APCs, likely resulting in immunomodulatory effects in the genital mucosa.

**The over-expression of a single oncogene alters the proteomic landscape of microparticles**

M.G. Amorim ^1^, D. Martins-de-Souza^2^, E. Dias-Neto^1^ and D.N. Nunes^1^

^1^Medical Genomics Lab, AC Camargo Hospital, Brazil; ^2^Max Planck Inst. & Ludwig-Maximilians-Universität, Germany

*Introduction*: Content differences may confer distinct biological functions to heterogeneous microparticle (MP) populations. The over-expression of one oncogene dramatically alters cell phenotype, but it is unknown whether it affects MP content. *Materials and methods*: MPs were isolated from conditioned medium of HB4a, a human mammary luminal epithelial cell line, and C5.2, a transformed HB4a clone over-expressing HER2, by differential sedimentation at 20K*g* and 100K*g*. MPs were characterised by transmission electron microscopy and proteomic analysis. SDS-PAGE was used for protein prefractionation prior to nanoflow liquid chromatography-mass spectrometry (nLC-MS/MS) and label-free (MPs) or isotope-coded protein labelling (cells) methods were used for proteome analysis. Experiments were performed in duplicates and only proteins identified by at least two peptides with 95% confidence were considered. *Results*: Proteomic data revealed high HER2 levels in C5.2 cells (>3000×) and in both C5.2-derived MPs, and allowed the quantitation of 589 (20k), 421 (100k) and 1468 (cells) proteins. 122 proteins were exclusively found in 20k MPs, and 60 proteins were exclusive to 100k MPs. HER2 over-expression resulted in the upregulation of 17 and 13% and the downregulation of 7 and 13% of the proteins (20k and 100k MPs, respectively). A high level of protein heterogeneity was seen between the MP subsets. Only 2/103 and 13/140 proteins increased in HB4- and C5.2-MPs, respectively, were seen in both MP subsets. Proteins capable of inducing malignant transformation were found in both MP subsets including two proteins involved in cell motility and invasion, cofilin and CD44, both increased >90×. *Conclusions*: MP subsets differentially shuttle many proteins, suggesting that their content is not arbitrarily defined and probably reflects distinct functions. HER2-upregulated proteins in MPs may drive cellular malignancy and are potential biomarkers for HER2+ cancer patients.

Funded by: FAPESP

**Exosomal microRNAs: export mechanisms and roles in hepatocellular carcinoma metastasis**

Maja Janas, David Morrissey and Lakshmi Raj

Novartis, USA

MicroRNAs (miRNAs) appear to be selectively sorted into exosomes by unknown mechanisms. In contrast to mRNAs and proteins, a single miRNA can regulate complex phenotypes by repressing hundreds of genes when delivered to a recipient cell. Indeed, exosomal miRNA secretion and uptake have been shown to affect multiple physiological processes as well as tumourigenesis. To understand the mechanisms and biological significance of selective miRNA export via exosomes and the role in hepatocellular carcinoma (HCC) metastasis, we performed deep sequencing of precursor and mature miRNAs across 8 HCC cell lines with different metastatic potential. We identified miR-21 as the most highly enriched miRNA in metastatic SK-HEP-1 exosomes but not in non-metastatic Huh-7 exosomes. Other miRNAs present exclusively in SK-HEP-1 exosomes included miR-378, miR-30a, miR-10a and let-7i, while miRNAs present exclusively in Huh-7 exosomes included miR-192, miR-191, miR-27b and miR-101. Moreover, miR-486-5p and miR-182 were commonly enriched in exosomes from both cell lines. These selectively exported miRNAs will be used as reporters in an RNAi screen for factors regulating the exosomal miRNA pathway using an HCC donor and receptor cell line system. In parallel, we have undertaken a proteomic approach to identify proteins associated with miRNAs in exosomes using quantitative mass spectrometry. These studies will help define mechanisms underlying selective exosomal miRNA secretion and will assess in vivo roles of exosomal miRNAs and factors required for their export in metastasis in an orthotopic HCC mouse model.

**Proteomic analysis of microparticles released from endothelial cells treated with vascular risk factors**

Ji Chen, X. Xiao, S. Chen, W.C. Grunwald Jr, S. Gu, C. Zhang, D.R. Cool and Y. Chen

Wright State University, USA

*Introduction*: Microparticles (MPs) might represent a novel network for intercellular communication since they convey the contents and characters from their parent cells and merge with contacted cells. Our previous study showed that pre-incubation with circulating MPs isolated from db/db diabetic mice induces dysfunction to endothelial progenitor cells and microvascular endothelial cell (ECs). This study was designed to investigate the underlying mechanism by analysing the protein profile of MPs released from the cECs treated with various vascular risk factors. *Material and methods*: Primary human microvascular ECs (CSC 2M1) bought from cell system (Kirkland, WA, USA) were used for the study. The cultured ECs were treated with high glucose (HG, 25 mmol/L), oxidised low density lipoprotein (ox-LDL, 50 μg/ml), HG plus ox-LDL and vehicle (culture medium) for 48 h. Then, the MPs released into supernatants were isolated by multiple centrifugations and the ECs were harvested, followed by protein extraction with lysis buffer plus protease inhibitors. The proteins were subjected to SDS-PAGE electrophoresis and the protein bands were cut off for in-gel digestion of protein. The resulting proteins were analysed by mass spectrometry on a Bruker Autoflex III MALDI TOF/TOF. *Results*: (1) On SDS-PAGE, the patterns of protein bands in EC-released MPs and ECs were different among different groups; (2) MS analysis showed that the protein profile of ECs was also different from MPs; (3) Interestingly, preliminary MS/MS analysis and database search found that HG plus ox-LDL treatment induced expression of alpha-2-macroglobulin isoform-1, alpha-1-inhibitor III precursor, pregnancy zone protein in ECs. *Conclusion*: This study demonstrates that various vascular risk factors affect protein expression in ECs and their released PMs, which could have clinical implications in EC function, vascular disease and Alzheimer's disease.

**Circulating microparticles carry high procoagulant activity in patients with essential thrombocythemia**

Marina Marchetti ^1^, C.J. Tartari^1^, L. Russo^1^, M. Panova-Noeva^1^, A. Leuzzi^1^, A. Rambaldi^1^, G. Finazzi^1^, B. Woodhams^2^ and A. Falanga^1^

^1^Ospedale Papa Giovanni XXIII, Italy; ^2^Stago R&D, France

This study evaluates the functional procoagulant features of plasma MP to explore their contribution to the hypercoagulable state commonly found in essential thrombocythemia (ET). Platelet-free plasma samples were obtained from 72 ET patients (37 positive for JAK2V617F mutation) and 73 control subjects. The calibrated automated thrombogram (CAT) was performed in plasma samples to determine thrombin generation of MP-associated tissue factor (TF) and procoagulant phospholipid (PPL) activity, and the STA Procoag PPL assay to measure MP-PPL activity only. Both thrombin generation and PPL procoagulant activities were found significantly elevated in ET patients compared to controls and were associated to significantly higher levels of TF antigen and FVIIa/AT complex. Thrombin generation was significantly greater in JAK2-V617F-positive compared to JAK2-V617F-negative patients and normal subjects. Significant correlations were found between the PPL assay and the different parameters of the CAT assay. No difference was seen between the thrombosis and no thrombosis group. Prospective studies are needed to test whether MP-associated thrombin generation and procoagulant activity may predict for thrombosis in these patients.

**Properties of platelet-derived microvesicles depend on the activation pathway**

M. Aatonen, M. Grönholm, D. Lopez-Contrarez and P.R.M. Siljander

Division of Biochemistry and Biotechnology, Department of Biosciences, University of Helsinki, Finland

*Introduction*: Platelets release both plasma membrane-derived microparticles (PMPs) and exosomes. Changes in in vivo microvesicle (PMV) levels correlate with various physiological and pathological states. Therefore, we have systematically compared PMVs generated by different platelet-activating pathways. *Materials and methods*: Platelets were isolated from healthy volunteers with modified-gradient procedure to improve sample purity and activated with physiologically relevant agonists and Ca2 + -ionophore A23187. Enriched PMP and exosome populations were isolated with differential centrifugations. MV number and size distribution were analysed by nanoparticle tracking analysis (NTA) and correlated with total protein, lipids and EM, respectively. Pooled samples from 6 donors were analysed with LTQ-Orbitrap-XL mass spectrometry. *Results*: Size distribution data (NTA,EM) showed that 64–85% of MVs were <250 nm depending on activation and 95–99% were <500 nm. Only 0.5–5% of PMVs was in the 0.5–1 µm range. PMVs induced by thrombogenic/immunological or A23187 pathways showed significant differences in the total protein and the lipid amounts and the proteomic cargo. Proteomic analysis of PMVs identified proteins of cytoplasmic (47%), surface (16%), organelle (15%) and unknown (14%) origins and 8% as secreted proteins. From 537 total proteins, 12 proteins were shared by all the exosome and PMP conditions, including CD9 and α-IIb integrin. Sixty percent of proteins were unique to each condition, whereas multimerin-1 was found in all exosomes but not in PMPs. *Conclusions*: Our data show that the activation pathway significantly modulates the quantity and the molecular composition (protein/lipid) of the subsequently formed PMVs, but not so much their size distribution. The scarcity of PMVs in the 0.5–1 µm range strongly urges to re-evaluate the data obtained with 1st generation flow cytometers. Agonist-specific proteomic profiles suggest that different PMVs should be individually tested in cell biological assays to reveal the physiological and pathological significance of PMVs.

**Human albumin reduces microparticle generation during blood storage**

Ionita Ghiran, M. Lawler, A. Glodek, A. Nicholson-Weller and P. Akuthota

BIDMC, USA

*Introduction*: During blood storage, red blood cells (RBC) undergo loss of RBC functions (deformability), loss of RBC structure (formation of microparticles) and increased release of inflammatory mediators such as ATP. RBC-derived microparticles are a significant part of the post-transfusion inflammatory response that leads to acute lung injury and decreased survival. Herein, we report an improved method for blood storage that prevents the formation microparticles during blood storage`. *Material and methods*: Membrane deformability of RBC was assessed using a 2-D microfluidic device. ROS generation and cell surface protein composition were measured in parallel by flow cytometry and western blotting. Microparticle isolation was performed using ultra-centrifugation. *Results*: Our data show that the deformability of stored RBC can be separated in 3 distinct populations: ~45% of the RBCs have transit times of 5.9±1.6 s, which is similar to fresh RBC. However, ~41% of RBC require ~3-fold longer transit times, while the remaining 9–16% RBC demonstrate markedly greater rigidity, requiring transit times >150 s. Next, we investigated the effect of microparticles on human neutrophils by co-incubating complement-opsonised microparticles with isolated neutrophils and measuring the cell surface levels of activated beta-2 integrins. Neutrophils co-incubated with complement-opsonised microparticles display increased levels of activated beta-2 integrins compared to neutrophils incubated with buffer alone or microparticle-free storage solution. Importantly, the addition of a low concentration of albumin during blood storage reduces microparticle production by over 90%. *Conclusions*: Our data suggest that the addition of a low concentration of albumin to blood storage solutions can significantly reduce the rate of microparticle generation and improve the hemodynamic properties of stored RBC, potentially reducing the detrimental effects of post-transfusion inflammatory response.

**Extracellular vesicles generated during apoptosis: does the size matter?**

B. Szántó^1^, K. Pálóczi^1^, A. Kittel^2^, K. Szabó-Taylor^1^, B. György^1^, T.G. Szabó^1^, A. Németh^1^, M. Szente-Pásztói^1^, M. Deli^3^, M. Csete^4^, A. Szalai^4^, A. Falus^1^ and W.I. Buzás ^1^

^1^Semmelweis University, Hungary; ^2^Institute of Experimental Medicine, Hungarian Academy of Sciences, Budapest, Hungary; ^3^Biological Research Centre of the Hungarian Academy of Sciences, Szeged, Hungary; ^4^University of Szeged, Hungary

*Introduction*: Extracellular vesicles released during apoptosis have received moderate attention over the past decades despite the fact that apoptosis is one of the major adaptive cell responses. *Materials and methods*: Here we investigated extracellular vesicles released by the CCRF CEM and U937 cell lines upon apoptosis induction by staurosporine and etoposide. Apoptosis and primary and secondary types of necrosis were assessed by flow cytometry in conditioned tissue culture supernatants. Fluorescence microscopy, transmission electron microscopy, atomic force microscopy and resistive pulse sensing (qNano) techniques were used to assess apoptotic cell-derived extracellular vesicles. *Results*: Both 2.5 µM staurosporin and 50 µM etoposide induced simultaneous release of both apoptotic microvesicles (<1 µm) and large apoptotic bodies (>1 µm). In contrast to other extracellular vesicles and platelets (both being sensitive to detergent lysis), in the case of apoptotic bodies and apoptotic microvesicles Triton X-100 only abolished plasma membrane annexin V binding, while forward and side scattering signals and PI staining were not abrogated. Furthermore, a higher proportion of large apoptotic bodies than apoptotic microvesicles showed increased membrane permeability to PI and was found positive for ER tracker staining. *Conclusions*: In contrast to earlier notions in the literature suggesting the preferential release of small size microvesicles at early stages of apoptosis, our work showed a continuous spectrum of vesicle sizes released throughout the course of apoptotic cell death. However, in spite of the size continuum of vesicles, structures below and above the arbitrary 1-µm size limit (used in the literature to distinguish microvesicles from apoptotic bodies) were found to be characterised by certain differential properties.

**Comprehensive glycan profiling of extracellular vesicles derived from lung cancer cell lines**

D.Y. Choi ^1^, J.H. Jung^1^, S.Y. Jeong^2^, H.J. An^2^ and K.P. Kim^1^

^1^Department of Molecular Biotechnology, Konkuk University, Seoul 143-701, Republic of Korea; ^2^GRAST, Chungnam national university, Daejeon, Republic of Korea

*Introduction*: Extracellular vesicles (EVs) are secreted by various cell types including tumour cells. They are composed of membrane, cellular proteins and RNA derived from their parent cells. They play an important role as mediators in extracellular communication. These biological functions may due to the glycosylation on membrane proteins. However, there is little study about glycosylations of EVs. In this study, we have profiled N-glycans of EVs derived from several lung cancer cell lines. We also compared glycans of EVs with glycans on their parent cell membranes to examine glycosylation correlation between origin cells and EVs. *Materials and methods*: Glycans from EVs were directly released by PNGase F without any further sample preparation. Released glycans were enriched by solid phase extraction (SPE) using a porous graphitised carbon (PGC) cartridge. The fractions were then analysed by two types of mass spectrometry. ultrafleXtreme MALDI-TOF/TOF MS (Bruker Daltonics) and Nano LC-chip Q-TOF MS (Agilent Technologies) were used for overall glycans profiling and quantitation, respectively. *Results*: We found that EVs contain over 75% of high-mannose glycans consisting of trimannosyl core (Man3 GlcNAc2) and [Man]2-6. The most abundant glycan is Man8 (Man8 GlcNAc2), while complex/hybrid type glycans with at least one fucose account for 25%. In order to study the differences of glycosylation between wild-type EVs and anticancer drug gefitinib (EGFR antagonist)-resistance type, we compared the glycans between PC9 and PC9/R. Total abundance of glycans on PC9 is slightly higher than that of PC9/R although the number of glycans analysed by LC/MS is quite similar. *Conclusions*: We will analyse bio- and analytical replica to confirm our preliminary data. In addition, we will profile N-glycans from more EVs derived from various cancer cell lines and their origin cancer cells, to examine the correlation of whether cancer cell lines and their EVs have similar glycan profile.

## Scientific Program 2013 ISEV meeting Thursday 18th April

**Plenary Oral Session 10 (Imperial Ballroom)**

**Chair: *W. Kuo and Y.S. Gho* 8:30-10:00**

8:30-9:30

Bioinformatics in the personal genome project and implications for exosome research

John Quackenbush

DFCI, USA

John Quackenbush is an American computational biologist and genome scientist. He is the Professor of Biostatistics and Computational Biology, Professor of Cancer Biology at the Dana-Farber Cancer Institute (DFCI), as well as the director of its Center for Cancer Computational Biology (CCCB). Quackenbush also holds an appointment as Professor of Computational Biology and Bioinformatics in the Department of Biostatistics at the Harvard School of Public Health (HSPH).

9:30-9:45

Protein budding occurs primarily at the plasma membrane

Stephen J. Gould and Jr-Ming Yang

Department of Biological Chemistry The Johns Hopkins University, Baltimore, MD 21205, USA

The biogenesis of secreted vesicles can potentially occur at any organelle of the exocytic and endocytic pathways – endoplasmic reticulum, Golgi apparatus, endosome or plasma membrane. Using a cargo-based approach, we investigated the relative contributions of these organelles to the budding of both acylated and integral membrane proteins. Our data indicate that the plasma membrane is the primary site of protein budding from animal cells, including human and fly cells. These results have important implications for both the basic cell biology of exosome and microvesicle biogenesis, as well as for the design and production of extracellular vesicles with specific biological functions.

9:45-10:00

To what do microparticles from blood plasma look like? Their morphology, size and phenotype revealed by cryo-EM and immuno-gold labelling

Alain Brisson, N. Arraud, R. Linares, S. Tan and C. Gounou

University of Bordeaux, France

Microparticles (MPs), also called extracellular vesicles, microvesicles, attract high interest as potential disease biomarkers; however, many basic questions concerning them remain unanswered, such as: To what do MPs from blood plasma look like? What is their morphology, their size distribution? Do they all bind Annexin-5 (Anx5)? How are they formed? How many are they? Our overall aim is to answer these questions, firstly to improve our basic understanding on MPs, secondly to design diagnosis assays on selected MP subpopulations. Our first objective was to provide a catalogue of all types of MPs present in plasma from healthy donors, considering this as a reference for further studies of MPs in various pathological conditions. We have developed an original approach based on cryo-transmission electron microscopy (cryo-EM) imaging and receptor-specific gold labelling. Cryo-EM allows revealing the various types of MPs present in pure platelet-free plasma (PFP). Gold nanoparticles functionalised with various ligands were developed for identifying specific MP subpopulations. We focused primarily on MPs exposing phosphatidylserine (PS), platelet-derived MPs and red blood cell-derived MPs, using gold particles functionalised with Anx5, anti-CD41 and anti-glycophorin antibodies, respectively. We show that PFP samples contain three main types of MPs: spherical vesicles, ranging from 50 to 500 nm, tubular vesicles, up to several micrometres long, and large cell fragments, from 2 to 10 µm. About 75% of MPs are smaller than 500 nm. Strikingly, only about 25% of MPs bind Anx5. We found also that about 20% of PFP MPs expose CD41, while most large cell fragments derive from red cells. Size histograms of the various MP subpopulations were determined, for the first time. The influence of aging, centrifugation and freezing on MP structure will be presented. This study provides novel basic knowledge on plasmatic MPs and will serve as a reference for further studies of MPs in disease.

**Coffee and Poster Viewing April 18**

**Poster Session III-IV 10:00-13:30**

**Parallel Oral Sessions 11-13 13:30-15:00**

**Oral Session 11 (Imperial Ballroom): Prokaryote to Eukaryote April 18**

**Chair: *Y.Y. Kim and D. Mulhall* 13:30-15:00**

Significance of history and nomenclature for extracellular vesicles, exosomes, matrix vesicles and microparticles

Douglas Mulhall

University of British Columbia, Canada

*Introduction*: Might a more inclusive and defined historical perspective improve research into extracellular vesicles (EV)? Clarifying early nomenclature is important for establishing an accurate historical perspective. *Methodological considerations*: In past decades due to technology limitations on morphology investigations, only basic terms were used, for example, “globules”, “platelet dust” and “elementary bodies”. Those terms are often excluded from present day EV literature searches and limit the scope of the early history. Some discovery dates are clear; for example, in 1969 Anderson and Bonucci each published descriptions of matrix vesicles in bone. Other discoveries are less defined for diverse EV types at diverse sites in diverse organisms. For example, in 1955 De Robertis and Bennett described vesicles of unknown origin in synaptic interspace in frog and earthworm, then recently Lachenal et al. observed synaptic vesicle-like exosomes released by neurons, apparently supporting the 1950s observation. Membrane-encased viruses described in the earlier 20th century were later identified by Gould as “trojan exosomes”, and viruses have emerged as important factors in the role of EV. Another historically significant nomenclature question: when is a vesicle extracellular? If EV enter cells are they then intercellular or intracellular? Early studies describe supposedly intracellular vesicles. Those might be the same EV which invade cells as described, for example, by Beveridge. As well, new references to EV as, for example, therapeutic adjuvants continue to broaden the nomenclature and require yet further historical investigation. *Conclusion*: The literature suggests EV were observed in more diverse environments than previously thought, possibly offering new evidence and investigative possibilities. Further investigation of early nomenclature and aligning it with expanding nomenclature today seem warranted to augment resources for research and development as well as for therapy.

**Proteomic characterisation of bacterial extracellular vesicles**

Yong Song Gho

POSTECH, Korea

In addition to mammalian cells, simple unicellular organisms (e.g. archaea, Gram-positive, and Gram-negative bacteria) also secrete nano-sized extracellular vesicles (EVs) for their intercellular communication. EVs derived from Gram-negative bacteria are called outer membrane vesicles (OMVs), whereas archaea and Gram-positive bacteria-derived EVs are called membrane vesicles. These membrane vesicles are spherical bilayered proteolipids carrying various bioactive molecules including proteins, genetic materials (RNAs and DNAs) and lipids. In this presentation, I will introduce the components and the high throughput proteomic studies of bacterial EVs. Moreover, current status and future direction on the patho/physiological functions of EVs in intercellular, interspecies and inter-kingdom communication will be covered.

**Exosomes are effectual vehicles for cell-to-cell bacterial protein toxins propagation**

C. Zanetti, A. Gallina, A. Fabbri, I. Parolini, Z. Boussadia, C. Coscia, M. Biffoni, A. Palermo, C. Fiorentini and M. Sargiacomo

Istituto Superiore di Sanità, Italy

*Introduction*: Among bacterial protein toxins, cholera toxin (CT) and cytotoxic necrotising factor 1 (CNF1) are well-known causative agents of specific signalling alterations in mammalian cells. Many physiological molecules have already been described to be incorporated and delivered by exosomes (exo) making them central in the transport in and out of the cells. Here we report, by strict methodological evidences, how exosomes serve as general mechanism for toxins to go through and spread from cell to cell. *Materials and methods*: Me665 human melanoma cells were metabolically labelled with a fluorescent C16 fatty acid to obtain exosomes containing fluorescent phospholipid (Fp-exo), that were purified by differential centrifugations, validated for number and fluorescence intensity by FACS, and for dimension with Nanosizer detector. Fp-exo transfer to cells was quantified by FACS. CT and CNF1 toxins, purified from *Vibrio cholerae* 569 B and *Escherichia coli* 392 ISS strains, were tested on cells: CT for morphological change and cAMP level, CNF1 for cytoskeleton modification and Rac GTPase activation by pull-down assay. Ab anti-CT was used to determine CT content in Fp-Exo by ELISA. *Results*: A fluorescent phospholipid-based methodology allowed us to estimate Fp-exo number, diameter and fluorescence intensity as well as to measure their transfer to cells. We found that Fp-exo purified from medium of Me665 cells incubated with a saturating amount of CT/CNF1 was loaded with toxins. In this context, we quantified the Fp-exo/CT ratio and the exo number per target cell apt to induce an increase in cAMP activity in a definite time. Hence, we estimated that the amount of CT carried by exo sufficient to achieve a raise of cAMP activity in a given number of cells is sensibly lower than CT alone. *Conclusions*: At least two toxins, belonging to a wide bacterial toxins family, appeared extremely sensitive biomarkers to enlighten exosome-mediated cell communication and homeostasis.

**Outer membrane vesicles-mediated release of protein from pathogenic bacteria**

S.N. Wai

Department of Molecular Biology, The Laboratory for Molecular Infection Medicine Sweden (MIMS), Sweden

*Introduction*: Outer membrane vesicles (OMVs) are constantly being discharged from the surface of the Gram-negative bacteria during growth. OMVs possess outer membrane proteins, LPS, phospholipids and some periplasmic constituents. Vesicles have been proposed to play a role in several contexts such as toxin delivery, DNA transport and evasion of the immune system. OMVs are able to fuse to target membranes and that small OMVs can serve as delivery “missiles” to host tissues. The mechanism(s) behind the ability of Gram-negative bacteria to export proteins via OMVs remains relatively unclear. *Materials and methods*: We use the molecular microbial pathogenesis approaches that studies OMVs from several different bacterial species in combination with nano-technological surface analyses of commensal and pathogenic variants of *Escherichia coli*. With the defined bacteria–host model systems we will aim at clarifying the “exosome-like” influence by bacterial OMVs both in case of pathogenic and commensal bacteria–host interactions. *Results*: In our recent studies, a cytolethal distending toxin (CDT), a genotoxin-associated OMV from *Aggregatibacter actinomycetemcomitans* was found to be internalised in the host cells via fusion with lipid rafts in the plasma membrane. In our current studies, we observed that OMVs from *Vibrio cholerae* adhere to and enter HeLa and HCT8 (human colon tumour) epithelial cells. We and others have now shown that OMVs contain peptidoglycan, which is sensed mainly by the pattern-recognition receptor NOD1 in the cytoplasm of host cells. *Conclusion*: OMVs release is promoting novel interactions between microbial cells and between eukaryotic host cells and microbes, including communication, release of antigens and secretion of virulence factors.

**Vesiculation from Pseudomonas aeruginosa under SOS**

Tao Wei ^1^, R. Maredia^2^, N. Devineni^2^, P. Lentz^1^, S.F. Dallo^1^, J.J. Yu^1^, N. Guentzel^1^, J. Chambers^1^, B. Arulanandam^1^ and W.E. Haskins^1^

^1^Biology Department. College of Science and Mathematics. Southwest Baptist University, USA; ^2^The University of Texas at San Antonio, USA

*Introduction*: Bacteria infections can be aggravated by antibiotic treatment that induces SOS response and vesiculation. This leads to a hypothesis concerning association of SOS with vesiculation. *Material and methods*: We conducted multiple analyses of outer membrane vesicles (OMVs) produced from the *Pseudomonas aeruginosa* wild type in which SOS is induced by ciprofloxacin and from the LexA non-cleavable (LexAN) strain in which SOS is repressed. The methodologies include microscopic analysis, OMV extraction and protein quantification, OMV lipid extraction and quantification, transmission electron microscope, macrophage cytotoxicity and MTT assay and OMV proteomic analysis. *Results*: The levels of OMV proteins, lipids and cytotoxicity increased for both the treated strains, demonstrating vesiculation stimulation by the antibiotic treatment. However, the further increase was suppressed in the lexAN strains, suggesting the SOS involvement. Obviously, the stimulated vesiculation is attributed by both SOS-related and -unrelated factors. OMV subproteomic analysis was performed to examine these factors, which reflected the OMV-mediated cytotoxicity and the physiology of the vesiculating cells under treatment and SOS. *Conclusions*: SOS plays a role in the vesiculation stimulation that contributes to cytotoxicity.

**Bacterial vesicles in the oceans: the potential global significance of vesicle production by the marine cyanobacterium *Prochlorococcus***

S. Biller, S. Roggensack, A. Thompson and S. Chisholm

MIT, USA

*Introduction*: Many species of pathogenic bacteria are known to release membrane vesicles (MVs), which can play diverse functional roles in mediating microbial interactions. Nothing is known about the abundance of MVs in the natural environment and their roles within the microbial communities that drive the major biogeochemical cycles of the planet. A sizable fraction of global primary production is carried out by the marine cyanobacterium Prochlorococcus – the smallest and most abundant photosynthetic organism on the planet. *Prochlorococcus* is found throughout the upper regions of the open ocean between 40°N and 40°S, with an estimated global population of 10^27^ cells. We are interested in the production of MVs by *Prochlorococcus* and their ecological roles within marine microbial communities. *Materials and methods*: We are examining vesicle release in laboratory cultures of *Prochlorococcus* using electron microscopy, nanoparticle analysis and biochemical techniques. *Results*: Multiple strains of *Prochlorococcus* release membrane vesicles. These structures average 80 nm in diameter (in contrast to the cell's diameter of 500–700 nm) and can be found at 10^7^–10^9^/ml in culture similar to the number of *Prochlorococcus* cells. *Prochlorococcus* MVs contain lipids, outer membrane and periplasmic proteins, and nucleic acids. Vesicles could potentially influence a number of processes in the ocean, including moving material between cells, serving as a source of fixed carbon for other bacteria or perhaps acting as a decoy for phage. Recent field studies suggest that MVs are abundant in both coastal and open ocean environments. *Conclusions*: This is the first description of extracellular vesicle production by a photosynthetic bacterium. Secreted membrane vesicles represent a previously unexplored component of the “dissolved organic carbon” in marine ecosystems and could provide important clues about the network of interactions among microbes and their environment.

**Oral Session 12 (Plaza Ballroom): Heart and Vessels April 18**

**Chair: *P. Quesenberry and D. Lyden* 13:30-15:00**

**Hypoxic reprogramming of exosomal microRNAs from human CD34+ progenitor cells augments its regenerative efficacy for ischemic tissue repair**

S. Misener^1^, R. Gupta^1^, D. Kim^1^, C. Kamide^1^, D.W. Losordo^2^, D.E. Vaughan^3^ and S. Sahoo ^1^

^1^Feinberg Cardiovascular Research Institute, Northwestern University, USA; ^2^Baxter Helathcare, USA; ^3^Feinberg School of Medicine, Northwestern University, USA

*Introduction*: In early clinical trials, human CD34+ stem cells have been shown to improve exercise tolerance in patients with myocardial ischemia and reduce amputation rates in limb ischemia. Recently, we have demonstrated that adult human CD34+ stem cells secrete exosomes (Exo) that mimic the angiogenic and therapeutic activity of the cells by transferring pro-angiogenic microRNAs. Exo has great potential to overcome the limitations of cell therapies for cardiovascular diseases, such as viability, retention and functional impairment of transplanted cells in the ischemic environment, while retaining its benefits. We hypothesise that the regenerative efficacy of the cell-free Exo derived from human CD34+ cells can be improved by hypoxic pre-conditioning, modulating its pro-angiogenic miRNA content. *Methods and results*: Exo from human peripheral blood-derived CD34+ cells cultured under hypoxia (H-Exo) were significantly proliferative, anti-apoptotic and angiogenic in vitro, as compared to Exo from cells under normoxia (N-Exo). Treatment of H-Exo significantly improved perfusion, increased capillary density and prevented ischemic limb amputation as compared to N-Exo, in a mouse model of hind limb ischemia. H-Exo was not significantly different in size, quantity secreted per cell, or, in expression of major proteins as compared to N-Exo, measured using DLS, 2-D DIGE and mass spectrometry analyses. Interestingly, H-Exo had significantly higher expression of several pro-angiogenic miRNAs. Trafficking studies using confocal microscopy and flow cytometry demonstrated transfer of fluorescent-labelled miRNAs/Exo to recipient endothelial cells both in vivo and in vitro. Currently, we are investigating the role of hypoxia-induced transcription factors instead of the H-Exo miRNA content. *Conclusion*: Our results demonstrate that hypoxia modulates the miRNA content of adult human CD34+ progenitor cell-derived exosomes improving its angiogenic and therapeutic potency.

**Monocyte-derived microparticles are elevated in patients with familial hypercholesterolemia: a potential atherosclerosis risk marker**

Morten Hjuler Nielsen ^1^, H. Irvine^2^, S. Vedel^3^, B. Raungaard^4^, H. Beck-Nielsen^5^ and A. Handberg^6^

^1^Department of Clinical Biochemistry, Aarhus University Hospital/Danish PhD School of Molecular Met; ^2^Department of Medicine and Cardiology A, Aarhus University Hospital; ^3^Department of Radiology, Aarhus University Hospital; ^4^Department of Cardiology, Aalborg Hospital, Aarhus University Hospital; ^5^Department of Endocrinology M, University of Southern Denmark, Odense; ^6^Department of Clinical Biochemistry, Aalborg Hospital, Aarhus University Hospital, Denmark

*Introduction*: Monocytes have been divided into three major subsets defined by their surface expression of CD14 and CD16: classical (CD14++CD16––), intermediate (CD14++CD16+), and non-classical (CD14+CD16++) monocytes. A high affinity to endothelial cells and the capacity to accumulate oxidised-low density lipoprotein (oxLDL) suggest that CD16+ monocytes are critical for atherosclerosis development. Microparticles (MPs) are increased in many diseases, including cardiovascular disease. oxLDL promotes shedding of MPs from monocytes, which may accelerate atherosclerosis. *Aim*: The current study has two purposes: (a) to examine to what extent CD16+ monocytes are elevated in hyperlipidemic individuals with high risk of atherosclerosis and (b) to examine the relation between circulating monocyte-derived microparticles (MMPs) and percentage of CD16+ monocytes. In addition, the relationship between oxLDL and monocyte phenotype as well as MP count was studied. *Methods*: Thirty individuals with familial hypercholesterolemia (FH) and 24 age- and gender-matched normolipidemic controls were included. Carotid intima-media thickness (IMT) was measured by ultrasonography. Monocytes and circulating microparticles (<1.0 µm) were characterised by flow cytometry using a BD FACSCalibur and a BD FACSAria III High Speed Cell Sorter, respectively. The microparticle gate was defined by an upper limit of 1.0 µm, and extending down to the noise threshold of the instrument. *Results*: FH individuals had higher percentage of CD16+ monocytes (p<0.02), higher IMT (p<0.03), increased oxLDL levels (p<0.001) and elevated levels of total MPs (p<0.03) and MMPs (p<0.001), compared with controls. MMPs correlated with IMT (rho=0.34, p<0.02) and CD16+ monocyte percentage (rho=0.38, p<0.005). OxLDL levels correlated with CD16+ monocyte percentage (rho=0.34, p<0.01) and were associated with IMT (r=0.34, p<0.01) and MMPs (r=0.45, p<0.001). *Conclusion*: Monocyte-derived microparticles are increased in hyperlipidemic individuals, and associated with non-classical CD16+ monocytes and IMT. Our data supports a model in which MMPs are released from monocytes upon activation by oxLDL.

**Endothelial cells require MIR-214 to secrete exosomes that suppress senescence and induce angiogenesis in human and mouse endothelial cells**

B.W.M. van Balkom ^1^, O.G. de Jong^1^, M. Smits^2^, J. Brummelman^1^, K. den Ouden^1^, P.M. de Bree^1^, M.A.J. van Eijndhoven^3^, D.M. Pegtel^3^, W. Stoorvogel^4^, T. Würdinger^2^ and M.C. Verhaar^1^

^1^Department of Nephrology and Hypertension, UMC Utrecht, Utrecht; ^2^Neuro-oncology Research Group, Department of Neurosurgery, VU University Medical Center, Amsterdam; ^3^Department of Pathology, VU University Medical Center, Amsterdam; ^4^Department of Biochemistry and Cell Biology, Faculty of Veterinary Medicine, Utrecht University, Utrecht, Netherlands

*Introduction*: Signalling between endothelial cells, endothelial progenitor cells and stromal cells is crucial for the establishment and maintenance of vascular integrity and involves exosomes, among other signalling pathways. Exosomes are important mediators of intercellular communication in immune signalling, tumour survival, stress responses and angiogenesis. The ability of exosomes to incorporate and transfer mRNAs encoding for “acquired” proteins or miRNAs repressing “resident” mRNA translation suggests that they can influence the physiological behaviour of recipient cells. We investigated whether miR-214, an miRNA that controls endothelial cell function and angiogenesis, plays a functional role in exosome-mediated signalling between endothelial cells. *Materials and methods*: Exosomes were isolated from cell culture supernatant by differential centrifugation and used for in vitro and in vivo functional assays for cell migration and angiogenesis. Cellular and exosomal miR-214 levels were reduced using anti-miR-transfection of exosome-producing cells. *Results*: We here demonstrate that in contrast to exosomes from control endothelial cells, exosomes from miR-214-depleted endothelial cells can no longer stimulate migration and angiogenesis in recipient cells. Exosome-delivered miR-214 repressed the expression of ataxia telangiectasia mutated in recipient cells, thereby preventing senescence and allowing blood vessel formation. Concordantly, specific reduction of exosome miR-214 content abolished their angiogenesis stimulatory function. *Conclusion*: Collectively our data indicate that endothelial cells require miR-214 to secrete exosomes that stimulate angiogenesis through silencing of ataxia telangiectasia mutated in target cells.

**Loss of contractile phenotype and mineral imbalance stimulate vascular smooth muscle cell calcification by enhanced exosome secretion**

Alexander Kapustin ^1^, M. Chatrou^2^, I. Drozdov, ^1^D. Soong^1^, M. Furmanik^1^, D. Alvarez-Hernandez^1^, P. Sanchis^1^, R. Molls^1^, R. Shroff^1^, X. Yin^1^, J. Skepper^3^, M. Mayr^1^, C. Reutelingsperger^2^, L.J. Schurgers^2^ and C.M. Shanahan^1^

^1^King's College London, United Kingdom; ^2^Maastricht University, Netherlands; ^3^Cambridge University, United Kingdom

*Introduction*: Vascular calcification is a regulated pathological process that is mediated by vascular smooth muscle cells (VSMCs) undergoing phenotypic modulation and triggered by the release of VSMCs-derived matrix vesicles (MVs) which serve as a nidus for pathological calcification in the pathological milieu. MV are normally protected from calcification by loading of a potent calcification inhibitor, fetuin-A. Here we studied the regulatory mechanisms of MV biogenesis and fetuin-A loading. *Materials and methods*: Human aortic VSMCs were isolated from medial explants of human aortic tissue. *Results*: Alexa488-labelled fetuin-A was internalised by human VSMCs, delivered to the early endosomes and late endosomal compartment where it was sorted for either lysosomal degradation or exocytosis in MVs. Biochemical and proteomic analysis revealed that MVs originated from multivesicular bodies (MVBs) and represent exosomes. Importantly, VSMCs exosome release was blocked by inhibitors of sphingomyelin phosphodiesterase 3, a regulator of exosome biogenesis, whilst inhibition of SMPD3 prevented VSMC calcification. Furthermore, treatment in calcifying conditions or differentiation into synthetic phenotype increased exosome secretion by VSMCs. Contrarily, acquisition of the contractile phenotype reduced exosome production. Importantly, only synthetic VSMCs were able to calcify, whereas contractile cells were protected against calcification. In agreement with our in vitro data, EM analysis of human vessel rings revealed MVB-like structures in VSMCs. Immunohistochemical staining showed an extensive staining of VSMCs-derived exosome markers, CD63 and annexin A6 in the atherosclerotic vasculature particularly in association with calcification. *Conclusions*: The circulating calcification inhibitor fetuin-A is recycled in VSMCs via the exosomal pathway. Exosome secretion is regulated by VSMC phenotypic conversion and represents a novel pathway for the regulation of VSMC calcification.

**Microvesicle protein levels are associated with increased risk for future vascular events and mortality in patients with clinically manifest vascular disease**

D.P.V. de Kleijn ^1^, D. Kanhai^2^, F. Visseren^2^, Y. van de Graaf^2^, A.H. Schoneveld^3^, L.M. Catanzariti^2^, L. Timmers^2^, L.J. Kapelle^2^, C.S.P. Uiterwaal^2^, S.K. Lim^4^, S.K. Sze^5^ and G. Pasterkamp^2^

^1^Surgery and CVRI NUS & NUHS, ICIN, UMC Utrecht, Singapore; ^2^UMC Utrecht, Netherlands; ^3^ICIN, Netherlands; ^4^IMB, Singapore; ^5^NTU, Singapore

*Background*: Microvesicles (MVs) are small plasma membrane vesicles that, among other, are involved in atherothrombotic processes. In the present study, we evaluated the risk of MV protein levels on the occurrence of new vascular events in patients with clinically manifest vascular disease. *Methods*: One thousand and sixty patients with clinically manifest vascular disease were prospectively followed for the occurrence of a new vascular event or death (median follow-up 6.4 years, interquartile range 5.2–7.3 years). MVs were isolated from plasma and MV protein levels of cystatin C, serpin G1, serpin F2 and CD14 were measured. Multivariable Cox proportional hazards models were used to estimate the risk for new vascular events, vascular mortality and all-cause mortality. *Results*: An increase in 1 standard deviation (SD) of cystatin C MV levels was related to an increased risk for myocardial infarction (HR 1.49; 95%CI 1.20–1.86), vascular mortality (HR 1.48; 95%CI 1.17–1.86), vascular events (HR 1.27; 1.07–1.52) and all-cause mortality (HR 1.41; 95%CI 1.18–1.69). Serpin F2 MV levels were related to an increased risk for myocardial infarction (HR 1.22; 95%CI 1.00–1.51), vascular mortality (HR 1.25; 95%CI 1.00–1.56) and all-cause mortality (HR 1.22; 95% CI 1.03–1.45). CD14 MV levels were related to an increased risk for myocardial infarction (HR 1.55; 95%CI 1.27–1.91), vascular mortality (HR 1.37; 95%CI 1.10–1.70), vascular events (HR 1.32; 95%CI 1.12–1.55), all-cause mortality (HR 1.36; 95%CI 1.15–1.62) and occurrence of ischemic stroke (HR 1.32; 95%CI 1.00–1.74). *Conclusions*: Cystatin C, serpin F2 and CD14 MV levels are related to an elevated risk for future vascular events and mortality in patients with clinically manifest vascular disease on top of existing risk factors.

**Endotoxin-induced monocytic microparticles have contrasting effects on endothelial inflammatory responses**

B. Wen^1^, V. Combes^1^, B. Bonhoure^1^, B.B. Weksler^2^, P.O. Couraud^3^, A. Magenau^1^, K. Gaus^4^ and G.E.R. Grau ^1^

^1^The University of Sydney, Australia; ^2^Cornell University, USA; ^3^Institut Cochin, France; ^4^The University of NSW, Australia

The blood–brain barrier is a multicellular anatomical system that maintains cerebral homeostasis and requires constant communication between its various components, notably endothelial cells. Microparticles (MPs), sub-micron plasma membrane vesicles, are biological effectors carrying cytosolic and surface markers mirroring their mother cell, but the ways they interact with their target cells remain poorly understood. Here we aimed to determine the properties of monocytic MP (mMPs) generated after endotoxin (lipopolysaccharide, LPS) activation and their effects on brain endothelium. Characterisation of mMPs by flow cytometry and RT-qPCR revealed that they carried similar pro- and anti-inflammatory profiles as their parent THP-1 cells. Upon co-culture with human microvascular brain endothelial cells (hCMEC/D3 line), mMP adhered in a shear-stress-resistant manner to endothelial cells. Under static conditions, the binding was significantly higher at 37°C than 4°C and this interaction increased in a time-dependent manner. Furthermore, confocal microscopy showed that MPs are internalised and subsequently compartmentalised. Using structured-illumination microscopy, by differential labelling of MP plasma membrane and cytosol, we showed that some MPs are able to release their cytoplasmic content within endothelial cells. Functionally, mMPs triggered specific responses in endothelial cells: they (a) induced the release of endothelial-derived MPs, (b) modified signalling pathways by diminishing endothelial pSrc (tyr416) expression and (c) promoted endothelial monolayer tightness, as demonstrated by increased trans-endothelial electrical resistance. These studies show that MPs derived from LPS-stimulated monocytes display a dual potential: exhibit enhanced pro-inflammatory properties, yet favour endothelial cell integrity. As such, these bioactive vesicles are capable of communicating with microvascular endothelial cells to elicit changes relevant to vascular permeability.

**Oral Session 13 (Georgian): Isolation Technology April 18**

**Chair: *E. Nolte-`t Hoen and P. Harrison* 13:30-15:00**

**Heparin affinity purification of extracellular vesicles**

Leonora Balaj^1^, A.A. Atai^1^, W. Weilin^1^, D. Mu^1^, B.A. Tannous^1^, J. Skog^2^, X.O. Breakefield^1^ and C.A. Maguire^1^

^1^Massachusetts General Hospital, USA; ^2^Exosome Diagnostics Inc, USA

*Introduction*: Isolation and purification of extracellular vesicles (EVs) from in vitro and in vivo biofluids is still a major challenge, and the most widely used isolation method still remains ultracentrifugation (UC). Affinity purification of biomolecules is an efficient way to achieve high purity without requiring expensive equipment. Previously, we have shown that heparin blocks EV uptake in mammalian cells in culture. *Materials and methods*: We compared 3 methods of purification: heparin affinity, UC and ExoQuick-TCTM. One millilitre of Affi-Gel^®^ Heparin Gel (Bio-Rad, Hercules, CA) was incubated with 1 ml of concentrated conditioned media (4°C). On day 2, the supernatant was collected, as well as 3 PBS washes. Lastly, 1 ml of 2 M NaCl was added to the beads and incubated overnight at +4°C. On day 3, the supernatant (eluate) was collected for downstream analysis. Another ml was used to purify EVs by UC and the last ml to isolate EVs with the commercially available kit ExoQuick-TC™ (System Biosciences, Mountain View, CA). *Results*: Here, we show that we can purify EVs from conditioned media using heparin-coated agarose beads. We confirm binding of EVs from 2 cancer cell lines (U87 and 293T) and normal HUVEC cells. We directly compared heparin-purified EVs to UC prepared EVs and ExoQuick-TCTM for the following characteristics: (a) purity by silver staining of SDS PAGE gels, (b) morphology by transmission electron microscopy, (c) EV markers by immunoblot and RNA analysis and (d) functionality by labelled EV uptake into mammalian cells. Heparin-purified EVs were of a higher purity and they retained the RNA content, morphology and functionality of UC EVs. *Conclusions*: Heparin-purified EVs contain EV-specific nucleic acid and proteins and are functional at uptake into recipient cells. We have discovered a simple and effective way to isolate a highly pure population of EVs using their apparent affinity for heparin.

**Direct detection of extracellular vesicles in human serum by exoscreen system**

Yusuke Yoshioka ^1^, Y. Konishi^1^, N. Kosaka^1^, H. Ohta^2^, H. Okamoto^2^, H. Sonoda^2^, H. Sasaki^3^ and T. Ochiya^1^

^1^Division of Molecular and Cellular Medicine, National Cancer Center Research Institute, Japan; ^2^Diagnostics Division, Shionogi & Co., LTD., Japan; ^3^Department of Urology, St. Marianna University School of Medicine, Japan

*Introduction*: Exosomes include both a common set of membrane and cytosolic proteins, and origin-specific subsets of proteins likely correlated to cell-type-associated characters. Therefore, detection of molecular features of tumour-derived exosomes may provide a novel approach to early detection, diagnosis and prognosis determination of tumours. The aim of this study is to detect cancer-specific antigens loaded on circulating exosomes and to provide a novel cancer biomarker. *Materials and methods*: We have developed a bead-based proximity assay named ExoScreen, which is based on AlphaLISA technique. In this assay, exosomes are captured by 2 antibodies modified in distinct ways. One is a biotinylated antibody, and the other is an antibody conjugated to AlphaLISA acceptor beads. To identify the component of tumour-derived exosomes, we performed proteomic analysis of exosomes derived from prostate cancer cell lines, PC3 cells and highly metastatic cell line PC-3M cells, as well as normal prostatic epithelial cell line PNT2 cells. *Results*: ExoScreen system allowed highly sensitive detection of exosomes in cell culture medium and human serum. The result of proteomic analysis revealed that several CD marker proteins were enriched in exosomes derived from prostate cancer cells compared to PNT2 cells. We evaluated this result with exosomes derived from PC3, PC-3M and PNT2 cells by ExoScreen using antibodies against these CD marker proteins. Although the signal intensity of CD9 was similar regardless of the cellular origin, those of the identified CD marker proteins were much higher in PC3 and PC-3M exosomes compared with PNT2 exosomes. Now we are investigating the relevancy of this system using serum from prostate cancer patients. *Conclusion*: These results suggest that ExoScreen system provides a widely applicable method to directly detect and quantify exosomes in human serum and also proposes a novel cancer biomarker.

**Engineering study: magnetic bead separation of microvesicle populations**

N.V.J. Wise ^1^, T. Grob^1^, S. Sheard^1^, K. Morten^2^ and J.S. Go^3^

^1^University of Oxford, United Kingdom; ^2^John Radcliffe Hospital, United Kingdom; ^3^Pusan National University, Republic of Korea

*Introduction*: The use of magnetic beads (MBs) in the detection and separation of particles at the microscale level has attracted much attention in recent years. MBs may be coated with an antibody specific to a surface antigen on a cell of interest. When exposed to a heterogeneous sample, the MBs attach specifically to the surface of target cells via antibody–antigen interaction. The application of an external magnetic field can manipulate the movement of MBs. As such, it is possible to separate targeted cells from a heterogeneous sample. In combination with a microfluidic device (MFD) and a magnetic field, MBs are a suitable technology for microvesicle isolation as they offer low limits of detection with high sample throughput at a low cost. *Materials and methods*: Using soft-lithography, an MFD was manufactured in polydimethylsiloxane. The path of the MBs through the MFD when influenced by external magnetic forces was modelled using finite element analysis, and the trajectory of the MBs was mapped. Samples were introduced into the device using hydrodynamic focusing. Fluorescent paramagnetic beads were used to establish the accuracy of the model. The separation efficiency of the MFD was analysed by flow cytometry. *Results*: Many factors were found to influence the MBs during separation experiments that were not predicted by the model. For example, hydrophobic surfaces caused additional drag forces; the use of a surfactant prevented this. The magnetic susceptibility of the beads was influenced by the bead concentration. The MBs must follow a trajectory that does not allow them to collide with the surfaces of the MFD. The position, size and shape of the magnet used and the modelling of the applied magnetic field are critical to a successful separation. *Conclusions*: Results obtained from this study into the optimum configuration of magnetic fields and microfluidic channels will allow the design of robust technology for reliable separation of microvesicle populations.

**Detection and profiling of erythrocyte-derived microvesicles from whole blood using microfluidic-assisted diagnostic magnetic resonance nanosensor**

J. Rho, J. Chung, R. Weissleder and H. Lee

Massachusetts General Hospital, USA

*Introduction*: Recent studies have established an integral role of microvesiculation in erythrocyte-aging processes. The number of erythrocyte-derived microvesicles (eMVs) increases over time in stored blood and thus could serve to monitor blood product quality. Considering that the risk of transfusion complications increases with aging of blood products, the use of eMVs as an indicator of blood product quality could have significant clinical implications. Here, we utilised cutting-edge diagnostic magnetic resonance (DMR) technology for analysis of eMVs in blood. *Materials and methods*: We have implemented a microfluidic device for one-step eMV isolation and labelling. The system incorporated a blood filter membrane to directly isolate eMVs from the whole blood. The isolated eMVs were then captured by antibody-coated microbeads and labelled with target-specific magnetic nanoparticles (MNPs). The DMR detection was performed using a miniaturised nuclear magnetic resonance (NMR) system. The decay rate (R2) of NMR signal was proportional to the MNP concentration, thus enabling the quantitation of target eMV markers. We profiled eMVs for glycophorin-A (GYPA), CD44, CD47 and CD55. *Results*: The measured DMR data showed excellent correlation with those of conventional methods (e.g. ELISA, nanoparticle-tracking analysis). Importantly, our assay identified GYPA as an efficient marker for eMV counting: the GYPA expression was highly elevated in eMVs, and the overall GYPA level was well correlated with total eMV numbers. The DMR data further showed that eMV counts in stored packed red blood cell (pRBC) units increased over time, validating the potential use of eMVs in assessing blood aging. *Conclusion*: We developed a microfluidic platform for (a) effective isolation and labelling of eMVs from whole blood and (b) quantification and profiling of different eMV markers. The developed system could be a valuable tool in monitoring blood aging and potential toxicity of eMVs, thereby improving the safety of blood product.

**Absolute particle numbers and miRNA abundance of cancer-cell-secreted microvesicle subpopulations**

M. Paulaitis, K. Agarwal, M. Saji and M. Ringel

Ohio State University, USA

*Introduction*: Cell-secreted microvesicles (MVs) consist of 2 populations, exosomes and shedding vesicles, which are released from all cell types in response to specific stimuli. Both populations contain miRNAs, although at different compositions. A feature that is thought to distinguish exosomes from shedding vesicles is their rich repertoire of miRNA. However, the individual contributions from these 2 populations of secreted MVs in transmitting differentiation-regulating signals to target cells have not been established. *Materials and methods*: Asymmetrical flow field fractionation–multiangle light scattering is used to fractionate the 2 populations of secreted MVs into exosomes and larger MVs, characterise the size distributions of the 2 populations and measure the absolute number of MVs in each population. The different fractions are further characterised based on their morphologies using cryo-transmission electron microscopy. In addition, qRT-PCR is used to quantify the copy numbers for selected miRNAs in the 2 fractions. *Results*: MVs secreted from different cancer cell lines in response to serum deprivation contain different populations of exosomes and larger MVs with the exosomes typically an order of magnitude greater in particle numbers. The particle numbers in both populations depend on the response time. We find miR-21, and depending on the cell line, other miRNAs in both MV populations, but at significantly different amounts for the different cell lines and as a function of response time. Moreover, the number of secreted MVs is significantly greater than the total copy numbers of miRNA for all cell lines and independent of the response time. *Conclusions*: We conclude that significant numbers of exosomes and larger MVs are released from cancer cell lines in response to serum deprivation, but only small numbers of these secreted MVs contain miRNA, suggesting that the mechanism for MV-mediated miRNA transfer to target cells is probabilistic nature.

**Complete exosome workflow solution: from isolation to characterisation**

Emily Zeringer, M. Li, T. Barta, V. Bagai, J. Schageman, S. Magdelano, R. Setterquist and A. Vlassov

Life Technologies, USA

Exosomes are a small subset of vesicles (30–150 nm) found in abundance in human body fluids, which function as carriers of a range of cargo, including different species of RNA and protein, between diverse locations in the body. The spectrum of current scientific interest in exosomes is wide and ranges from studying their functions and pathways to utilising them in diagnostics and therapeutics development. As such, there is a growing need for quick and easy methods for both isolation of exosomes and analysis of the containing cargo. Here, we describe our complete exosome workflow solution: (a) fast and efficient isolation of exosomes from cell media and various body fluids using Total Exosome Isolation reagents; (b) extraction of their “cargo” with Total Exosome RNA and Protein Isolation kit; (c) characterisation of exosomal RNA content using the personal genome machine (PGM) and qRT-PCR with TaqMan assays and (d) labelling exosomal RNA and lipid membranes with fluorescent dyes – enabling in vivo tracing to unravel exosomal functions and pathways.

**Coffee and Poster Viewing April 18**

**Poster Session III-IV 15:00-15:45**

**Parallel Oral Sessions 14-16 15:45-17:45**

**Oral Session 14 (Imperial Ballroom): Stem Cells April 18**

**Chair: *J. Aliotta and P. Kurre* 15:45-17:45**

**Mesenchymal stromal cell-derived extracellular vesicles inhibit in vitro and in vivo tumour cell proliferation**

S. Bruno ^1^, F. Collino^1^, M.C. Deregibus^1^, C. Grange^1^, C. Tetta^2^ and G. Camussi^1^

^1^University of Torino, Italy; ^2^Fresenius Medical Care, Germany

*Introduction*: Mesenchymal stromal cells (MSCs) have opposite effects on tumour, being able either to favour or to inhibit tumour growth. Factors produced by MSCs within the tumour microenvironment may be relevant for their biological effects. Recent studies demonstrated that extracellular vesicles (EVs) are an integral component of intercellular communication within the tumour microenvironment. *Materials and methods*: We have evaluated whether EVs derived from human bone marrow MSCs may stimulate or inhibit in vitro proliferation and cell death of HepG2 hepatoma, Kaposi's sarcoma and Skov-3 ovarian tumour cell lines by 5-bromo-2’-deoxy-uridine incorporation, ApoTox-GloTM Triplex assay and Tunel technique. Gene array profiles were performed to study gene expression of different cancer cell lines stimulated with EVs. In vivo, EVs were intratumour administrated in established tumours generated by subcutaneous injection of these cell lines in severe combined immunodeficiency disease (SCID) mice, and the growth of tumours was followed for 3 weeks. Tumour histology, proliferation and apoptosis were evaluated. *Results*: We found that EVs significantly affected cell cycle of all cell lines. An increase of cells in G0-G1 phase was observed suggesting a block in cell cycle progression; indeed, proliferation also significantly decreased. Moreover, EVs induced apoptosis in HepG2 and Kaposi's cells and necrosis in Skov-3. Gene array profile of different cancer cell lines stimulated with EVs showed activation of negative regulators of cell cycle, which may explain the biological effects. Different genes, involved in the control of cell cycle, were modulated in the diverse cancer cell lines. In vivo intratumour administration of EVs in established tumours significantly inhibited tumour growth in SCID mice. *Conclusions*: EVs from human MSCs inhibited in vitro cell proliferation and survival of different tumour cell lines and in vivo inhibited the growth of established tumours.

**Extracellular modulation of cell phenotype**

Peter Quesenberry ^1^, J. Aliotta^2^, M. Pereira^2^, M. Dooner^2^ and A. Sorokina^2^

^1^Division of Hematology & Oncology, USA; ^2^Rhode Island Hospital, Warren Alpert Medical School of Brown University, USA

*Introduction*: Extracellular vesicles modify mRNA, protein production and overall cell phenotype in a variety of target cells. *Materials and methods & results*: Murine-lung-derived vesicles enter murine marrow cells and induce mRNA expression of surfactants A-D, aquaporin-5 and clara-cell-specific protein. This cell entry is required for phenotype change, and 10–20% of marrow cells take up vesicles. These include all differentiated cell types and stem/progenitor cells. Experiments using species-specific mRNA for surfactants B and C in which rat lung was co-cultured with murine marrow, but separated from it by a cell-impermeable membrane, showed that immediately after co-culture both rat and mouse mRNA was highly expressed, but that the rat mRNA rapidly disappeared and the mouse mRNA persisted. The mouse surfactant B and C mRNA was highly expressed in cytokine-supported culture out to 13 weeks. Thus a stable epigenetic change was induced. The induction of surfactant B mRNA was found to be RNase sensitive. These data indicate that lung-derived vesicles induce a stable epigenetic change by transfer of an RNase-sensitive transcriptional agent separate from mRNA; presumably a small non-coding RNA. The lung-derived vesicles contain mRNA, protein and DNA including mitochondrial DNA and 239 species of microRNA. Further work with lung-derived vesicle phenotype change has shown that it is dependent upon the cell cycle status of the marrow progenitors and the treatment of the originator lung tissue, that is, radiation or no radiation. Similar results have been forthcoming with murine-liver-derived vesicles. *Conclusions*: Thus mesenchymal stem-cell-derived vesicles are mediators of epigenetic changes in target cells, and we further propose that the impact of mesenchymal stem cells in various tissue injury settings is mediated by vesicles, which then induce stable epigenetic change.

**Osteoblast-derived extracellular vesicles regulate haematopoietic stem cell fate**

Jess Morhayim, J. Demmers, T. de Jong, E. Braakman, J. Cornelissen, J. van de Peppel and H. van Leeuwen

Erasmus MC, The Netherlands

*Introduction*: Osteoblasts are major constituents of the haematopoietic stem cell (HSC) niche and play an important role in the control of HSC renewal and differentiation. Osteoblasts secrete extracellular vesicles (EVs) in the form of matrix vesicles involved in bone mineralisation; however, information about a role in cell–cell communication is still lacking. In this study, we focus on the characterisation of human osteoblast EVs and investigate their potential as mediators of communication with HSCs. *Materials and methods*: We used a human pre-osteoblast-based in vitro bone formation model to isolate EVs at various time-points during osteoblast differentiation and mineralisation by a series of ultracentrifugation steps. We characterised EVs by electron and atomic force microscopy and proteomics. Furthermore, we studied their interaction with human umbilical cord blood (UCB) CD34+ HSCs by fluorescent labelling and flow cytometric analysis, and investigated their impact on cell proliferation and differentiation. *Results*: Microscopic analyses demonstrated that osteoblast EVs are very heterogenic in size and morphology. Mass-spectrometry-based proteomic analyses identified known EV proteins and an interesting range of membrane and signalling proteins that may be linked to cell communication. Fluorescently labelled osteoblast EVs were internalised by CD34+ UCB-HSCs in a dose-dependent manner as detected by flow cytometry. Cultures of CD34+ UCB-HSCs with osteoblast EVs led to a donor-dependent 1.8–3.5-fold increase in total blood mononuclear cells while stimulating the proliferation of the CD34-expressing population by 2–3 fold. *Conclusions*: Osteoblasts secrete EVs packaged with proteins not primarily linked to mineralisation suggestive of a novel mechanism of intercellular communication. Osteoblast EVs are taken up by CD34+ UCB-HSCs and promote ex vivo expansion of CD34-expressing cells proving to be strong candidates to control the osteoblastic HSC niche.

**Exosomes differentially modulate the osteogenic and adipogenic induction of bone-marrow-derived mesenchymal stem and progenitor cells**

Thomas Lener ^1^, M. Öller^1^, D. Peckl-Schmid^2^, D. Streif^2^, M. Gimona^2^, S. Laner-Plamberger^1^, F. Rivera^1^, L. Aigner^1^ and E. Rohde^1^

^1^Paracelsus Medical University Salzburg, Austria; ^2^Paracelsus Medical University Salzburg – SCI-TReCS, Austria

*Introduction*: Bone-marrow-derived mesenchymal stem/progenitor cells (BM-MSPCs) and endothelial colony-forming cells (ECFCs) are attractive candidates for regenerative cell therapy. It is known that cell communication can be mediated either by direct cell–cell contact and paracrine factors or by cell-derived exosomes. Here, we tested whether exosomes derived from either progenitor cell type are sufficient to effectively modulate lineage induction in BM-MSPCs. *Methods*: BM-MSPCs were cultured in 10% human platelet lysate (HPL) in alpha-MEM. Proliferation and osteogenic and adipogenic differentiation of BM-MSPCs were tested in vitro in the presence or absence of exosomes derived from either MSPCs (homotypic exosomes) or ECFCs (heterotypic exosomes). Manual cell counting, colony formation and continuous impedance assays (xCELLigence^®^) were employed. Flow cytometric analysis was used to monitor lineage marker expression. Fluorescence and phase contrast microscopy was performed to visualise exosome uptake, and osteogenic and adipogenic differentiation, respectively. *Results*: We show that homotypic but not heterotypic exosomes potentiate the osteogenic and adipogenic induction in BM-MSPCs. Furthermore, after osteogenic induction, homotypic exosome stimulation maintains the secretion and deposition of mineralising matrix in BM-MSPCs without any inductive supplement. In contrast, heterotypic exosomes reduce osteo- and adipogenic differentiation in BM-MSPCs, but stimulate the proliferative potential of BM-MSPCs. *Conclusions*: These results show that homotypic exosomes promote osteogenic and adipogenic induction, whereas heterotypic (ECFC-derived) exosomes stimulate the proliferation of BM-MSPCs. This is the first description of enhanced lineage induction of BM-MSPCs by stem-cell-derived exosomes.

**Paracrine function of mesenchymal stem cells for differentiation of neural stem cells**

L.V. Braccioli ^1^, C.T.J. van Velthoven^1^, P.J. Coffer^2^, M.J. Lorenowicz^2^ and C.J. Heijnen^3^

^1^Laboratory of Neuroimmunology and Developmental Origins of Disease, UMC, Utrecht, The Netherlands; ^2^Laboratory of Cell Growth and Differentiation, UMC, Utrecht, The Netherlands; ^3^Laboratory of Neuroimmunology of Cancer-Related Symptoms, MD Anderson Cancer Center, Houston, Texas, USA

Impaired blood–brain gas exchange in the perinatal period can lead to damage to white and grey matter in the neonatal brain. Consequences of neonatal hypoxia ischemia (HI) are mortality or severe encephalopathy. Mesenchymal stem cells (MSCs) have a significant regenerative potential in animal models of perinatal brain damage and may represent a promising treatment. We have shown that intranasal administration of MSC induces partial regeneration of white and grey matter and improves functional outcome in a mouse model of HI (9-day-old pups). Neuroregeneration does not occur through differentiation of MSCs into neurons and oligodendrocytes. Therefore, we hypothesised that MSCs promote repair by stimulating endogenous stem cells in the brain. Neural stem cells (NSCs) are capable of differentiating into neurons, oligodendrocytes and astrocytes. In the brain, NSCs reside in specific areas. After HI, these NSCs may migrate to the lesion site to interact with the MSC that have migrated to the lesion site as well to form a regenerative niche. However, it is not known whether MSCs exert their effect on NSCs via direct cell-to-cell contact or by paracrine communication. We developed a novel co-culture system that allows the exchange of soluble factors, including exosomes, between MSC and NSC but does not allow cell-to-cell contact. Our preliminary data show that co-culture with MSC favours differentiation of NSC towards the neuronal and oligodendrocytic lineages when compared to NSC cultured without MSC. These data are consistent with our in vivo observation of grey and white matter regeneration after MSC treatment in HI mice. Recently, MSC-derived exosomes were proven to have a beneficial effect on the treatment of myocardial ischemia/reperfusion injury. To investigate whether MSC-derived exosomes are involved in the regenerative process in our model of HI, we are currently testing their effects in vitro on NSC differentiation and their in vivo effect using p9 mice subjected to HI.

**Exosome mediates the immunodulatory activity of human mesenchymal stem cell secretion**

Sai Kiang Lim

A*STAR Institute of Medical Biology, Singapore

*Introduction*: The use of mesenchymal stem cell (MSC) to treat intractable immune diseases such as graft-versus-host disease has been approved. Despite its well-documented efficacy, the underlying mechanism remains unknown and is thought to be mediated by its secretion. We have previously shown that exosome mediates the cardioprotective activity of MSC. Here, we propose that this exosome also mediates the efficacy of MSC against immune diseases through its capacity to activate monocytes and enhance the production of CD4+CD25+FoxP3+ regulatory T-cell (Treg) production. In addition, MSC exosomes are hypo-immunogenic and are negative for MHC class I and II, B7-1 (CD80), B7-2 (CD86) or CD40. *Materials and methods*: MSC exosomes were assessed for monocyte activation potential activity using 2 commercially available THP1 reporter cell lines, THP1-XBlue cells and MyD88-deficient THP1-XBlue (THP1-XBlue-defMYD) cells. The effect of exosomes on Treg production was assessed in vitro using CD4+ T cells that were activated with anti-CD3 and anti-CD28. For in vivo assessment, Tregs in the spleen of mice was assayed by FACS. *Results*: MSC exosomes polarised THP-1 cells towards a M2 phenotype with a polymyxin-resistant induction of both anti-inflammatory (e.g. IL-10) and pro-inflammatory (e.g. IL-1α, IL-6, TNF-α and IL-12p40) through MyD88-dependent and MyD88-independent signalling pathways. Primary human and mouse monocytes when exposed to MSC exosomes also displayed a similar M2 phenotype but with a more rapid response. MSC exosomes also have the potential to polarise CD4+ T cells to Treg in vitro and increase Treg in an exosome-treated mouse model. *Conclusion*: MSC exosome possesses many of the immunodulatory properties that have been ascribed to MSCs and could underpin the immunotherapeutic activity of MSC secretion.

**IVF embryos release extracellular vesicles which may act as an indicator of embryo quality**

C. Gardiner, J.F. Ferriera, M. Poli, K. Turner, T. Child and I.L. Sargent

University of Oxford, United Kingdom

*Introduction*: There is a need to improve the quantitative and objective assessment of IVF embryo quality to determine those which are most likely to form pregnancies after transfer to the mother. We hypothesised that cultured embryos would release extracellular vesicles (EVs) and the size and concentration of these EVs might reflect embryo quality. *Materials and methods*: Embryo quality was assessed by standard morphological criteria. Nanoparticle tracking analysis was used to measure EV size and concentration in culture supernatants from 239 embryos from 18 women (148 at day 3 and 91 at day 5). A fluorescent amphoteric dye (Cellmask™) was used to determine whether the particles present were membrane-derived EVs and an intercalating dye (SYBR Green™) was used to detect DNA. The relationship between EV size and concentration with day of embryo development and morphological quality was investigated. *Results*: EVs were present in all embryo supernatants (median EV per embryo: 7.9×107 day 3; 8.0×107/ml day 5). More than 85% of EVs stained with Cellmask showing them to be membrane-derived. Paired EV median size measurements taken at day 3 and day 5 of embryo development showed significant correlation (r=0.50, p<0.0001). Increasing EV size was strongly associated with decreasing embryo quality (202 nm good, 218 nm average, 222 nm poor and 227 nm arrested development; p<0.004). Normal EV size was observed in embryos with apparent developmental arrest at day 5 which then recovered at day 6. DNA was detected by fluorescent staining in a variable proportion of EV in all 18 samples tested. *Conclusions*: IVF embryos release EVs into the culture medium. EV numbers increase with developmental stage. EV size correlates with embryo quality and may predict recovery from apparent growth-arrested. Further studies are required to determine the relationship between embryo EV and implantation potential.

**Levels of circulating vesicular microRNA-31 increase with age and inhibit osteogenic differentiation capacity of mesenchymal stem cells**

Sylvia Weilner ^1^, E. Schraml^1^, K. Wassermann^1^, M. Wieser^1^, P. Messner^1^, K. Schneider^1^, L. Micutkova^2^, K. Fortschegger^1^, A.B. Maier^3^, R. Westendorp^3^; H. Resch^4^, S. Wolbank^5^, H. Redl^5^, P. Jansen-Dürr^6^, P. Pietschmann^7^, R. Grillari-Voglauer^1^ and J. Grillari^1^

^1^University of Natural Resources and Life Sciences, Austria; ^2^Institut für Biomedizinische Alternsforschung, Austria; ^3^University Medical Center Leiden, The Netherlands; ^4^KH der Barmherzigen Schwestern, Austria; ^5^Ludwig Boltzmann Institute for Experimental and Clinical Traumatology, Austria; ^6^Innsbruck Medical University, Austria; ^7^Medical university Vienna, Austria

*Introduction*: Ageing is a complex process that results in the decline of physiologic functions due to accumulation of damage in cells and tissues. The regenerative power of stem and progenitor cells has been found to decline with age and to be influenced by the systemic environment. Here, we set out to identify circulating factors of the aged systemic environment that influence the functionality of mesenchymal stem cells (MSCs). *Results*: While searching for responsible factors, we found that senescent endothelial cells (senECs), which were shown to accumulate with age in vivo, secrete CD63-positive extracellular vesicles (EVs) enriched in miR-31 compared to young cells. The presence of miR-31 within EVs was also visualised by electron microscopy in situ hybridisation. Since endothelial cells line the blood vessels, supporting indications that this phenomenon may also occur in vivo were provided by analysing human plasma samples. EVs isolated from plasma of elderly or from osteopenia patients exhibited elevated miR-31 levels compared to EVs of young donors. Furthermore, it could be demonstrated that CD63-positive EVs isolated from senECs are taken up by MSCs in a time- and dose-dependent manner via endocytosis. Exposing MSCs to EVs secreted by senECs or isolated from plasma of elderly donors significantly reduced their osteogenic differentiation potential compared to MSCs incubated with secreted EVs of young cells or circulating EVs of young donors. Finally, the impairment of osteogenesis by EVs was attributed to vesicular miR-31, and its underlying molecular inhibitory effect was illuminated by showing that miRNA-31 targets FZD3, a factor necessary for osteogenic differentiation. *Conclusion*: Summarising our data suggest that miR-31 is able to inhibit osteogenesis and that it is enriched within circulating CD63-positive EVs with age as well as in the case of osteopenia. Thus, it might serve as a diagnostic and therapeutic target whenever osteogenesis is a limiting factor.

**Platelet activation and microparticle formation in acute SIV infection**

Oral Session 15 (Plaza Ballroom): Infection: Virus and Prions April 18

Chair: *M. Kuehn and A. Hill* 15:45-17:45

Kelly Metcalf Pate ^1^, K. Witwer^1^, C.N. Morrell^2^ and J.L. Mankowski^1^

^1^Johns Hopkins University School of Medicine, USA; ^2^University of Rochester, USA

*Introduction*: Activated platelets participate in the innate immune response to pathogens. Platelet microparticles are released from activated platelets and are the most abundant microparticle in the blood. Platelet microparticles have the potential to amplify the influence of activated platelets on the innate immune response. We hypothesised that platelet activation and platelet microparticle formation may occur concurrently during acute HIV infection. *Materials and methods*: We examined platelet activation and platelet microparticle formation during acute infection in the SIV-infected macaque model of HIV infection. Expression of surface markers of platelet activation was queried in SIV-infected and uninfected pigtailed macaques with flow cytometry using 2 methods: p-selectin expression on platelets in platelet-rich plasma (n=10 SIV-infected, n=5 uninfected), and p-selectin and CD40L expression on CD42a+ platelets in whole blood (n=6 SIV-infected, n=3 uninfected). Microparticles were identified using flow cytometry of whole blood and the percentage of particles of platelet origin identified by expression of CD42a. *Results*: In platelet-rich plasma, an increase in the percentage of platelets expressing p-selectin (Mann–Whitney P=0.023) and an increase in the level of surface expression of p-selectin (Mann–Whitney p=0.031) were noted in SIV-infected macaques compared to controls on day 10 of acute infection. Platelet activation was further indicated by a trend of increased surface expression of p-selectin (Mann–Whitney p=0.14) and an increase in CD40L expression (Mann–Whitney p=0.036). Of 6 SIV-infected macaques, 4 concurrently demonstrated increased CD42a+ microparticle formation. *Conclusions*: Platelet activation occurs during acute SIV infection and is accompanied by increased platelet microparticle formation in some macaques. Further study on the role of platelet microparticles in the innate immune response to HIV is needed.

**Regulation of HCV RNA and MIR-122 by RAB27A-dependent exosome secretion pathway**

T.C. Chen and P. Sarnow

Department of Microbiology and Immunology, Stanford University, Stanford, CA, USA

Exosomes are microvesicles that are secreted from eukaryotic cells into the extracellular environment. It is thought that exosomes can transfer functional proteins, mRNAs and microRNAs from one cell to another. Several studies have found that viruses employ microvesicle biogenesis pathways, not only for virus assembly and release, but also for cell-to-cell communication in relation to immune response or to facilitate viral spread. Here, we investigate the roles for extracellular exosomes during hepatitis C virus (HCV) infection. Exosomes were purified from uninfected and infected cells by subsequent centrifugations. These exosome preparations contained exosome markers CD81 and CD63, but lacked the ER marker calnexin. Exosomes from infected cells contained the viral structure proteins core and E2, but lacked NS5A, suggesting that viral replication complexes were excluded from exosomes. Moreover, primer-extension studies demonstrated that HCV mRNA and microRNA miR-122 were present in exosomes. These findings argue that viral RNA, viral proteins and miR-122 RNA molecules can be transferred from cell-to-cell by exosomes. Indeed, inoculation of uninfected cells with exosomes, purified from infected cells, could initiate an infectious viral cycle. Moreover, exosome secretion pathway was shown to be controlled by Rab27a, a small GTPase protein. Depletion of Rab27a in cells by siRNAs resulted in decrease of extracellular exosomes. Knockdown of Rab27a in infected cells caused a decrease in intracellular and extracellular virus production. This finding implies that blocking the exosome secretion pathway does not affect HCV release. Interestingly, we found that the levels of HCV RNA and miR-122 decrease in Rab27a-depleted cells. Overexpression of miR-122 in Rab27a knockdown cells can rescue HCV RNA level. These findings suggest that interfering with the exosome secretion pathway affects HCV replication though decreasing the level of HCV RNA and miR-122.

**Identification of cellular factors affecting the exosomal release of infectious prions**

P. Leblanc ^1^, D. Vilette^2^, K. Laulagnier^3^, M. Provansal^4^, S. Lehmann^4^ and G. Raposo^5^

^1^CNRS UMR5239, LBMC, ENS LYON, France; ^2^INRA/ENVT UMR INRA/ENVT 1225 Interactions Hôtes- Agents Pathogènes Pathologie du bétail, France; ^3^Inserm U836, Neurodégénérescence et Plasticité, Grenoble Institut des Neurosciences, France; ^4^INSERM-UM1 U1040, Physiopathologie, diagnostic et thérapie cellulaire des affections neurodégénérati, France; ^5^CNRS-UMR144, Structure and Membrane Compartments, Institut Curie, Paris, France

Transmissible spongiform encephalopathies (TSEs) are fatal neurodegenerative disorders affecting humans and animals. These prion diseases are characterised by the conformational conversion of the normal cellular prion protein PrPC into an abnormal misfolded and partially protease resistant isoform called PrPSc. PrPSc is linked with prion infectivity and is the main component of the prion agent. In 2004, we were the first to identify that prion-infected cells release infectious prions in association with exosomes. The cellular factors and the mechanisms by which prions are recruited and released through the exosomal pathway are uncharacterised. The endosomal sorting complex required for transport (ESCRT) machinery is involved in the multivesicular body biogenesis and consequently in exosomes formation. Similarly, the sphingomyelinase2 (smase2)-dependent ceramid biosynthesis modulates exosomes biogenesis. Here, we investigated the role of these 2 pathways in the release of infectious exosomes. HIV-1 lentivectors encoding small interfering RNAs (ShRNAs) were selected to target different ESCRT components (Hrs, Tsg101, Chmp6 and Chmp2B) the VPS4 complex, the accessory protein Alix, the smase2 or a non-specific sequence (negative control). The murine neuroglial MovS cells infected with a sheep scrapie agent were transduced with the lentivectors, and the released exosomes were recovered by differential centrifugation. Presence of infectious prions was monitored by WB of abnormal PrPSc and by scrapie cell assay in secreted exosomes and in the corresponding cells. Our data indicate that prion accumulation within the cells can be differentially affected by the ESCRT machinery. In addition, some ESCRT components and the ceramid pathway can also selectively inhibit exosomal release of prions. The identification of cellular factors affecting the targeting of infectious prions to exosomes may eventually help in the screening and development of therapeutics.

**Extracellular vesicles derived from HIV-1-infected cells contain TAR RNA**

A. Narayanan ^1^, S. Iordanskiy^1^, R. Das^1^, S. Santos^2^, E. Jaworski^1^, G. Sampey^1^, M. Uglesias-Ussel^3^, K. Kehn-Hall^1^, M. Young^4^, C. Bailey^1^, C. Gilbert^5^, F. Romerio^3^ and F. Kashanchi^1^

^1^George Mason University, USA; ^2^George Washington University, USA; ^3^University of Maryland School of Medicine, USA; ^4^Georgetown University Medical Center, USA; ^5^CHUQ Research Center, Laval University, Canada

*Introduction*: Exosomes derived from virally infected cells have been shown to contain viral microRNAs (miRNAs). HIV-1 encodes its own miRNAs that regulate viral and host gene expression. The most abundant HIV-1-derived miRNA is the TAR miRNA. *Materials and methods*: We have utilised a series of filtration and ultracentrifugation methods to enrich for extracellular vesicles from culture supernatants of HIV-1-infected cells. We have characterised these vesicles by Western blotting and quantitative RT-PCR. Functionally, we have utilised reverse transcriptase assay to determine susceptibility of naïve cells to infection in the presence of exosomes. *Results*: We demonstrate the presence of TAR RNA in exosomes from cell culture supernatants of HIV-1-infected cells. The exosomes contained ~220 host proteins. We detected the host miRNA machinery proteins, Dicer and Drosha, in exosomes from infected cells. Analysis of inflammatory mediators in exosomes also revealed inclusion of distinct cytokines in exosomes of HIV-infected cells. Functionally, prior exposure of naïve cells to exosomes from infected cells increased susceptibility of the recipient cells to HIV-1 infection. Our analysis of patient material revealed that enriched exosomes from HAART-treated patients contained high copy numbers of TAR RNA. *Conclusion*: Taken together, our experiments demonstrate that HIV-1-infected cells produced exosomes that are uniquely characterised by their proteomic and RNA profiles that may contribute to disease pathology in AIDS.

**Exosomes in hepatitis C virus infection mediate CD81-independent transmission and are rich in Ago2-miR122-HSP90 complexes**

Gyongyi Szabo, T. Bukong, S. Bala, K. Kodys and K. Kodys

University of Massachusetts Medical School, USA

*Introduction*: Current anti-hepatitis C virus (HCV)-neutralising antibody therapy is severely limited by observed virus transmission without evidence of viral resistance to therapy. This indicates that HCV can subvert immune therapies and transmit infection though the precise mechanisms remain unknown. Evidence suggests that exosomes/microvesicles can mediate receptor-independent transfer of genetic material between cells though their role in HCV transmission remains unknown. Here, we aimed to determine the role of exosomes/microvesicles in the transmission of HCV infection. *Materials and methods*: Exosomes were purified from culture supernatants of HCV J6/JFH-1-infected Huh 7.5 cells and from patient serum samples using Exoquick reagent. The exosomes were visualised and quantified using Nanosight. Exosome composition as well as their capacity to mediate HCV transmission was determined using western blotting, real time quantitative PCR and RNA-CHIP analysis. *Results*: Exosomes from HCV J6/JFH-1-infected Huh 7.5 cells, some treatment naïve and all HCV-infected treatment non-responder patients contained HCV RNA and were rich in Ago2, miR-122 and HSP90. Compared to free HCV viruses, exosomes containing HCV RNA were capable of transmitting HCV infection even in the presence of a potent anti-CD81 antibody. Remarkably, the use of a miR-122 inhibitor or use of the HSP90 inhibitor, 17-demethoxygeldanamycin (17-DMAG), suppressed exosome-mediated transmission of HCV infection in vitro. *Conclusions*: HCV is present in exosomes in the circulation of some patients with chronic HCV infection. Exosomes derived from HCV-infected hepatoma cells and HCV-infected patients can mediate CD81-independent transmission of HCV. The use of a miR-122 or HSP-90 inhibitor can significantly suppress exosomal transmission of HCV infection, suggesting their use when treatment failure occurs with anti-HCV immune therapies.

**Exosomes are present in greater amounts in antiretroviral-naïve HIV-1-infected patients**

A. Hubert ^1^, C. Subra^1^, P.F. Labrecque^1^, M.A. Jenabian^2^, J.P. Routy^2^ and C. Gilbert^1^

^1^Centre de recherche du CHU de Québec, Université Laval, Canada; ^2^Chronic Viral Illness Service, Montreal Chest Institute, McGill University Health Centre, Canada

Exosomes represent microvesicles ranging between 30 and 100 nm in diameter, originating from endosomal compartments and released into the extracellular medium for communication between neighbouring cells. In addition, plasma-membrane-derived microvesicles or exosomes found in plasma reflect local activation and possibly viral production. Since human deficiency virus (HIV-1) particles share with exosomes and microvesicles common physical characteristics and both contain proteins, lipids and nucleic acids, discriminating between them remains difficult. We investigated whether or not exosomes of HIV-1-infected patients reflect disease progression. Using acetylcholinesterase (AChE) assay as specific marker of exosomes, Nanosizer particle size and distribution measurement to discriminate between microvesicles, exosomes and HIV-1 particles recovered from plasma and infected cells. To this end, we compared the particles collected in sera obtained from antiretroviral-regimen-naïve, successfully treated, elite controller patients and healthy subjects. Results showed a significant increase in microvesicle and exosome size in antiretroviral-naïve HIV-1-infected patients. AChE activity and microvesicle size were inversely correlated with CD4, platelet and neutrophil counts, and with CD4/CD8 ratio. In contrast, AChE activity and microvesicle size were positively correlated with CD8 count. Plasma microvesicles and exosomes derived from cells infected in vitro were enriched in Dap-3, a protein involved in cell death. These results indicate involvement of microvesicles in mediating cell death and in the inflammatory response in HIV-1-infected subjects. Characterisation of exosomes and microvesicles from HIV-1 patients provides a new pathway contributing to immunodeficiency and to disease progression, and their modulations may contribute to novel treatment strategy.

**EBV-specific micro-RNA via exosome: a key inter-cellular machinery between EBV+ tumour and tumour-surrounding immune cells?**

Ai Kotani

Tokai University, Japan

*Introduction*: EBV is associated with heterogeneous lymphomas, which are subdivided into three types of latency. Hodgkin's lymphoma (HL) is characterised by a minority of neoplastic HRS, which are embedded in non-neoplastic bystanders. Without them, the HRS cells are incapable of being engrafted in immunodeficient mice. In this context, the bystanders are tumour-supportive “inflammatory niche”. Because of the complexity of its interplay, the detailed mechanism remains elusive. Recently, small RNA was reported to be conjugated in exosomes, transferred to cells, and involved in tumour metastasis. Moreover, EBV-infected cells produce exosomes that contain viral encoded, EBV specific miRNAs (EBV-miRNAs). Accordingly, we hypothesised that exosomal EBV-miRNAs might redirect tumour surrounding immune cells from tumour reactive into tumour-supportive inflammatory niche, which ultimately leads to tumour progression. *Methods*: We evaluated the expression of EBV-miRNAs in EBV+HL clinical specimens by in situ hybridisation, their functional characterisation in vitro, and its effects on persistent infection and tumour development in vivo. *Results*: The exosomes produced by EBV+ cells (EBV-Ex) were harvested either from the media of the type III or type I EBV-infected cells, in which EBV-miRNAs were relatively rich or vacant. The EBV-Ex uptake was detected only in monocyte/macrophage (Mo/Mf) and EBV-miRNAs effects were potent on Mo/Mf in inducing CD69, IL-10, and TNF, suggesting the possibility that EBV-miRNAs might polarise Mo/Mf into tumour-associated Mf. In vitro, EBV-miRNAs suppress cell proliferation, while, in vivo mouse model, they are required to develop LPD, suggesting that EBV-miRNAs affect on the microenvironment to cause LPD. Most importantly, exosomal EBV-miRNAs were transferred in Mf in human EBV+ HL samples. *Conclusion*: EBV might utilise the exosomal machinery to secrete key viral-encoded miRNAs, through which EBV+ cells could modulate the tumour microenvironment.

**Novel route of prophage induction across bacterial species mediated by outer membrane vesicle-phage complexes**

M.J. Kuehn and A. Manning

Duke University Medical Center, United States

*Introduction*: Phage are viral particles that are abundant and ubiquitous in nature. A large proportion are bacteriophage, specifically infectious to bacteria. Many phage identify their host by interactions with the bacterial outer membrane. Bacterial outer membrane components can also be found as secreted membranous spheres known as outer membrane vesicles (OMVs). *Materials and Methods*: Previous work in our lab has shown that OMVs can bind the bacteriophage T4 irreversibly and quickly and that this interaction reduces the infectivity of the T4. Here, we explore the downstream consequences of T4-OMV complex formation on a non-T4 host species, *Pseudomonas aeruginosa*. *Results*: We found that T4-OMVs can induce a novel, D3 family prophage in *P. aeruginosa*, whereas un-complexed OMVs and T4 at the same concentrations could not. Lysozyme was sufficient, but inefficient, at inducing the prophage. By analyzing single gene mutants of *P. aeruginosa*, it was determined that T4-OMV-mediated prophage induction was independent of previously identified pathways, suggesting a novel response pathway. *Conclusions*: The data demonstrate that phage-OMV complexes can induce prophage in an unrelated bacterial species and that this represents a new mechanism for prophage induction within poly-microbial communities.

**Oral Session 16 (Georgian): Proteomics April 18**

**Chair: *R. Simpson and D. Di Vizio* 15:45-17:45**

**Proteomics of exosomes secreted by cancer cell lines and primary cells reveals oncogenic signalling and biomarker potential**

Connie Jimenez ^1^, M. De Wit^1^, R.J.A. Fijneman^1^, M. Lavaei^1^, M.J.A. Weerts^1^, S. Zweegman^1^, H. De Wit^2^, J.R.T. Van Weering^2^, T. Schaaij-Visser^1^, J.C. Knol^1^, S.R. Piersma^1^, T.V. Pham^1^, G.A. Meijer^1^, H.M. Verheul^1^ and M. Pegtel^1^

^1^VU University Medical Center, The Netherlands; ^2^VU University, The Netherlands

*Introduction*: Exosomes are small secreted membrane vesicles with important functions in intercellular communication. In cancer, these functions may include maintenance of cancerous traits. Importantly, exosomes carry tumour-specific antigens and have been identified in various biofluids. Here we employ mass-spectrometry-based proteomics, to identify exosome-enriched proteins and cancer(-type)-specific proteins to obtain insight into aberrant exosomal functions in cancer and for candidate biomarker discovery. *Methods*: Exosomes and soluble secretome were harvested by differential centrifugation from a panel of 9 human cancer cell lines and 2 primary human cells. The exosome preparation was verified by electron microscopy and western blot. Quantitative proteomics was based on Gel-nanoLC-MS/MS and spectral counting. The betabinomial test was employed for significance analysis. *Results*: The total dataset of exosome protein profiles contained 3,302 proteins with 1,300–1,400 proteins per cell line. The core exosome proteome shared by all cells comprised 343 proteins of which a subset was highly enriched in exosome relative to lysate or to soluble secretome, including multiple established exosome markers. The exosome-enriched proteins were associated with the terms RNA post-transcriptional modification, protein synthesis and cell signalling, among others. In cancer exosomes, we identified established tumour-type-specific antigens and proteins belonging to oncogenic pathways. *Conclusions*: Comparative analysis of exosome versus soluble secretome proteomes revealed a core exosome proteome associated with specific functions that may yield better insight into exosome biology. In addition, numerous new proteins were identified that might be involved in exosome-related tumourigenesis. On-going studies focus on secretome and exosome proteomics of chemosensitive and chemoresistant AML and NSCLC.

**Application of dynamic exclusion proteomics and quantitative proteomics to identify immune-related proteins in nasal exosomes: altered expression in respiratory diseases**

Cecilia Lässer ^1^, S.E. O'Neil^1^, C. Sihlbom^2^, S. Hansson^2^, B. Lundbäck^1^, Y.S. Gho^3^ and J. Lötvall^1^

^1^University of Gothenburg/Krefting Research Centre, Sweden; ^2^Proteomics Core Facility/University of Gothenburg, Sweden; ^3^Department of Life Science, Pohang University of Science and Technology (POSTECH), Republic of Korea

*Introduction*: Nasal lavage fluid (NLF) is easily collected, a sample that may be used for analysing mediators and biomarkers in airway diseases. The aim of this study was to establish the proteome of nasal exosomes and determine alterations during asthma and chronic rhinosinusitis (CRS). *Methods*: (a) Exosomes were isolated from 2 pools of NLF from healthy controls. The proteome was analysed by LC-MS/MS with the application of 2 exclusion lists. (b) Exosomes were isolated from pools of NLF from 4 groups, control, CRS, asthma and asthma/CRS, and were analysed using quantitative proteomics. Ingenuity pathways analysis and gene ontology term finder were used to analyse the functions of the exosomal proteome. *Results*: (a) The use of exclusion lists allowed the identification of 41 and 89 additional proteins in respective pool. Furthermore, 173 proteins gained new unique peptides in the second and third runs, thereby increasing confidence. In total, 604 proteins were identified in nasal exosomes, with one-third of the proteins being associated with immune-related functions. (b) In the disease groups, 59 of the immune-related proteins could be quantified. Several S100 proteins were down-regulated in the asthma/CRS groups compared to healthy controls. Mucins were up-regulated in both asthma and asthma/CRS but not in the CRS group when compared to healthy controls. *Conclusion*: Exclusion lists can be used to obtain a more thorough analysis of the exosomal proteome, which increases the opportunity to identify lower abundance proteins. Proteins associated with asthma susceptibility, such as mucins, were observed to be associated with exosomes of the asthma groups. The immunoregulatory S100 proteins were lower in the asthma/CRS patient which could reflect a weakened immune defence. This is the first study to analyse exosomes in the upper airways in disease, but also illustrates that identified proteins in the exosomes fraction may originate from secretions.

**Preliminary characterisation of post-translational modifications in protein constituents of circulating extracellular microvesicles**

S. Kreimer ^1^, A. Belov^1^, I. Ghiran^2^ and A.R. Ivanov^1^

^1^Barnett Institute of Chemical and Biological Analysis, USA; ^2^Beth Israel Deaconess Medical Center, USA

*Introduction*: Circulating extracellular microvesicles (cEMVs) shed by cells comprise a heterogeneous population of membrane-enclosed vesicles varying in size (20–1000 nm) and content. Fluctuations in cEMV attributes have been recently associated with physiological states and cellular functions. Thorough molecular characterisation of cEMVs is hindered by the complexity of their composition, high heterogeneity and low abundance in biological fluids. With these analytical challenges, little attention has been directed at profiling of post-translational modifications (PTMs) in cEMV proteins. PTMs are expected to influence the function and biological activity of cEMVs. This work is a pilot survey of proteomic and PTM profiles of cEMVs isolated from the media of cultured MCF-7 and red blood cells, and blood plasma. *Materials and methods*: cEMVs were isolated by multistage centrifugation and delipidated by liquid–liquid phase extraction. The proteins were digested in-solution, and an aliquot of each sample was subjected to phosphopeptide enrichment by solid phase extraction on TiO_2_. Ultralow nanoflow liquid chromatography (LC) coupled with data-dependent tandem mass spectrometry (MS) was used to analyse both phosphopeptide-enriched and phosphopeptide-depleted fractions as well as the total digests. Database searches were performed using an array of variable PTMs, including methylation, acetylation, ubiquitylation and nitration. Initial glycosylation profiling was performed using multi-lectin enrichment and LC–MS. Comparative functional characterisation of cEMVs isolated from different sources was performed using proteomics data and gene ontology (GO) term enrichment analysis. *Results*: The direct ultralow flow rate LC–MS-based proteomic profiling of cEMVs allowed us to qualitatively and quantitatively characterise close to 1,000 proteins. The evaluated combination of separation, MS and bioinformatics approaches resulted in detection of unique and common proteins and their PTMs from cEMVs of different origin. The results of the preliminary comparative analysis of cEMV proteomes, PTMs and GO terms from various cEMV sources including disease/control samples will be presented. *Conclusions*: This preliminary investigation demonstrated thorough proteomic characterisation of cEMVs isolated from different sources and determined an array of PTMs potentially involved in functional activity of cEMVs.

**Comprehensive lipidome analysis of colorectal cancer-cell-derived exosomes**

C.J. Fhaner^1^, H. Ji^2^, R.J. Simpson^2^ and G.E. Reid ^1^

^1^Michigan State University, USA; ^2^Latrobe University, Australia

There is an increasing recognition of the role that cancer-cell-derived exosomes play in cell–cell signalling and in effecting cellular responses upon fusion with a target cell, including immune system evasion, tumour growth and metastasis. Exosomal membrane lipid compositions are expected to play pivotal roles in the uptake/fusion of exosomes with target cells, and as signalling molecules involved in cell targeting and functional cellular responses. To date, although disruption of lipid metabolism pathways is well known to play a role in the onset and progression of various cancers, exploration of the functional role of exosome-derived lipids in the onset and progression of the disease, or the potential of exosomal lipidome profile changes to serve as effective biomarkers, has not been performed. Here, we describe a strategy consisting of sequential functional-group-specific chemical derivatisation reactions coupled with high-resolution “shotgun” electrospray ionisation mass spectrometry (ESI-MS) and “targeted” tandem mass spectrometry (MS/MS) for the comprehensive identification, characterisation and quantitative analysis of >600 individual glycerolipids, glycerophospholipids, sphingolipids and sterol lipids between the primary adenocarcinoma cell line, SW480, its isogenic metastatic derivative, SW620, and their secreted exosomes. Statistically significant differences were observed between the exosome lipid profiles and their respective parent cells, particularly for alkyl ether-linked glycerophosphocholine, alkenyl ether-linked glycerophosphoethanolamine and sphingomyelin lipids that have previously been identified as being associated with cancer development, or that play known roles as mediators in a range of physiological and pathological processes. The results obtained may therefore provide important new insights into the role of exosomes and aberrant lipid metabolism in the onset and progression of metastatic colorectal cancer.

**Quantitative proteomic and miRNome analysis of the platelet releasate**

Feidhlim Dervin, K. Wynne, E. Madden, G. Cagney, D.J. Fitzgerald and P.B. Maguire

Conway Institute of Biomolecular & Biomedical Research, Dublin, Ireland

*Introduction*: Atherosclerosis is the primary cause of heart disease and stroke and is a complex progressive disease. The platelet releasate is a fraction highly enriched for platelet granular and exosomal contents and important in the initiation and progression of atherosclerosis. To date, ~700 proteins have been identified to be released from platelets; however, quantification of these proteins, as well as their specific attribution to platelet exosomes, has not yet been elucidated. Furthermore, although the total platelet microRNA (miRNA) content from healthy subjects has been characterised, it is unknown if miRNA is also released from platelets. As the majority of miRNA detectable in serum is concentrated in exosomes and as exosomes are present in and released from platelets, it is plausible that the platelet releasate containing exosomes could contain miRNA. *Methods*: Here, we performed high-throughput mass spectrometry (MS) analysis of differentially activated platelet releasate. Analysis of released miRNA was carried out utilising Qiagen's solution for whole miRNome analysis with a preamplification step. *Results*: In brief, the platelet releasate was isolated and confirmed to be free from microparticle contamination. The platelet exosomal population in the releasate was verified using electron microscopy and immunological techniques. Using a state-of-the-art Q-Exactive MS approach, combined with MaxQuant database analysis, we quantified the release of >1500 proteins, 250 of which were directly attributed to platelet exosomes. Furthermore, we confirmed that miRNA is released from platelets upon activation and performed total miRNA array analysis. *Conclusion*: We have established that the platelet releasate comprises both soluble and exosomal contents and have quantified a panel of released proteins, and for the first time, released miRNA. Such released platelet effectors represent key therapeutic targets in the initiation and progression of atherosclerosis.

**Comparative proteomic analysis of large oncosomes and smaller extracellular vesicles derived from amoeboid prostate cancer cells**

S. You, M. Morello, W. Yang, M. Zandian, V. Minciacchi, M. Rotinen, S. Morley, M.R. Freeman and D. Di Vizio

Cedars Sinai Medical Center, United States

*Introduction*: We recently demonstrated that a new class of EV released from the blebbing plasma membrane of phenotypically “amoeboid” tumor cells can be very large (1-10 μm) (Di Vizio et al., 2009). Shedding of such “large oncosomes” can be induced by overexpression of oncoproteins, and large oncosomes can be visualized in tumor tissues (Di Vizio et al., 2012). Large oncosome formation was also demonstrated as a result of silencing of the cytoskeletal regulator Diaphanous related formin 3 (DIAPH3) through an ERK-mediated pathway (Hager, Morley et al., 2012). Here we used a quantitative proteomics approach to: 1) determine whether intracellular loss of DIAPH3 is reflected in proteomic composition of EV; 2) identify large oncosome protein content on a proteome scale; and 3) identify alterations induced in prostate cancer cells exposed to EV. *Material and Methods*: Large oncosomes and EV were isolated by differential centrifugation. The quantitative LC-MS/MS SILAC method was performed on whole cell lysates and different types of EV preparations. *Results*: We identified 2074 intracellular and 1542 EV proteins (FDR<0.05), with 354 EV-specific proteins. A significant enrichment in proteins involved in cell adhesion and migration was found in EV from DIAPH3-silenced cells in comparison with EV from control-transfected DU145 cells. Comparative proteomic analysis revealed that the protein content of large oncosomes and smaller EV significantly overlaps (>90%), and Gene Ontology (GO) analysis highlighted, in both cases, strong associations with cell cycle regulation, vesicle-mediated transport, and cell motility. We identified 79 proteins enriched in large oncosomes (FDR<0.05), including fibronectin, known to play a role in EV-induced transformation of fibroblasts. Canonical exosome markers were enriched in small EV in comparison with large oncosomes. In DU145 cells treated with EV, we found a strong association with lipid metabolism and cell adhesion. *Conclusions*: This is the largest quantitative proteomic analysis of tumor-derived microvesicles and the first comprehensive proteomic analysis of large oncosomes. We conclude that large oncosomes carry cargo proteins that reflect the malignant nature of the donor cell and may provide a source of biomarkers.

**Quantitative proteomic analyses of extracellular vesicles, membrane and cells in colorectal cancer cells**

D.S. Choi, D.K. Kim, Y.K. Kim and Y.S. Gho

Department of Life Science, Pohang University of Science and Technology, Gyeongbuk, Republic of Korea

*Introduction*: Mammalian cells including cancer cells secrete extracellular vesicles (EVs), the nanosized bilayered proteolipids. Using high-throughput proteomic analyses, several thousand vesicular proteins have been identified. The potential roles of these vesicular proteins in biogenesis and pathophysiological functions have been proposed. However, the sorting mechanism(s) of vesicular proteins during biogenesis and the relationship between vesicular proteins with cellular or membrane proteins remain unknown. *Materials and methods*: Label-free quantitative proteomic analyses were conducted among cells, membranes and EVs derived from human colorectal cancer cells (SW480). Tryptic peptides were separated by OFFGEL fractionation and analysed by LC-MS/MS. Abundance of proteins was calculated by label-free quantitation based on spectral counting. Gene Ontology analyses were performed via DAVID database. Results: We identified 2442, 2360 and 1410 proteins from SW480 cells, membranes and EVs, respectively. Gene Ontology analyses showed that the proteomes among cells, membranes and EVs are quite different. Vesicular proteins are mainly derived from cytosol, cytoskeleton, endosome and plasma membrane rather than other cellular compartments such as nucleus and mitochondria. Based on the comparison between the estimated abundance of proteins, we identified 112 cell-, 108 membrane- and 71 EV-enriched proteins. Cytoskeleton, Ca-regulated proteins and enzymes abundantly exist in both cells and EVs. Transporters, tetraspanins and integral membrane proteins abundantly exist in both membranes and EVs. Moreover, endosomal proteins and well-known vesicular marker proteins including CD9, CD81 and 14-3-3 proteins were only enriched in EVs. *Conclusions*: This study suggests that cellular proteins are specifically sorted into EVs. This information would enhance the understanding of the pathological function of EVs and protein sorting mechanism(s) during EV biogenesis

**Characterization of cancer exosomes contaning prominin-1**

Aurelio Lorico ^1^, J. Mercapide^1^, F. Anzanello^1^, M.R. Pope^2^ and G. Rappa^1^

^1^Roseman University of Health Sciences, United States; ^2^University of Iowa, United States

Development of effective anti-cancer strategies based on prevention and targeting of metastatic disease is of high priority for most types of cancer. Tumor-derived exosomes have been recently implicated in the metastatic process. We previously reported that prominin-1 had a pro-metastatic role in melanoma cells (Rappa et al., Stem Cells 26, 3008, 2008) and that microvesicles released from metastatic melanoma cells expressed high levels of prominin-1 (Rappa et al., Exp Cell Res 319, 810, 2013). With the goal to explore the mechanisms that govern proteo-lipidic-microRNA sorting in cancer exosomes and their potential contribution(s) to the metastatic phenotype, we here employed prominin-1-based immunomagnetic selection in combination with filtration and ultracentrifugation to purify prominin-1-expressing exosomes (prom1-exo) from melanoma and colon carcinoma cells. Prom1-exo contained 154 proteins, including all of the 14 proteins most frequently expressed in exosomes, and multiple pro-metastatic proteins, including CD44, MAPK4K, GTP-binding proteins, ADAM10 and Annexin A2. Their lipid composition resembled that of raft microdomains, with a great enrichment in lyso-phosphatidylcholine, lyso-phosphatidyl-ethanolamine and sphingomyelin. Micro-RNA profiling revealed 49 species of micro-RNA present at higher concentrations in prom1-exo than in parental cells, including 20 with cancer-related function. Extensive accumulation of prom1-exo was observed 3 h after their addition to cultures of melanoma and bone marrow-derived stromal cells (MSC). Short-term co-culture of melanoma cells and MSC resulted in heterologous prominin-1 transfer. Exposure of MSC to prom1-exo increased their invasiveness. Our study supports the concept that specific populations of cancer exosomes contain multiple determinants of the metastatic potential of the cells from which they are derived.

**Parallel Late-breaking abstract Oral Sessions I-III 18:00-18:45**

**Late Breaking Oral Session I (Imperial Ballroom) April 18**

**18:00-18:45**

**Flow cytometric analysis of individual microvesicles and virions**

A. Arakelyan^1^ , W. Fitzgerald^2^, L. Margolis^2^ and J.C. Grivel^2^

^1^National Institute of Child Health and Development, United States; ^2^Eunice Kennedy Shriver National Institute of Child Health and Human Development, United States

Microvesicles or small viruses with a diameter of 30–200 nm are well outside the light scattering detection range of most commercial cytometers. Here, we report on a technology, which allows the detection and characterization of antigens on individual microvesicles and virions using multi-colour flow analysis on commercial cytometers. The technology is based on capturing microvesicles/virions with nanoparticles (MNPs) coupled to antibodies specific for microvesicles or viral antigens, staining microvesicles/virions with monoclonal antibodies, separating the formed MNPs/microvesicles/virion complexes from unbound antibodies in a high gradient magnetic field. The stained complexes are then acquired on a flow cytometer set to trigger on fluorescence. Using HIV-1-specific-MNPs and microvesicles-specific-MNPs we studied the expression on HIV-1 virions of two cellular antigens, HLA-DR and LFA-1, and we characterized microvesicles that contaminate viral preparations. We found that, HIV virions are heterogeneous regarding their incorporation of these two markers and that, as previously reported, HIV virions exclude CD45. On the other hand, CD45 together with CD81 and CD63 are found on microvesicles and exosomes.

**Novel assay platform of poly(A)+ RNA quantification in exosomes and extracellular vesicles**

Masato Mitsuhashi

Chief Scientific Officer, Hitachi Chemical Research Center, Inc., Irvine, USA

*Introduction*: Exosomes and extracellular microvesicles (EMV) contain mRNA from mother cells, and are released into various biological fluids, such as plasma, urine, saliva, cerebrospinal fluid, etc. Thus, by characterizing and quantifying such mother cell-specific mRNAs in EMV, mother cell's information can be obtained non-invasively. However, because mRNA is extremely fragile and low abundant, conventional methods are not suitable for mRNA analysis in EMV. Here we introduce a novel assay platform of EMV collection and mRNA analysis. *Materials and methods*: We developed a highly efficient EMV capture membrane. By inserting it into 96-well filterplates, EMV is collected from any biological fluid in a standard bench-top centrifuge in a high throughput fashion. For large volume samples, such as urine and cell culture media, we also developed a unique device, which accepts 10 mL samples. After centrifugation, EMV capture membrane can be removed and inserted into a bare frame of the filterplate. EMV is then lysed on the membrane, and the resultant lysate is transferred to a 96-well oligo(dT)-immobilized microplate for poly(A)+ RNA (=mRNA) isolation, followed by cDNA synthesis and gene amplification. *Results*: The system was successfully used in a variety of clinical research. For a urine application, glomerulus-, proximal tubule-, distal tubule-, and collecting duct-specific mRNAs were detected and corresponded to the biopsy results in patients with kidney transplantation (presented at ATC, 2012). We also identified mRNA biomarkers of diabetic nephropathy (presentation at ASN, 2012). For a plasma application, erythroblast-, myeloblast-, and megakaryocyte-specific mRNAs were detected in plasma EMV, which were early markers of bone marrow (BM) recovery after BM transplantation (presentation at EBMT, 2012). *Conclusions*: The assay platform we developed was easy-to-use, high throughput, and sensitive enough to detect various disease-related poly(A)+ mRNAs in various clinical specimens.

**A parameter independent method of particle quantification using nanoparticle tracking analysis**

A.M. Malloy

NanoSight Ltd, United Kingdom

*Introduction*: Nanoparticle Tracking Analysis has become an increasingly popular methodology for measuring size and concentration of extracellular vesicles. Various groups have tested the applicability of the technology in measuring populations of vesicles. Whilst particle size measurement by NTA is absolute, research has demonstrated the need for standardisation of measurement procedures in order to obtain accurate concentration measurements using the technology. This paper will detail an improved methodology for particle quantification, resulting in vesicle quantification being independent of measurement parameters and sample type. *Results and Conclusion*: Measurement of calibration standards of various sizes and material types shown to produce consistent particle concentration measurements irrespective of size, material type, dilution and user parameters.

**Late Breaking Oral Session II (Plaza Ballroom) April 18**

**18:00-18:45**

**Glycoproteins and glycans from exosomes of ovarian carcinoma cells**

J. Batista^1^, S. Jorge^1^, C. Escrevente^1^, S. Kandzia^2^ and J. Costa^1^ *

^1^Laboratory of Glycobiology, ITQB-UNL, 2780-157 Oeiras, Portugal; ^2^GlycoThera GmbH, 30625 Hannover, Germany. *jcosta@itqb.unl.pt

*Introduction*: Exosomes have characteristic protein and lipid compositions, and recent studies have shown that they also have specific glycoprotein profiles and glycan signatures. Here, we have used proteomics and glycomics techniques to characterize glycoproteins and glycans from exosomes of ovarian carcinoma cells. *Material and methods*: Exosomes were collected by ultracentrifugation of the supernatants of ovarian carcinoma SKOV3 and OVM cells, and were further purified by sucrose gradient centrifugation. Glycoproteins have been analysed by lectin blotting and immunoblotting. N-glycans have been released with peptide N-glycosidase F, analysed by HPAEC-PAD, by NP-HPLC after labeling with 2-aminobenzamide and by MALDI-TOF and LC-ESI-TRAP mass spectrometry. *Results*: Specific glycoprotein profiles have been found for the exosomes with the Maackia amurensis, Sambucus nigra and concanavalin A lectins in comparison with microsomal fraction and plasma membrane. Furthermore, sialoglycoproteins enriched in exosomes have been identified by peptide mass fingerprinting, immunoblotting and immunoprecipitation. Concerning the N-glycans distinct profiles were also found for exosomes. Among several differences, exosomes had higher levels of sialylated glycans whereas the microsomal fraction was enriched in high mannose glycans. *Conclusions*: This work provides detailed information about glycoprotein and N-glycan composition of exosomes from ovarian cancer cells. Furthermore, it opens novel perspectives to further explore the functional role of glycans in the biology of exosomes.

**Hodgkin cells release microvesicle-associated CD30 to facilitate crosstalk with CD30L-positive immune cells in the tumor microenvironment**

M. Dams, H.P. Hansen and E. Pogge von Strandmann

University Clinic Cologne, Germany

*Introduction*: Hodgkin lymphoma (HL)-affected lymphoid tissue contains only a few disseminated tumor cells, which stimulate immune cells in the tumor microenvironment. The interaction between tumor cells and immune cells does not result in tumor rejection but rather supports tumor growth. In vitro data suggest that CD30-CD30 ligand interaction plays a key role in this scenario. CD30 is selectively expressed on tumor cells and the membrane-anchored CD30 ligand on immune cells. Because in HL tissue, tumor cells are generally not found in direct proximity to CD30 ligand positive immune cells, we investigated the role of released membrane vesicles for the CD30-CD30 ligand interaction between cells. *Material and methods*: CD30+ microvesicles from HL cell lines were studied by cryo and immune electron microscopy. Vesicle targeting to CD30L+ immune cells was studied in vitro by confocal microscopy and functional assays. Vesicle release was confirmed in 3D culture and in primary tumor tissue using HL-affected lymph nodes. *Results*: HL cell lines released the soluble ectodomain (sCD30) by shedding and membrane-anchored CD30 on microvesicles. The latter were heterogeneous and ranged in diameter from exosome-like 40-100 nm to 1000 nm microvesicles predominantly with a buoyant density of 1.075-1.115 g/ml in sucrose gradients. In contrast to sCD30, microvesicle-anchored CD30 as well as immobilized CD30 on artificial particles caused a CD30-dependent release of IL-8 in CD30 ligand expressing granulocytes. In 3D co-culture, the vesicles were released and guided by a network of tumor cell-derived protrusions/cytonemes and caused a polarization of CD30L-positive recipient cells. Such CD30 vesicle-decorated network was also found in HL tissue. *Conclusions*: This tubular/vesicular network might contribute to tumor cell communication with distant infiltrating immune cells to contribute to the proinflammatory HL microenvironment.

**Ciliated micropillar array for exosome isolation**

Zongxing Wang^1^ , H.J. Wu^2^, B. Godin^2^, J. Zhang^1^ and X. Liu^2^

^1^The University of Texas at Austin, United States; ^2^the Methodist Hospital Research Institute, United States

*Introduction*: Intact exosomes isolation from raw biological fluids remains a great challenge. Such fluids typically contain high abundance of proteins, protein aggregates and cell debris with similar biophysical characteristics as exosomes. In the conventional procedures to isolate exosomes, the recovery rate is low, the contamination is high, and the recovered exosomes may be damaged. In this presentation, we develop the fabrication protocols for novel ciliated micropillars (the micropillars with porous silicon nanowires on the sidewalls) as a convenient microfluidic tool for isolating exosomes from complex biological samples. *Material and methods*: We fabricate the prototype microfluidic devices consisting of ciliated micropillars for simultaneously multi-scale filtration of biofluids. The ciliated micropillar array can interact with biological fluids at multiple length scales: 1) cells are depleted from the micropillar array owing to the sub-micron inter-pillar spacing; 2) submicron cellular debris is excluded by the nanowire forest (30-200 nm inter-nanowire spacing); 3) proteins and other molecules pass through the inter-nanowire spacing without being captured; 4) exosomes are trapped in the interstitial sites within the nanowire forest. *Results*: Using liposomes as a model vesicle, we demonstrate that the prototype microfluidic devices preferentially trap specifically sized liposomes, while filtering smaller proteins and larger nanoparticles with dimensions on the order of cellular debris. Moreover the trapped liposomes can be release by dissolving the porous silicon nanowires in PBS buffer, thus allowing for their intact and highly purified recovery. A sample consisting real exosomes has also been tested. *Conclusions*: We not only provide a microfluidic solution for the rapid and intact exosome isolation, but also contribute the protocols for fabricating silicon nanowires on 3D structures which will find broad applications.

**Late Breaking Oral Session III (Georgian Room) April 18**

**18:00-18:45**

**Circulating small-size microparticles as indicators of worsening status in patients with systolic heart failure. possible implication in the ischemic response**

Silvia Montoro-Garciaa^1^ , E. Shantsila^1^, B.J. Wrigley^1^, F. Marin^2^ and Y.H. Lip^1^

^1^Centre for Cardiovascular Sciences, University of Birmingham, United Kingdom; ^2^Department of Cardiology, Hospital Universitario Virgen de la Arrixaca, Murcia, Spain

*Background*: Circulating microparticles (MPs) have been implicated in different disorders, but scarce data are available on their role in systolic heart failure (HF). However, the source and routes of release of small-sized MPs (sMPs) is only partially characterised in clinical samples due to technological limitations of their assessment. This study assesses for the first time the clinical and biological relationship of sMPs in different forms of ischemic and systolic HF and, to investigate their relation to markers of inflammation and repair. *Method*: A total of 49 patients with ischemic acute HF (AHF) and 39 patients with stable HF (SHF) were enrolled and compared to 25 stable coronary artery disease (CAD) patients. Annexin V-binding sMP (sAMPs), platelet CD42b+ sMP (sPMP) and endothelial CD144+ sMPs (sEMPs) counts were determined by high-resolution flow cytometry (Apogee A50, Apogee Systems). Different monocyte subpopulations and expression of inflammatory receptors (interleukin-6 receptor [IL6R] and Toll like receptor-4 [TLR4]) and pro-reparative scavenger receptor CD204 and vascular cell adhesion molecule-1 [VCAM-1] were analysed by in a BD FACSCalibur, Becton Dickinson). *Results*: sEMP were lower in both forms of HF (p = 0.008). Increased sAMP counts were present in AHF (p = 0.017) and severe HF (NYHA III-IV) as compared to disease controls (p = 0.013). sPMP counts positively correlated with EF (p = 0.006) in patients with AHF, and sAMP with IL-6 levels in SHF patients (p = 0.034). sAMP counts in acute phase strongly correlated with TLR4 expression in the three subsets of monocytes (all p < 0.01), although no correlations were observed in SHF nor CAD patients. Moreover, CD204 and IL6R expression were negatively correlated with sAMP in SHF (all p < 0.05). *Conclusions*: Circulating sAMPs in the acute phase might be alternative markers of a worsening state adding to the evidence for sMP involvement in cardiac reparative processes and highlighting underpinnings of pathophysiology.

**Immunomodulatory role of exosomes in sarcoidosis**

Khaleda Rahman Qazi, A. Eklund, J. Grunewald and S. Gabrielsson

Karolinska Institutet, Sweden

*Introduction*: Sarcoidosis is an inflammatory granulomatous disorder characterised by accumulation of polarised Th-1 type CD4 + -T cells, macrophages, and immune-effector cells within affected organs. The disease affects multiple organs, most commonly the lung. We have shown that exosomes from the bronchoalveolar lavage fluid (BALF) from sarcoidosis patients can be proinflammatory, but the mechanism behind this is yet to be explored. In order to investigate the mechanism of action of exosomes in sarcoidosis we first aimed for extensive characterisation of BALF exosomes by iTRAQ based proteomics assay which facilitates the comparative quantification of proteins. *Materials and methods*: BALF exosomes from 15 patients and 5 healthy controls were subjected to enzymatic digestion to generate peptides followed by labelling with different iTRAQ reagents and analysed by LC-MS/MS. To validate the accuracy of relative quantitation of some of the proteins of interest, immunoisolation with FACS, ELISA and western blot were performed. *Results*: More than 1500 distinct proteins were identified on the BALF exosomes, some of which displayed significant upregulation in sarcoidosis patients. Some of these are known to be associated with inflammation. All those proteins represent a broad spectrum of functional classes as complement proteins (C1-C9), complement regulators (factor H, I, vitronectin, CD55,CD59), vitamin D- and LPS-binding protein, serum proteins, enzyme cofactors, proteases and their inhibitors. Importantly, the expression of all the complement components was upregulated in the exosomes from patients. Conversely CD55 and CD59 levels were higher in the exosomes from healthy controls which was validated by immunoisolation, detected by FACS and ELISA. *Conclusions*: A large portion of proteins and their differential expression are identified for the first time from BALF exosomes from patients, which pave the way for investigating the role of exosomes in sarcoidosis.

**Engineering exosome-cargo molecule interactions to interrogate and direct the loading of RNA**

J.N. Leonard and M. Marcus

Northwestern University, United States

*Introduction*: To date, the mechanisms by which a subset of cellular proteins and RNAs are sorted into exosomes are poorly understood. To better understand exosomal loading in normal and disease conditions, and to enable effective exosome-based therapeutics, we are developing novel biochemical tools for interrogating and directing the incorporation of specific RNA cargo molecules into exosomes. *Materials and methods*: We have developed a system for engineering exosomal membrane proteins that bind to defined “packaging” sequences within engineered RNA cargo molecules. Using this system, we investigated whether these interactions conferred enhanced loading of engineered RNA cargo molecules into exosomes derived from human HEK293FT cells. *Results*: We demonstrated that using our engineered packaging system, we can direct the incorporation of specific proteins and RNA into exosomes. We also identified RNA motifs and features that significantly impact loading in a manner that cannot be explained by simple bulk flow or mass action driving forces. These results suggest that novel active sorting mechanisms and/or biophysical constraints dramatically modulate RNA loading into exosomes. *Conclusions*: These investigations establish the feasibility of engineering exosomes to incorporate specific luminal cargo molecules. Because this approach probes the limits of exosomal loading, it complements the characterisation of natural exosomal loading of endogenous RNA. The approach we describe may be extended to probe and modulate both incorporation and delivery of a wide range of cargo molecules. Each of these investigations serves to both improve our understanding of natural exosome biology and enable translation of this understanding into novel therapeutic capabilities.

**Poster Sessions III-IV April 18 8:30-17:30**

**Poster Session III (Arlington-Berkeley): Bone/Stem Cells April 18**

**Chair: *J. Aliotta and P. Kurre***

**Interactions between mesenchymal stem cells and endothelial cells via microparticles**

T.P. Lozito and R.S. Tuan

Center for Cellular and Molecular Engineering, University of Pittsburgh, Pittsburgh, PA, USA

*Introduction*: Tightly associated with blood vessels in their perivascular niche, human mesenchymal stem cells (MSCs) closely interact with endothelial cells (ECs). Microparticles (MPs) are membrane-derived vesicles released into the extracellular environment by various cell types and are capable of intercellular signalling. As biomolecular shuttles, MPs also transfer proteins and RNA from one cell to another. Here, we characterise interactions between MSCs and ECs via MPs in vitro. *Materials and methods*: MPs were isolated from serum-free medium conditioned by human microvascular ECs (HMEC-1) for 2h. After clearing of cell debris, MPs were isolated as ultracentrifugation pellets (2h, 100,000×g). Fluorescently labelled MPs were prepared from ECs labelled with DiI (Invitrogen), and GFP-containing MPs were isolated from ECs transfected with CMV-GFP lentivirus. MSCs were treated with 100 µg/ml MPs or vehicle controls, and analysed for MP uptake (live cell confocal microscopy and spectrofluorimetry), proliferation and migration (Roche xCELLigence System) and cytokine release (Western blotting). *Results*: DiI-labelled MPs fused with MSCs, transferring the fluorescent dye over the MSC surface. GFP was transferred to and retained in MSCs incubated with GFP-MPs, but not free GFP. MP treatment significantly increased MSC proliferation, migration and CCL2 secretion compared to vehicle controls. *Conclusions*: Only MP-associated proteins were taken up and retained by MSCs, suggesting a layer of cellular specificity in which MP biomolecules, but not secreted factors, are shuttled between MSCs and ECs. EC MPs also activated MSC behaviour, including cytokine production, proliferation and migration, suggesting that MP may functionally regulate MSCs through several mechanisms. Support: NIH TR000532-01; DoD W81XWH-10-2-0084; Commonwealth of PA SAP 4100050913.

**Microvesicles derived from mesenchymal stem cells: potent organelles for induction of tolerogenic signalling**

Aram Mokarizadeh, Nowruz Delirezh, Ahmad Morshedi, Ghasem Mosayebi, Amir-Abbas Farshid and Karim Mardani

Department of Immunology, Faculty of Medicine, Kurdistan University of Medical Sciences, Sanandaj, Iran

Generation and maintenance of immunological tolerance is a pivotal aim in the field of autoimmunity. Regulatory molecules of Programmed death ligand-1 (PD-L1), galectin-1 and TGF-β are described as key mediators of peripheral tolerance that actively suppress auto-reactive cells and inhibit their mediated tissue damages. Accordingly, biological intervention in host immune system for induction of peripheral tolerance is pivot to many of the recent studies. Mesenchymal stem cell-derived microvesicles (MVs) are viewed as potential mediators to shed peripheral tolerance toward auto-reactive cells via bearing of tolerogenic molecules. Here, MVs were isolated from mesenchymal stem cell (MSC) cultures’ conditioned medium. They were explored for the expression of PD-L1, Galectin-1 and membrane-bound TGF-β through flow cytometry. The immunoregulatory effects of MVs on splenic mononuclear cells (MNCs) derived from experimental autoimmune encephalomyelitis (EAE) affected mice were investigated using MTT assay, ELISA and flow cytometry. MVs derived from MSCs expressed PD-L1, galectin-1 and membrane-bound TGF-β. MVs exhibited the potential to inhibit auto-reactive lymphocyte proliferation and also the potency to promote them to secrete anti-inflammatory cytokines of IL-10 and TGF-β. Interestingly, inducing inflammatory setting on MSCs revealed the enhancing regulatory effects of MVs via increased expression of some regulatory molecules, specific.

**Characterisation of exosomes from human-induced pluripotent stem cells (hiPSCs) and dendritic cells differentiated from hiPSCs (hiPS-DCs)**

Y. Lee ^1^, A. Leishman^1^, P. Sachamitr^1^, T. Davies^1^, S. EL Andaloussi^2^, P.J. Fairchild^1^ and M.J.A. Wood^1^

^1^University of Oxford, United Kingdom; ^2^Karolinska Institutet, Sweden

*Introduction*: Exosomes are released by most cells into the extracellular environment. The protein and RNA content of these vesicles are unique to their parental source and may be transferred as a means of communication between cells, both locally and at a distance. Here, we identified and characterised exosomes released from human-induced pluripotent stem cells (hiPSCs) and dendritic cells differentiated from hiPSCs (hiPS-DCs). *Materials and methods*: hiPSCs were reprogrammed from fibroblasts and subsequently differentiated down the DC lineage over a period of 24 days. Half of the hiPS-DCs were then matured using a cocktail of cytokines. Exosomes were isolated from conditioned media collected from these cultures via ultracentrifugation and characterised using Western blotting (WB), nanoparticle tracking analysis (NTA), electron microscopy and sucrose density centrifugation. *Results*: NTA analysis showed that very few particles could be isolated from hiPSC cultures. Exosomal markers such as CD9, Alix and Flotillin-1 only emerged from hiPS-DC cultures late in the differentiation process (Day 17–24). NTA analysis showed that our hiPS-DCs release high number of particles (~1011) with a mode size of 150 nm. Separation on discontinuous sucrose gradients further proved that these vesicles floated at a density of 1.11–1.15 g/cm^3^. Immature and mature DCs produce similar concentrations of particles. However, exosomes obtained from the immature DCs float at a slightly broader range (1.11–1.21 g/cm^3^) than those derived from the mature DCs (1.13–1.19 g/cm^3^). *Conclusions*: Our data show that hiPS cells release only few exosomes. In contrast, a substantial yield of exosomes can be isolated after differentiating the hiPSCs into DCs. These hiPS-DC exosomes could potentially be exploited for immunotherapy and gene therapy purposes.

**Induction of pluripotent stem cells by ES artificial vesicle**

J.H. Kim ^1^, D.Y. Jeong^1^, S.C. Jang^2^, Y.S. Gho^2^ and J.S. Park^1^

^1^School of Interdisciplinary Bioscience and Bioengineering, POSTECH, Pohang, Republic of Korea; ^2^Division of Molecular and Life Sciences, Department of Life Science, POSTECH, Pohang, Republic of Korea

*Introduction*: Cell-based therapies including xenogenic and allogenic transplant turned out immunogenic, and an autologous cell can be a new strategy. At this point, induced pluripotent stem cell (iPS) has been a focus of many cell-based therapeutic applications. One of the well-known methods is embryonic stem (ES) cell extract injection into somatic cell. Similar to ES cells, the injected somatic cells developed into 3 germ layers in vivo. However, during the injection, permeabilisation process is used, which induces somatic cell damage; ES cell extract-preparing step requires detrimental dilution by lysis buffer as well as harsh conditions such as homogeniser and sonicator. We assumed that if ES cell extract is injected without those damaging step, it will be possible to induce dedifferentiation more efficiently. In this study, we used artificial vesicles derived from ES cell for extract delivery. After treating the artificial vesicles to somatic cells, we found the recipient expressed pluripotent RNA markers. *Materials and methods*: To make artificial vesicles, 129S2/SvPas ES-D3 cells were penetrated into small size filter, and artificial vesicles were separated by ultracentrifugation. 100 μg/ml artificial vesicles were treated to somatic cell as soon as they were made. *Results*: Artificial vesicles had 60–80 nm and ES factors were tightly enveloped in lipid bilayer. Their membrane protein phosphorylated cell growth related kinase in 10 min and were found in nucleus as well as cytoplasm just 6 h. Most of Oct-3/4 (pluripotent marker)-GFP somatic cells formed the colony-expressing GFP for 2–6 days. Average value of the period was higher than extract method that needs 35 mg/ml protein. The origin of colony was confirmed by single nucleotide polymorphism. *Conclusion*: Artificial vesicles require shorter time for inducing dedifferentiation, even if we use small amount of protein. The suggested method is simple and safer and will make a new era in iPS field.

**Annexin A1(+) Microparticles and IL-8 mediated the anti-inflammatory properties of apoptotic acute promyelocytic leukemic cells**

Hui-Chi Hsu ^1^, I.T. Li^2^, S.Y. Feng^3^ and W.H. Tsai^4^

^1^Taipei Veterans General Hospital, Taipei, Taiwan; ^2^ National Yang-Ming University, Taipei, Taiwan; ^3^Department of Physiology, School of Medicine, National Yang-Ming University, Taipei, Taiwan; ^4^Department of Respiratory Therapy, Taipei Medical University, Taipei, Taiwan

*Introduction*: Annexin A1 (AnxA1) originating from mature neutrophils and their microparticles (MPs) plays an important anti-inflammatory role in either the resolution phase of inflammation or in the all-trans retinoic acid (ATRA)-induced granulocytic differentiation in the acute promyelocytic leukemic (NB4) cells. However, the role of AnxA1 in the apoptotic ATRA-treated NB4 (ATRA-NB4) cells is still not clear. *Materials and methods*: The surface expression of Annexin V was determined in the ATRA-NB4 cells which were pretreated with ultraviolet (UV; 0–2000 mJ/cm^2^) 4h before the flow cytometric assay. UV-treated cells were co-cultured with NR8383 phagocytes for phagocytosis assay. Conditioning medium (CM) of UV-treated cells were collected to determine their adhesive activity and migratory activity. The levels of IL-8, MCP-1 and AnxA1-containing MPs [AnxA1(+) MPs] were determined in the CM. *Results*: The annexin V(+) apoptotic cells increased in a dose-dependent manner in the UV-treated ATRA-NB4 (UV-ATRA-NB4) cells (p<0.001), but not in UV-treated NB4 (UV-NB4) cells. Both UV-ATRA-NB4 cells and UV-NB4 cells were able to enhance the phagocytic activity of NR8383 cells in a UV-dose dependent manner (p<0.05 and p<0.01, respectively). In addition, a UV-dose dependent anti-adhesive activity and anti-migratory activity were observed in the CM of UV-ATRA-NB4 cells [CM(UV-ATRA-NB4)] (p<0.05 and p<0.05, respectively); however, this effect was not observed in the CM of UV-NB4 cells [CM(UV-NB4)]. Further studies demonstrated that the level of IL-8 decreased in an UV-dose dependent manner in the CM(UV-ATRA-NB4) (p<0.05); however, the IL-8 level had no significant change in the CM(UV-NB4). There was also no significant change in the level of MCP-1 in either CM(UV-ATRA-NB4) or CM(UV-BN4). Finally, the level of AnxA1(+) MPs increased in a UV-dosage dependent manner (p<0.05) in the CM(UV-ATRA-NB4); however, this was not observed in the CM(UV-NB4).

**Leukemic-microvesicles-bearing tissue factor implication in hypercoagulable state in acute promyelocytic leukaemia**

Damien Gheldof ^1^, F. Mullier^2^, N. Bailly^2^, J. Dognè^1^, B. Chatelain^2^ and C. Chatelain^3^

^1^Department of Pharmacy Namur Thrombosis and Hemostasis Center (NTHC)-Narilis, Belgium; ^2^Hematology Laboratory, CHU Mont-Godinne UCL, Belgium; ^3^Hematology, CHU Mont-Godinne UCL, Belgium

*Introduction*: Patients with haematological malignancy have a 28-fold increased risk of venous thromboembolism (VTE). Among patients with acute myelogenous leukaemia (AML), the 2-year cumulative incidence of VTE is 5.2%. The induction mechanism of a hypercoagulable state is not fully understood. Multifactorial aspects such as patients’ immobility, chemotherapy adverse effects or the overexpression of several procoagulant substances (i.e. tissue factor [TF]) by cancer cells are often evoked. Several studies strongly suggest that microvesicles (MVs) harbouring TF may have a procoagulant role in promoting VTE and possibly disseminated intravascular coagulation (DIC) commonly seen in acute promyelocytic leukaemia (APL) *Objectives*: The aim of this study is to assess the capacity of untreated APL cells to shed procoagulant MVs. *Methods*: APL cell lines (NB4 and HL-60) were cultured for 48h in liquid medium at 600,000 cells/ml. Cells and MVs were separated by filtrations (Millipore 0.1-0.22-0.45-0.65 µm). The procoagulant activity (PCA) was assessed by thrombin generation assay. Alternatively, MVs were incubated with anti-TF antibodies (10 µg/ml), with annexin V (0.5 µM) to assess the contribution of TF and phospholipids to the PCA. Alternatively, the cells were incubated with with HgCl_2_ (an activator of TF). *Results and discussion*: NB4 cells have a high PCA mainly triggered by MVs of size under 0.45 µm. Thus, NB4 cells spontaneously release MVs of various sizes, which can augment TGA. By using an anti-TF antibody (HTF-1) and annexin V, we confirm that the PCA of MVs is related to the expression of active TF and PL. Interestingly, we show that HL-60 cells have a weaker PCA since TF is mostly present in an inactive form. Moreover, HL-60 does not produce MVs<0.65 µm associated with PCA. *Conclusions*: MVs could have a predicting value for VTE and DIC in patients with acute promyelocytic leukaemia and could inform haematologists for the thrombosis prophylaxis.

**Chronic lymphocytic leukaemia-derived exosomes stimulate cells from bone marrow microenvironment**

E. Moussay ^1^, J. Paggetti^1^ and G. Berchem^2^

^1^CRP-Santé, Laboratory of Experimental Hemato-Oncology, Luxembourg; ^2^CRP-Santé, Laboratory of Experimental Hemato-Oncology CHL Luxembourg, Luxembourg

*Introduction*: Chronic lymphocytic leukaemia (CLL) is characterised by the accumulation of long-lasting, mature, but non-functional B lymphocytes in the blood and the primary lymphoid organs. Although CLL B cells can survive for long periods in vivo, cells are undergoing apoptosis quickly in vitro. This is strongly reduced in presence of bone marrow mesenchymal stem cells (MSCs) or endothelial cells. We recently reported the first profiling of circulating miRNA from plasma of CLL patients. We also noticed that primary CLL B cells can transfer in vitro vesicles to MSCs through 400 nm culture inserts. These observations prompt us to investigate whether CLL B cells secrete exosomes that could modify cells of the bone marrow microenvironment to produce tumour growth promoting factors locally to favour their own survival. *Methods and results*: We isolated, purified and characterised exosomes derived from CLL cell lines, primary cells culture supernatants and plasma from CLL patients. Proteins, mRNA and microRNAs contents were evaluated by high-throughput methods (LC-MS, microarrays) revealing in particular the presence of oncogenic molecules. In vitro, purified CLL-derived exosomes were found to enter rapidly in target cells (MSCs and endothelial cells) and to transfer proteins and miRNA. Exosomes can also be taken up ex vivo and in vivo by mouse bone marrow cells. CLL-derived exosomes activate key signalling pathways and modulate gene expression in target cells. Exosomes were also shown to increase MSC proliferation and migration of endothelial cells and MSCs. *Conclusions*: CLL-derived exosomes contain pro-oncogenic molecules and strongly affect key functions of MSCs and endothelial cells, which are critical components of the bone marrow microenvironment. Activation of these cells by CLL-derived exosomes could in return favour leukemic cells survival. The outcome of this work may lead to applications in both diagnosis and therapy development.

**Extracellular vesicles and their preferred target cell population**

J.M. Aliotta, A. Sorokina, M.S. Dooner, S. Wen, L.R. Goldberg, M. Deltatto, D.M. Adler, E. Papa, P.J. Quesenberry, M.G. Pereira, B. Ramratnam and E. Sears

Department of Medicine, Rhode Island Hospital, Warren Alpert Medical School of Brown University, USA

*Introduction*: Our group has shown that whole bone marrow (WBM) cells co-cultured with extracellular vesicles (EVs) from murine lung cells express pulmonary epithelial cell-specific proteins and mRNA. The mechanism for these observations is unknown. The exact WBM cell population affected by lung-derived EVs requires elucidation. Possible candidates include bone marrow-derived hematopoietic stem/progenitor cells and differentiated cells of a lymphoid, myeloid or erythroid lineage. By investigating the uptake of EVs and the subsequent modification of their host cells, we would obtain useful information for developing cell-based therapies. *Methods*: EVs were isolated from cell-free conditioned media made from lung cells as described by Théry et al. Lineage-depleted cells (Lin−) as well as differentiated cells of a lymphoid, myeloid or erythroid lineage were separated from murine WBM cells by flow cytometry. EVs were stained with PKH26 prior to co-culture with cells. After 48h of co-culture, cells were harvested and separated by flow cytometry based on their uptake of PKH26-labelled EVs. Sorted cell populations were then placed in secondary culture for additional time and analysed by RT-PCR for the expression of pulmonary epithelial cell genes. *Results*: All cell populations studied internalised EVs at varying degrees (6.9% of granulocytes, 11.5% of erythroid cells, 6.1% of B cells, 6.5% of T cells and 9.7% of Lin− cells). Cells that internalised EVs had significantly higher expression of pulmonary epithelial genes compared to WBM cells co-cultured without EVs. *Conclusions*: Many different bone marrow cell populations, including differentiated cells and hematopoietic stem/progenitor cells, internalise lung-derived EVs and subsequently express pulmonary epithelial cell genes in culture.

**Inhibition of TFPI increases the sensitivity of thrombin generation assay to procoagulant microvesicles**

D. Gheldof ^1^, F. Mullier^2^, B. Chatelain^2^, J. Dognè^1^ and C. Chatelain^3^

^1^Department of Pharmacy Namur Thrombosis and Hemostasis Center (NTHC)-Narilis, Belgium; ^2^Hematology Laboratory, CHU Mont-Godinne UCL, Belgium; ^3^Hematology, CHU Mont-Godinne UCL, Belgium

*Introduction*: Patients with cancer have a 7- to 10-fold increased risk of developing venous thromboembolism (VTE). Circulating microvesicles (MVs) could be a predictive biomarker for VTE in cancer. Thrombin generation assay (TGA) is a useful technique to detect procoagulant activity of MVs. However, TGA suffers from a lack of sensitivity due to the presence of tissue factor pathway inhibitor (TFPI) in plasma. *Aims*: To improve the sensitivity of TGA to tissue factor (TF) by limiting the interference of TFPI. *Methods*: Serial dilutions of MDA-MB231 cells were incubated for 45min at 37°C to generate MVs. Samples were then centrifuged and supernatants that contain MVs were used for TGA. Normal pooled plasma was incubated with inhibitor of TFPI or was diluted twice to decrease plasma level of TFPI. Lagtime was used as a surrogate marker of TGA to detect procoagulant activity of MVs. *Results*: (a) Inhibition of TFPI decreased twice the cell concentration needed for a significant reduction of lagtime and decreased 2.4-fold the intra-assay variability. (b) Plasma dilution had no impact on the TGA sensitivity when TGA was triggered by MVs derived from MDA-MB-231. *Conclusions*: Thrombin generation is a very sensitive method to study the procoagulant activity of TF-MVs. The sensitivity can be increased by inhibition of TFPI with specific monoclonal antibody against its Kunitz domain I. A two-time plasma dilution is an interesting cheaper alternative to study the procoagulant activity of MV by TGA with a good sensitivity, especially when low plasma quantities are available.

**Microvesicles-mediated trafficking of immunoglobulin free light chains**

G. Di Noto, L. Paolini and D. Ricotta

Universityof Brescia, Italy

*Introduction*: Plasma cell dyscrasias are immunosecretory disorders that can lead to haematological malignancies such as multiple myeloma (MM). Monoclonal immunoglobulin free light chains (FLCs) are present in the serum and urine of many patients with plasma cell diseases. FLCs producing cells and paraproteins themselves are able to induce different tissue damage. Studies demonstrate that exosomes can modulate immune-regulatory processes, set up tumour escape mechanisms and transfer physiological information; thus, we investigated if FLCs are processed via microvesicles in the blood stream. *Methods*: Serum samples from MM, amyloidosis (AL) and monoclonal gammopathy of undetermined significance (MGUS) patients were collected. We established an experimental protocol to test the release of microvesicles in endothelial and myocardial FLC-treated cells. The cell culture medium and serum samples were processed by utracentrifugation, density gradient separation and immunoaffinity capture beads. We analysed the microvesicles/exosomes profile with protein quantification, Western blot analysis and thin layer chromatography. *Results*: We show that after internalisation, FLCs are re-routed in the extracellular space via microvesicles/exosomes that could be re-internalised in contiguous cells. The isolated serum vesicles from MM, AL and MGUS patients contained FLCs and were strongly positive for Hsp70, annexin V and c-Src compared to MGUS and controls. *Conclusions*: In this study, we reveal a novel mechanism of FLCs processing that involves c-Src labelled extracellular vesicles. This evidences suggest a novel mechanism of c-Src activation during plasma cell dyscrasia and its involvement in proinflammatory events and tissue damage. The in vitro data are strongly confirmed by characteristics of in vivo-produced microvesicles. More strikingly, MGUS-derived microvesicles are different from those patients with MM or AL amyloidosis, suggesting a new prognostic tool.

**Quantitative proteomics of extracellular vesicles derived from human monocytic cells**

Y. Wang, N. Ismail, C.B. Marsh and M.G. Piper

Division of Pulmonary and Critical Care, The Ohio State University Wexner Medical Center, USA

*Introduction*: Previously, we quantified microRNA expression from human peripheral blood extracellular vesicles (EVs). We reported macrophage-derived EVs induce changes in gene expression to modulate monocyte survival and differentiation. We hypothesise proteins contained in the EVs may contribute to these functions. Using proteomic analysis, we characterised the proteins and their functions in the EVs. *Methods*: EVs were isolated from GM-CSF-derived macrophages. Proteins were extracted and processed from EVs. Quantified peptides were analysed in a HPLC system coupled to mass spectrometer. SEQUEST software was used to match MS/MS fragmentation spectra with sequences from the 2008 NCBI human database. GO SLIM software was used to categorise the proteins. Western blot was used to validate the proteins expression. To analyse proteins that facilitate microRNA packaging in the EVs, a modified IP to pull down RNA:protein complexes followed by qRT-PCR was used. *Results*: Proteomic analysis of EVs from GM-CSF-treated monocytes revealed the presence of 229 proteins and 21 proteins were presented in the EVs and not in the cells. Upon GM-CSF-treatment, we found an upregulation (>1.5 fold) of 28 proteins in the EVs. The majority of the EV proteins identified were of cytoplasmic and membrane origin. Predicted functions of the proteins included nucleotide, ion and lipid binding. Several proteins in the EVs, including heat shock, histone and cytoskeletal proteins, were validated. The EV proteins predicted to bind nucleotides in our proteomic analysis included hnRNPs and argonaute. We further show that these proteins were bound to miR-223 in the EVs and may function as chaperones to package microRNAs. *Conclusions*: Quantitative proteomic analysis provided a unique protein profile of EVs from human monocytic cells. Understanding the composition and function of these proteins may provide useful biomarkers and diagnostic tool in the host defense and inflammation

**Characterisation of extracellular vesicles in biological fluids without isolation**

J. Leung and E. Maurer-Spurej

University of British Columbia, Canada

*Introduction*: Cell-derived vesicles are present in most biological fluids and elevated concentration levels are often an indicator of disease. Standardised analyses of these particles have not been developed as testing is often complicated by variations between samples and interferences from other fluid components. Dynamic light scattering (DLS) is widely used for particle sizing and has been applied to suspensions of isolated extracellular vesicles. The aim of this study was to evaluate ThromboLUX, a DLS based instrument, to quantify and size microparticles (MPs) in plasma without the need for centrifugation or filtering. *Materials and methods*: Platelet concentrates were prepared by single-donor apheresis (n=9) or from pooled whole blood by the buffy coat (BC) method (n=10). In addition, a blood sample anticoagulated with EDTA was collected from each donor to obtain platelet rich plasma (PRP). The PRP and platelet concentrates were measured by ThromboLUX to examine the MP content and size distribution. The quantification of MP by ThromboLUX was compared to flow cytometry results. *Results*: ThromboLUX was able to detect the full size distribution of MP populations in plasma (particle diameters of 20–1100 nm) in the presence of platelets. The MP content measured directly from donor PRP samples highly correlated with the apheresis product (r=0.86). However, MP concentrations in donor samples were consistently higher, suggesting that the apheresis process does not collect all donor MP. The MP concentrations from ThromboLUX correlated significantly with concentrations determined from flow cytometry (r=0.99). *Conclusions*: These findings suggest ThromboLUX can be used as a diagnostic tool to detect and characterise extracellular vesicles in biological fluid samples without the need for isolation from other particles. For plasma samples, ThromboLUX can be used to pretest donors for MP, predict MP content of products or measure changes/differences in concentration.

**Poster Session III (Arlington-Berkeley): Capture April 18**

**Chair: *K. Witwer and S. Sharma***

**MARCKS effector domain as a curvature and lipid sensor**

Leslie Morton

Department of Chemistry and Biochemistry, USA

*Introduction*: Membrane curvature plays a vital role in cell signalling, endo- and exocytosis, membrane fusion and protein trafficking. It also characterises a primary feature of nanosized extracellular vesicles (EVs) exposing an overexpression of phosphatidylserine (PS). We report a peptide corresponding to the membrane protein myristoylated alanine-rich C-kinase substrate, MARCKS effector domain (ED), that selectively detects highly curved and phosphatidylserine (PS)-enriched membrane surfaces, including synthetic vesicles and EVs secreted from rats. Our hopes are to identify and develop potential probes to detect microvesicles based on both size (Ø = 0.03–1 µm) and PS-enriched surfaces. *Materials and methods*: Peptide synthesis, Lipid vesicle preparation, CD spec, Cosedimentation pull-down assay, Fluorescence enhancement/anisotropy, Ex vivo nanoparticle tracking analysis, In vivo staining of *C. elegans* gonads. *Results*: Our in vitro assays show MARCKS-ED preferentially targeting more highly curved vesicles, showing a stronger binding affinity for 30 nm vesicles relative to 400 nm vesicles containing PS. Our ex vivo results suggested the MARCKS-ED peptide does indeed target PS on secreted rat extracellular vesicles, proposing that the MARCKS-ED prefers highly curved vesicles in a complex, biological system. Our established *C. elegans* in vivo model confirmed that the MARCKS-ED peptide does select for cells exposing PS on the outer leaflet. *Conclusions*: In summary, our data show that MARCKS-ED can differentiate between different vesicle sizes with both synthetic phospholipid vesicles and rat-secreted extracellular vesicles. It further suggests that both the aromatic Phe residues and electrostatic interactions play a role in this curvature sensing behaviour. Currently, the MARCKS-ED peptide targets PS-expressed highly curved vesicles, which could be developed as a potential probe after further characterisation to detect exosomes, a new generation biomarker linked to metastasis.

**Magnetic beads for fast and reproducible isolation/characterisation of exosomes based on surface protein expression**

Axl Neurauter, B. Kierulf, A. Kullmann and K.W. Pedersen

Life Technologies, Norway

*Introduction*: Exosomes are small (30–120 nm) vesicles containing proteins, RNA and lipids, secreted by all cell types in culture and found in most body fluids. There is hope that miRNAs in circulating exosomes provide a screening tool for early detection of cancer. This will require access to highly specific, reproducible and automation friendly methods for exosome isolation/characterisation. The established standard for exosome isolation is ultracentrifugation. However, this method is not automation friendly and cannot discriminate between exosome subpopulations or other microvesicles. *Results*: Here, we present a versatile tool for exosome isolation/characterisation using Dynabeads^®^ with antibodies against classical exosome surface proteins (CD9, CD63 and CD81). Specific and reproducible exosome isolation and characterisation by flow cytometry was demonstrated for exosomes originating from a B-cell lymphoma and a colorectal cell line. The data were supported by Western blotting and immuno-electron microscopy. We also demonstrate that the microvesicles isolated by this method represent a pure fraction of exosomes. In addition, we are able to characterise cell-specific differences between exosomes from the 2 different cell types. *Conclusion*: We conclude that immunomagnetic separation is a rapid, easy to perform and reproducible method for characterisation of exosome fractions well suited for future automation needs. This method can also be tailored for isolation of cell specific exosomes (work in progress).

**EpiVeta: a coating agent that endows material surfaces with an affinity to EpCAM-expressing exosome**

Kazuhiro Hibino, K. Suga, M. Yoshida, T. Minamisawa, T. Sudo, S. Matsumura and K. Shiba

Japanese Foundation for Cancer Research, Japan

*Introduction*: EpCAM is known to be the surface marker of epithelial cells; it has been shown to be expressed on exosomes by certain cells. Because the increased expression of EpCAM in exosomes has been suggested among patients with certain cancer types, a methodology that differentiates mixtures of exosomes into EpCAM(+) and EpCAM(−) subpopulations is urgently required to develop diagnostic devices that use exosomes. Here, we introduce our system that coats the surfaces of various materials with peptide aptamers against EpCAM molecules. *Materials and methods*: A random copolymer of MPC and hydrophobic units with a carboxyl group were provided by the NOF Corporation. Conjugates between Ep114 and an MPC polymer were synthesised by employing click chemistry. *Results*: Our previous peptide phage experiments identified Ep114, a 12-mer peptide aptamer against EpCAM. Although in some cases, peptide aptamers lose their binding ability when they are detached from a phage body or transferred to foreign molecules, Ep114 shows strong and specific binding abilities vis-à-vis EpCAM when it is used as a synthetic peptide or displayed on a ferritin particle. To immobilise the Ep114 on the surfaces of various materials, we conjugated the peptide with an MPC (2-methacryloyloxyethyl phosphorylcholine) polymer that has been used as a coating agent to reduce the non-specific binding of biomolecules to the surfaces of materials. The resultant conjugant, EpiVeta, endows SiO_2_ and Au surfaces with an affinity against EpCAM; this was confirmed by the accessibility of anti-Ep114 antibody in physiological conditions. *Conclusions*: EpiVeta is a versatile coating agent that can endow various material surfaces with an affinity against EpCAM. By using EpiVeta, novel types of diagnostic and remedial devices for exosome medicine can be developed.

**Isolation of exosomes by bead-packed microfluidic chip**

Y.H. Heo, H.S. Song and J.Y. Kang

Korea Institute of Science and Technology, Republic of Korea

*Introduction*: Exosomes contain genetic information, such as DNA and RNA, and provide information about state of cells or tissues. Therefore, the analysis of exosomes offers clinical importance as biomarkers for disease diagnosis. However, conventional isolation methods including ultracentrifuge and magnetic immuno-isolation are considered as a bottleneck for the study of exosomes because those require large sample volume, long process time and intensive labour. In this work, we developed a method for rapid isolation of specific exosomes from the sample of small volume using bead-packed PDMS microfluidic chip. *Materials and methods*: Microfluidic chip was fabricated by soft lithography with the mould on silicon wafer and consists of bead-packing chamber and microfluidic channels. Beads were coated by anti-CD63 antibody. The beads were packed by filter. Exosomes were obtained from mouse P19EC by ultracentrifuge and then diluted in PBS. The suspension of 400 μl was flowed into the chamber and washed by PBS. The fluorescence images were photographed and the intensity was analysed. As a control, experiment with DOPC nanovesicle was carried out. The mRNAs in exosomes of the chamber was collected by the elution with Trizol. *Results and discussion*: When exosome suspension was concentrated to the packed beads, the ratio of remaining fluorescence after PBS washing was more than 80%, whereas that of DOPC nanovesicle was less than 50%. Exosomes were more selectively bound to beads than DOPC. The fluorescence of DOPC after washing, however, was also detected. The non-specific binding could be diminished by the modification of filter structure and surface treatment. Oct4, Nanog and α-actin were detected from the eluted solution of 100 μl. Further study on this chip will offer a simple sample preparation method for the isolation of exosome in small volume of samples such as blood, saliva and cerebrospinal fluid.

**Differentiating exosomes into subpopulations, using weak interactions between exosomes and material surfaces**

Mitsutaka Yoshida, K. Hibino, K. Suga and K. Shiba

Japanese Foundation for Cancer Research, Japan

*Introduction*: Exosomes are rich sources of information regarding the host cells that released them. However, bodily fluids contain mixtures of exosomes secreted from various cells, and methodologies that differentiate these exosomes into subgroups based on their characteristics are urgently required to develop diagnostic and therapeutic devices. Although an antibody-based methodology is promising in developing such devices, we are interested in the use of peptide aptamers for this purpose, given their versatility in conjugation with foreign molecules and their modulability in affinity and specificity. *Materials and methods*: HT-29 and HEK293 were used in the preparation of EpCAM(+) and EpCAM(−) exosomes, respectively. The immobilisation of Ep114 was determined through accessibility to anti-Ep114 antibody to the coated surface. *Results*: EpiVeta is a coating agent comprising MPC polymer and Ep114, an aptamer against EpCAM; we developed EpiVeta recently. We observed that Au and SiO_2_ can be coated by EpiVeta, whereas Teflon is not coated under the conditions that were used; this suggests that proper hydrophilicity is required for efficient coating. To develop an affinity column for the EpCAM-expressing exosome, we first tested Sephacry, Sepharose, TOYOPEARL and silica gel (63–200 nm), if they were tolerant to the polymer-coating conditions. Under the conditions used, only silica gel was able to pass the exosome after coating it with a MPC polymer (without Ep114). The effect of Ep114 on EpiVeta vis-à-vis the retention time for the silica column is now under examination. *Conclusions*: We showed that the surfaces of Au and SiO_2_ can be modified through the use of EpiVeta – a conjugate between Ep114 peptide and a MPC polymer – to have affinity to EpCAM; this has led to the development of a novel type of affinity column for isolating EpCAM(+) exosomes.

**LC purification of exosomes: way forward or dead end?**

J.Z. Nordin ^1^, Y. Lee^2^, O.P.B. Wiklander^1^, P. Vader^2^, C.I.E. Smith^1^ and S. EL Andaloussi^1^

^1^Karolinska Institutet, Sweden;^2^University of Oxford, United Kingdom

*Introduction*: Exosomes have gained increasing attention because of their involvement in important biological and pathological processes. Furthermore, these vesicles display therapeutic potential. Hence, optimising the protocol used for derivation of exosomes from cells is of utmost importance. The gold standard method to purify exosomes is by ultracentrifugation (UC). It is however, laborious, restricted by sample volumes and the yields are relatively poor. Spin filtration with size exclusion liquid chromatography (LC) fractioning is a more scalable and reliable method. *Materials and methods*: We compared UC, spin filtration and spin filtration with sequential LC fractioning for isolation of exosomes from cell culture media. The exosome samples were assessed by RNA and protein content analysis, Western blotting (WB), Nanoparticle tracking analysis (NTA) and electron microscopy. *Results*: The spin-filtered samples rendered higher yields as compared to the UC samples, according to WB bands of ALIX, CD9 and Tsg101, and higher particle counts on the NTA analysis. Total protein staining indicated an impurity in the spin-filtered sample, identified by mass spectrometry as a truncated form of albumin. The LC purification of spin filtered exosomes generated 3 fractions. Fraction 1 had strong bands for the exosomal markers and similarly high particle counts as the spin-filtered only sample. The truncated albumin was captured in fractions 2 and 3. The UC sample and fraction 1 only had traces of the truncated albumin left. *Conclusions*: By simple spin-filtration and sequential LC fractionation, high yields of exosomes can be purified from large media volumes. Hence, the LC purification method is promising, but needs further development to become the gold standard for exosome purification.

**Novel peptide with affinity for canonical heat shock proteins (HSPs) as a tool for capture and enrichment of extracellular microvesicles**

I.C. Chute ^1^ M. Caissie^1^, S. Griffiths^1^, S. Chacko^1^, M. Davey^1^, D. Barnett^1^, S. Melville^1^, S. Fournier^1^, M.V. Meli^2^, S. Lewis^3^, A. Ghosh^1^ and R.J. Ouellette^1^

^1^Atlantic Cancer Research Institute, Moncton, New Brunswick E1C 8X3, Canada; ^2^Department of Chemistry and Biochemistry, Mount Allison University, Sackville, New Brunswick E4L 1E2, Canada; ^3^New England Peptide, Gardner, MA, USA

*Introduction*: Extracellular microvesicles (eMVs) are a promising source material for future clinical applications, particularly as a minimally invasive means of detecting biomarkers used in diagnosis, monitoring of disease status and treatment efficacy. Current obstacles to the application of eMVs in the clinical setting include (a) efficiently isolating appreciable quantities of eMVs from various body fluids and (b) isolating eMVs of interest from mixtures of vesicular material originating from multiple cell types. The development of rapid and versatile disease-specific eMV isolation technologies will be critical for advancing clinical applications. Among the earliest identified protein markers for eMVs, heat shock proteins (HSPs) are well characterised and important in promoting cell survival in response to stressful conditions. Basal expression of HSPs in normal cells is generally low, but in cells afflicted with cancer or other pathological conditions, expression of HSPs is increased and localised to the surface of eMVs secreted by these cells. Here, we describe the discovery and validation of a peptide (Vn96) with specific affinity for canonical HSPs as a highly effective tool for the isolation and purification of HSP-containing eMVs from culture media and human body fluids. *Materials and methods*: eMVs isolated from cell culture media, urine and serum using Vn96 were characterised by nanoparticle tracking analysis (NTA), mass spectrometry, transmission electron microscopy, immunoblotting and atomic force microscopy. *Results*: Vesicles captured using the Vn96 affinity peptide display protein markers and physical characteristics accepted in the literature as indicative of eMVs. Vn96 demonstrates the ability to capture eMVs expressing HSP from urine, serum and cell culture media using centrifugation forces of 15,000 RCF for 10min. Yields of purified eMVs evaluated by protein content and NTA prove comparable to other established methods of eMV isolation. *Conclusion*: The efficiency of HSP affinity peptide (Vn96)-mediated capture of eMVs matches or exceeds currently accepted methods of eMV isolation while also providing greater specificity in capturing eMVs of particular clinical interest.

**Comparative omics of extracellular microvesicles isolated using Vn affinity peptides**

N. Crapoulet, S. Chacko, M. Caissie, S. Griffiths, D. Leger, I.C. Chute, M. Davey, D. Barnett, S. Melville, A. Kumar, S. Fournier, R.J. Ouellette and A. Ghosh

Atlantic Cancer Research Institute, Moncton, New Brunswick E1C 8X3, Canada

*Introduction*: We have engineered and validated a synthetic peptide (Vn96) with specific affinity for canonical heat shock proteins (HSPs) as a tool for rapid isolation and purification of HSP-containing extracellular microvesicles (eMVs). In most cancers, HSP expression is exceptionally high (chaperonopathies) and found on the surface of their secreted eMVs. Thus, our method isolates cancer specific-eMVs of increased clinical value and also compares favourably in terms of efficiency, cost and versatility. As part of the validation process for Vn peptides, we performed comparative genomic and proteomic characterisations of the eMV subsets obtained by Vn affinity protocols with those from established protocols such as ultracentrifugation and a commercial isolation reagent. The compatibility of Vn affinity technology with such downstream “omics” applications as next-generation sequencing (NGS) and mass spectrometry (MS) is essential for its ultimate use in the search for clinical biomarkers present in eMVs. *Materials and methods*: eMV isolates from conditioned media of breast cancer cell lines using Vn96 peptide were compared to those obtained by ultracentrifugation or using a commercial purification reagent. RNA samples isolated from eMVs were subsequently processed for NGS and also in parallel for hybridisation to human microarrays. Total protein from eMV isolates was purified and analysed by MS. *Results*: Analysis of the protein profiles of eMV isolates by MS demonstrated the presence of classical eMV markers in samples isolated by Vn96 and the similarities of these samples to those isolated by ultracentrifugation or commercial purification reagents. RNA profiles of eMV isolates were analysed by NGS and microarray, again showing similar patterns between the purification protocols. RNA and protein profiles were most consistent in the samples isolated from conditioned media. *Conclusions*: eMV subsets isolated using the Vn96 peptide are very similar and share many of the recognised eMV markers with those isolated by established methods at the genomic and proteomic level.

**Hydrostatic dialysis: a new method to enrich urinary vesicles for clinical analytics**

L. Musante, D. Tataruch, A. Benito Martin, D. Gu, G. Calzaferri and H. Holthofer

Dublin City University, Dublin, Ireland

*Introduction*: Since the first description of exosome vesicles in urine, new streams of research have targeted urinary vesicles as source of potential biomarkers. In addition to exosomes, other nanoparticles like exosome-like and membrane (shed) vesicles are released in the urine from all nephron segments and the urinogenital tract. The majority of currently applied isolation protocols for these vesicles include laborious centrifugation steps. To date, the efficiency of these approaches has not been investigated in respect to quantitative and qualitative yield analysis. Beyond the isolation methods, there are still important challenges to overcome for successful clinical application, especially for proteomic and transcriptomic analysis, which usually call for big volumes of urine not easily available in the clinical setting. *Materials and methods*: Urinary vesicles were first concentrated and dialysed by a simple hydrostatic dialysis system followed by ultracentrifugation. Enriched vesicles were characterised by Western blot and electron microscopy. *Results*: Applying this novel approach, we have gained several advantages: (a) Large volume of urine (200–400 ml) can be concentrated in a relatively short time. (b) The vesicle fraction can be concentrated up to 100 times. This meets the needs, for example, biobanking processes. (c) Introduction of dialysis step necessary to equalise all the urinary solutes under investigation. (d) A clinical pilot study has revealed that the minimum volume of urinary necessary to perform a proteomic analysis for a discovery phase is not less than 200 ml and 100 ml for a transcriptomic profiling. On the other hand, as low as 50 ml is needed for a validation analysis. Finally, 15 ml of urine routinely collected may be utilised for specific targeted approaches. *Conclusion*: A simple and versatile method to simplify and enrich vesicles from urine, which allows a novel approach for diagnostics, has been established.

**Urine exosome capture device to quantify glomeruli-, tubules-, and collecting ducts-specific mRNA as a discovery platform of diagnostic biomarkers**

T. Murakami and M. Mitsuhashi

Hitachi Chemical Research Center, Inc., Irvine, CA, USA

*Introduction*: Urinary exosomes and microvesicles (EMV) are promising biomarkers for renal diseases. Although the density of EMV is very low in urine, large quantity of urine can be easily obtained. Thus, we developed a unique filter device to absorb EMV from 10 ml urine, which is far more convenient than ultracentrifugation. However, when this device is applied to human urine samples, we found that some urine samples did not work well for EMV isolation, probably due to the difference in pH, salt concentrations, and so on. In order for our device to work for all urine samples equally, this project was initiated. *Materials and methods*: Using healthy donors’ urine samples, urinary EMV was isolated by a standard differential centrifugation method. Purified EMV was then suspended in various solutions, and applied to our filter device, followed by poly(A)+ RNA purification and RT-PCR to amplify various control, glomeruli-, tubules- and collecting duct-specific mRNAs (Exp. 1). Then, we developed a concentrated “equaliser” solution. This equaliser was added into actual human urine samples, and mRNAs were quantified (Exp. 2). *Results*: We observed that EMV binds to our filter device under wide pH and salt ranges; however, the yield decreased at higher pH and lower salt conditions (Exp. 1). After screening urine samples, we found one sample, which showed no amplification of control genes. Using this urine as a model system, we developed the “equaliser” solution, which improved RT-PCR sensitivities significantly (Exp. 2), suggesting that urine pH and salt concentration might affect the EMV absorption. Moreover, this equaliser showed no or very small effect of on many other urine samples, indicating that the equaliser solution does not hamper the assay results of those samples. *Conclusions*: We developed a unique exosomal mRNA quantification system applicable to biomarker discovery and clinical diagnostics. The system will become the next-generation diagnostics for renal diseases.

**VN96 Peptide: a novel tool for convenient and efficient enrichment of urinary EMVS containing informative prostate cancer protein and mRNA biomarkers**

M. L. Davey ^1^, M. Caissie^1^, S. Melville^1^, S. Fournier^1^, M. Savoie^2^ G. Breault^2^ and R. J. Ouellette^1^

^1^Atlantic Cancer Research Institute, Canada; ^2^Dr. Georges-L.-Dumont University Hospital Centre, Canada

*Introduction*: Early detection of prostate cancer (PCa) currently relies on serum PSA and digital rectal examination (DRE), both of which lack diagnostic specificity and prognostic value. Hence, there is a great demand for new and better PCa biomarkers. Urinary extracellular microvesicles (eMVs) have valuable potential as a novel, non-invasive and enriched source of biomarkers. Conventional means of eMV isolation involve time-consuming ultracentrifugation (UCF) or expensive commercial methods. Our group has developed a fast, simple and cost-effective method for enrichment of eMVs from urine using a peptide (Vn96) with affinity for heat shock proteins. *Materials and methods*: Post-DRE urine samples were collected from patients scheduled for prostate biopsy. eMVs were isolated in parallel using UCF and Vn96 affinity peptide techniques. Western blotting was used to assess and compare the presence of classical eMV markers (e.g. CD9, ALIX, HSP70) and prostate-specific markers (e.g. FOLH1, PSA). Total RNA was extracted from UCF and Vn96 eMVs. RT-qPCR was used to assess the expression of known urinary PCa markers (e.g. PSA, PCA3), a panel of 8 genes of interest to our group and promising new PCa markers previously reported in urine (e.g. SPINK1, PSCA, CD24, survivin). *Results*: Classical eMV and prostate-specific protein markers were readily detected in Western blots of Vn96-captured vesicles. In many instances, these proteins were detected in higher quantities in Vn96 eMV preparations compared to the corresponding UCF eMV preparations. RT-qPCR demonstrated the majority of transcripts of interest could be measured in both UCF and Vn96 enriched eMVs. PCA3/PSA ratio results compared well between UCF and Vn96 eMVs (r=0.89, n=62, p<0.01). Vn96 eMV RNA yielded improved test specificity and accuracy compared to urine sediment RNA. At a fixed sensitivity of 76%, urine sediment PCA3/PSA ratio specificity was 55% with an area under the (ROC) curve of 0.709, while Vn96 eMV PCA3/PSA ratio specificity was 69% with an AUC=0.803. The addition of FOLH1 transcript results to the Vn96 eMV PCA3/PSA ratio resulted in improved test specificity and accuracy (74% specificity and AUC=0.83). *Conclusions*: Vn96-captured urinary eMVs provide an enriched source of prostate-specific protein markers and diagnostically informative mRNA transcripts. A simple eMV enrichment method is critical to the discovery and clinical application of PCa biomarkers in urine.

**Fast and efficient method for isolation of exosomal-like vesicles from cell culture medium and body fluids: urine, saliva, plasma and serum**

Maksym Kremenskoy, Y. Kremenska and I. Kurochkin

A*STAR/Bioinformatics Institute, Singapore

Recently, investigation of exosomal-like vesicles (ELV) becomes in great request. These vesicles have great diagnostic potential for multiple diseases including cancer. Also, ELV could be utilised for multiple purposes such as biological drug or siRNA delivery tool. Isolation of ELV is crucial step for their further study or therapeutic purposes. We propose a method for fast and efficient nanovesicles purification. Particularly, this simple and reliable protocol increases ELV yield and eliminates ultracentrifugation, which is an essential part of a traditional protocol. In our study, we used conditioned medium obtained from several ovarian and breast cancer cell lines. We also utilised human body fluids like urine, saliva, plasma and serum. For ELV precipitation, we have tested different polymer-based solutions. Chromatography in column filled by various types of beads with different pore sizes was used for further ELV purification. ELV presence and yield was determined by Western blot, RNA profiling, nanoparticle tracking analysis and transmission electron microscopy. This novel procedure of exosome purification is faster compared to other existing methods of ELV purification and it produces ELVs essentially free of contaminating proteins and RNA. Using polymer-based method, we concentrated the ELVs from comparably large volumes. Then, we utilised columns that significantly simplified the procedure of ELV purification. Totally, the isolation of ELV by using our method lasts ~2.5h. Our newly developed method of ELV purification yields significantly higher amount of vesicles compared to that obtained using traditional protocol involving ultracentrifugation step. ELVs obtained by using our method are intact and suitable for RNA, DNA and protein analysis, and they also could be used for in vitro and in vivo functional studies. Our method allows processing a wide range of volumes of cell culture medium and body fluids (urine, saliva, etc.).

**Paper-based assays for accessible microvesicle isolation**

B.R. Lin^1^, M.Y. Hsu^2^, C.M. Cheng^1^ and C.C. Chen ^1^

^1^Institute of NanoEngineering and MicroSystems, National Tsing Hua University, Taiwan; ^2^Taichung Veterans General Hospital, Taiwan

*Introduction*: Many cell types release exosomes and microvesicles (EMVs), which are increasingly recognised to be involved in many different cellular processes, and can be obtained non-invasively from body fluids. Thus, the ability to isolate EMVs from the body fluid has far-reaching diagnostic, prognostic, therapeutic and basic biology implications. We have developed a new low-cost, point-of-care, disposable paper-based analytical devices capable of selective separation of EMVs and compatible for subsequent analyses, such as enzyme-linked immunosorbent assays and transcriptomic analysis. *Materials and methods*: Antibodies were immobilised on porous cellulose membranes via the chemical conjugation. Serum samples from healthy donors and aqueous humour samples from patients were used in this study. After rinsing, EMVs adherent to the paper were fixed for scanning electron micrograph (SEM). SEM images were analysed to extract morphological information of captured EMVs. The 2-tailed Student's t-test was used to determine p-values. *Results*: Subsets of EMVs from 72 µL of serum samples from healthy volunteers were isolated using filter papers whose surfaces were immobilised with anti-CD63 antibodies and annexin V, respectively. These subsets of EMVs appeared statistically different in size (p<2.4×10^−22^) and circularity (p<3.6×10^−9^). EMVs with CD63 molecules isolated on the filter paper were generally larger and more rounded (diameter=80.2±31.2, circularity=0.89±0.16, n=108) compared to EMVs with phosphotidylserine on the outer leaflet of the membrane (diameter=42.8±15.7, circularity=0.77±0.17, n=203). Capturing of EMVs from human aqueous humour samples using anti-CD63 antibodies coated filter paper was also demonstrated. *Conclusions*: In conclusion, a paper-based immunoaffinity method was developed to isolate EMVs with an easier user interface, aiming a quicker adoption of this technology in both the research and clinical communities.

**High yield of cancer biomarkers in micro- and nanovesicles obtained in shear stress field**

R. Stukelj ^1^, N. Zarovni^2^, V. Sustar^3^, R. Jansa^4^, K. Gersak^5^, M. Simundicæ^6^, N. Tozon^7^, A. Mrvar-Breèko^8^ and V. Kralj-Iglic^1^

^1^Laboratory of Clinical Biophysics, University of Ljubljana, Faculty of Health Sciences, Slovenia; ^2^Exosomics Siena S.p.A., Siena, Italy; ^3^Laboratory of Clinical Biophysics, Chair of Orthopaedics, Faculty of Medicine, University of Ljublja; ^4^Clinical Department of Gastroenterology, University Medical Centre Ljubljana; ^5^Department of Obstetrics and Gynaecology, University Medical Centre, Ljubljana, Slovenia; ^6^Prva-K Clinic for Small Animals, Ljubljana, Slovenia; ^7^Clinic for Surgery and Small Animals, Faculty of Veterinary Medicine, University of Ljubljana; ^8^Department of Anaesthetics and Surgical Intensive Care, University Medical Centre, Ljubljana, Slovenia

*Introduction*: Micro- and nanovesicles are created from all types of cells in a common basic biophysical process where cell fragments are released into a surrounding solution. In considering blood samples, it was recently indicated that a substantial pool of nanovesicles is created during isolation procedure in which shear stress may play an important role. Micro- and nanovesiculation could be amplified by augmenting surface of contact between the cells-containing fluid and the solid interface undergoing relative motion. To explore a possibility of increasing yield of biomarkers from tissue and body fluid samples for different diseases, especially cancer, we have visualised nanovesicles obtained from different tissues and blood and determined composition of the isolated material. *Methods*: We used parts of tumours excised for therapeutic reasons: human colon tumour, human ovary tumour, dog breast tumour, dog paw tumour, dog myoma and dog hystocytoma. We also used human blood. Tissue samples were shaken in the Millmix20 instrument constructed by Domel d.o.o., Železniki, Slovenia, with 5 mm ceramic beads (30 Hz, 10min), while fluid-derived samples were shaken with 0.1 mm glass beads (15 Hz, 5min). Shaken samples were centrifuged to isolate NVs. The isolates were observed by scanning electron microscope (SEM) and analysed by Western blot (WB) analysis. *Results*: Micro- and nanovesicles were observed in micrographs of isolates from blood and the fragmented tissue superanatant. The size of the fragments depended on the isolation procedure. Immunofluorescent signals of considerable intensities were recorded in WB of samples obtained by fragmentation in shear stress field from tumour tissue and from blood. *Conclusions*: Fragmentation of cells in shear stress field is a useful method for harvesting micro- and nanovesicles.

**Improved immunoassays for detection and quantitative analysis of selected protein biomarkers in human plasma exosomes**

N. Zarovni ^1^, G. Radano^1^, E. Lari^1^, A Corrado^1^, P. Guazzi^2^, J. Muhhina^2^ and A. Chiesi^1^

^1^Exosomics Siena SpA, Italy; ^2^Hansabiomed OU, Estonia

*Introduction*: Detection of exosomal proteins in biological samples often poses the problem of unspecific immunoreactivity that interferes with assays outcome and results in false positives or blockade of a specific signal. This can be linked to the purification method that determines exosome yield and purity. We optimised FACS and ELISA for detection of common and specific exosomal proteins, evaluating the purification by ultracentrifugation vs. immunocapture, testing diverse blocking solutions and correlating the assay readouts with quantification of exosomes using NTA and IR-spectrometry. *Materials and methods*: Exosomes were purified ultracentrifugation from human plasma samples. FACS analysis of beads-conjugated exosomes is performed upon incubation with alternative blocking agents and/or fixatives. ELISA was performed with ExoTESTTM assay using purified exosomes or precleared plasma samples. Anti-CD9 was used in combination with appropriate secondary and alternative primary antibodies for detection of specific tumour markers in clinical samples. *Results*: FACS and ELISA analysis of plasma exosomes revealed unspecific binding of secondary Ab that hampers detection and comparison of specific target proteins in different samples. This issue is complicated by inter-individual variability in immunoreactivity and exosome amount in healthy individuals. This observation was affected by exosome purification method, while unaltered by sample storage. We show that adequate blocking can decrease or abolish this artefact enabling accurate comparison of common or tissue-specific exosomal proteins. Quantitative ELISA was validated against NTA and IR-spectrometry. *Conclusions*: We propose optimised protocols providing increased sensibility and sensitivity in reproducible detection of proteins of interest on exosomes in relevant clinical samples, enabling development of ExoTESTTM assays with improved performance and resolution for research and diagnostic applications.

**Metrological characterisation of microvesicles from body fluids as non-invasive diagnostic biomarkers**

Rienk Nieuwland

Academic Medical Centre of the University of Amsterdam, The Netherlands

*Introduction*: To establish extracellular vesicles (EVs) as novel biomarkers, measurement results need to be quantitative and traceable. The detection of EV is a challenge, however, due to their small size (average diameter less than 100 nm) and heterogeneity, and the complexity of body fluids. State-of-the-art EV detection techniques measure only a minor fraction of all EV present, and thus the obtained information is incomplete, results are qualitative rather than quantitative, and cannot be compared between laboratories. *Materials and methods*: METVES (www.metves.eu) is a European Metrology Research Programme project (EMRP is jointly funded by the EMRP participating countries within EURAMET and the European Union; for additional information, please see http://www.euramet.org/fileadmin/docs/EMRP/JRP/JRP_Summaries_2011/Health_JRPs/HLT02_Publishable_JRP_Summary.pdf). This project is a collaboration between a university hospital (AMC) and 4 European metrology institutes (EJPD, PTB, SMD, VSL), started in June 2012, and aims to (a) explore, compare and develop methodologies for dimensional characterisation and to measure concentration, morphology and (bio) chemical composition using techniques such as small-angle X-ray scattering (SAXS), Anomalous SAXS and Atomic Force Microscopy, (b) develop methods for standardised collection and handling of human body fluids and (c) select and test synthetic and biological reference materials to allow traceable calibrations of EV measurements. *Results*: Initial results will be presented and discussed. *Conclusions*: METVES is a multidisciplinary approach to improve the traceable characterisation of MV in body fluids, thereby paving the road towards the use of EV as biomarkers of disease.

**Antigenic capturing, detection and phenotyping of exosomes using protein microarray**

Jørgensen Malene ^1^, R. Bæk^1^, E.K.L. Søndergaard^1^, S. Pedersen^2^, S.R. Kristensen^2^ and K. Varming^1^

^1^Department of Clinical Immunology, Aalborg University Hospital, Aalborg, Denmark; ^2^Department of Clinical Biochemistry, Aalborg University Hospital, Aalborg, Denmark

*Introduction*: The quantity and molecular composition of exosomes shed from different cell types differs considerably. Until now the “gold standard” for quantification, characterisation and phenotyping of exosomes is either by Western blotting or flow cytometry (FACS) using bead capturing. These types of analyses use considerable amounts of exosomal material (20–30 µg of protein derived from~10^8^ cells) and mostly gain results from only one analyte at the time. The aim of this study was to test the novel multiplexed platform of protein microarray used for capturing, detecting and profiling exosomes in plasma from healthy donors and cancer cell cultures. *Materials and methods*: The assay is based on the antibody capture of microvesicles and subsequent detection of the captured microvesicles by biotin-labelled anti-tetraspanin antibodies (CD9, CD63 and CD81). Twenty-one different antibodies were used to capture and phenotype the exosomes for both the general exosomer markers, membrane markers (e.g. PLAP, HLA-ABC) and tumour-associated antigens (e.g. EpCam, HER2, CD276 [B7H3]). The method was validated using the nanoparticle tracking analysis (NTA) before and after capturing of exosomes by protein microarray. *Results and conclusion*: Protein microarray utilises: the possibilities to detect and profile exosomes for 21 analytes simultaneously using only 2–10 µl of plasma or cell culture medium from ~10^4^ cells. After detection, the captured microvesicles were eluated to validate the size distribution by NTA. The validation showed that the protein microarray mainly captured microparticles below 100 nm in size. Thus, protein microarray is a well-functioning new method for exosome characterisation and phenotyping using only small amounts of starting material. The method was further used to characterise plasma exosomes from 80 healthy donors.

**Programmed interface between exosomes and material surfaces**

Kiyotaka Shiba, K. Suga, K. Hibino and M. Yoshida

Japanese Foundation for Cancer Research, Tokyo, Japan

*Introduction*: An understanding of the interaction between exosomes and the surfaces of various materials and its rational control underlie the development of diagnostic and remedial devices in the emerging area of exosome medicine. Here, we present quantitative analyses of the interactions between exosomes and the surfaces of inorganic materials; we also introduce the effects of the immobilisation of a peptide aptamer that has an affinity to a molecule expressed on exosomes, on the surfaces. *Materials and methods*: The interactions between exosomes and material surfaces were evaluated by QCM-D. An anti-EpCAM peptide aptamer, Ep114, was immobilised on material surfaces by using the conjugant between Ep114 and a MPC polymer. *Results*: Interactions of exosomes on the surfaces of Au, SiO_2_ or Al_2_O_3_ during a 500-min incubation time were insensible when the concentration exosome was 10^9^ particles/ml. However, exosomes whose concentration was 10^10^ particles/ml dramatically decreased the frequency of the sensors, and at the same time, increased the energy dissipation; this suggests that exosomes accumulated on the surfaces of the sensors. When the sensors were spin-coated with an 2-methacryloyloxyethyl phosphorylcholine (MPC) polymer, exosome interaction was diminished. When the QCM sensor was coated with EpiVeta, a conjugate between MPC and an EpCAM binding peptide, we experienced instability in QCM measurements, suggesting that refinements in the conditions of EpiVeta coatings are needed. *Conclusions*: The data of QCM-D measurements suggest that a high concentration of exosomes could form a soft and thick layer on the surfaces of Au, SiO_2_ or Al_2_O_3_. However, this interaction could be avoided by coating the surfaces with MPC, a polymer. Possible endowments rendered through specific interactions by using a peptide aptamer will be discussed.

**Quantification of nanosized extracellular vesicles with resistive pulse sensing**

J. de Vrij ^1^, S.L.N. Maas^1^, M. Sena-Esteves^2^, M.L. Lamfers^3^ and M.L.D. Broekman^1^

^1^Department of Neurosurgery, University Medical Center, Utrecht, The Netherlands; ^2^Department of Neurology and Gene Therapy Center, University of Massachusetts Medical School, Worcester, USA; ^3^Department of Neurosurgery, Erasmus Medical Center, Rotterdam, The Netherlands

*Introduction*: Cells secrete different types of extracellular vesicles (EVs), which may act as important entities in normal human physiology and in various pathological processes. The established methods for quantification of EVs require purification or pre-analytical handling of samples with labelling moieties. Our aim was to develop a method for high-throughput, labelling-free quantification of non-purified EVs. *Materials and methods*: Resistive Pulse Sensing (RPS) technology, which relies on the detection of particles upon their movement through a nanopore, was investigated for the ability to quantify nanosized EVs (<400 nm) in bodily fluids and cell culture supernatant. *Results*: RPS allowed for the quantification of EVs in blood, urine, pleural fluid and cell culture supernatants. Measurements were relatively easy and fast, and required only 30 µl per sample. In addition, the RPS-based technology allowed for size-distribution analysis of EVs. *Conclusion*: RPS technology offers a highly valuable addition to the currently used repertoire of methods for quantification and size-profiling of EVs. Side-by-side comparison with alternative methods is ongoing, and it is investigated whether modifications of the technique allow for phenotyping of subtypes of EVs.

**Counting of exosomes: validation of the accuracy of concentration measurements by particle tracking devices**

Clemens Helmbrecht and H. Wachernig

Particle Metrix GmbH, Meerbusch, Germany

Automated laser light scattering particle tracking techniques (APT) have found broad acceptance in the field of exosome analysis as they combine determination of particle size distribution, zeta potential measurement and particle counting in a single experiment. The focus of this communication is the validation of accuracy of the particle concentration measurement. To set the start conditions for APT, an automated sequence verifies the system quality, aligns the integrated optical microscope and optimises the camera settings. To avoid multiple identification of same particles, up to 11 positions within the measurement cell are scanned. The computation of particle concentration requires the particle number and the knowledge of volume containing the particles. The accuracy of the determination of particle concentration was verified with size standards ranging from 30 nm to 3 µm for various materials. For reference analysis, the particle concentration of the size standards was determined by ultrafiltration: an aliquot of the suspension was filtered through a polycarbonate pore filter and the difference of the filter mass before and after filtration was determined. We could show that the accuracy of the determination of particle concentration by APT lies within the experimental error of the reference analysis method performed by ultrafiltration.

**Novel approach for characterising circulating microparticles using imaging flow cytometry**

U.E. Erdbruegger ^1^, C.R. Rudy^1^, M.Y. Yeager^1^, K.D. Dryden^1^, S.H. Hart^1^ and J.L. Lannigan^1^

^1^University of Virginia Health System, Charlottesville, USA; ^2^Lumacyte, LLC, USA

*Introduction*: Microparticles (MPs) are submicron vesicles released from cell membranes in response to activation, cell injury or apoptosis. The clinical importance of evaluating MP has become increasingly recognised, although no standardised method exists for their measurement. Flow cytometry (FC) is the most commonly used technique, however, because of the small size of MP and the limitations of current FC instrumentation, accurate measurements are hampered by this methodology. We decided to investigate whether the use of FC combined with imaging, such as it is possible with the Imagestream imaging flow cytometer (ISX), would be a more robust approach to characterising MPs. *Methods*: MP pellets were obtained from platelet poor plasma from healthy donors and stained with flouresceinated annexin V and a series of antibodies associated with some of the more common sources of MP (anti-CD41, anti-CD235a, anti-CD45, anti-CD31 and anti-CD42b). Split samples were run on both a standard FACSCalibur FC and the ISX. *Results*: A robust gating strategy could be developed to detect MP with ISX using the equivalent of side scatter (SSC). MPs could clearly be separated from polystyrene beads, cell debris and cells. In addition, different phenotypes and a dynamic size range of MP of different origin (e.g. red blood cells, platelets, leukocytes) could be identified. Direct comparison of data analysis with conventional FC and ISX demonstrated that FC overestimates frequency of MP expressing multiple markers, mostly likely due to aggregate formation or coincident events. The ISX has sensitivity down to 200 nm, whereas FC sensitivity is 500 nm or greater. *Conclusions*: By combining flow cytometry with imaging, we were able to demonstrate improved detection, phenotyping and absolute counting of MPs, while also providing morphological confirmation and the ability to distinguish true single events from cell debris or particle aggregation. Evaluating MPs below 200 nm and sizing remain a challenge.

**Poster Session III (Arlington-Berkeley): Stem Cells/Vascular April 18**

**Chair: *D. Lyden and P. Quesenberry***

**Mesenchymal stem cell-derived exosomes regulate vascular cell phenotype in experimental models of pulmonary hypertension**

K. Sdrimas, C. Lee, S. Kourembanas and S.A. Mitsialis

Division of Newborn Medicine, Children's Hospital, Boston, MA, USA

*Introduction*: Pulmonary vascular remodelling, a hallmark of pulmonary arterial hypertension (PAH), is characterised by aberrant changes on all layers of the vessel wall. Vascular smooth muscle cells (VSMCs) contribute to remodelling by acquiring a proliferative, secretory and less contractile phenotype while endothelial cells (ECs) become apoptosis-resistant and angioproliferative. Adventitial fibroblasts also switch to a more migratory, myofibroblastic phenotype. We recently reported that exosomes derived from mesenchymal stem cells (MEX) have a protective effect in a mouse model of hypoxia-induced PAH. We hypothesise that MEX treatment prevents the phenotypic changes triggered by the signals that initiate vascular remodelling. *Materials and methods*: Human primary lung fibroblast cultures were established from a healthy donor and pulmonary artery ECs and VSMCs were obtained from Lonza Biologic. MEX were isolated and characterised as previously reported. Cultured cells were exposed to hypoxia (1–5% oxygen) and treated with MEX or fibroblast exosomes while stimulated by growth factors and conditions known to modulate fibroblast and VSMC phenotype. Hypoxic signalling pathways and phenotype-specific markers were examined. *Results*: MEX treatment inhibited the hypoxic activation of STAT3 in endothelial cells and abrogated the increases in α-smooth muscle actin (α-SMA) mRNA and protein, a typical myofibroblast marker, in TGF-β or hypoxia-exposed lung fibroblasts. MEX also reduced the migratory capacity of lung fibroblasts in modified Boyden chamber assays and ameliorated the PDGF-induced phenotypic changes of VSMCs. *Conclusions*: The protective effects of MEX treatment on PAH are partially mediated by the inhibition of phenotypic changes in vascular cells of medial and adventitial origin, preserving their normal, non-proliferative phenotype.

**Prostate cancer exosomes can alter the fate of mesenchymal stem cell differentiation**

R. Chowdhury, J. Webber, M.D. Mason, Z. Tabi and A. Clayton

Institute of Cancer and Genetics, Cardiff University, Cardiff, United Kingdom

*Introduction*: The reactive stroma in prostate (Pca) and other cancers are predominantly composed of tumour promoting myofibroblasts. Bone marrow mesenchymal stem cells (BM-MSC) are known to migrate into tumours possibly acting as precursors of stromal myofibroblasts. We hypothesise that cancer exosomes can modulate MSC fate to become disease-promoting myofibroblasts. *Materials and methods*: MSC's were defined by phenotype (flow cytometry) and capacity to undergo adipogenic differentiation. Exosomes purified using a sucrose cushion were characterised by Western blot, nanosight and flow cytometry of exosome-coated beads. The phenotypic changes of MSC's in response to exosomes was analysed by immunohistochemistry and ELISA. Exosome-deficient cancer cell conditioned media, generated by Rab27a knockdown, or by ultracentrifugation removal, were used as stimulus for MSC differentiation. *Results*: BM-MSC's expressed all expected phenotypic markers, and underwent adipogenic differentiation staining positive for Oil Red O. Purified PCa exosomes strongly inhibited adipogenic differentiation of MSC's, differentiating instead into α-smooth muscle actin positive myofibroblast-like cells, with elevated VEGF-A secretion. Rab27a knockdown decreased secretion of exosomes into culture media, which in turn failed to drive myofibroblastic differentiation of MSC. Similar results were generated by ultracentrifugation depletion of exosomes. *Conclusion*: We conclude that prostate cancer exosomes dominantly modulate MSC differentiation towards a VEGF-A high, myofibroblastic cell phenotype. Future studies will examine the in vivo importance of this for tumour progression.

**Exosomes and cardiac repair**

J.A. Maring ^1^, K.R. Vrijsen^2^, J. de Vrij^3^, J.P.G. Sluijter^2^ and M.J. Goumans^1^

^1^Leiden University Medical Centre, Leiden, The Netherlands; ^2^University Medical Centre, Utrecht, The Netherlands; ^3^Erasmus Medical Centre, Rotterdam, The Netherlands

*Introduction*: Despite advantages in treatment, cardiovascular disease remains a leading cause of death in the western world. After myocardial infarction, contractile tissue is lost and replaced by scar tissue. This can ultimately lead to heart failure due to the great stress put on the remaining tissue. Current therapies only treat symptoms, but do not solve the underlying cause, which is the loss of cardiomyocytes. Cardiomyocytes do not proliferate themselves and are therefore not capable of replacing lost tissue. However, cardiomyocyte progenitors cells (CMPCs) have been found in the heart, which are capable of differentiating to cardiomyocytes, endothelial cells and smooth muscle cells. These CMPCs could have great potential in achieving a therapy in which the lost cardiac tissue is replaced. *Results*: In vivo experiments have shown that only a small percentage of cells is recovered after injecting CMPCs after myocardial infarction. Yet, in these experiments, a positive effect of these cells could already be seen 2 days after myocardial infarction, suggesting a role for paracrine factors. The factors responsible for this were identified to be over 1000 kDa in size and are therefore likely to be vesicles like exosomes rather than secreted proteins. *Conclusions*: We want to investigate what the effect of exosomes from different sources is on the growth and behaviour of CMPCs, endothelial cells and fibroblasts, both in vitro and in vivo.

**Elevated endothelial-derived microparticles in patients with familial hypercholesterolemia: potential marker of endothelial dysfunction**

Morten Hjuler Nielsen ^1^, S. Vedel^2^, H. Irvine^3^, B. Raungaard^4^, H. Beck-Nielsen^5^ and A. Handberg^6^

^1^Department of Clinical Biochemistry, Aarhus University Hospital, Denmark; ^2^Department of Radiology, Aarhus University Hospital, Denmark; ^3^Department of Medicine and Cardiology A, Aarhus University Hospital, Denmark; ^4^Department of Cardiology, Aalborg Hospital, Aarhus University Hospital, Denmark; ^5^Department of Endocrinology M, University of Southern Denmark, Odense, Denmark; ^6^Department of Clinical Biochemistry, Aalborg Hospital, Aarhus University Hospital, Denmark

*Introduction*: Endothelial microparticle (EMP) counts are elevated in a variety of pathological conditions, including cardiovascular disease. The association between lipoprotein levels and the incidence of atherosclerosis-related diseases is well known. Moreover, the triglyceride/HDL-C ratio, commonly used as an indirect measure of the more atherogenic small, dense LDL particles, has been shown to predict extensive coronary disease. This study aimed to investigate the association between various lipoprotein particles on EMP release in patients with familial hypercholesterolemia (FH). *Materials and methods*: Thirty subjects with FH and 24 age, and gender-matched normolipidemic controls were included. Before study entry, FH subjects had an 8-week washout period of lipid-lowering medication. Carotid intima-media thickness (IMT), a marker of subclinical atherosclerosis, was measured by ultrasonography. Circulating EMPs (CD31+/CD42b−) were detected using a BD FACSAria III high-speed cell sorter. The microparticle gate was defined by an upper limit of 1.0 µm and a lower limit set above the background noise level of the instrument. *Results*: Total EMP counts were significantly increased in FH subjects, compared to healthy controls (p<0.003). Univariate analysis showed significant associations between IMT and total cholesterol (Total-C) (r=0.34, p=0.14), LDL cholesterol (LDL-C) (r=0.33, p=0.015) and oxidized LDL cholesterol (oxLDL-C) (r=0.34, p=0.011). Although, we did not find a direct association between IMT and EMP counts, we observed associations between EMPs and Total-C (r=0.33, p=0.005), LDL-C (r=0.33, p=0.006), oxLDL-C (r=0.35, p=0.001), and low levels of HDL-C (r=−0.34, p=0.023), as well as the TG/HDL ratio (r=0.50, p<0.001). *Conclusions*: EMPs are significantly elevated in patients with FH, and strongly associated to atherogenic cholesterol fractions. EMPs may provide new options for risk assessment.

**Gene expression profiling in circulating microparticles of patients with acute coronary syndrome**

Luigi M. Biasucci ^1^, M.M. Marcantoni^2^, A.S. Severino^2^, M.T.C. Cardillo^2^, R.V. Vergallo^2^, L.D. V Di Vito^2^, A.C. Caroli^2^ and G.D.M. de maria^2^, G.C. Copponi, G.C.^2^ and F.C. Crea^2^

^1^Cardiology, Italy; ^2^Catholic University, Italy

*Introduction*: Microparticles (MPs) are small vesicles released from activated cells. MPs are increased in acute coronary syndromes (ACS) and have been associated with procoagulant activity, yet little is known about their mRNA expression. We sought to characterise mRNA expression in MPs of ACS and stable angina (SA) pts. *Methods*: Blood samples were drawn from 32 pts: 16 with Non-ST elevation myocardial infarction (NSTEMI) and 16 with SA. Gene expression of NSTEMI and SA was compared with 17 healthy subjects. Total RNA was isolated from circulating MP. mRNA expression was examined with a PCR-array system for the atherosclerosis (AT) and transcription factors (TF) pathways. *Results*: One hundred and sixty-eight genes were investigated. Compared to controls, mRNA expression identified 5 modulated genes for the AT pathway between NSTEMI and SA (3 up- and 2 down-regulated in NSTEMI vs. SA). Elastin, matrix metalloproteinase-1 (MMP-1) and selectin showed differences in mRNA with a fold change (FC)>5 in NSTEMI vs. SA (p<0.05). On the contrary, angiotensin I-converting enzyme (ACE) and neuropeptide Y (NPY) mRNA were down-regulated in NSTEMI (p<0.05). The TF pathway revealed 8 modulated genes (1 up- and 7 down-regulated in NSTEMI pts). Androgen receptor (AR), forkhead box 01 (FOX01) were down-regulated with a FC>10 (p<0.05); MYC associated factor X (MAX), nuclear factor of kappa light polypeptide gene (NFKB1), v-rel reticuloendotheliosis viral oncogene (RELA) and signal transducer and activator of transcription 4 (STAT4) were down-regulated with a FC>5 in NSTEMI vs. SA (p<0.05). General transcription factor IIB was up-regulated in NSTEMI with a FC>3 (p<0.05). *Conclusion*:Our study revealed a different MP mRNA profile in NSTEMI pts compared to SA. The higher differences in mRNA expression were found among proteins involved in extracellular matrix synthesis or degradation, leukocyte infiltration and inflammation suggesting a role of MP also on plaque instability and as a potential novel therapeutic target.

**Detection of genetic material (DNA, microDNA, RNA and microRNA) in microvesicles of human saliva of insulin dependent and non-insulin dependent diabetic patients using mass spectroscopic analysis in support of W. G. Chen's 1968 hypothesis of genetic exchange**

Wilfred G. Chen

Urology Research Unit, Trinidad and Tobago

*Introduction*: Contrary to the central biological dogma that DNA is fixed and stable, the author proposed the Hypothesis of Genetic Exchange in 1968, which postulates that “there exists a system of exchange of intercellular and intertissue factors including genetic material DNA/RNA between cells and tissue, stem cells and adult cells. This is important for the homeostatic control of growth and differentiation. As a corollary to this hypothesis, disturbances of the exchanging system or the message themselves, result in abnormal growth even cancer.” Recently, there has been increasing evidence to support such a concept. More recently, Muller 2012 in a review of microvesicles exosomes as novel biomarkers of metabolic diseases, and the (patho) physiological roles of exosomes and microvesicles (EMV) associated with mi/ RNAs, states that “the engagement of EMVs for transfer of genetic material could be an efficient method within the human body to exchange biological information”. *Materials and methods*: On the basis of this hypothesis, mass spectroscopic analysis of salivary microvesicles is being investigated, as saliva is easily accessible, and may be useful in the detection and monitoring of diseases, local and systemic. Accurate analysis of macromolecules, in and out of cells, can be achieved using mass spectroscopy, in the standard state, without disturbances by ultra centrifugation, anticoagulants, lipoprotein particles and small platelets. A previous report has been presented on non-insulin-dependent diabetes. This report expands the studies to insulin dependent diabetes. *Results*: The results revealed microvesicles enclosing DNA molecules of varying length, some 25 nucleotides long, RNA molecules of varying length, some 25 nucleotides long and proteins in both healthy, non-insulin- and insulin-dependent diabetic patients. Further studies are continuing. *Conclusion*: This is the first demonstration of DNA molecules in salivary microvesicles of insulin-dependent diabetics and confirms their presence in non-insulin-dependent diabetics and healthy patients as previously reported. Microvesicles contain DNA, RNA and proteins making them possible carriers of intercellular information, supporting the Hypothesis of Genetic Exchange.

**Exosome-mediated tumor stromal support of mesenchymal stem cells**

P. Penfornis ^1^, F. Guillonneau^2^, J.D. Fernandes^1^, S.S. Dhule^3^ and R.R. Pochampally^1^

^1^Cancer Institute and Department of Biochemistry - University of Mississippi Medical Center, Jackson, United States; ^2^3P5 Proteomic Platform of the Université Paris Descartes, Sorbonne Paris Cité, Paris, France; ^3^Tulane University Health Sciences Center, New Orleans, LA, United States

*Introduction*: Our laboratory has shown that serum-deprived mesenchymal stem cells (SD-MSCs) have the surprising ability to survive for long periods (more than 60 days) by using autophagy. In addition, these SD-MSCs aid in tumour survival/growth both by paracrine effects of their secretome. In this study we characterize the exosomal fraction SD-MSCs secretome, and their role in tumour supportive properties. *Materials and Methods*: Exosomal fraction was obtained from serum deprived MSCs by concentration followed by ultracentrifugation method. Small RNA contained in exosomes were extracted and screened by next-generation sequencing. Proteins from exosomes were extracted and the trypsin digest of the Rapigest(tm)-resuspended pellet was analyzed using both nanoLC MALDI-TOF/TOF and nanoRSLC-ESI-LTQ-OrbiTrap MS/MS analyses. *Results*: Next-generation sequencing assays used to identify miRNAs secreted in the exosomes indicated presence of tumour supportive miRNA. Next, we pursued characterization of a new and abundant exosomal miRNA, which displayed an essential role on cancer cell proliferation. Proteomics revealed the presence of 300 different proteins most of which are growth factors (IGF-2, PAI-1), growth-factors binding proteins (IGFBPs), and metalloproteinase inhibitors (TIMP1, TIMP2). Both identified miRNA and proteins map the pathways involved in cell growth and proliferation, cell death, cell-to-cell signaling and metabolism. *Conclusion*: Our study revealed the complexity of exosomes secreted by MSCs. The miRNAs and proteins detected could act as indirect post-transcriptional regulators (miRNA) or as direct signaling pathway regulators (growth factors/integrins). This cell to cell communication through exosomes could reveal a novel mechanism for paracrine regulation of stromal cells in solid tumours.

**Galectin 3 is a component of CD63 positive circulating microvesicles and boosts osteogenic differentiation of mesenchymal stem cells**

Sylvia Weilner, E. Schraml^1^, H. Redl^2^, R. Grillari-Voglauer^1^ and J. Grillari^1^

^1^University of Natural Resources and Life Sciences, Austria; ^2^Ludwig Boltzmann Institute for Experimental and Clinical Traumatology, Austria

*Introduction*: Ageing is a complex process that results in the decline of physiologic functions due to accumulation of damage in cells and tissues as well as in reduced repair capacities. The regenerative power of stem and progenitor cells has been found to decline with age and to be influenced by the systemic environment. Supporting evidences are given by the observation that factors within the blood of aged mice are able to inhibit the differentiation capacity of adult stem cells. Here, we set out to identify circulating factors of the aged systemic environment that influence the functionality of adult stem cells. *Results*: While searching for systemic factors that influence osteogenic differentiation capacity of mesenchymal stem cells (MSCs), Galectin 3 was found. It was shown that Galectin 3 is not only secreted by CD63 negative extracellular microvesicles (EVs), but to a similar extent also by CD63 positive EVs derived from senescent endothelial cells. The fact that endothelial cells line the blood vessels supports the notion that secretion of Galectin 3 within vesicles may also occur in vivo. This idea was proven by investigating human plasma samples. Analysing the protein content of human plasma-derived EVs revealed that Galectin 3 is a component of circulating CD63 positive EVs. Furthermore, a possible genetic transfer of Galectin 3 via EVs was confirmed as coincubation of EVs and MSCs resulted in an uptake of EVs via endocytosis. Finally, overexpression of Galectin 3 was shown to boost osteogenic differentiation capacity of MSCs while reducing its expression with small interfering RNA inhibited osteogenesis. *Conclusion*: Summarising, our data suggest that Galectin 3 is able to enhance osteogenesis and that it is enriched within CD63 positive EVs isolated from human serum. Furthermore, endothelial cells were identified as a potential source for circulating EVs containing Galectin 3.

**Mesenchymal stem cell-derived exosomes interact with monocytes and mesenchymal stem cells**

Xiaoqin Wang, O. Omar, F. Vazirisani, Z. Mladenovic, P. Thomsen and K. Ekström

Department of Biomaterials, University of Gothenburg, BIOMATCELL VINN Excellence Center of Biomaterials a, Gothenburg, Sweden

*Introduction*: Both mesenchymal stem cells (MSCs) and monocytes play crucial roles in bone healing process. Monocytes and MSCs migrate to bone–implant interface and appear to co-operate during the transition from inflammation to regeneration. Hitherto, it has been suggested that their mode of interaction involves signalling via soluble factors. Nevertheless, how they communicate is still not clearly understood. Exosomes provide a new mode for communication between cells in the microenvironment or over a distance. The aim of this study was to investigate whether MSC-derived exosomes can mediate MSC–MSC and MSC–monocyte communication. *Materials and methods*: Human adipose-derived MSCs were expanded in media with exosome-free serum. Monocytes were isolated from human buffy coat (purity about 90%). Exosomes were isolated from collected MSC cultures, captured by anti-CD63 coated latex beads and characterised by flow cytometry (CD9, CD63 and CD81). MSC-derived exosomes were labelled with PKH67 and added to cultures with MSCs or monocytes. After 48h, the cells were harvested and the uptake was detected by flow cytometry and fluorescence microscopy. *Results*: MSC-derived exosomes were positive for CD9, CD63 and CD81. The detection by flow cytometry of PKH67-labelled MSC-derived exosomes in MSCs or monocytes showed that ~13% of MSCs and ~8% of monocytes were positive for the PKH67. Fluorescence microscopy revealed exosomes mainly localised over the cytoplasm of recipient cells. As judged by the intensity, the amount of exosomes differed among recipient cells. Taken together, the results suggest that MSC-derived exosomes were internalised in subpopulations of both MSCs and monocytes. *Conclusions*: The results demonstrate that human adipose-derived MSCs release exosomes that are internalised in human monocytes and other human adipose-derived MSCs. The present observations indicate that MSC-derived exosomes are vehicles for MSC–MSC and MSC–monocyte communication.

**Mesenchymal stem cell-derived exosome activates TLR-dependent and TLR-independent pathways to induce a biphasic monocytic expression of IL-10**

Bin Zhang, Y.J. Yin, R.C. Lai and S.K. Lim

Institute of Medical Biology, Singapore

*Introduction*: Interleukin (IL)-10 is an important anti-inflammatory cytokine that is critical in immune regulation. The clinical efficacy of mesenchymal stem cell (MSC) against immune diseases is thought to be mediated by secreted molecules. Although several molecules have been proposed as the secreted MSC immune-modulatory agent, none has been definitively validated. Our group has previously demonstrated that MSC exosomes protect against myocardial reperfusion injury in mice. In this study, we observed that these exosomes induced a biphasic IL-10 expression in THP-1 monocyte through Toll-like receptor (TLR) activation. *Materials and methods*: MSC exosomes were assessed for TLR activity using 2 commercially available THP1 reporter cell lines, THP1-XBlue and MyD88-deficient THP1-XBlue (THP1-XBlue-defMYD) cells, which carried a stably transfected NFκB-inducible secreted embryonic alkaline phosphatase (SEAP) reporter gene. TLR stimulation will induce THP1-XBlue but not THP1-XBlue-defMYD cells to secrete SEAP into the culture medium and express cytokine gene, for example, IL10. SEAP secretion was monitored by a colorimetric assay and cytokine gene expression by qRT-PCR. To assess exosome uptake, THP-1 cells were incubated with fluorescence-labelled exosomes and fluorescence-labelled exosomes coated on 100-µm polystyrene beads. The cells were monitored for fluorescence exosome uptake by FACS and IL-10 gene expression by RT-PCR. *Results*: Exposure to MSC exosomes induced SEAP secretion in THP1-XBlue but THP1-XBlue-defMYD cells, a biphasic IL-10 expression at 3h and 72h in THP1-XBlue cells, and a monophasic IL-10 expression at 72h in THP1-XBlue-defMYD cells. The 72h induction was abrogated by inhibiting exosome uptake through immobilisation of exosomes on beads. *Conclusions*: MSC exosomes induce a biphasic IL-10 expression in monocytes via 2 pathways. The first induction at 3h used a MyD88-dependent TRL signalling pathway that did not require internalisation.

**MicroRNA-146A impairs the endothelium-cardiomyocyte cross-talk in peripartum cardiomyopathy**

Sebastien Tabruyn ^1^, J. Halkein^1^, A. Haghikia^2^, M. Hoch^2^, N. Q. N. Nguyen^1^, M. Scherr^3^, K. Castermans^1^, L. Malvaux^1^, V. Lambert^4^, K. Sliwa^5^, A. Noel^4^, J. Martial^1^, D. Hilfiker-Kleiner^2^ and I. Struman^1^

^1^Unit of Molecular Biology and Genetic Engineering, GIGA-Cancer, University of Liège, Belgium; ^2^Department of Cardiology and Angiology, Medical School Hannover, Germany; ^3^Department of Hematology, Hemostasis, Oncology and Stem Cell Transplantation, Medical School Hannover, Germany; ^4^Laboratory of Biology of Tumor and Development, GIGA-Cancer, University of Liège, Belgium; Department of Medicine, Hatter Institute for Cardiovascular Research, University of Cape Town, South Africa

*Introduction*: Peripartum cardiomyopathy (PPCM) is a life-threatening pregnancy-associated heart disease in previously healthy women driven in part by the 16 kDa N-terminal prolactin fragment (16K PRL). *Methods and results*: We found that 16K PRL induces miR-146a expression in endothelial cells, which by down-regulating NRAS mediates its angiostatic effects. 16K PRL stimulated the release of miR-146a loaded exosomes from endothelial cells, which in a paracrine way increased miR-146a levels in cardiomyocytes and subsequently decreased ErbB4 expression, a feature associated with reduced metabolic activity. Mice with PPCM, due to a cardiomyocyte-restricted knockout of STAT3 (CKO), displayed increased cardiac expression of miR-146a associated with down-regulated NRAS and ErbB4. Blocking miR146a attenuated PPCM in CKO mice without interrupting full-length prolactin signalling indicated by normal nursing activities. MiR-146a is elevated in plasma and in hearts of PPCM patients, but not in patients with dilated cardiomyopathy. *Conclusion*: MiR-146a emerges as a novel specific biomarker and potential therapeutic target for PPCM.

**Comparison of circulating microparticles counts in patients with acute coronary syndrome with 2 methodologies**

Silvia Montoro-García ^1^, E. Shantsila^1^, A. I. Romero^2^, A. López-Cuenca^2^, F. Marín^2^, D. Hernández-Romero^2^, E. Orenes-Piñero^2^, M. Valdés^2^ and G. Y. H. Lip^1^

^1^Centre for Cardiovascular Sciences, University of Birmingham, Birmingham, United Kingdom; ^2^Department of Cardiology, Hospital Universitario Virgen de la Arrixaca, Murcia, Spain

*Introduction*: Circulating microparticles (MPs) have become promising biomarkers in many pathological situations. Measurement of MPs is not standardised and most studies have focused on relatively large MPs due to technological limitations. Two flow cytometric methodologies were compared for the enumeration and characterisation of different subtypes of MPs in acute coronary syndrome (ACS) patients and its changes during 30 days. *Methods*: We recruited 113 ACS patients (aged 68±12 years, 65% males); citrated platelet poor plasma was collected within 24h of percutaneous coronary intervention (PCI), at day 7 and day 30 post-PCI. Aliquots of the same samples were processed in 2 different flow cytometers (FCM), avoiding pre-analytical issues. Polystyrene (pp) beads (0.1-0.5-1 μm) and silica beads (0.1-0.3-0.8 μm) were used to sizing the MP gate in both FCMs. CD42b+ platelet MPs (PMPs), CD144+ endothelial MPs (EMPs) and CD14+ monocyte MPs (MMPs) were quantified in a high resolution Apogee A50 FCM and in a conventional FCM (FACSCalibur) with a 0.5 μm (pp beads) or 0.8 μm (silica beads) detection limit. CytoCounts beads were used for absolute counts in FACSCalibur. *Results*: MPs counts were significantly higher when the samples were processed with the A50, mainly PMPs (p=0.005). However, no changes were detected in MPs counts following PCI (PMPs p=0.51, EMPs p=0.40, MMPs p=0.24). When small-size MPs (sMPs) (<0.5 μm) were quantified in a high sensitive FCM, a significant increase in sPMPs and sEMPs (p=0.012 and p=0.015, respectively) was found during the follow-up period, but sMMPs remained constant (p=0.81). *Conclusions*: Latest generation of FCMs likely display higher sensitivity for MPs mainly due to lower detection limits and background noise. The size of pp beads is not comparable to biological size of MPs, and thus conventional FCMs might not reliably detect MPs. In the search of MPs as potential biomarkers, technological improvements should not be underestimated.

**The paracrine messenger service of cardiac progenitor cells**

V. Verhage, K.R. Vrijsen, S.A.J. Chamuleau, P.A. Doevendans and J.P.G. Sluijter

Department of Cardiology, University Medical Center Utrecht, The Netherlands

*Introduction*: In the past, several types of stem cells have been used to regenerate myocardium. Among them, cardiomyocyte progenitor cells (CMPCs) appear to be the most promising cells source showing encouraging regenerative capacity after transplantation by intramyocardial injections. In perspective to the low retention of transplanted cells, different regenerative mechanisms seem to be involved which contribute to the preserved cardiac function and capillary formation after transplantation. One of these mechanism may be the secretion of paracrine signals by CMPCs, and thereby enhancing endogenous repair. Here we demonstrate that CMPCs secrete small bilayered vesicles, called exosomes, which can stimulate angiogenesis. *Material & Methods*: Exosomes were isolated from conditioned medium of CMPCs using differential centrifugation and characterised by NTA, electron microscopy and western blotting. Angiogenic models were used to investigate the effect of exosomes on endothelial cells in vitro and a matrigel plug assay was placed subcutaneously in C57BL/6 mice to examine the effect in vivo. *Results*: Vesicles derived form CMPCs showed presence of several exosomal characteristics, including an average size of 85 nm and expression of exosomal markers flottilin-1 and CD9 around a density of 1.12 to 1.16 g/ml. Analysis of exosomal function on endothelial cells revealed that exosomes are internalised by endothelial cells in vitro, and uptake stimulated endothelial cell sprouting, migration and network formation. In vivo, exosomes induced influx of CD31+ and smooth muscle actin+ cells. Furthermore, we detected vascular endothelial growth factor (VEGF) in exosomes, which is a known angiogenic signalling protein. *Conclusion*: The constitutively secretion of exosomes by CMPCs shows to be a distinct component of the paracrine signalling pathway by inducing angiogenesis both in vitro as in vivo, and implicats the use of exosomes as potential therapeutic for cardiac regene.

**Poster Session IV (Statler): Infection April 18**

**Chair: *M. Pegtel and E. Ligeti***

**Specific delivery of siRNA TO B cells by Epstein–Barr virus-like particles**

S. Jochum ^1^ and R. Zeidler^2^

^1^Helmholtz Centre, Munich, Germany; ^2^Ludwig-Maximilians University, Munich, Germany

Epstein–Barr virus (EBV) is a γ-herpesvirus with a strong tropism for CD21+ human B cells. More than 90% of the worldwide population are asymptomatic carriers of EBV as the virus persists in latently infected B cells that are not eradicated by the immune system. Eventual reactivation into the lytic phase results in the synthesis and spread of virus. Epstein–Barr virions consist of an encapsidated viral DNA genome, surrounded by a compartment termed tegument and an enveloping membrane, carrying viral glycoproteins that confer the tropism to the particles. Virus assembly and egress exploits the cellular ESCRT machinery and recapitulates morphogenesis and shedding of extracellular vesicles to a high extent. We found that RNA species are packaged into infectious EBV particles. During fusion of EBV particles with a B cell, this RNA content is released into the cytoplasm and deploys immediate function in the newly infected cell, considerably promoting the establishment of latency independently of de novo transcription. Using a recombinant system for virus production, we can generate EBV-like particles that share the tropism and fusogenic properties of infectious Epstein–Barr virions, but lack the viral genome and therefore cannot establish a reproductive infection in the target cell. Their distinct B-cell tropism renders these virus-like particles (VLPs) efficient tools to, for example, transfer RNAs of choice specifically into B cells. As a proof-of-concept, we loaded EBV-VLPs with siRNA targeting housekeeping messengers and infected PBMCs with these loaded VLPs. A thorough analysis revealed exclusive infection of B cells. Consequently, the knock-down of the targeted transcripts was restricted to B cells as well. In a next step, we will load EBV-VLPs with siRNAs that are known to influence growth and fate of malignant B cells, such as Burkitt lymphoma cells and CLL cells.

**Coxsackie virus entry and spread in HeLa cells is aided by microvesicle release**

Jameel Inal ^1^ and S Jorfi^2^

^1^London Metropolitan University, United Kingdom; ^2^Cellula and Molecular Immunology Research Centre, London Metropolitan University, United Kingdom

*Introduction*: Microvesicles (MVs) released from the plasma membrane are important vectors in intercellular communications. Coxsackievirus B (CVB), a member of the enterovirus family, is the main cause of meningitis and encephalitis in infants which may result in neurodevelopmental defects. Calpains are calcium-dependant cysteine proteases that degrade cytoplasmic and cytoskeletal proteins and regulate a variety of actin-dependant cellular processes such as microvesiculation. *Materials and methods*: Coxsackievirus B1 (CVB1) was obtained from the Health Protection Agency (HPA, UK) and expanded by infecting HeLa cells. CVB1 was purified through centrifugation, and titres were determined by plaque assay on HeLa cells. For infection, cells were inoculated with CVB1 at a multiplicity of 50 PFU/cell and kept at 37°C until fixation for flow cytometry or immunofluorescence assays were performed. Cells and cell-debris-free culture supernatants were filtered through a 0.22 μm pore size membrane filter. MVs were recovered from the filter by washing and centrifugation (25,000 g for 1h). *Results*: CVB1 requires calpain activation for both entry and virus replication. Here, we show that knocking down calpain, using approaches such as small interfering RNA (siRNA), culminates in reduction of MV release, as we showed before, with another intracellular pathogen, the protozoan parasite, *Trypanosoma cruzi*. The reduction in MV release then abrogates CVB1 entry and spread in HeLa cells. The calpain inhibitor calpeptin also caused similar reduction in CVB1 entry and spread to healthy target cells. *Discussion*: Together, our findings provide evidence that CVB1 infected HeLa cells enhance MV production, and these MVs aid the spread of infection. Furthermore, inhibition of MV release using siRNA results in inhibition of CVB1 entry and spread.

**Detection of PrPTSE in exosomal fractions isolated from various models of transmissible spongiform encephalopathies by protein misfolding cyclic amplification**

P. Saa, O. Yakovleva, J. De Castro and L. Cervenakova

American Red Cross, USA

*Introduction*: Transmissible spongiform encephalopathies (TSEs) are neurodegenerative disorders characterised by the accumulation of PrPTSE, a misfolded version of the cellular prion protein, PrPC. PrPC and PrPTSE have been identified in exosomes from various TSE models, and intracerebral inoculation of PrPTSE-containing exosomes has induced disease in mice. PrPC-bearing exosomes have been isolated from blood components raising the possibility that they may contain PrPTSE and serve as vehicles for transfusion transmission of TSEs. Presence of PrPTSE in exosomes will provide new grounds for the design of diagnostic assays. Historically, exosome isolation is achieved from large sample volumes which are not suitable for clinical application. We report proof-of-concept experiments demonstrating detection of PrPTSE by protein misfolding cyclic amplification (PMCA) in exosomal preparations from small sample volumes, which can lead to a novel blood-based diagnostic test. *Materials and methods*: Exosomes were isolated with SBI or Invitrogen reagents from 5 ml-conditioned media of cellular models infected with TSE strains, as well as from uninfected controls. Exosomal pellets were resuspended in normal mouse brain homogenates and amplified by serial automated PMCA (saPMCA), a method able to multiply as little as 100 PrPTSE molecules. After proteinase K digestion, PrPTSE was detected by Western blot using the anti-PrP monoclonal antibody 6D11. *Results*: PrPTSE was specifically detected in exosome preparations isolated from TSE-infected cells. Different number of saPMCA rounds had to be performed to amplify PrPTSE molecules to detectable levels for each cell line, probably reflecting differences in PrPTSE levels. *Conclusions*: Our experiments provide an invaluable foundation for the development of new diagnostics and potential targets for TSEs treatment. Demonstration of PrPTSE in blood-circulating exosomes may lead to the identification of the cellular origin of prion-containing microvesicles and the site(s) of prion replication in blood.

**Possible involvement of calprotectin in increasing HIV-1 infectivity and exosome release from lymphocytes**

Caroline Subra, P. T. Tessier Philippe and C. G. Gilbert Caroline

^1^Centre de Recherche du CHU de Québec, Université Laval de Québec, Canada

Exosomes are nanometer-sized vesicles released from immune cells as means of intercellular communication or material exchange. Striking similarities are noted between exosomes and the enveloped retrovirus HIV-1. Studies indicate that they may be involved in HIV-1 pathogenesis. We have shown previously that contacting dendritic cells or CD4+ T lymphocytes (CD4TL) with HIV-1 enhances the secretion of exosomes that trigger apoptosis of CD4TL. In advanced HIV-1-infected patients, increased serum level of the heterocomplex of S100A8 and S100A9, known as calprotectin, has been reported in early studies. Increased serum level of calprotectin is indicative of the systemic inflammation that marks several diseases. In vitro, double-stranded RNA has been shown to increase calprotectin secretion by monocytes and macrophages and calprotectin-enhanced HIV-1 replication in CD4TL. We hypothesise that HIV-1 infection increases calprotectin secretion, which in turn increases exosome release. Neutrophils and myeloid cells isolated from healthy donors were contacted with HIV-1 particles or inflammatory mediators such as interferon and TRL agonists. Calprotectin secretion was measured using an ELISA method, while exosome release was measured as acetylcholinesterase activity in Optiprep gradient fractions of culture supernatant. Infectivity of virions was evaluated by using TZM-bl indicative cell line. Calprotectin secretion by macrophages was increased following contact with either HIV-1 or inflammatory mediators. Both S100A9 and calprotectin increased exosome release by non-infected CD4TL. Virion production by CD4TL, stimulated with calprotectin, was increased, and the virions were more infectious. In conclusion, S100A9 and calprotectin might influence exosomes release and accelerate the progression of HIV-1 infection.

**Activated monocyte-derived exosomes mediate microRNA transfer to neural cells: implications for neurodysfunction in HIV/HCV co-infection**

Archana Gupta ^1^, B. Sun^2^, H. Rempel^2^, B. Wigdahl^1^ and L. Pulliam^2^

^1^Drexel University College of Medicine, USA; ^2^Veterans Affairs Medical Center, San Francisco, USA

*Introduction*: We recently reported that 60% of subjects with HCV and virally suppressed HIV infection (co-infection) were cognitively impaired. In addition, we showed that peripheral monocytes in co-infection had a distinct activation profile. Since HCV is not considered neurotropic, it suggests that indirect viral mechanisms may be responsible for neural cell dysfunction. Several lines of evidence indicate that exosomes facilitate cell-to-cell communication wherein host RNA and proteins can be transferred to recipient cells. We hypothesise that monocyte/macrophages (M/MF) cross the blood–brain barrier and shed exosomes-containing microRNAs into the perivascular space. The exosomes are internalised by neural cells releasing miRNAs, which subsequently dysregulate neural cell function. *Materials and methods*: Primary monocytes from healthy controls (n=3) and HIV-suppressed HCV co-infected subjects (n=5) were cultured overnight in exosome-depleted media. Exosomes were harvested using Exoquick and characterised by electron microscopy and protein markers. Identification and relative levels of exosomal miRNAs were determined using a hybridisation array with 381 probes of well-characterised human miRNAs. Internalisation of Dil-C16-labelled exosomes by neurons (MAP2) and astrocytes (GFAP) was visualised by confocal microscopy. *Results*: Monocyte-derived exosomes from co-infected subjects had significantly elevated levels (>2 fold) of 4 miRNAs, 18b, 146b, 218 and 224, with targets that regulate neural cell functions. Importantly, primary monocyte-derived exosomes were internalised by primary human astrocytes and neurons, thereby transferring miRNAs to neural cells. *Conclusions*: The differential miRNA profiles from co-infected subjects are consistent with an exosomal/miRNA pathway that targets neural cell function. The transfer of miRNAs from activated monocytes to neural cells via exosomes may explain, in part, the neurological abnormalities associated with HIV/HCV co-infection.

**Leishmania release exosomes containing short RNA sequences**

Ulrike Lambertz ^1^, M.O. Ovando^2^, P. Unrau^2^, G. Ramasamy^3^, P. Myler^3^ and N. Reiner^1^

^1^University of British Columbia, Canada; ^2^Simon Fraser University, Canada; ^3^Seattle Biomedical Research Institute, USA

*Introduction*: During infection with leishmania, impairment of host macrophage microbicidal function has been linked to transport of leishmania virulence factors into macrophage cytosol. We have identified exosomes as a mechanism for shuttling of leishmania proteins to host macrophages. Moreover, we showed that leishmania exosomes can modulate innate and adaptive immune responses. Recent studies with mammalian cells showed that their exosomes can shuttle RNA between cells. We thus hypothesized that leishmania exosomes, in addition to proteins, also contain RNAs, which may play a role in leishmania pathogenesis. *Materials and methods*: RNA was extracted from purified exosomes and analyzed by PAGE and Bioanalyzer. RNA extracts were used to construct short RNA librariers using a double ligation procedure followed by attachment of TruSeq adapters and paired end sequencing with an Illumina MiSeq instrument. *Results*: Our recent findings clearly show that exosomes released by *Leishmania donovani* and *Leishmania braziliensis* contain RNA. Interestingly, in both species the size range of exosomal RNA was considerably narrower than that of total RNA, with the vast majority of sequences being shorter than 200 nt. Sequencing resulted in ~1.5 million reads per library. For the *L. donovani* library, 47% of reads mapped to the leishmania genome. 55% of these sequences matched to non-coding RNAs (rRNA, tRNA and snoRNA). In addition, 44% of sequences mapped to regions of the genome that currently lack annotation, suggesting that *L. donovani* exosomes may contain novel, previously uncharacterized transcripts. More detailed analysis of the L. braziliensis RNAs is in progress. *Conclusion*: To our knowledge, this is the first demonstration of exosomes released by a protozoan pathogen containing RNA sequences. Current experiments are focused on understanding the function of these RNA sequences in leishmania biology, particularly pathogen-host interactions.

**Investigating a possible role for exosomes in antiviral response of LL5 cells from *Lutzomyia longipalpis*, the main visceral leishmaniasis vector in Brazil**

Yara M. Traub-Csekö ^1^, A. N. Pitaluga^1^, B. Burleigh^2^

^1^Instituto Oswaldo Cruz, Fiocruz, Brazil; ^2^Harvard School of Public Health, USA

*Lutzomyia longipalpis* is the major vector for visceral leishmaniasis in Brazil. We are studying immune responses of this vector. We have determined a role for the IMD pathway in the vector infection by Leishmania through the silencing of the repressor Caspar. We also observed a differential modulation of a defensin expression in relation to Gram-positive and Gram-negative bacteria and also to route of infection. We have previously identified a non-specific antiviral response in LL5 cells in response to transfection with double-stranded RNA (dsRNA). This was the first report of this kind of response in an insect cell line. We are presently identifying the mechanisms by which LL5 cells recognise dsRNA, using various approaches. We have performed deep-sequencing of transcripts from cells transfected with dsRNA or mock-transfected (control). We are also investigating the involvement of exosomes in the anti-viral response. Exosomes were observed both in transfected and mock-transfected cells by electron microscopy. No gross differences in protein composition were seen by 1-dimensional protein gels. The exosomes protein composition was determined by mass spectroscopy, and these data are under analysis. miRNAs were detected in the exosomal fractions. These are presently being sequenced in search for differential small RNAS expression in cells transfected with dsRNA.

**Dendritic cell immunoreceptor is involved in exosomes release**

Myriam Vaillancourt, C. Mukeba-Mfunyi, A. A. Lambert and C. Gilbert

Centre de recherche du CHU de Québec, Université Laval, Canada

Originating in endosomal compartments in haematopoietic and epithelial cells, exosomes are microvesicles that play a role in intercellular communication. Their release from dendritic cells coincides with the capture and transfer of human immunodeficiency virus (HIV-1). HIV-1 is known to bind to DCIR, a C-type lectin receptor expressed on the surface of dendritic cells, from which its transfer to CD4 T lymphocytes occurs. We studied the impact of HIV-1 binding on DCIR-associated signalling pathways, the role of this receptor in exosome release and the potential role of HIV-1-induced exosome release in HIV-1 pathogenesis. Exosome release from suitably stimulated DCIR-bearing cell lines is measurable by acetylcholinesterase activity in the extracellular microvesicle fraction. Raji-CD4-DCIR cells were stimulated with monoclonal anti-DCIR or virus. Markers of exosomes and membrane-associated materials were detected using immunoblots. Both types of stimulation increased exosome release from wild-type Raji-CD4, while release from the mutant cell line Y7F, in which phenylalanine replaces the tyrosine residue of the DCIR intracellular ITIM motif, was insignificant. Exosomes were purified using immunocapture with anti-acetylcholinesterase and used to stimulate neutrophils, CD4TL and Th17-polarised CD4TL. Exosomes released from HIV-1-infected cells increased apoptosis in CD4TL but not in neutrophils. Th17-polarised CD4TL were more sensitive to exosome-induced apoptosis than to apoptosis induced by reactive oxygen species. These results show that DCIR plays a signalling role in the production of exosomes and that the ITIM motif is involved. Exosomes derived from HIV-1-infected cells might play a role in HIV-1 pathogenesis by increasing apoptosis in Th17, a cell type involved in slowing the progression of the disease during the early stages of infection and, in particular, in maintaining the integrity of the intestinal mucosa.

**Gram-negative outer membrane vesicles elicit a potent innate immune response in the lung**

K.S. Park ^1^, J. Lee^1^, J. Lötvall^2^, Y.K. Kim^1^ and Y.S. Gho^1^

^1^Department of Life Science, Pohang University of Science and Technology, Republic of Korea; ^2^Department of Internal Medicine, Krefting Research Centre, University of Gothenburg, Sweden

*Introduction*: Gram-negative bacteria such as *Acinetobacter baumannii* and *Pseudomonas aeruginosa* are associated with nosocomial respiratory infection worldwide. It is known that these bacteria have the capacity to release outer membrane vesicles (OMVs), the bilayered proteolipids with diameters of ~20–250 nm. These OMVs are heterogeneous complexes of lipopolysaccharide, flagellin and outer membrane proteins that can be recognised by the innate immune system. In this study, we examined in detail the capacity of OMVs to induce innate immune response in the mouse lung. *Materials and methods*: OMVs were isolated from *A. baumannii* and *P. aeruginosa* cultures by ultracentrifugation. Purified OMVs were analysed by nanoparticle tracking and transmission electron microscopy. Mice were intra-nasally exposed to OMVs (10 µg), and then sacrificed at 0, 6, 12, 24 and 48h. Innate inflammation was assessed using histology and quantification of chemokines mRNA in the lung and adhesion molecules in serum. Cytokines production in vitro was studied in the lung epithelial cells and macrophages. *Results*: Nanoparticle-tracking analysis showed that the average diameters of OMVs from *A. baumannii* and *P. aeruginosa* are 158.7±11.8 and 152.3±11.6 nm, respectively. Both OMVs given to the mouse lung caused a time-dependent pulmonary cellular inflammation, characterised by increase of neutrophils and macrophages. OMVs increased the concentrations of CXCL1 and CCL5 mRNA in the mouse lung, and ICAM-1 and E-selectin in serum. Further, OMVs induced the release of IL-8 by lung epithelial cells and TNF-α and IL-6 by macrophages. *Conclusions*: This study shows that *A. baumannii* and *P. aeruginosa* OMVs induce innate immune response in the mouse lung. The role of OMVs in clinical disease warrant further studies, as bacterial OMVs in addition to live bacteria may be good therapeutic targets to control the pulmonary bacterial infection.

**Active immunisation with *Staphylococcus aureus*-derived extracellular vesicles protects pneumonia induced by Staphylococcus spp.**

S.J. Choi, W.H. Lee, O.Y. Kim, T.S. Shin, S.G. Jeon, Y.S. Gho and Y.K. Kim

Department of Life Science, Pohang University of Science and Technology (POSTECH), Republic of Korea

*Background*: In post-antibiotic era, the intensive use of antibiotics has dramatically increased the frequency of drug-resistance bacteria. Among these, *Staphylococcus aureus* is one of the most important bacterium, and methicillin-resistant *S. aureus* (MRSA) is a serious and growing threat in hospitals and also communities. Recently, we found that *S. aureus* can secrete vesicles into the extracellular milieu. *Objective*: To evaluate the effect of vaccination with *S. aureus*-derived extracellular vesicles (EVs) on the protection of pneumonia induced by *Staphylococcus spp*. *Methods*: *S. aureus*-derived EVs were isolated and immunised, via different routes, to 6-weeks-old C57BL/6 mice 3 times. *S. aureus*-derived EV-reactive IgG antibody in the serum and the production of T cell cytokines from splenocytes were measured after the immunisation. The effects of the immunisation with *S. aureus*-derived EV were evaluated in a pneumonia mouse model induced by *S. aureus* and *S. epidermidis*. *Results*: Immunisation with *S. aureus*-derived EVs induced the production of serum OMV-reactive IgG, irrespective of the immunisation routes. However, the production of IFN-gamma and IL-17 from splenocytes was induced by intramuscular (IM) route, but not by intraperitoneal (IP) or subcutaneous (SC) routes. *S. aureus*-induced pneumonia and lethality were effectively prevented by IM immunisation with *S. aureus* EVs, but not by IP or SC immunisation. Moreover, the IM immunisation with *S. aureus* EVs protects *S. epidermidis*-induced pneumonia. *Conclusion*: *S. aureus*-derived EV can be a good vaccine candidate with broad-spectrum and easy manufacturing.

**Exosomes released from *M. avium*-infected human macrophages are strong primers for ROS induction in human neutrophils**

S. Subramanian ^1^, J. Fischer^1^, S. Winter^1^, J. Rybniker^1^, N. Robinson^2^ and P. Hartmann^1^

^1^Infectious Diseases Lab, Department of Internal Medicine, University Hospital of Cologne, Cologne; ^2^Institute for Medical Microbiology, Immunology and Hygiene, University Hospital of Cologne, Cologne, Germany

*Introduction*: Neutrophils are rapidly recruited to the site of mycobacterial infections; however, they are less well studied than macrophages known as the natural host cells of mycobacteria. Some of the previous studies have shown that human neutrophils are capable of killing avirulent *M. avium*, a ubiquitous opportunistic pathogen. Virulent strains of *M. avium*, however, are less effectively eliminated. We are interested in how exosomes released from *M. avium*-infected macrophages act on neutrophils and whether virulence of *M. avium* does have a role with regard to the functional capacities of exosomes released from macrophages. *Materials and methods*: Human-monocyte-derived macrophages (HMDM) were infected with clinical isolates of *M. avium*. Uninfected and latex-bead-fed macrophages served as control. Exosomes were purified from equal volumes of supernatants by sucrose density gradient and ultra centrifugation and further characterised by surface markers (MHC-I, MHC-II, HSP70) and electron microscopy. So far we analysed the potential of macrophage-derived exosomes to act on human neutrophils with regard to chemotaxis in multiwell Boyden chambers and induction of reactive oxygen species (ROS) by luminol assay. *Results*: *M. avium*-infected macrophages released more exosomes than uninfected or bead-fed macrophages. However, there was no difference in exosome release induced by avirulent and virulent *M. avium*. Exosomes released from *M. avium*-infected macrophages were chemoattractive for neutrophils independently of the strain's virulence. Exosomes themselves were not capable of inducing ROS in neutrophils; however, upon priming with 25 µg/ml of exosomes for 30min, ROS production was significantly higher after stimulation with fMLP. *Conclusion*: Our data indicate that exosomes released from macrophages upon infection with avirulent as well as virulent *M. avium* strains recruit neutrophils to the site of infection and are capable of inducing a pro-inflammatory phenotype.

**Functional characterisation of dendritic cell-derived exosomes during DENV infection**

Sharon Martins, M.J. Soares, C.N. Duarte dos Santos, S. Goldenberg, J. Bordignon and L.R. Alves

Fundação Oswaldo Cruz (FIOCRUZ), Brazil

*Introduction*: Dengue virus (DENV) is the most important arboviral infection in the world and cause dengue fever or even more severe clinical outcomes such as dengue haemorrhagic fever and dengue shock syndrome. Dendritic cells (DCs) are important targets of DENV, being crucial on the activation of adaptive immune responses. Exosomes are secreted vesicles derived from the endocytic pathway, and its composition seems to be very specific in infectious conditions, suggesting that the exosomal pathway can be used to enhance immune responses or to favour viral evasion. This work aims to characterise the composition and immune properties of exosomes derived from DCs infected with 2 DENV3 strains with different infection phenotypes, relating exosomal composition with immunological outcomes and disease severity. *Materials and methods*: Monocyte-derived dendritic cells were generated from CD14+ cells isolated from peripheral blood of healthy donors and infected with dengue virus strains DENV3 5532 or DENV3 290, in distinct times and moieties of infection (MOI). DC-derived exosomes were isolated from culture supernatants by centrifugation, filtration and ultracentrifugation steps, and used for further RNA content characterisation. Total RNA was analysed by next generation sequencing using SOLiD sequencer, and CLC workbench was used for data analysis. *Results*: In this work, we showed that DC-derived vesicles contain mRNAs and miRNAs and that vesicles derived from cells infected with DENV3 5532 strain (that is known to induce more apoptosis on infected and bystander cells than DENV3 290 strain) contains mRNAs related to apoptotic processes, while infection with DENV3 290 does not generate vesicles containing mRNAs involved in this pathway. *Conclusions*: We suggest that DC-derived exosomes can carry apoptotic signals to bystander cells during DENV3 5532 infection. Further studies will be necessary to determine the role of DC-derived exosomes during DENV infection.

**Discrete pathogenic roles of plasma membrane microparticles in experimental cerebral malaria**

F. El-Assaad^1^, A. Cohen^1^, C. Cassano^1^, G. E. R. Grau^1^ and V. Combes ^2^

^1^The University of Sydney, Australia; ^2^La Jolla Infectious Diseases Institute, The University of Sydney, Australia

Microparticles (MPs) are submicron plasma-membrane-derived vesicles involved in coagulation, inflammation, cell adhesion and cell–cell communication. MP numbers are increased in cerebral malaria (CM) patients and in the corresponding murine model. In the latter, we have shown that blocking MP release genetically or pharmacologically confers protection against CM, suggesting a role of MP in neuropathogenesis. The in vivo production, cellular origins, fate, pathogenicity and miRNA contents of MP during *Plasmodium berghei*-ANKA infection of CM-susceptible (CM-S) and CM-resistant (CM-R) mice were evaluated. Upon infection, CM-S but not CM-R mice had raised plasma levels of MP, predominantly of platelet, endothelial and erythrocytic origin. To determine the in vivo fate of MP in CM-S mice, we adoptively transferred labelled MP from mice with CM into normal or infected recipient mice. Circulating MPs were quickly cleared following i.v. injection, and immunofluorescent imaging showed arrested MPs lining the endothelium of brain vessels of infected, but not healthy, recipient mice. Intravenously transferred MP from TNF-activated endothelial cells induced brain and lung pathology in uninfected CM-S recipient mice. MPs are known to interact with, and modify, their target cells. Of increasing interest is the miRNA content these MPs can transfer. We purified MPs from the blood of infected and healthy CM-S mice and analysed their miRNA using microarrays. Of the 1,111 miRNA present on the array, in infected vs. healthy samples, respectively, 260 vs. 248 were detected, 47 were common and 85 vs. 73 were unique. Among those in common, differences in expression were detected, particularly in the miRNA involved in controlling Th1 responses (pro-inflammatory cytokine induction), apoptosis, homeostasis and immunomodulation. These data warrant further investigation into the representation of MPs as a marker of CM risk and suggest an active role for MPs in the pathogenesis of neurological lesions.

**Exosomes isolated from ACS carry antimicrobial peptides**

S. Amin^1^, P. Wehling^2^, Y. B. Erol^1^, M. A. Sarker^1^, D. Aubert^3^, S. Irsen^4^, J. Feydt^4^, P. Lou^5^, J. Reinecke^2^ and M. Weisshaar ^1^

^1^University of Applied Science Bonn-Rhein-Sieg, Germany; ^2^Orthogen AG, Germany; ^3^IZON, United Kingdom; ^4^CAESAR, Germany; ^5^Izon, United Kingdom

*Introduction*: Microvesicle (MV) is a collective term for a broad range of membrane particles including exosomes, shedding vesicles and apoptotic blebs, which are released from and taken up (by fusion, endocytosis, or target-receptor interactions, e.g. via tetraspanins) by most cell types. MVs have been identified in many body fluids, including blood, breast milk, cerebrospinal fluid, saliva and urine. MVs carry various cargos from their mother cells, including lipids, specific protein and nucleic acid (DNA, mRNA and miRNA), which can be translated into functions in the recipient cells. Proteins, mRNA and miRNA may be sorted into some MVs such as exosomes in a selective and energy-dependent manner. The objective of this study is to show that exosomes isolated from autologous conditioned serum (ACS) carry antimicrobial peptides. *Methods*: In our study the production of ACS involves the incubation of the autologous, venous blood in the presence of medical glass beads at 37°C during an increasing time scale like 3, 6, 9, 12 and 24h. The antibacterial activity was measured by agar diffusion test using *E. coli* K12. *Results*: The qNano measurements confirmed that the highest amount of exosomes in ACS was found after 9h of incubation: 831,010 vesicles per millilitre with a mean size of 165 nm. With longer incubation, there is a mixture of MVs, disrupted exosomes and aggregates between 280–400 nm. The agar diffusion test showed that the antibacterial activity in exosomes diluted in water is highest after 6-h incubation with an inhibition zone from 10.9 mm (r:0.95 mm). Heated samples showed no antibacterial activity at all as well as the supernatant and the serum itself due to heat denaturation of the peptides-qNano analysis showed also that the supernatant had no vesicles at all, but a high antibacterial could be detected, indicating the presence of the peptides this could also be demonstrated by Ninosight analysis. *Conclusions*: ACS contains exosomes carrying peptides with antibacterial activity, so we can conclude that serum exosomes may play a role as carrier for anti-microbial peptides.

**Outer membrane vesicles derived from Gram-negative bacteria induce vascular dysfunction by up-regulation of endothelial ICAM-1 expression and leukocyte infiltration**

J. H. Kim ^1^, Y. J. Yoon^2^, J. Lee^1^, E. J. Choi^1^, N. Yi^3^, K. S. Park^1^, J. Park^3^; J. Lötvall^4^, Y. K. Kim^1^ and Y. S. Gho^1^

^1^Department of Life Science, Pohang University of Science and Technology, Republic of Korea; ^2^School of Interdisciplinary Bioscience and Bioengineering, Pohang University of Science and Technology, Republic of Korea; ^3^Department of Mechanical Engineering, Pohang University of Science and Technology, Republic of Korea; ^4^Department of Internal Medicine, Krefting Research Centre, University of Gothenburg, Sweden

*Introduction*: An important feature of Gram-negative bacteria is their ability to constitutively release outer membrane vesicles (OMVs), 20–200 nm spherical bilayered proteolipids. These OMVs harbour several bacterial virulence factors and pathogen-associated molecular patterns, which can induce severe systemic inflammatory response. However, the effect of OMVs in endothelial ICAM-1 expression and leukocyte infiltration into tissues has not been previously studied. *Materials and methods*: From the culture supernatant of *Escherichia coli*, OMVs were isolated by a 150,000 g ultracentrifugation. ICAM-1 expression was studied in vitro in human umbilical vein endothelial cells, human coronary artery endothelial cells, human dermal microvascular endothelial cells and HMEC-1 cells. Mice were intraperitoneally, intravenously or subcutaneously exposed to OMVs, and endothelial ICAM-1 expression was assessed using immunohistochemistry. *Results*: Bacteria-free OMVs derived from *E. coli* increased the ICAM-1 expression and enhanced leukocyte binding on human endothelial cells in vitro dose-dependently. The OMV-induced ICAM-1 expression was blocked by cycloheximide (a protein synthesis inhibitor) and BAY11-7082 (NF-κB inhibitor), but not by the inhibitors of ERK (PD98059), JNK (SP600125) and p38 MAPK (SB203580). Moreover, intraperitoneally, intravenously or subcutaneously injected OMVs increased ICAM-1 expression in endothelial cells and the number of infiltrated neutrophils. When compared to wild-type mice, this OMV-induced inflammation was reduced in ICAM-1−/− and TLR4−/− mice. Furthermore, we showed that OMVs were more potent than free lipopolysaccharide in these ICAM-1 expression in vitro and inflammatory response in vivo. *Conclusions*: This study shows that OMVs may be involved in vascular dysfunction during bacterial infection.

**Poster Session IV (Statler): Inflammation April 18**

**Chair: *M. Piper and S. Gabrielsson***

**Microparticle-associated immune complexes predominate in rheumatoid arthritis synovial fluid**

N. Cloutier ^1^, S. Tan^2^, L. H. Boudreau^1^, C. Cramb^3^, R. Subbaiah^4^, L. Lahey^3^, A. Albert^1^, R. Shnayder^5^, R. Gobezie^6^, P. A. Nigrovic^5^, R. W. Farndale^7^, W. H. Robinson^3^, A. Brisson^2^, D. L. Lee^5^ and E. Boilard^1^

^1^CHUQ Research Center, Université Laval, Québec, Canada; ^2^Molecular Imaging and NanoBioTechnology, University Bordeaux, Bordeaux, France; ^3^Department of Medicine, Stanford University School of Medicine, Stanford, USA; ^4^Department of Orthopaedics, Case Western Reserve University, Cleveland, USA; ^5^Division of Rheumatology, Immunology and Allergy, Brigham and Women's Hospital, Boston, USA; ^6^The Cleveland Shoulder Institute, University Hospitals of Cleveland, Cleveland, USA; ^7^University of Cambridge, Downing Site, Cambridge, United Kingdom

*Introduction*: Immunoglobulins, antigens and complement can assemble to form immune complexes (ICs). ICs can be detrimental as they propagate inflammation in autoimmune diseases. Like ICs, submicron extracellular vesicles termed microparticles (MPs) are present in the synovial fluid from patients affected with autoimmune arthritis. *Materials and methods*: We examined MPs in rheumatoid arthritis (RA) using high sensitivity flow cytometry and electron microscopy. *Results*: We find that the MPs in RA synovial fluid are highly heterogeneous in size. The observed larger MPs were in fact MP-containing ICs (mpICs) and account for the majority of the detectable ICs. These mpICs frequently express the integrin CD41, consistent with platelet origin. Despite expression of the Fc receptor FcγRIIa by platelet-derived MPs, we find that the mpICs form independently of this receptor. Rather, mpICs display autoantigens vimentin and fibrinogen, and recognition of these targets by anti-citrullinated peptide antibodies contributes to the production of mpICs. *Conclusions*: Taken together, our data demonstrate an unappreciated feature for ICs and MPs in RA and reveal that platelet MPs can be a source of autoantigens.

**Complement deposition on endothelial microparticles in vasculitis**

Maria Mossberg ^1^, R. Kahn^1^, A. Ståhl^1^, C. Heijl^2^ and D. Karpman^1^

^1^Department of Pediatrics, Lund University, Sweden; ^2^Department of Nephrology, Lund University, Sweden

*Introduction*: Vasculitis is an inflammatory condition in and around vessel walls causing secondary tissue damage to various organs. Endothelial microparticles liberate during the acute phase of vasculitis. In vivo and in vitro data suggest the involvement of the complement system which may play a role in the subsequent inflammation. *Materials and methods*: In this study, plasma samples from children and adults with vasculitis (n=14) and controls (n=13) were investigated for the presence of microparticles from endothelial cells. Microparticles of endothelial origin were identified by flow cytometry on the basis of their size and the presence of endothelial cell markers, CD105 and CD144. These microparticles were assayed for the presence of complement C3 and C9. *Results*: Results showed that patients with acute vasculitis had higher levels of endothelial microparticles, median 1779 microparticles/µl plasma (range 394–6012) compared to controls 30 microparticles/µl plasma (range: 0–623), p<0.05. C3 was present on endothelial microparticles, median 662 microparticles/µl (range: 0–1606). A subset also expressed C9 on their membrane, 693 microparticles/µl plasma (range: 53–1617). Control samples expressed significantly lower levels of C3, 13 (0–209), and C9, 5 (0–220), p<0.05 and p<0.001, respectively. *Conclusions*: C3 and C9 are present on circulating endothelial microparticles in vasculitis. These results indicate complement activation on the endothelium which may contribute to the cell damage and promote inflammation. Future studies will elucidate the role the complement system has on circulating endothelial microparticles during acute vasculitis.

**Patients with vasculitis express kinin B1-receptors on circulating leukocyte-derived microparticles**

Robin Kahn, M. Mossberg, A. Ståhl and D. Karpman

Lund University, Sweden

*Introduction*: Kinin B1-receptors are upregulated during chronic inflammation and ligand binding leads to inflammation, pain and capillary leakage. The B1-receptor has been associated with neutrophil migration to sites of inflammation and has been shown to be essential for inflammation in animal models. Vasculitis is a chronic inflammatory condition typically presenting with leukocyte infiltration and subsequent tissue damage. Both endothelial and leukocyte microparticles have been shown to be upregulated during acute vasculitis but the presence of B1-receptors on microparticles has not been studied. *Materials and methods*: Patients presenting with acute vasculitis (n=9) were included in the study. The patients were not receiving any immunosuppressive treatment at time of sampling. Citrated plasma was taken at the onset of disease. Plasma from healthy individuals (n=3) was used as control. The presence of B1-receptors on leukocyte-derived microparticles in plasma was analysed by flow cytometry. Microparticles staining for CD14 were considered to be of monocyte origin, CD66 of neutrophil origin, CD19 of B cell origin and CD3 of T cell origin. Levels of microparticles are shown as numbers of microparticles per microlitre of plasma. *Results*: The patients had high levels of leukocyte-derived microparticles expressing the B1-receptor (median 1450/µL, range 226–1980). The microparticles were predominantly of monocyte- (median 420/µL, range 58–1130), neutrophil- (median 274/µL, range 72–971) and B cell-origin (median 278/µL, range 22–540) although some T cell-derived microparticles (median 101/µL, range 28–215) were also seen. The healthy individuals had low levels of leukocyte-derived microparticles (median 56/µL, range 50–80). *Conclusions*: We demonstrate B1-receptor expression on microparticles derived from leukocytes. Patients with vasculitis had high levels of B1-receptor expressing microparticles as compared with health controls.

**Platelet microparticles: key roles in transcellular communication, and alterations in miRNA and protein composition in type 2 diabetics**

Sherry Spinelli, A. E. Casey, N. Blumberg and R. P. Phipps

University of Rochester Medical Center, USA

*Introduction*: Microparticles (MPs) are submicron-sized membrane vesicles released into the blood by platelets, and other vascular cells, and contain cellular information in the form of bioactive proteins, nucleic acids, lipids and other molecules. Platelet MPs are the dominant MP source in plasma and are elevated in inflammatory diseases. Our group has shown that platelet MPs transport transcription factors (e.g. peroxisome proliferator activated receptor gamma [PPARγ]), in addition to key cytokines, tissue factor, regulatory RNAs and other pro-inflammatory and prothrombotic molecules. Because MPs are key players in inflammation and thrombosis, we are investigating the mechanisms of platelet MP messaging that direct the aberrant pathophysiology associated with inflammatory diseases, such as type 2 diabetes and cardiovascular disease. *Materials and methods*: Unit donations from type 2 diabetics and healthy individuals were obtained, and platelets were isolated by centrifugation. Platelet MPs were generated from washed platelets following activation and labelled for uptake determination in human monocytes and mouse fibroblasts. Protein and RNA were isolated from both platelet and MP pellets for proteome analysis and miRNA profiling. RNA purity was determined by PCR to confirm the absence of white cell contamination. *Results*: Our data demonstrate that (a) platelet MPs are internalised by target monocytes and endothelial cells, and impact their function, and (b) healthy and diabetic MP payloads released from platelets are different in both protein and miRNA content (diabetics are pro-inflammatory). *Conclusions*: Findings from our continuing studies will advance our understanding of the pathophysiology of type 2 diabetes, benefit early disease detection and allow us to develop new methods to monitor patient progress when attempting disease intervention through drug therapy, diet and exercise.

**Microvesicles as a magnifying glass: uncovering potential biomarkers in juvenile idiopathic arthritis**

Genoveva Keustermans, W. de Jager and B. J. Prakken

University Utrecht Medical Center, The Netherlands

*Introduction*: Juvenile idiopathic arthritis (JIA) is a common chronic inflammatory diseases in childhood. Although generally manifested as an inflammation of the joints, some JIA patients exhibit uveitis, a severe inflammation in an immuno-privileged zone. The localised inflammation and fluctuating progression and regression profile of patients require the implementation of a plethora of treatment techniques. Despite remission as a result of the use drugs, the chronic and relapsing nature of the disease requires continuous treatment which, in itself, causes adverse side effects. It is important to uncover a biomarker that can efficiently predict patient responses to therapy as well as determine if patients will progress or regress as a result of treatment. Microvesicles are key messengers containing many immune signalling molecules including cytokines, molecules known to play a major role in JIA. Due to the localised inflammation and uveitis phenomenon seen in JIA, we aim to analyse if micro vesicles isolated from patients can provide a source of biomarkers, giving specific information on molecules that can be targeted for treatment and allow the disease state to be monitored. *Materials and methods*: Microvesicles were isolated from the blood and synovial fluid of patients with various subtypes of JIA. Vesicular protein profiles were then compared using Luminex technology. *Results*: Pilot data showed that plasma microvesicles from polyarticular JIA patients have a more pronounced inflammatory phenotype, expressing higher levels of TNF, IFN, MIG and IP10. This increased level may explain the more severe disease profile of polyarticular versus oligoarticular patients. *Conclusions*: Preliminary data seem to indicate a difference in information carried within the microvesicles of individuals with JIA, strengthening the hypothesis that microvesicles carry important information regarding the inflammatory process.

**Impact of human BALF exosomes on bronchial epithelial cells: smokers vs. non-smokers**

P.J. Martin ^1^, C. Lepers^1^, M. Hille^1^, A. Verdin^1^, S. Firmin^1^, F. Roy Saint-Georges^2^, S. Billet^1^ and P. Shirali^1^

^1^Unité de Chimie Environnementale et Interactions sur le Vivant (EA4492), ULCO, Dunkerque, France; ^2^Service de Pneumologie, Groupe Hospitalier de l'Institut Catholique de Lille, France

*Introduction*: Oxidative stress and inflammation are key contributors to chronic obstructive pulmonary disease (COPD) pathogenesis and lung carcinogenesis caused by cigarette smoke. The clinical expression of lung diseases in smokers is not well explained by currently defined variations in gene expression or simple differences in smoking exposure. To understand smoking-related diseases, it is important to measure changes that occur with chronic cigarette smoke exposure. Several studies have recently shown the presence of exosomes in bronchoalveolar lavage fluid (BALF) and their implication in asthma and sarcoidosis. However, their role in smoking-related diseases is unknown. Our aim was to investigate whether exosomes from smokers and non-smokers BALF have different influence on the normal human bronchial epithelial cells BEAS-2B phenotype. *Materials and methods*: BEAS-2B cells were exposed to BALF exosomes isolated from healthy patients either smoker or non-smoker. RT-qPCR was used to measure XME and inflammatory gene expression as well as miRNA. Cytokines in cell culture supernatant were quantified by ELISA. *Results*: BEAS-2B exposed to BALF exosomes showed increased XME genes expression (e.g. CYP1B1 and NQO1, p<0.01 and p<0.05, respectively). We could also show that exosomes from smokers lead to a decrease of the potential anti-oncogenic miRNA miR-26b expression and a significant increase of the pro-inflammatory cytokine IL-8 production by BEAS-2B, compared to non-smokers (p<0.05). *Conclusion*: BALF exosomes from smokers might contribute to lung inflammation and oxidative stress by increasing XME genes expression and cytokine generation in airway epithelium.

**Gutmicrobiota-derived extracellular vesicles are an important causative agent in the development of insulin resistance by high-fat diet**

Y. Kim, Y. Choi, Y. Kwon, S. Hong; S. G. Jeon, S. Ryu and Y. S. Gho

POSTECH, Republic of Korea

*Introduction*: The gastrointestinal tract is the primary site of interaction between the host immune system and microorganisms, both symbiotic and pathogenic. Diet can alter the composition of gut microflora. Extracellular vesicles (EVs) are key mediator to determine gut microbiota and host interaction. *Hypothesis*: Gutmicrobiota-derived EVs are a key mediator in the development of high-fat diet (HFD)-induced metabolic dysregulation. *Methods*: To evaluate the role of gutmicrobiota-derived EVs on the development of insulin resistance, stool EVs from regular chow diet (RCD) and HFD-fed mice, and EVs from specific bacteria were isolated, and then insulin resistance was evaluated. Metagenomics from stool samples was performed to identify bacterial source of EVs. *Results*: Stools harboured lots of EVs from Gram-positive and Gram-negative bacteria, and host cells. The size and amount of stool EVs were decreased by HFD compared to RCD. Metagenomic analysis showed that the proportion of Gram-positive and Gram-negative bacteria in stools was similar between RCD and HFD mice. However, in terms of the source of EVs in stools, proteobacteria phylum was predominant in both RCD and HFD mice. Among the proteobacteria phylum, *Pseudomonas spp*: are the main source of stool EVs, and *P. extremaustralis-* and *P. panacis*-derived EVs were increased, but *P. cedrina* EVs decreased by HFD. Furthermore, in vitro and in vivo application of *P. panacis* EVs induced insulin resistance, whereas *P. cedrina* EVs did not. *Conclusion*: The present data suggest that gutmictobiota-derived EVs play an important role in the development of insulin resistance and then type 2 Diabetes.

**Alpha-hemolysin in extracellular vesicles derived from *Staphylococcus aureus* plays an important role in the development of atopic dermatitis-like skin inflammation**

S.W. Hong ^1^, E.B. Choi^1^, B.Y. Pyun^2^, Y.S. Gho^1^ and Y.K. Kim^1^

^1^POSTECH, Republic of Korea; ^2^Suncheonhyang hospital, Republic of Korea

*Introduction*: Atopic dermatitis (AD) is a chronic inflammatory skin disease. Although the pathogenesis of atopic dermatitis is not clear, skin barrier disruption and colonisation with *Staphylococcus aureus* are thought to be major causative factors. *S. aureus* may affect skin inflammation by secreting toxins, such as α-hemolysin, which is a component of *S. aureus*-derived extracellular vesicles (EVs) based on recent proteomics. *Objective*: To evaluate the role of α-hemolysin in *S. aureus* EVs on the development of atopic dermatitis. *Methods*: The presence of α-hemolysin in EVs was detected by western blot. To compare the cytotoxic effect on keratinocytes, cell viability and lactate dehydrogenase were measured after treating soluble α-hemolysin and α-hemolysin-containing EVs. Then, keratinocytes were stained with annexin V and 7-AAD to discriminate the type of cell death. By administrating α-hemolysin and EVs to tape-stripped mouse skin, skin pathology was evaluated. *Results*: α-Hemolysin-positive *S. aureus* secreted EVs, which harbour α-hemolysin. α-Hemolysin-positive EVs were more cytotoxic to keratinocytes compared to soluble α-hemolysin, although both were cytotoxic. In addition, α-hemolysin-negative EVs did not induce keratinocyte death. In terms of the cell death type, α-hemolysin-positive EVs induced keratinocyte necrosis, whereas soluble α-hemolysin induced keratinocyte apoptosis. As for the in vivo effects, α-hemolysin-positive EVs induced atopic-dermatitis-like skin inflammation in tape-stripped mouse skin, whereas α-hemolysin-negative EVs did not. In addition, soluble α-hemolysin induced only epidermal hyperplasia, but not dermal inflammation. *Conclusion*: α-Hemolysin in EVs secreted from *S. aureus* is important in the development of atopic dermatitis.

**Malaria microvesicles are potent messengers that induce physiological responses in immune cells and parasites**

Pierre-Yves Mantel ^1^, A. Hoang^2^, I. Goldowitz^1^, D. Potashnikova^3^, B. Hamza^2^, I. Vorobjev^3^, I. Ghiran^4^, M. Toner^2^, D. Irimia^2^, A. Ivanov^5^, N. Barteneva^3^ M. Marti^1^

^1^Harvard School of Public Health, USA; ^2^Massachusetts General Hospital, USA; ^3^Children's Hospital Boston, USA; ^4^Beth Israel Deaconess Medical Center, USA; ^5^Northeastern University, USA

Human malaria represents a combination of syndromes such as anaemia, parasite dependent and independent activation of a pro-inflammatory response, and the recruitment and infiltration of inflammatory cells. Emerging key molecules in these processes are microvesicles released from parasite-infected red blood cells (RMVs). Here we present the first detailed characterisation of RMV composition and function in the human malaria parasite *Plasmodium falciparum*. Proteomics profiling of RMVs revealed enrichment of multiple host and parasite proteins, in particular, parasite antigens associated with host cell membranes and proteins involved in parasite invasion into red blood cells. We show that RMVs are quantitatively released during the asexual parasite cycle but before egress. Moreover, RMVs are internalised by infected red blood cells, and they stimulate production of transmission stage parasites in a dose-dependent manner, suggesting an important role in cellular communication between parasites and with the host innate immune system.

**Microvesicle-mediated interaction between T. cruzi and host cells: effects on gene expression**

Florencia Cabrera-Cabrera, M.R. García-Silva, P. Zorrilla and A. Cayota

Institut Pasteur of Montevideo, Montevideo, CP, Uruguay

*Introduction*: Microvesicles (MVs) of trypanosomatid carry virulence factors that affect regulatory and signalling pathways in the host cell. *T. cruzi* invasion of MV-treated cells is 5-fold greater than in non-treated ones. Our group showed that *T. cruzi* MV content is only transferred to cells susceptible to infection, yet when non-susceptible cells artificially incorporate MV content, they become susceptible. These data suggest that parasite MVs may be preparing host cells for infection. This work focused on identifying changes in the host cell gene expression induced by MVs. *Material and methods*: *T. cruzi* MVs were obtained by ultracentrifugation of the culture media of nutrient-deprived epimastigotes. HeLa cells were incubated with MVs, and RNA was extracted at 6, 24 or 72 h post-interaction and processed for microarray analysis. *Results*: Nearly 5,000 genes were differentially expressed in at least 1 of the 3 conditions assayed when compared to non-treated cells. About 700 had an absolute fold change ≥3. Gene expression profiles of cells at 6 and 24 h post-treatment were more similar to each other than to those of non-treated cells or at 72 h post-treatment, indicating that at this point, modifications elicited by MVs were reversed. Affected genes were involved in signalling pathways, such as that of Wnt, which could lead to cytoskeleton rearrangements and increased Ca2+ levels, two phenomena crucial for successful infection. Processes such as response to external stimuli, vesiculation and lipid metabolism seem to be up-regulated. The formation of vesicles during the entry of the parasite is also a key point during infection. A swift to energy production from lipids has been reported during *T. cruzi* infection. *Conclusions*: Pathogenic MVs are capable of modifying the host cell environment in order to make it more propitious for infection. The changes that occur in the cell fade in time, probably due to the lack of infection in our experimental setting.

**Cigarette smoking induces an increase in neutrophil/monocyte microvesicles in susceptible subjects**

L. Cantone ^1^, M. Hoxha^1^, L. Angelici^1^, M. Di Francesco^2^, L. Pergoli^1^, L. Vigna^3^, V. Dolo^2^, P.A. Bertazzi^1^, A.C. Pesatori^1^ and V. Bollati^1^

^1^University of Milan, Italy; ^2^University of L’Aquila, Italy; ^3^Fondazione IRCCS Ospedale Maggiore Policlinico, Mangiagalli e Regina Elena, Italy

*Introduction*: Cigarette smoking is a major risk factor of cardiovascular disease (CVD), especially in those subjects showing susceptibility conditions, such as obesity. Tobacco smoke is known to increase inflammation in the lungs, possibly through the release of proinflammatory cell-derived membrane vesicles (MVs). MVs might be the ideal candidate to mediate the effects of tobacco smoke, since they could be produced by the respiratory system, reach the systemic circulation and get involved in systemic diseases. *Material and Methods*: Smoking habits information and blood sample were collected from 106 overweighed/obese (32 males and 74 females) subjects enrolled at the Obesity and Work Outpatients Clinic (University of Milan-Italy). Study subjects were categorized as current (CS), former (FS) and never smokers (NS). Plasma MVs were isolated by centrifugation at 4°C to eliminate debris, and ultracentrifugation (UC) for 75' at 110,000×g at 4°C. MVs enrichment was confirmed by transmission electron microscopy. MVs were immunophenotyped by flow cytometry according to their cellular origin, using AbCD66 (neutrophils), AbCD14 (monocytes) and AbCD61 (platelets). Data set was analysed using the FlowJo software (TreeStar, USA). Univariable and multivariable regression analysis adjusted for sex and body mass index (BMI) were applied to investigate the relationship between variables. *Results*: MVs CD66+ and CD61+ were positively associated with neutrophil (Δ%=11.1; p=0.05) and platelet count (Δ%=0.4; p=0.02) respectively. CD66+ and CD14+ MVs were increased in CS compared with NS (Δ%=−43; p=0.02 and Δ%=−44; p=0.05 respectively). Moreover, CD66+ MVs showed a positive trend moving from NS to FS to CS (p-trend=0.04). No significant interactions were found between smoking and sex, and smoking and BMI. *Conclusions*: Our findings showed that cigarette smoking may induce alterations in MVs release, thus suggesting a possibly novel biological mechanism linked to CVD risk factors.

**Poster Session IV (Statler): Therapy April 18**

**Chair: *D. Gupta and R. Schiffelers***

**Sequences within RNA coding for HIV-1 GAG p17 are efficiently targeted to exosomes**

Claudia Arenaccio, Paola Di Bonito, Maurizio Federico and Sandra Columba Cabezas

Istituto Superiore di Sanità, Rome, Italy

The convergence of exosome and HIV biogenesis would imply that viral products can be incorporated in exosomes. This was already proven for Gag HIV-1 protein. HIV-1 Gag molecules associate with exosomes seemingly by virtue of their higher-order oligomerisation. Conversely, no conclusive evidences regarding possible association of HIV-1 RNA with exosomes have yet been reported. To analyse HIV-1 incorporation in exosomes, we use HXBH10 HIV-1 clone, and its derivatives Gag defective mutants within the p17 matrix N-terminus have been employed. The pNL4-3.Luc and its DSL3 derivative were used for analysing the in?uence of the HIV-1 packaging signal in viral genome exosome incorporation. For the ectopic expression of HIV-1557–663 sequences, these have been PCRamplified from the HXBH10 HIV-1 molecular clone using Gag-specific primers cloned in pTarget vector in either sense or antisense orientations in accurate sequence. Here we report evidences that full-length, but not single or double spliced, HIV-1 RNA species are incorporated in exosomes. Deletion mutant analysis indicated that the presence of stretch sequences was found associating with exosomes also out of the HIV-1 context, thus indicating that the presence of stretch sequences within the 5’ end of the Gag p17 open-reading frame is sufficient for the incorporation of HIV-1 RNA in exosome. These sequences were found associating with exosomes also out of the HIV-1 context, thus indicating the diversion towards the vesicular compartment can occur without the need of additional HIV-1 sequences. Finally, the incorporation of genomic HIV-1 RNA in exosomes significantly increased when producer cells express HIV-1 defective for viral genome packaging. Considering the already documented activity of RNA molecules following exosome-mediated delivery in target cells the transmission by exosomes of active HIV-1 RNA molecules to bystander cells would have relevant biologic significances.

**In vivo biodistribution of exosomes**

O.P.B. Wiklander ^1^, J.Z. Nordin^1^, P. Vader^2^, I. Mäger^2^, L. Alvarez-Erviti^3^, C.I.E. Smith^1^, M.J.A. Wood^2^ and S. EL Andaloussi^1^

^1^Karolinska Institutet, Solna, Sweden; ^2^University of Oxford, Oxford, UK; ^3^University College London, London, UK

*Introduction*: Exosomes are small membrane vesicles that participate in cell-to-cell communication. Owing to their potential as a natural shuttle of genetic material, exosomes are studied for their role in physiological and pathological processes, as possible new diagnostic tools, and as novel therapeutic agents for gene therapy. The biogenesis of exosomes is being unraveled by recent publications. However, the biodistribution of exosomes in vivo remains incompletely understood. In vivo biodistribution studies are of utmost importance for the development of exosomal therapies. Here we demonstrate and compare two methods of tracking exosomes in vivo in real time, utilizing the IVIS imaging system. *Materials and methods*: Exosomes were isolated from different cell sources and characterised using western blot and Nanoparticle-tracking analysis. The exosomes were enriched with firefly luciferase protein or labelled with lipophilic tracers for luminescence or fluorescence imaging, respectively. Mice were given single i.v. injections of exosomes and monitored in the optical imaging system IVIS spectrum. *Results*: Luciferase-enriched exosomes produced luminescence in vitro. In vivo, exosomes labelled with a fluorescent marker could be detected for at least 48 hours. The main accumulations of exosomes were in all experiments visualised in spleen, liver and lungs and could also be detected in kidneys, blood and brain. Central nervous system-targeted exosomes increased the distribution to brain by 2-fold. Tumour-bearing mice treated with exosomes displayed accumulation in tumour tissue. *Conclusion*: These findings suggest that the exosomal biodistribution can be studied in vivo and used for various animal models. More research needs to be done to investigate the stability of fluorescent markers in vivo and to evaluate the luciferase approach to track exosomes.

**Extremely low-frequency magnetic fields significantly enhance the potency of chemotherapeutic drugs for the targeted treatment of cancer**

Jameel Inal ^1^ and D. Stratton^2^

^1^London Metropolitan University, London, UK; ^2^London Metropolitan University, Cellular and Molecular Immunology Research Centre, London, UK

*Introduction*: We showed previously that an alternating current, pulsed, extremely low-frequency electromagnetic field (ELFMF) can cause transient damage to the plasma membrane. This study had the aim of seeing whether such ELFMF could enhance the action of chemotherapeutic drugs by making them more accessible to the cancer cell. *Material and methods*: The ELFMF generator plates used to treat cells were set at 14 cm apart, and the field strength was 0.3 µT at 10Hz, 6V AC. The 100 ms duration of the AC field was controlled by a square-wave-triggered pulse. *Results*: This study shows a potential new use for ELFMFs in conjunction with chemotherapeutic drugs to enhance the effectiveness of cancer treatment and reduce chemotherapy regimens. We found that the potency of chemotherapeutic drugs was increased 10-fold when co-stimulated with ELFMFs, such that 1/10th of the normal therapeutic level of drug had the same effect as the normal dose without ELFMF stimulation. Furthermore, the ELFMFs were targeted, showing no effect on adjacent cells and had similar effects on all the cell lines studied. Cancer cells exposed to ELFMFs experience a transient loss of viability. Upon removal of ELFMF, cell viability returned to normal. During stimulation, the cells exhibited increased permeability to macromolecules via the transient membrane damage, leading to accumulation of the drugs within the cells’ cytoplasm along a concentration gradient. Once removed from the magnetic field, the macromolecules become “trapped” within the cell. *Conclusions*: The type of targeted cancer therapy proposed in this study, using ELFMF, will provide a significant change in cancer treatment as much lower levels of chemotherapeutic drug need be administered.

**Exosomes from marrow stromal cells expressing mir-146B inhibit glioma growth in rat brain**

Mark Katakowski, B. Buller, X.G. Zheng, Y. Lu, F. Jiang and M. Chopp

Henry Ford Hospital, Michigan, MI, USA

*Introduction*: miRNAs are abundant in extracellular exosomes and can be transferred by release and uptake, resulting in cross-cellular gene regulation. Bone marrow stromal cells (MSCs) release exosomes which contain functional miRNA. Therefore, we tested if MSC exosomes could be used as a delivery vehicle for anti-tumor miRNA therapy of malignant glioma. Human mir-146b is located on chromosome 10 within 10q24-26, a region lost in a majority of glioblastoma multiformes. We have demonstrated that mir-146b reduces glioma cell motility and invasion, and that epidermal growth factor receptor (EGFR) mRNA is a binding-target for miR-146b silencing. As miR-146b inhibits EGFR expression and suppresses malignancy in glioma cells, we hypothesised that MSC exosomes containing miR-146b could have an anti-tumor effect in vivo. *Material and methods*: A DNA expression plasmid was employed to express miR-146b or cel-miR-67 in primary rat MSCs. Exosomes were collected and harvested using ExoQuick-TC precipitation solution. Real-time PCR was employed to detect miRNA cel-miR-67 or miR-146b in exosomes or 9L gliosarcoma cells. Western blot was used to measure EGFR and NF-κB protein. We employed a Fischer rat model of primary brain tumour. Exosomes (50µg) or delivery vehicle was used to treat tumour-bearing rats by intratumoural injection 5 days after tumour implant. Rats were sacrificed 10 days post-implantation, and tumour volume was measured. *Results*: Treatment with exosomes from miR-146b-transfected MSCs (M146-exo) reduced 9L rat gliosarcoma cell growth and expression of EGFR protein in vitro. Intratumoural injection of M146-exo significantly reduced glioma xenograft growth in a rat model of primary brain tumour. *Conclusions*: These findings indicate that MSCs may be used to package therapeutic anti-tumour miRNA into MSC exosomes, and these exosomes can be used to deliver therapeutic miRNA into tumour.

**Physiologic oxygen concentrations alter exosome release and RNA incorporation by acute myeloid leukemia cells**

J.Y. Huan ^1^, J.R. Chevillet^2^, N.I. Hornick^1^, M. Tewari^2^ and P. Kurre^1^

^1^Oregon Health & Science University, Portland, OR, USA; ^2^Fred Hutchinson Cancer Research Center, Seattle, WA, USA

*Introduction*: A relatively lower oxygen concentration (1%*–*6%) is critical for maintaining stem cell quiescence and self-renewal while promoting leukemia progression and angiogenesis. We recently reported that acute myeloid leukemia (AML) cells release exosomes that modulate transcriptional activity, growth factor secretion and phenotypic behaviour of stromal bystander cells. Here we determine the relative impact of physiologic oxygen concentrations on the secretion of exosomes and incorporation of RNA by AML cells. *Material and methods*: HL-60 and Molm-14 leukemia cell lines, hypoxia chamber, RNA Bioanalyzer, nanoparticle tracking instrument, and qRT-PCR. *Results*: Under physiologic (1% O2) and tissue culture (21% O2) conditions, cell viability of >90% was maintained using gas-permeable culture flasks. Following culture for 48 hours, nanoparticle-tracking analysis revealed a significant increase in exosome production in HL-60 and Molm-14 cells. RNA Bioanalyzer analysis showed no difference in the integrity and quality of cellular and vesicle RNA isolated from two culture conditions. Overall RNA yield from exosome fractions was correlated with increased exosome production at low oxygen concentration, whereas cellular RNA yield was unchanged. The expression of hypoxia-inducible factor (HIF) and VEGF, a key target of HIF1a and a regulator of angiogenesis, was significantly elevated. Other transcription factors, including a critical regulator of myeloid differentiation (CEBPα) and two tumor suppressors (SHIP1 and E2F1) were down-regulated under low oxygen conditions. Importantly, levels of those transcripts were differentially represented in vesicles, suggesting that hypoxia has dual effects on both vesicle release and RNA incorporation. *Conclusions*: At physiologic oxygen concentrations, conditioned AML cells increase their production of exosomes and incorporation of affecting vesicle biogenesis in AML cells and may differentially impact bystander cells in the surrounding microenvironment.

**Extracorporeal exosome removal: a therapeutic strategy to address an evolutionary survival mechanism shared by cancer and infectious viral pathogens**

James Joyce, A.M. Marleau, R.P. Duffin and R.H. Tullis

Aethlon Medical Inc., San Diego, CA, USA

*Introduction*: Exosome secretion is a notable feature of malignancy owing to the diverse roles of these nanovesicles in tumour-mediated immune suppression, angiogenesis, metastasis, and resistance to therapeutic agents. As cancer exosomes represent critical targets that have not yet been addressed by drug therapies, we have devised a novel approach involving systemic haemofiltration of exosomes using a medical device strategy, Aethlon's Hemopurifier. *Methods and results*: The Hemopurifier consists of the lectin Galanthus nivalis agglutinin (GNA) immobilised in the outer-capillary space of a plasma filtration cartridge that is compatible with standard dialysis units. GNA-based capture is mediated by high mannose glycoproteins abundant on the surfaces of cancer exosomes, allowing exosomes to be eliminated from the entire circulatory system. Significantly, since GNA exhibits specificity for a spectrum of disease-promoting particles displaying high mannose signatures, this device strategy can be deployed against a breadth of targets. Indeed, preliminary studies of Hemopurifier therapy in hepatitis C virus-infected patients has documented the capture of up 300 billion virions, having similar sizes and surface glycosylation signatures as exosomes, during a single 6-hour treatment. By reducing the systemic load of disease-mediating particles, patient responses to standard of care therapies can be improved and accelerated by Hemopurifier therapy. *Conclusions*: Owing to the presence of common molecular signatures in cancer and viral infections, the Hemopurifier holds promise for addressing systemic immune suppressive factors in various diseases, including the production of malignant exosomes that is a hallmark of many cancers.

**Construction of polyvalent RNA nanoparticles for escorting siRNA, ribozyme and miRNA into cells and exosomes**

F. Haque, D. Shu, Y. Shu, P. Rychahou, B.M. Evers and P. Guo

University of Kentucky, Lexington, KY, USA

*Introduction*: One of the advantages of RNA nanotechnology is the feasibility to construct RNA nanoparticles carrying multiple therapeutics with defined structure and stoichiometry. Herein, we report the construction of thermodynamically and chemically stable X-shaped and tri-star shaped RNA nanoparticles to carry 1, 2, 3 or 4 siRNA, miRNA, RNA aptamer or ribozymes using re-engineered RNA fragments derived from the pRNA of bacteriophage phi29 DNA packaging motor. *Results and conclusions*: The nanoparticles self-assemble very efficiently in the absence of divalent salts, resistant to denaturation by 8 M urea, and does not dissociate at ultra-low concentrations. The delta G of the nanoparticles is extremely low, and the slope of the temperature melting curve is close to 900. We proved that each arm of the helices in the X-motif or tripods can harbour 1 siRNA, ribozyme or aptamer without affecting the folding of the central X or 3WJ core, and each daughter RNA molecule within the nanoparticle folds into respective authentic structure and retains their biological and structural function independently. Gene silencing effects were progressively enhanced as the number of the siRNA in each pRNA-X nanoparticles gradually increased from 1 to 2, 3 and 4. More importantly, systemic injection of the ligand-containing nanoparticles into the tail-vein of mice revealed that the RNA nanoparticles remained intact without showing any signs of dissociation or degradation; and strongly bound to cancers without entering liver, lung or any other organs or tissues. Pharmacokinetic analysis revealed that its half-life was extended 100-fold compared to the siRNA counterpart. Particles tested in vivo revealed that they did not induce cytokines, interferon-I, antibody and toxicity while retaining favourable pharmacokinetics profiles.

**Enhanced immunostimulatory activity of exosome–DNA nanovesicles**

Tamer Kahraman, Gozde Gucluler and Ihsan Gursel

Biotherapeutic ODN Research Lab., Department of Molecular Biology and Genetics, Bilkent University, Ankara, 06800, Turkey

Exosomes are naturally occurring, membranous nanovesicles known to function as intercellular communication vectors. To exploit exosomes as a nucleic acid delivery system, we externally loaded them with a TLR9 ligand and explored their immunostimulatory properties. Exosomes isolated from RAW264.7 cell line supernatant by differential centrifugation and ultracentrifugation steps. They were loaded with two types of CpG oligos via a patented technique. These exosomes were then labelled with a lipophilic fluorescent dye and incubated with immune cells. Following stimulation, cells were analysed by flow cytometer and confocal microscopy for exosome binding and uptake while supernatants were used for pro-inflammatory cytokine secretion by ELISA. Labelled exosomes containing CpG ODNs were internalised by cells much higher level than their free ODN counterparts as revealed by FACS and confocal studies. Co-localisation signals for FITC-exosome and Cy3-DNA were observed during assessing internalisation. Exosome-loaded CpG ODNs gave higher IL6, IL12 and IFNg secretion than free CpG ODN. Exosomes were protecting their cargo from nuclease attack, and cellular entry was shown to be dependent on scavenger receptor. In a tumour model in C57Black6 mice, OVA-specific priming led to total clearance of EG7-derived thymoma tumour even after 6 months of immunisation. Here, a novel exosome-based nucleic acid delivery system was developed which offers an alternative approach to synthetic platforms. Problems associated either with viral or non-viral delivery systems, such as persisting inflammation leading to undesirable tissue destruction or generation of antibodies against injected carrier components could be alleviated with the present technique, since patient's own exosomes from plasma could be utilised for immunotherapy. This technology may help to develop individualised immunotherapeutic delivery approaches for labile and valuable biomolecules such as RNA, DNA or even peptides against infectious diseases or cancer therapy.

**Fat-graft-derived exosomes: a promising tool for skin repair and remodeling**

Marco Aurelio Pellon

Burns And Trauma Unit - Clinica Sao Vicente, Rio De Janeiro, Brazil

*Introduction*: Many authors describe an unexpected improvement in the quality of the skin overlying an area of the fat grafting. This beneficial effect has been used to correct and improve scars, burns, radiation injuries and pigmentation. The author describes a cross-talk between the subcutaneous adipose tissue and the skin, controlling hair growth and epidermis regeneration. After a skin trauma, damaged cells and tissues release a great number of molecules. Many of these molecules are released by activated cells into exosomes carrying its RNA which will be transferred to neighboring cells, affecting the recipient cell's protein production and even assisting in DNA repair. The exodus of exosomes is facilitated by the low pH condition of the inflammatory tissue, which increases the permeability of the cell membrane. *Material and Methods*: A clinical study was conducted in patients with burns and deep wounds, which showed all stages of skin regeneration and morphological changes during the healing process. Were also documented the changes after fat grafting upon and below the injured tissue in acute and chronic phases. *Results*: The author observed the significant changes in the tissue's morphology below the burns and deep wounds due to genetic communication between the damaged and the survivor cells, even in the absence of vascular communication. *Conclusions*: When a damage of the skin's structures occurs, that information gets to the underlying cells via cell-cell interactions with RNA transfer, carrying gene-regulatory function from the damaged to the target cells resulting in a change of adipokines production and expression. In a reverse way, where the aggression occurs in subcutaneous adipose tissue, the exosomes released by injured and ischemic adipocytes transfer their genetic information to neighboring cells, reaching the overlying skin, playing a pivotal role in stem cell plasticity and cellular regeneration, restoring skin health.

## Scientific Program 2013 ISEV meeting Friday 19th April

**Plenary Oral Session 17 (Imperial Ballroom)**

**Chair: *F. Hochberg and M. Wauben*  8:30-9:30**

**Designing Systems for Drug Delivery, A Template for Exosome Studies**

Robert Langer

MIT, Cambridge, USA

Robert Samuel Langer, Jr. is an American engineer and the David H. Koch Institute Professor at the Massachusetts Institute of Technology. Langer is regarded for his contributions to medicine and biotechnology, especially drug delivery, including transdermal delivery systems.

**Plenary Oral session 18 (Imperial Ballroom) 9:30-10:00**

**9:30-9:45**

**Biogenesis of tumour-derived microvesicles**

Crislyn D'Souza-Schorey^1^, A.E. Sedgwick^2^, J. Clancy^2^ and V. Chari^2^

^1^Department of Biological Sciences, USA; ^2^University of Notre Dame, USA

Increased MAPK signalling, small GTPase activation, cytoskeletal rearrangements and the directed targeting of proteases to sites of extracellular matrix (ECM) degradation, all accompany the process of tumour cell invasion. We have shown that nucleotide cycling on ARF6, a Ras-related small GTP-binding protein, regulates the release of protease-loaded, plasma-membrane-derived microvesicles from tumour cells into the microenvironment to promote ECM degradation. These “invasive” microvesicles are distinct from exosomes, can be isolated from peripheral body fluids and appear to be linked to amoeboid motility of tumour cells. They are also distinct from invadopodia, which are actin-rich protrusions at the adherent face of the cell and also mediate cell invasion. While pre-invasive tumour cells shed microvesicles, the amount of shed microvesicles increases with invasiveness. We suggest that the release of protease-loaded microvesicles potentially serves as a mechanism to bring about matrix degradation at distal locations, creating paths of “least resistance” as tumour cells traverse distances and invade through surrounding tissue. Current efforts are directed at understanding how extracellular cues and the specific targeting of distinct cargo enables the biogenesis of microvesicles.

**Tumour-derived exosomes promote pre-metastatic niche formation and organotropism**

Hector Peinado, Ayuko Nitadori Hoshino, Tang-Long Shen, Masa Aleckovic, Yibin Kang, Jacqueline Bromberg and David Lyden

Cornell University, Ithaca, NY, USA

Metastasis is the most deadly aspect of cancer due to a lack of appropriate therapies. Tumour-secreted factors have been recently recognized to be one of the main culprits for metastatic progression. Tumour-secreted factors such as VEGF-A, PlGF, TGF, TNF-a and LOX have been shown to play active roles in the recruitment of bone marrow (BM)-derived cells to the primary tumour microenvironment and pre-metastatic niches. We have found that tumour-derived exosomes are abundantly secreted into the circulation in highly metastatic murine models and in patients with stage IV metastatic disease. Tumour-derived exosomes induce vascular leakiness, and hypoxic and pro-inflammatory changes at pre-metastatic sites. Moreover, tumour-derived exosomes preferentially fuse and “educate” BM-derived progenitor cells to a pro-vasculogenic phenotype characterised by upregulation of Tie-2, VEGF-A, VEGFR2, TSP1 and ADAM10. We found that B16-F10 melanoma-derived exosomes help establish pre-metastatic niches, including the recruitment of “educated” BM cells, in specific organs destined to be involved in future metastasis, whereas LLC lung cancer-derived exosomes predominant in the lung as the main organ of metastasis. These results suggest that tumour-derived exosomes may have a role in metastatic organotropism, whereby cancer metastasises to specific organs, as proposed by Stephen Paget's “seed and soil” hypothesis more than a 100 years ago. We believe that the identification of exosomes proteins, with their prognostic and therapeutic potential, may also partially explain the specific receptor–ligand binding of exosomes to a pre-metastatic niche defining an organotropic site for future metastasis.

**Coffee and Poster Sessions V-VI 10:00-13:30**

**ISEV General Assembly 12:00-13:30**

**Parallel Oral Sessions 21-23 13:30-15:15**

**Oral Session 21 (Imperial Ballroom): Therapy: Nanoparticles April 19**

**Chair: *F. Hochberg and R. Schiffelers* 13:30-15:15**

**Extracellular vesicles versus synthetic drug delivery systems advantages and drawbacks**

Ray Schiffelers

University Medical Center Utrecht, The Netherlands

I would like to contrast, in an introductory talk, my 20 year experience with synthetic drug delivery systems with our (and others’) results with extracellular vesicles. What are the unique selling points of extracellular vesicles? But also, what are their (unique) drawbacks? Starting from making/harvesting them and loading them with the desired compounds, to shelf-life and colloidal stability, to tissue targeting and target cell interaction and ultimately delivery of cargo and read-out of therapeutic effects.

**Exosomes for delivery of small RNAs to tumours**

P. Vader^1^, J.Z. Nordin^2^, O.P.B. Wiklander^2^, Y. Lee^1^, T.C. Roberts^1^, Y. Seow^3^, El S. Andaloussi^2^ and M.J.A. Wood^1^

^1^University of Oxford, UK; ^2^Karolinska Institutet, Sweden; ^3^Agency for Science, Technology and Research, Singapore

*Introduction*: Exosomes are important mediators of cell-to-cell communication by transporting a variety of cargos, including miRNAs, between cells. In this study we evaluated whether exosomes can be engineered to deliver small RNAs to tumours. *Material and methods*: Exosomes were isolated from HEK293T cells and characterised using western blot and Nanoparticle-tracking analysis. Small RNA-carrying exosomes were prepared by overexpression of shLuc or let-7b in the exosome donor cells. Uptake and knockdown efficiency was evaluated in B16F10 melanoma tumour cells in vitro. Biodistribution of DiR-labelled exosomes after intravenous administration was determined in mice bearing a subcutaneous B16F10 tumour. *Results*: Overexpression of shLuc or let-7b in HEK293T cells led to increased secretion via exosomes. Exosomes were able to deliver these small RNAs to tumour cells in vitro. In vivo biodistribution in tumour-bearing mice showed accumulation of exosomes in liver, spleen and tumour tissue. *Conclusions*: These data suggest that exosomes can be used to deliver therapeutic small RNAs for the treatment of cancer.

**Heterogeneity in packaging and targeting of extracellular microRNAs in breast cancer**

L. Palma, M. Havens, M. Hastings and D. Duelli

Rosalind Franklin University, USA

*Introduction*: Cells regulate each other remotely in many ways. An emerging mechanism is the exchange of microRNA (miRNA) packaged into membrane-coated vesicles. We previously reported that malignant transformation changes the export of extracellular miRNAs (ex-miRs) by affecting whether a particular miRNA species is released selectively or retained by the cell. We here describe the basis of selective ex-miR release and transfer to target cells. *Material and Methods*: cell culture, milk, mice; differential buoyant velocity centrifugation, proteomics and transcriptomics; miRNA FISH and IF, qRT-PCR; antibody production, IP, transfer assays, immunoblotting, and EM. *Results*: We show that ex-miRs are packaged mutually exclusively in different carriers released from breast cancer cells. Selectively released miR-451 associates with exosomes and miR-1246 with nucleosomes, and neutrally released miR-16 associates with unconventional “L-” exosomes. In contrast, normal cells release these ex-miRs in a single type of vesicle. We demonstrate that uptake of ex-miRs and its consequences are cell-type and carrier-type specific. For example, T-cells accumulate ex-miR-16 and ex-miR-451, while megakaryocytes internalise miR-16 but not miR-451. The received miR-16 represses BCL2 in T-cells, triggering apoptosis, but causes cell cycle arrest in megakaryocytes. Finally, monocytes do not acquire miR-16 or miR-451 from the extracellular environment, and the cells differentiate. *Conclusions*: Malignant transformation induces de novo extracellular vesicles, into which ex-miRs are assorted mutually exclusively. This separation determines in part the cell-type-specific delivery of extravesicular cargo and can explain how ex-miRs can simultaneously activate cancer-promoting cells and block anti-cancer cells. We propose a model wherein ex-miR-cell signalling occurs similar to virus–cell interactions, including the need for ligand–receptor interactions, and subsequent cellular trafficking.

**Generation of exosome-mimetic nanovesicles for cytosolic RNAs and protein delivery**

D.Y. Jeong^1^, W.J. Jo^1^, J.H. Kim^1^, S.C. Jang^2^, Y.S. Gho^2^ and J.S. Park^1^

^1^School of Interdisciplinary Biosicence and Bioengineering, POSTECH, Republic of Korea; ^2^Life science and Division of Molecular and Life Sciences, POSTECH, Republic of Korea

*Introduction*: cell-secreted exosomes or microvesicles have recently discovered to contain proteins as well as messenger RNAs and micro RNAs. Those cell-secreted natural exosomes are nanoscale diameter compartments of endocytic origin with an enclosed lipid bilayer membrane, having ability to deliver biological components from the origin cells. In this study, we mimicked cell-secreted exosomes by utilising living cells for cytosolic RNA and proteins delivery. For this purpose, we fabricated the nanovesicles by extruding living cells through constrictive microchannels. *Material and methods*: The microfluidic channel made with PDMS softlithograpy and was composed of parallel arranged several tens of cell-breaking channels. The murine ES cell line was used as a source for the nanovesicles. This cell was extruded through the fabricated microchannels by using syringe pump. To check the surface effect during the generation of cell-derived artificial vesicles, hydrophilic and hydrophobic surface of microchannels were prepared. *Results*: Nanoparticles were observed to form only from the hydrophilic microchannel, suggesting the surface property in terms of hydrophilicity and hydrophobicity is important for nanovesicles. The nanoparticles were found to have a bilayer membrane structure by TEM, and the size of them was determined to be 60–120 nm by using DLS. In western blot analysis, ICAM-1, Oct 3/4 and actin were confirmed. In RT-PCR, Oct 3/4 and actin were also found in nanovesicles. For cellular uptake, according to treatment time of nanovesicles, their position in the cell was changed membrane to inside of the cell. *Conclusion*: The nanoartificial vesicles containing RNAs, membrane and cytosolic proteins of origin cells were generated by a simply extruding cells through a microchannel. These nanovesicles can be used in extracellular vesicles research fields such as uptake mechanism and cellular material delivery pathway.

**Fabrication of 14 different RNA nanoparticles for specific tumour targeting without accumulation in normal organs**

Y. Shu, D. Shu, F. Haque and P. Guo

University of Kentucky, USA

*Introduction*: Due to structure flexibility, RNase sensitivity and serum instability, construction of RNA nanoparticles with concrete shapes for in vivo application remains challenging. Here we report the construction of 14 RNA nanoparticles with solid shapes for targeting to cancers specifically. *Results and conclusions*: These RNA nanoparticles were resistant to RNase degradation, stable in serum for more than 36 hours and stable in vivo after systemic injection. Applying RNA nanotechnology and exemplifying with these 14 RNA nanoparticles, we establish the technology and develop a “toolkits” utilising a variety of principles to construct RNA architectures with diverse shapes and angles. RNA loops, cores, junction motifs and palindrome sequences from phi29 motor pRNA were gathered in a “toolkit” to demonstrate their utility for fabrication of dimers, twins, trimers, triplets, tetramers, quadruplets, pentamers, hexamers, heptamers and other higher order oligomers, as well as branched diverse architectures via hand-in-hand, foot-to-foot and arm-on-arm interactions. These novel RNA nanostructures harbour resourceful functionalities for numerous applications in nanotechnology and medicine. It was found that all incorporated functional modules, such as siRNA, ribozymes, aptamers and other functionalities folded correctly and functioned independently within the nanoparticles. Incorporation of all functionalities was achieved prior, but not subsequent to, the assembly of the RNA nanoparticles, thus ensuring the production of homogeneous therapeutic nanoparticles. Most importantly, upon systemic injection, these RNA nanoparticles targeted cancer exclusively in vivo without trapping in normal organs and tissues. This finding opens a new territory for cancer targeting and treatment. The versatility demonstrated by one biological RNA molecule implies immense potential concealed within the RNA nanotechnology field.

**Electroporation-induced aggregation overestimates the efficacy of siRNA loading into extracellular vesicles**

S. Stremersch, K. Braeckmans, S.C. De Smedt and K. Raemdonck

Ghent Research Group on Nanomedicines, Lab of General Biochemistry and Physical Pharmacy, Ghent Univ, Belgium

*Introduction*: At present there is a growing interest in exploiting cell-derived membrane vesicles (MVs) as nucleic acid [e.g. small interfering RNA (siRNA)] delivery tools. However, clinical application of MVs requires efficient methods to load them with exogenous therapeutic siRNA. *Material and methods*: MVs were isolated from MCF-7 and HEK293T cells and electroporated with Cy5-labelled siRNA in an Optiprep™ electroporation buffer as previously reported [1]. The encapsulation efficiency was quantified by fluorescence fluctuation spectroscopy (FFS). Nanoparticle-tracking analysis (NTA) and confocal microscopy were applied to analyse aggregate formation. *Results*: FFS revealed a siRNA loading in MVs of ~20% after electroporation, in line with data reported previously [1,2]. Interestingly, electroporating siRNA in the absence of MVs yielded analogous results. NTA verified the emergence of large aggregates, likely due to the electroporation-induced release of aluminium cations from the cuvette electrodes. Confocal microscopy revealed a distinct co-precipitation of siRNA in the aluminum aggregates, explaining the FFS results. Reducing aggregate formation by adding EDTA markedly decreased the aspecific siRNA precipitation. Electroporation of siRNA in an acidic citrate buffer entailed no detectable aggregation since citrate acts as an aluminum chelator and the acidic pH prevents the formation of insoluble aluminium oxyhydroxides [3]. When quantifying the siRNA loading under these conditions, no significant encapsulation of siRNA was detected neither in the absence nor in the presence of MVs. *Conclusions*: Altogether, our data provide evidence that electroporation-induced precipitation may strongly bias the assumed encapsulation of siRNA into MVs. New strategies to improve siRNA loading in MVs are of key importance for future clinical application.

References

1. Alvarez-Erviti et al., Nat. Biotechnol., 2011.

2. El-Andaloussi et al., Nat. Protoc., 2012.

3. Dabbs et al., Langmuir, 2005.

**Tissue-specific gene silencing monitored in circulating RNA**

Alfica Sehgal^1^, Q. Chen^1^, D. Gibbing^2^, J. Harrop^1^, R. Falzone^1^, J. Gollob^1^, D.W. Sah^1^ and D. Bumcrot^3^

^1^Alnylam Pharmaceuticals, USA; ^2^University of Ottawa, Canada; ^3^Koch Institute for Integrative Cancer Research at MIT, USA

*Introduction*: Target gene modulation is the primary objective for RNA antagonist strategies, including small interfering RNA (siRNA), microRNA (miRNA) therapeutics and antisense oligonucleotide therapeutics. In clinical applications, monitoring tissue-specific target mRNA modulation requires tissue procurement from patients (a difficult and invasive process which highly limits its utility in practice). Here we describe a non-invasive method for monitoring gene modulation using isolated extracellular RNA. *Methods*: Circulating extracellular RNA was isolated from serum or cerebrospinal fluid using differential centrifugation. It was processed to measure levels of circulating mRNA after systemic or tissue-specific delivery of siRNA against different genes. The isolated RNA was also utilised for the detection of RISC-mediated cleavage products as a proof of an RNAi mechanism in serum. Results were consistent across multiple species including rats, monkeys and human subjects. *Results and conclusions*: Here we have demonstrated that RNAi-mediated target gene silencing in the liver (by systemic administration of siRNA) results in quantitative reductions in serum mRNA levels which closely track the degree and kinetics of tissue mRNA silencing. We have also used circulating RNA to demonstrate that RNA reduction in liver was a result of RNAi, RISC-mediated process. This was true in rats, monkeys as well as human subjects enrolled in the ALN-TTR02 clinical trial. Furthermore, administration of an anti-miRNA oligonucleotide directed against a liver-specific miRNA was found to result in decreased levels of the miRNA in circulation. As another application of this technique, we detected exogenous mRNA of genes expressed using adenovirus in liver in the circulating mRNA. Finally, this method was extended to non-liver tissues; silencing of a brain-expressed mRNA was monitored and quantified in cerebrospinal fluid following intraparenchymal CNS infusion of a specificsiRNA. In conclusion, we have developed a non-invasive method for monitoring the modulation effects of oligonucleotide therapies utilising easily accessible blood as a starting point. We showed that these findings can be extended to patients with liver cancer, both for detection of liver and tumour-expressed genes. This technique will greatly aid the clinical development of oligonucleotide-based therapeutics by serving as an easily accessible biomarker of drug activity.

**Oral Session 22 (Plaza Ballroom): Biogenesis and Targeting April 19**

**Chair: *C. Théry and M. Zöller* 13:30-15:15**

**Role of heparan sulphate proteoglycan and caveolin-1 in membrane raft-mediated endocytosis of cancer-cell-derived extracellular vesicles**

Helena Christianson^1^, Katrin J. Svensson^1^, Toin H. van Kuppevelt^2^, Jin-ping Li^3^ and Mattias Belting^1^

^1^Lund University, Sweden; ^2^Nijmegen Centre for Molecular Life Sciences, The Netherlands; ^3^Uppsala University, Sweden

*Introduction*: Multiple biological activities have been described for extracellular vesicles (EVs) in various physiological and pathophysiological contexts. However, the mechanisms of EV uptake by recipient cells, and how this pathway may be targeted remain largely unknown. Here, we provide evidence that heparan sulphate proteoglycans (HSPGs) function as internalising receptors of cancer cell-derived EVs with exosome-like characteristics. *Results*: Internalised exosomes co-localised with cell-surface HSPGs in recipient cells, and exosome uptake was specifically inhibited by exogenous HS, whereas closely related chondroitin sulphate had no effect. Using several cell mutants, we provide genetic evidence of a receptor function of HSPG in exosome uptake, which was dependent on intact HS 2-O-sulphation as well as N-sulphation. We further show that enzymatic depletion of cell-surface HSPG, or pharmacological inhibition of endogenous PG biosynthesis by xylosides significantly attenuates exosome uptake. On a functional level, exosome-induced ERK1/2 signalling activation was attenuated in PG-deficient mutant cells as well as by xyloside treatment of wild-type cells. Importantly, exosome-mediated stimulation of cancer cell migration was significantly reduced in PG-deficient cells, and by treatment with heparin. In agreement with a previously described pathway for HSPG-mediated endocytosis, internalised exosomes were shown to co-localize with the lipid raft marker cholera toxin B, and exosome uptake was reduced in response to membrane cholesterol depletion using methyl-β-cyclodextrin. Interestingly, the lipid raft associated protein caveolin-1 (CAV1) negatively regulated the uptake of exosomes. We show that exosome uptake appears dependent on unperturbed ERK1/2-HSP27 signalling, which is negatively influenced by CAV1 during internalization of exosomes. *Conclusion*: Our data advance the general understanding of exosome-dependent transfer of molecular information and offer potential str.

**Engineering of photoactive exosomes for cancer therapy**

J.S. Lee, J.Y. Kim and J.H. Park

KAIST, Republic of Korea

*Introduction*: Exosomes have been extensively investigated in cancer because they are believed to play an important role in the development of cancer, where their delivery of biological molecules seems to contribute to angiogenesis and metastasis. However, there has been little effort to arm tumour exosomes with functional molecules for effective cancer therapy. Here, we demonstrate that therapeutic exosomes can be produced from tumour cells treated with membrane fusogenic liposomes (MFLs) loaded with therapeutic agents and be further delivered to adjacent tumour cells to amplify cancer therapy. *Material and methods*: Membrane fusogenisity of liposomes was optimised on tumour cells by adjusting their lipid compositions. Photoactive exosomes were collected 48 h after treating tumour cells with MFL loaded with Dil (dye) or ZnPc (photosensitiser). For photodynamic therapy (PDT), tumour cells were cultured both on membrane filter and on lower chamber of the transwell. At 30 h after treating the cells on the filter with ZnPc-loaded MFL, all of the cells in the transwell were irradiated with a 660-nm laser source for 5 min, and their viability was tested with calcein AM assay. *Results*: MFL were able to deliver the loaded hydrophobic Dil or ZnPc into the membrane of tumour cells. Exosomes secreted from the cells also contained significant amount of the hydrophobic agents in their membrane. In the PDT experiment, exosomes produced from tumour cells on the transwell filter pre-treated with ZnPC-loaded MFL were found to effectively carry the ZnPc to tumour cells on the lower chamber. Laser irradiation led to significant photodynamic destruction of the cells both on the filter and on the lower chamber. *Conclusions*: In this study, photoactive exosomes were produced from tumour cells pre-treated with photosensitiser-loaded MFL and were further used for amplified photodynamic therapy. This exosome-based drug delivery has great potential to improve the treatment of cancer.

**Mechanisms that control the sorting of miRNAs into extracellular vesicles**

Carolina Villarroya-Beltri, C. Gutierrez-Vazquez, F. Sanchez-Cabo, D. Perez-Hernandez, J. Vazquez, M. Mittelbrunn and F. Sanchez-Madrid

CNIC, Spain

Several reports indicate that RNAs are selectively incorporated into extracellular vesicles (EVs); however, little is known about the mechanisms. We have identified short RNA sequences over-represented in miRNAs enriched in EVs. We have also identified proteins that specifically bind these sequences and control the loading of miRNAs into EVs. These findings provide potential tools for the packaging of selected regulatory RNAs into EVs and their use in biomedical applications.

**Palmitoylation drives sorting of EBV oncoprotein LMP1 into exosomes, to restrain constitutive NFkB activation**

Frederik Verweij, M.A.J. Eijndhoven, C. Jimenez, J.M. Middeldorp and D.M. Pegtel

VUmc Cancer Center Amsterdam, The Netherlands

Developing B cells require a CD40-mediated signal from T cells (T cell help) to survive in the germinal centre reaction. Epstein-Barr virus infects naive B cells and through expression of a specific latency program drives the newly infected cells through a GC reaction into memory B cells. This process relies heavily on timely expression of the virus-encoded CD40 mimic latent membrane protein 1 (LMP1), thereby playing a key role in establishing viral persistence. Unlike CD40, LMP1 activates NFkB constitutively and poses a risk factor for EBV-associated lymphomas. Despite this knowledge, over 90% of the world population is persistently infected with EBV, seemingly without causing much harm. How EBV-infected cells expressing oncogenic LMP1 are protected from malignant conversion is poorly understood. We proposed that a major proportion of LMP1 is restrained from signalling by incorporation into multivesicular bodies, resulting in secretion of the protein via exosomes. We discovered that a single mutation in the active palmitoylation site of LMP1 results in increased intracellular aggregation and elevated recruitment of a key signalling mediator TRAF2. This co-aggregation enhanced NFkB overstimulation and delayed secretion of the protein via exosomes. Moreover, the palmitoylation mutant shows a 50% lower transformation capacity compared to wild type. This is in line with recent studies indicating that the correct localisation of a protein in a cell is crucial for its signalling. Signalling receptors such as the Met receptor or Src kinase need to be internalised and localise to endosomal membranes before they can signal, although they are already activated at the cell surface. We are currently investigating whether palmitoylation is a general mechanism driving sorting of proteins into exosomes. This would be an interesting similarity with virion formation, since certain viral proteins (e.g. influenza HA) depend on palmitoylation to be incorporated in the budding virion.

**Exosomes derived from Schwann cell are internalised by neurons and promote axonal regeneration**

M.A. Lopez-Verrilli, F. Picou and F.A. Court

Millennium Nucleus in Regenerative Biology (MINREB), Catholic University of Chile, Chile

*Introduction*: Schwann cells (SCs) are the glial component of the peripheral nervous system (PNS). After nerve injury, SCs dedifferentiate to a progenitor-like state and guide axons to their target tissues. Contact and soluble factors participate in the crosstalk between SCs and axons during axonal regeneration. We have previously shown that in the PNS, ribosomes are transferred from SCs to axons, but the transfer mechanism was not investigated. We propose that exosomes mediates macromolecular transfer between SCs and neurons; therefore, we investigated the functional relevance of this process during axonal growth and regeneration. *Material and methods*: SC primary cultures were obtained from rat sciatic nerves. Exosomes were purified from SC-conditioning medium by ultracentrifugation and characterised by morphological and biochemical analysis. Exosome internalisation in dorsal root ganglia (DRG) sensory neurons was observed using vital dyes, immunofluorescence, confocal microscopy and electron microscopy. Axonal growth and regeneration assays were performed in embryonic and adult DRG in vitro and in rat crushed-sciatic nerves in vivo. *Results*: Here we show that SCs secrete exosomes which are internalised by axons. SC-derived exosomes markedly increase neurite growth and axonal regeneration in vitro and enhance regeneration after sciatic nerve injury in vivo. In addition, SC-derived exosomes activates in neurons previously identified genes crucial for the expression of a regeneration program. *Conclusions*: We demonstrate for the first time that SCs secrete exosomes and that these exosomes are internalised by axons. SC-derived exosomes increase axonal growth and axonal regeneration after injury in vitro and promote axonal regeneration in peripheral nerves in vivo. Our results support the role of SC exosomes to maintain axonal integrity and to improve nerve repair after peripheral injury.

**Active Wnt proteins are secreted on exosomes**

J.C. Gross^1^, V. Chaudhary^1^, K. Bartscherer^2^ and M. Boutros^1^

^1^German Cancer Research Center (DKFZ), Germany; ^2^Max Planck Institute for Molecular Biomedicine, Germany

*Introduction*: Wnt signalling is important in developmental processes and highly conserved from fly to human. As morphogens, hydrophobic Wnt proteins are acting over a distance to induce patterning and cell differentiation decisions. Recent studies have identified several factors that are required for the secretion of Wnt proteins; however, how Wnt proteins travel in the extracellular space remains a largely unresolved question. We investigated the role of exosomes as a mean of extracellular spreading of Wnt proteins. *Results*: We show that Wnt proteins are secreted on exosomes both during *Drosophila* development and in human cells. Purified exosomes carry active Wnt proteins on their surface and can induce Wnt signalling activity in target cells. Furthermore, we investigate the sorting of Wnt proteins onto exosomes: the Wnt cargo receptor Evi/WIs shuttles Wnt into MVBs and is also present on exosomes, which is impaired by interfering with MVB sorting and maturation. By an in vivo RNAi screen, we identify the R-SNARE protein Ykt6 as a factor required for Wnt secretion in *Drosophila*. Further studies in human cells demonstrate its evolutionarily conserved function. Ykt6 mediates the transport of Wnt proteins together with their cargo receptor Evi/WIs through endosomal compartments onto exosomes. *Conclusions*: We demonstrate by biochemical and genetic approaches that a portion of functional Wnt proteins is secreted on exosomes. Exosomal Wnt secretion is an alternative route for secretion of active Wnt proteins.

**Genetic modulation and imaging of biogenesis, secretion and fusion reveals roles of *Drosophila* seminal fluid exosomes in sperm signalling and reprogramming female behaviour after mating**

Laura Corrigan, S.J. Fan, C. Gandy, A. Leiblich, R. Patel, S. Redhai, J. Morris, F. Hamdy and C. Wilson

University of Oxford, UK

*Introduction*: Our ability to study the mechanisms of exosome biogenesis and secretion is impeded by the small size of the subcellular compartments in which these events occur. Mammalian seminal fluid has been shown to contain exosomes, whose physiological role after mating is yet to be demonstrated in vivo. Secretions of the accessory gland (AG) epithelium contribute to *Drosophila* seminal fluid. We have previously shown that the secretions of secondary cells (SC) in the AG increase the fecundity and reproductive competitiveness of males. *Method*: Using genetic tools in flies to activate inducible gene expression specifically in SCs, exosomes secreted by SCs were fluorescently labelled with CD63-GFP. Reproductive tissues were imaged by confocal and electron microscopy. *Results*: The intracellular secretory and endocytic compartments of SCs are very large (2–10µm in diameter), allowing us to visualise endocytic trafficking, intraluminal vesicle formation and exosome secretion in real time and in fixed tissue. We find that only SCs within the AG secrete CD63-GFP-labelled exosomes that are formed in lysotracker-positive multivesicular bodies, which dynamically fuse with other secretory compartments. SC exosome secretion is strongly reduced by knockdown of ALiX. We show that these exosomes are transferred to females during mating where, like isolated exosomes from human prostate, they fuse with sperm, as well as specific epithelial cells of the female reproductive tract. Blocking SC exosome production suppresses post-mating effects on female behaviour, suggesting that these exosomes transfer important signals from males to females. *Conclusions*: Secondary cells of the *Drosophila* AG are an attractive new in vivo model cell type to study exosome biogenesis and secretion in a genetic system. Our data demonstrate an important physiological role for exosomes in reproduction and reveal the detailed dynamics of their formation and secretion for the first time in vivo.

**Oral Session 23 (Georgian): Multiomics April 19**

**Chair: *Y.S. Gho and S. Mathivanan* 13:30-15:15**

**The genetics of gene expression in exosomes**

Stephen Montgomery, K.S. Smith, V. Anaya and E. Mitsunaga

Stanford University, USA

*Introduction*: Genetic studies of gene expression using microarrays and RNA sequencing have elucidated considerable tissue-specificity of regulatory variation. These studies, however, have been limited to considering individual cells and tissues in isolation and therefore are restricted in their ability to assess genetic effects driving cellular interactions. Exosomes are important extracellular vesicles packaging miRNA facilitating intercellular communication. To understand how genetic variation influences exosome-mediated intercellular communication, we have sought to characterise the genetic diversity of the exosome transcriptome in a 3 generation, 17 member family. *Materials and methods*: Using whole genome data, we have further sequenced the total cell RNA and exosomal miRNA from lymphoblastoid cell lines of a 17 member family to characterise the influence of genetics on exosome miRNA gene expression. Our method of exosome isolation and sequencing involved untracentrifugation and filtration through a 200 nm filter. Exosomal pellets were treated with Trizol, and then we proceeded with organic phase isolation through centrifugation and subsequent ethanol precipitation of RNA species. Isolated RNA was quality checked and quantified using a Bioanalyzer 2100. Small RNA species were converted to cDNA using the TruSeq Small RNA procedure. Indexed samples were sequenced on the MiSeq Personal Sequencer (Illumina). Exosome purification was then verified using electron microscopy. *Results*: We report the exosomal miRNA species for each individual and further dissect the relative contributions to exosomal miRNA content from paternal and maternal alleles. We further assess the impact of *cis*-linked regulatory variation on exosomal miRNA within the family. *Conclusions*: This work provides new avenues for assessing the genes involved in exosome biogenesis and packaging and a detailed look at the influence of genetics on exosome miRNA content.

**Quantitative proteomic analysis of cancer exosomes using a novel-modified aptamer-based array (SOMAscan™)**

Aled Clayton^1^, J.P. Webber^1^, B. Lollo^2^, T. Bartlet^2^ and E. Katilius^2^

^1^Cardiff University, Institute of Cancer & Genetics, UK; ^2^SomaLogic Inc, 2945 Wilderness Place, Boulder, CO 80301, USA

*Introduction*: Mass spectrometric proteomics have been conducted on extracellular vesicles, of diverse origins. Whilst informative, limitations of such approaches include a lack of quantitation, and low sample throughput. Multiplex protein-array methods potentially overcome such issues; however, most current platforms are based on antibodies and are limited to less than 100 in their repertoire of protein coverage. *Materials and methods*: We have used a novel affinity based proteomic technology to examine the protein signature of extracellular cell-derived vesicles called exosomes. The technology allows the simultaneous quantitative measurement of over 1,000 proteins, through a new class of protein binding reagents called SOMAmer™ (Slow Off-rate Modified Aptamer). Exosomes were highly purified from the Du145 prostate cancer cell line, by pooling selected fractions from a continuous sucrose gradient (within the density range of 1.1–1.2 g/ml), and examined under standard or lytic conditions by the SOMAscan™ array (version 3.0). Cell lysates were also prepared, and the profiles were compared. For comparison purposes, samples were prepared at the same total protein concentration, which was determined using the microBCA assay. *Results*: Exosomes were found to be enriched in over 100 proteins, compared to cells, and these included factors of known association with cancer exosomes (such as MFG-E8, integrins αvβ3 and MET), and also factors less widely reported as exosomally associated (such as Nogo Receptor, ITIH4, IL17RA, G-CSF). Many of the newly identified proteins were validated on these and on exosomes from other cancer cell lines using individual SOMAmer reagents, or antibodies. *Conclusion*: This protein array approach is a sensitive and effective tool for the analysis of exosome composition and has potential utility in a broad range of clinical scenarios as a high throughput basis for protein-biomarker discovery.

**Advanced molecular tools for sensitive detection of microvesicles and exosomes as biomarkers**

Kamali-Moghaddam Masood, J. Yan and D. Wu

Uppsala University, Sweden

Despite a large number of protein biomarker candidates presented in the literature, only a small group of proteins have been demonstrated to be clinically useful. Identification of reliable biomarkers in the blood requires access to technologies with sufficient specificity and sensitivity to meet the complexity of blood proteomic. In addition, the focus on proteins as biomarker has now being expanded to other biomolecules such as high-molecular-weight microvesicles and exosomes. We have previously described a sensitive and specific assay (4PLA) for detection of complex target structures such as exosomes in which the target is first enriched via an immobilised antibody and subsequently detected by using four other antibodies with attached DNA oligonucleotides. The requirement for coincident binding by 5 antibodies to generate an amplifiable reporter DNA molecule results in increased specificity and sensitivity. A 4PLA assay was established to detect prostasomes in plasma samples from prostate cancer patients with different Gleason scores, and age-matched healthy men are used to prove prostasome could be a potential biomarker for prostate cancer. Using 4PLA we were able to detect significantly elevated levels of prostasomes in blood samples from patients with prostate cancer compared with healthy men. The assay distinguished patients with high and medium prostatectomy Gleason scores (8–9 and 7, respectively) from those with low score (≤6), which reflect the disease aggressiveness. We have now established assays for several exosomes including 2 from colorectal cancer cell lines. Furthermore, we have developed a method for surface protein profiling of complex target molecules that enable us to distinguish exosomes originated from different sources.

**Exosomes secreted by human cells transport largely mRNA fragments that are enriched in the 3’-untranslated regions**

Igor V. Kurochkin and A.O. Batagov

Bioinformatics Institute, A*STAR, Singapore

*Introduction*: It was demonstrated that exosomal mRNA is functional in target cells as it could be translated into proteins. Cellular mRNAs vary in length from 400 to 12,000 nt. However, the majority of RNA present in exosomes has a size distribution between 25 and 700 nt. This suggests either that mRNAs are not major species of RNA delivered by exosomes or that exosomal mRNA is fragmented. *Material and methods*: The expression data on intracellular and exosomal RNAs were derived from GSM339549 and GSM339550. For every probe on Agilent 44K oligonucleotide array, exosome to cell enrichment ratio (ECER) was calculated. RNA expression was analysed using quantitative real-time RT-PCR (qRT-PCR). The expression ratio between RNA regions representing a given transcript was calculated. *Results*: We found that for about 60% of exosomal mRNAs, only a fraction of their corresponding probes was detectable on the array. Exosomal mRNA fragmentation is characterised with a specific structural pattern. The closer to the 3’-end of the transcript the fragments were localised, the larger fraction among the secreted RNAs it constituted. Supporting this, the proximity of probes to their transcript's 3’-end strongly positively correlated with their secretion efficiency as measured by ECER. These calculations have been confirmed by qRT-PCR analysis of exosomes secreted by human glioma cell line SF295. *Conclusions*: We provide evidence that exosomes secreted by human cells transport mostly mRNA fragments derived from 3'UTRs. Thus we need to reassess the assumption that RNA messages delivered by exosomes are mainly translated into proteins by recipient cells. Instead we propose that RNA fragments delivered by exosomes play regulatory roles as 3'UTRs contain elements that confer subcellular localisation of mRNA and are rich in miRNA-binding sites. Thus secreted 3'UTRs may act as competing RNAs to regulate stability, localisation and translation activity of mRNAs in target cells.

**Omics-technologies applied to the study of extracellular vesicles to identify candidate non-invasive biomarkers for liver injury**

E. Rodriguez-Suarez, J. Conde-Vancells, E. Gonzalez, F. Royo, L. Palomo and J.M. Falcon-Perez

CIC bioGUNE, Spain

Liver injury ranging from mild infection to life-threatening liver failure is a serious worldwide health issue, and a major goal in liver pathology is the identification of molecular markers for its early detection, that is, before clinical manifestations are produced. Our goal is to identify novel molecular biomarkers of liver injury that may be analysed in samples obtained non-invasively. Extracellular vesicles are small natural membrane vesicles released by a wide variety of cell types into the environment, and they can be detected in blood and urine samples awaking great interest in identifying non-invasive disease biomarkers. Previously, our group had demonstrated that hepatocytes are able to secrete extracellular vesicles enriched in metabolic enzymes. In the current work, we have achieved a thorough qualitative and quantitative analysis of the proteome of hepatocyte-derived extracellular vesicles challenged to different model toxins. By applying label-free quantitation proteomics, we were able to detect significant de-regulation of a number of proteins from these vesicles after treatment. We have validated in vivo some of these markers supporting the view that quantitative proteomics on extracellular vesicles using in vitro systems is a suitable strategy to unravel non-invasive candidate biomarkers for disease. Our work provides a repertoire of non-invasive candidate markers for liver damage.

**RNA profiling of exosomes**

Alexandre (Sasha) Vlassov, E. Zeringer, M. Li, T. Barta, J. Schageman, S. Magdaleno and R. Setterquist

Life Technologies, United States

Exosomes are 30–150 nm vesicles containing unique RNA and protein cargo, secreted by all cell types in culture. They are also found in abundance in body fluids, including blood, saliva, urine, CSF and breast milk. At the moment, the mechanism of exosome formation, the makeup of the cargo, biological pathways and resulting functions are incompletely understood. One of their most intriguing roles is intercellular communication – exosomes function as the messengers, delivering various effector or signaling macromolecules between specific cells. There is an exponentially growing need to dissect structure and the function of exosomes and utilise them for development of novel diagnostics and therapeutics. Here, we report characterisation of exosomal RNA cargo using qRT-PCR and next generation sequencing techniques. Analysis of the RNA content of exosomes derived from cell culture media, human blood serum, urine, saliva and milk revealed extremely diverse population of miRNA, mRNA, rRNA, piRNA, snoRNA, tRNA and other short ncRNA sequences (while minimal DNA, if any, was detected). Overall, exosomal cargo reflects the RNA content of the parental cell – in terms of sequences and levels of unique sequences – and thus is extremely valuable for diagnostic development. However, a number of RNA targets show significant differences in levels between the exosome and parental samples, thus opening a possibility of their utilisation as reliable markers. The reason for these differences is not fully understood but could provide useful information regarding sorting of particular RNA sequences into exosomes. The work presented here is the first step towards developing standardised techniques and protocols for isolation of exosomes and downstream analysis of their constituents. In a similar fashion, disease-specific RNA signatures residing within the exosomes can be discovered and used for the development of superior, sensitive and minimally invasive diagnostic alternative to biopsies.

**Multi-omics characterisation of subsets of platelet-derived extracellular vesicles (pl-evs) implicates their involvement in the pathogenesis of vascular and neurologic diseases**

Gerd Schmitz^1^, A. Pienimäki-Römer^1^, K. Kuhlmann^2^, T. Konovalova^1^, A. Boettcher^1^, G. Liebisch^1^ and E. Orso^1^

^1^University Hospital Regensburg, Germany; ^2^Ruhr University Bochum, Germany

*Introduction*: In order to characterise human platelet-derived extracellular vesicles (PL-EVs) and their impact in disease pathogenesis, human haemapheresis platelet concentrates (PLCs for transfusion) were investigated by multi-omics approaches. *Materials and methods*: Five subsets of PL-EVs (F1-F5) were isolated by filtration followed by a three-step differential density gradient ultracentrifugation. The EV-subsets were subjected to lipidomic, proteomic and transcriptomic characterisations. *Results*: PL-EV subsets F1-F2 (lowest density) show the highest content of free cholesterol (37% of platelet cholesterol) and express 50% of vesicular and endosomal sorting complex (ESCRT)-associated proteins (25%; ALIX, CD36, CD9). In addition F1 and F2 also contain 45% of all RNA-binding proteins (incl. Argonaute), reflecting a significant enrichment of miRNA in PL-EVs. Subsets F1-F2 are also enriched in CD62P, Annexin V and the Parkinson's disease-related α-synuclein. Subsets F2-F4 show the highest content of CD63 and LAMP-2. The neurodegeneration-associated amyloid-β precursor protein (APP) and the apoptotic lipid 7-ketocholesterol are peaked in subsets F3-F4. Subsets F3-F5 are enriched in proteins implicated in vascular diseases, such as caveolin-1, apolipoproteins (apo) -I, -J and E. The subset F5 (highest density) contains 42% of all mitochondrial proteins and show the highest content of lysophosphatidic acid (LPA) (0.4%), phosphatidylserine (PS) (19%), ceramide (Cer) (1.3%), phosphatidylglycerol (0.06%) and cardiolipin (CL) (0.4%). *Conclusions*: PL-EV-subsets show different miRNA, protein and lipid composition, indicating their involvement in diverse processes. Enrichment of neurodegeneration or vascular disease-related proteins in certain PL-EV-subsets suggests their potential involvement in these pathologies. The significantly higher content of PS, Cer, LPA, CL and mitochondrial proteins in F5 proposes this EV-subset of mitochondrial origin.

**Coffee and Poster Viewing April 19**

**Poster Sessions V-VI 15:15-16:00**

**Plenary Oral session 24 (Imperial Ballroom) April 19**

**Chair:** *J. Lötvall and X. Breakefield* 16:00-**17:00**

**The biology of small and large non-coding RNAs**

Phillip Sharp

MIT, United States

Phillip Allen Sharp, is an American geneticist and molecular biologist who co-discovered RNA splicing. He shared the 1993 Nobel Prize in Physiology or Medicine with Richard J. Roberts for “the discovery that genes in eukaryotes are not contiguous strings but contain introns, and that the splicing of messenger RNA to delete those introns can occur in different ways, yielding different proteins from the same DNA sequence”. The topic of Dr Sharp's presentation will be “The biology of small and large non-coding RNAs”.

**Parallel Oral Sessions 25-27 17:15-18:30**

**Oral Session 25 (Imperial Ballroom): Endocrine/Exocrine April 19**

**Chair: *E.I. Buzás and P. Robbins* 17:15-18:30**

**The origin of circulating CD36+ microparticles in type 2 diabetes**

L.F. Lincz^1^, M.J. Alkhatatbeh,^2^ A.K. Enjeti,^1^ S. Acharya^3^ and R.F. Thorne^2^

^1^Hunter Haematology Research Group, Calvary Mater Newcastle Hospital, NSW, Australia; ^2^School of Biomedical Sciences and Pharmacy, Faculty of Health, the University of Newcastle, NSW, Australia; ^3^Department of Diabetes, John Hunter Hospital, NSW, Australia

*Introduction*: Elevated plasma levels of the fatty acid transporter, CD36, have been shown to constitute a novel biomarker for type 2 diabetes mellitus (T2DM). We recently reported such circulating CD36 to be entirely associated with cellular microparticles (MPs). The aim of the present study was to determine the absolute levels and cellular origin(s) of these CD36+MPs in persons with T2DM. *Materials and Methods*: An ex vivo case-control study was conducted using plasma samples from 33 obese individuals with T2DM (BMI=39.9±6.4 kg/m^2^; age=57±9 years; 18M:15F) and age and gender matched lean and obese non-T2DM controls (BMI=23.6±1.8 and 33.5±5.9 kg/m^2^, respectively). Flow cytometry was used to analyse surface expression of CD36 together with tissue specific markers: CD41, CD235a, CD14, CD105 and phosphatidyl serine on plasma MPs. An ELISA was used to quantify absolute CD36 protein concentrations. *Results*: CD36+MP levels were significantly higher in obese people with T2DM (p<0.00001) and were primarily derived from erythrocytes (CD235a+=35.8±14.6%). In contrast, the main source of CD36+MPs in non-T2DM individuals was endothelial cells (CD105+=40.9±8.3% and 33.9±8.3% for lean and obese controls, respectively). Across the entire cohort, plasma CD36 protein concentration varied from undetectable to 22.9 µg/ml and was positively correlated with CD36+MPs measured by flow cytometry (p=0.0006) but only weakly associated with the distribution of controls and T2DM (p=0.021). Multivariate analysis confirmed that plasma CD36+MP levels were a much better biomarker for diabetes than CD36 protein concentration (p=0.009 vs. p=0.398, respectively). *Conclusions*: Both the level and cellular profile of CD36+MPs differ in T2DM compared to controls, suggesting that these specific vesicles could represent distinct biological vectors contributing to the pathology of the disease.

**The role of EVs in inflammation**

Edit Buzás

Semmelweis University DGCI, Hungary

The proposed presentation would summarise state-of-the-art information regarding the role of different types of EVs, both in acute and chronic inflammation (the latter being associated with autoimmune diseases, allergy, atherosclerosis and cancer). This talk will highlight recent advances in our knowledge of the roles of EVs in the induction, maintenance and regulation of inflammation, for example, by carrying and/or releasing damage associated molecular patterns (DAMPs), by delivering inflammatory or anti-inflammatory cytokines and by their cross-talk with other mediators.

**Muscle secreted exosome-like vesicles: a new paradigm for muscle-pancreas cross talk?**

Sophie Rome^1^, A. Forterre^2^, G. Vial^3^, A. Besse^3^, K. Chikh^3^, H. Vidal^3^, J. Rieusset^3^ and E. Lefai^3^

^1^CarMeN Laboratory (INSER1060/INRA1235), France; ^2^CarMen Laboratory (INSERM1060/INRA1235), France; ^3^CarMeN laboratory (INSERM1060/INRA1235), France

*Introduction*: Exosomes are nanovesicles derived from the late endosomal system, which are released from cells constitutively. Recently, proteins secreted by skeletal muscle have been shown to play important roles in intercellular communication. To determine whether exosomes would participate in this molecular dialogue, we isolated and characterised mouse Quadricep-released exosomes under normal conditions or during insulin-resistance (IR). Then, we determined if exosomes secreted by IR muscle could affect insulin secretion of pancreatic β-cells. *Materials and Methods*: C57/black6 mice were fed for 16 weeks with stardard chow diet (SD) or with SD enriched with 20% palmitate (HPD). Nanovesicles released from control mice (SD) quadriceps were characterised by proteomic, electron microscopy and western-blot (WB). The miRNA contents of SD and HPD nanovesicules from quadriceps was determined by TLDA arrays. β-cell MIN6 were incubated with 2µg of nanovesicles from SD or HP muscles, and insulin secretion was quantified by ELISA tests. *Results*: HPD mice were insulin-resistant based on insulin tolerance tests and showed altered insulin-stimulated PkB phosphorylation in gastrocnemius muscles. Proteomic analysis validated that quadricep-secreted nanovesicles have exosome-like (EL) properties and also contained specific muscle cell proteins. HPD muscles secreted less exosome-like vesicles (ELP) than SD (0.61µg+0.17 vs. 0.30µg+0.06, p<0.05) and had a higher size (20% increase). Seven miRNAs were differentially expressed between HPD and SD muscle-secreted ELV. Incubation of MIN6 with HPD exosome-like vesicles lead to a twofold decrease of insulin secretion compared with SD vesicles. *Conclusions*: During diet-induced insulin-resistance, myotubes release a new class of exosome-like vesicles which participate in functional alterations of pancreatic β-cells.

**Podocyte ectosome formation is increased in diabetic kidney disease**

D. Burger, J.F. Thibodeau, C.E. Holterman, K.D. Burns and C.R.J. Kennedy

Kidney Research Centre, Canada

*Background*: Diabetes is the leading cause of end stage renal disease. While molecular mechanisms underlying diabetic renal injury remain elusive, both hyperglycemia and increased glomerular pressure damage podocytes, specialised glomerular epithelial cells critical to renal filtration. In the present study, we examined whether podocyte ectosome formation reflects glomerular injury. *Methods*: We utilised a conditionally immortalised human podocyte cell line (HPOD) as well as two mouse models of progressive diabetic kidney disease: streptozotocin (STZ) and OVE26 mice. HPODs were either exposed to 10% equibiaxial cyclical stretch or high glucose conditions (HG, 25 mM). Cells were also treated with 20 mM mannitol as an osmotic control. To determine the in vivo significance of podocyte ectosome formation, we probed whether podocytes release ectosomes into urine in diabetic mice. Ectosomes were isolated from media/urine and quantified by Annexin V (total ectosomes) or podocalyxin (podocyte ectosomes) labelling and flow cytometry. *Results*: Cyclic stretch was associated with a 3-fold increase in ectosome release after 5 hours (P<0.01, n=6). Similarly, HG increased ectosome release 2-fold after 24 hours (P<0.05, n=6). Cyclic stretch in combination with HG was associated with a 4-fold increase in ectosome release (P<0.001, n=6). Mannitol had no effect on ectosome formation from either normal or stretched podocytes. Similarly, neither Ang II, nor TGF-β had any effect on podocyte ectosome formation over 24 hours (P>0.05, n=8). In vivo, both mouse models of diabetes displayed typical hallmarks of renal injury (proteinuria, mesangial expansion). STZ-treated mice displayed increased urinary podocyte ectosomes as compared with untreated mice (17478±8329 ectosomes/mg Creatinine vs. 7 ±7, P<0.05, n=5–7). Similarly, in OVE26 mice, urinary podocyte ectosomes were elevated compared with their wild-type littermates (6956±2386 vs. 9±9, P<0.01, n=5–8). Finally, urinary ectosome levels were positively correlated (r=0.47, P<0.05) with albuminuria, a measure of glomerular injury. *Conclusions*: Our results suggest that podocytes produce ectosomes which are released into urine and may be indicative of glomerular injury. Such processes may be mediated by mechanical stretch and hyperglycemic conditions.

**Microvesicle-mediated transfer of double stranded RNA POLY(I:C) delivers a pro-survival signal during trail-induced apoptosis of rheumatoid arthritis synovial fibroblasts**

Mojca Frank Bertoncelj^1^, B.A. Michel^2^, R.E. Gay^1^, S. Gay^1^ and A. Jüngel^1^

^1^Center of Experimental Rheumatology, University Hospital Zurich, Switzerland; ^2^Department of Rheumatology, University Hospital Zurich, Switzerland

*Introduction*: We have shown recently that Poly(I:C) (PIC)-induced monocyte-derived microvesicles (MV) induce production of pro-inflammatory cytokines and deliver a pro-survival signal during TRAIL-induced apoptosis of rheumatoid arthritis synovial fibroblasts (RASF). The exact mechanism of PIC entry into the cells remains unknown. In this study, we investigated whether monocyte-derived MV delivers PIC into RASF thereby contributing to the resistance of RASF to apoptosis. *Materials and Methods*: Flow cytometry and RNAse III digestion were used to study the association and transfer of fluorescently labelled PIC with monocyte-derived MV. QPCR was performed to analyse PIC-inducible genes in MV-treated RASF. TRAIL-induced apoptosis was assessed in RASF stimulated with PIC-induced MV or PIC-pre-incubated MV and in RASF transfected with PIC+Lipofectamine 2000 using Annexin V/PI staining and/or Immunoblot for cleaved caspase 3. *Results*: Fluorescently labelled PIC is incorporated in monocyte-derived MV during stimulation of monocytes with PIC and is in contrast to soluble PIC resistant to degradation with RNAse III. MV-associated PIC is still detected in cell culture supernatants of RASF, 46h after monocyte stimulation with PIC. PIC-induced MV efficiently transfers PIC to RASF and compared to MV from untreated cells (control MV) strongly induce the expression of several PIC-inducible genes, such as interferon-β. MV, pre-incubated with PIC, but not control MV, decrease TRAIL-induced apoptosis of RASF to a similar extent as PIC-induced MV. This further supports the anti-apoptotic action of MV-mediated delivery of PIC which is in sharp contrast with an extensive pro-apoptotic effect of lipofectamine-transfected PIC. *Conclusion*: Extracellular PIC associates with monocyte-derived MV and is effectively transferred to RASF via MV. In contrast to transfected cytosol-delivered PIC, MV-delivered PIC may preferentially activate different cellular pathways resulting in its pro-survival effects.

**Oral Session 26 (Plaza Ballroom): RNA analysis April 19**

**Chair: *L. Balaj and H. Tahara* 17:15-18:30**

**Microfluidic device for isolation and genetic analysis of microvesicles**

Jaehoon Chung, H. Shao, L. Balaj, X.O. Breakefield, R. Weissleder and H. Lee

Massachusetts General Hospital, United States

*Introduction*: Microvesicles (MVs) have emerged as a novel biomarker for diagnosis and prognosis of various human diseases. For example, circulating microvesicles in peripheral blood can be used to detect cancer with minimally invasive procedures. We herein present a new lab-on-chip system that can selectively enrich tumour-borne MVs and analyse their genetic information. *Materials and Methods*: The developed platform integrated three microfluidic components, with each part respectively performing MV capture, RNA extraction and RT-PCR. The first fluidic chamber was densely packed with microbeads that are functionalised with target-specific antibodies (e.g. EGFR) so as to capture cancer-MVs. The RNA contents of captured MV were then released by injecting lysis buffer; the second chamber packed with glass beads are then used to capture and purify the released RNAs. Finally, eluted RNAs are reverse-transcribed in the third chamber, and the amount of cDNA is quantified. *Results*: The MV capture by the system was examined by flowing streptavidin-coated beads (110 nm) through the first chamber filled with biotin-coated beads (10 µm). We could achieve >98% capture efficiency up to a flow rate of 1 mL/hr. Next, we characterised RNA extraction efficiency. MVs in lysis buffer were introduced and purified inside the glass-bead chamber. After purification, captured RNAs were eluted and transferred to the third chamber for RT-PCR. The amount of extracted RNAs by the fluidic system was comparable to those by a standard method (RNeasy Micro Kit, Qiagen). The fluidic platform significantly streamlined the entire assay procedure, allowing one-step, single-chip MV analysis. *Conclusions*: We have developed a fluidic-based platform that can perform target-specific MV capture, their RNA extraction, and on-chip RT-PCR. Replacing time-consuming and extensive sample preparation, we envision that the system could be a valuable tool for basic and clinical research by enabling rapid, one-step

**Distinct RNA profiles in sub-populations of extracellular vesicles (apoptotic bodies, microvesicles and exosomes)**

R. Crescitelli^1^, C. Lässer^2^, T.G. Szabö^3^, A. Kittel^3^, I. Dianzani^1^, E.I. Buzás^3^ and J. Lötvall^2^

^1^Department of Health Sciences, University of Eastern Piedmont, Novara, Italy; ^2^Department of Internal Medicine, Krefting Research Centre, University of Gothenburg, Gothenburg, Sweden; ^3^Department of Genetics, Cell- and Immunobiology, Semmelweis University, Budapest, Hungary

*Introduction*: Exosomes (EXOs), microvesicles (MVs) and apoptotic bodies (ABs) are extracellular vesicles (EVs) under intense investigation currently. As RNA profiles in different EV-studies differ significantly, the relative content of RNA in different EVs needs to be described. *Aim*: To determine RNA profiles in EXOs, MVs and ABs. *Method*: Sub-populations of EVs were isolated from the supernatant of 3 different cell lines (HMC-1 [human mast cell line], TF-1 [erythroleukemia cell line] and BV2 [mouse microglia cell line]), using 2 different centrifugation-based protocols. Cells were first removed by centrifugation (300g), and EVs in the supernatant were collected using differential centrifugations. Protocol 1: ABs were collected at 2000g. Supernatant was filtered through 0.8 m pores and MVs were pelleted by ultracentrifugation at 12,200g. Protocol 2: ABs and MVs were collected together at 16,500g. Supernatant was filtered through 0.2 m filters and EXOs were pelleted by ultracentrifugation at 12,0000g. RNA was isolated with miRCURYTM RNA Isolation Kit (Exiqon) and analysed with a bioanalyser. EV size has been analysed by electron microscopy (TEM). *Results*: RNA profiles showed that ABs (2000g) contain significant amounts of rRNA and small RNAs, whereas MVs (12,200g) contain no or little rRNA and low quantities of small RNAs. The pellet composed by both ABs and MVs (16,500g) showed the presence of significant amounts of rRNA and small RNAs, indicating that the rRNA contribution evolves from ABs and not from MVs. EXOs contain significant amounts of small RNAs but little or no rRNA Pellets were sectioned, and EVs were visualised by TEM. Different EVs had different size distribution, shape and electron density, characteristic of ABs, MVs and EXOs. *Conclusions*: We demonstrate that the sub-populations of EVs have very different RNA profiles and morphological characteristics. Centrifugation-based protocols are simple, fast and cheap approaches to distinguish different EVs.

**The small RNA content of different extracellular vesicle subsets released during immune cell interactions**

E.N.M. Nolte-'t Hoen^1^, M. Waasdorp^1^, W.J.C. Geerts^2^, W. Stoorvogel^1^ and M.H.M. Wauben^1^

^1^Department of Biochemistry and Cell Biology, Faculty of Veterinary Medicine, Utrecht University, Utrecht, The Netherlands; ^2^Department of Cellular Architecture and Dynamics, Utrecht University, Utrecht, The Netherlands

*Introduction*: We previously applied deep sequencing to analyse small RNAs in extracellular vesicles (EV) released during interaction of dendritic cells (DC) and T cells, which is a key event in the initiation of immune responses. Besides miRNAs, we found a large variety of small noncoding RNA species representing pervasive transcripts or RNA cleavage products overlapping with protein coding regions, repeat sequences, or structural RNAs. Among the most abundant released small RNAs were sequences derived from vault RNA, Y-RNA, and signal recognition particle (SRP) RNA, which are also known to be encapsidated as host RNAs by several retroviruses. The selective release and increasing evidence for their role in gene regulation strongly suggest that these RNAs are released to modify the function of target cells. It is currently unknown whether different sub-populations of EV contain different small RNA species. *Materials and methods*: We separated EV sub-populations released during DC-T cell interactions by buoyant velocity gradient ultracentrifugation. The RNA content of EV was analysed using RT-qPCR whereas their morphology was investigated using transmission electron microscopy (TEM). *Results*: Besides EV that reach their equilibrium density of ~1.15 g/ml after 14 h of ultracentrifugation (“fast-floating EV”), a specific subset of RNA-containing EV reached this equilibrium density only after 60 h of ultracentrifugation (“slow-floating EV”). The analysed miRNAs and vault-/Y-/SRP-RNA species distributed differently over these 2 EV sub-populations. Using TEM, slow-floating vesicle-like structures appeared morphologically different from fast-floating EV. *Conclusion*: We found evidence that during DC-T cell interactions, at least 2 different EV sub-populations are released that differ in migration velocity in density gradients, RNA content and morphology.

**The difference of serum RNA profile: RNA extraction method and detection method**

H. Akiyama^1^, S. Kondo^1^, S. Takizawa^1^, N. Kosaka^2^ and T. Ochiya^2^

^1^Toray Industries, Inc., Japan; ^2^National Cancer Center Research Institute, Japan

Serum miRNA profiles are widely reported by many research groups but they do not always coincide well. The reason of this difference is suggested to be, at least in some cases, due to a difference in the RNA extraction or detection method. In this report, we compare the miRNA expression profiles of RNAs extracted from (a) extracellular vesicles precipitated by ultra-centrifuging, (b) precipitation of ExoQuick, (c) whole serum by QIAGEN miRNeasy and (d) whole serum by Toray 3D-GeneTM RNA extraction reagent from liquid sample kit. In addition, the comparison of data obtained by different detection tools will be also shown. In these days, the whole miRNA expression profiling can be done by multiple qRT-PCR, microarrays, and Next Generation Sequencing. However, the feature of each method will reflect to the obtained data, and the data obtained by different tools do not match perfectly. We will compare the serum miRNA expression profiles detected by (a) “TaqMan” Array MicroRNA Card, (b) QIAGEN miScript miRNA PCR Array and (c) “3D-Gene” Human miRNA oligo chip. We will discuss the base of method for serum miRNA profiling, and the difference between the detection methods, with or without pre-amplification.

**Clustering and candidate motifs detection in exosomal mirnas**

D. Gupta, P. Gaur

University of ALLAHABAD, India

The world of exosome research is growing rapidly since last few years and nowadays specially with concern to miRNAs derived from them. The clustering pattern and motifs give immense information about miRNAs and a way to analyse newly discovered miRNAs in exosomes more informatively. A computational approach for detecting candidate motifs and clustering patterns in miRNAs derived from exosomes is presented over here. Details of miRNAs carried by exosomes were derived from 'Exocarta' compendium for the species *Homo sapiens* and *Mus musculus*. The proposed method has made the use of some algorithms to perform the computational tasks and the results were compared and validated with available online web tools like BLASTN (for similarity search) and MEME (for motifs). This computational approach gives a good way to analyse miRNAs while making use of several different algorithms at a time which is not always feasible using web tools. The analytics on newly discovered miRNAs in exosomes could facilitate search of new biomarkers.

**Oral Session 27 (Georgian): Tissue injury April 19**

**Chair: *B. van Balkom and B. Gyorgy* 17:15-18:30**

**Extracellular vesicles in pregnancy**

Ian Sargent

Nuffield Department of Obstetrics and Gynaecology, University of Oxford, Oxford, UK

Extracellular vesicles (EV) are known to be shed by a large number of cell types, both as part of normal physiology and disease processes. Pregnancy adds a new dimension to this subject as there is a foreign body, the foetus, added to the equation which contributes to the population of EV circulating within the mother. Exactly, it is not foetus but the placenta which is the primary source of these vesicles. Any vesicles released from placenta will pass directly into the maternal circulation where they will engage with maternal immune and cardiovascular systems. The consequences of this vesicle shedding are believed to be important for the maintenance of normal pregnancy; constituting a major signalling mechanism between mother and foetus. Conversely, abnormal shedding of vesicles has been implicated in disorders of pregnancy, in particular pre-eclampsia and, more recently, pre-term birth. Although often ignored by mainstream science, pregnancy affords a unique opportunity to study EV in normal physiology and disease. Firstly, placenta carries specific markers which allow the vesicles to be distinguished from those produced by other cell types in blood. Secondly, unlike any other condition, it is known exactly when the pregnancy begins and when it ends, allowing changes in vesicle shedding to be followed throughout the normal and disease states and thirdly, the source of the vesicles, the placenta, is uniquely available at the end of the pregnancy for study. Importantly, circulating placental vesicles allow us to take, in effect, non-invasive placental biopsies at different stages of pregnancy, providing information about its function and having significant potential as biomarkers.

**Differential distribution of pre-eclampsia biomarkers in two distinct populations of membrane vesicles from plasma**

Tan Soon Sim^1^, S.K.S. Newman^2^, K.H. Tan^3^ and S.K. Lim^1^

^1^Institute of Medical Biology, Singapore; ^2^School of Biological Sciences, Nanyang Technological University, Singapore; ^3^Department of Maternal Fetal Medicine, KK Women's and Children's Hospital, Singapore

*Introduction*: Bodily fluids including plasma contain different types of membrane vesicles such as exosomes, apoptotic bodies etc. These membrane vesicles could serve as sentinels of disease and disease progression as their type, composition and biological activities depend on the pathophysiological state of the secreting cells. Membrane vesicles could be used to discover biomarkers and this is advantageous in circumventing the confounding effect of high abundance proteins such as albumin and immunoglobulins. Since membrane vesicles constitute only a fraction of plasma which is a complex biological fluids, assaying for biomarkers within vesicles will be inherently more sensitive. *Material and Methods*: Two distinct types of lipid membrane vesicles from plasma of severe pre-eclampsia pregnant women and matched controls were isolated by magnetic bead technology based on their different affinities for cholera toxin B chain and Annexin V that binds GM1 ganglioside and phosphotidylserine respectively. The isolated vesicles were assayed for several reported pre-eclampsia biomarkers using enzyme-linked immunosorbent assays (ELISAs) and antibodies array. *Results*: Less than 1% of plasma protein was present in each of the 2 plasma membrane vesicle preparations. Several previously reported candidate plasma biomarkers, ANP, BNP, sFlt-1, PlGF and CRP, were investigated for their presence in circulating membrane vesicles and whether their levels correlate with pre-eclampsia. Using p<0.01 to define statistically significance in this study, we observed that the level of ANP, BNP, sFlt-1 and PlGF in CTB-bound membrane vesicles, and the level of Flt1 and PlGF in AV-bound membrane vesicles were higher in women with pre-eclampsia. Of the 4 candidate biomarkers reported to be elevated during preeclampsia, namely ANP, BNP, sFlt-1 and CRP, 3 (ANP, BNP and sFlt-1) were elevated in the CTB-bound vesicles and only one (sFlt-1) was elevated in the AV-bound vesicles. PIGF, which was reported to be reduced during preeclampsia, was elevated in both CTB- and AV-bound vesicles. *Conclusions*: These results provide proof of concept that the proteins within plasma vesicles can be potential biomarkers for pre-eclampsia diagnosis and prognosis. The differential distribution of the reported pre-eclampsia biomarkers between the 2 different plasma vesicles provide additional levels of accuracy and precision.

**Plasma extracellular vesicles as biomarkers and mediators of sepsis-associated acute kidney injury: potential protective role of blood purification techniques**

Vincenzo Cantaluppi^1^, F. Figliolini^1^, D. Medica^1^, A.D. Quercia^1^, S. Dellepiane^1^, A. Pacitti^2^, G.P. Segoloni^1^, C. Tetta^3^ and G. Camussi^1^

^1^University of Turin, Italy; ^2^Cuneo Hospital, Italy; ^3^Fresenius Medical Care, Germany

*Introduction*: Sepsis is the predominant cause of acute kidney injury (AKI) and mortality in critically ill patients. During sepsis, extracellular vesicles (EVs) are released from activated leukocytes and platelets playing a potential role in AKI. The aims of this study were to evaluate: (a) the role of plasma EVs as mediators of AKI and biomarkers of disease severity, (b) the protective role of blood purification techniques through removal of plasma EVs. *Methods*: We analysed all patients treated by dialysis for AKI in the period 2001–10. Patients’ outcome was assessed 28 days after study admission. In 30 patients with sepsis and AKI, plasma samples were collected for EV analysis (Nanosight, FACS, proteomic and RNA profiling) during different blood purification techniques. We also evaluated the effects of plasma EVs on cultured human kidney-derived endothelial and tubular epithelial cells. *Results*: Thousand eight hundred and thirty three patients were treated by dialysis, and sepsis was the main cause of AKI: 415/1833 (22.6%). We distinguished 2 groups: sepsis (S) and non-sepsis (NS, 1418/1833: 77.4%). In the S group, mortality was 302/415 (72.9%) whereas in NS group was 804/1418 (56.7%), p<0.05. We observed a 6- to 8-fold increase of EV plasma concentration in S vs. NS group. EVs in S group showed an enhanced expression of Fas-ligand, CD40-ligand and an enrichment for mRNAs and microRNAs involved in inflammation and apoptosis. EVs were internalised in cultured kidney cells inducing functional alterations and cell death. Blood purification techniques performed using citrate as anticoagulation associated with a decrease of plasma EV levels and EV-induced cell injury. *Conclusions*: In sepsis, plasma EVs are potential biomarkers of outcome and are involved in the pathogenetic mechanisms of AKI through the triggering of apoptosis in kidney cells. Blood purification using citrate may limit the release of EVs from activated leukocytes and platelets protecting from AKI and multiple organ failures.

**Extracellular vesicles derived from mesenchymal stem cells promote renal cell recovery after ischemic injury through miRNA regulation**

R.S. Lindoso^1^, F. Collino^2^, S. Bruno^2^, A. Vieyra^1^, M. Einicker-Lamas^1^ and G. Camussi^2^

^1^Carlos Chagas Filho Biophysics Institute – UFRJ, Brazil; ^2^Molecular Biotechnology Center-UNITO, Italy

*Introduction*: Mesenchymal cells (MSC) are known by its potential use in tissue regeneration. In renal injury, MSC promote renoprotection and stimulates proliferation. The extracellular vesicles (EVs) are possible mediators of these effects through the transference of miRNAs, which are involved in the regulation of several mechanisms, including repair processes. The aim of this study was to analyse the role of EVs in renal repair, identifying the miRNAs that are involved in these processes. *Material and methods*: HK-2 cells (human renal epithelial cells) were treated with antimycin A (20 µM) for 1 h, promoting ATP depletion. EVs were obtained from supernatants of human MSCs (hMSCs), cultured overnight in RPMI with 0.5% of BSA and submitted to a centrifugation of 6,000 g for 20 min followed by 150,000 g for 1 h at 4°C. Incorporation of EVs, obtained from hMSC previously stained with Vybrant™ Dil and SYTO^®^ RNASelect™, in HK-2 cells was analysed by confocal microscopy. Proliferation was evaluated by viable cell counting (Trypan Blue). Cell death assay was performed by FACS analysis (Annexin V/propidium iodide). Profiling of 365 mature miRNAs was performed by TaqMan^®^ Arrays, and qRT-PCR was used to confirm some miRNAs. *Results*: The EVs inhibited apoptosis (50% reduction) and led to an increase (50%) in proliferation after injury. The EV incorporation increased 100% after injury and the CD29, CD44 and alpha V receptors are involved in this process. The transference of RNAs present in MVs was also observed in renal cells. The qRT-PCR analysis identified several miRNAs modulated by the injury and treatment with MVs. *Conclusions*: These results show that EVs promote renal recovery, reducing apoptosis and increasing proliferation. EVs can transport RNAs inside renal cells after injury and modulate miRNA expression. We demonstrated the potential of EVs in the treatment of renal diseases, and the involvement of miRNAs in these processes.

**Transfer of monocrotaline-induced pulmonary hypertension to healthy mice via extracellular vesicles**

Jason Aliotta, M. Pereira, A. Sorokina, S. Wen, M.S Dooner, D. Adler, E. Papa, M. Deltatto, L. Goldberg, P.J. Quesenberry and J.R. Klinger

Rhode Island Hospital, USA

*Introduction*: We have shown that extracellular vesicles (EVs) isolated from mice with monocrotaline (MCT)-induced pulmonary hypertension (PH) induce PH in healthy mice. A crosstalk between the pulmonary vasculature and bone marrow has been described in PH, as the pulmonary endothelium releases factors that influence bone marrow (BM) differentiation and more circulating BM-derived progenitor cells are in patients with PH. We hypothesise that EVs from MCT-injured mice induce PH by interacting with the pulmonary vasculature or by influencing BM cell phenotype. *Materials and methods*: Lung and plasma EVs (LEVs, PEVs) from MCT-injured and control mice were co-cultured with pulmonary endothelial cells (ECs) or BM-derived lineage-depleted cells (Lin-). Co-cultured Lin- cells were transplanted into irradiated mice. Forty two days later, recipient right ventricular (RV) hypertrophy was assessed by RV-to-body weight (RV/BW) ratio (mg/g) and pulmonary vascular re-modelling by blood vessel wall thickness-to-diameter (WT/D) ratio. Apoptotic co-cultured ECs were quantified. *Results*: RV/BW and WT/D ratios were similar in mice transplanted with Lin- cells+control LEV, PEV versus Lin- cells cultured without EV but higher mice transplanted with Lin- cells+MCT LEV vs. control LEV (1.29+0.16 vs. 0.65+0.012mg/g; 0.121+0.012 vs. 0.069+0.009%, p<0.05) and MCT PEV vs. control PEV (1.41+0.25 vs. 0.63+0.14mg/g; 0.131+0.011 vs. 0.061+0.009%, p<0.05). ECs+control EVs had similar rates of apoptosis vs. ECs cultured without EV. ECs+MCT EVs had significantly lower rates of apoptosis vs. control EVs (LEV, 2.2+0.9% vs. 7.1+0.6%; PEV, 1.8+0.7% vs. 7.9+0.7%, p<0.05). *Conclusions*: EVs from MCT-injured mice induce features of PH in healthy mice, potentially by inducing an anti-apoptotic phenotype in pulmonary vascular endothelial cells, a mechanism known to be important in the pathogenesis of human PH. EV may also alter BM cell phenotype which in turn contributes to the development of PH.

**Poster Sessions V-VI April 19 8:30-17:30**

**Poster Session V (Arlington-Berkeley): Immune system April 19**

**Chair: *M. Wauben and P. Askenase***

**Serum-derived extracellular vesicles modulate host-microbe responses by altering microbe induced TLR activity and phagocytosis**

Jeroen van bergenhenegouwen^1^, L. Rutten^1^, N. Kettelarij^1^, A.D. Kraneveld^2^, J. Garssen^2^ and A.P. Vos^1^

^1^Danone research Centre for Specialised Nutrition, The Netherlands; ^2^Department of Pharmacology, Utrecht Institute for Pharmaceutical Sciences (UIPS), Utrecht University, The Netherlands

*Introduction*: Extracellular vesicles (EV) can be purified from virtually any body fluid and consist of various types of membrane vesicles that are released from cells. The previously reported presence of pattern recognition receptors (PRR; including but not limited to TLRs) on EVs triggered the hypothesis that serum derived EVs can intervene with TLR activity. To that end we examined the effect of intact human serum (HS), EV depleted human serum (HS-D) and collected EVs (HS-EV) for their capacity to modulate host-microbe responses. Here we describe a previously unrecognised role of EVs in modulating host-microbe responses. *Materials and methods*: Heat-inactivated serum-derived EVs were collected using ExoQuick according to the manufactures instructions. Depleted serum and vesicle-containing pellets were collected. *Results*: THP-1 and HEK transfectant TLR2/6 and TLR5 responses were reduced by HS or HS-EVs but not HS-D. THP-1 and HEK transfectant TLR4 activity was increased by HS, HS-EV and HS-D. Non-TLR dependent responses were not affected. In-line with the effects of HS, HS-EV and HS-D on specific TLR responses, moDC-derived IL-6 and TNFa release was differentially affected upon stimulation with bacteria expressing different surface TLR-ligands (*B. breve*, TLR2/6; *L. rhamnosus*, no or concealed surface TLR ligands; *S. typhimurium*, TLR2/1,TLR4, TLR5). Co-culturing moDCs and bacteria resulted in bacterial aggregation which was dependent on the presence of HS-EVs. In addition, moDC phagocytosis was found to be increased by HS-EVs, with the largest effects seen on bacteria devoid of detectable surface TLR ligands. *Conclusion*: Serum-derived EVs bind bacteria and modulate specific TLR and moDCs responses, thereby significantly altering host-microbe responses. More research is needed to investigate the biological relevance of these mechanisms.

**B cell-derived exosomes activate naive B cells—implication for vaccine design**

C. Gutzeit^1^, M. Gentile^2^, N. Nagy^3^, J. Gumz^1^, H. Vallhov^1^, E. Klein^1^, A. Cerutti^2^, S. Gabrielsson^1^ and A. Scheynius^1^

^1^Department of Medicine Solna, Translational Immunology Unit, Karolinska Institutet, Sweden; ^2^Institut Municipal d'investigation Medica (IMIM), Barcelona, Spain; ^3^Department of Microbiology, Tumor and Cell Biology, Karolinska Institutet, Stockholm, Sweden

*Introduction*: Exosomes are nano-sized membrane vesicles released from a variety of cells. They have been demonstrated to have immune stimulatory-, inhibitory- or tolerance-inducing effects, depending on their cellular origin. Therefore, they are investigated for use in vaccine and immune therapeutic strategies. In this study, we asked whether B cell-derived exosomes can influence B cell biology and thereby skew B cell responses. In a previous study, we observed that the exosomes derived from an Epstein-Barr virus (EBV) transformed B cell line (LCL1) target B cells through the gp350-CD21 ligand-receptor interaction. Additionally, LCLs release exosomes which harbour EBV latent membrane protein 1 (LMP1). LMP1 signalling replaces CD40 signalling in B cells in vivo and induces proliferation and class-switch recombination (CSR). *Material and methods*: Here, we used human B cell-derived exosomes to study their capacity to shuttle LMP1 to naïve B cells thereby influencing human B cell biology. *Results*: Our data suggest that human B cell-derived exosomes bind to primary naïve B cells and after uptake are able to induce proliferation, differentiation and CSR. *Conclusion*: B cell-derived exosomes might be of use to design and engineer exosomes for immunotherapy or vaccine approaches where the skewing of B cell responses is desired.

**Neutrophil-derived microvesicle numbers are increased by TNF-α treatment through caspase-8 and NF-κB activation**

B.L. Johnson, P.S. Prakash and C.C. Caldwell

University of Cincinnati, Cincinnati, OH, USA

*Introduction*: Sepsis remains the leading cause of mortality in intensive care units. Microvesicles can be generated from both activated or apoptotic cells, both of which are known to occur during sepsis. Previously, we have demonstrated that neutrophil-derived microvesicles (NDMVs) are produced at inflamed foci during sepsis or pneumonia. In the current set of experiments, we sought to elucidate molecular mechanisms underlying NDMV generation. *Methods*: Two distinct neutrophil isolation procedures were utilised to assess mechanisms underlying NDMV production: (a) isolation after a peritoneal injection of thioglycolate (TGA) and (b) isolation from bone marrow of untouched mice. These isolated neutrophils were treated with a variety of mediators that altered NDMV numbers as determined by flow cytometry. *Results*: First, we found that TGA-elicited neutrophils produced NDMVs, ex vivo, without any additional stimuli. This production was enhanced by the activation of myosin light chain kinase, caspase 8 and Rho kinase. Additionally, we determined that NDMV generation could be inhibited by increased intracellular cAMP pathway and activation of PKA. Secondly, we evaluated whether TNF-α treatment upon neutrophils isolated from bone marrow could generate NDMVs. We observed that TNF receptor 1 (TNFr1) and TNF receptor 2 (TNFr2) activation independently and additively increased NDMV numbers. Caspase 8 inhibition diminished NDMVs generated through TNFr1 activation, and inhibition of NF-κB abrogates NDMVs generated after activation of both TNFr1 and TNFr2.

**Active immunisation with *Enterobacter aerogenes*-derived outer membrane vesicles protects sepsis-induced lethality via T cell-mediated immunity**

W. Lee, S. Choi, O. Kim, T. Shin, S.G. Jeon, Y.S. Gho and Y. Kim

Pohang University of Science and Technology, Pohang, Republic of Korea

*Background*: Sepsis is characterised by a whole body inflammatory state and the presence of bacterial infection. In post-antibiotic era, the intensive use of antibiotics has dramatically increased the frequency of resistance amongst human pathogens. *Enterobacter* spp. has developed broad-spectrum resistance to multiple classes of antibiotics. *Objective*: To evaluate the effects of vaccination with *Enterobacter aerogenes*-derived outer membrane vesicles (OMV) in the prevention of lethality induced by *Enterobacter* spp. *Methods*: *E. aerogenes*-derived OMV were isolated and immunised intraperitoneally to 6 weeks old C57BL/6 mice thrice. *E. aerogenes* OMV-reactive IgG antibody in the serum and the production of T cell cytokines from splenocytes were measured after the immunisation. The effects of the immunisation with *E. aerogenes*-derived OMV were evaluated in a sepsis mouse model, induced by interperitoneal injection of *Enterobacter* spp. *Result*: Immunisation with *E. aerogenes* OMV induced the production of serum OMV-reactive IgG, OMV-specific IFN-gamma and IL-17 from splenocytes. In addition, the immunisation with *E. aerogenes* OMV prevented lethality induced by *E. aerogenes* and *E. clocae* infection. Moreover, adoptive transfer of splenocytes or T lymphocytes isolated from OMV-immunised mice protected naive recipient mice against *E. aerogenes*-induced lethality. *Conclusion*: *E. aerogenes*-derived OMV can be a good vaccine candidate, and proteins in the OMV are useful vaccine targets to prevent *Enterobacter* infection. Our data continue to build a framework depicting the molecular mechanisms regulating NDMV generation. Our observations that TNF-α, caspase 8 and NF-kB can increase NDMV numbers agree with our earlier observation of increased accumulation of NDMVs under inflammatory conditions. Future experiments will allow us to determine what impact NDMVs have upon leukocytes in the inflammatory milieu.

**T cell derived microvesicles induce mast cells production of IL-24: a possible link to inflammatory skin diseases**

I. Shefler^1^, M. Pasmanik-chor^2^, Y.A. Mekori^3^ and A.Y. Hershko^4^

^1^Meir Medical Center, Kfar Saba, Israel; ^2^Tel Aviv University, Tel Aviv, Israel; ^3^Meir Medical Center and Tel Aviv University, Israel; ^4^Meir Medical Center and Tel Aviv University, Israel

*Background*. It has recently been shown that microvesicles derived from activated T cells can stimulate human mast cells. This pattern of activation involved the MAPK system and resulted in degranulation and the release of several cytokines such as IL-8 and oncostatin M (J Immunol 2010;185:4206). To characterise this novel pathway of mast cell activation, we analysed the specific gene expression profiling by microarray analysis and identified that this stimulation leads to the production of several cytokines and chemokines, here-to-fore unknown in mast cells, such as IL-24. *Methods*: T cell-derived microvesicles were labelled with PKH67 to allow visualisation of their interaction with human mast cells. Consequent gene expression profiling was studied by whole genome microarray and analysed for the identification of cellular pathway clusters. Expression of three selected genes, CCL3, CCL7 and IL-24, was validated by qRT-PCR and specific ELISA. IL-24, that has not been here-to-fore recognised in mast cells, was also tested for its effect on keratinocyte STAT3 phosphorylation and for its presence in mast cells in psoriatic skin lesions. *Results*: The uptake and internalisation of T cell-derived microvesicles into human mast cells occurred within 24 hrs. This led to the robust upregulation of several clusters of genes, notably those that are cytokine-related. Amongst these, IL-24 appeared to be a hallmark of microvesicle-induced activation. Mast cell-derived IL-24, in turn, activates keratinocytes *in vitro* as manifested by STAT3 phosphorylation and is produced in mast cells within psoriatic lesions. *Conclusion*: Production of IL-24 is a unique feature of microvesicle-induced mast cell activation, as its production by these cells has not been recognised so far. We propose that this mast cell-derived cytokine may contribute to the pathological findings in T cell-mediated skin inflammation.

**Phenotype and function of γδT cell exosome-like vesicles: potential for therapeutics?**

J.L. Welton^1^, J.M. Falcon-Perez^2^, D. Gil^3^, M. Clement^4^, L. Wooldridge^4^, M. Eberl^4^ and A. Clayton^5^

^1^Cardiff Institute of Infection and Immunity and the Institute of Cancer and Genetics, School of Medicine, Cardiff, UK; ^2^CIC bioGUNE, Derio, Bizkaia, Spain; ^3^Structural Biology Unit, CIC bioGUNE, Derio, Spain; ^4^Cardiff Institute of Infection and Immunity, School of Medicine, Cardiff University, Cardiff, UK; ^5^Institute of Cancer and Genetics, School of Medicine, Cardiff University, Cardiff, UK

*Introduction*: A small subset of unconventional human T cells, γδT cells, play complex roles in host immunity against pathogens and cancers. A key function is antigen presentation, comparable to that of dendritic cells. These cells are easily expandable and potentially useful therapeutically. Here, we characterise the phenotype and function of extracellular vesicles secreted by these cells. *Materials and methods*: Vesicles were purified from γδT cell-conditioned medium by serial ultracentrifugation, and analysed by electron microscopy (EM), nano-particle tracking (NanoSight), flotation on sucrose gradients, western blot and flow cytometry. Activation of αβCD8+ T cells was assessed by secretion of MIP-1β, a potent proinflammatory chemokine (ELISA), following the addition of peptide-pulsed γδT cell-derived exosome-like vesicles. *Results*: Cryo-EM and nano-particle tracking revealed majority of vesicles to be <100 nm, with very small electron dense (~30 nm) vesicles being the most prevalent. Flow cytometry showed the expression of γδT cell receptor, MHC Class I and II and tetraspanins. Multivesicular body and lysosomal markers, TSG101 and LAMP2, were observed by Western blot. Flotation on a sucrose gradient revealed that γδT cell-derived vesicles float at a density (1.23–1.27 g/ml) outside the range typical for exosomes. Preliminary results show that γδT cell exosome-like vesicles trigger MIP-1β secretion from αβCD8+ T cells, in an antigen peptide-dependent manner. *Conclusions*: Here, we describe a previously unstudied exosome source, showing that γδT cells produce a heterogeneous population of mainly small and dense vesicles phenotypically similar to exosomes. These vesicles exhibit peptide-presenting function, activating chemokine secretion by antigen-specific αβCD8+ T cells. Future work will evaluate and compare the potency of these exosome-like vesicles with other sources of T cell activation, as well as their potential to be therapeutically valuable.

**Exosomes released by chronic myelogenous leukemia cells modulate γδT cell activities**

L. Saieva^1^, S. Taverna^1^, A. Flugy^1^, S. Meraviglia^1^, M. Eberl^2^, F. Dieli^1^, G. De Leo^1^ and R. Alessandro^1^

^1^University of Palermo, Palermo, Italy; ^2^Cardiff University, Cardiff, UK

*Introduction*: Exosomes are small vesicles of 40–100 nm diameter of endosomal origin which are secreted from different cell types, including cancer cells. Several data from the previous years have showed that exosomes are messengers in intercellular communication and that tumour cells can use these vesicles to affect immune function. Some reports detail lymphocyte activation following tumour–exosome interactions while others describe immune-suppressive effects. The ability of tumour cells to evade or suppress an active immune response is considered to be a significant factor in the development and progression of tumours. We studied the effects of exosomes released by Chronic Myelogenous Leukemia (CML) cell line, K562, on γδT cell activities, to better understand the interaction between cancer-exosomes and the immune system. *Materials and methods*: Exosomes were isolated and purified from K562 cells by ultracentrifugation of conditioned culture medium. Exosomes were added to γδT cells at different doses and analysed for NKG2D, CD69, CD25, IFNγ and TNFα. To determine proliferation and apoptosis, γδT cells were stained for CFSE and Annexin V. *Results*: We demonstrate that CML-derived exosomes are able to downregulate NKG2D receptor, CD69/CD25 activation marker and impair the ability of γδT cells to produce IFN γ and TNFα, suggesting their inhibitory effect on lymphocytes function. Finally, we show that addition of exosomes to γδT cells impair their proliferation and induce apoptosis. The suppressive activity of K562 exosomes was susceptible to inhibition with SB-431542 and involved a crucial role of the TGFβ, suggesting that TGFβ probably might be the main immunosuppressive cytokine present in our exosome preparation responsible for the observed antiproliferative and inhibition effect. *Conclusions*: These findings suggest a role of CML exosomes in the modulation of γδT cells function and increase our knowledge on exosomes-mediated immune-escape mechanism.

**Effect of human melanoma exosomes on the function of antigen-specific CD8+ T cells**

M. Bürdek^1^, V. Huber^1^, P. Squarcina^1^, A. Cova^1^, P. Romero^2^, V. Umansky^3^, P. Altevogt^3^, C. Castelli^1^, B. Seliger^4^ and L. Rivoltini^1^

^1^Fondazione IRCCS Istituto Nazionale dei Tumori, Milan, Italy; ^2^Ludwig Center for Cancer Research, University of Lausanne, Lausanne, Switzerland; ^3^German Cancer Research Center, Heidelberg, Germany; ^4^Martin Luther University Halle-Wittenberg, Halle, Germany

*Introduction*: Tumour exosomes carry molecules involved in CD8+ T cell triggering, such as HLA-class I and tumour antigens. However, little is known about their ability to directly engage the T cell receptor (TCR) and the functional outcome of this interaction. We investigated HLA-A2 expression on melanoma exosomes and their ability to directly interact with melanoma-specific, HLA-A2-restricted CD8+ T lymphocytes. *Materials and methods*: Exosomes were derived from different melanoma cells, including two clones of the same line expressing HLA-A2 (Mel624.38) or not (Mel624.28). Vesicles were isolated by differential centrifugations and analysed for their expression of CD63, tumour-associated antigens and HLA-A2 molecules by flow cytometry of exosome-coated latex beads and Western blot. The stimulatory capacity of these exosomes on HLA-A2-restricted, CD8+ T cell clones or oligoclonal cell lines derived from tumour infiltrating lymphocytes and displaying known antigen-specificity was tested by IFNγ ELISpot, cytokine release assay and flow cytometry. *Results*: High amounts of HLA-A2 were detected on the surface of Mel624.38 exosomes. However, levels of HLA-A2 expression drastically depend on cell culture conditions. HLA expression was associated with a significant activation of antigen-specific, CD8+ T cells, detected by IFNγ release and upregulation of the activation markers, CD25 and CD137. Interestingly, not all molecules linked to TCR triggering appear to be modulated by exosome-T cell interaction and T cell stimulation. This was strictly dependent on the level of HLA-A2 expressed by exosomes. *Conclusions*: Melanoma cell-derived exosomes may directly engage and stimulate antigen-specific, CD8+ T cells. Whether exosome-TCR interaction can lead to full T cell activation, suboptimal triggering due to inefficient TCR cross-linking or even induction of anergy and apoptosis is presently under investigation, as well as the analysis of involved signalling pathways.

**Bacterial-protoplast derived nanovesicles as novel antigen delivery system for potent humoral and cellular immune response**

O.Y. Kim^1^, S.J. Choi^1^, S.C. Jang^1^ W.H. Lee^1^ J. Lötvall^2^, Y.K. Kim^1^ and Y.S. Gho^1^

^1^Department of Life Science, Pohang University of Science and Technology, Pohang, Republic of Korea; ^2^Department of Internal Medicine, Krefting Research Centre, University of Gothenburg, Gothenburg, Sweden

*Introduction*: With spreading resistance of existing pathogens to standard interventions, the development of effective and safe vaccine platform is crucial for maintaining a healthier life. This need for a new vaccine delivery system has brought our attention to the use of outer membrane vesicles (OMVs), a nano-sized membrane vesicle secreted from bacteria, as a novel vaccine candidate. However, OMVs possess toxicity issue as OMVs include components of bacterial toxins. To circumvent these problems, we newly designed bacteria protoplast-derived nanovesicles (PDNVs), deprived of outer membrane components, as novel antigen delivery system. *Materials and methods*: PDNVs were fabricated by the breakdown of *Escherichia coli* protoplast through a nano-sized filter. The immunogenicity of PDNVs was confirmed by its ability to maturate dendritic cells and induce antigen-specific humoral and cellular adoptive immunity. To examine the vaccination effect, mice were immunised with PDNVs loaded with bacterial antigens for 3 weeks with 1 week interval. The immunised mice were challenged with lethal dose bacteria and survival was monitored. *Results*: PDNVs and OMVs derived from the same bacteria had similar size and vesicular shape, but PDNVs had significantly higher productivity with high antigen loading capacity and no adverse effects were observed when administered to mice. In addition, PDNVs induced the maturation of dendritic cells, and mice immunised with antigen loaded PDNVs developed effective antigen-specific antibodies, as well as memory T cell response. Moreover, mice vaccinated with bacterial antigen-loaded PDNVs survived the antibiotic resistant super bacteria challenge. *Conclusions*: Here, we showed that PDNVs, non-living nanocarrier devoid of outer membrane content, induced potent antigen-specific humoral and cellular immune response without any adverse effects. This suggests that PDNVs are novel candidates as new antigen delivery system.

**Poster Session V (Arlington-Berkeley): Microenvironment April 19**

**Chair: *M. Belting and H. Peinado***

**Tumour-derived microvesicles enhance EMMPRIN-dependent human breast cancer invasion**

K. Menck, T. Pukrop, L. Dyck, C. Binder and F. Klemm

Department of Hematology/Oncology, University Medical Center Goettingen, Goettingen, Germany

*Introduction*: The establishment of a favourable tumour niche is a key step in tumour progression. Recently, plasma membrane-derived microvesicles (diameter 100–1,000 nm) have been identified as novel mediators in tumour-stroma interactions. However, little is known about the effect of tumour-derived microvesicles (T-MV) on the tumour cells themselves. *Materials and methods*: T-MV were isolated from the supernatant of human breast cancer cells by differential ultracentrifugation, characterised by Western Blotting and tested for their influence on tumour invasion in Boyden chamber assays. *Results*: T-MV were able to highly enhance human breast cancer invasion in an autologous and heterologous way. In contrast, they had no effect on benign mammary epithelial hTERT-HME1 cells. The pro-invasive effect is specifically attributed to T-MV since benign hTERT-MV did not influence tumour invasion. By blocking MV uptake into MCF-7 breast cancer cells with the endocytosis inhibitor dynasore, the pro-invasive effect of T-MV could be reduced by 40% showing that MV uptake is partly necessary for MV-induced tumour invasion. Additionally, we searched for possible candidate proteins which mediate the pro-invasive MV phenotype and found the MMP-inducer EMMPRIN enriched on T-MV. By shRNA-mediated knockdown of EMMPRIN in SK-BR-3 breast cancer cells, the pro-invasive potential of T-MV was significantly reduced. Since EMMPRIN is a highly glycosylated protein, we further investigated the influence of the EMMPRIN glycosylation status for MV function. Deglycosylation of T-MV by the N-glycosidase PNGaseF decreased MV-mediated breast cancer invasion in Boyden chamber assays. *Conclusions*: Breast cancer cells release T-MV which enhance invasion of their cell of origin as well as of the surrounding tumour cells. For the pro-invasive function of T-MV, an uptake into the recipient cell as well as expression of highly-glycosylated EMMPRIN on the T-MV surface are essential.

**Exosome from brain metastasis breast cancer cell promote the blood–brain barrier destruction**

N. Tominaga, N. Kosaka, M. Ono, H. Nakagama and T. Ochiya

National Cancer Center Research Institute, Japan

*Introduction*: Brain metastasis, which is an important cause of cancer morbidity and mortality, occurs in at least 30% of patients with breast cancer. A key event of brain metastasis is the migration of cancer cells through the blood–brain barrier (BBB), which constitutes the endothelium and the surrounding cells. However, these mechanisms are poorly understood. Meanwhile, there are abundant evidences that exosomes regulate cancer metastasis and drug resistance. Considering that tumour-derived exosomes travel to distant organs, we hypothesised that breast cancer cell-derived exosomes would play a role in brain metastasis. *Materials and methods*: MDA-MB-231-D3H2LN breast cancer cell line was injected intracardially to C.B-17/Icr-scid/scid female mice. When brain metastasis was found, the brain was extracted and the tumour cells were harvested from the metastatic foci. Following drug selection, the resulting cell populations (BMD1a) were subjected to the second round of *in vivo* selection yielding BMD2a and BMD2b cell populations. To assess the effect of exosome from BMD cell line, BBB model kit, which composes primary cultures of monkey (*Macaca irus*) brain capillary endothelial cells, brain pericytes and astrocytes, was employed. Using this kit, we investigated the association of the cancer derived-exosomes in the BBB breakdown and the invasiveness of the cancer cells. For exosome preparation, the conditioned medium from cancer cells was incubated in a non-serum medium for 2 days and ultracentrifuged at ~110,000 g for 70 min at 4°C. *Results and conclusions*: We found that the BMD cell line-derived exosomes broke down the BBB *in vitro* compared to the original cell line. Of note, non-invasive breast cancer cell line MDA-MB-231-D3H1 infiltrated brain parenchyma side of the *in vitro* BBB model kit when BMD cell line-derived exosomes were added to the culture medium. These results suggest that the breast cancer cell derived-exosomes promote brain metastasis.

**Exosomal transport of annexin A2 and its role in breast cancer microenvironment**

S. Maji, S.I. Thamake and J.K. Vishwanatha

University of North Texas Health Science Center, Fort Worth, TX, USA

*Introduction*: Exosomes secretion is higher in cancer when compared to normal cells. Deciphering the function of exosomes and the role of exosomal proteins in the tumour- microenvironment interaction may lead to new diagnostic and therapeutic approaches. Annexin A2 (AnxA2) is a Ca2-dependent phospholipid-binding protein, known to associate with the plasma membrane and the endosomal system. It is upregulated in cancer and implicated in many cancer-associated functions. Recent studies have found AnxA2 to be one of the most abundant proteins in the exosomes, but its function in the exosomes has not yet been fully studied. Our objective is to characterise exosomal AnxA2 and study its role in breast cancer microenvironment. *Materials and methods*: Exosomes isolated from conditioned media of MCF10A (non-malignant) and MCF10CA1A (malignant) were characterised by Western blotting and Nanotrac Analyzer, TEM and AFM to study the correlation between exosomal AnxA2 and the breast cancer progression. *In vitro* tube formation assay and *in vivo* matrigel plug assay were performed to study the role of exosomal AnxA2 in angiogenesis. *Results*: Successful isolation of exosomes was confirmed by TEM, and the presence of specific exosomal markers LAMP1, TSG101, CD81 and HSC70 was determined. Surface expression of exosomal AnxA2 was found to increase markedly in MCF10CA1A than MCF10A via AFM. Average size of the exosomes was found to be 61.7 nm, but no significant size difference was found between MCF10A and MCF10CA1A-derived exosomal population. Increased angiogenic potential was observed in the case of MCF10CA1A when compared to MCF10A-derived exosomes. *Conclusion*: These studies reveal increased surface expression of exosomal AnxA2 in cancer-derived exosomes when compared to normal. Furthermore, we found that exosomal AnxA2 is functionally active (MCF-10CA1A exosomal AnxA2 having more angiogenic potential than MCF-10A), indicating a possible role of exosomal AnxA2 in cancer progression.

**miRNA biogenesis and microparticles function**

F. Luk^1^, J. Gong^2^, S. Bajan^3^, G. Hutvagner^3^, G.E.R. Grau^4^ and M. Bebawy^1^

^1^School of Pharmacy, University of Technology, Sydney, Australia; ^2^School of Pathology, The University of Sydney, Sydney, Australia; ^3^Faculty of Engineering and Information Technology, Centre for Health Technologies, University of Technology, Sydney, Australia; ^4^Vascular Immunology Unit, Sydney Medical School, The University of Sydney, Sydney, Australia

*Introduction*: Microparticles (MPs) are membrane vesicles (0.1–1 µm) released from normal and malignant cells. The transfer of surface antigens, proteins and genetic materials by MPs has been identified as an important cell-to-cell communication mechanism. Previously, we have shown that MPs, which carry selectively packaged proteins, mRNA and miRNA, mediate the acquisition and dominance of donor cell traits in cancer. More recently, we demonstrated that MPs also contain mRNA of enzymes that are essential for miRNA biogenesis. This study was aimed to examine the role of miRNA biogenesis in regulating the donor cell trait dominance, mediated by MPs. *Materials and methods*: Realtime PCR is used to validate the presence of mRNA encoding P-glycoprotein (P-gp) and Argonaute 2 (Ago2) in MPs isolated from drug resistant human breast cancer cells (MCF-7/DX). Western blotting and confocal microscopy were used to detect the expression and localisation of these proteins in the cells prior to and following MP co-culture with drug sensitive recipient cells. *Results*: Expression of Ago2 mRNA was confirmed in MPs isolated from MCF7/DX cells. Increased Ago2 protein expression was identified in recipient cells following MP transfer. Immunofluorescence images have shown co-localisation of P-gp and Ago2 within the MP donor cells. *Conclusions*: Previously, we have shown that MPs with selectively packaged genetic materials and proteins were significant in mediating intercellular communication. The current study shows that MPs could aid the exchange of genetic message (miRNA), as well as transfer components of the mechanism that are responsible for gene regulation. These readily usable machineries facilitate a more efficient regulation towards the target cells. Furthermore, the co-localisation of P-gp and Ago2 indicates a close association between the resistance proteins and biogenesis machinery in the MP-mediated acquisition of multidrug resistance in cancer. This work was supported by NHMRC APP1007613.

**Priming metastatic niches: role of ECM signalling and exosome targeting**

Tang-Long Shen^1^, A. Hoshino^2^, D.C. Lyden^2^ and H.S. Peinado^2^

^1^National Taiwan University, Taipei, Taiwan; ^2^Weill Medical College, Cornell University, New York, NY, USA

Metastasis is a multistep systemic process in which tumour cells enable specifically targeting to and growing on pre-determined organs/tissues. However, it is little known how tumour cells educate “pre-metastatic niches” to permit the occurrence of metastasis. According to recent studies, tumour-derived exosomes play an important role in accomplishment of this process. We have found that several extracellular matrixes were up-regulated in the pre-metastatic phase of the lung in the tumour-bearing, orthotropic mouse model of melanoma and breast cancer. Complementarily, the corresponding integrins were present in the tumour-derived exosomes and tumour cells, which mediated the majority of exosome targeting and tumour metastasis onto the lung. To validate the role of the ECM–integrin interactions in the formation of pre-metastatic niches and subsequent metastasis, gene knockout and overexpression were employed to disrupt and promote the tumour progressive phenotypes, respectively, in orthotropic mouse models. Further studies will continue to examine the dynamic role of these ECM molecules on stroma remodelling in the lung, in association with tumour progression evoked by exosomes.

**Reversal of chemosensitivity and induction of cell malignancy of a non-malignant prostate cancer cell line upon extracellular vesicle exposure**

K. Panagopoulos, M. DelTatto, D. Pantazatos, D.R. Mills, J. Renzulli, P. Quesenberry and D. Chatterjee

Rhode Island Hospital, Providence, RI, USA

*Introduction*: Extracellular vesicle (ECV) trafficking is a fundamental cellular process that occurs in cells, and is required for many different aspects of pathophysiology. We report several phenotypic changes mediated by ECVs isolated from non-malignant and malignant prostate cells, as well as patient biopsied prostate tumour samples. *Materials and methods*: The human prostate carcinoma cell lines DU145 and RC1 cell lines were used. ECV isolation was conducted for prostate patient cancer tissue samples, as well as for the DU145 and PrEC cell lines. Non-malignant human PrECs and malignant DU145 prostate cells were co-cultured with ECVs from normal or malignant prostate tissue. Mass spectrometry analysis was performed by nano-LC-ESI-MS/MS using an Ultimate3000 nano-LC system. Ingenuity Pathway was used to identify protein networks according to biological functions and/or diseases. *Results*: RC1 undergo apoptosis when co-cultured with ECVs isolated from DU145 cells. DU145 cells become resistant to CPT when co-cultured with ECVs from RC1 cells. ECVs isolated from non-malignant PrEC cells can reverse soft agar colony formation of malignant DU145 cells, with the reciprocal effect also observed. Putative proteins were identified via antibody analysis that may be responsible for the phenotypic changes. Isolation of ECVs from 2 Gleason Grade 8 prostate cancer patients significantly induced soft agar colony formation of non-malignant PrEC cells. IPA analysis confirmed the presence of common proteins and pathways amongst the five patient samples. *Conclusions*: We can reverse the resistance of prostate cancer cells to CPT via ECVs. Growth in soft agar can be inhibited via ECVs isolated from non-malignant cells. Phosphoproteomic analysis revealed the transfer of proteins in our co-culture model. Our study provides the basis for examining proteins released by ECVs that are associated with disease progression and phenotype switching.

**Proteomic study of prostate cancer cell-derived extracellular microvesicles for the identification of potential therapeutic targets**

Gloria Polanco, E.A. Arigi, M.B. Cox and I.C. Almeida

University of Texas at El Paso, El Paso, TX, USA

*Introduction*: In prostate cancer, extracellular microvesicles (EMVs) have been shown to alter the tumour microenvironment by increasing motility and apoptotic resistance. It is, therefore, important to survey their content to identify potential players that can then be therapeutically targeted. The present study uses LC-MS/MS approaches to survey proteins present in EMVs derived from LNCaP prostate cancer cells, employing a protocol that minimises potential contaminants that could interfere with proteomic studies. *Materials and methods*: After eliminating cell debris, the cell supernatant was centrifuged for 2 hrs at 100,000xg to collect larger EMVs. The resulting supernatant was then centrifuged for 16 hrs at 100,000xg to collect smaller EMVs. EMV enrichment was confirmed by transmission electron microscopy and nanoparticle tracking analysis. EMV preparations were subjected to trypsin digestion and 1D LC-MS/MS analysis of the resulting peptides was conducted in a LTQ-XL ESI-linear ion trap-MS. Bioinformatic analysis was performed using Sorcerer-SEQUEST and Scaffold Software. *Results*: We identified 298 proteins in two biological replicates, with five technical replicates each. Amongst the proteins identified were ADAM10 (key for cell proliferation and migration), apoptosis inhibitor programmed cell death 6-interacting protein isoform 2 and several components relevant for androgen receptor regulation (which is important for prostate cancer cell survival and proliferation), including α-catenin, β-catenin, FKBP52, HSP70 and HSP90. *Conclusion*: Using a EMV enrichment methodology that minimises protein contaminants, as well as 1D LC-MS/MS, we were able to identify proteins important for cancer-cell migration, survival, proliferation and activation of the androgen receptor pathway. Future directions include quantitative proteomic analysis, as well as functional studies to determine the role of these cargo proteins in prostate cancer.

**Heparan sulphate proteoglycan side chains are essential for exosome-induced differentiation of cancer-associated stromal cells**

J.P. Webber, M.D. Mason, Z. Tabi and A. Clayton

Cardiff University, Cardiff, UK

*Introduction*: Aberrantly altered stromal myofibroblasts surrounding carcinomas promote angiogenesis and tumour progression. TGFβ is thought to direct differentiation of normal stroma to this disease-promoting myofibroblastic phenotype. Cancer exosomes often exhibit TGFβ-tethered through the heparan sulphate proteoglycan Betaglycan to their surface. Here, we compare the effects of exosomal and soluble TGFβ (sTGFβ) on stromal differentiation and stroma-assisted tumour growth. *Materials and methods*: Du145 prostate cancer exosomes, purified by sucrose cushion, were analysed by flow cytometry, nanoparticle tracking and western blot. Patient-matched normal or tumour-associated stromal cells were obtained from prostatectomy specimens. Myofibroblast differentiation was determined by the onset of α-smooth muscle actin (αSMA) expression, and growth factor secretion was measured by ELISA. Angiogenic function was assessed by stroma co-culture with HUVECs. *In vivo* assessment of stromal-assisted tumour growth involved a sub-cutaneous xenotransplantation murine model. *Results*: αSMA expression was triggered in normal stroma, by exosomes or sTGFβ in a comparable manner, but exosomes were unique in triggering pro-angiogenic factors. This correlated with the capacity of stroma to organise HUVEC into vessel-like structures *in vitro* and support tumour growth *in vivo*. Rab27a knockdown attenuated Du145 exosome secretion and subsequent tumour–stroma interactions, failing to induce stromal differentiation or promote tumour growth *in vivo*. Digestion of exosomal heparan sulphate side chains did not alter exosomal-TGFβ levels, yet abrogated exosome-mediated differentiation. *Conclusion*: Exosome delivery of TGFβ requires co-reception, through heparan sulphate proteoglycans such as Betaglycan. This provides a qualitatively distinct delivery of TGFβ, resulting in stromal differentiation to a disease-promoting myofibroblast phenotype.

**GIST exosomes mediated transformation: role in tumour spread**

Safinur Atay^1^, J. Crow^2^, L. Rink^3^ and A.K. Godwin^2^

^1^University of Kansas Medical Center, Kansas City, KS, USA; ^2^Department of Pathology and Laboratory Medicine, University of Kansas Medical Center, Kansas City, KS, USA; ^3^Developmental Therapeutics Program, Fox Chase Cancer Center, Philadelphia, PA, USA

*Introduction*: GISTs (Gastrointestinal Stromal Tumours) are the most common mesenchymal tumours of the digestive tract, but their molecular aetiology and cellular origin are unknown. Most adult GISTs are known to be “driven” by gain-of-function mutations in KIT, and to a lesser extend PDGFRA. GISTs are believed to arise from the Interstitial Cells of Cajal (ICC), the pacemaker cells of the gastrointestinal tract. The similarity between GISTs and ICCs is further borne out by the expression of the KIT protein (also called CD117) in non-neoplastic ICC and most GISTs. ICCs also require the KIT kinase and its ligand stem cell factor (SCF) for their development. Several studies have shown that tumour cells produce and utilise nanosized vesicles, called exosomes, transporting various cargo reflective of the cells of origin, to communicate with and alter the surrounding microenvironment. In this study, we demonstrate for the first time that GIST-derived exosomes can induce a GIST-like phenotype in human smooth muscle cells (ULTR) via the transfer of mutant KIT. *Method of study*: Conditioned media from ULTR and GIST-T1 cells were used to purify exosomes by ultracentifugation and sucrose gradient. Expression of exosomal markers, p-KIT (Tyr 719) and total KIT on exosomal and cellular lysates were analysed by western blot and flow cytometry. Uptake experiments were performed by adding PKH67-labelled T1-derived exosomes (TEX) to ULTR cells in culture and analysed by flow cytometry. Activation of ERK1/2 pathway and induction of ICC-associated markers were determined after co-culture.

**Evaluation of the roles of nanometer sized extracellular vesicles on lung cancer progression**

W.L. Huang1, C.C. Lin^2^ and W.C. Su^2^

^1^Institute of Oral Medicine, National Cheng Kung University College of Medicine and Hospital, Tainan, Taiwan; ^2^Department of Internal Medicine, National Cheng Kung University College of Medicine and Hospital, Tainan, Taiwan

*Introduction*: Recently, nanometer sized extracellular vesicles (EVs) were shown to play important roles in many physiological and pathological processes, including tumour. However, the contribution of EVs in lung cancer progression is still largely unknown. In this study, we investigated the roles of EVs in lung cancer using a malignant pleural effusion (MPE) model, in which soluble components play important roles. *Materials and methods*: EVs were isolated using both ultra-centrifugation (UC) and ultra-filtration (UF) methods, and evaluated by TEM, Nanosight and Western blotting. IL-6 and VEGF contents in EVs were evaluated by ELISA. The effects of EVs on cell signalling and vascular permeability were evaluated by Western blotting and Miles assay, respectively. *Results*: The UF method was shown to be ideal for rapidly isolating urine EVs, which can be carried out by regular centrifuges instead of ultra-centrifuges fitting better for clinical needs. However, it has not been tested using tumour samples. We showed that the UF method is comparable to the classical UC method for isolating EVs from both cell culture and clinical lung cancer samples. TEM and Nanosight revealed that EVs isolated using both methods were closed vesicles of nanometer size. Specific markers including Alix, CD63 and Tsg101 were detected. We further showed that lung cancer cells could uptake EVs and trigger oncogenic signals, such as Stat3 and Akt. We have previously shown that IL-6/Stat3/VEGF pathway plays an important role in cancer metastasis. Here, we showed that EVs from culture supernatants and clinical MPE samples carried high level of IL-6 and VEGF and triggered vascular permeability changes. *Conclusions*: These results demonstrated that the UF method is ideal for isolating tumour-associated EVs from both cell culture and clinical samples. In addition, lung cancer-associated EVs may contribute to cancer progression by triggering oncogenic signals with the IL-6 and VEGF cargos.

**Active protein kinase A is secreted on tumour exosomes**

V. Porcari^1^, M. Kasari^2^, E. Lari^1^, G. Radano^1^, P. Guazzi^3^, C. Fondelli^1^, A. Chiesi^3^ and N. Zarovni^1^

^1^Exosomics Siena SpA, Siena, Italy; ^2^Kinasera OÜ, Tartu, Estonia; ^3^Hansabiomed OU, Estonia

*Introduction*: Aberrant protein kinase (PK) activity in cancer is a potential therapeutic and diagnostic target. Tumour cells secrete the catalytic subunit of PKA (PKAc), causing multifold increase in blood level of PKAc. Though extracellular PKA was proposed as generic cancer marker, its use in screening and monitoring of cancer has been hampered by the lack of tools for its reliable detection in complex biological samples. The use of novel ARC-Lum probe for measurement of active PKAc in plasma was impeded by the protein release from damaged or activated platelets. We used ARC-Lum probe to assess the presence of PKA on exosomes from tumour cells *in vitro* and from healthy donors and cancer patients, to assess if targeting exosomes rather than soluble PKs improve the signal to noise ratio. *Materials and methods*: Exosomes were purified by ultracentrifugation or immunocapture from cell supernatants and plasma samples. PKAc on exosomes was assessed by WB, FACS and ELISA. Binding of ARC-1139 to exosomes was assessed in a TRF assay. Sensitivity and specificity of the probe was determined by titration of recombinant human PKAc, and by addition of the PKA activator cAMP and specific inhibitor PKI. *Results*: We reported the association of active PKA to tumour exosomes. This otherwise cytosolic protein was displayed on the vesicle surface and maintained its activity on circulating exosomes. TRF assay showed fine linearity between TRF signal and exosome amount. Observed exosomal PK levels varied in different individual plasma samples and appeared dependent on exosome integrity and assay protocol. *Conclusion*: Active PKA is displayed on exosomes derived from tumour cells while scarcely detectable or absent on normal circulating exosomes. The protein is also detected on exosomes from samples obtained from melanoma patients using ARC-1139 probe-based TRF assay. Real potential for accurate resolution between patients and control samples needs to be addressed in enlarged samples set.

**VEGF-dependent angiogenic activity of extracellular vesicles isolated from mouse melanoma tissue**

E.J. Choi^1^, Y.J. Yoon^2^, S.R. Kim^1^, S.C. Jang^1^, B.S. Hong^1^, J.H. Kim^1^, J. Lötvall^3^, Y.K. Kim^1^ and Y.S. Gho^1^

^1^Department of Life Science, Pohang University of Science and Technology, Republic of Korea; ^2^School of Interdisciplinary Bioscience, Pohang University of Science and Technology, Republic of Korea; ^3^Department of Internal Medicine, Krefting Research Centre, University of Gothenburg, Gothenburg, Sweden

*Introduction*: Various mammalian cells, including cancer cells, shed extracellular vesicles (EVs) into the surrounding tissues. Although mammalian EVs play multiple roles in the tumour growth and metastasis by promoting angiogenesis, the biological functions of tumour tissue-derived EVs *in vivo* have never been investigated. Here, we isolated and characterised the mouse melanoma tissue-derived EVs, and investigated their roles in neovascularisation. *Materials and methods*: EVs were isolated from mouse melanoma tissues by serial centrifugation and density gradient ultracentrifugation. These purified EVs were analysed by electron microscopy, dynamic light scattering and Western blotting analyses. Mouse Matrigel plug assay was carried out to examine the pro-angiogenic activity of the EVs. *Results*: Isolated EVs from mouse melanoma tissues were closed lipid-bilayered vesicular forms with ~150 nm size in diameter. These EVs were enriched with vesicular marker proteins (CD9, CD81 and Tsg101), tyrosinase, F4/80, alpha-smooth muscle actin and E-selectin, suggesting that melanoma cell-, macrophage-, fibroblast- and endothelial cell-derived EVs are present in the melanoma microenvironment. When tumour-derived EVs within Matrigel were injected subcutaneously into mice, a massive formation of CD31-positive vessel-like structures and infiltration of F4/80-positive macrophages were observed. We observed that EVs harboured VEGF and significant amount of VEGF was detected in EV-treated Matrigel. Moreover, SU5416 (a VEGF inhibitor) almost completely blocked EV-induced macrophage infiltration and angiogenesis. *Conclusions*: This study showed that both cancer cell- and stromal cell-derived EVs were present in the tumour microenvironment. These tumour tissue-derived EVs have VEGF-dependent angiogenic activities. Further studies should be warranted to decipher the molecular mechanisms involved in the role(s) of EVs in tumour growth, angiogenesis and metastasis.

**Ovarian cancer cell-secreted exosomes induce molecular and phenotypic changes in cells**

Vanessa Enriquez^1^, J.C. da Silveria^1^, M.A. Spillman^2^, Q.A. Winger^1^ and G.J. Bouma^1^

^1^Colorado State University, Fort Collins, CO, USA; ^2^University of Colorado Denver, CO, USA

Ovarian cancer is the fifth most deadliest cancer amongst women in the United States, and the most lethal gynaecological malignancy in the world. Recent studies reveal that human tumour cells release cell-secreted vesicles called exosomes. Exosomes are endosome-derived vesicles containing bioactive materials, including miRNAs that can be detected in the bloodstream and urine. Importantly, stem cell factor LIN28, a regulator of let-7 miRNAs, is present in ovarian cancer cells. Our preliminary data revealed a potential regulatory role of LIN28-let-7 miRNA in ovarian cancer cells, that may play a role in cancer metastasis via their secreted exosomes. We hypothesised that ovarian cancer cell-secreted exosomes are taken up by target cells and induce changes in gene expression and cell behavior. Our objectives were to: (a) determine the effects of IGROV1 cell-secreted exosomes on HEK293 cells, (b) identify genes related to the epithelial to mesenchymal transition (EMT) pathway, that are modulated in HEK293 cells following exposure to IGROV1-secreted exosomes and (c) identify miRNAs present in IGROV1-secreted exosomes, that are predicted to target genes involved in EMT. Our data revealed that IGROV1-secreted exosomes are taken up by HEK293 cells. In addition, HEK293 cells treated with IGROV1-secreted exosomes had increased levels of LIN28, and demonstrated increased invasion and migration (p<0.04). Finally, various genes involved in EMT, including TIMP1 (25-fold higher), FOXC and NOTCH1 (11-foldhigher), CDH1 (6-fold higher), MMP2 (5-fold higher), MMP9 (4-fold higher) and ZEB1 (3-fold higher) were up-regulated in HEK293 cells, that have taken up IGROV1-secreted exosomes. Elucidating the molecular and phenotypic effects that ovarian cancer cell-secreted exosomes have on non-cancerous cells, will lead to greater understanding and insight into cancer metastasis and tumour development.

**Prostate cancer derived exosomes could promote prostate cancer progression via activation of the ERK cell signalling pathway**

Elham Hosseini Beheshti, C. Liu, Tomlinson Gao and E.S. Guns

The Vancouver Prostate Centre (UBC), Vancouver, Canada

*Introduction*: Prostate cancer (PCa) is the leading diagnosed and third most lethal malignancy in men. During early stages of the disease, when confined to the prostate, it is androgen-dependent, and yet curable by surgical intervention or radiation treatment. Cancer which has metastasised may be treated using hormone withdrawal therapy; however, over time many PCas invariably became resistant to treatment and progresed to Castration Resistant Prostate Cancer (CRPC), which ultimately results in death. While numerous treatment resistance mechanisms have already been identified, understanding factors contributing to CRPC continues to be an important research focus. Exosomes are nanometer sized cup-shaped membrane vesicles which are secreted from normal and cancerous cells. Exosomes could play a significant role in paracrine signalling pathways, thus potentially influencing cancer progression. We explored the ERK pathway in exosomes since it has the potential to influence a wide variety of cell functions, as diverse as cell proliferation to apoptosis. *Methods*: Exosomes were purified from the conditioned media of different prostate cell lines using differential centrifugation, followed by an ultracentrifugation step in 30% sucrose. Further analysis using Western blot (WB) and transmission electron microscopy validated the purity and integrity of isolated exosomes. Alterations in the ERK cell signalling pathway in recipient cells were then studied, upon exposure to exosomes derived from a panel of prostate cell lines. *Results*: Our WB data confirmed a pure enrichment of exosome markers in the exosome isolate. WB data also indicates that exosomes derived from PCa cell lines will activate ERK phosphorylation and subsequent signalling via Ras/Raf/MEK pathway. *Conclusion*: The Ras/Raf/MEK/ERK signalling pathway may be activated in a panel of prostate cells by exosomes derived from PCa cell lines. Exosomes could, therefore, influence signalling pathways involved in PCa progression.

**Role of interleukin 8 in exosome-mediated crosstalk between chronic myelogenous leukaemia cells and bone marrow stromal cells**

C. Corrado, S. Raimondo, A. Flugy, G. De Leo and R. Alessandro

Universita degli studi di Palermo, Italy

*Introduction*: Chronic myelogenous leukaemia (CML) is a myeloproliferative disorder characterised by t(9:22) (q34:q11) reciprocal translocation, resulting in the expression of chimeric Bcr–Abl oncoprotein with constitutive tyrosine kinase activity. Exosomes (Exo) are small vesicles of 40–100 nm in diameter released by many cell types including cancer cells that have a role in cell-to-cell communication and tumour–stroma interaction, thus potentially affecting cancer progression. It is well known that stromal microenvironment contributes to disease progression through the establishment of a bi-directional crosstalk with cancer cells. In bone marrow, stromal cells are able to sustain the growth and survival of leukemic cells by protecting malignant cells from chemotherapy-induced death; contrarily, leukaemia cells induce changes in the bone marrow architecture. Our hypothesis is that exosomes could have a functional role in this crosstalk. *Materials and methods*: Cell lines used in experiments are LAMA84, a human CML cell line, and HS5, a bone marrow derived human stromal cell line. Quantitative gene expression analysis for IL8, IL6 and VEGF was performed by TaqMan RT-PCR; colony formation assay was performed with methylcellulose; adhesion assay was performed with LAMA84 and HS5 cell lines; Western blot analysis was performed with antibodies anti-Akt, anti-pAkt, anti-Erk 1/2 and anti-p-Erk 1/2. *Results*: Treatment of HS5 cells with LAMA84-derived Exo induced a significant increase of IL8 as well as LAMA84 cell adhesion to stromal monolayer. For these reasons, we decide to treat CML cells with recombinant IL8 (rIL8). Addition of rIL8 to LAMA84 activates survival pathways and increases the adhesion of leukemic cells to stroma. The inhibition of IL8 receptors, CXCR1 and CXCR2, on LAMA84 cells revert the effects described previously. *Conclusions*: Our data show that LAMA84-derived Exo modulate bone marrow microenvironment, increasing the production of the IL8 on stromal cells; moreover IL8 is able to affect leukaemia cell proliferation and survival in a paracrine fashion.

**Characterisation of extracellular vesicles in chronic lymphocytic leukaemia**

Franziska Haderk, P. Lichter and M. Seiffert

German Cancer Research Center DKFZ, Germany

*Introduction*: The pathogenesis of chronic lymphocytic leukaemia (CLL) is stringently associated with a tumour-supportive microenvironment. Of note, CLL cells themselves initiate promotive responses in surrounding cells, and Extracellular Vesicles (EVs) released by CLL cells represent a newly discovered mechanism of cell communication. Thus, we aim to characterise EVs present in blood plasma of CLL patients and released by primary CLL cells in culture in order to understand their role within microenvironment. *Material and methods*: EVs were isolated from blood plasma of CLL patients and healthy donors and also from supernatant of primary CLL cells in culture. Purification of EVs was performed by serial centrifugation and density-based separation. RNA and protein lysates of EVs and respective cells of origin were analysed via Bioanalyser profiling and quantitative RT-PCR or via Coomassie-staining of SDS-PAGE gels and mass spectrometry, respectively. *Results*: We were able to isolate EVs from blood plasma of CLL patients and healthy controls. Protein profiling comparing lysates of EV and peripheral blood mononuclear cells from same donor revealed an EV-specific protein profile. Proteins present in EVs include fibronectin precursor, CD36, MHC class I proteins and heat shock proteins. In order to characterise CLL-cell derived EVs, we established different culture systems of CLL cells, which mimic microenvironmental niches in CLL. Analyses regarding the amount of EVs produced as well as the RNA and protein composition are currently conducted. Of note, first results in Mec1 cells, a CLL cell line, revealed an enrichment of small RNAs and shuttling of microRNA-155 in EVs. *Conclusion*: CLL cell-derived EVs might be involved in the establishment of a promotive microenvironment. Thus, their characterisation will help to identify EV-associated molecules involved in CLL pathogenesis, with the final goal to develop new therapeutic options for CLL.

**Microvesicles and epithelial mesenchymal transition in the development of cancer**

A. Haidery^1^ and J.M. Inal^2^

^1^London Metropolitan University, London, UK; ^2^Cellular and Molecular Immunology Research Centre, London Metropolitan University, London, UK

*Introduction*: Microvesicles released by tumour cell lines are thought to play an important role in both extracellular matrix (ECM) invasion and the evasion of immune system. We have examined the role of MVs derived from a T cell line (Jurkat) on PNT2 cells (normal prostate cells) to see if they carry some bioactive molecules capable of inducing an epithelial to mesenchymal transition (EMT). *Materials and methods*: PNT2 cells (4x103) were seeded for 14hr in 12-well plates and cultured in complete growth medium. Cells were washed twice with RPMI and treated with 30 µg/ml of Jurkat cell-derived MVs (37°C for 5 days). Changes in morphology and in expression of vimentin and E-cadherin by flow cytometry using the Guava EasyCyte and immunofluorescence microscopy (Olympus IX81 inverted microscope) were noted. *Results*: By microscopic analysis, PNT2 cells, which are of epithelial origin, treated with Jurkat MVs, showed some morphological changes. They became elongated, motile and morphologically mesenchymal-like as previously documented. The molecular changes were confirmed by immunohistochemical techniques using the fluorescencent microscope and flow cytometer. The experimental (MV-treated cells) compared to control (untreated cells) expressed high levels of mesenchymal markers such as vimentin and low levels of epithelial markers such as E-Cadherin. Proteins from PNT2 control cells and MV-treated cells were profiled by SDS-PAGE for identification by mass spectrometry. *Conclusions*: The correlation between EMT and malignancy has been well documented in almost all carcinomas of epithelial origin. EMT basically explains how epithelial tumour cells can escape from primary residence, travel to distant sites and establish secondary tumours? Our work is the first report of EMT on normal prostate cell lines induced by microvesicles.

**Role of microRNAs shuttled by exosomes in the crosstalk between chronic myelogenous leukaemia and endothelial cells**

Riccardo Alessandro, S. Taverna, L. Saieva, A. Flugy and G. De Leo

University of Palermo, Italy

*Introduction*: Exosomes are membranous nanovesicles derived from endosomal membrane compartments that are released from cell surface following activation or stimuli. Exosomes contain proteins, mRNA and microRNAs (miRNAs) and function as mediators in cell-to-cell communication. Exosomes can be transported within different cells thus affecting phenotypes of the recipient cells. A number of studies have described exosomes as new players in modulating tumour microenvironment, promoting angiogenesis and tumour development. Neovascularisation is known to exert an important role in the progression of chronic myelogenous leukaemia (CML). CML is a myeloprolifetive disorder characterised by the presence of Philadelphia chromosome. This rearrangement results in the production of chimeric bcr-abl oncoprotein with a constitutive tyrosine kinase activity. Our previous work showed that CML exosomes were involved in modulating angiogenesis, and other work in a different cancer histotype indicated that hypoxia triggers a pro-angiogenic pathway mediated by the release of exosomes. Because, exosomes are involved in the horizontal transfer of information, through the export of miRNAs, we focus on the role of miRNAs isolated from CML exosomes in angiogenesis. *Material and methods*: Exosomes were collected from LAMA84 conditioned medium by different centrifugation steps. The miRNAs found in exosomes were characterised by miRNA-array and RT-PCR validation. HUVEC were treated with exosomes for 6–24 h, analysed for expression of different genes with RT-PCR and evaluated for protein secretion by ELISA. *Results*: Our preliminary data indicated that CML exosomes shuttle miRNAs involved in angiogenesis, we focused on miRNA 22, 126, 150 and 886. We also observed that the treatment of endothelial cells with CML-exosomes down-regulate CXCL12 gene expression, and we are currently investigating whether miRNA 126 and 886, targeting CXCL12, are involved in this process. *Conclusion*: CML-derived exosomes contain miRNAs that can target genes involved in angiogenesis.

**Effect of tumour cell-derived exosomes on immune- and mesenchymal stem cells**

M. Harmati^1^, A. Marton^1^, R.L. Katona^1^, E. Gyukity-Sebestyen^1^, E. Kusz^1^, Z. Tarnai^2^, E. Duda^1^, J. Minarovits^2^, K. Nagy^2^, I. Nagy^1^, B. Horvath^1^, C. Vizler^1^ and K. Buzas^3^

^1^Hungarian Academy of Sciences, Biological Research Centre, Hungary; ^2^Faculty of Dentistry, University of Szeged, Hungary; ^3^Hungarian Academy of Sciences, Biological Research Centre; Faculty of Dentistry, University of Szeged, Hungary

*Introduction*: Malignant melanoma (MM) is an especially aggressive skin cancer. The mechanism of its rapid metastasis formation, high genetic variability and effective immune escape mechanisms are not explained yet. Exosomes are among the potential mediators of communication between melanoma cells and their environment. To explore the interaction of melanoma cell derived exosomes (mcde) and their microenvironment, we investigated their effect of mesenchymal stem cells (MSC). *Material and methods*: Mouse MSCs from adipose tissue were pre-treated with B16 mouse melanoma cell derived exosomes, then the rate of spontaneous apoptosis and the expression of multipotent stromal cell markers were analysed by flow cytofluorometry. *Results*: We have found marked differences in the expression CD44, integrin alpha 4, CD29 and CD106, 7 days after induction. The ratio of late apoptotic or necrotic/early apoptototic cells decreased after mcde treatment (0.43/1 versus 0.98/1). We sequenced the small RNA content of melanoma cell derived exosomes by SOLiD 5500xl technology, and the sequences were annotated in CLC Genomics Workbench version 5.5.1. We have found the highest expression values of mir205, mir31, mir21a and mir15b. To explore the potential in vivo tumour-promoting effect of exosomes, B16F1 tumour-bearing mice were injected with mcde-induced-MSCs i.v., the control mice received untreated MSC. The survival rate at day 42 was 38% after pre-conditioned MSC treatment versus 85% un-induced MSCs. *Conclusions*: Our data suggest that melanoma cell derived exosomes re-educate mesenchymal stem cells through their miRNA cargo, giving rise to a cell population that support metastasis formation. Supported by OTKA PD 84064, TÁMOP-4.2.2/B-10/1-2010-0012, TÁMOP-4.2.2. A-11/1/KONV-2012-0025.

**Association of exosome secretion and drug resistance in aggressive B-cell lymphomas**

Thiha Aung^1^, D. Vogel^2^, R. Koch^2^, K. Menck^2^, S.I.A. Mohring^2^, B. Chapuy^3^, L. Truemper^2^ and G.G. Wulf^2^

^1^University Medicine Goettingen, USA; ^2^University Medicine Goettingen, Germany; ^3^Dana Farber Cancer Institute, USA

*Introduction*: Although, significant proportions of patients with aggressive B-cell lymphoma are cured by R-CHOP immunochemotherapy, tumour cell susceptibility to chemotherapy varies with mostly fatal outcome in cases of resistant disease. Here, we analysed the clearance of chemotherapy associated with exosome release and ABCA3 expression. *Material and methods*: Diffuse large B-cell lymphoma (DLBCL) cell lines SuDHL4, Balm3 and OCI Ly1 were pulsed with doxorubicin, vincristine and vinblastine in vitro, washed twice and incubated in exosome-free medium for 24hr. Lymphoma derived exosomes were analysed by HPLC measurement and ABC transporter A3 (ABCA3) expression. The combination treatment of chemotherapy and the pharmacological blockade of exosome formation (indomethacine, rapamycin and U18666A) and the silencing of ABCA3 by using short hairpin-RNA (shRNA) viability were measured by MTT. *Results*: Using HPLC measurement, we were able to detect different amounts of doxorubicin, vincristine and vinblastine in exosomes and cell culture supernatants. Several mechanisms have been described to interfere with cellular exosome secretion in different biological systems, ranging from agents perturbing MVB biogenesis, such as rapamycin, to agents perturbing membrane cholesterol supply, such as U18666A and indomethacin. We confirmed significant inhibitions of exosome release occurring at nontoxic concentrations of the drugs. Diminished exosome release was associated with increased efficacy of immunochemotherapy. Also, silencing of ABCA3 by lentiviral shRNA constructs reduced exosome release from the cell line models Balm-3, Su-DHL-4 and OCI-Ly1. Concordantly, silencing ABCA3 also increased the susceptibility of lymphoma cells to immunochemotherapy. *Conclusion*: We found that exosomes participates in resistance to chemotherapy. Both pharmacological blockade and silencing of ABCA3 enhanced the susceptibility of target cells to cytotoxic agents.

**Exosome-mediated wnt-signalling augments side population in aggressive lymphoma**

Raphael Koch^1^, M. Demant^1^, T. Aung^1^, A. Guentsch^1^, N. Diering^1^, B. Chapuy^2^, L. Truemper^1^ and G.G. Wulf^1^

^1^University Medicine Goettingen, Germany; ^2^Dana Farber Cancer Institute, USA

*Introduction*: Patients with aggressive B-cell lymphoma are treated in curative intention. However, some patients experience fatal relapse originating from refractory lymphoma cells with the capacity for clonogenic regrowth. Here, we addressed repopulation capacity of lymphoma cell subpopulations and the mechanisms regulating the population composition in growing tumour. *Material and methods*: We identified side population (SP) cells in diffuse large B-cell lymphoma cell lines and patient samples with DNA-binding dye Hoechst33342, analysed clonogenicity in vitro and in vivo and screened for differentially expressed genes and DNA-methylation patterns. A GFP-containing lentiviral vector construct was used to keep track of side population cells cultured among mixed cultures of SP and non-SP cells. Manipulation of canonical wnt-signalling was performed by lentiviral sh-RNA constructs and pharmacological tankyrase-inhibition by XAV-939. In vitro, data were supported by in vivo experiments using a chorioallantoic membrane-assay. *Results*: Colony assays and suspension cultures of sorted SP and non-SP cells revealed the restriction of clonogenic potential to SP cell population and the resurgence of non-SP cells from purified SP cell progenitors, while mixed culture assays using a GFP-vector construct tracing SP- vs. non-SP-population revealed the homeostasis between two populations, showing both SP and non-SP cells contributing to either cell compartment. SP cells show enhanced canonical wnt-signalling and increased exosomal secretion of wnt3a. Suppression of canonical wnt-signalling resulted in reduced clonogenicity. Exosome stimulation of DLBCL cell lines resulted in increased clonogenicity, stabilisation of beta catenin and enhanced TOP/FOP activity. *Conclusion*: Here we show that tumour cells reversibly switch between states of autonomous and non-autonomous clonogenicity and such transitions are regulated by exosome-mediated wnt signalling.

**Glioblastoma transfer miRNAs into microglial cells through extracellular vesicles**

E.R. Abels^1^, K.E. van der Vos^1^, J. Skog^2^, S.E. Hickman^1^, J. El Khoury^1^ and X.O. Breakefield^1^

^1^Massachusetts General Hospital, USA; ^2^Exosome Diagnostics, Inc., USA

*Introduction*: Extensive evidence indicates that tumours can change the phenotype of normal cells to their advantage. To understand the ability of glioblastoma multiforme (GBM) to manipulate their microenvironment we focus on the interaction of tumour-released extracellular vesicles (EVs) with microglia. *Material and methods*: To determine the expression levels of miRNAs in GBM-derived EVs, EVs from primary human GBM cells were isolated, and levels of miRNAs were analysed and compared to cellular levels using a miRNA expression-profiling panel (Illumina). The uptake of EVs was analysed in a Transwell co-culture system with 1 µm pores. Fluorescently labelled primary GBM cells, shedding EVs, and mouse microglial cell lines, KW3 and N9, were plated in upper and bottom wells respectively. In addition, EVs isolated from GBM cells by differential ultracentrifugation were added to microglia. The transfer of miRNAs was analysed with Taqman assay, 24 and 48 h after exposure of microglia to GBM EVs. *Results*: Microglia co-cultured with fluorescently labelled GBM cells showed increased levels in fluorescence indicating the uptake of EVs. After 24 h, 50% of the cells had taken up EVs, increasing up to 80% after 48 hr. Internalisation of labelled EV was imaged with fluorescence microscope. The level of miR451 in microglia was monitored after exposure to GBM-EVs and showed up to 15-fold increased levels after 24 and 48 h of exposure as compared to untreated microglia. We are currently assessing changes in levels of mRNAs targeted by miR451 in microglia. *Conclusions*: This study supports a role for EVs in intercellular communication in which microglia are able to take up EVs shed by GBM cells. Moreover, the transfer of miR451 from GBM cells to microglia is mediated by EVs. Future experiments will focus on physiologic effect of the EV-mediated transfer of miR451 in microglia, which should give insight into how GBM-derived EVs can alter tumour microenvironment.

**Potential roles of osteosarcoma-derived exosomes in pre-metastatic niche formation in the lung**

Takeshi Katsuda, T. Fujiwara, N. Kosaka, A. Kawai and T. Ochiya

National Cancer Center Research Institute, Japan

*Introduction*: Lung metastasis significantly deteriorates the prognosis in osteosarcoma (OS). However, the mechanism regulating lung metastasis is poorly understood and thus, any possible mechanism should be investigated. Recent studies have shown the involvement of tumour-derived exosomes in the progression of malignancy. Here, we investigated the role of OS-derived exosomes in lung metastasis. *Materials and methods*: We used a highly metastatic human OS cell line 143B cells expressing firefly luciferase and established a derivative cell line with shRNA knockdown of neutral sphyngomyelinase 2 (143B-KD-nSMase2), an important regulator for exosome secretion. The 143B cell-derived exosomes were isolated by ultracentrifigation. Original 143B cells were orthotopically transplanted to the right tibia of nude mice at 1.5 106 cells/mouse (group 1). Mice similarly transplanted with 143B-KD-nSMase2 cells were divided into 2 groups and were intravenously administered 200 µL PBS (group 2) or 5 µg-exosomes/200 µL PBS (group 3) twice a week for 3 weeks. Lung metastasis was monitored by IVIS system. *Results*: Three weeks after orthotopic transplantation, lung metastasis was observed in 7/10 mice in group 1. In contrast, only 3/10 mice showed lung metastasis in group 2, indicating the decreased metastasis following the inhibition of exosome secretion. Remarkably, however, 7/10 mice showed metastasis in group 3, indicating that systemically administered exosomes restored metastatic ability. Accordingly, the histology of lungs confirmed that the number of metastatic foci were dramatically reduced in group 2 compared with group 1, whereas that in group 3 was comparable to group 1. *Conclusion*: The results demonstrated that exosomes secreted by 143B cells into the circulation promoted lung metastasis. Given the accessibility of exosomes to distant organs, we hypothesize the roles of exosomes in pre-metastatic niche formation in the lung and are now exploring the underlying molecular mechanism.

**Cellular senescence increases release of α5β1 and α2β1 integrin-enriched microvesicles that reduce cell migration and invasion of MDA-MB-231 cells**

P.V.E. van den Berghe, J. Hernandez, P. Timpson, A. Ivanov, S. Zanivan, P.D. Adams and J. Norman

Beatson Institute for Cancer Research, UK

Cellular senescence is an irreversible proliferation arrest that can be evoked by stresses such as DNA damage, excess rounds of replication or oncogenes. Senescent cells influence their surrounding stroma through an active secretory program, which, for example, has been shown to recruit immune cells to clear senescent cells. We have found that upon induction of senescence (either by excess replication, or expression of oncogenic Ras or Raf mutants), fibroblasts released 5-fold more microvesicles than replicating cells. Senescent cell-derived microvesicles floated at a buoyant-density of 1.10–1.15 g/ml on a sucrose-density gradient and electron microscopy revealed that they have a mean diameter of approximately 150–200nm, indicating that they are on average rather larger than the exosomes originating from multi-vesicular bodies. Furthermore, mass-spec analysis revealed that senescent cells sort 100-fold more β1 integrins into these microvesicles by comparing with replicating cells, and the integrins in senescent microvesicles are in an active conformation and competent to bind ligands such as fibronectin. These microvesicles can be efficiently internalised by MDA-MB-231 breast cancer cells. However, microvesicle uptake by MDA-MB-231 cells was strongly reduced when we exposed microvesicles to a 3D cell-derived matrix prior to uptake, suggesting that integrin-matrix interactions hamper microvesicle uptake. The presence of senescent microvesicles on the 3D cell-derived matrix reduced the ability of these cells to move in a persistent fashion. Furthermore, invasion of MDA-MB-231 cells in a 3D-collagen matrix was reduced in the presence of senescent microvesicles. Taken together, these data indicate that senescent cells can release integrin-rich microvesicles which alter the way in which cancer cells migrate through extra-cellular matrix.

**Regulated release of macrophage-derived matrix vesicles from lipid raft domains: a role for formation of microcalcifications in vulnerable plaques**

J.D. Hutcheson^1^, S.E.P. New^2^, C. Goettsch^1^, M.A. Rogers^1^ and E. Aikawa^1^

^1^Center for Interdisciplinary Cardiovascular Sciences, Harvard Medical School, Brigham and Women's Hospital, Boston, MA, USA; ^2^University College of London, London, UK

*Introduction*: We recently demonstrated that macrophage-derived matrix vesicles (MVs) are correlated with the formation of microcalcifications within the fibrous cap of atherosclerotic plaques. Aggregation of MVs is hypothesised to lead to growth of microcalcifications, contributing to plaque vulnerability and rupture, and resulting in more than 50% of cardiovascular-associated deaths. Despite the connection between MVs and cardiovascular morbidity, the mechanisms of MV release by macrophages remain unknown. *Materials and methods*: Nanoparticle tracking analysis (NTA, NanoSight) was used to characterise the release of MVs from the RAW264.7 macrophage cell line, following treatment with the pro-inflammatory cytokine TNF-α (20 ng/ml). Lipid raft domains were identified by confocal microscopy using cholera toxin-based staining of GM1 gangliosides. *Results*: Kinetic studies using NTA indicate that untreated RAW cells release MVs at a constant rate (average R2=0.95) over 24 h. Treating RAW264.7 cells with TNF-α leads to a 1.8-fold increase in MV secretion rate for 6 h. After this initial rate increase, further MV release is completely suppressed up to 24h. TNF-α is a known activator of intracellular sphingomyelinases, enzymes involved in sphingomyelin metabolism. A neutral sphingomyelinase inhibitor, GW4869, suppresses the TNF-α-dependent rapid release of MVs. Sphingomyelin is a major component of plasma membrane lipid rafts, and confocal microscopy reveals disorganisation of lipid rafts within RAW264.7 cells after 6 h of TNF-α treatment. *Conclusions*: We hypothesise that physiological MV release is regulated by controlled organisation of specialised lipid domains. *In vivo*, pro-inflammatory activation of macrophages may disrupt the physiological release of MVs within the plaque, leading to MV accumulation and formation of microcalcifications. Further characterisation of these domains may reveal therapeutic targets to prevent the formation of microcalcifications.

**Myotube-derived exosomal miRNAs down-regulate Sirtuin1 in myoblasts during muscle cell differentiation**

Karim Chikh, S. Rome, A. Forterre, A. Jalabert, S. Pesenti, V. Euthine, A. Granjon and E. Lefai

CarMeN laboratory, INSERM 1060/INRA1235, France

*Introduction*: Previous studies have demonstrated that miRNAs are exported outside of cells and enclosed in small vesicles, like exosomes. It has recently been established that exosomes can mediate intercellular cross-talk through the tranfer of specific miRNAs. In this work, we addressed the question of whether skeletal muscle (SkM) exosomes contained specific miRNAs, and whether they could act as “endocrine signals” during myogenesis. *Materials and methods*: miRNAs within exosomes released from C2C12 myoblasts and myotubes were characterised by TaqMan Low-Density Arrays. *Results*: We compared the miRNA repertoires found in exosomes released from C2C12 myoblasts and myotubes, and found that certain muscle cell miRNAs were exported into exosomes (171 and 182 miRNAs, in exosomes from myoblasts and myotubes, respectively). Some miRNAs were expressed at higher levels in exosomes than in their donor cells and vice versa, indicating a selectivity in the incorporation of miRNAs into exosomes. Moreover, miRNAs from C2C12 exosomes were regulated during myogenesis. The predicted target genes of these regulated exosomal miRNAs were mainly involved in the control of important signalling pathways for muscle-cell differentiation (Wnt signalling pathway). We also demonstrated that exosomes from myotubes could transfer small RNAs (miRNAs or siRNAs) into myoblasts. Bioinformatic prediction indicated that Sirtuin 1 (Sirt1) was a target gene of multiple myotube-exosome miRNAs. Using the Sirt1 3’-UTR region, we demonstrated that myotube-secreted miRNAs are functional and can silence Sirt1 in myoblasts. Since Sirt1 regulates muscle gene expression and differentiation, our results indicate that myotube-secreted miRNAs could contribute to the commitment of myoblasts to differentiation. *Conclusion*: miRNA exosomal transfer would be a powerful means by which gene expression is orchestrated during myoblast differentiation to regulate SkM metabolic homeostasis.

**Personalised therapeutic management in multiple myeloma**

S. Rajeev Krishnan^1^, F. Luk^1^, G.E.R. Grau^2^, R.D. Brown^3^, Y.L. Kwan^4^ and M. Bebawy^1^

^1^University of Technology, Sydney, Australia; ^2^University of Sydney, Darlington, Australia; ^3^Royal Prince Alfred Hospital, Camperdown, Australia; ^4^Concord Repatriation General Hospital, Concord West, Australia

*Introduction*: Multiple myeloma (MM) is an incurable haematological malignancy affecting plasma cells marked by highly heterogeneous survival rate. Relapse is a significant impediment to the successful treatment of MM clinically. One of the main causes for relapse is drug resistance to cancer chemotherapy. Currently, risk stratification to MM sub-groups and categorisation of complete response to therapy are assessed based on molecular, cytogenetic markers using bone-marrow biopsy, as available systemic markers are incompetent in this regard. We are exploring the clinical significance of our recent in vitro and in vivo findings of a novel non-genetic basis to MDR, whereby tiny vesicles called microparticles (MPs) shed from cancer cell's surface transfer MDR phenotype intercellularly. MPs isolated from the blood of patients who suffer from MM will be phenotyped for resistance, adhesion and dissemination markers, and assessed whether these characteristics are predictive of treatment outcome. *Materials and methods*: We have analysed 30 de-identified MM patients. The platelet-free plasma was ultracentrifuged, and MM-derived microparticles were identified and quantified with flow cytometry using Annexin V450, CD 138 APC in BD TruCount tubes. Also, platelet-derived MPs were identified and excluded using CD41a PE, and compared to age-matched healthy volunteers. *Results*: Plasma cell-derived MPs were identified based on the CD138 and Annexin expression. The number of systemic plasma cell-derived microparticles was found to be significantly higher in MM patients compared to the healthy volunteers. *Conclusions*: There are elevated numbers of microparticles in MM which potentially correlate with tumour aggressiveness. Phenotyping MM-derived microparticles holds the potential for a personalised systemic biomarker to predict therapeutic response.

**Exosomes derived from HTLV-1 infected cells contain the viral protein tax**

A. Narayanan^1^, E. Jaworski^1^, R. Van Duyne^2^, S. Iordanskiy^1^, I. Guendel^1^, R. Das^1^, G. Sampey^1^, M. Chung^1^, A. Popratiloff^2^, K. Kehn-Hall^1^, C. Bailey^1^ and F. Kashanchi^1^

^1^George Mason University, Fairfax, VA, USA; ^2^George Washington University, Washington, DC, USA

*Introduction*: Human T-lymphotropic virus type 1 (HTLV-1) is the causative agent of adult T-cell leukemia (ATL). The HTLV-1 transactivator protein Tax has been identified as a critical component in the proliferation and transformation of human primary T-cells. This 40 kDa phosphoprotein not only manipulates chromatin remodelling within the host, but also subverts host cell DNA damage response mechanisms, cell cycle progression and apoptosis. *Materials and methods*: We utilised a combination of filtration and ultracentrifugation approaches to enrich for exosomes from culture supernatants of HTLV-infected cells. We employed western blots, mass spectrometry and cytokine arrays to proteomically characterise the host and viral components in these exosomes. We used RT-PCR approaches to determine the presence of viral transcripts in these exosomes, as well. *Results*: Our results demonstrated that exosomes derived from HTLV-infected cells contain traditional exosome proteins. Our proteomic studies revealed that these exosomes contain viral components, such as gp46 and Tax, as well as inflammatory mediators, including IL-6 and IL-10. We also investigated the presence of HTLV-1 mRNA transcripts of Env, Tax, HBZ and 5'LTR contained within exosomes. We evaluated the functional impacts of treating naïve recipient cells with exosomes secreted from HTLV-1-infected cells, and we determined that the exosomes were able to induce a response in reactive oxygen species (ROS) production. *Conclusion*: Our observation that the viral protein Tax is contained within exosomes and may be transmitted in an extracellular capacity raises important implications to pathogenesis associated with HTLV infections.

**Poster Session V (Arlington-Berkeley): Neural system April 19**

**Chair: *L. Rajendran and X. Breakefield***

**Analysis of exosomal microRNAs in cerebrospinal fluid from Alzheimer's disease patients**

O. Cagsal-Getkin and C.R. Vanderburg

Harvard Neurodiscovery Center-MGH, Boston, MA, USA

*Introduction*: Alzheimer's disease (AD), the most common type of ageing-associated dementia, is characterised by progressive accumulation of extracellular amyloid plaques comprised of amyloid β-peptide, and intracellular neurofibrillary tangles composed of aggregates of protein tau. Recent evidence suggests that MicroRNAs, short non-coding RNA molecules that regulate gene expression, may play a critical role in AD. We propose an innovative approach towards comparing cerebrospinal fluid (CSF) miRNAs profiles in AD and age-matched controls focusing on the miRNAs that are present in exosomes. *Materials and methods*: Exosomes from CSF were purified by successive centrifugations and filtration. Exosome pellets were evaluated by electron microscopy and western blotting. To prepare exosomal miRNA, extracts of exosome-derived RNA were bound to the matrix by mixing Picopure miRNA-binding buffer (Arcturus) with exosome RNA-containing miRNA extraction buffer, and RNA including small RNA was eluted in elution buffer (Arcturus). For the profiling approach, we had to pre-amplify the miRNA samples. In order to generate amplified sense RNA; we used the NCode miRNA Amplification System (Invitrogen). Using a liquid phase miRNAs multiplex bead arrays (Luminex), we characterised exosome-derived miRNAs profiles. We selected differentially expressed miRNAs and measured their expression by quantitative PCR. *Results*: Our results show that miRNAs can be recovered from frozen CSF and their expression can distinguish AD samples. By Luminex analysis, we identified differentially expressed miRNAs in AD CSF, and found that one of the miRNAs was significantly upregulated in AD CSF samples. *Conclusion*: This study provides evidence of differentially expressed exosomal miRNAs that are isolated from small amount real-life samples. Taken together and in agreement with previously published data, our findings support that these miRNA(s) could potentially serve as sensitive and specific biomarkers for AD.

**Influence of the cellular prion protein on exosome composition and trafficking**

C. Falker, H.C. Altmeppen, D.K. Thurm, M. Glatzel and S. Krasemann

University Medical Center Hamburg-Eppendorf, Hamburg, Germany

Prion diseases are fatal neurodegenerative disorders caused bythe transmission or spontaneous generation of “prions”. PrPSc, a misfolded conformer of the cellular prion protein (PrPC), represents an essential component of prions. PrPC and PrPSc were shown to be released via exosomes in mouse neuroblastoma cells (N2a) and other cell types. Moreover, inoculation of mice with PrPSc-containing exosome preparations leads to prion disease. The putative ability of PrPSc-loaded exosomes to also target and infect distant cell types makes exosomes a likely key player in the spread of prions from organ-to-organ in the living organism. However, the role or putative function of PrPC on exosomes is not well-defined. N2a cells express high amounts of PrPC and can be infected with mouse-adapted prion strains. To further investigate the role of PrP on exosomes, we generated an N2a PrPC knock-out cell line by establishing the design, assembly and expression of transcription activator-like effector nucleases (TALENs). Using this cell line, we analysed the influence of PrPC expression on the general release of exosomes by applying dynamic light scattering techniques, to quantify the number of exosomes. The latter value was compared to the respective protein content and PrPC-dependent exosome protein composition. In the second step, we investigated the uptake of PrPC-containing versus knockout exosomes by N2a cells and determined the subsequent intracellular fate of these exosomes. Preliminary data acquired in neuronal cell lines showed an altered uptake behaviour and intracellular trafficking of PrPC knockout exosomes. Exosomes devoid of PrPC were internalised more efficiently, and immunofluorescent co-staining with intracellular markers revealed a preferential lysosomal targeting. These data could be confirmed in primary neurons, where we were able to show that PrPC on exosomes negatively regulates exosome uptake. Taken together, our data hint to an active function of PrPC on exosomes.

**On the origin of cytosolic proteins in the human cerebrospinal fluid**

G. Minakaki^1^, S. Mathivanan^2^ and L. Rajendran^1^

^1^Systems & Cell Biology of Neurodegeneration, Division of Psychiatry Research, University of Zurich, Zurich, Switzerland; ^2^Department of Biochemistry, La Trobe Institute for Molecular Science, La Trobe University, Melbourne, Australia

*Introduction*: A major challenge in translational medicine is the identification of neurodegenerative disease biomarkers. The cerebrospinal fluid (CSF) is in the centre of scientific attention, since disturbances in neuronal homeostasis may be reflected in alterations in disease-related protein levels. The content of exosomes – vesicles with a unique morphology, protein and lipid composition – also deserves attention as a potential source of biomarkers, because disease-related proteins are among its constituents. Could it be that the CSF also receives a significant number of neuron-secreted exosomes which propagate with its flow and release their cytosolic content? In the present study, we are testing this hypothesis through an unbiased, systematic computational approach. *Materials and methods*: Combining the physiological human CSF proteome and ExoCarta 2012 Database, we created a cross-reference database, based on the EMBL-EBI International Protein Index (IPI) list, from UniProtKB/Swiss-Prot. The analyses were performed on candidates uniquely identified by Gene Symbol, Gene Entrez ID, IPI and Molecular Weight (MW). Cellular compartment categorisation was performed using the DAVID Bioinformatics Resources 6.7, NIAID/NIH Functional Annotation Tool. *Results*: The proteomes were screened for overlapping candidates. We found that 16% of the CSF proteome candidates have also been identified in exosomes. Of particular interest to our analysis, 60% of the CSF-cytosolic proteins are also encountered in exosomes. The comparison of the MW distribution of the CSF and exosome proteomes revealed that size may be a level of cytosolic protein secretion selection. *Conclusions*: The significant overlap between the cytosolic constituents of the CSF and exosome proteomes and distinct size distribution support that CSF-cytosolic proteins are to an extent contributed by exosomes. In the near future, higher-sensitivity proteomic approaches may provide a pool of novel disease biomarkers.

**Can cocaine by affecting A2a-D2 colocalisation in lipid rafts modify the intercellular transfer of A2a and D2 via the roamer type of volume transmission?**

A. Woods^1^, S. Genedani^2^, C. Carone^2^, M. Guescini^3^, G. Leo^3^, V. Stocchi^3^, D. Borroto-Escuela^4^, M. Morales^1^, D. Guidolin^5^, K. Fuxe^4^ and L.F. Agnati^6^

^1^NIDA IRP NIH, Baltimore, MD, USA; ^2^University of Modena Reggio Emilia Italy; ^3^Urbino, Italy; ^4^Karolinska Institutet, Sweden; ^5^University of Padova, Italy; ^6^IRCCS, Lido Venice University, Italy

Our work focuses on intercellular transfer of A2a and D2 receptors (R) via microvesicles in cell cultures and on action of cocaine on intercellular transfer of GPCRs via roamer type of volume transmission. Microvesicles referred to as exosomes [vesicles segregated within multivesicular bodies (MVBs) lumen] and shedding vesicles originated by direct budding from plasma membrane (PM). Shedding vesicles can be formed from lipid raft (LR) domains of PM, preceded by budding of small cytoplasmic protrusions, which detach by fission of their stalk. While exosomes formed by MVBs allow transfer of “packets of signals” (RNA, proteins, trophic factors), shedding vesicles from LRs may be the vehicle for transferring signalosomes to target cells, creating new transient integrative complex molecular networks in target cells, and hence a possible redeployment of entire neural networks cocaine affects dopamine (DA) transmission, and its action at DA Rs (especially D2) is affected by activation of A2a Rs. Thus, A2a–D2 interactions have been proposed as a possible new target for neuro-psychiatric disorders and drug addiction. Cocaine (150 nM; 8h) causes substantial and highly significant rise of membrane-associated D2 immunoreactivity (IR) and a small increase of membrane-associated A2a R in CHO cell cultures. Higher concentration of cocaine increases GM1 in CHO PM. GM1, a major component of LRs, involved in localisation and trafficking of GPCRs and in release of shedding vesicles. Proteins in GPCRs are associated with PM and sorted in shedding vesicles. Microvesicles can transfer competent GPCRs in HEK293T cell cultures, transiently transfected with A2a and D2 Rs. This report investigates whether cocaine at suitable concentrations (below 1 µM; 8 h) modulates release of microvesicles in cell cultures of CHO and COS-7 cells, transiently transfected with A2a and/or D2R. We will investigate whether cocaine modulates the release of microvesicles from cultures of astrocytes.

**A pathogenic role for exosomes in the brain of Down's syndrome patients: enhanced secretion of exosomes enriched with neurotoxic metabolites of the amyloid precursor protein**

E. Levy^1^ and S.A. Gauthier^2^

^1^New York University School of Medicine, New York, NY, USA; ^2^Nathan S. Kline Institute for Psychiatric Research, Orangeburg, NY, USA

*Introduction*: *Invitro* studies have shown that cultured neurons secrete exosomes containing full-length amyloid precursor protein (flAPP) and the APP processing products, carboxyl-terminal fragments (APP-CTFs) and amyloid β. We have shown that exosomes secreted into the brain extracellular space of transgenic mice, overexpressing human APP, transport higher levels of flAPP and APP-CTFs than exosomes secreted in the brain of wild-type mice, with no difference in the number of secreted exosomes. We investigated the potential pathogenic role of secretion of flAPP and APP-CTFs via the exosome secretory pathway, *in vivo* in the brain of Down's syndrome patients. *Materials and methods*: We developed a novel protocol to isolate exosomes secreted into the extracellular space of murine and human brain tissue, and demonstrated that brain tissues frozen for long periods of time yield exosomes with the same characteristics as exosomes isolated from either media of cultured cells or from freshly removed murine brains. *Results*: In accordance with the increased levels of flAPP and APP-CTFs in the brain of patients with chromosome 21 trisomy, exosomes secreted into the brain extracellular space of Down's syndrome patients are loaded with higher levels of flAPP and APP-CTFs than exosomes secreted in the brain of diploid individuals. Moreover, exosome secretion is enhanced in the brains of Down's syndrome patients compared to diploid controls. The ratio of APP-CTFs to flAPP is higher in brain exosomes compared to brain homogenates in both Down's syndrome and controls. Thus, high-level secretion of exosomes enriched with the APP-CTFs suggests a mechanism for the early onset of Alzheimer's disease, including deposition of amyloid β, in Down's syndrome patients. *Conclusions*: The exosome-secretory pathway plays a pathogenic role in the brain of Down's syndrome patients by enhanced secretion of exosomes enriched with APP-CTFs, neurotoxic proteins that are also a source of secreted amyloid β.

**Astrocytes secrete a heterogeneous population of microvesicles that share many exosomal markers and contain the co-chaperone STI1**

G. Hajj^1^, C. Arantes^2^, M.S. Dias^1^, I. Porto-Carreiro^3^, M. Roffe^1^, M. Prado^4^, R. Linden^3^ and V.R. Martins^1^

^1^AC Camargo Hospital, São Paulo, Brazil; ^2^University of São Paulo, São Paulo, Brazil; ^3^Federal University of Rio de Janeiro, Rio de Janeiro, Brazil; ^4^Robarts Reasearch Institute, Ontario, Canada

*Introduction*: The co-chaperone Stress Inducible Protein 1 (STI1) is secreted by astrocytes, and has neurotrophic properties upon binding to prion protein (PrPC) at the neuronal surface. However, STI1 lacks a signal peptide, and pharmacological approaches pointed that it does not follow a classical secretion mechanism. *Materials and methods*: We used diverse ultracentrifugation protocols, size exclusion chromatography, vesicle labelling, electron microscopy and nanotrack analysis to extensively characterise microvesicles (MVs). We used ERK1/2 activation assays and fluorecent microscopy to evaluate binding and activity of the vesicles. *Results*: Astrocyte MVs vary in size from 50 to 500 nm and present many exosomal markers, even though only a subpopulation had the typical exosomal morphology. The only protein present exclusively in vesicles that have exosomal morphology was PrPC. Multivesicular bodies were also found in astrocytes, containing intra-luminal vesicles of different sizes. STI1 is incorporated into MVs surface and promotes activation of ERK1/2 in neurons. *Conclusions*: These results indicate that astrocytes secrete a population of MVs of different size and morpholgy that labelled for classical exosomal markers. STI1 secretion in MVs is essential for its ability to trigger neuronal signalling. Supported by Sao Paulo State Foundation (FAPESP) and National Institute for Translational Neuroscience (INNT).

**Deep sequencing of exosomal microRNA associated with Alzheimer's disease**

Lesley Cheng, S.A. Bellingham, C.X. Sun, C.Y.J. Quek, B.M. Coleman, C.L. Masters and A.F. Hill

University of Melbourne, Melbourne, Australia

*Introduction*: Alzheimer's disease (AD) is the most common form of dementia in humans, and much evidence exists to suggest that the onset of AD occurs much earlier than our current ability to diagnose the disease. Diagnostic tests for AD need to be greatly improved in order to implement preventative strategies. Expression profiling of microRNA (miRNA) genes has diagnostic potential in human diseases such as cancer. Additionally, extracellular vesicles, such as exosomes, have been shown to provide a target for identifying miRNA based biomarkers. We have previously identified a panel of miRNA's that are dysregulated in prion diseases, which are also neurodegenerative disorders. We hypothesise that the profile of exosomal miRNA's may provide a reflective diagnostic picture to predict the onset and various stages of AD. *Materials and methods*: We isolated exosomal RNA from human serum and urine collected from the Australian Imaging, Biomarkers and Lifestyle Flagship Study (a cohort of healthy control and AD patients). We used a deep-sequencing to analyse the exosomal miRNA. Bioinformatics analysis was performed and used to generate miRNA expression profiles and characterise the small noncoding RNA species present in the exosomes. *Results*: We have established a deep-sequencing workflow to screen for candidate exosomal miRNA associated with AD in biological fluids. We have found a unique profile of microRNA associated with AD that could be potentially used as a disease biomarker in humans. In addition, brain specific microRNA and other RNA species were found in human serum exosomes which may provide insight into the role of cell-to-cell communication by exosomes within a diseased state. *Conclusions*: The significance of this work would allow the development of a diagnostic test comprising of a profile of RNA biomarkers associated with AD and has potential for other neurodegenerative diseases such as Parkinson's disease.

**The ability of neuronal exosomes to eliminate Alzheimer's amyloid-β protein**

Kohei Yuyama, H. Sun and Y. Igarashi

Hokkaido University, Sapporo, Japan

*Introduction*: Neuronal exosomes contain several amyloid-forming proteins related to neurodegenerative disorders, including amyloid β-protein (Aβ) in Alzheimer's disease and prion protein in Creutzfeldt-Jakob disease. However, the role of exosome against their dynamics is largely unknown. In this study, we examined the potency of exosomes in Aβ assembly and clearance. *Material and methods*: (a) We collected exosomes from culture media of neuro2a cells (N2a) or mouse primary cortical neurons by sequential ultracentrifugation. (b) We measured the level of Aβ amyloid in the mixture of the purified exosomes and monomeric Aβ to examine the ability to assemble Aβ. (c). To identify which cells can uptake exosomes, we exposed fluorescent-labelled exosomes to several types of cells, and then measured uptake level of exosomes and (d) We regulated exosome secretion in transwell cultures of N2a and microglia, and measured extracellular Aβ levels by ELISA to study the involvement of exosomes in Aβ clearance. *Results*: (a) We found higher levels of Aβ fibrils in the mixtures of Aβ with exosomes than that without exosomes, indicating the exosomes have the ability to promote Aβ amyloidogenesis. (b) We observed that the exosomes were uptaken into microglia but not neurons. We also confirmed that the exosome-bound Aβ was incorporated into microglia and (c) We identified that secretion of neuronal exosomes was modulated by sphingolipid metabolism. In the transwell study, upregulation of exosome secretion by treatment with sphingomyelin synthase siRNA enhanced Aβ uptake into microglia and significantly decreased extracellular Aβ levels. *Conclusions*: These results suggest that Aβ binds and assembles on the surface of exosomes and is delivered into microglia to degrade together with exosomes. Future challenges are to understand the exosome functions in Aβ-related pathology *in vivo*. The potential of exosomes to support Aβ clearance might be useful for the therapy of Alzheimer's disease.

**Exosomal and non-exosomal mediated release of α-synuclein: implications for Parkinson's disease**

Garima Thakur

University of Zurich, Zurich, Switzerland

*Introduction and Objectives*: α-Synuclein mutations/locus duplication or triplication of the synuclein gene leads to its aggregation, loss of its normal functions, initiating the pathological processes such as Parkinson's diseases. α-Synuclein is a cytosolic protein primarily localised to presynaptic vesicles. Recent evidence suggests that it could be released from the cells and be propagated in a prion-like manner also infecting grafted stem cells thus hampering therapeutic measures. The trafficking pathway of α-synuclein release into the extracellular space, and its re-entry into cellular cytosol of the recipient cells are still not understood properly. How much of the secreted α-synuclein is released via exosomes, and how much is released in non-exosomal form is currently unknown. It appears that at least some fraction of the secreted α-synuclein is released in the non-exosomal form. Our aim is to investigate the cellular mechanisms involved in the release of α-synuclein. *Methods*: We studied the unconventional secretion of α-synuclein by lipid binding assays and chemical compound inhibitors against Tec/Btk kinases (*in vivo* and *in vitro*). The secretion and intracellular levels of α-synuclein were measured via ELISA. For cell cyototoxicity assay we performed Lactate Dehydrogenase Assay (LDH). *Results*: α-synuclein is released from cells via both exosomal and a non-vesicular process regulated, in part, by the activity of the Tec/Btk kinases. *In vitro* and *in vivo* inhibition of Tec/Btk kinases by the inhibitor LFM-A13 inhibits α-synuclein release, revealing a putative drug target for therapeutic applications. *Conclusion*: Taken together, our results not only describe a novel mechanism through which α-synuclein is released, but also identified a novel drug/drug target to inhibit the release of α-synuclein, which may aid in the inhibition of disease propagation.

**Endothelial ectosomes are elevated in a rodent model of chronic cerebral hypoperfusion**

S. Schock^1^, D. Burger^2^, H. Edrissi^1^, R. Cadonic^1^, A.M. Hakim^3^ and C.S. Thompson^1^

^1^Ottawa Hospital Research Institute, University of Ottawa, Ottawa, Canada; ^2^Kidney Research Centre, Ottawa Health Research Institute, University of Ottawa, Ottawa, Canada; ^3^Ottawa Hospital Research Institute, University of Ottawa, Ottawa, Canada

*Introduction*: Cerebral small vessel disease (CSVD) results in a decrease in brain perfusion, is thought to cause the majority of lacunar or “silent” strokes, and is associated with cognitive impairment. An early pathological feature of CSVD is an increase in blood brain barrier (BBB) permeability. Ectosomes released into the circulation from by a variety of cell types, increase in several cardiovascular disorders. In the present study, circulating levels of ectosomes were measured in a rat model of CSV. *Methods*: Chronic cerebral hypoperfusion (CCH) was induced in male Long Evans rats by permanent bilateral common carotid artery occlusion. Circulating ectosomes were examined by flow cytometry and electron microscopy. Purified ectosomes were added to cultured rat brain microvascular endothelial cells, cell viability assessed by LDH assay and barrier permeability assessed by measuring electrical resistance (TEER). *In vivo*, BBB permeability was assessed using the Evans Blue assay. *Results*: Twenty four hours following the induction of CCH, circulating levels of annexin V+ and VE-cadherin+ectosomes were greatly elevated. When added to cultured cells, ectosomes induced death receptor and caspase 3-dependent cell death. When ectosomes were added to artificial endothelial barriers there was a significant decrease in electrical resistance, and when injected into the circulation of unoperated rats there was an increase in BBB permeability. *Conclusions*: CCH causes an elevation in circulating levels of ectosomes of endothelial cell origin. When these particles are purified and added to cultured cells they induce apoptosis, and when added to artificial endothelial barriers they cause a decrease in the electrical resistance. When injected into the circulation of unoperated rate the ectosomes cause a decrease in BBB permeability. These particles may be involved in the disruption of the BBB that accompanies human CSVD and a variety of other neurological conditions.

**Poster Session VI (Statler): Tissue injury/repair April 19**

**Chair: *B. Gyorgy and B. van Balkom***

**Exosomes in intercellular communication within the epidermal-melanin unit**

Alessandra Lo Cicero^1^, G. van Niel^2^, C. Delevoye^2^, F. Marsens^2^ and G. Raposo^2^

^1^Institut Curie, Paris, France; ^2^Institut Curie, Centre de recherche, Paris, France

In the thin outermost layer of the skin, the epidermal-melanin unit corresponds to the association of 1 melanocyte with 30–40 surrounding epidermal keratinocytes. Melanocytes are specialised in the production of the pigment melanin, which is synthesised within lysosome related organelle called melanosomes. The epidermal-melanin unit favours the transfer of mature melanosomes from the melanocyte to the keratinocytes to photoprotect the skin against the ionising radiation. The contact zone between melanocytes and keratinocytes is called the “pigmentation synapse”. In this area, the cells can communicate via the release of soluble factors that participate in the regulation of the pigmentation. We have characterised a new way of communication between primary human keratinocytes and melanocytes through the release of exosomes. To investigate the role of exosomes on the modulation of the pigmentation, we have used primary cells from Caucasian and black donors. We have characterised the exosomes released from these cell types, and we have analysed the effects of keratinocyte exosomes on melanocytes. Exosomes isolated from black donors increase the level of melanin when in contact with Caucasian melanocytes, due to the modulation of tyrosinase activity, the increase of the levels of melanosomal proteins, (PMEL) and proteins involved in melanosome transport (Rab27a). Conversely, exosomes isolated from Caucasian keratinocytes and incubated with black melanocytes decrease the production of melanin and the expression of melanosomal and transport proteins: PMEL, TYRP1 and Rab27a. These results demonstrate a role for exosomes in intercellular communication within the epidermis and their role in the regulation of pigmentation.

**Escherichia coli-derived extracellular vesicles induce emphysema via both IFN-gamma- and IL-17-dependent pathways**

Jun-Pyo Choi^1^, E. Choi^1^, Y. Kim^1^, S.G. Jeon^1^, Y.S. Gho^1^, Y. Kim^1^ and Y. Jee^2^

^1^Department of Life Science, POSTECH, Pohang, Republic of Korea; ^2^Department of Internal Medicine, Danguk University, Cheonan, Republic of Korea

*Introduction*: Lipopolysaccharide (LPS), a component of outer membrane from the gram-negative bacteria, is present in indoor dust and known as one of the factor to induce the airway inflammation. Gram-negative bacteria (e.g. Escherichia coli) secrete vesicles into the extracellular milieu, called by outer membrane vesicles (OMV) or extracellular vesicles (EV). Our recent evidence indicates that gram-negative bacteria EV in indoor dust induce Th1 and Th17 neutrophilic pulmonary inflammation. *Objective*: To evaluate the role of E. coli-derived EV on the development of lung pathology. *Methods*: To induce lung pathology, E. coli EV were administrated intranasally into the airways of 6-weeks-old mice 6 times on days 0, 1, 7, 8, 14 and 15. Pulmonary inflammation, emphysema and immunologic parameters were evaluated 48 h after the final EV administration. *Result*: E. coli-derived EV were present in indoor dust. *In vivo* application of the EV induced uptake by airway epithelial cells and also by alveolar macrophages, which elicits pulmonary inflammation accompanied by up-regulation of pro-inflammatory mediators. Repeated inhalation of E. coli-derived EV caused neutrophilic inflammation and emphysema in a dose-dependent manner. These phenotypes were accompanied by the production of both Th1 and Th17 cells. Additionally, emphysema induced by E. coli-derived EV was partially eliminated by the absence of IFN-gamma or IL-17. *Conclusion*: E. coli-derived EV induce lung emphysema via both IFN-gamma and IL-17 dependent pathways.

**Extracellular vesicles (EVS) from activated fibroblasts promote lung fibrotic remodelling**

S.S. Wong^1^, M. Cernelc-Kohan^1^, M. Checa^1^, M. Negreros^1^, C. Taype de Roberts^1^ and J.S. Hagood^2^

^1^Department of Pediatrics University of California-San Diego, La Jolla, CA, USA; ^2^Department of Pediatrics University of California-San Diego and Rady Children's Hospital of San Diego, San Diego, CA, USA

*Introduction*: Idiopathic pulmonary fibrosis (IPF) is an incurable/fatal disease characterised by the differentiation of fibroblasts to myofibroblasts. In response to proinflammatory cytokines *in vitro*, fibroblasts release membrane-originating extracellular vesicles (fEVs, including exosomes, microvesicles and microparticles). We also detected the presence of similar EVs in bronchoalveolar lavage fluid (BALF) from IPF. In this study, we further examined the effects of fEVs in a model of lung fibrosis. We hypothesise that fEVs from activated fibroblasts transfer profibrotic signalling molecules and affect lung repair. *Materials and methods*: fEVs were isolated from conditioned media (CM) of normal human lung fibroblasts (NHLF) in SFM with IL-1β/TNF-α. After a series of centrifugations, fEVs were characterised by transmission EM (TEM) and nanoparticle tracking analysis (NTA). Using a bleomycin (BL) model of lung fibrosis in NOD/scid/IL2Rgamma(null) mice, we delivered fEVs iv. on D14, and collected lung tissues D21 after BL administration. *Results*: Treatment of BL-mice with fEVs (BL-fEVs) obviously increased lung fibrotic remodelling compared to that of BL-PBS mice, as measured by H&E and histopathology. Analyses of qPCR revealed that lungs of BL-fEVs had obviously higher expressions of FnEDA, SERPINE1, and ACTA2. Moreover, pSMAD2/3, but not SMAD2/3, immunoreactivity increased in lungs of BL-fEVs mice, as determined by Western blot. It suggests an additional fibrotic signalling transduction through transfer of fEVs containing myofibroblastic phenotype. Studies of immunoimaging (IF/IHC) for these responses and co-cultures for genetic transfer are on-going. *Conclusions*: fEVs exaggerated the BL-induced fibrotic effects *in vivo*. fEVs released by activated fibroblasts are of importance for sustaining or amplifying profibrotic cellular phenotype programming by transfer of molecular signals (supported by NIH #HL082818 & Pulmonary Fibrosis Foundation).

**Exosomes released from the human neural stem cell line CTX0E03 enhance fibroblast migration within an in vitro scratch assay model of wound repair**

R. Corteling, D. Ravirala and J. Sinden

ReNeuron Ltd, Guildford, Surrey, UK

*Introduction*: CTX0E03 is a conditionally immortalised human neural stem cell (hNSC) line derived from fatal cortical tissue, genetically modified to incorporate the c-mycERtam transgene to allow its stable proliferation under the regulation of 4-hydroxytamoxifen (4OHT). We have previously demonstrated its functional efficacy in pre-clinical models of stroke and peripheral arterial disease. The mechanism by which the cells function is largely unknown, however, a number of recent studies have indicated hNSCs promote repair by angiogenesis, neurogenesis and immunomodulation. We report here for the first time that CTX0E03 cells release exosomes which stimulate human fibroblast migration in an *in vitro* model of wound repair. *Materials and methods*: CTX0E03 cells were continuously cultured for up to 6 weeks using Integra CELLine bioreactors in the presence of growth factors and 4OHT. Released exosomes were purified by ultracentrifugation and quantified by BCA protein assay. Transmission electron microscopy was used to confirm the presence of exosomes and a combination of flow cytometry and western blotting to characterise the final product. The ability of purified exosomes to stimulate fibroblast migration was determined using ibidi inserts and primary human dermal fibroblasts under basal conditions. *Results*: CTX0E03 cells constitutively release exosomes. The resulting purified exosome fraction was >99% positive for CD63 and CD81 and has a greater expression level of ALIX compared to the corresponding microvesicle fraction. Exosomes purified from all time points (weeks 2–6) during the continuous culture period significantly enhanced fibroblast migration and wound healing, with a peak response between 5–10µg/ml compared to basal conditions. *Conclusion*: Exosomes released from the human neural stem cell line CTX0E03 enhance fibroblast migration in an *in vitro* model of wound healing suggesting that exosomes may contribute to the mechanisms by which hNSCs promote repair.

**Analysis of extracellular vesicles in vitreous samples**

B. Gyorgy^1^, Z. Recsan^1^, A. Kittel^2^, K. Paloczi^1^, L. Turiak^3^, K. Vekey^1^, J. Nemeth^1^, Z.Z. Nagy^1^ and E.I. Buzas^1^

^1^Semmelweis University, Budapest, Hungary; ^2^Institute of Experimental Medicine, Hungarian Academy of Sciences, Budapest, Hungary; ^3^Chemical Research Center of the Hungarian Academy of Sciences, Budapest, Hungary

*Introduction*: Extracellular vesicles (EVs) were previously shown to participate in angiogenesis, tissue remodelling and tissue regeneration. In this work, we aimed at first time characterisation of protein and RNA profiles of EVs isolated from human vitreous samples. *Materials and methods*: We analysed EVs from vitreous fluid collected during vitrectomy from patients with proliferative diabetic retinopathy and primary rhegmatogenous retinal detachment. EVs were isolated using differential centrifugation, and were visualised by transmission electron microscopy (TEM). Size histograms of EV preparations were determined by a resistive pulse sensing approach (qNano). Cellular origin of EVs was determined by flow cytometry (FC). Protein and RNA profiles of EVs were analysed by mass spectrometry (MS) and bioanalyzer (Agilent), respectively. *Results*: TEM clearly shows various populations of EVs in the vitreous fluid. Peak EV size was around 150 nm in diameter. The presence of EVs in vitreous fluid was also confirmed using FC-based on annexin V binding. Most EVs in the vitreous fluid were derived from platelets and endothelial cells. MS revealed classical EV-associated proteins including actin, actin-binding proteins (e.g. ankyrin), tubulin, clusterin, heat shock proteins and enzymes. However, we also identified eye-specific proteins in EVs including retinal dehydrogenase, retinol binding protein and lens specific proteins (lensin, crystallin etc.). Most importantly, RNA profiling has revealed that miRNA molecules were present in vitreous-fluid-derived EVs in very high amounts. *Conclusion*: In this work, we successfully isolated and characterised EVs from vitreous fluid and demonstrated the presence of miRNAs in these structures. Demonstration of angiogenesis-inducing miRNAs in vitreous fluid EVs may lead to identification of new biomarkers or novel therapeutic targets in eye disorders.

**Effects of exosomes on the cell cycle regulation of mast cells?**

G.V. Shelke^1^, C. Lässer^2^, C. Malmhäll^2^, J. Nilsson^3^, Y.S. Gho^4^ and J. Lötvall^5^

^1^Krefting Research Centre, University of Gothenburg, Gothenburg, Sweden; ^2^Krefting Research Center, University of Gothenburg, Gothenburg, Sweden; ^3^Sahlgrenska Cancer Center, University of Gothenburg, Gothenburg, Sweden; ^4^Deptartment of Life Science, Pohang University of Science and Technology, Pohang, Republic of Korea; ^5^Krefting Research Center, University of Gothenburg, Gothenburg, Sweden

*Introduction*: Exosomes have multiple regulatory effects in recipient cells. Previously, our group has shown that exosomes shuttle functional RNA between cells, and we have described the presence of mature miRNA in exosomes (Human Mast Cell-1: HMC1). In this study, we hypothesised that exosomes may influence the regulation of the cell cycle in recipient cells, and thus act as secondary regulators. *Materials and methods*: To investigate this, HMC1-derived exosomes (Exo) were harvested by multiple centrifugations and filtration steps. Cell cycle arrest was induced by exposure of HMC-1 cells to 150 uM hydrogen peroxide (H2O2). The effect of HMC1-Exo pre-treatment was studied using cell viability/proliferation in recipient cells, upon H2O2 exposure at different points of time. Highly abundant miRNAs in HMC1-exo, previously reported, were further analysed using DIANA miPath-3.0 (strict) software. *Results*: A Non-toxic dosage of H202 induces inhibition of cell proliferation in HMC-1. HMC-1-Exo pre-treated cells significantly improved survival and proliferation, when compared to non-treated cells after exposure to H202. Pathway analysis of abundant miRNA (fold change≥2) shows 42 target genes that are involved in various phases of cell cycle transition. These pathways include key cell cycle regulators such as CDKN2A, WEE1 and CHEK2. *Conclusions*: Taken together, these preliminary results suggest that HMC1-Exo can modulate the cell cycle arrest imparted by H2O2 on HMC-1 cells, and may be a potential secondary regulator of the cell cycle.

**Abundance of active lysyl oxidase like 2 on the surface of exosomes by endothelial cells is increased upon stimulation with collagen-I or hypoxia**

O.G. de Jong, B.W.M. Balkom, L.M. van der Waals, H. Gremmels and M.C. Verhaar

Department of Nephrology and Hypertension, University Medical Center Utrecht, Utrecht, The Netherlands

*Introduction*: Endothelial cells secrete exosomes that can stimulate migration and angiogenesis in an autocrine manner. A quantitative proteomics analysis of these exosomes previously identified 1,354 proteins, of which several showed altered abundances after exposure of their producing cells to hypoxic conditions. Proteins that showed altered abundances were mainly involved in cytoskeletal and extracellular matrix remodelling. One of the up-regulated proteins, lysyl oxidase like 2 (LOXL2), was of specific interest given its multiple roles in extracellular matrix (ECM) remodelling, angiogenesis and cell differentiation. *Material and methods*: Exosomes were collected from the cell culture supernatant of HMEC-1 cells by differential ultracentrifugation and analysed by sucrose density gradient analysis. LOXL2 activity was determined using an Amplex red-based assay, using cadaverine as a substrate. *Results*: We determined that LOXL2 is associated with exosomes using sucrose density gradient analysis, showing peak fractions of both LOXL2 and Flotillin-1 at a density of 1.10–1.11 g/ml. This association was confirmed by immune electron microscopy. By immunoblotting, we confirmed a 2-fold increase of LOXL2 in exosomes grown in 2% O2, as well as on a collagen-I coated surface. Using a proteinase K protection assay, we found that LOXL2 is present on the surface of exosomes, placing it in the same compartment as the ECM. Furthermore, a lysyl oxidase activity assay showed that LOXL2 is enzymatically active in the intact exosomes, and that exosomal lysyl oxidase activity increases 1,5-fold when ECs are cultured in hypoxic conditions. *Conclusion*: We show that LOXL2 is associated with endothelial cell-derived exosomes. Its abundance on these exosomes is increased by culturing of endothelial cells under hypoxia or on collagen-I coated surfaces. These data suggest a role for endothelial cell-derived exosomes in ECM remodelling through LOXL2 delivery, under the regulation of h.

**Extracellular vesicles derived from endothelial progenitor cells accelerate glomerular healing in anti-Thy1.1 nephritis through inhibition of complement-mediated injury and triggering of angiogenesis**

Vincenzo Cantaluppi^1^, D. Medica^1^, F. Figliolini^1^, M. Delena^1^, C. Mannari^2^, M. Migliori^3^, V. Panichi^3^, C. Tetta^4^ and G. Camussi^1^

^1^University of Turin, Turin, Italy; ^2^University of Pisa, Pisa, Italy; ^3^Versilia Hospital, Lido di Camaiore (LU), Italy; ^4^Fresenius Medical Care, Germany

*Introduction*: Glomerulosclerosis, following immune-mediated nephritis, is a leading cause of chronic kidney disease. Endothelial progenitor cells (EPCs) have an angiogenic activity due to paracrine factors, including extracellular vesicles (EVs). The aim of this study was to evaluate whether EPC-EV accelerate glomerular healing in anti-Thy1.1 glomerulonephritis, and the pattern of RNAs involved in glomerular regeneration. *Methods*: EPCs were isolated from peripheral blood, and EVs were characterised for protein and RNA content. Wistar rats were treated with an infusion of 400 g anti-Thy1.1 antibody in the presence or absence of 30 g EPC-derived EVs, treated with vehicle or 1U/ml RNase. EVs from human fibroblasts were used as control. Proteinuria, creatinine clearance and histology were evaluated on day 4, 7 and 14. *In vitro*, we studied the effects of EPC-EVs on glomerular endothelial cells, podocytes and mesangial cells. *Results*: EPC-EVs carried mRNAs coding for complement inhibitors (Factor H, DAF, CD59) and pro-angiogenic microRNAs (miR-126, miR-296). EPC-EVs reduced mesangial cell activation, leukocyte infiltration, apoptosis and proteinuria and ameliorated renal function in Thy1.1-treated rats. These effects were not observed while treating EPC-EVs with RNase or using fibroblast EVs. EPC-EVs were internalised in mesangial cells, endothelial cells and podocytes, preserving their functional integrity. *In vitro*, we observed the transfer of mRNAs coding for human Factor H, DAF and CD59 in rat mesangial cells, overcoming the cross-species barrier. EVs triggered angiogenesis in glomerular endothelial cells through the transfer of selected microRNAs and mRNAs (eNOS, Akt), and preserved permeselectivity and expression of nephrin in podocytes. *Conclusions*: EPC-EVs accelerated glomerular healing in anti-Thy1.1 nephritis by triggering angiogenesis, preserving podocyte function and inhibiting mesangial cell injury through transfer of mRNAs coding for complement inhibitors and selected microRNAs.

**Extravesicular and total protein content of microparticles produced by wound healing myofibroblasts cells**

Amélie Langlois^1^, S.L. Larochelle^1^, S.S. Siagh^2^, H.G. Genest^3^ and V.J.M. Moulin^4^

^1^Centre LOEX de l'Université Laval, Canada; ^2^Université Laval, Québec, Canada; ^3^Centre hospitalier de l'Université Laval de Québec, Québec, Canada; ^4^Centre LOEX de l'Université Laval, Université Laval surgery department, Canada

Microparticles (MP) from blood cells have been widely studied. Despite the fact that the production of MP by myofibroblasts (Wmyo) during wound healing process has already been observed, it has never been characterised in literature. Thus, as a remedy to this lack of knowledge in the field of cellular communication, the proteome of MP has been determined using the two-dimensional differential in-gel electrophoresis (2D-DIGE). This method allowed us to investigate MP produced by six different populations of Wmyo. Because of the use of different Cydye labelling methods, we were able to analyse the extravesicular proteins and the total protein content of MP for each of the population of Wmyo. Fluorescent labelling of every gel were acquired using Typhoon Variable Mode Imager system, and all images were analysed by Delta2D software. The spots of interest had to contain highly constant and extremely conserved proteins in all populations. With these choices of parameters and Scaffold Viewer software, 133 spots were sent to mass spectrometry analysis for further characterisation. From this investigation, 292 different proteins were identified, with 95% probability of peptides being unique to one protein. This extensive variety of proteins includes several enzymes, elongation factors and a large proportion of cytoskeleton-related protein. Amongst all these new findings, one is more interesting: vimentin, a type 3 intermediate filament protein, usually in the cytoplasm, has been found to be extravesicular in MP produced by Wmyo. This result was confirmed with flow cytometry analysis and should help us to define the mechanism used by Wmyo to secrete MP. Further research will be needed to clarify this idea. Finally, knowledge of the protein content of MP used by Wmyo to communicate between each other will enable a better comprehension of the wound healing process.

**A hypothesis for a hidden circulation system with cell-free DNA molecules, microvesicles and microparticles: the primo vascular system (Bonghan system), a putative acupuncture meridian system**

Byung-Cheon Lee

Ki Primo Res. Lab./Korea Advanced Institute of Science and Technology, Daejeon, Republic of Korea

*Introduction*: Since 2002, we have investigated the anatomical reality of the primo vascular system (PVS, formerly Bonghan system) corresponding to the acupuncture meridian system. Considering all available data, we found that the PVS contained many different kinds of particles: cell-free DNA molecules, microvesicles and microparticles. Thus, we present evidence to support the hypothesis that the PVS may be a system for circulating cell-free DNA molecules, microvesicles and microparticles. *Material and methods*: In order to visualise the PVS, we have developed several methods: the alcian blue, the trypan blue and the chromidium-hematoxylin staining methods. We examined PVS tissues by using light and electron microscopes. Also, we were able to trace the PVS by using DiI (1,1’-dioctadecyl-3,3,3’,3’-tetramethylindocarbocyanine perchlorate) injection for over 12 hrs. *Results*: The examination of PVS samples with light and electron microscopes showed that the PVS contained cell-free DNA molecules, microvesicles and microparticles. Also, the morphological characteristics of the PVS were different in different tissues, among which the PVS above the pia mater of the brain showed the most dynamic cell-free DNA molecules. *Conclusion*: Our data suggest that PVS is deeply related with cell-free DNA molecules, microvesicles and microparticles. Given that PVS is a novel circulation system, the hypothesis that a cell-free DNA circulation system exists may shed new light on the function of the meridian system.

**Telocytes and stem cells: intercellular signalling via telopodes and extracellular vesicles**

Laurentiu M. Popescu

National Institute of Pathology, Romania

What are telocytes? Telocytes (TCs) are a newly described (2010) distinct type of interstitial cells. TCs are defined by their very long prolongations (hundreds of micrometers) called telopodes (Tps); see www.telocytes.com and Wikipedia. Patch voltage-clamp data show that TCs are unexcitable cells. TCs were found in many organs, where they form a 3D network among the organ-specific cells, blood capillaries, nerve endings and immunoreactive cells. Beyond the ultrastructural portrait of TCs, the immuno-phenotype, gene profile and the microRNA imprint were also described. TCs are key players in intercellular signalling. Tps establish direct contacts (junctions) with neighbouring cells (long distance signalling). Of particular interest are the junctions (nanocontacts) between stem and/or progenitor cells and TCs in myocardium *in situ*, as found by electron tomography. Short distance (local) signalling of TCs is achieved by extracellular vesicles, mainly by shed vesicles (*in situ* electron microscopy indicated an average diameter of 180 nm; n=6,094). Cultured cardiac TCs release growth factors (VEGF), cytokines (IL6) and chemokines (CXCL-1), presumably through shed vesicles (SELDI-TOF-MS and xMAP Technology). Using the membrane impermeable calcein and RNA oligos, we found *in vitro* that TCs can shuttle microRNAs to other cells through vesicles. Cardiac TCs co-cultured with cardiac stem cells in a transwell system (400 nm pores) accelerated the stem cell differentiation to cardiac myocytes. This makes the tandem TCs-stem cells a valuable candidate for cell therapy in myocardial infarction.

**Insights on small-size microparticles in acute coronary syndromes (ACS): relevance to fibrinolytic status, reparative markers and outcomes**

S. Montoro-Garcia^1^, E. Shantsila^1^, A. Lopez-Cuenca^2^, L. Tapp^1^, A.I. Romero^3^, D. Hernandez-Romero^3^, E. Jover^3^, E. Orenes-Pinero^3^, F. Marin^3^, M. Valdés^3^ and G.Y.H. Lip^1^

^1^Centre for Cardiovascular Sciences, University of Birmingham, Birmingham, UK; ^2^Department of Cardiology, Hospital Universitario Virgen de la Arrixaca, Murcia, Spain; ^3^Department of Cardiology, Hospital Universitario Virgen de la Arrixaca, Murcia, Spain

*Background*: Majority of microparticles (MPs) have usually not been included into prior analyses due their small size and limited resolution of conventional equipment. Our aim was to assess the levels of MPs of different cellular origin, sized below 0.5 µm polystyrene beads, denoted as small-size microparticles (sMP), and their relation to markers of cardiovascular repair and their impact on prognosis in patients with acute coronary syndromes (ACS). *Methods*: We compared the levels of sMP between patients with ST-segment elevation myocardial infarction (STEMI, n=50), non-STEMI (n=47), stable coronary artery disease (CAD, n=40) and healthy individuals (HC, n=40). In a separate study, the prognostic value of sMP was assessed in patients with non-STEMI (n=160). Annexin V-binding sMP (sAMP), platelet CD42b+ sMPs (sPMP), endothelial CD144+ sMP (sEMP) and monocyte CD14+ sMP (sMMP) were quantified using high resolution flow cytometry. Endothelial progenitor cells (EPCs) and monocyte expression of scavenger receptors were also quantified by flow cytometry. Fibrinolytic factors were measured by ELISA. *Results*: Counts of sAMP and sEMP were lower in STEMI after PCI (p<0.001 and p=0.025, respectively) but not in non-STEMI vs. CAD. sAMP were positively correlated with EPCs in non-STEMI (p<0.001). Likewise, plasminogen activators were negatively correlated with sAMP in non-STEMI and STEMI (p=0.02 and p=0.002, respectively). In non-STEMI patients, sEMP and sMMP were independently predictive for future admissions related to heart failure (p=0.034 and 0.013, respectively) and sPMP for major bleedings (p=0.002). The sAMP/EPCs ratio was higher in patients (before PCI) compared to STEMI patients. *Conclusions*: The sAMP/EPCs ratio could reflect a change in the apoptotic/reparative potential, this being a putative indicator for vascular repair. Thus, sMPs could be potentially implicated in the modulation of the post-ACS reparative response to injury, with prognostic implications.

**Cardiopulmonary bypass procedure induces extracellular vesicle formation**

R.M. Schiffelers, K.M.K. de Vooght, G. Andringa, C. Lau, M.A. de Jong, T.J. de Laar, R. van Wijk and W.W. van Solinge

University Medical Center Utrecht, Utrecht, The Netherlands

*Introduction*: Cardiopulmonary bypass procedure (CBP) gives stress on blood cells. We hypothesised that contact with exogenous surfaces induces blood cell vesiculation. *Materials and methods*: In the clinical set-up, blood from CBP-patients was collected from the heart–lung machine. In the *ex vivo* set-up, blood from healthy volunteers entered a neonatal CBP without connecting a patient to the machine. Extracellular vesicles and red blood cells were isolated by (ultra)centrifugation procedures and characterised with Nanosight regarding size and number. The interaction of red blood cell vesicles with endothelium was quantified using a pseudoperoxidase assay and visualised after labelling of vesicles with PKH67. mRNA expression levels of selected proteins involved in iron homeostasis were quantified by qPCR. *Results*: Surprisingly, vesicle formation could not be detected in blood collected from patients undergoing CBP. This could mean either that vesicles are not formed during the CBP or that the clearance rate of vesicles surpasses the formation rate. By subjecting blood to the *ex vivo* set-up, clearance could be eliminated. Here, vesicle formation was prominent and dependent on duration of CBP. Judging by the red colour of the vesicles, the predominant source were red blood cells. Apart from clearance by macrophages, these vesicles could be cleared by other cells and induce phenotypic changes. When we incubate these vesicles with activated endothelium, we observed an uptake and processing of vesicles, which was enhanced in the presence of lactadherin. This uptake led to upregulation of iron processing-enzymes such as heme-oxygenase-1, ferritin and ferroportin. *Conclusions*: Our results show that extracellular vesicles are formed during CBP, but these vesicles are rapidly cleared in patients. Apart from macrophages, extracellular vesicles can be taken up by other cell types and induce phenotypic changes that could potentially contribute to the diffused inflammatory state after CBP.

**Exosomal microRNA modulates pathways of liver fibrosis by regulating connective tissue growth factor (CTGF) expression in fibrogenic cells during chronic injury**

L. Chen^1^ and D.R. Brigstock^2^

^1^The Research Institute at Nationwide Children's Hospital, Ohio State University, Columbus, OH, USA; ^2^Department of Surgery, The Research Institute at Nationwide Children's Hospital, Ohio State University, Columbus, OH, USA

*Introduction*: To establish the regulation of Connective Tissue Growth Factor (CTGF) expression in hepatic stellate cells (HSC) by delivery of miR-214 in exosomes. *Materials and methods*: Exosomes isolated from conditioned medium of mouse HSC were characterised by Western blot, dynamic light scattering, zeta potential analysis and cryo-TEM. MiR-214 was detected by RT-PCR. Donor HSC were treated with GW4869 to inhibit exosome production and recipient HSC were transfected with a luciferase reporter vector under the control of a wild type CTGF 3’-UTR or a mutant CTGF 3’-UTR lacking the miR-214 binding site. *Results*: HSC exosomes were bi-membrane vesicles, 80–150 nm in diameter, negatively charged (-26mV), and positive for CD9. MiR-214 was present in exosomes and its levels were increased by transfection of HSC with pre-mir-214. MiR-214 levels in exosomes but not in cell lysates were reduced by pre-treatment of the cells with GW4869. Co-culture of miR214-transfected donor HSC with CTGF UTR luciferase reporter-transfected recipient HSC resulted in miR-214-dependent and GW4869-dependent regulation of activity of the wild type, but not mutated, CTGF 3’-UTR. CTGF 3-UTR activity in activated recipient HSC was more greatly inhibited in a GW4869-dependent manner by co-culture with freshly isolated donor HSC (high endogenous miR-214 levels) than with highly activated donor HSC (low endogenous miR-214 levels). Similar results were obtained for the human LX-2 HSC line. *Conclusions*: MiR-214 is exported exosomally between neighbouring HSC, suppressing CTGF production by the cells, After injury, miR-214 expression is suppressed, resulting in its reduced exosomal delivery and increased CTGF expression in recipient cells. Exosome-dependent delivery of miRs between neighbouring HSC represents a novel mechanism by which fibrotic pathways are regulated during chronic liver injury.

**Poster Session VI (Statler): RNA biomarkers April 19**

**Chair: *A. Hill and E. Nolte-’t Hoen***

**RNA profiles in microvesicles and exosomes from different melanoma cell lines**

Taral Lunavat, M. Eldh, C. Lässer, G. Shelke, R. Crescitelli and J. Lötvall

University of Gothenburg, Gothenburg, Sweden

*Introduction*: Extracellular vesicles such as microvesicles (MVs) and exosomes (EXOs) are nanosized vesicles that are released by most cells, including different melanoma cells. These vesicles contain RNA that implies a role in cell-to-cell communication, which gives opportunity for biomarker discovery and, more recently, its use in gene therapy. In this study, we compare the RNA profile of EXOs and MVs released from several melanoma cell lines. *Material and method*: The 2 melanoma cell lines, A375 and MML-1, were used for this study. Cells and apoptotic bodies were eliminated by centrifugation at 300*g* and 2000*g*. MVs were then isolated from the supernatant and pelleted at 16,500*g*. The supernatant from the MV isolation was filtered (0.20µm) before EXOs were pelleted at 120,000*g*. Total RNA was isolated from MVs and EXOs using the miRCURY™ RNA isolation kit (Exiqon) and was analysed using Agilent 2100 Bioanalyser. The concentration of RNA was normalised per million cells in both the cell lines. *Results*: When we compared RNA between EXOs and MVs in the individual cell lines, the EXO-enriched pellet from A-375 were shown to contain substantial RNA than the MV- enriched pellet. In MML-1, the amount of RNA in the 2 vesicle types was similar. This suggests that loading of RNA into the various types of extracellular vesicles is different between different cell lines. A375 EXO seems to be more prominent to be loaded with RNA than the MVs. In addition, surprisingly rRNA was present in both EXOs and MVs in both cell lines, but to a much lower extent in the EXOs. *Conclusions*: The RNA content in EXOs and MVs often differs, as does the RNA content in these different subgroups of vesicles released by different cells, even though they are of the same tumour background. To understand the biology of vesicles released by different cells, the role of EXOs and MVs and their differential RNA content must be considered.

**MicroRNA profiles of liver-perfusate-derived exosomes from patients with metastatic uveal melanoma and a comparison with other tumour-cell-derived exosomes**

M. Eldh^1^, R. Olofsson^2^, C. Lässer^1^, J. Svanvik^3^, M. Sjöstrand^1^, J. Mattsson^2^, P. Lindnér^3^, D.S. Choi^4^, Y.S. Gho^4^ and J. Lötvall^1^

^1^Department of Internal Medicine and Clinical Nutrition, Krefting Research Centre, Sahlgrenska Academy at University of Gothenburg, Gothenburg, Sweden; ^2^Department of Surgery, Institute of Clinical Sciences, Sahlgrenska Academy at University of Gothenburg, Gothenburg, Sweden; ^3^Transplant Institute, Institute of Clinical Sciences, Sahlgrenska Academy at University of Gothenburg, Gothenburg, Sweden; ^4^Division of Molecular and Life Sciences, Department of Life Science, Pohang University of Science and Technology, Pohang, Republic of Korea

*Introduction*: Uveal melanoma arises from melanocytes within the eye, and about one-third of the patients ultimately develop liver metastases, with poor prognosis. No reliable diagnostic or prognostic biomarkers are available. Isolated hepatic perfusion (IHP) has been used to distribute high local concentration of chemotherapy with minimal systemic exposure, and has a 70% positive response rate with prolonged survival. The aim of this study was to characterise exosomes in the perfusate of patients with uveal melanoma liver metastases undergoing IHP, and to determine the microRNA (miRNA) content of these exosomes. *Material and methods*: Liver perfusate from 12 patients undergoing IHP were collected directly from the liver circulation, and isolated exosomes were visualised by electron microscopy (EM) and identified by flow cytometry for CD9, CD63 and CD81. Exosomal RNA was analysed by capillary electrophoresis and real-time PCR, and the miRNAs were further analysed by clustering and KEGG pathway analyses using Cluster 3.0 and DIANA-miRPath v.2.0 software. *Results*: Liver perfusate contain CD9, CD63 and CD81 positive exosomes. The liver-perfusate-derived exosomes were shown to have no or little rRNA, and enrichment of lower molecular weight RNA. The miRNA profiles of the exosomes were similar between patients, but not between different melanoma or other tumour cell lines. The clustering and KEGG pathway analyses of the miRNA suggest pathways related to melanoma, together with glioma, hedgehog signalling and prostate cancer. *Conclusions*: Liver perfusate contains exosomes from melanoma cells, and their miRNA content may be a potential source of diagnostic biomarker for uveal melanoma. The discovery of new biomarkers can make a discernible difference, as there are no good treatments for uveal melanoma and an early detection might change the patients prognosis.

**The relevance of exosomal miRNAs in triple-negative breast cancer progression**

K. O'Brien^1^, C. Corcoran^1^, S. Rani^1^, S. McDonnell^2^, L. Hughes^2^, J. Crown^3^, M. Radomski^1^ and L. O'Driscoll^1^

^1^School of Pharmacy and Pharmaceutical Sciences & TBSI, Trinity College Dublin, Dublin, Ireland; ^2^School of Chemical and Bioprocess Engineering, University College Dublin, Dublin, Ireland; ^3^Molecular Therapeutics for Cancer Ireland & St. Vincent's University Hospital, Dublin, Ireland

*Introduction*: We have previously shown that triple-negative breast cancer (TNBC) exosomes from Hs578Ts(i)8 conditioned medium (CM), versus its less aggressive isogenic variant, Hs578T exosomes, increase the proliferation, invasion, motility and migration of 3 breast cancer cell lines and stimulate angiogenesis. This study aimed to analyse miRNA contents and identify differentially expressed miRNAs in Hs578T cell and exosomes variants to potentially identify mediators of TNBC progression. *Materials and methods*: TaqMan low-density arrays (384 miRNAs) and qPCR validations (TaqMan small miRNA assay) were performed on samples from Hs578T and Hs578Ts(i)8 cells and exosomes (isolated from CM by filtration and ultracentrifugation) (n=3). miRNA CT values of <35 were taken as present, and ≥35 as absent from the sample. Fold changes were calculated by determining 2(-ΔΔCT), upon normalisation of CT values to RNU48 (endogenous control). *Results*: A total of 308 miRNAs were detected in Hs578T; 92% of which are in exosomes, 13% present in exosomes only and 79% found in cells and exosomes. For Hs578Ts(i)8, 270 miRNAs were detected; 94% present in their exosomes, 19% found in exosomes only and 75% found in both. Of the 3 miRNAs upregulated (>2 fold) and 209 downregulated (>2 fold) in Hs578Ts(i)8 exosomes versus Hs578T exosomes, 1 was commonly upregulated, and 70 commonly downregulated in Hs578Ts(i)8 cells and exosomes. Several miRNAs were validated by qPCR, two of which were chosen for functional analysis. miR-X and miR-Y are reduced by 33 and 77 fold in Hs578Ts(i)8 exosomes and are reduced by 1×10^3^ fold and 2×10^3^ fold in Hs578Ts(i)8 cells. *Conclusions*: Exosomes may communicate by transferring differential miRNA contents. We believe that we have identified a panel of miRNAs contributing to these events. Further analysis of miR-X and miR-Y is underway to determine their function in cells and exosome-mediated phenotypic alterations.

Funded by: Marie Keating Foundation PhD Scholarship at Trinity College Dublin.

**Functional transfer of the miR-200 microRNA family from metastatic to non-metastatic breast cancer cells via microvesicles**

M.T.N. Le, R.P. Henriques and J. Lieberman

Program in Cellular and Molecular Medicine, Boston Children's Hospital, Boston, MA, USA

*Introduction*: microRNAs (miRNAs) have been discovered in microvesicles (MVs) that circulate in the bloodstream, generating excitement about their possible use as non-invasive biomarkers. The miR-200 family of miRNAs, which regulates the mesenchymal to epithelial transition (MET), is one of these new biomarkers as its members are elevated in the plasma of cancer patients. However, the functional significance of the circulating miR-200 family is unknown. A previous study in our lab identified the miR-200 family as a miRNA signature of metastatic breast cancer 4T1 cells. In an isogenic set of mouse breast cancer cells with distinct metastatic capabilities, the miR-200 family was only expressed in tumour cells able to colonise distant sites and form macrometastases. Moreover, ectopic expression of miR-200 miRNAs in non-metastatic 4TO7 cells enabled these cells to form macroscopic metastases. *Material and methods*: We isolated MVs from 4TO7 and 4T1 cells and compared the expression of miR-200 miRNAs by qRT-PCR or firefly assay. 4TO7 cells were co-cultured with 4T1 cells and assayed for the levels of miR-200s and their downstream targets. The transfer of miR-200s from 4T1 to 4TO7 cells were also examined by a luciferase assay and live cell imaging. *Results*: Our preliminary data reveal that 4T1 cells, but not 4TO7 cells, release all the members of the miR-200 family into microvesicles in tissue culture supernatants and in the circulation. Moreover, direct or transwell co-culture with 4T1 cells results in an increase in miR-200 miRNAs, downregulation of miR-200 targets, and upregulation of MET markers in 4TO7 cells. *Conclusions*: These data suggest that 4T1 cells secrete miR-200 miRNAs in microvesicles that are taken up by 4TO7 cells with functional consequences to promote MET and in metastasis.

**Different exosome cargo from plasma/bronchoalveolar lavage in non-small-cell lung cancer**

Marta Rodriguez Moreno^1^, J. Silva Leal^1^, A. Lopez Alfonso^2^, F. Bonilla Velasco^3^ and V. Garcia Barberon^1^

^1^Fundacion para la Investigacion Biomedica del Hospital, Universitario Puerta de Hierro-Majadahonda, Madrid, Spain; ^2^Department of Medical Oncology, University Hospital Infanta Leonor, Madrid, Spain; ^3^Department of Medical Oncology, University Hospital Puerta de Hierro Majadahonda, Madrid, Spain

*Introduction*: Exosomes were isolated and characterised in plasma and bronchoalveolar lavage from patients with non-small-cell lung cancer and from patients with several pulmonary diseases. *Material and methods*: Exosomes from 30 and 75 patients with tumour and non-tumour pathology were quantified by acetylcholinesterase activity and characterised by western blot and confocal microscopy. Differences in exosome cargo were analysed by miRNA PCR. *Results*: Exosomes were detected in greater amounts in plasma than in BAL in both pathologies (p<0.001). The most miRNAs evaluated by PCR array were detected in tumour plasma, tumour BAL and non-tumour BAL pools, but only 56% were detected in non-tumour plasma pool. Comparing the top 10 miRNAs with the highest levels detected in each pool, we only found elevated homology between the BAL samples of the 2 pathologies. In tumour plasma, we found a higher percentage of miRNAs with increased levels than in tumour BAL and non-tumour plasma. *Conclusions*: Data show the differences between BAL and plasma exosome amount and miRNA content and support the view that tumours may preferentially use blood to spread the release of specific genetic material, in relation with the local airway.

**Profiling of microRNAs in exosomes from PC-3 prostate cancer cells**

N.P. Hessvik, S. Phuyal, A. Brech, K. Sandvig and A. Llorente

Oslo University Hospital – The Norwegian Radium Hospital, University of Oslo, Oslo, Norway

*Introduction*: Exosomes contain miRNAs and might therefore be vehicles transferring genetic information between cells. In addition, miRNAs associated with exosomes present in biological fluids can potentially be used as biomarkers. The aim of this study was to investigate whether there is a sorting of miRNAs into exosomes in the prostate cancer cell line PC-3 and to identify exosome-enriched microRNAs that may potentially be used as biomarkers for prostate cancer. *Materials and methods*: Exosomes were isolated from the conditioned media of prostate cells by ultracentrifugation and inspected by electron microscopy. The presence of the exosomal markers CD9, CD63 and CD81 was confirmed by western blot. MiRNA microarray analysis was used to identify miRNAs in our samples, and RT-qPCR was performed on selected miRNAs. *Results*: MiRNA microarray analysis revealed that the miRNA profile of PC-3 released exosomes was similar to the profile of the corresponding parent cells. Nevertheless, a sorting of certain miRNAs into exosomes was observed, and low number miRNAs (miRNAs with a low number in their name) were found to be underrepresented in these vesicles. Moreover, the miRNA profile of PC-3 cells resembled the miRNA profile of RWPE-1 cells, though some miRNAs were found to be differently expressed in these cell lines. RT-qPCR on selected miRNAs and snRNAs confirmed the results from the miRNA arrays. Finally, Ingenuity Pathways Analysis was used to predict functions regulated by the miRNAs identified in exosomes and cells. *Conclusions*: In this study we show that exosomes released from PC-3 cells contain miRNAs, and that the profile of exosomal miRNAs resembles the miRNA profile of the parent cells. Interestingly, we show that there is a selection of some miRNAs into exosomes and that low number miRNAs are underrepresented in these vesicles.

**Identification of exosomal microRNAs involved in the ovarian cancer associated omental microenvironment by next generation sequencing method**

Chi Lam Au Yeung, T. Tsuruga, N.N. Co, T.L. Yeung, C.S. Leung, K.K. Wong and S.C. Mok

The University of Texas MD Anderson Cancer Center, Houston, TX, USA

*Introduction*: Advanced stage ovarian cancers spread beyond the pelvis and preferentially metastasize to the omentum, which is mainly composed of adipose tissue, suggesting that the omental microenvironment is a favourable niche for ovarian cancer cells. However, the mechanisms underlying the effect of omental adipose tissue on ovarian cancer progression are poorly understood. Recent studies showed that exosomes also contain non-coding RNAs such as microRNAs (miRNAs). Thus, we hypothesise that exosomal miRNAs may play an important role in omental metastasis of ovarian cancer cells. *Material and methods*: Ion Torrent next generation sequencing method was performed on miRNAs isolated and enriched from exosomes and cell lysates of ovarian cancer cell lines (OVCA), the epithelial component of microdissected omental ovarian cancer tissues (CT), normal omental adipose tissues (OMN) and ovarian cancer-associated omental adipose tissues (OMT). *Results*: By integrating the miRNA expression profiles, a total of 17081 miRNAs and their variants were identified in the samples examined. Among the identified miRNAs and variants, 1,335 of them showed an increase in copy numbers in CT compared to those in OVCA, suggesting that they were up-regulated by mediators in the omental microenvironment in situ. Among them, 65 expressed at significant higher levels in OMT-derived exosomes compared with those in OMN-derived exosomes and OVCA-derived exosomes. Subsequent prediction of miRNA targets using the Ingenuity Pathways Analysis software showed that a majority of these 65 miRNAs were associated with cell proliferation, migration and invasion, apoptosis, cell cycle arrest and angiogenesis. *Conclusions*: Exosomal transfer of OMT-derived miRNAs in the omental microenvironment is one of the mechanisms that could alter miRNA expression, and subsequently mRNA expression in the neighbouring ovarian cancer cells, which may facilitate ovarian cancer progression in the omental tumour microenvironment.

**Exosome-mediated cancer metastasis is a novel target for cancer therapy**

N. Kosaka, M. Ono, K. Hagiwara, Y. Yoshioka, T. Katsuda, N. Tominaga and T. Ochiya

National Cancer Center Research Institute, Tokyo, Japan

Cell–cell communications of cancer cells and microenvironmental cells are critical for the acquisition of malignancy in human cancer; however, the precise molecular mechanisms of cell-cell communications of cancer cells and microenvironmental cells remain unclear. Here we show the contribution of nSMase2-mediated exosomes from cancer cells to the cancer cell metastasis in vivo via the induction of angiogenesis in the tumour. In addition, exosome from metastatic cancer cells encapsulated the angiogenic microRNAs, miR-210, resulting in the activation of endothelial cells. Furthermore, we have also discovered that exosomal microRNAs from mesenchymal stem cells led the cancer cells to become a quiescent status. Thus, these results suggest that inhibition of exosome in cancer microenvironment might be a novel treatment for cancer metastasis. For this purpose, we have developed an exosome inhibitor screening assay by combining 2 simple methods; one is a cell-based high-throughput assay to monitor the modulation of exosome secretion, and the other one is high-sensitive quantification methods, ExoScreen, to confirm the result of exosome secretion from the first assay. Our results show the usefulness of exosome inhibitors in the development of research tools and therapeutics.

**Circulating microvesicles contain critical elements of the RISC complex**

David Spetzler, K. Brown, M. Millis, S. Smith, K. Yeatts, Z. Zhong, A. Stark, Y. Kojima and J. Garcia

Caris Life Sciences, USA

Circulating microvesicles (cMV) are small, membrane-bound vesicles shed from cells and are thought to be involved in intercellular communication. It has been discovered that cMVs contain microRNAs (miRs), which, in cells, must be bound to Argonaute (Ago) protein as part of the RNA-induced silencing complex (RISC) in order to regulate translation. The protein GW182 is a functional partner of Ago and is another important component of some types of RISCs. We investigated here whether miRs present in cMV are bound to Ago protein as a RISC, and whether GW182 is associated with Ago and cMV from human plasma and cultured cells. First, we immunoprecipitated Argonaute2 (Ago2) and CD81 (a cMV-specific marker) from purified cMV from cells in culture. Copy numbers for let-7a and miR-16 were determined from these precipitates under both native and lysed conditions. Our data demonstrates that under non-lysed conditions, the vast majority of these 2 miRs were found in the CD81 positive population, with minimal amounts in the Ago2 positive population. However, upon lysis the proportions reversed, and most of the miR was associated with Ago2. These results show that these miRs are loaded into Ago2 on the inside of exosomes. Therefore it is likely that following exosomal endocytosis, these Ago2-miR complexes will be immediately functional and able to inhibit translation of the complementary mRNA. Next, we used antibodies directed toward Ago2 and GW182 to immunoprecipitate the proteins from plasma. A western analysis determined that GW182 and Argonaute co-precipitate, suggesting that these 2 proteins retain their functional relationship in plasma. RNA isolated from the immunoprecipitates showed that the GW182-associated miR profile contained individual miRs whose abundance either equalled or surpassed that of their matched Ago2 immunoprecipitated miRs. These data imply that GW182 maintains an association with the family of Argonaute proteins and a subset of cMV in human plasma.

**Poster Session VI (Statler): Late Breaking April 19**

**Chair: A. Hill and E. Nolte-’t Hoen**

**Exosomal lipids impact on notch signaling to drive differenciated SOJ-6 cells towards apoptosis but promote cell survival by inducing SDF-1 production in cancer stem-like MiaPaCa-2 cells**

Sadia Beloribi and D. Lombardo

INSERM UMR 911 CRO2, Marseille, France

*Introduction*: We showed that exosomes produced by human pancreatic cancer cells induced death of differenciated cancer cells via apoptosis. We demonstrated that cell death occurred when the Notch pathway was abrogated. As exosomal proteins and sugars were not responsible for cell death, we hypothesised that apoptosis depends on exosomal lipids. Plus we thought that the cell sensitivity relies on the markers they express. *Material and methods*: We produced synthetic exosomes-like nanoparticles, or SELN which mimicked lipid composition of exosomes. By using inhibitors, siRNA, WB, FACS, confocal microscopy and qRT-PCR, we tried to understand how SELN drove the well-differenciated human pancreatic cancer SOJ-6 cells to apoptosis, whereas the “cancer stem-like” MiaPaCa-2 cells were resistant. *Results*: We showed that SELN interact with SOJ-6 cells at the level of rafts. This interaction impacts on Notch pathway to perturb the gamma-secretase. NICD the intracellular domain of Notch, being not released may not translocate to the nucleus to promote Hes-1 expression. Besides, this inactivated the PTEN/GSK-3β axis and increased the ratio Bax/Bcl-2 driving cells towards apoptosis. Sensitive SOJ-6 cells express high levels of the members of the Notch pathway, whereas resistant MiaPaCa-2 cells are rich in CXCR-4 receptor and in the Hedgehog pathway members. Indeed, MiaPaCa-2 cells are sentitive to cyclopamine, an Hedgehog pathway inhibitor, but upon SELN incubation they become resistant. We supposed that SELN induce the production of a protective factor and found out that SDF-1, a ligand of CXCR-4 receptor known to be released by the stroma to promote tumor growth, is detected in supernatants of MiaPaCa-2 cells following incubation with SELN. SDF-1 may promote cell survival through the activation of Akt. *Conclusions*: We reported that exosomal lipids were responsible for cell death and we are progressively demonstrated that they affect more pathways than the Notch one depending cell.

**Exosomes as key players in the adipocyte-melanoma cross-talk**

Laurence Nieto, I. Lazar, M. Ducoux-Petit, B. Monsarrat, O. Schiltz-Burlet and C. Muller

IPBS – CNRS, France

During the development of a tumour, a crosstalk takes place between tumour cells and the surrounding microenvironment. As one of the key players in this dialogue the role of adipocytes is receiving increasing attention. Indeed, emerging data indicate that a bidirectional crosstalk is established between cellular components of adipose tissue and cancer cells contributing to inflammation, extra-cellular matrix remodelling and energy supply within tumours. Malignant melanoma results from the transformation of melanocytes which are pigment-producing cells predominantly encountered in the skin. While melanoma is treatable by surgical resection when caught in its early stages, it is the cause of tens of thousands of deaths each year worldwide when diagnosed too late. Little thought has been given to the role of adipose tissue in melanoma progression although it is the main component of the deeper layers of the skin, the hypodermis. Therefore, the bioactive molecules secreted by adipose tissue may contribute to the initiation the vertical growth phase of primary melanomas and support the growth of locally invasive tumours. Cross-talk mechanisms are likely to be multifactorial, involving the release of both soluble factors and possibly exosomes. Adipocytes are obtained from the in vitro differentiation of a murine pre-adipocyte cell line. Several melanoma cell lines are used. Coculture, exosome preparation and characterisation, invasion assays, proteomics studies, are performed as already described. We have shown that conditioned medium from mature adipocytes strongly stimulates the migratory and invasive abilities of melanoma cells, this activity being mediated by exosomes. We have characterised the protein content of exosomes secreted by mature adipocytes cocultivated with cancer cells or cultured alone. We are currently identifying the molecular mechanism which governs this process. Indeed, our work reveals that exosomes are master actors in adipocyte/melanoma dialogue.

**Novel method of exosome quantification and cellular uptake using the Amnis ImageStreamX**

C.A. Franzen, P. Simms, K.E. Foreman and G.N. Gupta

Loyola University Medical Center, United States

*Introduction*: Exosomes contain proteins, mRNA, and miRNA, thus potentially modulating signaling pathways in recipient cells. Moreover, detection and analysis of exosomes are useful for disease diagnosis and treatment. However, their small size makes them difficult to analyse. In this study, we analysed exosomes produced by bladder tumour cells using Amnis ImageStreamX. *Methods*: Amnis ImageStreamX detection limits for beads area and fluorescence were determined with Nano Fluorescent Particle Size Standard Kit. Exosomes were isolated by ultracentrifugation from bladder cancer cell conditioned media, labeled with PKH-26, and analysed on the ImageStreamX with an internal standard added to determine concentration. Exosomes were co-cultured with bladder cancer cells, which were stained for Coxsackie and Adenovirus Receptor, and analysed for internalisation. Using the spot count wizard (IDEAS software), we determined the number of PKH-26+ spots. We measured total uptake using overall PKH-26 fluorescence intensity. *Results*: Exosome uptake increased with longer co-culture times, and, by establishing a dose response curve for uptake, we determined that the lower detection cutoff was 6.4×10^7^ exosomes. The fluorescence intensity of labeled exosomes decreased with overnight storage at 4°C or −20°C, but uptake was not affected. Treatment with trypsin and deconvolution microscopy confirmed that exosomes were internalised. Finally, co-culture at 4°C completely blocked uptake. *Conclusions*: We show here a novel method for quantitating exosomes isolated from cell culture conditioned media. We determined the threshold level of exosomes and optimal incubation time to achieve efficient uptake. We found that storing exosomes overnight did not impact uptake. The exosomes taken up by recipient cells were internalised, as shown by trypsin treatment and deconvolution microscopy. Finally, we found by incubating cells with exosomes at 4°C that uptake is an active and specific process.

**The novel role of mast cell derived exosomes in non small cell lung carcinoma**

L. Li^1^ and H. Xiao^2^

^1^Shanghai Jiao Tong University, China; ^2^ShangHai jiaotong University, China

*Introduction*: Lung cancer is the leading cause of cancer deaths worldwide. Non-small cell lung cancer (NSCLC) accounts for 80% of lung cancer patients and they are usually associated with poor prognosis. Mast cell-derived exosomes play an important role in the development and progression of non-small cell lung carcinoma. Exosomes may represent a vehicle by which one cell communicates with another, actually delivering RNA and, in turn, modulating recipient-cell protein production and adjusting Non-small cell lung cancer cell cycle. This paper explores the role and mechanism of mast cell-derived exosomes in non-small cell lung cancer microenvironment. *Material and methods*: 1. C-kit expression was evaluated in 146 patients with confirmed NSCLC. 2. Exosomes were prepared from the supernatant of mast cells by differential centrifugations. 3. The percentage of cells in different phases of the cell cycle was analysed using flow cytometry analysis. 4. Regulation of cell cycle G1-related proteins was analysed by Western blot. *Results*: 1) The results of an immunohistochemical analysis demonstrated that c-kit is expressed by 33.3% of non-small cell lung cancer. Kaplan-Meier survival analysis and a log-rank test revealed that patients with c-kit expression had a tendency toward lower survival than c-kit-negative patients (median survival, 19 months versus 22months, P=0.048). 2) The electron micrographs of the exosomes revealed rounded structures with a size of approximately 30–100 nm, similar to previously described exosomes. 3) Non-small cell lung cancer cells were serum starved for 48 hr and released into 10% FBS-containing medium for 24hr. Released in the serum at the same time, the influence of mast cell-derived exosomes on the non-small cell lung cancer cell cycle were the number of cells in G1 phase reduced and the S-phase cells increased. It showed that mast cell derived-exosomes would promote cell proliferation. Furthermore, we explored the molecular mechanisms of regulation of the G1 phase protein. *Conclusions*: A novel role for mast cell exosomes may be the delivery of c-kit to lung cancer cells that engulf the vesicle. Mast cells utilise this mechanism to broadcast local environmental signals to tumour cells. This work reveals an intercellular communication pathway whereby mast cell derived exosomes mobilise c-kit/PI3K-Akt signaling to drive lung cancer proliferation. Key word£ Mast cells; exosomes; c-kit; PI3K; non-small cell lung carcinoma.

**Identification of a transformed subpopulation of liver epithelial cell-derived microvesicles that stimulate NK cytotoxicity**

M. Srajer Gajdosik^1^, D. Yang^1^, D. Pantazatos^2^, L. Cao^2^, D. Josic^2^, K.E. Brilliant^1^, D. Chatterjee^2^, P.J. Quesenberry^2^, D.C. Hixson^2^ and D.R. Mills^2^

^1^Rhode Island Hospital, United States; ^2^Rhode Island Hospital/Brown University, United States

*Introduction*: Microvesicles (MV) have been described as playing important roles in cancer progression and regulation of immune cell responses. Here, we examined MVs released from the stem-like liver epithelial line WB-344 (WB), GP7TB (GP7), a transformed line derived from WB, and GP7TB.SA, a subpopulation of GP7 cells isolated in soft agar assays and describe the effect of GP7 and GP7.SA-derived MVs on rat hepatic natural killer (NK) cell activity. *Material and methods*: Proteomic analysis was completed at the Rhode Island Hospital Center of Biomedical Research Excellence (COBRE) Proteomics Core Facility (Providence, RI). Hepatic NK cells were isolated from Fischer rats using antibodies to remove B cells, T cells, monocytes, macrophages and granulocytes. Isolated NK cells were incubated in the presence or absence of cell-derived MVs for 1h at 37°C and NK cytotoxicity was measured against YAC-1 target cells using a 4 h 51Cr-release assay. *Results*: Electron microscopy showed differences in the size (WB>GP7>GP7.SA) and number (WB<GP7<GP7.SA) of shed MVs. Proteomic comparison of WB vs GP7 MV showed 70 proteins unique to the GP7 population mainly associated with cell adhesion and intracellular signaling. Comparison of GP7 vs GP7.SA identified 68 proteins unique to the GP7.SA population mainly involved in regulation of cell death and motility. Necl-5 interacts with CD226 on NK cells to induce cytolytic killing and was found on GP7 and GP7.SA MVs. Pre-incubation of hepatic NK cells with GP7.SA-derived MVs significantly stimulated NK-mediated cytotoxicity. In contrast, MVs purified from GP7 had no effect. *Conclusions*: Changes identified between these related cell lines are reflected in extracellular vesicle release as determined by morphology, proteomics and function. Cytolytic studies show that Necl-5 expression on MVs is not sufficient to modulate NK activity. This model has the potential to identify co-activating molecules that stimulate the NK anti-tumour response.

**Filling the blanks on MP: proposing a color compensation approach for membrane microparticles (MP) analysis on multicolor flow cytometry (FC)**

K. Abuarquob, T. Franks and D. Ramon

University of Michigan, Department of Pathology, United States

*Introduction*: Circulating MP are used to monitor various physiological and pathological processes. FC is the method of choice for MP characterisation, but the lack of standardisation is an obstacle for clinical application. We propose a multicolor MP compensation approach, following what is used for cellular FC. *Material and methods*: Lymphocyte cells were isolated and then incubated with Staurosporine for 10 h/RT. Cells were then centrifuged and supernatant with lymphocyte MP named as MP-Comp was stored at −80°C. In separate tubes, 10 μl of MP-Comp was stained with anti-CD45 conjugated to FITC, PE, PC5 and PC7. 20 μl of MP-Comp was co-stained with anti-CD4-FITC, CD52-PE, CD3-PC5 and CD2-Cy7. Compensation was tested daily, using a BC FC500 4-color compensation protocol. Isolated MP from healthy donors were stained with leukocyte, platelet and endothelial markers, along with Annexin. Fluorescent beads (MegamixTM, sized 0.5, 0.9, 3 μm) were plotted on FS and SS log scales for size identification. MP/μl calculation was established using Flow CountTM beads. MP-Comp performance was evaluated with Levey Jennings charts. The reproducibility of donor samples was calculated by CV%. *Results*: MP-Comp's performance for every tested channel show values within 2 SD on the Levey Jennings charts. Similar results were generated FS and SS. After sample running, the number of each MP population was collected and for the leukocyte MP we obtain a good reproducibility with CV%=7–10%. However, endothelial and platelet MP showed a CV%=19–30%. *Conclusions*: MP detection and quantification by FC not only requires a special calibration for size but also for the different antigen density. Here we propose a compensation approach with a similar size and antigen density as circulating MP. A good reproducibility and CV% is obtained for lymphocyte MP but less ideal for platelet and endothelial MP, and this observation may indicate the need of a specific MP-Comp for each MP population.

**Expression of EGFR in exosomes derived from prostate cancer cell lines, LNCap xenograft serum and patient plasma/serum**

G. Kharmate, E.H. Beheshti and E.S. Guns

Vancouver Prostate Centre, Canada

*Introduction*: Prostate cancer (PCa) is the second leading cause of death in men. Recently our lab published an in depth proteomic analysis of exosomal cargo derived from a panel of PCa cell lines. Membrane associated receptors as mediators of cell growth were highlighted to be important in exosome cargo. However, the role of membrane receptors such as EGFR derived from PCa in cell proliferation and invasion remains elusive. Studies suggest, alterations in EGFR mediated signaling pathways may be associated with hormone refractory PCa. While PCa cells and tissues express EGFR, it is unknown whether exosomes derived from PCa cells and patient serum contain EGFR. The objective of this study is to determine whether the exosomes derived from PCa cell lines, LNCap xenograft serum and patient plasma or serum contain EGFR. *Methods*: Exosomes were isolated from conditioned media from different PCa cell lines (DU145, PC3, LnCap, VCap, C42 and RWPE-1; LNCap xenograft serum as well as patient plasma/serum. Isolation was carried out using differential centrifugation and ultracentrifugation on a sucrose density gradient. Exosomes isolation was validated by TEM, expression of exosomal markers and NanoSight™ analysis. *Results*: TEM confirmed exosomes derived from xenograft and patient plasma/serum. Exosome markers Alix, CD9 or LAMP2 validated isolation. Our results show that exosomes derived from PCa cells, LNCap xenografts and patient plasma or serum express EGFR. Although exosomes from serum are not organ specific, further studies are warranted to determine prostate as the origin of exosomes containing EGFR. Our study also revealed that a sucrose assisted centrifugation method was superior for exosomes isolation as compared to ExoQuick™. *Significance*: This is the first report showing the expression of EGFR in exosomes derived from PCa patient plasma/serum and may provide further insight into the role of EGFR transfer via exosomes in PCa progression.

**Human and murine endothelium derived microparticles contain HMGB-1 but not RAGE**

P. Jeziorczak^1^, S.K. Kaul^1^, E.R.J. Jacobs^2^, K.A.P. Pritchard Jr^1^ and J.C.D. Densmore^1^

^1^Medical College of Wisconsin/Children's Research Institute, United States; ^2^Medical College of Wisconsin, United States

*Introduction*: Although modern ventilator strategies support patients with Acute Respiratory Distress Syndrome (ARDS), no direct therapy is available in clinical practice. Our group has previously demonstrated the role of endothelium-derived microparticles (EMPs) in lung injury pathogenesis using a complete murine model. A putative mechanism for this injury is the activation of the Receptor of Advanced Glycation Endproducts (RAGE) by high-mobility group box-1 (HMGB-1). We hypothesise that human and murine endothelial cell lines (HUVEC and MS-1) contain HMGB-1 and RAGE, that EMPs contain HMGB-1 but not RAGE, and that blockade of HMGB1 with glycyrrhizic acid and HMBG1-binding heptamer peptide (HBHP) will improve endothelial barrier integrity. *Material and methods*: Mile Sven 1 (MS-1) and Human Umbilical Vein Endothelial Cells (HUVECs) were stimulated with murine plasminogen activation inhibitor-1 (PAI-1, 50 ng/mL and 10 ng/mL respectively) to generate mouse and human EMPs. Western blot analysis with normalisation to β-actin was used to determine the presence of RAGE and HMGB-1. To assess HMGB1 contribution to pulmonary capillary leak, transendothelial resistance over a human pulmonary artery endothelial cell (HPAEC) confluent monolayer was measured continuously for 16 hours with and without HMGB1 (20 μg/mL), glycyrrhizic acid (GA, 840 μg/mL), and HBHP (8 μg/mL). *Results*: Both murine and human EMPs contain HMGB-1, but lack full length and soluble RAGE. MS-1 cells and HUVECs both contain RAGE and HMGB-1. HMGB-1 does induce HPAEC permeability and is partially reversed by cotreatment with inhibitors GA and HBHP. *Conclusions*: Endothelium derived microparticles contain HMGB, but not RAGE. This pattern is preserved between murine and human EMPs. Interestingly, the cell lines of origin contain both RAGE and HMGB-1, indicating a selective inclusion of HMGB-1 in EMPs for propagation of inflammatory response. EMPs and HMGB-1 are therapeutic targets for the treatment of ARDS.

**Endothelial exosomes targeting endothelia in vivo**

A. Banizs^1^, T. Huang^2^, J.S. Lee^3^, W. Shi^2^ and J. He^2^

^1^University of Virginia, United States; ^2^Department of Radiology/University of Virginia, United States; ^3^Department of Radiology/University of Virginia, United States

*Introduction*: The objective of this study is to evaluate exosomes from endothelial cells for foreign nucleic acid delivery and in vivo biodistribution. *Material and methods*: Exosomes originating from endothelial cells from mouse aorta were characterised by transmission electron microscopy and ELISA. FACS studies evaluated the interaction between exosomes and primary endothelial cells through DiO fluorescent dye labeling of exosomes. Gene silencing effect of exosomes/siRNA targeting luciferase was performed in transfected endothelial cells. 18F isotope labeled exosomes were used for microPET imaging and to quantitatively evaluate the biodistribution. *Results*: TEM and ELISA confirmed the quality, quantity and identity of exosomes. DiO labeled exosomes were able to bind and internalise endothelial cells. Exosomes loaded with siRNA against luciferase were able to reduce luciferase level in primary endothelial cells. The biodistribution study showed that endothelial exosomes were rapidly cleared from circulation, accumulated at remarkable level in organs like heart and brain, while kidneys and liver demontrated less activity. *Conclusions*: These results suggest that endothelial exosomes can deliver foreign molecules into endothelial cells in vitro and target endothelia in vivo, therefore, they have potential as delivery vectors.

**Differential activity of soluble and vesicle-associated ligands for the activating NK cell receptor NKp30 in chronic lymphocytic leukemia**

K.S. Reiners, H.P. Hansen and E. Pogge von Strandmann

University Clinic Cologne, Germany

*Introduction*: Natural killer (NK) cells are a major component of the anti-tumour immune response. However, NK cell dysfunction has been reported in various hematological malignancies, including chronic lymphocytic leukemia (CLL). NK cell activity is regulated by a set of activating and inhibiting receptors that are engaged by target cell-derived ligands. Here, we compared the influence of tumour cell-derived soluble and vesicle-associated ligand BAG6(BAT3) engaging the activating NK cell receptor NKp30 on NK cells in CLL. *Material and methods*: We quantified soluble and vesicle-associated BAG6 in the serum of CLL patients and healthy donors and analysed the release of BAG6 from tumour cell lines and primary CLL cells. Primary NK cells were incubated with purified BAG6-positive vesicles or supernatant with soluble BAG6. The NK cell activation was determined by FACS by measuring the activation marker CD69 and NK cell receptors, which are involved in target cell recognition. The NK cell-mediated cytotoxicity against tumour cells was measured in europium release assays. *Results*: Incubation of healthy NK cells with soluble CLL plasma factors suppressed NK cell cytotoxicity and down-regulated the surface receptors CD16 and CD56 on NK cells. This inhibition could be attributed to soluble BAG6 that was detectable in the plasma of CLL patients, with the highest levels at advanced disease stages. In contrast, NK cells were activated when BAG6 was presented on the surface of exosomes. The latter form was released by non-CLL cells via an nSmase2-dependent pathway after cellular stress. Such cells were eliminated by lymphocytes in a xenograft tumour model in vivo. Here, exosomal BAG6 was essential for tumour cell killing because BAG6-deficient cells evaded immune detection. *Conclusion*: Taken together, the findings show that the dysregulated balance of exosomal versus soluble BAG6 expression may cause immune evasion from NK cell immunosurveillance in CLL (Reiners, Blood, Epub 3/2013).

**Catabolic effects of endothelial cell-derived microparticles on intervertebral disc cells**

P.H.I. Pohl^1^, T. Cuperman^2^, T. Lozito^2^, H.J. Moon^1^, K. Ngo^1^, T. Yurube^1^, G. Sowa^1^, R. Tuan^2^, J.D. Kang^1^ and N. Vo^1^

^1^Department of Orthopaedic Surgery, University of Pittsburgh Medical Center/The Ferguson Lab For Or, United States; ^2^Department of Orthopaedic Surgery, University of Pittsburgh Medical Center/Center of Cellular and, United States

*Introduction*: Neo-vascularisation, a prominent feature of injured and degenerated discs, requires the invasion of endothelial cells, the primary producer of microparticles (MPs). MPs, also known as microvesicles or exosomes, are 0.1 to 1 μm particles shed from cell plasma membrane blebs in response to stimulus or stress. MPs consist of membrane and cytoplasmic subcomponents along with a number of proteins and extracellular RNA characteristics of their parental cells. Emerging evidence suggest that MPs are biologic information messengers with important roles in both in pathological and non-pathological processes. Because the interaction between endothelial MPs and disc cells are likely to occur during neovascularisation, the objective of this study is to evaluate the influence of the endothelial MPs on disc cells metabolism. *Material and methods*: Endothelial microparticles (EMPs) were isolated as described (J Cell Physiol. 2012 227:534–49) from the culture media of a microvascular endothelial cell line grown on monolayer culture for two weeks. 15 μl of 14mg/ml EMPs (total protein) were added to each well of the 6-well plate of human annulus fibrous (hAF) cell cultures, and the cultures were incubated for 3 days. Gene expression of MMP-1, MMP-2, MMP-3, MMP-13, VEGF, collagen type I and aggrecan were determined by qRTPCR. *Results*: Increased gene expression of MMP-1 (41.8-fold), MMP-3 (5.1-fold) and MMP-13 (6.2-fold) were observed in EMP-treated hAF cells compared to untreated hAF cells. In contrast, aggrecan gene expression decreased in EMP-treated hAF cells compared to untreated hAF cells. *Conclusions*: Endothelial cell microparticles promote catabolic metabolism in disc cells. If confirmed in vivo, this could represent a new mechanism by which disc extracellular matrix is degraded during the degenerative process.

**A nanotube and vesicle model of cell adhesion and intercellular communication between NK and susceptible target cells**

Viladimir Kuznetsov

Bioinformatics Institute, Singapore

Natural killer(NK) cells are the major cellular effectors in the innate immune system, which function to abrogate a variety of 'susceptible' target cells (TC) including infected and transformed cells. These cells respond “spontaneously” against their TC. The roles of multiple activating receptors in NK cells communication with the TC are not yet clearly understood. In our early studies (Kuznetsov, 1991, 1996), we have proposed the ‘harpoon’ model of NK-cell recognition which suggested a central role of intercellular contacts and small vesicle's traffic from the NK surface to TC surface (and vice versa). The model permitted the transfer of many types of receptor and counter-receptor molecules from the surface of one conjugated cell to another by vesicles or membrane fragments. Susceptibility of TC has been explained by a presence of multiple disorders in the organisation of extracellular matrix-surface membrane-submembrane and cytoskeleton protein assembly of the NK-sensitive TCs. After transferral through the intercellular cleft, the free NK receptors and counter-receptors could be localised on both cell surfaces at the contact region between conjugated cells. By this model the NK cell can “harpoon” the TC and enhance the binding forces between cells up to the critical level and then switch on killing mechanisms for the TC. Recently, NK cells have been shown to form membrane nanotubes over long distances, which physically link cell bodies can traffic vesicles (Chauveau et al, 2010) and aids TC lysis. These findings support of our model and help better understand the nature of the wide polymorphism of TCs. A mathematical model of the NK cell binding and cytotoxic reaction is described and used to interpret several nonlinear peculiarities of the NK cytotoxic response and predicts some new phenomena. We suggest new approaches of manipulation of cell membranes and extracellular matrix which might transform NK-resistant target cells in NK sensitive cells and vice versa.

## Scientific Program 2013 ISEV meeting Saturday 20th April

**Parallel Oral Sessions 28-30 8:30-10:00**

**Oral Session 28 (Imperial Ballroom): Cancer: resistance and metastasis April 20**

**Chair: *H. Peinado and M. Belting* 8:30-10:00**

**Microparticle-mediated transfer of ABC drug efflux transporters confers cancer multidrug resistance**

M. Bebawy^1^, R. Jaiswal^2^, J. Gong^3^, J. Lu^3^, P. Dalla^3^, C.L. Luk^3^ and G. Grau^4^

^1^School of Pharmacy, Graduate School of Health, Australia; ^2^The University of Technology Sydney, Sydney, Australia; ^3^The University of Technology, Sydney, Australia; ^4^Sydney Medical School, Australia

*Introduction*: Drug resistance is a major cause of cancer treatment failure with multidrug resistance (MDR) being the most serious trait. In MDR, cancer cells display a broad cross-resistance to unrelated drugs by virtue of the overexpression of the efflux transporters P-glycoprotein (P-gp) and multidrug resistance-associated protein 1 (MRP1). Recently, we discovered a novel non-genetic basis to MDR, whereby microparticles (MPs) transfer P-gp intercellularly from MDR donor cells to drug-sensitive recipient cells. We describe the contribution of MPs in the transfer of cellular cargo in the occurrence and stability of cancer MDR in vitro and in vivo. *Methods*: MPs isolated from MDR cancer cells were co-cultured with their drug-sensitive counterparts. MP mediated interactions were demonstrated by flow cytometry, confocal imaging and scanning electron microscopy. Functional P-gp and MRP1 transfer was assessed by direct immunolabelling and drug exclusion assays, whereas acquired transcripts and regulatory microRNAs were assessed using microarray and quantitative real time PCR. MP-mediated P-gp transfer to sensitive cells was also shown in vivo using an MCF-7 murine tumour xenograft model. *Results*: We show that MPs isolated from MDR cells carry and transfer functional resistance proteins, nucleic acid species and machinery for MP and miRNA biogenesis within their cargo. We also show that MPs “re-template” recipient cells’ to reflect the donor cell phenotype as early as 4 hours following MP exposure. We also show that P-gp was acquired and penetrates to the core of the recipient drug-sensitive tumours when exposed to a single s.c MP dose in vivo. This acquired trait was stable for at least 2 weeks post MP exposure. *Conclusions*: We demonstrate a novel cancer MDR pathway mediated by MPs. This has enormous implications in the dissemination and acquisition of cancer traits. This provides a novel therapeutic target in clinical oncology.

Funded by: The New South Wales Cancer Council and National Health and Medical Research Council.

**The long non-coding RNA-ROR is increased in tumour cell exosomes and mediates chemoresistance in hepatocellular cancer**

K. Takahashi, I. Yan, H. Wen and T. Patel

Mayo Clinic, USA

*Introduction*: Hepatocellular cancers (HCC) are highly resistant to chemotherapy. We have shown that HCC cells can release extracellular vesicles containing microRNA that can modulate cell signalling pathways. Long non-coding RNAs (lncRNAs) are being implicated in disease pathogenesis, but their role in HCC is unknown. Thus, our aims were to evaluate the involvement and contribution of extracellular vesicle lncRNA in mediating innate chemoresistance in HCC. *Materials and Methods*: Malignant (HepG2, Hep3B, HepG2ST, Huh7 and PLC) or non-malignant hepatocytes were used. Expression of selected lncRNA or mRNA was performed using qRT-PCR. Extracellular vesicles were isolated by ultracentrifugation, and exosomal content verified by density gradient centrifugation, nanosight and EM. Cell viability and apoptosis were examined. Chemotherapeutic stress was induced by sorafenib, campothecin or doxorubicin. siRNA was used to modulate lncRNA expression. *Results*: lncRNA expression profiling identified 21 lncRNA that were increased (>2 log fold, p<0.05) in HepG2 HCC cells compared to non-malignant hepatocytes. Of these, lncRNA-ROR was amongst the top most significantly up-regulated, and furthermore its expression was also increased by 1.7 to 4.8-fold in a panel of HCC cells. lncRNA-ROR was increased in HepG2 derived exosomes compared to their cells of origin. In HepG2 cells and exosomes, lncRNA-ROR expression was increased by sorafenib. siRNA to lncRNA-ROR increased sorafenib (1 μM, 24 h) induced apoptosis and decreased cell viability in response to 1–100 μM sorafenib, doxorubicin or camptothecin compared to controls. Expression of caspase8, GSTP1 and GADD45B were increased >2-fold, whereas Bcl-2 expression was decreased by siRNA to lncRNA-ROR. Incubation with exosomes increased lncRNA-ROR expression and reduced chemotherapy-induced cell death in recipient cells. *Conclusions*: These data provide mechanistic insights into primary chemoresistance in HCC by showing that (a) lncRNA-ROR is highly upregulated, expressed in response to chemotherapeutic stress and plays a functional role in chemoresistance and (b) lncRNA-ROR in exosomes could modulate chemosensitivity. These findings implicate exosome-mediated intercellular signalling by lncRNA as mediators of chemotherapeutic response.

**Microvesicles shed from gefitinib-resistant non-small lung cancer cells regulate the tumour microenvironment through AKT/mTOR signalling**

D.Y. Choi^1^, S. You^2^, J. Jung^1^, M.R. Freeman^2^, K.P. Kim^1^ and J. Kim^2^

^1^Konkuk University, Republic of Korea; ^2^Cdars-Sinai Medical Center, Los Angeles, CA, USA

Lung cancer is the most common cause of death from cancer worldwide. The epidermal growth factor receptor (EGFR) is expressed in up to 80% of non-small cell lung cancers (NSCLC). EGFR-tyrosine kinase inhibitors (TKIs), including gefitinib, have been used for NSCLC patients with frequent recurrence due to chemoresistance. The most common mechanism of acquired drug resistance gefitinib is the secondary EGFR T790M mutation, which is detected in up to 60% of EGFR mutant NSCLC patients. The EGFR mutant PC9 cell line harbouring EGFR T790M, PC9R, has been used as in vitro model of drug resistance. Tumour microvesicles (TMVs) shed from cancer cells contain genetic and other molecular information that might be useful for diagnostic purposes to differentiate gefitinib-resistant NSCLC (non-responders) from sensitive patients (drug responders). The main objective of this study was to identify unique protein and phospholipid signatures of TMVs derived from gefinitib-resistant PC9R cells by proteomics analysis and lipid MALDI profiling. Using nano-LC–MS/MS, we identified 664 TMV proteins enriched in PC9R cells and 338 TMV proteins enriched in PC9 cells with high confidence (FDR<0.05, fold change ≥2), revealing pathologically relevant and potential diagnostic marker proteins for gefitinib-resistant NSCLC. Relevant cellular processes identified by bioinformatics analyses include AKT1/mTOR and NF-κB signalling, drug response, mitotic cell cycle cytoskeleton regulation. TMV harvested from PC9R cells increased proliferation and invasion of recipient tumour cells. Moreover, lipid MALDI profiling suggests that unique membrane phospholipids reside in secreted TMV. Our findings provide new insight into a TMV signature consisting of unique oncoproteins and phospholipids, and will aid in the development of novel diagnostic and treatment strategies for gefinitib-resistant NSCLC.

**Exosomes contribute to radiation-induced cell migration**

W. Tristram Arscott^1^, A. Tandle^1^, S. Zhao^1^, J. Shabason^1^, I. Gordon^1^, C. Schlaff^1^, G. Zhang^2^, P. Tofilon^1^ and K. Camphausen^1^

^1^NCI/NIH, USA; ^2^NIBIB/NIH, USA

*Introduction*: Exosome production is a ubiquitous process augmented by cellular stress, including irradiation. Recent studies highlight the ability of exosomes to convey information to cells through DNA, RNA and protein, and their signalling appears to play an important role in cancer progression and response to treatment. In this study, we explore the radiation influence on exosome secretion and signalling. *Materials and Methods*: Nanoparticle tracking analysis (NTA) was used to quantify exosome abundance following irradiation. PKH26-labelled exosomes enabled the study of exosome uptake in co-culture. Transwell assays were used to study exosome influence on cell migration and invasion. cDNA, miRNA and protein arrays, combined with Ingenuity Pathway Analysis software, were used to determine the molecule profile of exosomes. Comparisons were made between exosomes derived from irradiated cells (post-radiation exosomes) and those from non-irradiated cells. *Results*: Irradiation of various glioma cell lines and normal astrocytes increased exosome abundance as measured by NTA. Post-radiation exosomes were more readily taken up by cells in co-culture, and enhanced the migration and invasion of cells upon uptake. Molecular profiling of post-radiation exosomes revealed an increase in targets related to signalling pathways involved in cell migration, including CTGF mRNA and IGFBP2 protein, which are known mediators in glioma cell migration. Co-culture of post-radiation exosomes resulted in increased CTGF protein by the recipient cells, as well as enhanced activation of TrkA, FAK, Paxillin and Src. *Conclusion*: Our data demonstrate that radiation influences exosome abundance and alters exosome composition and signalling in target cells upon uptake, promoting an invasive phenotype. Further characterisation of this effect in gliomas and other cancers is warranted to determine if targeting exosomes may be a viable approach for cancer therapy.

**Heparanase regulates secretion, composition and function of tumor cell-derived exosomes**

Camilla Thompson, A.P. Purushothaman, V.C.R. Ramani and R.D.S. Sanderson

University of Alabama, Birmingham, United States

*Introduction*: Emerging evidence indicates that exosomes play a key role in mediating tumor-host crosstalk and that exosome secretion, composition and function are altered as tumors progress to an aggressive phenotype. The underlying mechanisms for this are unknown. Heparanase is an enzyme whose expression is associated with enhanced tumor growth, angiogenesis and metastasis. *Materials and methods*: Human cancer cell lines were exposed to exogenous heparanase or transfected with either a vector enhancing heparanase expression (heparanase-high), a control vector (heparanase-low) or a vector containing a mutated enzymatically inactive form of heparanase. Exosomes were isolated from medium conditioned by cell lines and subjected to western blotting, protein quantification, ELISAs, or used in function assays. To determine composition, equal amounts of exosomes from heparanase-high and heparanase-low cells were used in ELISAs. To determine function, equal amounts of exosomes were tested for their ability to stimulate tumor cell spreading or endothelial cell invasion. *Results*: Enhanced heparanase expression in cancer cells, or exposure of cells to exogenous heparanase dramatically increased exosome secretion. This requires the enzymatically active form of the enzyme. Heparanase also impacts exosome protein cargo as reflected by higher levels of syndecan-1, VEGF and HGF in exosomes secreted by heparanase-high expressing compared to heparanase-low expressing cells. In functional assays, exosomes from heparanase-high cells stimulated spreading of tumor cells on fibronectin and invasion of endothelial cells better than did exosomes from heparanase-low cells. *Conclusions*: These studies reveal a key role for heparanase in regulating exosome secretion, composition, and biological activity. Therapeutic targeting of heparanase in cancer thus has the potential to interfere with exosome-mediated tumor-host crosstalk thereby blocking tumor progression and metastasis.

**Fibroblast exosomes mobilise autocrine Wnt-planar cell polarity signalling in breast cancer metastasis**

Valbona Luga^1^, L. Zhang^2^, A.M. Viloria-Petit^3^, A.A. Ogunjimi^2^, M.R. Inanlou^2^, E. Chiu^2^, A.N. Hosein^4^, M. Buchanan^4^, M. Basik^4^ and J.L. Wrana^2^

^1^University of Toronto, Toronto, Ontario, Canada; ^2^Samuel Lunenfeld Research Institute, Toronto, Ontario, Canada; ^3^University of Guelph, Guelph, Ontario, Canada; ^4^Lady Davis Institute for Medical Research, Montreal, Quebec, Canada

*Introduction*: Cancer-associated fibroblasts (CAFs) play a critical role in tumour progression. However, the cross talk between tumour cells and CAFs in metastasis is poorly understood. The planar cell polarity (PCP) pathway regulates tissue polarity in a wide array of developmental processes, including convergent extension movements, and adult homeostasis. Although several PCP pathway components are overexpressed in cancer and regulate tumour cell growth and migration, it is unclear whether PCP signalling is directly involved in cancer metastasis. *Materials and methods*: Breast cancer cells (BCCs) were treated with conditioned media (CM) produced by a mouse fibroblast cell line (L cells), or human CAFs and were tested for protrusive active and motility in vitro. Next, BCCs alone or together with L cells were orthotopically injected in mice, which were examined for tumour growth and lung metatases. L cell-secreted factors were identified by subjecting the CM to protein chromatography and MS analysis. Fibroblast exosomes were isolated on a sucrose gradient after differential ultracentrifugation or by immunocapture and then analysed by MS, EM and IB. The expression of core PCP components, Fzd, Dvl, Vangl and Pk, and Wnt ligands in BCCs and the exosome component Cd81 in L cells was downregulated by RNAi and the effect in BCC motility and metastasis was determined. Finally, the association of BCC-produced Wnt11 with Cd81-positive exosomes was determined. *Results*: Fibroblast exosomes promote BCC protrusions, motility and metastasis via Wnt-PCP signalling. In mice, co-injection of BCCs with fibroblasts dramatically enhances metastasis that is dependent on Pk1 in BCCs and Cd81 in fibroblasts. Finally, BCC-produced Wnt11 tethers to fibroblast exosomes upon their internalisation by BCCs. *Conclusion*: Our work reveals an intercellular communication pathway, whereby fibroblast exosomes are internalised by BCCs and mobilise autocrine Wnt-PCP signalling to drive BCC migration and metastasis.

**Oral Session 29 (Plaza Ballroom): Therapy: targeting and loading April 20**

**Chair: *D. Gupta and P. Guo* 8:30-10:00**

**Cancer immunotherapy and exosomes**

Margot Zöller, D. Zech and X. Gu

Tumour Cell Biology, University Hospital of Surgery, Germany

*Introduction*: Tumour exosomes being reported to suppress or promote a cancer-directed immune response, we evaluated, whether and which steps in immune response induction can be affected by tumour exosomes and how impaired responsiveness can be circumvented. *Material and methods*: Culture derived exosomes from rat and mouse solid tumours and a mouse leukemia were analysed in vitro and in vivo for uptake by leukocytes, impact on immune response induction and potential therapeutic efficacy. *Results*: Tumour exosomes bind to and are taken up by all leukocyte subpopulations in vivo and in vitro, uptake by macrophages and dendritic cells (DC) exceeding that by lymphocytes. Tumour exosomes slightly affect T cell activation and expansion accompanied by reduced TCR complex initiated signal transduction. Tumour exosomes did not affect DC maturation, had no impact on regulatory T cells and only in vivo led to a slight expansion of myeloid-derived suppressor cells. In vitro, tumour exosomes promoted NK and CTL activity. In vivo, initiation of tumour growth was slightly accelerated, when animals received tumour exosomes concomitantly with tumour cells, tumour growth acceleration being waved in animals receiving a DC vaccine. However, when DC were loaded with tumour exosomes, tumour growth was significantly retarded, the therapeutic efficacy of tumour exosome-loaded DC exceeding that of tumour antigen-loaded DC. The DC-exosome vaccination-induced effect was tumour specific as revealed by trogocytosis, antigen-specific proliferation and CTL activity. We are currently evaluating the path of exosome uptake-induced DC activation and antigen presentation. *Conclusion*: In 3 models of solid tumours and a leukemia, we did not observe severe hindrance of immune response induction by tumour exosomes. Instead, in tumour-bearing mice exosome-treated DC induced a superior response compared to tumour antigen-/lysate-loaded DC. Whether the pronounced immune response is due to exosomes providing a more complete sour.

**Non-invasive in vivo imaging, tissue biodistribution and clearance analyses of intravenously administered extracellular vesicles**

C.P. Lai, O. Mardini, C.A. Maguire, B.A. Tannous and X.O. Breakefield

Massachusetts General Hospital, Harvard Medical School, Boston, MA, USA

*Introduction*: Recent studies have shown promising results in using extracellular vesicles (EVs) as therapeutic vehicles for targeted delivery, thereby overcoming biological barriers such as immune rejection and the blood–brain barrier. Yet, much remains unknown about the in vivo properties of EVs such as tissue distribution, and blood/urine clearance – important parameters that will define their therapeutic effectiveness and potential toxicity. *Material and methods*: To spatiotemporally detect and track EV in vivo, a novel EV reporter was developed by engineering a membrane-bound (mb) variant of the naturally secreted Gaussia luciferase (Gluc) fused to a transmembrane version of a biotin acceptor peptide (BAP), termed mbGluc-BAP. *Results*: EVs isolated from HEK 293T cells expressing mbGluc-BAP revealed a significant amount of mbGluc and biotinylation. Importantly, the mbGluc component of the reporter is active on EVs and exhibited a significant bioluminescence signal upon addition of its substrate coelentrazine (CTZ). Upon i.v. injection of nude mice with EVs carrying the mbGluc-BAP followed by CTZ, real-time localisation of EVs in organs including the liver and the kidneys was detected using in vivo bioluminescence imaging. To further determine EV biodistribution, clearance, and half-life, organs were harvested at different time points after EV i.v. administration. A dynamic EV distribution across organs was observed with the liver and kidneys exhibiting the highest Gluc signal, indirectly correlating with EV levels. Notably, EVs have a short half-life of <30 min in most tissues while most EV signal was cleared from circulation by the kidneys in 6 h with a half-life of <30 min. *Conclusions*: Efforts are currently underway in using clinically relevant imaging agents coupled to streptavidin to further track EVs in vivo. These findings will enable a better understanding of EV's role in different fields, including cancer and neurological diseases, as well as their potential use in clinical therapy.

**Unique roles for cytoplasmic actin isoforms in mechanically regulating endothelial microparticle formation**

S. Latham^1^, C. Chaponnier^2^, V. Dugina^2^, P.O. Couraud^3^, G.E.R. Grau^1^ and V. Combes ^4^

^1^The University of Sydney, Sydney, Australia; ^2^Universite de Geneve, Geneva, Switzerland; ^3^INSERM/Institut Cochin, France; ^4^The University of Sydney/La Jolla Infectious Disease Institute, La Jolla, CA, Australia

Elevated levels of endothelial microparticles (MP) are observed in numerous diseases, supporting their role as effectors and marker of vascular dysfunction. Whilst various cellular components, including the actin-cytoskeleton, have been implicated in MP production, their precise mechanisms of action are not fully understood. We examined vesiculation of human cerebral endothelial cells, in response to various agonists, including *Plasmodium falciparum*-infected erythrocytes. All agonists significantly increased MP release, as seen by flow cytometry and scanning electron microscopy (SEM). These data provide new insight into in the kinetics, patterns of vesiculating cell recruitment and degrees of response specific to each stimulus. Cell stimulation induced reorganisation of cytoplasmic α- and g-actins, F-actin, vinculin and talin. Their subcellular localisations were defined by confocal microscopy and western blot analyses showed that their expression varied between MP and their mother cell. α-Actin redistribution into basal stress fibres after stimulation was associated with increased apical actin-rich particulate structures whose distribution correlated with electron-lucent membrane protrusions observed by SEM. Three-dimensional structured illumination microscopy also provided information on the actin organisation within the purified MP. Pharmacological inhibition of the Rho-kinase pathway with Y-27632 abolished basal α-actin stress fibre formation, minimising apical actin-rich structures, significantly reducing membrane protrusions and MP release to near basal levels. siRNA experiments demonstrate that selective silencing of b-actin, but not of g-actin, prevents TNF-stimulated cells from releasing higher MP levels. Our data strongly suggest that the actin cytoskeleton plays more than a contractile role in vesiculation, with novel actin-rich structures increasing plasma membrane disruption following activation due to their mechanical interaction with basally localised α-actin.

**Comprehensive deep-sequencing analysis reveals non-random small RNA incorporation into tumour exosomes and biomarker potential**

D. Koppers-Lalic^1^, M. Hackenberg^2^, M.E. van Eijndhoven^1^, Y. Sabogal Pineros^1^, D. Sie^1^, B. Ylstra^1^, J.M. Middeldorp^1^ and D.M. Pegtel^1^

^1^VUmc, The Netherlands; ^2^University of Granada, Spain

*Introduction*: The packaging and export of small non-coding RNAs (sncRNAs) through endo-exosomal pathway and their delivery to recipient cells is emerging as an important process in gene regulation and intercellular communication. However, these mechanisms are not well understood. Viral infection and malignant transformation strongly impact the cellular assortment of sncRNAs that could provide mechanistic clues into ncRNA release. We proposed that the exosomal sncRNA content might change upon an altered cellular state. *Methods/Results*: We performed a comprehensive deep-sequencing of exosomal sRNA that originate from Epstein Barr virus-infected B cell lines and associated B cell lymphomas. We identified all annotated sRNA classes (~17 defined species) although RNA Pol III transcripts such as Y RNAs and vault RNAs in general dominate the exosomal small RNA pool. Using sensitive stem-loop RT-PCRs, we confirmed those transcripts in exosomes produced by cell lines and importantly, in exosomes in vivo. Our data shows that in cells under stress specific class of tRNA-derived sRNA fragments (tRFs) are produced. Identified tRFs are specifically abundant in exosomes that originate from lymphomas, implying that cellular stress induced by malignant transformation is evident in extracellular material. Seemingly exosomal miRNAs are a reflection of the cellular abundance; however, close examination indicated that selection of exosomal miRNAs is associated with their 3'-end heterogeneity (isomiRs), reinforcing the idea that 3' modification of sRNAs is biologically relevant. In fact this post-transcriptional modification affected sorting of all sRNAs. *Conclusion*: Collectively our findings indicate that small ncRNA release via exosomes occurs in a non-random fashion that is likely linked to cytoplasmic gene-regulatory mechanisms. We conclude that a sorting mechanism for sncRNA incorporation into exosomes exists and that exosomal sncRNAs are useful as biomarkers.

**Loading of siRNA into extracellular vesicles by electroporation is accompanied by extensive siRNA aggregate formation**

S.A.A. Kooijmans^1^, M.J.A. Wood^2^, R.M. Schiffelers^1^ and P. Vader^2^

^1^University Medical Center Utrecht, The Netherlands; ^2^University of Oxford, UK

*Introduction*: The use of extracellular vesicles (EVs) as drug delivery systems is hampered by the lack of techniques to quantitatively load EVs with nucleic acids. We characterised the use of electroporation for the loading of siRNA into EVs, which was described previously (1,2). Materials and methods: EVs were isolated from HEK293T or Neuro2A cells, mixed with siRNA in Optiprep electroporation buffer, and electroporated as described before (1,2). Aggregate formation was analysed by NTA, and loading efficiency was determined by fluorescence spectroscopy (FS) and quantitative reverse transcription PCR (RT-PCR). *Results*: Electroporation of EVs with fluorescently labelled siRNA resulted in loading efficiencies of 20-25% when determined by FS, or 4% when determined by RT-PCR. Remarkably, electroporations performed in the absence of EVs resulted in a similar or even higher siRNA “loading efficiency”. NTA and light microscopy showed extensive formation of insoluble siRNA aggregates ranging from 50 nm to 10 µm in size after electroporation, which could be dramatically reduced by addition of 1 mM EDTA to the electroporation buffer. FS revealed that EDTA decreased siRNA precipitation to 3% in the absence of EVs, and even further in the presence of EVs (1%). Encapsulation in both scenarios was lower than 0.1% when analysed by RT-PCR. Other strategies to reduce aggregate formation, e.g. the use of cuvettes with conducting polymer electrodes, also dramatically reduced siRNA precipitation. When EVs were electroporated with siRNA under these conditions, loading efficiency was below 0.05%. *Conclusions*: Our results show that electroporation of EVs with siRNA is accompanied with extensive siRNA aggregate formation, which may cause severe overestimation of the amount of siRNA actually loaded into EVs. The low loading efficiency under precipitate-reducing conditions highlights the necessity for more efficient loading methods.

References

1. Wood et al. Nat Biotechnol 2011.

2. Wood et al. Nat Protoc 2012.

**In-membrane self-assembly and formation of exosome-like aggregates-new insights into peptide–cell interactions**

James Liang, L. Chen and N. Patrone

Stevens Institute of Technology, Hoboken, NJ, USA

*Introduction*: The interaction of peptides with cells has been well studied. The electrostatic attraction of cationic peptides to the cell membranes is believed to be the critical step which explains why bioactive peptides are positively charged, with very few exceptions. We know that peptide self-assembling into aggregates in high concentration solutions with defined structure is a common phenomenon. Because peptide binding to cells is several hundred times concentrated on cell membranes, peptide self-assembly may also happen on cell membranes. *Materials and methods*: Interactions of a fluorescence-labelled peptide, PTP-7b, (FITC-FLGALFKALSHLL) with live human cells were studied using confocal microscopy and electron microscopy. *Results*: Interactions of PTP-7b with cell membranes included 3 steps: PTP-7b peptides were attracted to negatively charged cell membranes, bound to cell surfaces and inserted into the plane of cell membranes. The bound PTP-7b peptide could migrate along the cell surface to self-assemble into aggregates at certain locations on cell membranes. As the peptide aggregates became larger, they inserted deep into cell membranes and formed cross membrane aggregates. Because of the difference between intracellular and extracellular environments, the intracellular parts of the PTP-7b aggregates were dissolved and released PTP-7b into the cytoplasm. In the late phase, negatively charged and polar lipid molecules in cell membranes participated in PTP-7b self-assembly on cell membranes and formed exosome-like aggregates composed of a mixture of peptide and lipids. As lipid molecules were continuously extracted into the exosome-like aggregate from cell membranes, the integrity of the cell membranes was disrupted and led to a rapid cell lysis eventually. *Conclusions*: Peptide self-assembly might be equally as important as the charges and secondary structures of peptides and contribute greatly to the biological activity of therapeutic peptides.

**Oral Session 30 (Terrace Room): Neurodegeneration April 20**

**Chair: *L. Rajendran and X. Breakefield* 8:30-10:00**

**Characterisation of amyloid-associated structures at the surface of exosomes**

G. van Niel^1^, C. Fort^1^, A. Di Cicco^2^, I. Hurbain^1^, L. Rochin^1^, G. Raposo^1^ and D. Levy^2^

^1^Institut Curie/CNRS-UMR144, Paris, France; ^2^Institut Curie/CNRS UMR168, Paris, France

Amyloids are insoluble proteins or peptide aggregates with a cross β-sheet structure and are often associated with pathological conditions, including Alzheimer's disease and prion diseases. Amyloids also have a normal biological function as demonstrated by the functional amyloids derived from the protein PMEL, that act as structural scaffolds for melanin deposit in melanosomes of pigment cells. PMEL amyloidogenesis occurs in specific intracellular compartments, i.e. multivesicular bodies (MVBs), where PMEL is sorted and processed on intraluminal vesicles (ILVs). Our previous electron tomography reconstruction of melanosomes supports the hypothesis that ILVs are the site of nucleation of amyloid fibrils. To investigate the formation of amyloids at the surface of ILVs, we have analysed the structure of secreted ILVs as exosomes from PMEL-expressing cells by cryo-electron microscopy (cryo-EM). Our observations at a close to native state by cryo-EM revealed unreported multilayer structures on the surface of exosomes that are reminiscent of reported structures of synthetic amyloid oligomers. The formation of these structures correlates with the presence and the processing of amyloidogenic domains of PMEL, indicating that they may represent the first description at nanoscale of amyloid formation in vivo from the surface of exosomes. These data provide further insight into the role of MVBs and exosomes in both functional and pathological amyloidogenesis, as pathological amyloidogenic substrates (prion, α-synuclein, Tau and APP) have also been reported on exosomes.

**Exosome-mediated transfer of luminal and cytosolic amyloids**

Lawrence Rajendran

University of Zurich, Zurich, Switzerland

Neurodegenerative diseases, including Alzheimer's disease (AD), Parkinson's disease and prion diseases, are characterised by protein aggregation and deposition in specific brain regions. While the exact pathological significance of these aggregates remains to be conclusively resolved, the biology behind their formation is crucial to the understanding of the disease. Recent findings on the release and spread of several amyloid proteins, both luminal and cytosolic, suggest a model where these proteins can be released from affected cells in the form of amyloid seeds, and then re-enter other cells and aid in the spread of the disease. How are these aggregates released from the cells? Once released, how do they form plaques and propagate in the aqueous extracellular space to gain access to their host counterparts? How are cytosolic proteins and amyloids released from cells? We propose that exosomes, endocytically derived nanovesicles, are a major way to shuttle cytosolic proteins and amyloids out of the cell. Release of β-amyloid (Aβ) peptides on exosomes aids in the plaque formation. We provide evidence that amyloids involved in neurodegeneration, such as Aβ, are released via exosomes and that exosome-associated amyloids act as seeds for plaque formation.

**Microglia convert inert Abeta aggregates into neurotoxic amyloid-species through the shedding of microvesicles**

Claudia Verderio^1^, J. Pooja^2^, E. Turola^3^, A. Ruiz^2^, A. Bergami^4^, G. Magnani^4^, G. Comi^4^, D. Dalla Libera^4^, G. Legname^5^, R. Ghidoni^6^, R. Furlan^4^ and M. Matteoli^7^

^1^CNR Institute of neuroscience, Italy; ^2^Department of Biotechnology and Translational Medicine, Milan, Italy; ^3^Department of Biotechnology and Translational Medicine, Milano, Italy; ^4^INSPE, Division of Neuroscience, HSR, Milan, Italy; ^5^SiSSA, University of Trieste, Italy; ^6^IRCCS Fatebenefratelli, Brescia, Italy; ^7^IRCCS Humanitas Rozzano, Milan, Italy

*Introduction*: We have shown that production of microvesicles (MVs) from microglia increases during inflammation and that MVs potentiate neurotransmission. As Alzheimer's disease (AD) pathology is characterised by progressive microgliosis and neuronal loss, aim of this study was to investigate whether reactive microglia may use MVs to mediate neuronal damage. *Methods*: MVs were isolated by centrifugation from the medium of primary microglia or the cerebrospinal fluid (CSF) collected from AD patients. MVs were then incubated with Ab1-42 for 10 h and exposed to cultured neurons for 1h. Neuron viability was assessed by monitoring calcium concentration and annexin-V/PI staining. Soluble Ab1-42 oligomers were quantified by analysing Ab 1-42-488 binding to neurons, while Ab1-42 aggregates were measured by the thioflavin assay. Quantification of myeloid MVs in the CSF was performed by flow cytometry. *Results*: We found that Ab1-42 pre-incubated overnight with MVs, but not Ab1-42 or MVs alone, extensively bind neuronal processes and affect the number of viable neurons. Dendrites exposed to Ab1-42-treated-MVs had a fragmented appearance and displayed abnormal calcium concentration. Interestingly, neuronal damage was strongly inhibited by NMDA antagonists, by antibodies to soluble amyloid species and by the amyloid-interacting protein PrP. This suggests that MVs facilitate generation of toxic soluble amyloid species, which activate NMDA channels and trigger excitotoxicity. Notably, higher levels of microglial MVs were detected in the CSF of AD patients as compared to sex- and age-matched healthy controls, and similar neurotoxic damage was induced by human MVs upon pre-incubation with Ab 1-42. *Conclusions*: These data indicate that production of microglia-derived MVs is high in AD patients and that MVs enhance Ab1-42 toxicity, thus providing a new link between microgliosis and AD degeneration. The identification of MVs as a novel mechanism by which reactive microglia contribute to AD pathogenesis may open new therapeutic strategies.

**The role of exosomes in Parkinson's disease**

Antony Cooper^1^, S.M.Y. Kong^1^, B.K.K. Chan^1^, J.S. Park^2^, K.J. Hill^1^ and C.M. Sue^2^

^1^Garvan Institute of Medical Research, Australia; ^2^Kolling Institute, Australia

Parkinson's disease is a chronic, progressive, complex and debilitating neurodegenerative disorder that results primarily from the selective loss of midbrain dopaminergic neurons, although more widespread pathology can occur, especially at later stages of the disease. Although the molecular mechanisms underlying Parkinson's disease are unknown, considerable evidence supports the involvement of the cytosolic protein alpha-synuclein in a central causative role. During the course of the disease, alpha-synuclein aggregates are detected in a topographically predictable manner as the pathology “spreads” through anatomical connections throughout the brainstem, limbic and autonomic systems and neocortex. This pattern, in conjunction with the discovery of extracellular alpha-synuclein in cultured cell media, blood and cerebrospinal fluid, has led to the proposal of extracellular transmission of toxic alpha-synuclein aggregates between neurons as a basis for the neurodegenerative progression of Parkinson's disease. We have discovered a protein whose expression level can modulate the extent of externalised alpha-synuclein associated with exosomes, potentially by influencing exosome biogenesis. Ongoing studies are addressing the role this protein plays in Parkinson's disease.

**Infectious prions are associated with intact, morphologically distinct exosomes: implications for other neurodegenerative diseases**

A. Hill, B. Guo, L. Cheng, C. Quek, E. Hanssen, V.A. Lawson, S.A. Bellingham and B.M. Colemasn

University of Melbourne, Australia

*Introduction*: Neurodegenerative diseases affecting humans, such as Alzheimer's, Parkinson's and prion diseases, are associated with the misfolding and spreading of neurotoxic forms of the pathogenic proteins involved in each of these disorders. Prion diseases arise by the misfolding of the prion protein (PrP) which gains a unique infectious property and has been shown to transfer prion infection in association with exosomes. We have shown that prion infection alters the ultrastructure and miRNA content of exosomes, suggesting the cargo of these vesicles can alter the morphology and genetic content of the vesicles. *Materials and methods*: We have used cryo-electron microscopy, infectivity bioassays, and next generation sequencing from highly purified preparations of exosomes derived from control, prion infected, and cured infected cells to investigate the components of these vesicles required for the transmission of prion infection to new cells. RNAi and chemical inhibitor approaches to knock down components of the exosome biogenesis pathways were used to determine their effects on prion infection. *Results*: We find that curing prion infected cells results in morphological changes in the ultrastructure of exosomes to resemble those of uninfected cells, suggesting the infectious pathway affects the biogenesis of exosomes. Furthermore, disruption of the structure of exosomes using chemical or physical methods affects the levels of prion infectivity, indicating intact vesicles are required for the successful transfer of infection. Sequence analysis of the RNA content of vesicles derived from highly purified exosome preparations also demonstrates alterations in the levels of specific miRNA profiles between control and infected cells. *Conclusions*: Our results have implications for other neurodegenerative diseases, such as Alzheimer's and Parkinson's disease in which the pathological proteins (Aβ and α-synuclein, respectively) have also been associated with exosomes.

**The use of CSF microvesicle RNA for diagnosis of Alzheimer's disease**

J. Skog^1^, C. Romain^1^, P. Scheltens^2^, C.B. Oudejans^2^, S. Belzer^1^ and Teunissen^2^

^1^Exosome Diagnostics Inc., New York, NY, USA; ^2^VU Medical Center, Amsterdam, The Netherlands

*Introduction*: This work follows on the heels of our prior clinical studies identifying tumour-specific markers and mutations in plasma and cerebrospinal fluid (CSF) exosomes for the diagnosis and monitoring of patients with cancer. We have found that CSF is a stable source of intact miRNA and mRNA that can be used for gene expression profiling. There is a critical need for prognostic and predictive biomarkers in the neurodegenerative space, including Alzheimer's disease (AD). Current diagnosis of AD relies largely on documenting mental decline, but is not conclusive until later stages of the disease, when the patients already have severe damage in the brain. Early diagnosis could lead to treatment of the disease in its preliminary stage, as well as to be used to monitor disease progression. *Methods*: We have developed a platform to reproducibly extract high-quality CSF microvesicle RNA that can be used for RNA profiling from frozen biobanked CSF. After validation of the platform for both mRNA and miRNA, microvesicle RNA was extracted from 10 AD patients and 10 age-matched patients with subjective memory complaints (SMC). The patients were previously analysed for Tau, pTau and A?42 and subjected to a mini mental state exam. The CSF samples were processed in a blinded fashion and the microvesicle RNA was profiled on the OpenArray for 758 miRNAs. *Results*: Over a hundred miRNAs were robustly detected in each sample. There was a general downregulation of the miRNAs in the AD patients and several miRNAs seemed to be dysregulated. This study shows that CSF microvesicles can be robustly extracted and used for RNA profiling. In addition, several miRNAs show promise to be able to differentiate AD patients from patients with SMC.

**Parallel Oral Sessions 31-33 10:30-12:00**

**Oral Session 31 (Imperial Ballroom): Cancer: cell-to-cell communication April 20**

**Chair: *J. Rak and A. Clayton* 10:30-12:00**

**MicroRNAS in glioma biology**

Ennio Antonio Chiocca, Daisuke Ogawa, Pierpaolo Peruzzi, Sean Lawler, Agnieszka Bronisz and Jakub Godlewski

Harvey Cushing Neuro-Oncology Laboratories, Department of Neurosurgery, Brigham and Women's Hospital, Harvard Medical School, Boston, MA, USA

Our laboratory has focused on the role of microRNAs in glioma biology. We have employed several models to study the global expression profile of microRNAs in gliomas. In particular, we have been studying 2 microRNAs, one which plays a role in glioma stem-like cell self-renewal (miR-128) and one which contributes to glioma cell decisions related to migration vs. proliferation (miR-451). In the former example, we now can show that miR-128 loss is an early event in glioma genesis that modulates the function of the polycomb repressor complex (PRC), a major epigenetic regulator. In the latter, we can show that miR-451 levels fluctuate in response to nutrient availability and that enforced expression of miR-451 renders glioma cells more sensitive to chemo-radiation. In vivo, miR-451 expression correlates significantly with an invasive phenotype. These findings illustrate how transitions from global analyses of microRNA expression to single microRNA functional analyses can provide novel and valuable insights into glioma biology and provide possible relevance to roles of microRNAs delivered by tumour extracellular vesicles.

**Understanding the role of tumour-secreted exosomes in tumour microenvironment education**

Hector Peinado

Weill Cornell Medical College, USA

Historically, researchers have focused on tumour phenotype and genotype in seeking strategies that block tumour metastasis. However, evidence accumulated in the past decade supports a crucial role for secreted factors priming cells in the surrounding tumour microenvironment as critical components regulating primary tumour growth and metastatic behaviour. Recent findings of microvesicle-based information transfer processes by exosomes promise to revolutionise our understanding of tumour progression and the role educating the tumour microenvironment. We have recently found a novel role for tumour-secreted exosomes promoting a pro-metastatic phenotype of bone marrow progenitor cells through the MET oncogene in a process called “education”. Our data suggest that c-MET-dependent activation of signalling pathways influence the behaviour of cells within the tumour microenvironment. Importantly, the analysis of plasma-circulating exosomes in melanoma patients demonstrated that they contain specific signatures being critical factors in diagnosis and prognosis of melanoma patients. Our studies suggest that specific oncogenes shed on tumour exosomes dictate the behaviour of the tumour microenvironment favouring metastasis. Targeting specific tumour-derived exosome cargo will contribute to the reduction of metastases providing a rationale for development of targeted therapeutic approaches.

**Collateral cell transformation by exosome-like extracellular vesicles harbouring mutant H-RAS oncogene**

T.H. Lee, L. Montermini, B. Meehan, N. Magnus, D. Garnier and J. Rak

Montreal Children's Hospital Research Institute, Montreal, QC, Canada

*Introduction*: Cancer cells emit membrane-derived organelles referred to as extracellular vesicles (EVs). EVs have been implicated in the intercellular communication, as well as in cancer invasion, metastasis, angiogenesis and activation of the coagulation system. Cancer-derived EVs contain intact oncogenes (e.g. EGFR or Ras), and they may be taken up by other cells, whereupon they induce transformation-like changes. We asked whether transfer of EVs containing mutant H-ras would lead to transient or permanent changes in the phenotype of susceptible cells. Here we show the latter to be the case. *Materials and methods*: Ras-containing EVs (oncosomes) were collected from Ras-3, an intestinal epithelial cell (IEC-18) line that has been transfected with a V12 mutant active c-H-ras human oncogene. Ras-containing oncosomes were incubated with non-transformed immortalised fibroblasts (RAT-1), which were then analysed over time for mutant Ras (protein and DNA) content, colony formation ability and the expression of VEGF, a known target of Ras and a mediator of angiogenesis. *Results*: Oncosomes contained both Ras protein and DNA, the former of which was also detected in recipient RAT-1 cells for at least 7 days. RAT-1 cells harbouring EV-derived Ras triggered the expression of Ras target genes (e.g. VEGF) and acquired features of permanent cellular transformation, such as a more spindle-shaped morphology, and formation of foci in long-term monolayer cultures. Clones of RAT-1 cells isolated from such foci exhibited permanent content of mutant H-ras sequences and retained their transformed phenotype in passage. *Conclusions*: Our findings suggest that EVs derived from viable cells may carry intact mutant H-ras sequences and can mediate the transfer of this oncogene into susceptible normal cells, causing their long-term (permanent) transformation. Whether such EV-mediated spreading of oncogenes is a part of tumour pathogenesis is discussed.

**Differential protein expression and miR content of sorted subsets of circulating microvesicles from cancer patients and healthy controls**

S. Smith, D. Holterman, K. Brown, J. Duncan, J. Zhong, A. Stark, M. Millis, T. Tinder and D. Spetzler

Caris Life Sciences, Irving, TX, USA

MicroRNAs (miRs) are small non-coding RNAs that are 20 to 25 nucleotides in length and regulate the expression of entire families of genes. A major source of circulating miRs in cancer patients is believed to be circulating microvesicles (cMV) within biological fluids such as blood. The transfer of these modifiers of RNA translation from diseased cells into the bloodstream can have broad impacts on disease detection, progression and/or prognosis. The goal of these studies was to determine whether there are differences in miR composition within different subpopulations of cMV, based on surface protein composition. We used flow cytometry to phenotype and sort plasma-derived cMV from 20 individuals (3 breast cancer, 2 lung cancer, 6 prostate cancer, 1 bladder cancer and 6 non-cancer controls). cMV were stained for proteins associated with membranes, such as tetraspanins (CD9, 63, 81) Ago2 and/or GW182 using a Beckman Coulter MoFlo XDP. For phenotypic analysis, events were gated on tetraspanin expression to distinguish cMV from nano-sized irrelevant debris, and co-expression of GW182 and Ago2 was determined. Quadrant-based sorting was performed for single- and double-positive events. miR content was determined using conventional Taqman probes with the ABI 7900 thermal cycler on extracted RNA from the sorted cMV. The results of these studies demonstrate that unfractionated cMV were not able to discriminate cancers from non-cancers using miRs-let-7a, -16, -22, -148a or -451 in this population of patients. When sorted tetraspanin+, Ago2+ and/or GW182+ populations of cMV were compared, miR expression was generally 5-fold higher in cancer patients than in healthy controls. These studies demonstrate that cMV can be consistently phenotyped, analysed and sorted using a flow cytometer, and subpopulations of cMV contain unique miR profiles which can be useful in distinguishing cancer plasma from non-cancer plasma.

**Microvesicles shed from DIAPH3-silenced, amoeboid prostate cancer cells enhance growth of other tumour cells and suppress proliferation of immune cells**

Jayoung Kim^1^, S. You^1^, S. Morley^2^ and M.R. Freeman^1^

^1^Cedars-Sinai Medical Center, Los Angeles, CA, USA; ^2^Boston Children's Hospital, Boston, MA, USA

*Objectives*: Metastatic castration-resistant prostate cancer (mCRPC) is a leading cause of death in American men, but mechanisms controlling progression to advanced disease are still poorly understood. Genomic loss of the cytoskeletal regulator DIAPH3 occurs frequently in mCRPC. DIAPH3 silencing in prostate cancer and other cell background evokes a transition from a mesenchymal to an amoeboid phenotype, as well as increases tumour cell migration, invasion and metastasis. The main objective of this study was to identify potential biological functions of microvesicles (MV) produced by prostate cancer cells, in which DIAPH3 was silenced. *Methods*: The NanoSight platform was used to assess the degree of MV shedding from DIAPH3 knockdown DU145 cells (DIAPH3 KD) and controls. Shed MVs were harvested from conditioned medium using an established ultracentrifugation protocol. Western blot analysis, immunofluorescence cell staining and cell proliferation assays were used for functional assays. RNA profiling was performed to identify MV cargo. *Results*: DIAPH3 KD cells secreted about 2-fold more MVs than control DU145 cells. MV shedding was increased by the activation of EGFR-Erk/MAPK signalling by a potent growth factor, HB-EGF and/or p38MAPK inhibition. MV preparations from DIAPH3 KD cells promoted cell proliferation and invasive behaviours of recipient cells and suppressed proliferation of human immune cells, suggesting a potential impact of MV shedding on cancer epithelia as well as the tumour microenvironment. We also found that MVs contained micro RNAs (miRNAs) linked to malignant behaviour and disease progression. *Conclusions*: Our findings suggest that MVs produced in mCRPC by the loss of DIAPH3 expression may condition the tumour microenvironment, by activation of cancer cells and suppression of tumour-infiltrating immune cells.

**Oncogenic H-Ras reprograms Madin–Darby canine kidney (MDCK) cell-derived exosomal proteins during epithelial–mesenchymal transition**

Richard Simpson^1^, B. Tauro^2^, R. Mathias^2^, D. Greening^2^, S. Gopal^2^ and H. Ji^2^

^1^La Trobe Institute for Molecular Science, La Trobe University, Australia; ^2^La Trobe Institute for Molecular Science, La Trobe University, Australia

Commonly reported during early stages of development, wound healing and fibrosis, epithelial–mesenchymal transition (EMT) facilitates loss of epithelial cell architecture and acquisition of mesenchymal cell characteristics. Cancer cells at the leading tumour edge also undergo EMT, leading to increased aggressiveness, invasiveness and metastatic potential of carcinoma cells. While soluble-secreted proteins in the tumour microenvironment facilitate EMT progression, very little is known about the involvement of extracellular vesicles (EVs). To assess the contribution of EVs during EMT, we conducted a proteome analysis of highly purified exosomes released from Madin–Darby canine kidney (MDCK) cells transformed with oncogenic H-Ras (21D1 cells). Exosomes were purified using OptiPrep density gradient centrifugation and protein content was identified by GeLC-MS/MS. Comparative protein profiling of exosomes from 21D1 and parental MDCK cells revealed pronounced reprogramming of MDCK cell upon oncogenic H-Ras-induced EMT. Hallmark EMT protein markers observed in whole cells undergoing EMT correlate with those seen in exosomes. Constitutive expression of oncogenic Ras in epithelial MDCK cells induced exosome-mediated release of factors linked to promoting the metastatic niche, such as MMPs, annexins and integrins. Significantly, splicing and transcription factors were uniquely induced in 21D1 exosomes. Examination of exosomes from a panel of colorectal cancer cell lines harbouring KRAS or BRAF mutations also revealed the expression of a key transcription/splicing factor, observed in MDCK exosomes upon H-Ras-induced EMT. Our findings reveal that exosomes from oncogenic Ras-transformed cells are reprogrammed with metastatic niche factors, including transcription/splicing factors, which might be capable of inducing EMT in recipient cells.

**Oral Session 32 (Plaza Ballroom): Therapy-Stem Cells April 20**

**Chair: *N. Atai and B. Giebel* 10:30-12:00**

**Successful treatment of therapy-refractory acute graft-versus-host disease with mesenchymal stem cell-derived exosomes**

L. Kordelas^1^, V. Rebmann^2^, A.K. Ludwig^2^, S. Radtke^2^, P.A. Horn^2^, D. Beelen^1^ and B. Giebel^2^

^1^Department of Bone Marrow Transplantation, University Hospital, Germany; ^2^Institute for Transfusion Medicine, University Hospital Essen, Essen, Germany

*Background*: Graft-versus-Host Disease (GvHD) is a major cause of morbidity and mortality after allogeneic stem cell transplantation. Several studies reported a beneficial effect of systemically applied mesenchymal stem cells (MSCs) for preventing or treating acute GvHD. Apparently, MSCs secrete immune-modulatory factors, which impair inflammation and, thus help to suppress GvHD. Even though the nature of these factors remain elusive, small MSC-derived membrane vesicles, exosomes, might exert these functions. *Methods*: Aiming to treat a 22-year old female patient suffering from severe therapy-refractory cutaneous and intestinal GvHD Grade 4 with MSC-derived exosomes, exosomes from bone marrow-derived MSCs of four different unrelated stem cell donors were enriched. Upon comparing their immune modulatory properties *in vitro*, the exosome preparation with the strongest immune suppressive effect was selected. This preparation was administered in escalating doses into the patient. Impacts on the clinical symptoms and the immune response were documented. *Results*: Administration of the exosome preparation, with the strongest immune suppressive *in vitro* effect, to the GvHD patient was well tolerated. The cutaneous and intestinal GvHD symptoms improved significantly and the dosage of immunosuppressive agents – particularly of steroids – could be reduced. In line with this, a reduction of the pro-inflammatory cytokine response was observed during the course of the therapy. *Conclusions*: MSC exosome preparations exert immune suppressive functions *in vitro* and *in vivo*. Since the *in vivo* administration seems to be safe, MSC exosome administration appears to be a promising new treatment option for therapy-refractory GvHD.

**Targeted delivery of therapeutics with artificial bio-inspired nanovesicles that mimic exosomes**

S.C. Jang^1^, O.Y. Kim^1^, C.M. Yoon^1^, D.S. Choi^1^, T.Y. Roh^1^, J. Park^2^, J. Lotvall^3^, Y.K. Kim^1^ and Y.S. Gho^1^

^1^Department of Life Science, Pohang University of Science and Technology, Pohang, Republic of Korea; ^2^Department of Mechanical Engineering, Pohang University of Science and Technology, Pohang, Republic of Korea; ^3^Department of Internal Medicine, Krefting Research Centre, University of Gothenburg, Gothenburg, Sweden

*Introduction*: After discovering the natural function of exosomes in transporting RNA and protein, exosomes have been highlighted as the drug or siRNA delivery vehicles. However, low productivity of exosomes is one of the limitations for its clinical use. Therefore, the development of exosome-like vesicles with a substantially greater yield is attractive for future nano-sized drug delivery systems. Here, we developed artificial bio-inspired nanovesicles that mimic exosomes with high production yield. *Materials and methods*: Artificial nanovesicles were generated from monocytes/macrophages by serial extrusion, through a series of polycarbonate membranes in the absence or presence of various molecules, including chemotherapeutics, proteins, nucleic acids and nanoparticles. The targeted delivery of drug-loaded nanovesicles was examined on the TNF-alpha-treated human umbilical vein endothelial cells (HUVECs) or ICAM-1 expressing HT1080 cells *in vitro* and mouse CT26 tumour or human A549 tumour-bearing mice *in vivo*. *Results*: Artificial nanovesicles had similar characteristics as that of exosomes, but had more than 100-fold higher yield. Monocytes or macrophages-derived nanovesicles were specifically targeted to TNF-alpha-treated HUVECs and ICAM-1 expressing HT1080 *in vitro* and, efficiently delivered their cargos (including chemotherapeutics, RNase A, Q-dot, siRNA and GFP plasmid) into the target cells via receptor-mediated endocytosis. Furthermore, chemotherapeutics-loaded nanovesicles targeted to tumour endothelium, reduced primary and metastatic tumour growth without adverse effects in *in vivo* allograft and xenograft mouse tumour models. *Conclusions*: In this study, we have developed the artificial bio-inspired nanovesicles as potent drug delivery systems. Our experiments demonstrate a proof of the concept for the efficient delivery of therapeutics using exosome-like artificial nanovesicles.

**Exosomes with membrane-associated TGF-β1 from genetically modified dendritic cells inhibit experimental autoimmune encephalomyelitis in mice independent of MHC-restriction**

Zhijian Cai

Institute of Immunology, Zhejiang university, China

We have demonstrated exosomes from secreted TGF-β1 gene-modified dendritic cells (DCs) (sTGF-β1-EXO) delay the development of murine inflammatory bowel disease (IBD). In this study, we isolated exosomes from membrane-associated TGF-β1 gene-modified DCs (mTGF-β1-EXO) and found mTGF-β1-EXO are more immunosuppressive than sTGF-β1-EXO in vitro. Treatment with mTGF-β1-EXO, even after disease onset, inhibited the development and progression of myelin oligodendrocyte glycoprotein (MOG) peptide-induced experimental autoimmune encephalomyelitis (EAE). Treatment of mice with mTGF-β1-EXO impaired antigen-specific Th1 responses and IFN-γ production, but promoted IL-10 responses ex vivo. Treatment with mTGF-β1-EXO decreased the frequency of Th17 cells in EAE mice, which might be associated with the down-regulation of p38, ERK, STAT3 and NF-κB activation and IL-6 expression in DCs. Treatment with mTGF-β1-EXO increased the frequency of splenic and inguinal lymph node Tregs, and adoptive transfer of CD4+CD25+ T cells from the mTGF-β1-EXO-treated EAE mice dramatically prevented the development of EAE in the recipients. Moreover, treatment with mTGF-β1-EXO from C57BL/6 mice effectively inhibited the proliferation of T cells from BALB/c mice and proteolipid protein peptide-induced EAE in BALB/c mice. These indicate that mTGF-β1-EXO possess more powerful immunosuppressive ability and can effectively inhibit the development and progression of EAE in different strains of mice.

**Biochemical potential of MSC exosome**

Ruenn Chai Lai^1^, R.W.E. Yeo^1^, S.S. Tan^1^, B. Zhang^1^, Y.J. Yin^1^, T.S. Chen^1^, S.K. Sze^2^, A. Choo^3^, F. Arslan^4^, D.P.V. de Kleijn^5^ and S.K. Lim^1^

^1^Institute of Medical Biology, Singapore; ^2^Nanyang Technological University, Singapore; ^3^Bioprocessing Technology Institute, Singapore; ^4^Utrecht Medical Center, The Netherlands; ^5^Cardiovascular Research Institute, Singapore

*Introduction*: Of the different stem cells in clinical trials, mesenchymal stem cells (MSCs) are presently the stem cells in the most advanced stage of clinical testing and for the widest range of indications. The ease of isolation from adult tissues, large ex vivo expansion capacity and apparent therapeutic efficacy in a wide range of disease indications have made MSCs the stem cell of choice for regenerative medicine. Clinical and animal studies have demonstrated that secreted trophic factors, and not stem cell differentiation, likely mediated much of the therapeutic efficacy of MSCs. This paradigm shift in the therapeutic mechanism of MSCs has started to transform MSC therapy from a cell- to biologic-based therapy. Our group has identified the exosome, a secreted membrane vesicle, as an active therapeutic factor in MSC secretion. In this study, we profiled the proteome of MSCs to elucidate the biochemical potential of MSC and its relevance to therapeutic activity. *Material and Methods*: The proteome of MSC exosome was profiled by LC-MS/MS, and the predicted biochemical activities of the proteome were validated by enzymatic and cellular assays. *Results*: Mass spectrometry revealed a large and varied protein cargo with the potential to regulate a wide array of biochemical and cellular processes. These include enhancing glycolysis to increase cellular ATP production and glycolytic intermediates for anabolic activities, inducing adenosine-mediated activation of survival kinases (e.g. ERK and AKT) via CD73, reducing complement activation through CD59 and reducing denatured protein through 20S proteasome. *Conclusions*: As these processes are fundamental, non-tissue-specific processes in ameliorating tissue injury and promoting tissue repair, MSC exosomes could potentially underpin the therapeutic efficacy of MSC in diverse disease indications. This would provide a rationale to transform present MSC-based therapies into MSC-exosome-based therapies.

**Microparticles from activated/apoptotic lymphocytes preserve endothelial cell integrity**

Ramaroson Andriantsitohaina, R. Soleti, T. Benameur, E. Lauret and M.C. Martinez

INSERM U1063, France

Microparticles (MPs), small membrane-derived fragments shed from various cell types during activation and/or apoptosis, represent a new class of biological information mediators. MPs generated from T lymphocytes undergoing activation and apoptosis exert a beneficial potential effect on the cardiovascular system through their dual capacity to increase nitric oxide and reduce reactive oxygen species production. This study investigated the effect of MPs on both the angiogenesis and apoptosis of human umbilical vein endothelial cells. MPs induced the formation of capillary-like structures in an in vitro model, by regulating cell proliferation. Both cell adhesion and expression of proteins involved in this process, such as Rho A, and phosphorylation of focal-activated kinase were increased by MPs, via a ROCK inhibitor-sensitive pathway. MPs increased mRNA and protein levels of pro-angiogenic factors as measured by qRT-PCR and western blot. MPs significantly attenuated the increase in reactive oxygen species (ROS) associated with Act D-induced apoptosis at early phase of apoptosis (2 h), by acting directly as ROS scavengers, owing to their ability to carry active antioxidant enzymes such as catalase and the 3 isoforms of the superoxide dismutase. Furthermore, the effects of MPs on the late phases of apoptosis were associated with the ability of these vesicles to increase the expression of manganese-superoxide dismutase through internalisation process. Interestingly, the effects induced by MPs on the formation of capillary-like structures, expression of adhesion molecules and pro-angiogenic factors and on apoptosis were reversed when Sonic Hedgehog signalling was pharmacologically inhibited with cyclopamine. These findings illustrate new mechanisms by which MPs from T lymphocytes exert their vasculo-protective effects by improving endothelial function under pathological conditions in which cell integrity is impaired.

**Membrane TRAIL-armed exosomes as a novel and effective anti-tumour therapy**

V. Huber^1^, P. Squarcina^1^, P. Bürdekx^1^, C. Chiodoni^1^, L. Botti^1^, B. Vergani^2^, E. Vergani^1^, A. Villa^2^, M.P. Colombo^1^, A.M. Gianni^1^ and L. Rivoltini^1^

^1^Fondazione IRCCS Istituto Nazionale dei Tumori, Italy; ^2^Universita degli Studi di Milano - Bicocca, Italy

*Introduction*: Exosomes deliver signals or molecules to target cells and serve as therapeutic tool. Since most cancer cells, in contrast to normal counterpart, show sensibility towards TRAIL-induced apoptosis, membrane TRAIL-armed exosomes could represent a more efficient tool for delivering death signals to the tumour, as compared to soluble TRAIL. *Material and Methods*: The poorly immunogenic erythroblastoid cell line K562 was stably transduced with a lentiviral vector containing membrane-bound TRAIL. Exosomes were isolated by differential centrifugations. TRAIL expression and exosomal nature were assessed by flow cytometry, ELISA, electronmicroscopy, Nanosight and western blot. Apoptosis was evaluated by Annexin/PI staining and Caspase 3 activation. *In vivo*, tumour necrosis and Ki67 expression were verified by immunohistochemistry on lesions of mice bearing TRAIL sensitive KMS11 or SUDHL4 tumours. *Results*: TRAIL transduced K562 cells secrete significant amounts of highly mTRAIL-positive exosomes which induce relevant apoptosis in SUDHL4 (80%) and KMS11 (40%) haematological cancers and melanoma cell lines in vitro. In vivo studies show that i.v. injection of TRAIL-exosomes induce significant necrosis in perivascular regions of KMS11 tumours. However, this is not paralleled by an equivalent impact on tumour growth, suggesting that the vascular damage may not be sufficient to halt progression of tumours displaying a limited sensitivity to TRAIL, such as KMS11. Preliminary experiments with SUDHL4 cells point to a potential impact on tumour growth of TRAIL-exosome adoptive transfer. Ongoing experiments with melanoma-bearing mice will reveal whether TRAIL exosomes could be also used to target solid tumours in vivo. *Conclusions*: TRAIL exosomes appear as an effective tool for inducing apoptosis of tumour cells. If this hypothesis would be confirmed in vivo, these nanovesicles might become a feasible tool for delivering pro-apoptotic signals to tumour site and contribute to disease control.

**Oral Session 33 (Terrace Room): Bacteria and Infection April 20**

**Chair: *M. Pegtel and E. Ligeti* 10:30-12:00**

**Neutrophil-derived antibacterial microvesicles are increased in bacteremic patients**

Erzsebet Ligeti^1^, C.I. Timar^1^, M.A. Lorincz^1^ and Z. Ivanyi^2^

^1^Department of Physiology, Semmelweis University, Hungary; ^2^Clinic for Anesthesiology and Intensive Care, Semmelweis University, Hungary

*Introduction*: We have discovered that upon stimulation with opsonised bacteria, isolated neutrophilic granulocytes (PMN) are able to produce microvesicles (MVs) that impair the growth of selected bacteria (Timar et al. Blood 2013). The question arises whether similar MVs are also produced in vivo, in healthy individuals or in infected patients. *Materials and methods*: MVs were prepared by centrifugation and filtration of human blood serum. PMN-derived MVs were detected on the basis of double-staining with antibodies against the PMN-specific surface markers CD11b and CD177. Aggregation of isolated stained MV with non-opsonised bacteria expressing green fluorescent protein was followed by fluorescent and confocal microscopy. *Results*: PMN-derived MVs were detectable in the serum of healthy individuals, but these vesicles did not form large aggregates with bacteria. In the serum of patients suffering from *S. aureus* infection, the number of PMN-derived MVs was increased fourfold, and ex vivo these vesicles formed large aggregates and clumped up to 40% of the prevailing bacteria. Clumping was inhibited by neutralising the anionic surface charge of MV, by antibodies blocking beta-2 integrins, by inhibition of cytoskeletal remodelling or signalling via PI3K. *Conclusion*: Circulating PMN-encountering bacteria in the blood are also able to produce MVs that impair bacterial mobility by aggregation.

Funded by: OTKA 75084 and TAMOP4.2.2/B10/1-2010-0013.

**Gut microbiota-derived extracellular vesicles are important on the development of inflammatory bowel disease**

Chil-sung Kang^1^, H.G. Moon^2^, S.G. Jeon^2^, Y.S. Gho^2^ and Y.K. Kim^2^

^1^Pohang University of Science and Technology, Pohang, Republic of Korea; ^2^Department of Life Sciences, Pohang University of Science and Technology, Pohang, Republic of Korea

*Introduction*: Inflammatory bowel disease (IBD) occurrence is increased annually by westernised diet. Gut of mammalian hosts is home to a diverse collection of microorganisms, collectively termed the gut microbiota, which have profound effects on human health. However, owing to the complexity of the gut microbiota, our understanding of the roles of commensal and pathogenic bacteria in establishing a healthy intestinal barrier and in IBD pathogenesis is evolving only slowly. Metagenomics, sequencing of DNA from an environmental sample, offers a powerful means to better understand the complex relationships between hosts and their bacterial inhabitants. Recently, we found that bacteria-derived extracellular vesicles (EVs) are causally related with inflammatory diseases. *Material and methods*: To make experimental colitis, 3% dextran sulfate sodium (DSS) was administered orally into female C57BL/6 mice for 5 days. EV was isolated from stools of DSS colitis and control mice. To evaluate the proportion of stool bacteria and bacterial EV, metagenome analysis was performed using indigenous 16S rDNA. To elucidate the effect of bacterial EV on the IBD pathogenesis, 2% DSS were administered orally with bacteria and bacterial EV. *Results*: Metagenomics of stool bacteria and bacterial EV showed that the proportion of bacteria was not different drastically between stools of colitis and control mice; however, the proportion of TM7 phylum in stools was markedly increased in colitis mice compared to control mice; the proportion of EV derived from *Bacteroides acidifaciens* and *Akkermansia muciniphila* was decreased drastically in stools from colitis mice. When *A. muciniphila*-derived EV was treated orally in DSS colitis mice, colitis index, such as the loss of body weight, diarrhoea and colon shrinkage, were ameliorated. *Conclusion*: Gut homeostasis may be related with gut microbiota-derived EV, and IBD can be developed in the absence of gut microbiota-derived EV, which maintain gut homeostasis.

**The effect of membrane vesicles from *Staphylococcus aureus* and *Staphylococcus epidermidis* on monocytes and macrophages**

Forugh Vazirisani, M. Trobos, X. Wang, Z. Mladenovic, A. Palmquist, O. Omar, K. Ekström and P. Thomsen

Department of Biomaterials, BIOMATCELL VINN Excellence Center of Biomaterials and Cell Therapy, University of Gothenburg, Gothenburg, Sweden

*Introduction*: Gram-negative bacteria produce outer membrane vesicles with the ability to deliver virulence factors to host cells. However, little is known on the ability of Gram-positive bacteria to release and communicate via membrane vesicles (MVs). The Gram-positive bacteria *S. aureus* and *S. epidermidis* are responsible for numerous hospital-acquired infections and biofilm formation on implanted biomaterials. It is, therefore, of interest to explore if MVs secreted from these bacteria have a role in such events. The aim of the present study was to examine whether *S. aureus* and *S. epidermidis* release MVs and, if so, whether these MVs can cause cell death of monocytes and macrophages. *Material and methods*: MVs were isolated from *S. aureus* and *S. epidermidis* cultures (109 CFU/ml) by centrifugation and filtration steps. The collected MVs were evaluated for size and morphology by electron microscopy and nanoparticle tracking analysis. MVs (10, 50 or 100 µg/ml) were added to THP-1 monocytes or PMA-differentiated macrophages. After 24h, cell death was evaluated using propidium iodide and flow cytometry. *Results*: Both *S. aureus* and *S. epidermidis* were shown to secrete MVs with a size of ~100–200 nm. The results of flow cytometry analysis showed a dose-dependent reduction in the viability of monocytes and macrophages when incubated with *S. aureus* MVs. The *S. aureus* MVs (≥50 µg/ml) induced a potent cytotoxic effect on both monocytes and macrophages with more than 50% dead cells compared to control. The MVs from *S. epidermidis* revealed a relatively less toxic effect. *Conclusions*: The results demonstrate that MVs are released from Gram-positive bacteria. The MVs from *S. aureus* were more pathogenic to monocytes and macrophages compared to *S. epidermidis*. The MVs from *S. aureus* and *S. epidermidis* provide a possible source of virulence factors and toxins to immune cells, which may contribute to subsequent cellular pathology and cell death.

**Inhibition of complement protein 5 reduces *Neisseria meningitidis*-induced microparticle-associated tissue factor activity as measured by thromboelastography in whole blood**

R.Ø. Øvstebø^1^, M.S.H. Hellum^1^, P.K. Kierulf^2^, P.B. Brandtzaeg^3^, T.E.M. Mollnes^4^ and C.E.H. Henriksson^1^

^1^Department of Medical Biochemistry, Oslo University Hospital, Oslo, Norway; ^2^Department of Medical Biochemistry, Oslo University Hospital and University of Oslo, Oslo, Norway; ^3^Department of Pediatrics, Oslo University Hospital and University of Oslo, Oslo, Norway; ^4^Department of Immunology, Oslo University Hospital and University of Oslo, Norway

*Background*: *Neisseria meningitidis* (*Nm*) causes meningococcal sepsis with massive activation of the coagulation and complement cascades. Lipopolysaccharides present in the outer membrane of *Nm* induce tissue factor (TF), the main initiator of blood coagulation. The anaphylatoxin C5a, a potent complement activation product, is also able to induce TF. We investigated the interplay between the complement and the coagulation system with special focus on *Nm*-induced TF-positive microparticles (MPs) by inhibiting the complement cascade with eculizumab, a monoclonal antibody inhibiting the cleavage of C5. *Methods*: Whole blood (anticoagulated with the specific thrombin inhibitor lepirudin) from healthy donors (n=7), exposed to 108 Nm/mL±eculizumab (Soliris^®^)100 µg/mL for 4 h (37°C) was sequentially centrifuged 2 times (2000×g,15 min, RT). Then, MPs were isolated from plasma and washed by repeated centrifugation 3 times (17000×g, 30 min, RT). Pelleted MPs were added to freshly drawn citrated whole blood containing corn trypsin inhibitor (CTI), and time to clot formation was examined using a thromboelastograph (ROTEM). *Results*: MPs obtained from whole blood exposed to 108 Nm/mL gave a significant shortening of the time to clot formation: 303 s±99 s (mean±SD) compared with MPs isolated from unexposed blood: 1015 s±210 s (p=0.001). Adding eculizumab in addition to *Nm* significantly lengthened the time to clot formation: 438 s±127 s (p=0.012) compared to MPs from *Nm* alone. Pre-incubating MPs obtained from *Nm*-exposed blood with anti-TF antibodies prolonged time to clot formation: 1049 s±122 s, indicating an MP-associated TF-dependent clot forming activity. The times to clot formation remained unaffected of eculizumab or bacteria alone. *Conclusion*: Inhibition of C5 reduced MP-associated TF-dependent time to clot formation in whole blood, implying that the complement and coagulation cascades are interplayers in this in vitro model mimicking meningococcal sepsis.

**Characterisation of exosomal RNA from *M. tuberculosis*-infected macrophages: potential use as molecular biomarkers for tuberculosis**

Jeff Schorey, P.P. Singh and L. Li

University of Notre Dame, USA

*Introduction*: Recently, it has been found that exosomes contain microRNAs and messenger RNA that can be transferred between cells affecting cellular activity. Importantly, the presence of exosomal RNA in body fluids, such as serum, urine and saliva, has suggested their potential use as biomarkers for various diseases, including cancer. However, the capability of exosomes in transporting genetic material in context of a tuberculosis (TB) infection has not been studied. *Methods*: The macrophage cell line Raw 264.7 was infected with *Mycobacterium tuberculosis* to reach approximately 80% infection, and the culture media was isolated 72 h post-infection. The culture media was filtered using a 0.22 micron filter to remove bacteria, cells and larger vesicles and centrifuged at 100,000xg to obtain an exosome-enriched vesicle population. *Results*: We characterised the exosomes released from *M. tuberculosis*-infected and uninfected Raw 264.7 cells for the presence of mRNA and microRNA with a hypothesis that the exosomes from infected macrophages might carry a unique subset of RNAs. We have identified mouse microRNAs in exosomes; however, quantitative analysis showed an overall down-regulation of microRNAs in exosomes released from infected macrophages. We also identified a subset of mRNA transcripts which differed between exosomes derived from *M. tuberculosis*-infected compared to uninfected macrophages. Most interestingly, we have identified mycobacterial transcripts in exosomes released from infected macrophages. *Conclusion*: Our results suggest that characterisation of exosomal RNA during an in vivo TB infection can potentially identify novel biomarkers for TB.

**Exosomes containing mycobacterial antigens induce a protective immune response against *M. tuberculosis* infection in mice**

Y. Cheng and J.S. Schorey

Department of Biological Sciences, University of Notre Dame, USA

*Introduction*: Tuberculosis caused by intracellular pathogen *Mycobacterium tuberculosis* remains one of the most deadly infectious diseases worldwide. Our previous studies showed that macrophages infected with *M. tuberculosis* release exosomes carrying mycobacterial lipids and proteins with highly immunogenic activities. In addition, it was found that exosomes isolated from macrophages infected with *M. bovis* BCG or pulsed with *M. tuberculosis* culture filtrate proteins (CFP) triggered significantly antigen-specific CD4+ and CD8+ cell-mediated immune responses in the lungs, spleens and mediastinal lymph nodes in mice after intranasal administration. Interestingly, the immune responses induced by purified exosomes were independent from the exogenous adjuvants, demonstrating that the exosomes have both immunogenic and adjuvant properties. *Materials and methods*: In exosome-priming experiments, wild type C57BL/6 mice were immunised with purified exosomes released by macrophages, and then analysed for antigen-specific immune responses or challenged with *M. tuberculosis* H37Rv to measure bacterial burden in the lungs and spleens. For BCG-priming and exosome-boosting experiments, mice were first vaccinated with *M. bovis* BCG and subsequently rested for 8 months before boosted with exosomes. *Results*: Exosomes purified from macrophages treated with *M. tuberculosis* culture filtrate protein not only primed a protective immune response, but also strongly boosted prior BCG-vaccinated mice against an *M. tuberculosis* infection as defined by decreased bacterial burden in the lungs and spleens, as well as decreased pathology. *Conclusions*: Our findings suggest that exosomes might serve as a novel cell-free priming or boosting vaccine against *M. tuberculosis* infection.

**Closing Session (Imperial ballroom)         12:15-13:30**

**Abstract Prizes                12:15-12:30**

**ISEV-2013 Highlights**

**Cell and Molecular Biology: M. Wauben         12:30-12:45**

**Clinical Applications: P. Quesenberry           12:45-13:00**

**Closing Ceremony/Presentation of ISEV-2014       13:00-13:3**

